# Impacts of dietary exposure to pesticides on faecal microbiome metabolism in adult twins

**DOI:** 10.1186/s12940-022-00860-0

**Published:** 2022-05-03

**Authors:** Robin Mesnage, Ruth C. E. Bowyer, Souleiman El Balkhi, Franck Saint-Marcoux, Arnaud Gardere, Quinten Raymond Ducarmon, Anoecim Robecca Geelen, Romy Daniëlle Zwittink, Dimitris Tsoukalas, Evangelia Sarandi, Efstathia I. Paramera, Timothy Spector, Claire J. Steves, Michael N. Antoniou

**Affiliations:** 1 Gene Expression and Therapy Group, King’s College London, Faculty of Life Sciences & Medicine, Department of Medical and Molecular Genetics, Guy’s Hospital, London, SE1 9RT UK; 2 Department of Twin Research and Genetic Epidemiology, King’s College London, London, UK; 3 Service de pharmacologie, toxicologie et pharmacovigilance, UF Toxicologie analytique environnementale et santé au travail, CHU de Limoges, Limoges, France; 4 Center for Microbiome Analyses and Therapeutics, Leiden University Medical Center, Leiden, The Netherlands; 5 Metabolomic Medicine Clinic, Health Clinics for Autoimmune and Chronic Diseases, 10674 Athens, Greece; 6 NEOLAB S.A., Medical laboratory, 125 Michalakopoulu Str, 11527 Athens, Greece

**Keywords:** Pesticides, Biomonitoring, Gut microbiota, Dietary habits, Food intake

## Abstract

**Background:**

Dietary habits have a profound influence on the metabolic activity of gut microorganisms and their influence on health. Concerns have been raised as to whether the consumption of foodstuffs contaminated with pesticides can contribute to the development of chronic disease by affecting the gut microbiome. We performed the first pesticide biomonitoring survey of the British population, and subsequently used the results to perform the first pesticide association study on gut microbiome composition and function from the TwinsUK registry.

**Methods:**

Dietary exposure of 186 common insecticide, herbicide, or fungicide residues and the faecal microbiome in 65 twin pairs in the UK was investigated. We evaluated if dietary habits, geographic location, or the rural/urban environment, are associated with the excretion of pesticide residues. The composition and metabolic activity of faecal microbiota was evaluated using shotgun metagenomics and metabolomics respectively. We performed a targeted urine metabolomics analysis in order to evaluate whether pesticide urinary excretion was also associated with physiological changes.

**Results:**

Pyrethroid and/or organophosphorus insecticide residues were found in all urine samples, while the herbicide glyphosate was found in 53% of individuals. Food frequency questionnaires showed that residues from organophosphates were higher with increased consumption of fruit and vegetables. A total of 34 associations between pesticide residue concentrations and faecal metabolite concentrations were detected. Glyphosate excretion was positively associated with an overall increased bacterial species richness, as well as to fatty acid metabolites and phosphate levels. The insecticide metabolite Br2CA, reflecting deltamethrin exposure, was positively associated with the phytoestrogens enterodiol and enterolactone, and negatively associated with some N-methyl amino acids. Urine metabolomics performed on a subset of samples did not reveal associations with the excretion of pesticide residues.

**Conclusions:**

The consumption of conventionally grown fruit and vegetables leads to higher ingestion of pesticides with unknown long-term health consequences. Our results highlight the need for future dietary intervention studies to understand effects of pesticide exposure on the gut microbiome and possible health consequences.

**Supplementary Information:**

The online version contains supplementary material available at 10.1186/s12940-022-00860-0.

## Background

Human exposure to pesticides has been linked to a variety of diseases triggered by acute intoxication [1], occupational exposures or residential proximity to pesticide applications [2–4]. Whether typical low levels of pesticide exposure stemming from dietary and domestic use can contribute to disease development is strongly debated. Nevertheless, adverse effects from chronic exposure during vulnerable periods like pregnancy are well known for some insecticides, such as organophosphates [5–7], DDT [8] and pyrethroids [9, 10].

Controversies around human health effects of pesticides largely originate from the limited ability of current risk assessment procedures employed by government regulatory agencies to predict chronic adverse effects. Animal model systems have been traditionally used to evaluate the toxicity of pesticides. However, toxic effects of pesticides are not always accurately detected in the battery of animal bioassays performed during precommercial stages of assessment. This is the case for neurodevelopmental toxic effects [11], cancer caused by early life exposures [12], as well as metabolic disorders and fertility problems caused by endocrine disruptors [13]. This also holds true for the consequences of pesticide exposure on the gut microbiota [14], which are of interest because of the large enzymatic repertoire harboured by gut microorganisms conferring them the ability to modify the toxicity of chemicals. In some cases, the toxicity of xenobiotics can be enhanced after direct chemical modification by the gut microbiome [15]. This has been linked to a variety of health outcomes locally in the gut, such as intestinal damage and severe diarrhea [16], but also at distant organ sites, such as for melamine-induced renal toxicity [17]. Xenobiotics can also affect human health indirectly by changing gut microbiome composition [18], decreasing the protective effects of some bacteria or modulating the production of bacterial metabolites [19, 20]. In addition, government regulators do not assess mixture effects of pesticide exposure, which animal studies reveal toxicity at doses where individual compounds show no adverse outcomes [21]. This further compromises the ability of current risk assessment procedures to predict any negative health outcomes from pesticide exposure.

Dietary habits have a profound influence on the metabolic activity of gut microbial species and their influence on human metabolism. Healthy dietary patterns strongly associate with gut microbiota profiles known to be cardiometabolic markers of health [22]. The gut microbiome responds rapidly to dietary changes [23]. Switching to an animal-based diet causes an increase in bile acid secretion to cope with the higher fat intake and this selects for bacteria that are resistant to bile acid. In contrast, switching to a plant-based diet favours bacteria, which can utilise plant polysaccharides. This modulates the capacity of gut bacteria to synthesize vitamins and cofactors [24]. Although the effects of varying levels of macronutrients on the metabolism of gut bacteria are increasingly studied, little is known about the effects of possible contaminants such as pesticides.

Recent initiatives have been launched to harmonise and aggregate pesticide biomonitoring data in the EU with the European Joint Program HBM4EU [25], in the US with the CDC’s National Health and Nutrition Examination Survey [26], or the French national programmes Elfe (French Longitudinal Study since Childhood) and Esteban (Environment, Health, Biomonitoring, physical Activity, Nutrition) [27]. However, no comprehensive biomonitoring of pesticide exposure has been undertaken to date in the UK population. The first aim of our project is to start to fill this crucial gap in our knowledge by studying the exposure to pesticides in 65 twin pairs in the UK.

Since animal studies are not always accurate predictors of chronic health risks from pesticide exposure, estimating population-level exposure by direct biomonitoring is becoming one of the most successful strategies to evaluate human health effects [28]. Association studies are increasingly performed to link chemical exposures with human disease development [28]. This strategy allowed linking exposure between the pesticide-derivative heptachlor epoxide and Type 2 Diabetes [29]. We also provided the first associations between urinary pesticide excretion, food frequency questionnaires, and the composition and function of the faecal microbiome determined by shotgun metagenomics and metabolomics. The combination of metagenomics and metabolomics has proven to be the method of choice to study the faecal metabolic environment [30], and to evaluate the disturbance of this ecosystem by pesticides [31]. This allows associations to be made between dietary factors, pesticide exposure, and faecal microbiome composition and function.

Our study revealed a widespread exposure to different insecticide residues while contamination by fungicides and herbicides was less frequent. Analysis of dietary choices further suggested that insecticide exposure was due to the ingestion of contaminated fruit and vegetables. Associations between pesticide excretion and faecal microbiome composition were detected, suggesting that pesticides can be metabolised by gut bacteria. Overall, our study lays the foundation for larger epidemiological as well as dietary intervention studies designed to assess the link between pesticide exposure and human health.

## Methods

### Participants and pesticide exposure estimation

Subjects were monozygotic twins enrolled in the TwinsUK cohort [32]. The St. Thomas’ Hospital Research Ethics Committee approved the study. All individuals provided informed written consent. Twins were selected based on their answers to a food frequency questionnaire (FFQ) modified to include a question on organic food consumption [33]. Our aim was to define two groups of individuals, one less likely to be exposed to pesticides than the other because of organic food consumption. Consumption of legumes, fresh fruits and vegetables were estimated using existing FFQ data following the EPIC-Norfolk guideline [34]. Relevant FFQ items were converted to grams consumed per week as previously described [33]. Responses to the additional question “Please indicate to what extent you consume, when available, organic fruits and vegetables?” were used as modifiers to estimate potential for pesticide exposure, with the per weekly gram consumption being multiplied by the relevant weight (Table S2). Individuals who responded that they did no eat fruits and vegetables were removed from analysis. This resulted in a proxy estimate for potential of pesticide exposure from the diet (hereafter referred to as ‘pesticide exposure’). Differential pesticide exposure between twin pairs was assumed for pairs with a > 1 standard deviation difference of estimated exposure and who fell within different categories. A total of 977, mostly female, twin pairs answered questions on organic food dietary intake from the TwinsUK questionnaire. Study inclusion criteria were as follows; 1) only monozygotic twin pairs, 2) discordance for organic food consumption. Among these 977 individuals, 65 twin monozygotic twin pairs were found to be discordant for organic food consumption. Thus, only these 65 twin monozygotic twin pairs were selected for inclusion in our study. An additional FFQ was completed by participants at the time of faecal and urine sample collection to eliminate any confounding effects of temporality on the association between pesticide exposures and patterns of faecal microbiome metabolism.

Geographic location of the individuals from this study was based on their postcode centroid. The 111 individuals with geographic location were from different UK regions, namely East Midlands (9), East of England (16), London (10), North East (2), North West (15), South East (30), South West (19), West Midlands (6), and Yorkshire and The Humber (4). The discrimination between rural and urban environments was established with the Land Cover Map 2015 (LCM, version 1.2), which was downloaded from the Centre for Ecology and Hydrology via the ‘Digimap’ portal. Individuals were considered as rural or urban based on their surrounding environment in a 1 km^2^ area.

### Pesticide screening in urine samples

A general pesticide screening in urine samples was undertaken to assess the presence of 186 pesticide residues in a highly multiplexed detection assay with a low detection limit of 0.1 μg/L per compound. This included residues from common insecticides, herbicides and fungicides, which are used in agricultural and domestic settings. The LC-MS/MS system included a Shimadzu NEXERA X2 series and 8060 triple quadrupole mass spectrometer. Identification and quantification of pesticides was performed in positive and negative mode using multiple reaction monitoring (MRM) of a quantification and additional qualifier ion. When a pesticide was detected, it was included in a follow-up targeted assay to accurately quantify urinary concentrations against a standard curve.

Glyphosate and AMPA were measured after derivatization with FMOC-Cl (9-fluorenylmethyl chloroformate) with a Shimadzu NEXERA X2 series and 8060 triple quadrupole mass spectrometer. Glyphosate ^13^C2^15^N was used as an internal standard (IS) and was purchased as a solution at 100 mg/L (LGC, UK). A total of six calibration standards of glyphosate and AMPA (LGC, UK) between the higher limit of quantification (LOQ) and the lower LOQ (namely, between 0.1 to 10 μg/L) were used for the calibration. Chromatographic separations were performed at 40 °C on a Kinetex C18 100A column (100 × 2.10 mm, 2.6 μm particles) (Phenomenex, France). Identification and quantification of glyphosate-FMOC and AMPA-FMOC were performed in negative mode using MRM of a quantifier ion (390.2/62.9 and 331.9/110.1, respectively) and an additional qualifier ion (389.9/168.1 and 331.9/62.9, respectively).

Pyrethroid metabolites were measured in urine after hydrolysis with β-glucuronidase (*Helix Pomatia*) with a Shimadzu LC-20 AD and AB SCIEX API 5500 QTrap triple quadrupole mass spectrometer. For this, 3-PBA ^13^C and trans-Cl_2_CA ^6^D were used as IS. Six calibration standards between the higher LOQ and the lower LOQ (namely, between 0.025 to 10 μg/L) were necessary for the calibration with 3-PBA, 4-FPBA, 2,2-dichlorovinyl-2,2-dimethylcyclopropane-1-carboxylic acid (Cl_2_CA) (cis and trans), and cis − 3-(2,2-dibromovinyl)-2,2-dimethylcyclopropane-1-carboxylic acid (Br_2_CA) Chromatographic separations were performed on a Atlantis T3 column (150 × 2.10 mm, 5 μm particles) (Waters, USA). Mobile phase A contained 0.1% formic acid and phase B included (95/5) methanol acidified with 0.1% formic acid and phase A. Identification and quantification of 3-PBA, 4-FPBA, Cl_2_CA (cis and trans) and Br_2_CA were performed in negative mode using MRM of a quantifier ion (213.0/92.9, 231.0/93.1, 208.9/36.9 and 342.9/80.8, respectively) and an additional qualifier ion (213.0/65.1, 231.0/65.1, 207.0/35.0 and 296.8/80.9, respectively).

Organophosphate metabolites (dialkyl phosphate, DAP) were measured in urine after extraction with ethyl acetate and diethyl ether with a Shimadzu NEXERA X2 series and 8060 triple quadrupole mass spectrometer. DMP ^6^D, DMTP ^6^D, DMDTP ^6^D, DEP ^10^D, DETP ^10^D and DEDTP ^13^C_4_ were used as IS. DMTP ^6^D, DMDTP ^6^D and DEDTP ^13^C_4_ (LGC, UK) at 97, 98 and 95% purity respectively. A total of 6 calibration standards between the higher LOQ and the lower LOQ (namely, between 2 to 100 μg/L) were necessary for the calibration for DMP, DMTP, DMDTP, DEP, DETP and DEDTP. Chromatographic separations were performed on an INERTSIL ODS3 column (100 × 2.10 mm, 5 μm particles) (GL Sciences INC., JAPAN). Identification and quantification of DMP, DMTP, DMDTP, DEP, DETP and DEDTP were performed in negative mode using MRM of a quantifier ion (125.4/63.1, 141.3/126.1, 157.3/112.1, 153.4/79.1, 169.4/95.1 and 185.3/157.2, respectively) and an additional qualifier ion (125.4/79.1, 141.3/96.1, 157.3/142.1, 153.4/125.1, 169.4/141.1 and 185.3/111.1, respectively).

Exposure to dithiocarbamates was detected by measurement of carbon disulfide (CS_2_) in urine after acid hydrolysis at high temperature. Benzene ^6^D (LGC, UK) was used as an IS. Five calibration standards between the higher LOQ and the lower LOQ (namely, between 10 to 500 μg/L) were necessary for the calibration with carbon disulfide. The HS-GC-MS system included a Perkin Elmer TurboMatrix HS 40 and a Shimadzu QP 2010 quadrupole mass spectrometer. Chromatographic separations were performed on a RTX1 column (30 m × 0.32 mm × 4 μm) (RESTEK, France). Carrier gas was helium. For separation, the temperature was increased from 50 °C to 200 °C in 9 min. Identification and quantification of carbon disulfide were performed in impact electronic ionization mode using SIM of a quantifier ion (75.9) and an additional qualifier ion (77.9).

More details of the methods describing the measurement of glyphosate, pyrethroid metabolites, organophosphate metabolites, and dithiocarbamates, are available as supplementary material.

### Faecal microbiota

Faecal samples were collected at home by the recruited volunteers and stored at King’s College London. All samples have been processed within 2 hours after reaching the laboratory. They were stored at − 80 °C without the addition of a stabilising agent. DNA was extracted from 100 mg faecal samples using the Quick-DNA Faecal/Soil Microbe Miniprep Kit (ZymoResearch) according to the manufacturer’s instructions. Minor adaptations were made as previously described [35] as follows: 1. bead beating was performed at 5.5 m/s for three times 60 seconds (Precellys 24 homogeniser, Bertin Instruments) and 25 μL elution buffer was used to elute the DNA, following which the eluate was run over the column once more to increase DNA yield. A negative control (no sample added) and a positive control (ZymoBIOMICS Microbial Community Standard, ZymoResearch) were processed for DNA extraction and subsequently sequenced. DNA was quantified using the Qubit HS dsDNA Assay kit on a Qubit 4 fluorometer (Thermo Fisher Scientific).

Shotgun metagenomics was performed under contract by GenomeScan (Leiden, The Netherlands). The NEBNext® Ultra II FS DNA module (cat# NEB #E7810S/L) and the NEBNext® Ultra II Ligation module (cat# NEB #E7595S/L) were used to process the samples. Fragmentation, A-tailing and ligation of sequencing adapters of the resulting product was performed according to the procedure described in the NEBNext Ultra II FS DNA module and NEBNext Ultra II Ligation module Instruction Manual. The quality and yield after sample preparation was measured with the Fragment Analyzer. The size of the resulting product was consistent with the expected size of approximately 500–700 bp. Clustering and DNA sequencing using the NovaSeq6000 platform was performed according to manufacturer’s protocols. A concentration of 1.1 nM of DNA was used. DNA sequencing data was acquired using NovaSeq control software NCS v1.6. All information regarding samples, quality checks, experimental procedures, and the resulting data that was generated is available as Supplementary File 1.

Metabolon Inc. (Durham, NC, USA) was contracted to conduct the metabolomics analysis for human faecal samples as previously described [31]. Each sample extract was analysed on four independent instrument platforms: two different separate reverse phase ultra-high performance liquid chromatography-tandem mass spectroscopy analysis (RP/UPLC-MS/MS) with positive ion mode electrospray ionisation (ESI), a RP/UPLC-MS/MS with negative ion mode ESI, as well as a by hydrophilic-interaction chromatography (HILIC)/UPLC-MS/MS with negative ion mode ESI. Raw data was extracted, peak-identified and QC processed using Metabolon’s hardware and software as previously described (DeHaven et al. 2010). Faecal metabolites were identified by comparison to libraries of authenticated standards with known retention time/index, mass to charge ratio, chromatographic and MS/MS spectral data. Peak area values allow the determination of relative quantification among samples (Evans et al. 2009).

### Urine metabolomics

The urine metabolomics analysis was performed as before [36] and is an adaptation of a protocol originally published by Tanaka and colleagues [37]. Briefly, a liquid-liquid extraction was first performed to extract the urine organic acids after mixing the sample with 2-ketocaproic and tropic acids as internal standards (both from Sigma Aldrich, St. Louis, MO, USA). Hydroxylamine hydrochloride (Sigma Aldrich) was added to oxidise 2-keto acids. N,O,-bis-(trimethylsilyl) trifluoroacetamide (Supelco Bellefonte, PA, USA) containing 1% trimethylchlorosilane (Supelco Bellefonte) was then added to convert organic acids to corresponding trimethylsilyl (TMS) ethers, required to impart volatility. Volatile TMS esters were separated by gas-chromatography. Detection was performed using an electron impact mass spectrometer in scan mode with a mass range between 50 and 550 m/z. Obtained spectra are compared with published spectra for the compounds of interest to achieve identification. The absolute quantification of organic acids is performed using the calibration curves of standard compounds to internal standard ratios. Concentrations were normalized to creatinine. The quality assurance of the Organic acids’ methodology was assessed by participation in the quality control scheme of the European Research Network for Diagnosis of Inherited disorders of Metabolism (ERNDIM): Qualitative urine Organic acids and Quantitative urine Organic acids [38]. Precision, linearity and recovery for this method has been published [39].

### Statistical analysis

Pesticide biomonitoring data are often left-censored because a proportion of the individual’s urinary concentrations are below the level of detection. Summary statistics for pesticide urinary concentrations were calculated using a maximum likelihood estimation with R package NADA v1.6–1 [40] when the number of left-censored values was below 50%. In case the number of missing values was too high (detection frequency < 20%), only the detection frequency was reported. The relationship between the Healthy Eating Index 2010 (HEI), or the pesticide exposure index created from the consumption of fruit and vegetables, with pesticide urinary concentrations was evaluated using regression equations for singly censored data using maximum likelihood estimation with R package NADA v1.6–1 [40].

Random Forest classification of the 124 urine samples in which glyphosate could be measured, was performed by using faecal microbiome parameters as predictors using R package Caret (version 6.0–84) [41]. Since the two classes were not balanced (58 non-organic food consumers and 66 organic food consumers), down-sampling was done prior to processing with the trainControl function. Input variables were scaled and centred. The optimisation of the number of variables for splitting at each tree node (mtry) was done with default parameters. Accuracy was estimated using repeated cross-validation (5-fold, repeated 10 times). The model was trained using 66% of the dataset while the quality of this model was evaluated using predicted sample classification of the remaining 34% of the dataset. The quality control metrics were calculated using the confusionMatrix function from Caret. This function calculates the overall accuracy along a 95% confidence interval, with statistical significance of this accuracy evaluated with a one-side test comparing the experimental accuracy to the ‘no information rate’.

Shotgun metagenomics datasets were analysed with Rosalind, the BRC/King’s College London high-performance computing cluster. First, data was pre-processed using the software package pre-processing v0.2.2 (https://anaconda.org/fasnicar/preprocessing). In brief, this package concatenates all forward reads into one file and all reverse reads into another file, and then uses trim_galore to remove Illumina adapters, trim low-quality positions and unknown positions (UN) and discard low-quality (quality < 20 or > 2 Ns) or too-short reads (< 75 bp). This software package also removes contaminants (phiX and human genome sequences) and ultimately sorts and splits the reads into R1, R2 and UN sets of reads. The microbiome of human faecal samples was analysed using MetaPhlan3 (v 3.0.14) [42] and Humann2 (v 0.10) [43] with the UniRef90 database to characterise composition and function.

While pesticides with a detection frequency over 80% were considered as continuous variables, those detected in 50–80% of the samples were dichotomized as detected/undetected as recommended by the European Human Biomonitoring Initiative (HMB4EU) and as previously described [44]. Pesticides with detection frequencies below 20% were not carried forward in the association study (Table 1). The association study was conducted with a linear-mixed model considering age as a covariate and family relationship as a random effect with MaAsLin (Microbiome Multivariable Association with Linear Models) 2.0 (package version 0.99.12) [45]. Metagenome taxa detected in less than 20% of the individuals were removed and 211 species were carried forward for the association analysis. The metabolome data was log-transformed, while the metagenome taxonomic composition was transformed using an arcsine square root transformation. The Benjamini–Hochberg method was used to control the False Discovery Rate (FDR) of the MaAsLin analysis. Shannon and Simpson diversity indices, and species richness, were calculated with the vegan R package version 2.5–6 [46].. Nonmetric multidimensional scaling of Bray-Curtis dissimilarity with stable solution from random starts, with axis scaling, was performed with vegan R package [46]. Statistical significance of Bray-Curtis dissimilarity differences according to pesticide residue levels was calculated by Permutational Multivariate Analysis Of Variance (PERMANOVA) with 1000 permutations [47].

**Table 1:** Summary statistics of pesticide residues detected in the urine samples. The summary statistics values (μg/L) were estimated using the maximum likelihood inference for left-censored values. <LOD indicates that a reliable value could not be estimated because less than 50% of the samples contained quantifiable amounts of a given compound. In addition to these compounds, fipronil sulfone was detected in 1 sample (LOD of 0.1 μg/L) but not quantified. * 6 samples were missing. LOD, limit of detection; DF, detection frequency; IQR, 5% and 95% quantiles

pesticide group	active ingredient	metabolite	LOD	DF	median	max	IQR
dithiocarbamates	dithiocarbamates	carbon disulphide (CS2)	5	10.8	<LOD	83	<LOD
pyrethroids	cypermethrin, permethrin, cyfluthrin	trans 2,2-dichlorovinyl-2,2-dimethylcyclopropane-1-carboxylic acid (Trans Cl2CA)	0.02	96.9	0.18	23.0	0.027–1.2
		cis 2,2-dichlorovinyl-2,2-dimethylcyclopropane-1-carboxylic acid (Cis-Cl2CA)	0.01	98.4	0.07	7.1	0.014–0.38
	most pyrethroids	3-phenoxybenzoic acid (3-PBA)	0.015	80.0	0.12	10.6	0.039–1.8
	cyfluthrin	4-Fluoro-3-phenoxybenzoic acid (4F-3-PBA)	0.015	10.0	<LOD	0.10	<LOD
	deltamethrin	cis − 3-(2,2-dibromovinyl)-2,2-dimethylcyclopropane-1-carboxylic acid (Br2CA)	0.015	95.4	0.077	1.64	0.014–0.42
organophosphorus	Unspecific of methyl- organophosphates, e.g., dimethoate, chlorpyrifos-methyl, azinphos-methyl, malathion, fenthion, phosmet	dimethylphosphate (DMP)	1	14.6	<LOD	51.6	<LOD
		dimethylthiophosphate (DMTP)	1	58.5	5.2	88.6	0.83–31.9
		dimethyldithiophosphate (DMDTP)	1	2.3	<LOD	36.5	<LOD
	Unspecific metabolite of ethyl- organophosphates e.g., chlorpyrifos, diazinon, ethion, coumaphos, terbufos	diethylphosphate (DEP)	0.5	75.4	2.5	180	0.35–17.4
		diethylthiophosphate (DETP)	1	1.5	<LOD	5.7	<LOD
glyphosate	glyphosate	glyphosate*	0.05	53	0.045	2.8	0.0025–0.84
		aminomethylphosphonic acid (AMPA)*	0.1	5.6	<LOD	1.4	<LOD
N,N-dialkylarylamides	DEET	N,N-Diethyl-meta-toluamide (DEET)*	0.1	11.3	<LOD	8	<LOD
neonicotinoid	imidacloprid	imidacloprid	0.05	1.6	<LOD	1.1	<LOD

## Results

**Fig. 1:**
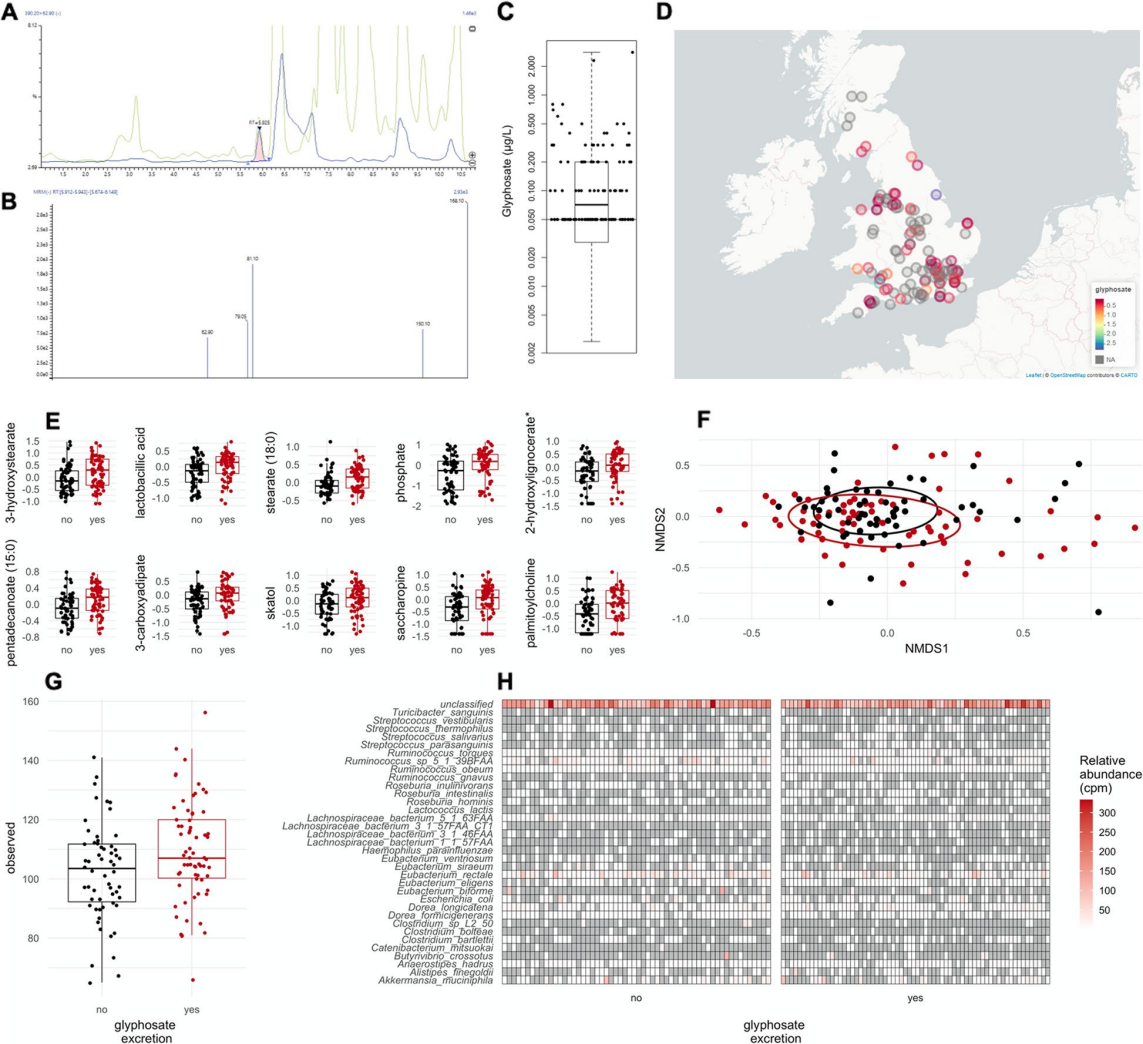
Association between urinary concentrations of glyphosate and faecal microbiota metabolism in 124 individuals. **A** Detection of glyphosate in a urine sample spiked with 0.1 μg/L of glyphosate. **B** MRM transition spectrum for the same sample. **C** Urinary glyphosate levels as a boxplot with the highest censoring threshold (LOQ) shown as a horizontal line. **D** Glyphosate levels according to living areas. **E** Faecal metabolites, which have the largest difference in abundance as box plots. **F** Beta diversity using Bray-Curtis dissimilarity. **G** Alpha diversity as the number of observed species. **H.** Relative abundance (as copies per million, cpm) for the bacteria contributing to the abundance (white to red: relative abundance; grey: undetected) of the shikimate pathway (as MetaCyc: chorismate biosynthesis I)

This observational study included 130 volunteers (93% woman, aged 63.8 ± 10.4 years). All were monozygotic twins enrolled in the TwinsUK cohort. Their BMI was 24.5 ± 4.3 kg/m2. We screened urine samples for the presence of 186 residues of insecticides, herbicides and fungicides. Pyrethroid and organophosphorus residues from insecticides were the most abundant pesticides detected in all urine samples, followed by DEET and imidacloprid (Table 1). The herbicide glyphosate was found in 53% of the urine samples although it was below the LOQ (< 0.1 μg/L) in 10 cases (8%) (Fig. 1A-C). Exposure to dithiocarbamates measured by the detection of carbon disulphide was found in 10.8% of the samples.

Our original objective was to assess if regular consumption of organic food products results in different urinary pesticide levels. A total of 65 monozygotic twin pairs discordant for organic food consumption (one twin eats an organic diet whereas the other does not) were selected for this investigation. However, when a new FFQ was performed at the time of urine collection, only 15 pairs were discordant for organic food consumption. In addition, in the majority of cases organic food formed only part rather than an exclusive component of the diet (Supplementary Table S3). The inconsistency in the answers provided to the nutrition questionnaire, an issue raised in other studies [48], convinced us to drop this component of the investigation, as any findings would be deemed as inconclusive. The observed inconsistency between the results of the FFQ before recruitment and during the study is an important finding, which supports the need for dietary intervention studies to accurately determine the effects of an organic diet on the gut microbiome.

We then evaluated if dietary habits are associated with the excretion of pesticide residues to understand if the consumption of fruit and vegetables is a major source of exposure in the UK using the Healthy Eating Index 2010 (HEI) and a pesticide exposure index created from the consumption of fruit and vegetables [33]. We noted that individuals who are regularly consuming organic products had higher healthy eating index values (P Wilcoxon = 0.02). This is not surprising because individuals who eat organic diets lead generally healthier lifestyles than individuals eating conventionally grown foodstuffs [49]. DMTP levels, a metabolite of methyl-organophosphates, was associated with fruit and vegetable consumption (p cenreg = 0.04). This suggested that organophosphate exposure is at least in part related to food contamination. Geographic location did not predict urinary pesticide levels across 9 UK regions (Fig. 1D and S1). Postcode was available for 123 individuals, which were stratified as 30 rural and 93 urban individuals. Similarly, we did not find a difference in pesticide excretion between rural or urban individuals (Fig. S2).

**Fig. 2:**
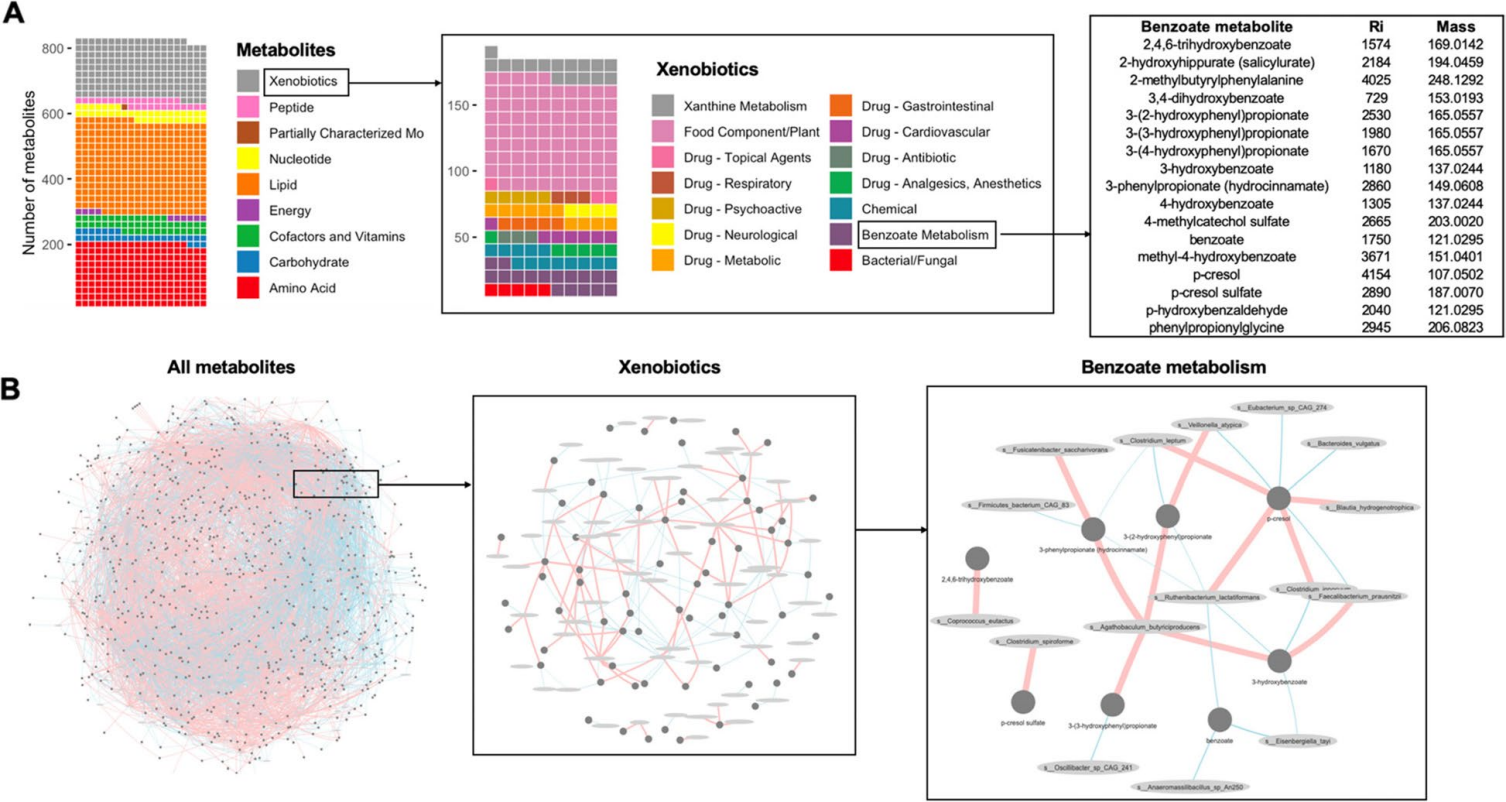
Deep phenotyping of the faecal microbiome in 130 individuals (65 twin pairs). **A** Faecal metabolomics allow the detection of a large number of metabolites from different classes, including xenobiotics**. B** Correlations between the abundance of gut microorganisms and metabolites (positive, red; negative, blue) inform on the interaction between environmental exposures and the metabolism of the gut microbiome

As dietary habits have a profound influence on the biochemical activity of gut microbial species and influence metabolism, the faecal microbiome of the 65 twin pairs was studied by shotgun metagenomics and metabolomics. Faecal metabolite profiles contained xenobiotics, including 84 food components and 46 compounds annotated as pharmaceuticals or pharmaceutical metabolites. In addition, we also detected a large number of endogenous compounds such as 197 amino acid derivatives, 30 carbohydrates, and 47 cofactors and vitamins, as well as hundreds of lipids, steroids, corticosteroids and endocannabinoids (Fig. 2A).

Taxonomic composition of the faecal microbiome was evaluated by counting clade-specific marker genes. Composition profiles of the faecal microbiomes were typical for Western developed countries, with the most represented taxa assigned to the phyla Firmicutes (56.8%) and Bacteroidetes (21.1%) among the microorganisms which could be classified by Metaphlan. In total, we identified 16 phyla including 603 species. These were mostly bacteria (493 species), and a few Archaea (4 species), eukaryotes (3 species) and viruses (103 species). Faecal microbiome composition showed high interindividual variation, as only 14 species (out of 26 with an average relative abundance over 1%) were present in 80% of the samples.

Information on both taxonomic and metabolite composition allows the study of faecal microbiome metabolism (Fig. 2B). We detected 6890 correlations between 708 faecal metabolites and 164 bacterial species with a false discovery rate (FDR) of < 0.2. Filtering out poorly correlated variables (*ρ* < 0.3) retained 1391 correlations. There were 122 negative and 82 positive correlations to xenobiotic metabolites. These can inform on the interaction between environmental exposures and metabolism of the gut microbiome. For example, N-(2-furoyl) glycine, a furan derivative originating from food subjected to high heat, negatively correlated with multiple species such as *Eggerthella lenta*, *Clostridium bolteae*, *Clostridium CAG 58* and *Flavonifractor plautii*. Our data also reveal information about correlations between the abundance of dietary bioactive compounds and the abundance of gut microorganisms. For instance, *Firmicutes bacterium CAG:110* was negatively correlated to 7-methylurate, paraxanthine, 3-methylxanthine, 1-methylxanthine, 1,7-dimethylurate, 3,7-dimethylurate and 1-methylurate. The abundance of the product of dietary polyphenol metabolism enterolactone was positively correlated to the abundance of *Ruminococcus callidus, Intestinibacter bartlettii, Bifidobacterium animalis* and *Coprococcus catus.* However, compounds known to be used as pesticides were not detectable.

Although the effects of macronutrients on gut bacterial metabolism is increasingly studied, little is known about the consequences of pesticide exposure despite the increasing number of laboratory animal studies showing perturbations of the gut microbiome [14]. We evaluated the association between pesticide excretion and the composition of the faecal microbiome (Supplemental Excel Tables). A total of 34 associations between urinary insecticide residue concentrations and faecal metabolite concentrations had an FDR below 0.2 (Table 2). The insecticide metabolite Br2CA, reflecting deltamethrin exposure, was positively associated with the phytoestrogens enterodiol and enterolactone, as well as negatively associated with some N-methyl amino acids (N-methylalanine, N-methylglutamate, N-2-methylarginine and N-acetyl-1-methylhistidine) (Table 2). *Bacteroides eggerthii* and *Clostridium symbiosum* were positively associated to the metabolism of pyrethroids (Table 2). Although associations between single pesticide metabolites and the faecal microbiota were limited, the total urinary molar sums of the dimethyl-containing (sumDMP) and diethyl-containing (sumDEP) metabolites were positively associated to several *Clostridium* spp. (Table 2). Insecticide levels did not significantly influence microbiome diversity in most cases (Table S1), and only the levels of DMP was positively associated to the number of observed species.

**Table 2:** Significant associations between urinary excretion of pesticide residues and the composition of the faecal microbiota evaluated using shotgun metagenomics and metabolomics. Statistical models were established with MaAsLin2, using the pesticide levels as predictors. The model coefficient value (effect size) and the standard error from the model are reported along the *p*-values and its False Discovery Rate (FDR). Associations with FDR < 0.2 for creatinine adjusted models are reported

Response	Predictor	effect size	stderr	pval	FDR
	**METABOLOMICS**				
Br2CA	N-methylglutamate	−0.15	0.04	0.0009	0.12
	enterolactone	0.17	0.05	0.0011	0.13
	N-succinyl-isoleucine	−0.12	0.04	0.0012	0.13
	palmitoyl-ethanolamide	−0.18	0.05	0.0017	0.13
	N-methylalanine	−0.20	0.06	0.0019	0.13
	2-hydroxyglutarate	−0.16	0.05	0.0026	0.14
	N-acetyl-1-methylhistidine.	−0.18	0.06	0.0026	0.14
	enterodiol	0.19	0.06	0.0030	0.14
	N2-methylarginine	−0.07	0.02	0.0038	0.16
	piperidine	−0.15	0.05	0.0041	0.16
	stearoyl-ethanolamide	−0.21	0.08	0.0060	0.17
	2-oxo-1-pyrrolidinepropionate	−0.08	0.03	0.0061	0.17
	dihydroferulate	−0.12	0.04	0.0073	0.19
	1-palmitoyl-2-arachidonoyl-GPC	0.11	0.04	0.0076	0.19
Trans.Cl2CA	oleoyl.linoleoyl.glycerol	0.23	0.07	0.0013	0.15
	linoleoyl-linoleoyl-glycerol	0.23	0.07	0.0014	0.15
	4-hydroxybenzoate	−0.14	0.05	0.0020	0.15
	glycerol	0.25	0.08	0.0023	0.15
	1-oleoyl-GPC18	0.25	0.08	0.0031	0.19
	anacardic.acid	0.30	0.10	0.0034	0.19
3.PBA	arachidoylcarnitine	−0.25	0.07	0.0005	0.12
	carotenediol	−0.13	0.04	0.0006	0.12
	3-methylurate	0.18	0.05	0.0007	0.12
	allantoin	−0.27	0.08	0.0007	0.12
	erucoylcarnitine	−0.23	0.07	0.0008	0.12
	lysine	−0.11	0.04	0.0027	0.18
glyphosate	3.hydroxystearate	0.51	0.14	0.0003	0.19
	lactobacillic acid	0.25	0.07	0.0010	0.19
	stearate	0.25	0.07	0.0010	0.19
	phosphate	0.41	0.12	0.0011	0.19
	2-hydroxylignocerate.	0.33	0.10	0.0011	0.19
	pentadecanoate	0.24	0.07	0.0013	0.19
	3-carboxyadipate	0.27	0.08	0.0019	0.19
	saccharopine	0.34	0.11	0.0019	0.19
	**METAGENOMICS**				
Br2CA	*Faecalitalea cylindroides*	0.06	0.0002	1.3E-30	1.6E-28
	*Streptococcus_anginosus_group*	0.11	0.03	0.003	0.19
DEP	*Hungatella hathewayi*	−0.03	7.3E-5	2.0E-33	9.5E-31
	*Collinsella aerofaciens*	−0.19	0.007	2.65E-08	4.2E-6
	*Clostridium bolteae*	0.04	0.01	0.002	0.19
PBA	*Bacteroides eggerthii*	0.16	0.01	3.9E-8	1.9E-5
	*Clostridium symbiosum*	0.13	0.02	6.9E-5	0.02
sumDEP	*Hungatella hathewayi*	−0.03	7.3E-5	2.2E-33	1.1E-31
	*Collinsella aerofaciens*	−0.18	0.007	2.7E-8	4.2E-6
	*Clostridium bolteae*	0.05	0.01	0.0009	0.07
	*Enterorhabdus caecimuris*	0.01	0.004	0.001	0.1
sumDMP	*Clostridium citroniae*	0.02	2.8E-5	3.8E-43	5.49E-41
	*Veillonella dispar*	0.002	3.9E-5	2.0E-22	2.2E-20
	*Clostridium bolteae*	0.08	0.002	0.001	0.07
	*Clostridium innocuum*	0.14	0.04	0.001	0.07
	*Eubacterium siraeum*	−0.13	0.04	0.004	0.16

We recently described a metabolomic signature for glyphosate exposure in the gut microbiome of rats [31]. We thus evaluated if there was a correlation between glyphosate urinary levels and faecal microbiome composition and function in the TwinsUK cohort. The 10 metabolites, which discriminate glyphosate-exposed rats from unexposed animals did not significantly predict the detection of glyphosate in the 124 individuals with a classification accuracy of 65% (95% CI [0.49, 0.79]) (Fig. S3). Positive associations to glyphosate levels mostly included fatty acid metabolites (Fig. 1E). Microbial composition measured by Bray-Curtis dissimilarity was no different between individuals who excreted glyphosate and those for whom glyphosate was undetected (Fig. 1F). A linear-mixed model considering age and sequencing depth as a covariate, and family relationship as a random effect, showed that the species richness was higher in individuals who excreted glyphosate (p lmer = 0.01) (Fig. 1G) but not for Shannon (p lmer = 0.17) and Simpson diversity indices (p lmer = 0.60). The positive association between phosphate and glyphosate (Fig. 1E) points to an influence on phosphate metabolism, which could be due to microbial metabolism of glyphosate [31]. We also estimated the relative contribution of the different bacterial species to core functions of the gut microbiome. The relative abundance of genes from the shikimate pathway among bacteria, was no different between the individuals who excreted glyphosate and those for whom glyphosate was undetected (Fig. 1H).

We performed a urine metabolomics analysis to evaluate whether pesticide urinary excretion also associates with physiological changes [36]. This consisted of a targeted analysis of 36 organic acids in a subgroup of 61 subjects. No differences were detected in the concentrations in organic acids between a group of 28 individuals who did not present detectable glyphosate levels in their urine compared to a group of 33 individuals with detectable glyphosate levels (Table S3). Overall, no associations between pesticide residues and the composition of the urine metabolome were found (Supplemental Excel Tables).

## Discussion

The consequences of pesticide exposure on human gut microbial community composition, function and metabolic health are currently unknown. This is despite the increasing number of studies in laboratory animals showing perturbations of the gut microbiome by pesticides [14]. As a first step in filling this important knowledge gap, we performed the first pesticide biomonitoring survey of the British population, and subsequently used the results of this study to perform the first pesticide association study on gut microbiome composition and function in individuals from the TwinsUK registry.

Levels of glyphosate, pyrethroid and organophosphorus residues were comparable to those of previous studies performed with other European populations [50, 51]. A large range of pesticides were applied in the UK in 2016, including insecticides (316 t), fungicides (5902 t), herbicides (7806 t), and molluscicides (161 t) [52]. Our results suggesting that the exposure to DMTP, a metabolite of methylorganophosphates (e.g. dimethoate, chlorpyrifos-methyl, azinphos-methyl, malathion, fenthion, phosmet) is related to diet is in accord with previous studies, which showed that individuals eating an organic diet had lower levels of urinary insecticides than those eating conventional non-organic products [53]. This could point to a possible source of health risks as the exposure to organophosphate during sensitive periods of life has been linked to a variety of diseases such as neurobehavioral problems after prenatal exposure [5]. The consumption of fruit and vegetables is the major source of pesticide exposure in the UK [54]. However, the consumption of agricultural products sprayed with pesticides is not always the most important source of exposure as pesticides are frequently found in dust [55] and ambient air [56]. Pesticides are also used by the amenity sector (e.g., golf courses, local authorities, lawn care operators, sport stadiums), with 80 t of pesticides applied in 2016 (77% by glyphosate, 61 t) [52]. Domestic use is also an important source of exposure with the use of herbicides to clear weeds in private gardens, or the use of insecticides indoors (e.g. anti-mosquito sprays, impregnated animal pet collars for flea control) [57].

Although we could not test whether pesticide excretion is different between individuals eating organic food and those who do not, the observed inconsistency between the results of the FFQ before recruitment and during the study is nevertheless an important result as it supports the need for intervention studies to evaluate this parameter [58, 59]. Only a few randomized controlled trials have been conducted that report lower pesticide body burden with consumption of organic food, such as the ORGANIKO trial [60, 61], and the study by Hyland and colleagues [62]. The need for an intervention study to evaluate health benefits of an organic diet is further supported by the finding that individuals who are regularly consuming organic products in our study had higher healthy eating index values. An organic diet is multifactorial and difficult to clearly define, which makes self-assessment prone to subjective bias [49]. Organic diet consumers have generally healthier lifestyles than individuals eating conventionally grown foodstuffs. This is a known confounding factor in epidemiological studies associating a decreased pesticide exposure through organic food consumption to health benefits [49]. Although organic food consumers may be healthier, this can be in part due to other demographic and lifestyle covariates as these individuals tend to have a higher physical activity, smoke less, are more educated and make heathier dietary choices. One way of at least partially mitigating these lifestyle confounding factors in determining health benefits of an organic diet would be to focus on vulnerable groups, such as pregnant farmworkers who do not necessarily have access to proper safety equipment in rural environments and are thus exposed to higher pesticide levels [63].

The findings of our study provide a foundation for larger environmental epidemiology investigations linking pesticide exposure to metabolic perturbations and their health consequences. Environmental levels of pesticide exposure have been suggested to disturb gut microbial metabolism. The insecticide metabolite Br2CA, reflecting deltamethrin exposure, was negatively associated with amino acid metabolites (Table 2). Deltamethrin transformation by *Bacillus thuringiensis* has been found to cause a downregulation of energy metabolism [64], although a direct comparison of these findings to the gut microenvironment is speculative. Microbial abundance and diversity was not found to be decreased by glyphosate as theorised by some authors [65] but increased, which is coherent with the findings of our recent study in rats where we found glyphosate interference with gut microbial metabolism [31]. The positive association between phosphate and glyphosate points to an influence on phosphate metabolism, which could be due to the metabolism of glyphosate by the gut microbiome as hypothesised previously [66]. Only one other study in humans has made a direct link between the degradation of organophosphate insecticides in the gut microbiome with negative consequences on glucose metabolism [67] although this involved higher exposed pesticide users [68].

The major limitation of this study is its sample size. The number of individuals we investigated is sufficient to provide reliable information on environmental levels of exposure, since reference values in biomonitoring studies require a sample size ranging from 73 to 120 individuals [69]. However, our sample size is low to find associations between pesticide excretion and gut microbiome composition. Small differences in alpha diversity (an effect size of 0.55) between two groups of 55 individuals can be detected with 80% statistical power [70], which provides sufficient power to suggest that the increased microbial diversity observed in individuals excreting detectable levels of glyphosate is reliable. However, gut microbiome taxonomic data is typically over-dispersed and zero-inflated [71]. There is no gold standard for statistical analysis of EWAS data, and it is not clear how a list of statistically significant associations can be translated to information usable for public health policies [72]. In our study, more than 50% of the datapoints were equal to 0 for 525 species out of the 603 detected. In this case, when a value is 0, it is not clear whether the species is absent or undetected. In addition, a large number of unidentified factors influence the results of gut microbiome studies [73]. These factors can be technical covariates such as the DNA extraction procedure [35, 74], or the sequencing approach [75], demographic differences [76], lifestyle changes such as the intake of prescription medications [77], alcohol consumption frequency and bowel movement quality [78], or even socioeconomic factors [79]. In addition, there is no gold standard for hardware and software for taxonomic assignment of shotgun metagenomics datasets [80]. Our study is thus a first step towards the understanding of pesticide-induced gut microbial changes in human populations, but larger studies will be needed.

## Conclusions

In conclusion, although consumption of fruit and vegetables has known health benefits, we show that if conventionally grown this leads to higher ingestion of pesticides with unknown long-term health consequences. We found that individuals who are regularly consuming organic products had higher healthy eating index values but that other lifestyle choices are, in all likelihood, also contributing factors. We provide the first evidence of an association between pesticide excretion and changes in gut microbiome metabolism at environmental levels of exposure in the UK population. Our findings highlight the need for future dietary interventional studies to understand the impact of pesticide exposure on gut microbiome composition and function and its health implications.

## Supplementary Information

**Additional file 1.**

**Additional file 2.**

**Additional file 3.**

**Additional file 4.**

## Data Availability

The data generated in this study are held by the Department of Twin Research at King’s College London. The data can be released to bona fide researchers. The application can be found at https://twinsuk.ac.uk/resources-for-researchers/access-our-data/. The data is anonymized and conform to GDPR standards.
