# Supplementary material for: Impacts of dietary exposure to pesticides on faecal microbiome metabolism in adult twins
Source: Environ Health. 2022 May 3;21:46. doi: 10.1186/s12940-022-00860-0 (PMC9063241; doi:10.1186/s12940-022-00860-0)
Supplement: Supplementary file 2 — Additional file 2. [file 12940_2022_860_MOESM2_ESM.docx]

**SUPPLEMENTARY MATERIAL**

**Table S1**. Calculation if the alpha and beta diversity could be influenced by pesticide exposure levels. The p-values for differences in beta diversity were estimated with a PERMANOVA test conducted with R-vegan function adonis. P-values for different alpha diversity indices (p-shannon, Shannon index ; p-simpson, Simpson index, p-observed ; observed species) were calculated with linear-mixed models considering considering age as a covariate and the family relationship as a random effect.

| **Metabolites** | **p_beta** | **p_shannon** | **p_simpson** | **p_observed** |
| --- | --- | --- | --- | --- |
| Glyphosate | 0.25 | 0.17 | 0.60 | 0.01 |
| Trans.Cl2CA | 0.36 | 0.67 | 0.90 | 0.18 |
| Cis.Cl2CA | 0.17 | 0.59 | 0.71 | 0.69 |
| 3-PBA | 0.05 | 0.36 | 0.85 | 0.09 |
| DEP | 0.09 | 0.51 | 0.75 | 0.09 |
| Br2CA | 0.13 | 0.88 | 0.57 | 0.27 |
| sumDAP | 0.06 | 0.93 | 0.91 | 0.53 |
| sumDEP | 0.09 | 0.53 | 0.76 | 0.09 |
| sumDMP | 0.04 | 0.69 | 0.96 | 0.30 |

**Table S2.** Comparative organic acid analysis in urine between a group of 28 individuals who did not present detectable glyphosate levels in their urine compared to a group of 33 individuals with detectable glyphosate levels. Concentrations of organic acids are expressed as mmol/mol Creatinine. Mean ± are provided along p-values of a t-test adjusted using the FDR method.

|  | **Undetected** | **Detected** | **Adjusted p-value** |
| --- | --- | --- | --- |
| **citric acid** | 150 ± 110 | 170 ± 120 | 0.92 |
| **aconitic acid** | 32 ± 22 | 44 ± 31 | 0.92 |
| **isocitric acid** | 6.8 ± 4.6 | 8.0 ± 5.2 | 0.92 |
| **2-ketoglutaric acid** | 8.2 ± 10 | 8.1 ± 6.4 | 0.98 |
| **succinic acid** | 12 ± 23 | 13 ± 19 | 0.98 |
| **fumaric acid** | 0.046 ± 0.25 | 0.15 ± 0.48 | 0.92 |
| **malic acid** | 1.1 ± 0.51 | 1.2 ± 1.4 | 0.92 |
| **3-hydroxy-3-methylglutaric acid** | 0.082 ± 0.30 | 0.21 ± 0.45 | 0.92 |
| **lactic acid** | 71 ± 170 | 42 ± 53 | 0.92 |
| **pyruvate acid** | 6.2 ± 4.1 | 6.6 ± 6.7 | 0.98 |
| **3-hydroxybutyric acid** | 1.2 ± 0.78 | 1.4 ± 1.4 | 0.92 |
| **pyroglutamic acid** | 16 ± 9.1 | 18 ± 11 | 0.92 |
| **3-hydroxyisovaleric acid** | 7.4 ± 5.0 | 10 ± 7.2 | 0.92 |
| **methylmalonic acid** | 1.1 ± 0.48 | 1.1 ± 0.54 | 0.98 |
| **homovannilic acid** | 1.9 ± 1.1 | 2.2 ± 1.2 | 0.92 |
| **5-hydroxyindoloacetic acid** | 2.2 ± 1.3 | 3.5 ± 4.5 | 0.92 |
| **vanillilmandelic acid** | 1.5 ± 0.66 | 1.8 ± 0.96 | 0.92 |
| **4-hydroxyphenylacetic acid** | 10 ± 11 | 12 ± 5.5 | 0.92 |
| **orotic acid** | 0.050 ± 0.26 | 0.030 ± 0.17 | 0.97 |
| **glutaric acid** | 0.97 ± 0.40 | 0.92 ± 0.30 | 0.92 |
| **2-hydroxyglutaric acid** | 4.2 ± 2.5 | 3.7 ± 2.2 | 0.92 |
| **glycolic acid** | 44 ± 28 | 42 ± 26 | 0.97 |
| **oxalic acid** | 7.7 ± 5.4 | 9.2 ± 10 | 0.92 |
| **glyceric acid** | 1.9 ± 1.1 | 2.1 ± 1.7 | 0.92 |
| **2-hydroxyisobutyric acid** | 13 ± 6.7 | 13 ± 7.3 | 0.98 |
| **X2.hydroxybutyric acid** | 0.36 ± 0.61 | 0.54 ± 0.80 | 0.92 |
| **ethylmalonic acid** | 1.5 ± 0.72 | 1.5 ± 0.71 | 0.98 |
| **methylsuccinic acid** | 0.046 ± 0.25 | 0.15 ± 0.84 | 0.92 |
| **adipic acid** | 1.5 ± 0.89 | 1.6 ± 0.91 | 0.92 |
| **suberic acid** | 0.94 ± 0.64 | 0.94 ± 0.68 | 0.98 |
| **methylcitric acid** | 0.71 ± 0.53 | 0.71 ± 0.48 | 0.98 |
| **4-hydroxyphenypyruvic acid** | 1.1 ± 0.41 | 1.0 ± 0.41 | 0.92 |

**Table S3**. Responses to and corresponding weights applied to the food frequency questionnaire question “Please indicate to what extent you consume, when available, organic fruits and vegetables?” to estimate a proxy for the potential of pesticide exposure.

| Response | Weight applied to fruit and vegetables (g/week) |
| --- | --- |
| 0 - I don’t eat fruits and vegerables | Removed from analysis |
| 1 Almost all non-organic fruits and vegetables | 2 |
| 2 A mixture of organic and non-organic fruits and vegetables | 1 |
| 3 Almost all organic fruits and vegetables | 0.2 |


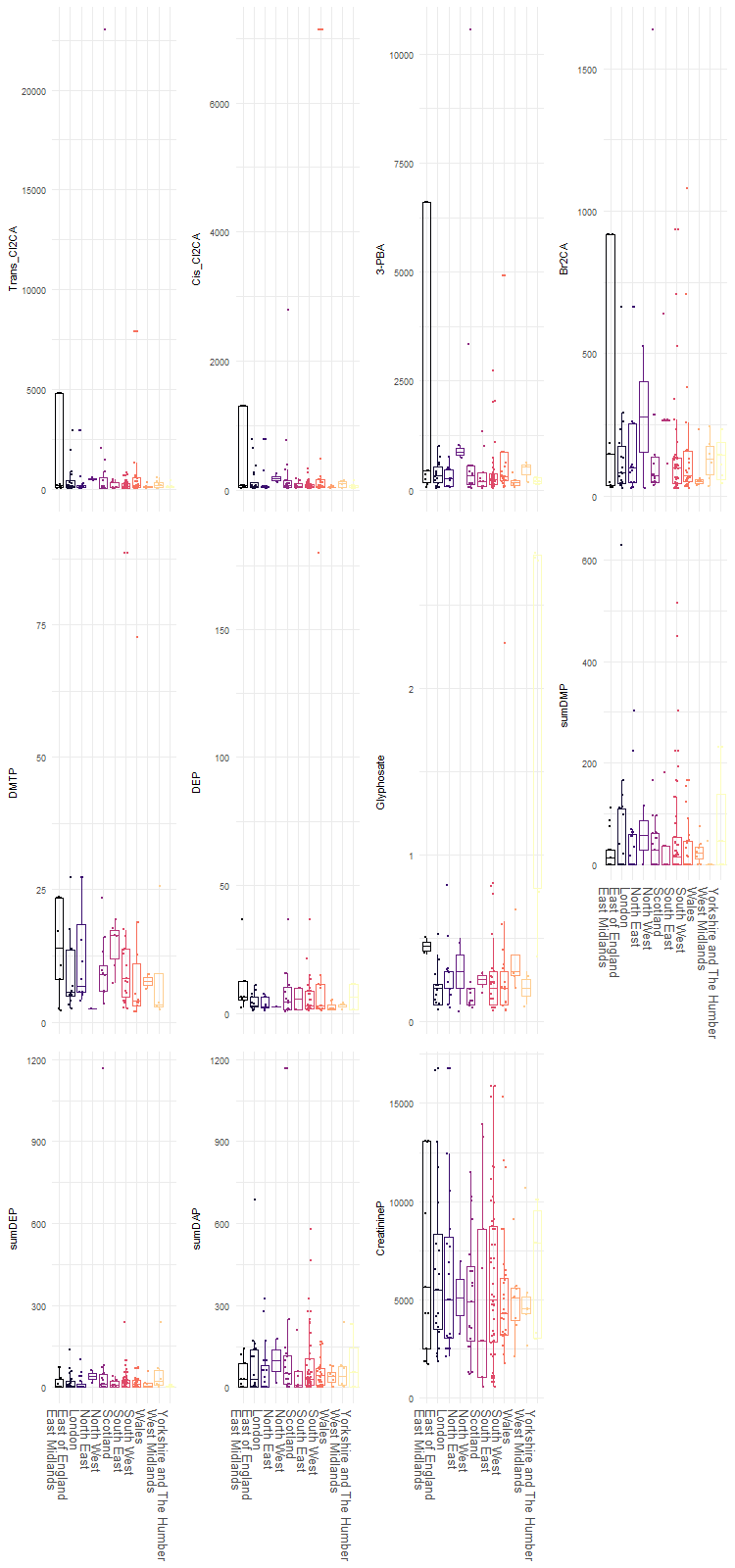


**Figure S1**. Pesticide urinary excretion across 9 UK regions.


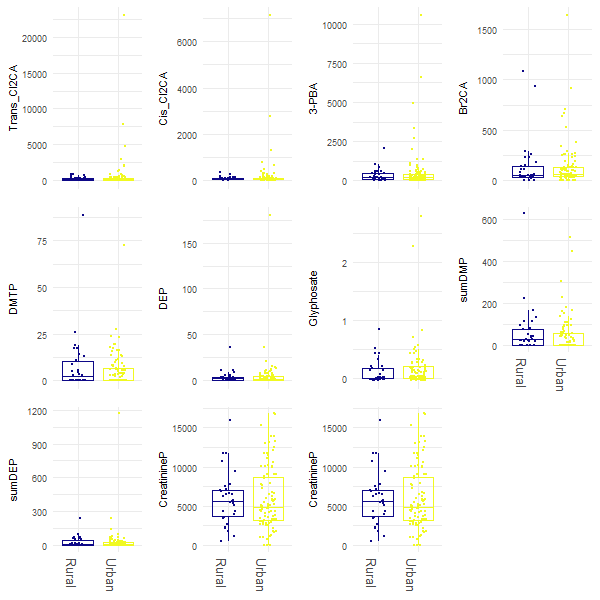


**Figure S2**. Rural/urban classification had no influence on pesticide urinary excretion.


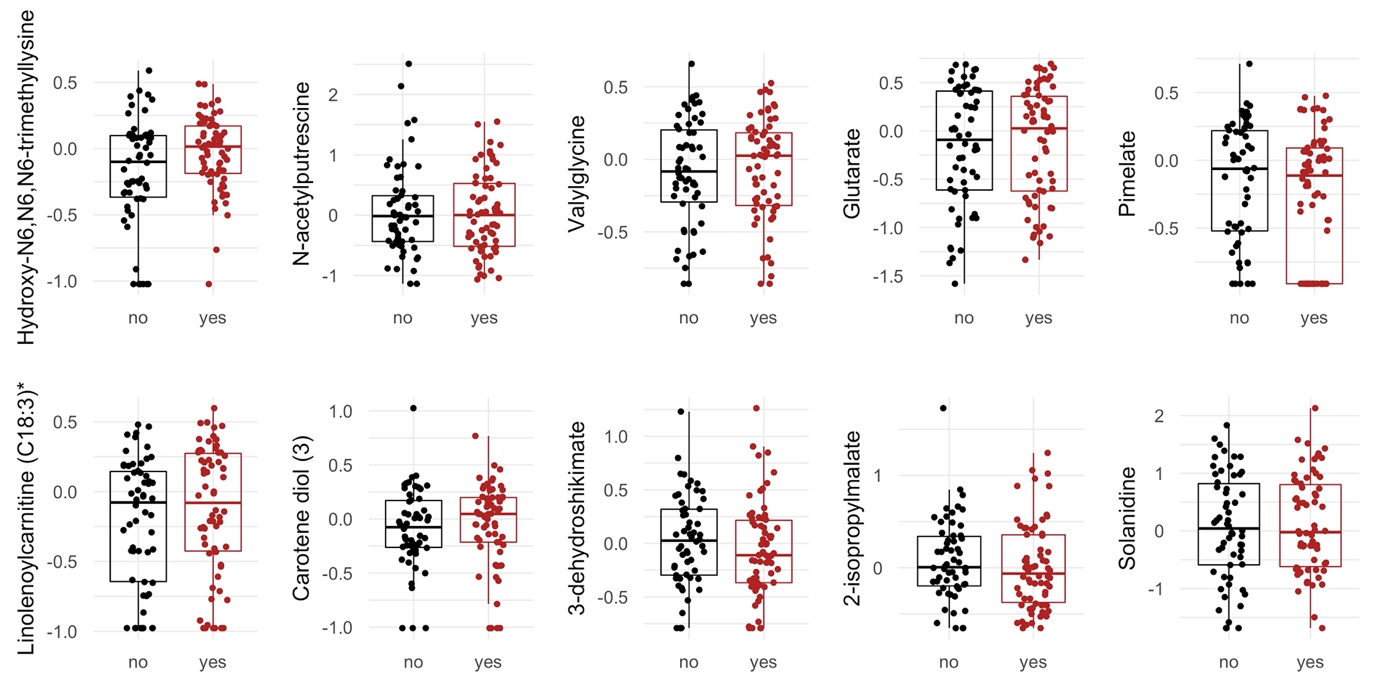


**Figure S3**. The 10 metabolites, which are known to characterise the effects of glyphosate on the faecal metabolome in rats. Log-transformed abundance values are shown as box plots.

**Detailed pesticide exposure estimation**

**General pesticide screening in urine samples**

Urine was extracted with acetonitrile and QuEChERS salt and then injected into the LC/MS-MS system. For this, Albendazol, Carbendazim ^3^D, Phosalone ^10^D were used as internal standards (IS). Albendazol (LGC, UK) at a purity of 98.1 % was dissolved in acetonitrile and 10 % formic acid to obtain a working solution of 2 mg/L. Carbendazim ^3^D (LGC, UK) at a purity of 96.6 %, was dissolved in dimethylformamide to obtain a working solution of 2 mg/L. Phosalone ^10^D purchased as a solution at 100 mg/L (LGC, UK), was diluted in acetonitrile to obtain a working solution of 1 mg/L.

For sample preparation and extraction, 10 µL of IS and 5 mL of acetonitrile was added to 1 mL of urine. The sample was agitated before centrifugation at 3000 g for 5 min and the supernatant was transferred to a 15 mL glass tube. One gram of QuEChERS Mix 1 (MgSO_4_/NaCl/C_6_H_5_Na_3_O_7_, 2H_2_O/C_6_H_6_Na_2_O_7_, 1.5H_2_O) (4/1/1/0.5) (w/w/w/w) was added to the sample and agitated before being centrifuged again at 3000 g for 5 min. The organic phase was then transferred to a 15 mL glass tube and evaporated to dryness under nitrogen flow. The dried sample was taken in 200 µL of (50/50) mobile phase solutions. A 2 µL aliquot of the re-dissolved sample solution was injected into the LC-MS/MS system.

The LC-MS/MS system included a Shimadzu NEXERA X2 series and 8060 triple quadrupole mass spectrometer. Chromatographic separations were performed on a Raptor Biphenyl column (100 x 2.10 mm, 2.7 µm particles) (Restek, France). Mobile phase A contained 0.002 % formic acid in 2 mM ammonium formiate and phase B consisted of methanol and 0.002 % formic acid in 2 mM ammonium formiate. Identification and quantification of pesticides was performed in positive and negative mode using multiple reaction monitoring (MRM) of a quantification and additional qualifier ion. To meet the criteria for a positive identification, the ratio between the quantitative and the qualifying transition ions had to fall within ±20% of that established by the calibration standards.

A total of 186 compounds were measured in the urine samples from 130 twins from the TwinsUK cohort. Organophosphates, pyrethroids, and glyphosate do not appear in this table because they were measured using targeted quantitative assays.

|  |  | **1st transition** | | **2nd transition** | |  |
| --- | --- | --- | --- | --- | --- | --- |
| **Name** | **LD (µg/L)** | **m/z(1)** | **m/z(2)** | **m/z(1)** | **m/z(2)** | **Ret. Time** |
| 2.4 DMA | 0.1 | 121.9 | 107.2 | 121.9 | 77.25 | 5.893 |
| Abamectin | 0.1 | 890.6 | 305.4 | 890.6 | 567.4 | 13.631 |
| Acephate | 0.1 | 184 | 143 | 184 | 49 | 2.419 |
| Acequinocyl |  | 402 | 343.3 | 384.9 | 343.25 | 15.345 |
| Acetamiprid | 0.1 | 222.9 | 126.1 | 222.9 | 90.1 | 6.133 |
| Acibenzolar acid | 0.1 | 179.2 | 57.05 | 179.2 | 107.1 | 3.337 |
| Acibenzolar-S-methyl | 0.1 | 210.9 | 136.2 | 210.9 | 140.1 | 9.481 |
| Ametryn | 0.1 | 228 | 186 | 228 | 68 | 7.853 |
| Azadirachtin | 0.1 | 743 | 725 | 743 | 625 | 8.097 |
| Azinphos-methyl | 0.1 | 318.2 | 132.2 | 318.2 | 76.5 | 9.837 |
| Azoxystrobin | 0.1 | 404 | 329 | 404 | 344 | 10.546 |
| Bifenazate | 0.1 | 301 | 170.3 | 301 | 152.2 | 9.618 |
| Bitertanol | 0.1 | 338.4 | 269.3 | 338.4 | 99.3 | 10.282 |
| Boscalid | 0.1 | 342.9 | 307.2 | 342.9 | 271.2 | 9.074 |
| Bromacil | 0.1 | 261 | 205 | 263 | 207 | 6.242 |
| Bupirimate | 0.1 | 317 | 166 | 317 | 108 | 9.949 |
| Buprofezin | 0.1 | 306 | 201 | 305.9 | 106.2 | 11.782 |
| Carbaryl | 0.1 | 202 | 145 | 202 | 127 | 6.894 |
| Carbendazim | 0.2 | 192 | 160 | 192 | 132 | 5.258 |
| Carbetamide | 0.1 | 237.1 | 192 | 237.1 | 118 | 5.916 |
| Carbofuran | 0.1 | 222 | 123 | 222 | 165 | 6.841 |
| Carbofuran-3-hydroxy | 0.1 | 255 | 163 | 255 | 220 | 5.243 |
| Chlorantraniliprole | 0.1 | 483.1 | 452 | 483.1 | 285.1 | 8.618 |
| Chlorfluazuron | 0.1 | 540.1 | 383.1 | 541.1 | 384.1 | 12.859 |
| Clofentezine | 0.1 | 303 | 138 | 303 | 75 | 11.536 |
| Clothianidin | 0.1 | 250 | 132 | 250 | 169 | 4.756 |
| Cyantraniliprole | 0.1 | 474.8 | 286 | 474.8 | 444 | 7.861 |
| Cycloxydim A | 0.5 | 324.2 | 236.1 | 324.2 | 134.2 | 8.097 |
| Cycloxydim B | 0.1 | 324.2 | 236.1 | 324.2 | 134.1 | 11.761 |
| Cyflufenamid | 0.1 | 413 | 295 | 413 | 241 | 10.931 |
| Cymoxanil | 5 | 199.3 | 111.2 | 199.3 | 83.2 | 5.588 |
| Cyproconazole | 0.1 | 292 | 70 | 292 | 125 | 8.997 |
| Cyprodinil | 0.1 | 226 | 93 | 226 | 77 | 9.908 |
| DEET | 0.5 | 192 | 119 | 192 | 91 | 7.756 |
| Desmedipham | 0.1 | 318 | 182 | 318 | 136 | 7.689 |
| Diazinon | 0.1 | 305 | 169 | 305 | 153 | 10.151 |
| Dichlorvos | 1 | 222.9 | 108.9 | 220.9 | 108.9 | 6.184 |
| Difenoconazole | 0.1 | 405.8 | 251 | 405.8 | 337.1 | 11.857 |
| Diflubenzuron | 0.1 | 311.2 | 158.2 | 311.2 | 141.2 | 9.323 |
| Dimethoate | 0.1 | 229.8 | 171.2 | 230 | 125 | 5.321 |
| Dimethomorph | 0.1 | 388 | 301 | 388 | 165 | 10.5 |
| Dinocap | 0.2 | 295.1 | 209.1 | 295.1 | 134.1 | 10.59 |
| Dinotefuran | 0.1 | 203 | 114 | 203 | 87 | 3.332 |
| Dithianon | 1 | 296 | 264 | 296 | 238.1 | 12.855 |
| Diuron | 0.1 | 233 | 72 | 233 | 46 | 6.821 |
| DMF | 0.2 | 150 | 107.1 | 150 | 77.1 | 5.989 |
| DMPF | 0.1 | 163 | 122.1 | 163 | 107.1 | 4.467 |
| DMST | 0.2 | 215 | 106 | 215 | 79 | 6.621 |
| Dodine | 0.2 | 228.2 | 57.3 | 228.1 | 43.2 | 8.246 |
| Emamectin B1a | 0.1 | 886.3 | 158.2 | 886.2 | 82.3 | 12.427 |
| Emamectin B1b | 5 | 872.7 | 158.3 | 872.7 | 82.3 | 12.214 |
| Epoxiconazole | 0.1 | 330 | 121 | 330 | 101 | 9.921 |
| Etoxazole | 0.1 | 360.2 | 141.2 | 360.3 | 113.2 | 12.694 |
| Fenamiphos | 0.1 | 304 | 201.1 | 304.1 | 216.5 | 9.404 |
| Fenamiphos sulfone | 0.1 | 336 | 266 | 336 | 188 | 6.946 |
| Fenamiphos sulfoxide | 0.1 | 320 | 233 | 320 | 108 | 6.825 |
| Fenarimol | 0.1 | 331.1 | 268.2 | 331.1 | 189.3 | 9.252 |
| Fenazaquin | 0.1 | 307 | 57 | 307 | 161 | 13.199 |
| Fenbuconazole | 0.1 | 337.2 | 70.2 | 337.2 | 91.3 | 10.115 |
| Fenbutatin oxide | 0.5 | 518.9 | 91.2 | 518.9 | 196.5 | 14.316 |
| Fenhexamid | 0.2 | 302.2 | 97.3 | 301.8 | 55.2 | 8.726 |
| Fenoxycarb | 0.1 | 302 | 116 | 302 | 88 | 10.043 |
| Fenpropidin | 0.1 | 274 | 147 | 274 | 117 | 8.046 |
| Fenpropimorph | 0.1 | 304 | 147 | 304 | 117 | 9.83 |
| Fenpyrazamine | 0.2 | 331.9 | 230.3 | 331.9 | 216.2 | 9.558 |
| Fenpyroximate | 0.2 | 422 | 366 | 422 | 138 | 13.546 |
| Fenthion | 0.5 | 279 | 169 | 279 | 247 | 10.741 |
| Fenthion sulfone | 0.2 | 311 | 125 | 311 | 109 | 8.027 |
| Fenthion sulfoxide | 0.1 | 294.9 | 280.1 | 295 | 109.2 | 7.852 |
| Fipronil | 0.1 | 435 | 329.9 | 435 | 250 | 8.131 |
| Fipronil sulfone | 0.5 | 450.9 | 414.9 | 450.9 | 282 | 8.685 |
| Flazasulfuron | 0.1 | 408 | 182 | 408 | 139 | 8.463 |
| Flonicamid | 0.1 | 228.1 | 81.1 | 228 | 146.1 | 3.878 |
| Fluazinam | 0.1 | 462.9 | 397.5 | 463 | 415.5 | 11.214 |
| Flubendiamide | 0.2 | 683 | 408 | 683 | 274 | 9.57 |
| Fludioxonil | 0.1 | 247.1 | 180.1 | 247.1 | 126.1 | 7.577 |
| Flufenoxuron | 5 | 489 | 158 | 489 | 140 | 12.21 |
| Fluopyram | 0.2 | 397 | 173 | 397 | 145 | 8.582 |
| Fluquinconazole | 0.1 | 376 | 349 | 376 | 307 | 10.129 |
| Flurtamone | 0.2 | 334 | 247 | 334 | 178 | 8.252 |
| Flusilazole | 0.1 | 316 | 247 | 316 | 165 | 9.845 |
| Flutriafol | 0.2 | 302 | 70 | 302 | 123 | 7.408 |
| Foramsulfuron | 0.5 | 453 | 182 | 453 | 139 | 7.886 |
| Formetanate HCl | 0.1 | 222 | 165.1 | 222 | 93.1 | 3.344 |
| Hexaconazole | 0.5 | 314.3 | 159.2 | 314.3 | 70.2 | 9.289 |
| Hexythiazox | 0.1 | 353 | 228 | 353 | 168 | 12.932 |
| Hydramethylnon | 0.1 | 495 | 323 | 495 | 151 | 10.117 |
| Imazalil | 0.1 | 297.1 | 159.2 | 296.9 | 41.2 | 9.479 |
| Imidacloprid | 0.1 | 256 | 209 | 256 | 174 | 5.747 |
| Indaziflame | 5 | 302 | 158.1 | 302 | 105.1 | 9.141 |
| Indoxacarb | 0.1 | 528 | 203.2 | 528 | 150.2 | 12.034 |
| Isoproturon | 0.1 | 207 | 72 | 207 | 46 | 7.063 |
| Isoxaben | 0.2 | 333 | 165 | 333 | 107 | 9.638 |
| Kresoxim methyl | 0.1 | 314 | 267 | 314 | 116 | 10.862 |
| Lufenuron | 5 | 511.2 | 158.2 | 511.2 | 141.2 | 11.26 |
| Malaoxon | 0.1 | 315 | 99 | 315 | 127 | 7.086 |
| Malathion | 0.2 | 347.8 | 285.1 | 348 | 127 | 9.365 |
| Mepanipyrim | 0.1 | 224 | 77 | 224 | 106 | 9.375 |
| Mephosfolan | 0.1 | 270 | 139.9 | 270 | 196 | 7.287 |
| Mesosulfuron methyl | 0.1 | 503.9 | 182.2 | 504.2 | 139.2 | 8.937 |
| Metalaxyl | 0.1 | 280 | 220 | 280 | 192 | 8.246 |
| Metaldehyde | 1 | 193.9 | 62.2 | 193.9 | 106.6 | 5.2 |
| Metamitron | 0.1 | 203 | 175.1 | 203 | 104.1 | 5.471 |
| Metconazole | 0.1 | 320 | 70 | 320 | 125 | 9.758 |
| Methamidophos | 0.1 | 141.9 | 125 | 141.9 | 93.5 | 1.854 |
| Methidathion | 0.1 | 320 | 145 | 320 | 85 | 9.01 |
| Methiocarb | 0.2 | 226 | 169 | 226 | 121 | 8.182 |
| Methiocarb sulfone | 0.1 | 275 | 258 | 275 | 122 | 5.963 |
| Methiocarb sulfoxide | 0.1 | 242 | 185 | 242 | 122 | 5.673 |
| Methomyl | 0.1 | 163 | 88 | 163 | 106 | 4.507 |
| Methoxyfenozide | 0.1 | 369 | 149 | 369 | 91 | 9.109 |
| Metsulfuron methyl | 0.1 | 381.9 | 167.2 | 381.9 | 199 | 6.8 |
| Myclobutanil | 0.2 | 289 | 70 | 289 | 125 | 8.721 |
| Norflurazon | 0.2 | 304.1 | 140.1 | 304 | 283.5 | 7.907 |
| Novaluron | 0.1 | 493 | 158 | 493 | 141 | 10.433 |
| Nuarimol | 1 | 314.9 | 207.2 | 314.9 | 251.2 | 9.327 |
| Omethoate | 0.1 | 214 | 125 | 214 | 183 | 3.151 |
| Oryzalin | 0.5 | 345.2 | 281.2 | 345.2 | 116.2 | 9.348 |
| Oxamyl | 0.1 | 237 | 72 | 237 | 90 | 4.441 |
| Oxydemeton methyl | 0.1 | 246.9 | 169.2 | 246.9 | 125 | 4.569 |
| Oxydemeton methyl sulfone | 0.1 | 262.8 | 169.15 | 262.8 | 109.15 | 4.787 |
| Penconazole | 0.1 | 284 | 70 | 284 | 159 | 9.54 |
| Phenmedipham | 0.2 | 318 | 168 | 318 | 136 | 7.796 |
| Phorate sulfone | 0.1 | 292.8 | 171 | 292.8 | 115 | 7.934 |
| Phorate sulfoxide | 0.1 | 277 | 143 | 277 | 199 | 7.677 |
| Phosalone | 0.2 | 368 | 182 | 368 | 111 | 11.882 |
| Phosmet | 0.1 | 318.2 | 160.25 | 318.2 | 133.25 | 9.855 |
| Phosmet oxon |  | 302 | 160 | 302 | 133.1 | 7.527 |
| Phoxim | 0.1 | 299 | 77 | 299 | 129 | 11.125 |
| Piperonyl butoxide | 0.1 | 356 | 177 | 356 | 119 | 12.061 |
| Pirimicarb | 0.1 | 239 | 182 | 239 | 72 | 7.726 |
| Pirimicarb desmethyl | 0.1 | 225 | 72 | 225 | 168 | 6.329 |
| Prochloraz | 0.1 | 375.8 | 308 | 375.8 | 266.1 | 11.428 |
| Profenofos | 0.1 | 373 | 303 | 375 | 347 | 11.416 |
| Prohexadione Ca | 1 | 212.9 | 157.25 | 213.4 | 157.25 | 4.892 |
| Propamocarb | 0.2 | 189 | 102 | 189 | 74 | 3.054 |
| Propargite | 0.1 | 368 | 231 | 368 | 175 | 12.777 |
| Prosulfuron | 0.1 | 420.3 | 141.3 | 419.8 | 167.2 | 7.704 |
| Prothioconazole | 0.1 | 342.1 | 100 | 344.1 | 100 | 9.736 |
| Prothioconazole desthio | 0.2 | 311.9 | 70.2 | 311.9 | 125.2 | 9.276 |
| Pymetrozine | 0.1 | 218 | 105.2 | 218.3 | 78.2 | 4.925 |
| Pyraclostrobin | 0.1 | 388 | 163 | 390 | 163 | 11.969 |
| Pyridaben | 0.2 | 365 | 309 | 365 | 147 | 13.795 |
| Pyridafol | 0.2 | 206.9 | 77.1 | 208.9 | 104.1 | 5.611 |
| Pyridate | 0.2 | 378.8 | 207.1 | 378.8 | 104 | 13.946 |
| Pyrifenox | 0.1 | 295 | 93 | 297 | 93 | 10.3 |
| Pyrimethanil | 0.1 | 200 | 107 | 200 | 82 | 8.229 |
| Pyriproxyfen | 0.2 | 322 | 96 | 322 | 78 | 12.618 |
| Quinalphos | 0.2 | 299 | 147.2 | 299 | 163.2 | 10.346 |
| Quinoxyfen | 0.2 | 308 | 197 | 308 | 162 | 12.296 |
| Rimsulfuron |  | 432.4 | 182.3 | 432.4 | 325.4 | 7.972 |
| Rotenone | 0.1 | 395 | 213 | 395 | 192 | 12.906 |
| Spinetoram J | 0.1 | 748.2 | 142.3 | 748.2 | 98.25 | 13.256 |
| Spinetoram L | 0.1 | 760.2 | 142.35 | 760.2 | 98.15 | 13.548 |
| Spinosad A | 0.1 | 732.3 | 142.3 | 732.1 | 98.3 | 12.427 |
| Spinosad D | 0.1 | 746.2 | 142.3 | 746.2 | 98.3 | 13.037 |
| Spiromesifen | 0.1 | 371 | 273 | 371 | 255 | 12.663 |
| Spirotetramat | 0.1 | 374 | 216 | 374 | 302 | 10.009 |
| Spirotetramat cis keto hydroxy | 0.1 | 318 | 268.3 | 318 | 214.1 | 7.625 |
| Spirotetramat enol | 0.1 | 302.2 | 216.3 | 302.2 | 117.2 | 6.873 |
| Spirotetramat enol glucoside | 0.1 | 464 | 216.2 | 464 | 270.2 | 4.726 |
| Spirotetramat mono hydroxy | 0.1 | 304 | 254.3 | 304 | 119.1 | 6.747 |
| Spiroxamine | 0.1 | 298 | 144 | 298 | 100 | 7.828 |
| Sulfosulfuron | 0.1 | 471.2 | 211.2 | 470.9 | 261.2 | 8.028 |
| Tebuconazole | 0.1 | 308 | 70 | 308 | 125 | 9.315 |
| Tebufenozide | 0.1 | 353 | 133 | 353 | 105 | 10.569 |
| Tebufenpyrad | 0.2 | 334 | 117 | 334 | 145 | 11.264 |
| Teflubenzuron | 5 | 381.1 | 158.2 | 381.2 | 141.2 | 11.363 |
| Tetraconazole | 0.1 | 372 | 159 | 372 | 70 | 8.808 |
| TFNA | 0.5 | 190.2 | 69.05 | 190.2 | 119.15 | 1.372 |
| TFNG | 0.2 | 248.9 | 148.1 | 248.9 | 203.2 | 2.311 |
| Thiabendazole | 0.2 | 202 | 175 | 202 | 131 | 6.001 |
| Thiacloprid | 0.1 | 253 | 126 | 253 | 90 | 6.719 |
| Thiamethoxam | 0.1 | 292 | 211 | 292 | 181 | 5.002 |
| Thiodicarb | 0.1 | 355 | 88 | 355 | 108 | 9.036 |
| Thiophanate methyl | 0.1 | 343 | 151 | 343 | 311 | 7.137 |
| Triadimefon | 0.2 | 293.9 | 69.3 | 294.2 | 197.2 | 8.68 |
| Triadimenol | 0.2 | 296.9 | 133.2 | 296.9 | 105.2 | 9.597 |
| Trichlorfon | 0.1 | 257 | 109 | 257 | 220 | 4.7 |
| Tridemorph | 0.5 | 298 | 130 | 298 | 98 | 10.257 |
| Trifloxystrobin | 0.1 | 408.9 | 186.1 | 408.9 | 206.2 | 11.722 |
| Triflumizole | 0.1 | 346 | 278 | 346 | 73 | 10.635 |
| Triflumuron | 0.1 | 359 | 156 | 359 | 139 | 9.814 |
| Triforine | 0.1 | 434.7 | 390 | 434.7 | 98.2 | 7.34 |
| Vamidothion | 0.1 | 288 | 146 | 288 | 118 | 5.518 |

**Glyphosate biomonitoring in urine samples**

Glyphosate and its major metabolite, aminomethylphosphonic acid (AMPA), were successfully measured in 124 urine samples (note: an insufficient volume of urine was available for the remaining 6 samples). Glyphosate and AMPA were measured following derivatization with FMOC-Cl (9-fluorenylmethyl chloroformate). Samples were then extracted with diethyl ether and injected into the LC/MS-MS system. Glyphosate ^13^C2^15^N was used as an IS, and was purchased as a solution at 100 mg/L (LGC, UK) and diluted in deionized water to obtain a working solution of 0.5 mg/L. Glyphosate and AMPA (LGC, UK) were of 98.69% and 99% purity respectively, and were dissolved in deionized water to obtain working solutions at increasing concentrations ranging from 0.01 to 50 mg/L. These standard solutions were used to spike glyphosate-free urine for the preparation of the calibration curves for standards. Six calibration standards between the higher limit of quantification (LOQ) and the lower LOQ (namely, between 0.1 to 10 µg/L) were necessary for the calibration.

FMOC (Acros Organics, Belgium) was prepared at 50 g/L and was used for the derivatization reaction. Glyphosate and AMAP already derivatized with FMOC were purchased from LGC (98 %, 99.6 % purity respectively). Working solutions of glyphosate-FMOC and AMAP-FOMC at 0.1 and 1 mg/L respectively were used to spike glyphosate-free urine samples to prepare internal quality controls at 0.5 and 5 µg/L.

A 50 µL volume of IS and 1 mL of 0.5 M tetraborate buffer (pH 9) were added to 1 mL of urine. Then, 3 mL of the FMOC solution was added and the sample allowed to stand for 30 min in the dark. For the extraction of the formed derivatives, 1 mL of 6M HCl and 6 mL of diethyl ether were added to each sample and agitated for 15 min before centrifugation at 3000 g for 5 min. The organic phase was then transferred to a 15 mL glass tube and evaporated to dryness under nitrogen flow. The dried sample was taken up in 200 µL of (50/50) mobile phase solutions and a 10 µL aliquot injected into the LC-MS/MS system. The calibration standards were treated in the same way after spiking of the appropriate volume of the working solutions.

The LC-MS/MS system included a Shimadzu NEXERA X2 series and 8060 triple quadrupole mass spectrometer. Chromatographic separations were performed at 40 °C on a Kinetex C18 100A column (100 x 2.10 mm, 2.6 µm particles) (Phenomenex, France). Mobile phase A contained 0.05% formic acid and phase B included acetonitrile and 0.05% formic acid. Identification and quantification of glyphosate-FMOC and AMPA-FMOC were performed in negative mode using MRM of a quantifier ion (390.2/62.9 and 331.9/110.1, respectively) and an additional qualifier ion (389.9/168.1 and 331.9/62.9, respectively). To meet the criteria for a positive identification, the ratio between the quantitative and the qualifying transition ions (derived from the precursor ion) had to fall within ±20% of that established by the calibration standards.

**Biomonitoring of pyrethroid metabolites in urine samples**

Pyrethroid metabolites are measured in urine after hydrolysis with β-glucuronidase (Helix Pomatia) followed by extraction with hexane and then injected into the LC/MS-MS system.

For this, 3-PBA ^13^C and trans-Cl_2_CA ^6^D were used as IS. Both IS, purchased as solutions of 100 mg/L (LGC, UK and CLUZEAU INFO LABO, France), were diluted in (50/50) acetonitrile and 2mM ammonium formiate buffer (pH 3) at 0.05 mg/L to obtain IS working solutions. For calibration curves, working solutions containing 3-PBA in methanol at 1 g/L, 4-FPBA at 100 mg/L in acetonitrile, 2,2-dichlorovinyl-2,2-dimethylcyclopropane-1-carboxylic acid (Cl_2_CA) (cis and trans) at 10 mg/L in methanol (CLUZEAU INFO LABO, France) and cis−3‐(2,2‐dibromovinyl)‐2,2‐dimethylcyclopropane‐1‐carboxylic acid (Br_2_CA) at 10 mg/L in methanol (CLUZEAU INFO LABO, France), were prepared at increasing concentrations ranging from 0.01 to 2 mg/L. These solutions were used to spike pyrethroid-free urine for the preparation of the calibration curve standards. Six calibration standards between the higher LOQ and the lower LOQ (namely, between 0.025 to 10 µg/L) were necessary for the calibration.

To 5 mL of urine, 25 µL of IS and 1.25 mL of 1 M sodium acetate buffer (pH 4.8) were added. Then, 20 µL of β-glucuronidase (Helix Pomatia) was added and samples incubated overnight for 16 h at 37 °C. For the extraction of the hydrolyzed molecules, 1 mL of 37 % HCl and 6 mL of hexane were added to the samples and agitated for 10 min before centrifugation at 2000 g for 5 min. The organic phase was then transferred to a 15 mL glass tube. Hexane (6 mL) was added to the remaining aqueous phase and agitated for 10 min before centrifugation at 2000 g for 5 min. The two organic phases were then combined into one tube. Then, 3 mL of 0.1 M NaOH was added to this final organic phase and agitated for 10 min before centrifugation at 2000 g for 5 min. After removal of the upper phase, 200 µL of 37 % HCl and 6 mL of hexane were added to the samples and agitated for 10 min before centrifugation at 2000 g for 5 min. The upper phase was then transferred to a 10 mL glass tube and evaporated to dryness under nitrogen flow. The dried sample was taken up in 80 µL of water and 0.1 % formic acid (70/30) and 10 µL injected into the LC-MS/MS system. The calibration standards were treated in the same way after spiking of the appropriate volume of the working solutions.

The LC-MS/MS system included a Shimadzu LC-20AD and AB SCIEX API 5500 QTrap triple quadrupole mass spectrometer. Chromatographic separations were performed on an Atlantis T3 column (150 x 2.10 mm, 5 µm particles) (Waters, USA). Mobile phase A contained 0.1% formic acid and phase B included (95/5) methanol acidified with 0.1% formic acid and phase A. Identification and quantification of 3-PBA, 4-FPBA, Cl_2_CA (cis and trans) and Br_2_CA were performed in negative mode using MRM of a quantifier ion (213.0/92.9, 231.0/93.1, 208.9/36.9 and 342.9/80.8, respectively) and an additional qualifier ion (213.0/65.1, 231.0/65.1, 207.0/35.0 and 296.8/80.9, respectively). To meet the criteria for a positive identification, the ratio between the quantitative and the qualifying transition ions (derived from the precursor ion) had to fall within ±20% of that established by the calibration standards.

**Organophosphate metabolite biomonitoring**

Organophosphate metabolites (dialkyl phosphate, DAP) were measured in urine after an extraction with ethyl acetate and diethyl ether. They were then injected into the LC/MS-MS system.

DMP ^6^D, DMTP ^6^D, DMDTP ^6^D, DEP ^10^D, DETP ^10^D and DEDTP ^13^C_4_ were used as IS. DMTP ^6^D, DMDTP ^6^D and DEDTP ^13^C_4_ (LGC, UK) at 97%, 98% and 95% purity respectively, were dissolved in methanol to obtain a working solutions of 1 g/L. DMP ^6^D and DEP ^10^D (CLUZEAU INFO LABO, France) at purities of 95 % and 99 % respectively, were dissolved in methanol to obtain working solutions of 1 g/L. DETP ^10^D was purchased as a solution at 100 mg/L (CLUZEAU INFO LABO, France). IS solutions were mixed to obtain a working solution of 1 mg/L per IS. DMP, DETP and DEDTP (SIGMA-ALDRICH, USA) at purities of 100 %, 98 % and 90 % respectively, were dissolved in methanol. DMTP and DMDTP (LGC, UK) of 96 % and 95 % purity respectively, were dissolved in methanol. DEP (CHEM Service, USA) at a purity of 99.5 % was dissolved in methanol. DMP, DMTP, DMDTP, DEP, DETP and DEDTP were mixed to obtain working solutions at increasing concentration ranging from 0.1 to 10 mg/L. These standards solutions were used to spike DAP-free urine for the preparation of the calibration curve standards. A total of 6 calibration standards between the higher LOQ and the lower LOQ (namely, between 2 to 100 µg/L) were necessary for the calibration.

To 2 mL of urine, a 20 µL aliquot of IS was added. Then, 4 g of sodium chloride, 5 mL of diethyl ether and 1 mL 6 M HCL were added, and samples agitated for 15 min before centrifugation at 2000 g for 5 min. The organic phase was transferred to a 15 mL glass tube. The aqueous phase was re-extracted with 5 mL ethyl acetate and agitation for 15 min before centrifugation at 2000 g for 5 min. The organic phase from this second extraction was combined with the first organic phase before evaporation to dryness under nitrogen flow. The dried sample was taken up into 1 mL of (50/50) methanol and 2 mM ammonium formiate (pH 3) and 5 µL injected into the LC-MS/MS system. The calibration standards were treated in the same way after spiking of the appropriate volume of the working solutions.

The LC-MS/MS system included a Shimadzu NEXERA X2 series and 8060 triple quadrupole mass spectrometer. Chromatographic separations were performed on an INERTSIL ODS3 column (100 x 2.10 mm, 5 µm particles) (GL Sciences INC., JAPAN). Mobile phase A contained 2 mM ammonium formiate (pH 3) and phase B included (90/10) acetonitrile and 2 mM ammonium formiate (pH 3). Identification and quantification of DMP, DMTP, DMDTP, DEP, DETP and DEDTP were performed in negative mode using MRM of a quantifier ion (125.4/63.1, 141.3/126.1, 157.3/112.1, 153.4/79.1, 169.4/95.1 and 185.3/157.2, respectively) and an additional qualifier ion (125.4/79.1, 141.3/96.1, 157.3/142.1, 153.4/125.1, 169.4/141.1 and 185.3/111.1, respectively).

**Dithiocarbamate biomonitoring in urine samples**

Dithiocarbamates are measured by carbon disulfide (CS_2_) in urine after acid hydrolysis at high temperature. The CS_2_ produced was injected into a headspace GC/MS system. For this, Benzene ^6^D (LGC, UK) used as an IS at 2 g/L was diluted in methanol to obtain a working solution of 1 mg/L. Carbon disulfide was purchased as a solution at 100 mg/L (LGC, UK), was diluted in methanol to obtain working solutions at increasing concentrations ranging from 0.2 to 10 mg/L. These standard solutions were used to spike dithiocarbamate-free urine for the preparation of the calibration curve standards. Five calibration standards between the higher LOQ and the lower LOQ (namely, between 10 to 500 µg/L) were necessary for the calibration.

Tin(II)chloride (SnCl_2_) purchased from PROLABO (France), was dissolved in 5 M HCl to obtain a working solution of 5 g/L with this solution being used for the acid hydrolysis. To 2 mL samples of urine, 100 µL of IS and 3 mL of the SnCl_2_ reagent were added in headspace glass tubes and were crimped. Samples were agitated for a few second and then incubated for 15 min at 100 °C. The samples were then injected into the HS-GC-MS system.

The HS-GC-MS system included a Perkin Elmer TurboMatrix HS 40 and Shimadzu QP 2010 quadripole mass spectrometer. Chromatographic separations were performed on a RTX1 column (30 m x 0.32 mm x 4 µm) (RESTEK, France). Carrier gas was helium. For separation, temperature was increased from 50 °C to 200 °C in 9 min. Identification and quantification of carbon disulfide were performed in impact electronic ionization mode using SIM of a quantifier ion (75.9) and a additional qualifier ion (77.9).
