# Supplementary material for: Impacts of dietary exposure to pesticides on faecal microbiome metabolism in adult twins
Source: Environ Health. 2022 May 3;21:46. doi: 10.1186/s12940-022-00860-0 (PMC9063241; doi:10.1186/s12940-022-00860-0)
Supplement: Supplementary file 4 — Additional file 4. [file 12940_2022_860_MOESM4_ESM.pdf]

GenomeScan Report

Next Generation Sequencing

	Robin Mesnage
	King's College London
Address:	Dept of Mecial and Molecular Genetics
	8th floor
	Tower Wing Guy's Hospital London UK
Project reference:	103613
Project start date:	2019-02-28
Project manager:	David van der Meer
Service type:	Illumina Next Generation Sequencing
Progress:	Finished
Document status:	v1 Final

Summary

This project report contains all information regarding submitted samples, qualitychecks, experimental procedures, and the resulting data that was generated inyour project.

A total of 130 samples were sequenced using Illumina sequencing technology. Data analysis was not part of the scope of this project

If you have any additional questions regarding this report, please do not hesitate to contact our Project Manager.

Materials

GenomeScan received in total 130 sample(s) in this batch. The samples were received in good condition on: 2019-03-05. To assess the quality of the samples, the concentration of the sample was determined using the Fragment Analyzer. Detailed quality metrics can be found in the Sample Summary table (Table 1) and the Appendix of this report. All sample(s) met our quality requirements.

Table 1. Sample information and quality metrics

GS_ID	Customer ID	RQN	Active	Entry QC (ng/ul)	Entry QC Passed
103613-001-001	60341	N/A	Yes	106.77	Yes
103613-001-002	85581	N/A	Yes	10.28	Yes
103613-001-003	79842	N/A	Yes	6.78	Yes
103613-001-004	73031	N/A	Yes	47.87	Yes
103613-001-005	79841	N/A	Yes	5.09	Yes
103613-001-006	54851	N/A	Yes	95.25	Yes
103613-001-007	79021	N/A	Yes	52.77	Yes
103613-001-008	51841	N/A	Yes	136.65	Yes
103613-001-009	81271	N/A	Yes	102.60	Yes
103613-001-010	76471	N/A	Yes	154.97	Yes
103613-001-011	50391	N/A	Yes	43.04	Yes

GS_ID	Customer ID	RQN	Active	Entry QC (ng/ul)	Entry QC Passed
103613-001-012	85462	N/A	Yes	69.16	Yes
103613-001-013	90692	N/A	Yes	138.77	Yes
103613-001-014	90691	N/A	Yes	21.55	Yes
103613-001-015	25022	N/A	Yes	18.83	Yes
103613-001-016	51842	N/A	Yes	75.26	Yes
103613-001-017	73532	N/A	Yes	79.37	Yes
103613-001-018	7112	N/A	Yes	4.32	Yes
103613-001-019	81202	N/A	Yes	62.35	Yes
103613-001-020	11332	N/A	Yes	159.30	Yes
103613-001-021	23932	N/A	Yes	66.28	Yes
103613-001-022	68172	N/A	Yes	175.14	Yes
103613-001-023	NC1	N/A	Yes	2.36	Yes
103613-001-024	PC3	N/A	Yes	25.85	Yes
103613-001-025	11331	N/A	Yes	76.24	Yes
103613-001-026	76841	N/A	Yes	277.39	Yes
103613-001-027	80971	N/A	Yes	246.14	Yes
103613-001-028	25021	N/A	Yes	121.82	Yes
103613-001-029	23191	N/A	Yes	3.52	Yes
103613-001-030	85582	N/A	Yes	64.01	Yes
103613-001-031	23192	N/A	Yes	328.97	Yes

GS_ID	Customer ID	RQN	Active	Entry QC (ng/ul)	Entry QC Passed
103613-001-032	96512	N/A	Yes	195.22	Yes
103613-001-033	64811	N/A	Yes	180.13	Yes
103613-001-034	58481	N/A	Yes	118.17	Yes
103613-001-035	80972	N/A	Yes	57.78	Yes
103613-001-036	23101	N/A	Yes	146.06	Yes
103613-001-037	10891	N/A	Yes	12.96	Yes
103613-001-038	83012	N/A	Yes	33.23	Yes
103613-001-039	83641	N/A	Yes	220.38	Yes
103613-001-040	81272	N/A	Yes	169.65	Yes
103613-001-041	23931	N/A	Yes	232.35	Yes
103613-001-042	60342	N/A	Yes	14.89	Yes
103613-001-043	64812	N/A	Yes	150.02	Yes
103613-001-044	85521	N/A	Yes	152.93	Yes
103613-001-045	85461	N/A	Yes	23.27	Yes
103613-001-046	76472	N/A	Yes	124.64	Yes
103613-001-047	PC2	N/A	Yes	32.37	Yes
103613-001-048	NC2	N/A	Yes	2.02	Yes
103613-001-049	83022	N/A	Yes	7.34	Yes
103613-001-050	23102	N/A	Yes	10.33	Yes
103613-001-051	6511	N/A	Yes	186.95	Yes

GS_ID	Customer ID	RQN	Active	Entry QC (ng/ul)	Entry QC Passed
103613-001-052	57992	N/A	Yes	14.49	Yes
103613-001-053	23801	N/A	Yes	5.88	Yes
103613-001-054	90702	N/A	Yes	160.79	Yes
103613-001-055	58482	N/A	Yes	116.97	Yes
103613-001-056	54852	N/A	Yes	191.61	Yes
103613-001-057	76262	N/A	Yes	101.98	Yes
103613-001-058	23802	N/A	Yes	9.91	Yes
103613-001-059	83802	N/A	Yes	5.28	Yes
103613-001-060	73111	N/A	Yes	123.68	Yes
103613-001-061	85522	N/A	Yes	97.34	Yes
103613-001-062	73531	N/A	Yes	259.62	Yes
103613-001-063	73512	N/A	Yes	25.22	Yes
103613-001-064	6512	N/A	Yes	4.03	Yes
103613-001-065	73511	N/A	Yes	7.90	Yes
103613-001-066	7431	N/A	Yes	242.86	Yes
103613-001-067	68171	N/A	Yes	8.48	Yes
103613-001-068	34371	N/A	Yes	166.98	Yes
103613-001-069	57991	N/A	Yes	148.58	Yes
103613-001-070	83011	N/A	Yes	39.56	Yes
103613-001-071	73112	N/A	Yes	27.62	Yes

GS_ID	Customer ID	RQN	Active	Entry QC (ng/ul)	Entry QC Passed
103613-001-072	90701	N/A	Yes	230.10	Yes
103613-001-073	96022	N/A	Yes	278.20	Yes
103613-001-074	10892	N/A	Yes	6.09	Yes
103613-001-075	78842	N/A	Yes	31.76	Yes
103613-001-076	24091	N/A	Yes	215.40	Yes
103613-001-077	90112	N/A	Yes	7.21	Yes
103613-001-078	83801	N/A	Yes	34.21	Yes
103613-001-079	7432	N/A	Yes	111.64	Yes
103613-001-080	94272	N/A	Yes	293.50	Yes
103613-001-081	24092	N/A	Yes	242.05	Yes
103613-001-082	4632	N/A	Yes	8.23	Yes
103613-001-083	57851	N/A	Yes	134.56	Yes
103613-001-084	94442	N/A	Yes	336.54	Yes
103613-001-085	81391	N/A	Yes	4.10	Yes
103613-001-086	57852	N/A	Yes	54.42	Yes
103613-001-087	33051	N/A	Yes	139.28	Yes
103613-001-088	83021	N/A	Yes	32.19	Yes
103613-001-089	82461	N/A	Yes	11.95	Yes
103613-001-090	32952	N/A	Yes	3.09	Yes
103613-001-091	81392	N/A	Yes	283.12	Yes

GS_ID	Customer ID	RQN	Active	Entry QC (ng/ul)	Entry QC Passed
103613-001-092	4631	N/A	Yes	50.53	Yes
103613-001-093	96021	N/A	Yes	159.46	Yes
103613-001-094	58101	N/A	Yes	263.78	Yes
103613-001-095	NC3	N/A	Yes	1.56	Yes
103613-001-096	PC3	N/A	Yes	19.47	Yes
103613-001-097	64231	N/A	Yes	29.31	Yes
103613-001-098	58102	N/A	Yes	6.13	Yes
103613-001-099	16372	N/A	Yes	15.92	Yes
103613-001-100	66982	N/A	Yes	13.29	Yes
103613-001-101	32951	N/A	Yes	3.27	Yes
103613-001-102	51442	N/A	Yes	8.44	Yes
103613-001-103	16302	N/A	Yes	8.20	Yes
103613-001-104	81201	N/A	Yes	116.34	Yes
103613-001-105	59341	N/A	Yes	7.06	Yes
103613-001-106	82462	N/A	Yes	8.79	Yes
103613-001-107	83642	N/A	Yes	13.45	Yes
103613-001-108	9972	N/A	Yes	10.31	Yes
103613-001-109	90111	N/A	Yes	6.94	Yes
103613-001-110	9971	N/A	Yes	3.13	Yes
103613-001-111	76261	N/A	Yes	113.18	Yes

GS_ID	Customer ID	RQN	Active	Entry QC (ng/ul)	Entry QC Passed
103613-001-112	17232	N/A	Yes	167.76	Yes
103613-001-113	76842	N/A	Yes	49.18	Yes
103613-001-114	NC4	N/A	Yes	3.45	Yes
103613-001-115	PC4	N/A	Yes	31.86	Yes
103613-001-116	82731	N/A	Yes	177.74	Yes
103613-001-117	94271	N/A	Yes	31.83	Yes
103613-001-118	201	N/A	Yes	8.49	Yes
103613-001-119	95882	N/A	Yes	71.02	Yes
103613-001-120	33052	N/A	Yes	21.76	Yes
103613-001-121	34372	N/A	Yes	170.19	Yes
103613-001-122	17231	N/A	Yes	125.41	Yes
103613-001-123	59342	N/A	Yes	4.49	Yes
103613-001-124	96511	N/A	Yes	166.25	Yes
103613-001-125	73032	N/A	Yes	3.72	Yes
103613-001-126	94441	N/A	Yes	163.80	Yes
103613-001-127	67751	N/A	Yes	21.99	Yes
103613-001-128	50392	N/A	Yes	156.47	Yes
103613-001-129	95881	N/A	Yes	127.20	Yes
103613-001-130	64232	N/A	Yes	14.59	Yes
103613-001-131	67752	N/A	Yes	92.49	Yes

GS_ID	Customer ID	RQN	Active	Entry QC (ng/ul)	Entry QC Passed
103613-001-132	79022	N/A	Yes	15.91	Yes
103613-001-133	16371	N/A	Yes	4.77	Yes
103613-001-134	66981	N/A	Yes	7.65	Yes
103613-001-135	78841	N/A	Yes	102.71	Yes
103613-001-136	51441	N/A	Yes	18.25	Yes
103613-001-137	16301	N/A	Yes	2.59	Yes
103613-001-138	7111	N/A	Yes	49.10	Yes

Experimental procedures

The NEBNext® Ultra II FS DNA module (cat# NEB #E7810S/L) and the NEBNext® Ultra II Ligation module (cat# NEB #E7595S/L) were used to process the samples. Fragmentation, A-tailing and ligation of sequencing adapters of the resulting product was performed according to the procedure described in the NEBNext Ultra II FS DNA module and NEBNext Ultra II Ligation module Instruction Manual. The quality and yield after sample preparation was measured with the Fragment Analyzer (Table 2 and Appendices). The size of the resulting product was consistent with the expected size of approximately 500-700 bp.

Table 2. Library construction

GS_ID	Customer ID	Molarity (nM)	Index/Barcode	Prep QC Passed
103613-001-001	60341	66.13	dIDT97	Yes
103613-001-001	60341	31.66	dIDT97	Yes
103613-001-002	85581	4.33	dIDT98	Yes
103613-001-002	85581	1.49	dIDT98	Yes
103613-001-003	79842	0.92	dIDT99	Yes
103613-001-003	79842	4.65	dIDT99	Yes
103613-001-004	73031	46.36	dIDT100	Yes

GS_ID	Customer ID	Molarity (nM)	Index/Barcode	Prep QC Passed
103613-001-004	73031	7.37	dIDT100	Yes
103613-001-005	79841	0.40	dIDT101	Yes
103613-001-005	79841	4.74	dIDT101	Yes
103613-001-006	54851	7.58	dIDT102	Yes
103613-001-006	54851	60.44	dIDT102	Yes
103613-001-007	79021	10.00	dIDT103	Yes
103613-001-007	79021	59.08	dIDT103	Yes
103613-001-008	51841	7.60	dIDT104	Yes
103613-001-008	51841	68.05	dIDT104	Yes
103613-001-009	81271	27.12	dIDT105	Yes
103613-001-009	81271	45.66	dIDT105	Yes
103613-001-010	76471	57.35	dIDT10	Yes
103613-001-011	50391	43.98	dIDT11	Yes
103613-001-012	85462	68.04	dIDT12	Yes
103613-001-013	90692	56.71	dIDT13	Yes
103613-001-014	90691	6.22	dIDT14	Yes
103613-001-015	25022	4.52	dIDT15	Yes
103613-001-016	51842	57.59	dIDT106	Yes
103613-001-016	51842	21.18	dIDT106	Yes
103613-001-017	73532	19.87	dIDT107	Yes

GS_ID	Customer ID	Molarity (nM)	Index/Barcode	Prep QC Passed
103613-001-017	73532	58.31	dIDT107	Yes
103613-001-018	7112	29.09	dIDT108	Yes
103613-001-018	7112	14.54	dIDT108	Yes
103613-001-019	81202	100.01	dIDT19	Yes
103613-001-020	11332	118.84	dIDT20	Yes
103613-001-021	23932	47.37	dIDT21	Yes
103613-001-022	68172	75.65	dIDT22	Yes
103613-001-023	NC1	1.21	dIDT23	Yes
103613-001-024	PC3	26.31	dIDT24	Yes
103613-001-025	11331	8.62	dIDT109	Yes
103613-001-025	11331	65.52	dIDT109	Yes
103613-001-026	76841	59.27	dIDT26	Yes
103613-001-027	80971	68.52	dIDT27	Yes
103613-001-028	25021	74.92	dIDT28	Yes
103613-001-029	23191	6.38	dIDT29	Yes
103613-001-030	85582	77.37	dIDT30	Yes
103613-001-031	23192	59.70	dIDT31	Yes
103613-001-032	96512	65.57	dIDT32	Yes
103613-001-033	64811	7.81	dIDT110	Yes
103613-001-033	64811	61.78	dIDT110	Yes

GS_ID	Customer ID	Molarity (nM)	Index/Barcode	Prep QC Passed
103613-001-034	58481	64.48	dIDT34	Yes
103613-001-035	80972	76.25	dIDT35	Yes
103613-001-036	23101	78.87	dIDT36	Yes
103613-001-037	10891	5.10	dIDT37	Yes
103613-001-038	83012	32.55	dIDT38	Yes
103613-001-039	83641	53.22	dIDT39	Yes
103613-001-040	81272	74.07	dIDT40	Yes
103613-001-041	23931	77.17	dIDT111	Yes
103613-001-041	23931	9.91	dIDT111	Yes
103613-001-042	60342	5.12	dIDT42	Yes
103613-001-043	64812	65.76	dIDT43	Yes
103613-001-044	85521	60.40	dIDT44	Yes
103613-001-045	85461	5.59	dIDT45	Yes
103613-001-046	76472	77.18	dIDT46	Yes
103613-001-047	PC2	29.25	dIDT47	Yes
103613-001-048	NC2	1.23	dIDT48	Yes
103613-001-049	83022	6.24	dIDT112	Yes
103613-001-049	83022	1.26	dIDT112	Yes
103613-001-050	23102	4.44	dIDT50	Yes
103613-001-051	6511	89.26	dIDT51	Yes

GS_ID	Customer ID	Molarity (nM)	Index/Barcode	Prep QC Passed
103613-001-052	57992	6.27	dIDT52	Yes
103613-001-053	23801	4.76	dIDT53	Yes
103613-001-054	90702	67.95	dIDT54	Yes
103613-001-055	58482	86.66	dIDT55	Yes
103613-001-056	54852	80.73	dIDT56	Yes
103613-001-057	76262	56.48	dIDT113	Yes
103613-001-057	76262	10.72	dIDT113	Yes
103613-001-058	23802	5.19	dIDT58	Yes
103613-001-059	83802	6.03	dIDT59	Yes
103613-001-060	73111	58.95	dIDT60	Yes
103613-001-061	85522	59.76	dIDT61	Yes
103613-001-062	73531	64.79	dIDT62	Yes
103613-001-063	73512	24.45	dIDT63	Yes
103613-001-064	6512	3.88	dIDT64	Yes
103613-001-065	73511	1.03	dIDT114	Yes
103613-001-065	73511	3.70	dIDT114	Yes
103613-001-066	7431	56.09	dIDT66	Yes
103613-001-067	68171	4.15	dIDT67	Yes
103613-001-068	34371	58.16	dIDT68	Yes
103613-001-069	57991	106.55	dIDT69	Yes

GS_ID	Customer ID	Molarity (nM)	Index/Barcode	Prep QC Passed
103613-001-070	83011	32.20	dIDT70	Yes
103613-001-071	73112	29.78	dIDT71	Yes
103613-001-072	90701	61.81	dIDT72	Yes
103613-001-073	96022	11.42	dIDT115	Yes
103613-001-073	96022	44.96	dIDT115	Yes
103613-001-074	10892	4.54	dIDT74	Yes
103613-001-075	78842	25.00	dIDT75	Yes
103613-001-076	24091	34.39	dIDT76	Yes
103613-001-077	90112	4.77	dIDT77	Yes
103613-001-078	83801	19.91	dIDT78	Yes
103613-001-079	7432	93.42	dIDT79	Yes
103613-001-080	94272	61.14	dIDT80	Yes
103613-001-081	24092	15.35	dIDT116	Yes
103613-001-081	24092	71.18	dIDT116	Yes
103613-001-082	4632	4.58	dIDT82	Yes
103613-001-083	57851	43.54	dIDT83	Yes
103613-001-084	94442	43.83	dIDT84	Yes
103613-001-085	81391	2.84	dIDT85	Yes
103613-001-086	57852	42.92	dIDT86	Yes
103613-001-087	33051	41.91	dIDT87	Yes

GS_ID	Customer ID	Molarity (nM)	Index/Barcode	Prep QC Passed
103613-001-088	83021	28.73	dIDT88	Yes
103613-001-089	82461	3.92	dIDT117	Yes
103613-001-089	82461	0.90	dIDT117	Yes
103613-001-090	32952	2.88	dIDT90	Yes
103613-001-091	81392	47.38	dIDT91	Yes
103613-001-092	4631	39.39	dIDT92	Yes
103613-001-093	96021	57.45	dIDT93	Yes
103613-001-094	58101	52.06	dIDT94	Yes
103613-001-095	NC3	0.73	dIDT95	Yes
103613-001-096	PC3	4.88	dIDT96	Yes
103613-001-097	64231	3.42	dIDT97	Yes
103613-001-098	58102	1.01	dIDT98	Yes
103613-001-099	16372	3.41	dIDT99	Yes
103613-001-100	66982	2.77	dIDT100	Yes
103613-001-101	32951	1.14	dIDT101	Yes
103613-001-102	51442	2.58	dIDT102	Yes
103613-001-103	16302	1.93	dIDT103	Yes
103613-001-104	81201	27.16	dIDT104	Yes
103613-001-105	59341	1.15	dIDT105	Yes
103613-001-106	82462	1.55	dIDT106	Yes

GS_ID	Customer ID	Molarity (nM)	Index/Barcode	Prep QC Passed
103613-001-107	83642	1.93	dIDT107	Yes
103613-001-108	9972	4.75	dIDT108	Yes
103613-001-109	90111	0.95	dIDT109	Yes
103613-001-110	9971	1.08	dIDT110	Yes
103613-001-111	76261	26.42	dIDT111	Yes
103613-001-112	17232	8.85	dIDT112	Yes
103613-001-113	76842	20.76	dIDT113	Yes
103613-001-114	NC4	0.21	dIDT114	Yes
103613-001-115	PC4	8.10	dIDT115	Yes
103613-001-116	82731	17.27	dIDT116	Yes
103613-001-117	94271	12.61	dIDT117	Yes
103613-001-118	201	1.68	dIDT118	Yes
103613-001-119	95882	18.32	dIDT119	Yes
103613-001-120	33052	3.73	dIDT120	Yes
103613-001-121	34372	20.45	dIDT121	Yes
103613-001-122	17231	13.32	dIDT122	Yes
103613-001-123	59342	0.88	dIDT123	Yes
103613-001-124	96511	8.57	dIDT124	Yes
103613-001-125	73032	1.30	dIDT125	Yes
103613-001-126	94441	22.67	dIDT126	Yes

GS_ID	Customer ID	Molarity (nM)	Index/Barcode	Prep QC Passed
103613-001-127	67751	11.92	dIDT127	Yes
103613-001-128	50392	11.58	dIDT128	Yes
103613-001-129	95881	20.19	dIDT129	Yes
103613-001-130	64232	2.19	dIDT130	Yes
103613-001-131	67752	16.58	dIDT131	Yes
103613-001-132	79022	1.95	dIDT132	Yes
103613-001-133	16371	1.33	dIDT133	Yes
103613-001-134	66981	1.13	dIDT134	Yes
103613-001-135	78841	9.60	dIDT135	Yes
103613-001-136	51441	1.57	dIDT136	Yes
103613-001-137	16301	1.53	dIDT137	Yes
103613-001-138	7111	11.76	dIDT138	Yes

Clustering and DNA sequencing using the NovaSeq6000 was performed according to manufacturer's protocols. A concentration of 1.1 nM of DNA was used. Detailed run information per flow cell can be found in Table 3. NovaSeq control software NCS v1.6 was used.

The experiments were performed at the following site(s): GenomeScan B.V., Plesmanlaan 1d, 2333 BZ, Leiden.

Primary data analysis and results

Image analysis, base calling, and quality check was performed with the Illumina data analysis pipeline RTA3.4.4 and Bcl2fastq v2.20. Quality-filtered sequence tags are placed in the 'Raw Data' folder on the accompanying hard disk.

The flow cell information and total raw yield (Mb) for each sample are summarised in Tables 3 and 4.

Table 3. Run info

Flowcell ID	Device	Run length
HL3NFDSXX	Novaseq 6000	150-8-10-150

Flowcell ID	Device	Run length
HLFNLDSXX	Novaseq 6000	151-8-8-151
HLC37DSXX	Novaseq 6000	151-8-8-151

Table 4. Run yields per sample

GS_ID	Sample ID	Yield (Mb)	Clusters	% >=Q30
103613-001-001	60341	4,494	14,882,117	90.20
103613-001-002	85581	10,102	33,448,823	90.28
103613-001-003	79842	8,906	29,489,564	91.03
103613-001-004	73031	8,310	27,516,086	89.93
103613-001-005	79841	9,367	31,016,019	89.57
103613-001-006	54851	9,709	32,148,320	90.24
103613-001-007	79021	11,667	38,652,757	89.49
103613-001-008	51841	10,107	33,467,150	90.45
103613-001-009	81271	10,323	34,180,955	91.34
103613-001-010	76471	19,041	63,471,221	92.80
103613-001-011	50391	16,764	55,878,429	93.51
103613-001-012	85462	17,132	57,106,791	92.63
103613-001-013	90692	10,770	35,899,080	93.31
103613-001-014	90691	21,129	70,430,008	93.06
103613-001-015	25022	11,394	37,978,708	93.28
103613-001-016	51842	9,954	32,960,865	92.13

GS_ID	Sample ID	Yield (Mb)	Clusters	% >=Q30
103613-001-017	73532	8,441	27,950,780	92.03
103613-001-018	7112	5,547	18,399,607	91.54
103613-001-019	81202	9,565	31,884,998	92.00
103613-001-020	11332	19,914	66,381,512	88.50
103613-001-021	23932	7,810	26,034,029	92.25
103613-001-022	68172	8,382	27,940,911	91.88
103613-001-023	NC1	513	1,709,335	75.09
103613-001-025	11331	7,790	25,794,642	90.56
103613-001-026	76841	7,650	25,499,739	93.33
103613-001-027	80971	6,340	21,134,182	92.89
103613-001-028	25021	7,908	26,360,241	92.22
103613-001-029	23191	8,331	27,768,773	92.76
103613-001-030	85582	6,568	21,892,287	92.30
103613-001-031	23192	7,086	23,618,848	93.47
103613-001-032	96512	9,867	32,889,842	93.17
103613-001-033	64811	10,231	33,876,451	90.25
103613-001-034	58481	6,286	20,953,627	92.12
103613-001-035	80972	5,353	17,841,863	93.22
103613-001-036	23101	7,678	25,594,749	91.78
103613-001-037	10891	8,112	27,039,711	94.15

GS_ID	Sample ID	Yield (Mb)	Clusters	% >=Q30
103613-001-038	83012	9,170	30,568,317	92.47
103613-001-039	83641	8,552	28,507,000	92.95
103613-001-040	81272	8,888	29,627,969	92.47
103613-001-041	23931	12,536	41,510,769	91.43
103613-001-042	60342	14,901	49,668,427	92.48
103613-001-043	64812	7,746	25,819,044	93.44
103613-001-044	85521	9,940	33,132,212	93.03
103613-001-045	85461	12,019	39,876,325	93.47
103613-001-046	76472	8,271	27,570,112	92.08
103613-001-047	PC2	9,355	31,182,454	92.40
103613-001-049	83022	9,469	31,355,426	91.27
103613-001-050	23102	9,824	32,746,351	92.88
103613-001-051	6511	7,700	25,667,509	91.64
103613-001-052	57992	9,066	30,221,048	92.64
103613-001-053	23801	9,125	30,416,296	93.47
103613-001-054	90702	6,445	21,483,741	93.02
103613-001-055	58482	6,873	22,908,924	91.81
103613-001-056	54852	11,010	36,699,987	91.44
103613-001-057	76262	8,832	29,245,204	90.61
103613-001-058	23802	9,214	30,713,532	93.37

GS_ID	Sample ID	Yield (Mb)	Clusters	% >=Q30
103613-001-059	83802	7,717	25,723,602	93.65
103613-001-060	73111	7,785	25,950,066	93.17
103613-001-061	85522	8,175	27,250,317	93.54
103613-001-062	73531	10,097	33,657,420	92.94
103613-001-063	73512	9,098	30,325,944	93.36
103613-001-064	6512	16,760	55,866,227	93.72
103613-001-065	73511	6,975	23,097,054	91.42
103613-001-066	7431	9,532	31,772,964	93.42
103613-001-067	68171	11,609	38,696,166	93.66
103613-001-068	34371	6,168	20,561,598	93.52
103613-001-069	57991	7,721	25,735,596	92.05
103613-001-070	83011	6,853	22,842,872	93.52
103613-001-071	73112	8,155	27,184,617	93.47
103613-001-072	90701	10,982	36,607,384	93.48
103613-001-073	96022	8,894	29,451,437	91.25
103613-001-074	10892	11,608	38,692,310	93.96
103613-001-075	78842	8,575	28,584,127	93.79
103613-001-076	24091	9,931	33,103,057	92.80
103613-001-077	90112	9,088	30,292,906	93.68
103613-001-078	83801	9,092	30,305,115	93.69

GS_ID	Sample ID	Yield (Mb)	Clusters	% >=Q30
103613-001-079	7432	12,240	40,801,423	91.70
103613-001-080	94272	9,171	30,568,621	92.94
103613-001-081	24092	8,060	26,690,054	91.45
103613-001-082	4632	9,189	30,628,735	93.65
103613-001-083	57851	9,488	31,628,234	92.87
103613-001-084	94442	6,593	21,977,655	93.44
103613-001-085	81391	9,265	30,882,527	93.63
103613-001-086	57852	10,273	34,244,561	93.78
103613-001-087	33051	9,321	31,070,039	93.32
103613-001-088	83021	18,237	60,790,894	92.91
103613-001-089	82461	4,301	14,241,827	90.41
103613-001-090	32952	8,349	27,829,230	93.94
103613-001-091	81392	8,529	28,429,456	93.40
103613-001-092	4631	8,500	28,333,272	93.88
103613-001-093	96021	7,057	23,524,132	93.41
103613-001-094	58101	7,428	24,760,565	92.41
103613-001-097	64231	6,270	20,899,621	93.71
103613-001-098	58102	9,159	30,530,203	93.73
103613-001-099	16372	10,023	33,410,570	93.89
103613-001-100	66982	8,473	28,241,963	93.88

GS_ID	Sample ID	Yield (Mb)	Clusters	% >=Q30
103613-001-101	32951	6,051	20,169,530	93.66
103613-001-102	51442	9,858	32,858,360	93.80
103613-001-103	16302	10,355	34,517,748	93.80
103613-001-104	81201	12,287	40,957,267	93.98
103613-001-105	59341	6,861	22,869,496	93.56
103613-001-106	82462	9,925	33,082,903	93.93
103613-001-107	83642	6,048	20,159,918	93.12
103613-001-108	9972	13,264	44,213,859	93.78
103613-001-109	90111	6,984	23,280,720	93.29
103613-001-110	9971	5,827	19,423,101	92.35
103613-001-111	76261	12,980	43,268,316	93.87
103613-001-112	17232	23,356	77,535,454	92.77
103613-001-113	76842	18,970	62,965,490	92.39
103613-001-116	82731	16,916	56,147,909	92.16
103613-001-117	94271	17,174	56,998,192	93.18
103613-001-118	201	4,726	15,753,506	93.90
103613-001-119	95882	16,306	54,125,985	92.04
103613-001-120	33052	9,048	30,158,727	93.45
103613-001-121	34372	24,491	81,286,219	93.27
103613-001-122	17231	18,905	62,746,169	92.83

GS_ID	Sample ID	Yield (Mb)	Clusters	% >=Q30
103613-001-123	59342	6,499	21,664,513	93.91
103613-001-124	96511	20,212	67,085,370	92.51
103613-001-125	73032	11,486	38,288,143	93.97
103613-001-126	94441	22,688	75,303,758	93.06
103613-001-127	67751	31,024	102,967,650	93.76
103613-001-128	50392	19,519	64,786,870	92.77
103613-001-129	95881	23,233	77,108,537	92.88
103613-001-130	64232	6,928	23,016,932	93.38
103613-001-131	67752	26,742	88,764,603	93.41
103613-001-132	79022	8,904	29,680,373	93.30
103613-001-133	16371	6,703	22,343,363	93.87
103613-001-134	66981	6,308	20,951,616	93.34
103613-001-135	78841	21,279	70,629,697	92.99
103613-001-136	51441	5,204	17,345,452	93.89
103613-001-137	16301	5,826	19,359,048	93.50
103613-001-138	7111	19,166	63,619,836	93.31

The raw data in the 'Raw data' directory are coded with the flow cell numbers and the per sample data can be found within these run folders.

Deviations, additions, exclusions from the test method

This project was performed in compliance with the requirements and specifications set by GenomeScan and the customer. All data are within specifications.

There were no deviations from the standard test procedures.

Data transfer and formats

The data files are stored on the accompanying data disk as part of the results delivery. The raw data can be found in the 'Raw data' folder on the disk. The report can be found in the 'Reports' folder. If data analysis is performed, the results can be found in the analysis folder.

All files provided are in either plain text (.txt), tab-delimited text (.tab, .txt), PDF format (.pdf), or OpenDocument format(.odt, .ods). Plain text files can be opened in any text editor although some files for the Next Generation Sequencing service may be too large and an editor optimised for large files is required. Tab-delimited files can be opened in any text editor or spreadsheet application. PDF files require Adobe Reader to open and OpenDocument files can be opened in Microsoft Office 2007 SP2 or later versions.

Tab delimited files (.tab) are formatted using a dot as decimal separator and without thousand separators. They should be opened using an English locale to avoid incorrect translation of decimal and thousand separators.

This project report was digitally signed by:

Gerard Gathier
Technician

Stephanie van den Oever
Project Manager

Date: 2019-07-22

Fragment Analyzer Project Summary:

Data Files: 2019 06 18 13H 16M.raw, 2019 06 18 14H 35M.raw

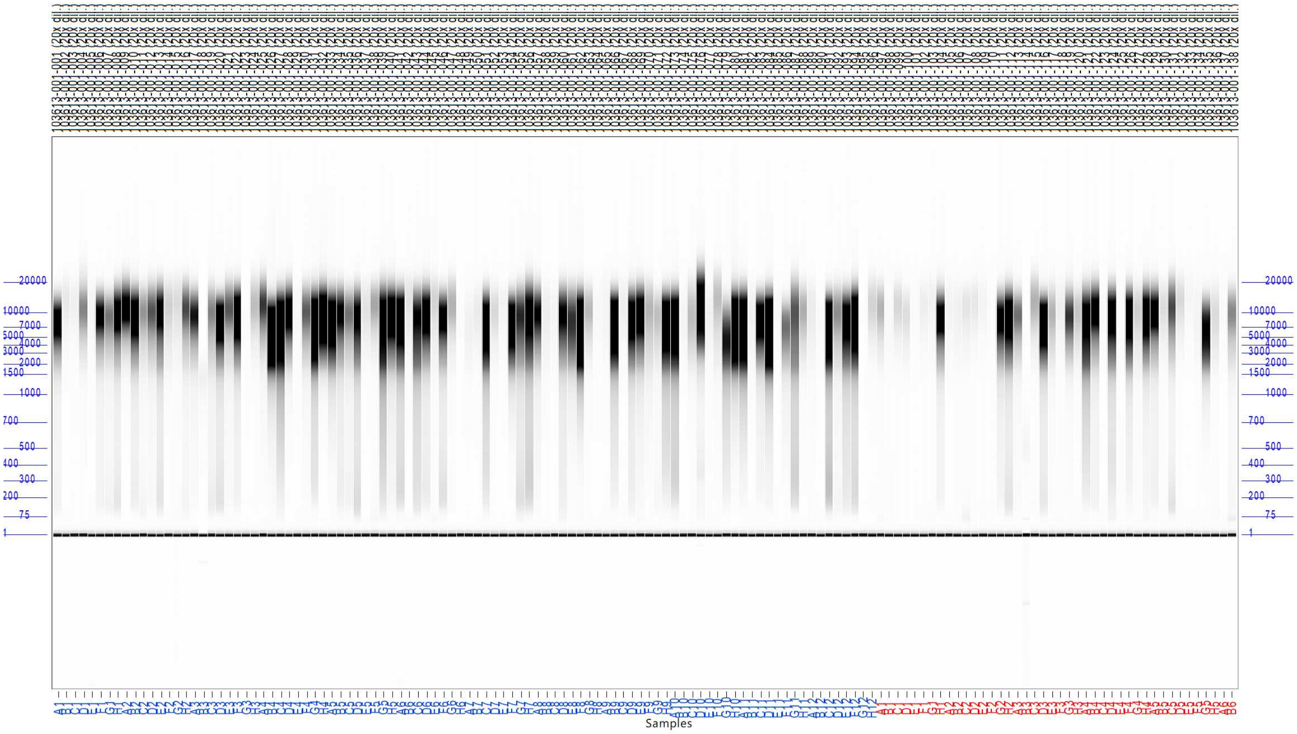

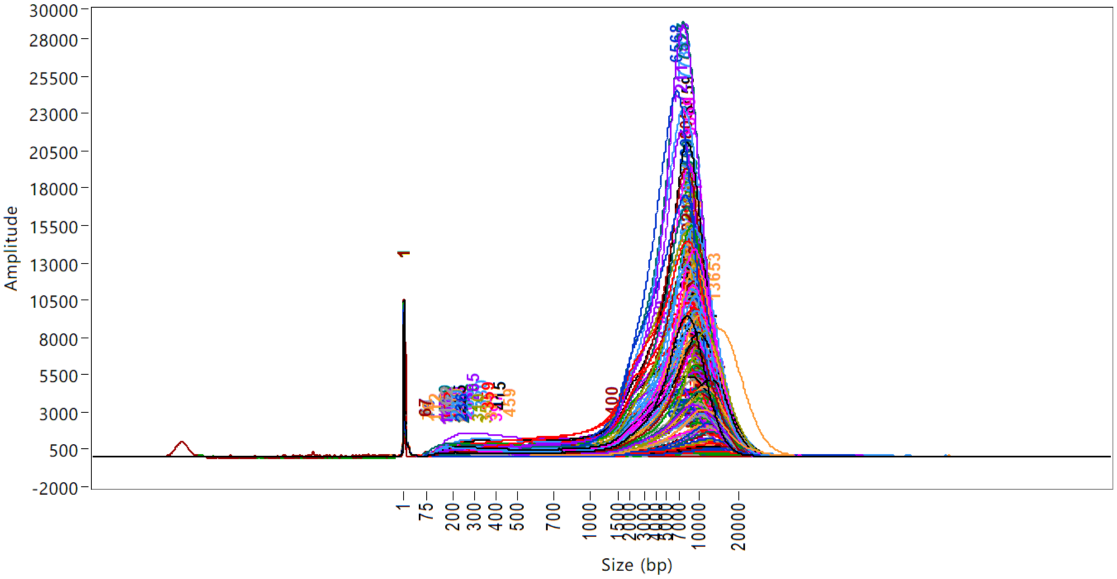

 Copyright 2015 Advanced Analytical Technologies, Inc.

Filename and Data Path: X:\Lopende Opdrachten\GAII\103613\EntryQC\EntryQC_gDNA HS_103613(pl1) 13-16-27\2019 06 18 13H 16M.raw
Created: Tuesday, June 18, 2019 1:45:41 PM
# of Capillaries: 96
Array Serial #: 111918-04SFS
Effect Length: 33 cm
Array Usage Count: 89
FA Version #: 1.2.0.11
Device Serial #: 3658

METHOD INFORMATION
Method Name: DNF-488-33 - HS Genomic DNA.mthds
Gel Prime: No
Full Conditioning: Yes
Gel Prime to Buffer: No
Gel Selection: Gel 1
Perform Prerun: 6.0 kV, 30 sec.
Rinse: No
Marker 1: No
Rinse: Tray: 3, Row: A, # Dips: 1
Sample Injection: 5.0 kV, 15 sec.
Separation: 6.0 kV, 50.0 min.
Tray Name: EntryQC_gDNA HS_103613(pl1)

Analysis Mode: gDNA

NOTE

 Copyright 2015 Advanced Analytical Technologies, Inc.

Filename and Data Path: X:\Lopende Opdrachten\GAII\103613\EntryQC\EntryQC_gDNA HS_103613(pl2)_103713_103165-032 14-35-54\2019 06 18 14H 35M.raw
Created: Tuesday, June 18, 2019 3:04:59 PM
# of Capillaries: 96
Array Serial #: 111918-04SFS
Effect Length: 33 cm
Array Usage Count: 90
FA Version #: 1.2.0.11
Device Serial #: 3658

METHOD INFORMATION
Method Name: DNF-488-33 - HS Genomic DNA.mthds
Gel Prime: No
Full Conditioning: Yes
Gel Prime to Buffer: No
Gel Selection: Gel 1
Perform Prerun: 6.0 kV, 30 sec.
Rinse: No
Marker 1: No
Rinse: Tray: 3, Row: A, # Dips: 1
Sample Injection: 5.0 kV, 15 sec.
Separation: 6.0 kV, 50.0 min.
Tray Name: EntryQC_gDNA HS_103613(pl2)_103713_103165-032

Analysis Mode: gDNA

NOTE

2019 06 18 13H 16M.raw
A1: 103613-001-001 (20x dil.)
GQN: 1.9

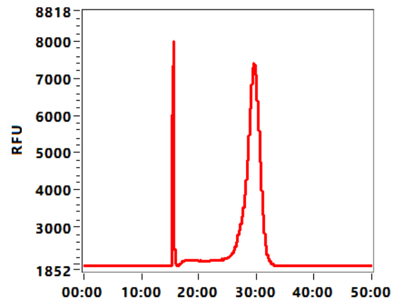

2019 06 18 13H 16M.raw
B1: 103613-001-002 (20x dil.)
GQN: 5.0

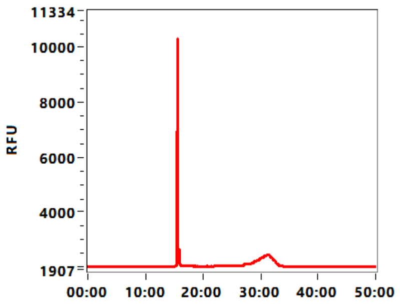

2019 06 18 13H 16M.raw
C1: 103613-001-003 (20x dil.)
GQN: 4.6

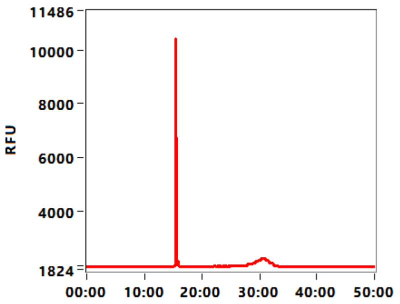

2019 06 18 13H 16M.raw
D1: 103613-001-004 (20x dil.)
GQN: 4.7

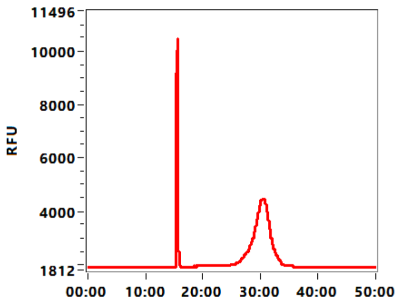

2019 06 18 13H 16M.raw
E1: 103613-001-005 (20x dil.)
GQN: 4.1

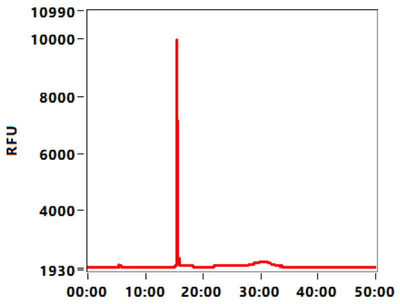

2019 06 18 13H 16M.raw
F1: 103613-001-006 (20x dil.)
GQN: 2.9

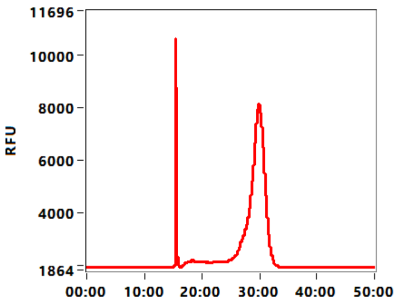

2019 06 18 13H 16M.raw
G1: 103613-001-007 (20x dil.)
GQN: 2.4

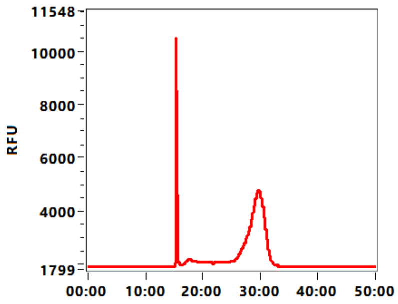

2019 06 18 13H 16M.raw
H1: 103613-001-008 (20x dil.)
GQN: 3.1

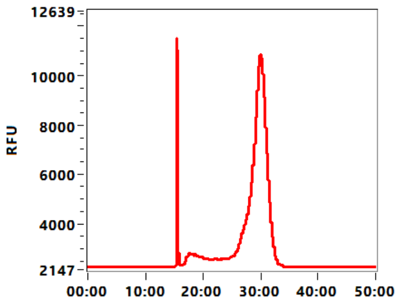

2019 06 18 13H 16M.raw
A2: 103613-001-009 (20x dil.)
GQN: 5.5

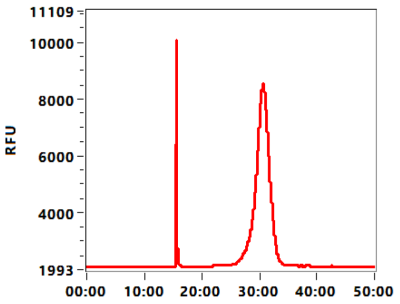

2019 06 18 13H 16M.raw
B2: 103613-001-010 (20x dil.)
GQN: 2.9

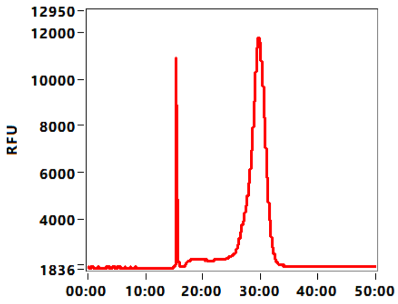

2019 06 18 13H 16M.raw
C2: 103613-001-011 (20x dil.)
GQN: 3.3

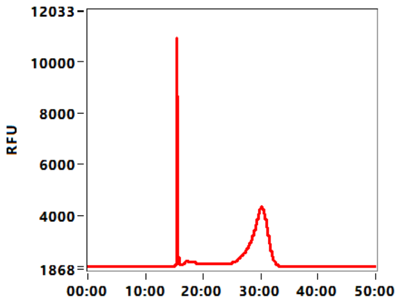

2019 06 18 13H 16M.raw
D2: 103613-001-012 (20x dil.)
GQN: 3.7

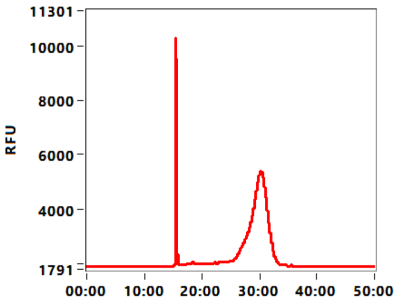

Copyright 2015 Advanced Analytical Technologies, Inc.

2019 06 18 13H 16M.raw
E2: 103613-001-013 (20x dil.)
GQN: 3.1

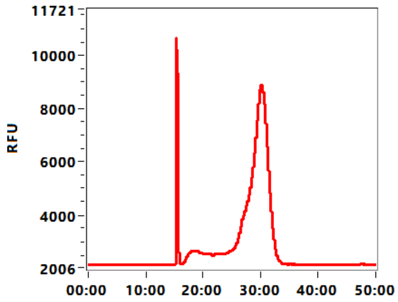

2019 06 18 13H 16M.raw
F2: 103613-001-014 (20x dil.)
GQN: 4.3

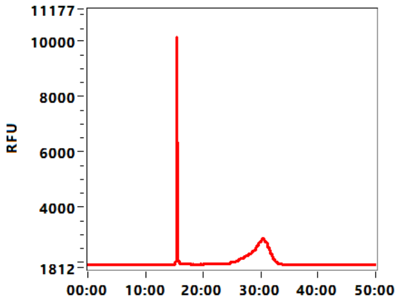

2019 06 18 13H 16M.raw
G2: 103613-001-015 (20x dil.)
GQN: 4.4

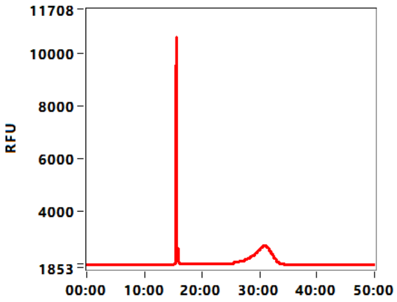

2019 06 18 13H 16M.raw
H2: 103613-001-016 (20x dil.)
GQN: 4.0

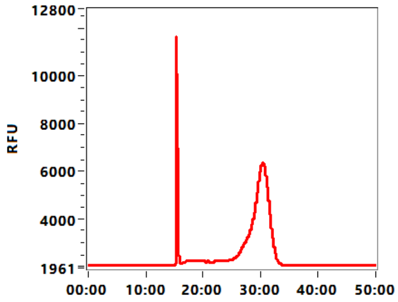

2019 06 18 13H 16M.raw
A3: 103613-001-017 (20x dil.)
GQN: 3.2

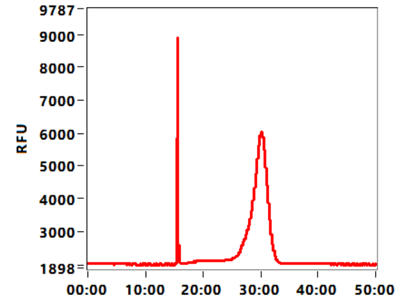

2019 06 18 13H 16M.raw
B3: 103613-001-018 (20x dil.)
GQN: 0.1

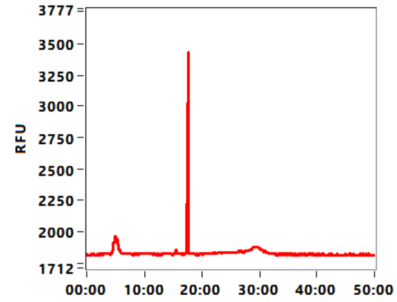

2019 06 18 13H 16M.raw
C3: 103613-001-019 (20x dil.)
GQN: 2.9

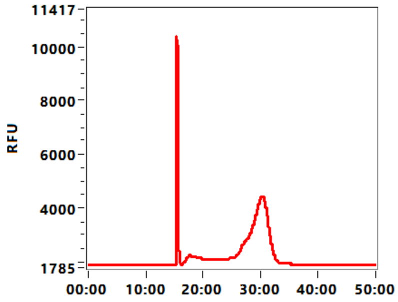

2019 06 18 13H 16M.raw
D3: 103613-001-020 (20x dil.)
GQN: 1.8

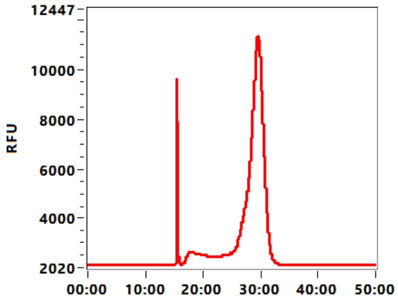

2019 06 18 13H 16M.raw
E3: 103613-001-021 (20x dil.)
GQN: 4.3

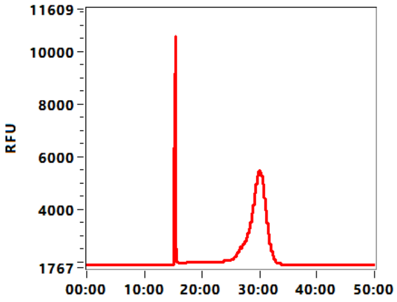

2019 06 18 13H 16M.raw
F3: 103613-001-022 (20x dil.)
GQN: 3.1

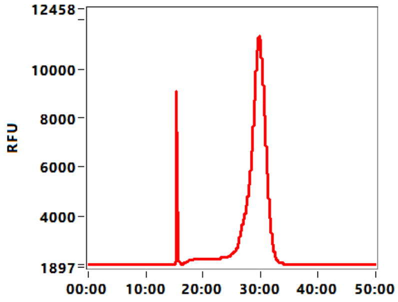

2019 06 18 13H 16M.raw
G3: 103613-001-023 (20x dil.)
GQN: 2.8

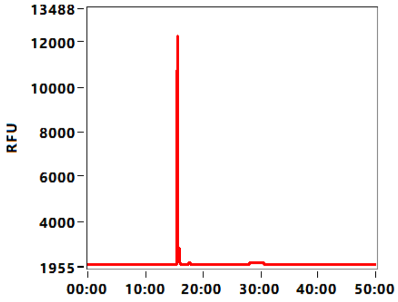

2019 06 18 13H 16M.raw
H3: 103613-001-024 (20x dil.)
GQN: 5.4

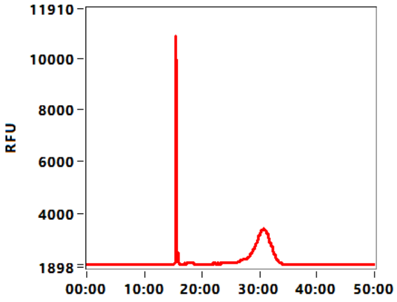

 Copyright 2015 Advanced Analytical Technologies, Inc.

2019 06 18 13H 16M.raw
A4: 103613-001-025 (20x dil.)
GQN: 5.3

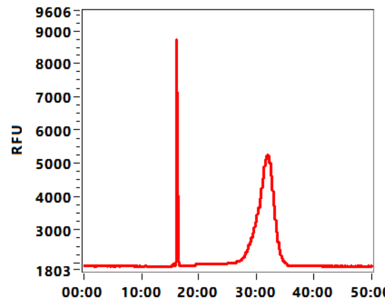

2019 06 18 13H 16M.raw
B4: 103613-001-026 (20x dil.)
GQN: 1.0

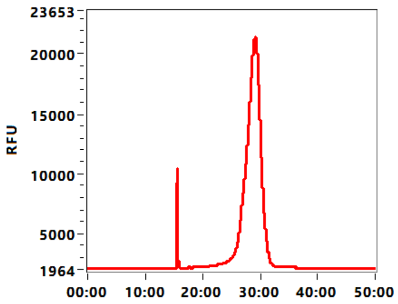

2019 06 18 13H 16M.raw
C4: 103613-001-027 (20x dil.)
GQN: 1.7

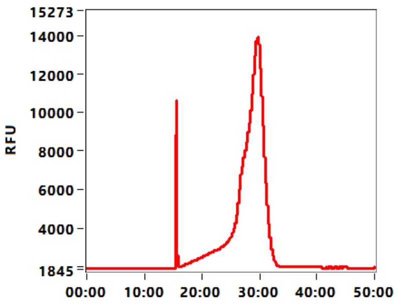

2019 06 18 13H 16M.raw
D4: 103613-001-028 (20x dil.)
GQN: 4.1

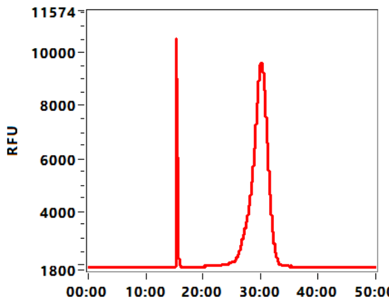

2019 06 18 13H 16M.raw
E4: 103613-001-029 (20x dil.)
GQN: 3.1

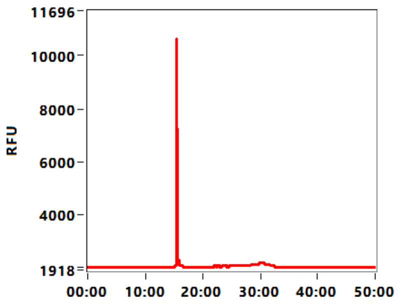

2019 06 18 13H 16M.raw
F4: 103613-001-030 (20x dil.)
GQN: 3.7

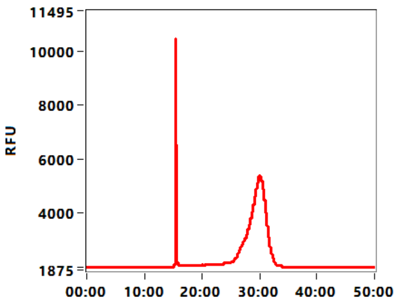

2019 06 18 13H 16M.raw
G4: 103613-001-031 (20x dil.)
GQN: 1.7

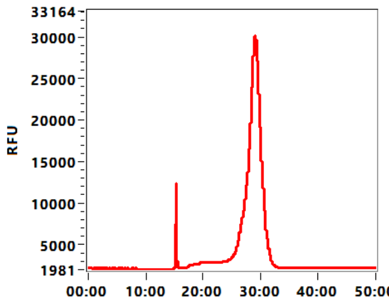

2019 06 18 13H 16M.raw
H4: 103613-001-032 (20x dil.)
GQN: 3.4

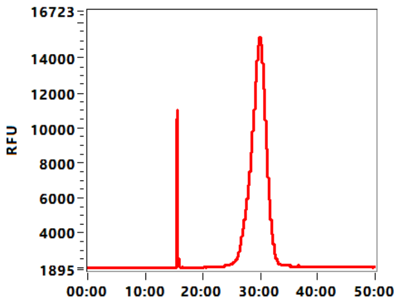

2019 06 18 13H 16M.raw
A5: 103613-001-033 (20x dil.)
GQN: 2.4

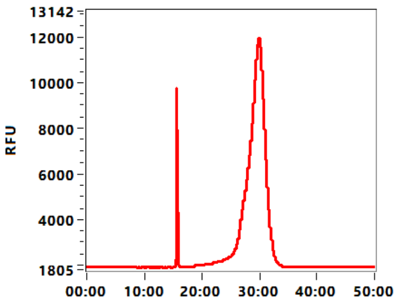

2019 06 18 13H 16M.raw
B5: 103613-001-034 (20x dil.)
GQN: 3.0

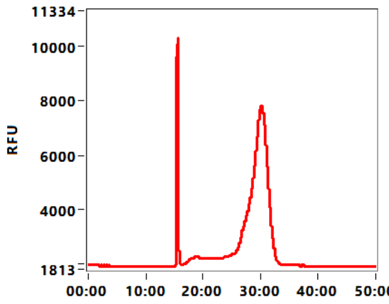

2019 06 18 13H 16M.raw
C5: 103613-001-035 (20x dil.)
GQN: 2.8

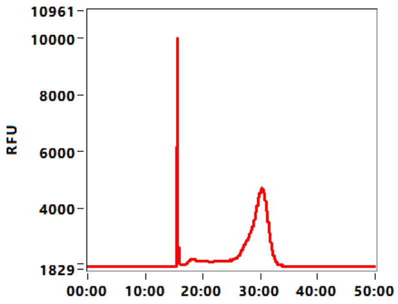

2019 06 18 13H 16M.raw
D5: 103613-001-036 (20x dil.)
GQN: 2.3

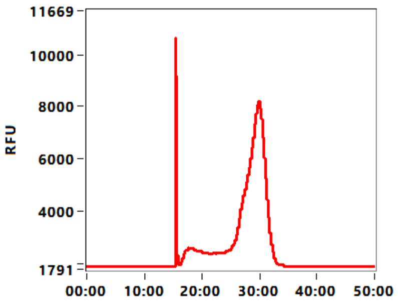

 Copyright 2015 Advanced Analytical Technologies, Inc.

2019 06 18 13H 16M.raw
E5: 103613-001-037 (20x dil.)
GQN: 4.5

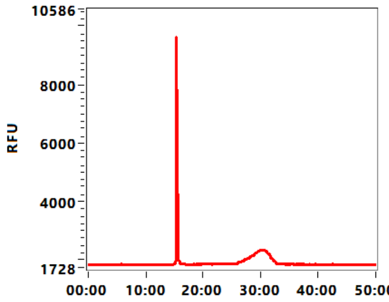

2019 06 18 13H 16M.raw
F5: 103613-001-038 (20x dil.)
GQN: 4.9

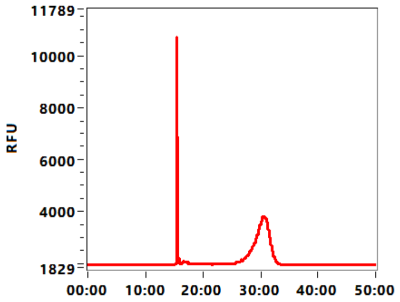

2019 06 18 13H 16M.raw
G5: 103613-001-039 (20x dil.)
GQN: 2.1

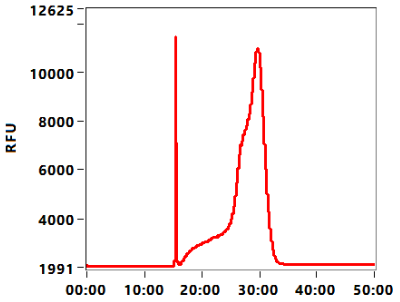

2019 06 18 13H 16M.raw
H5: 103613-001-040 (20x dil.)
GQN: 3.2

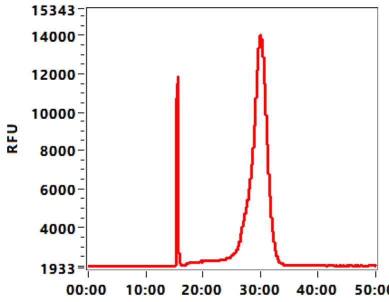

2019 06 18 13H 16M.raw
A6: 103613-001-041 (20x dil.)
GQN: 2.2

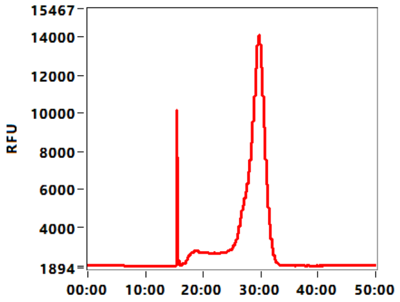

2019 06 18 13H 16M.raw
B6: 103613-001-042 (20x dil.)
GQN: 5.0

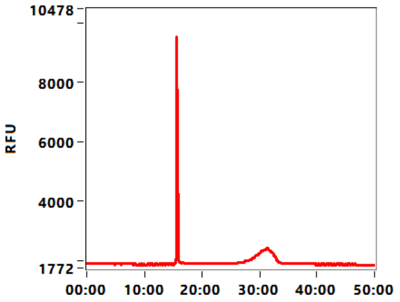

2019 06 18 13H 16M.raw
C6: 103613-001-043 (20x dil.)
GQN: 1.9

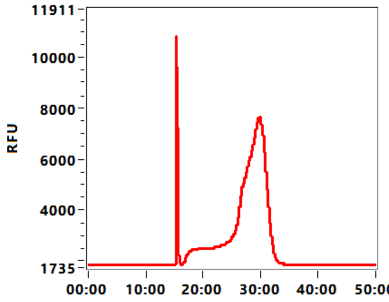

2019 06 18 13H 16M.raw
D6: 103613-001-044 (20x dil.)
GQN: 3.1

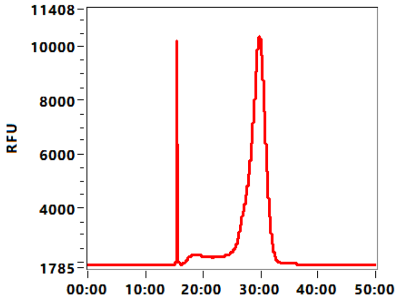

2019 06 18 13H 16M.raw
E6: 103613-001-045 (20x dil.)
GQN: 4.5

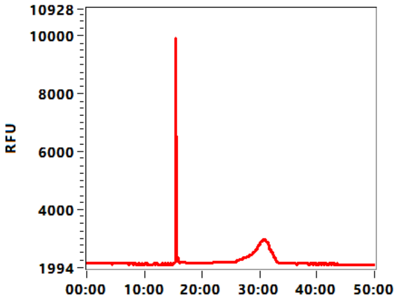

2019 06 18 13H 16M.raw
F6: 103613-001-046 (20x dil.)
GQN: 2.2

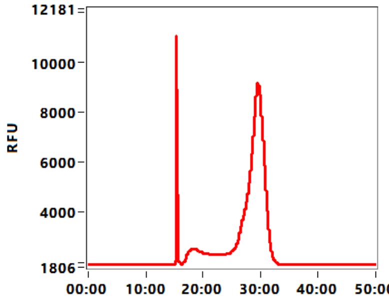

2019 06 18 13H 16M.raw
G6: 103613-001-047 (20x dil.)
GQN: 5.1

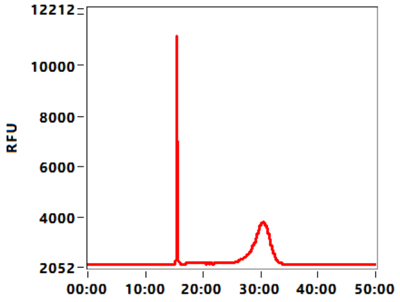

2019 06 18 13H 16M.raw
H6: 103613-001-048 (20x dil.)
GQN: 3.8

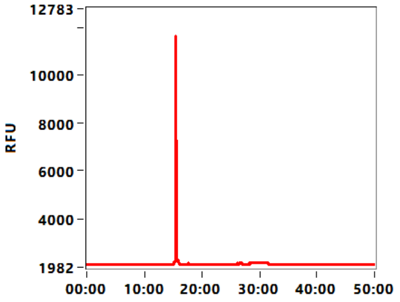

 Copyright 2015 Advanced Analytical Technologies, Inc.

2019 06 18 13H 16M.raw
A7: 103613-001-049 (20x dil.)
GQN: 2.0

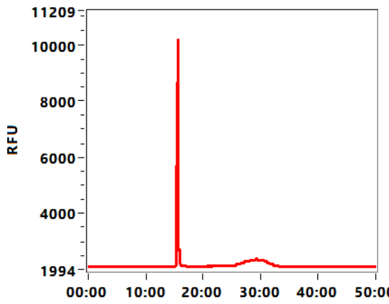

2019 06 18 13H 16M.raw
B7: 103613-001-050 (20x dil.)
GQN: 4.1

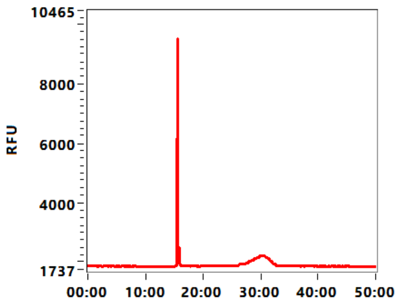

2019 06 18 13H 16M.raw
C7: 103613-001-051 (20x dil.)
GQN: 1.7

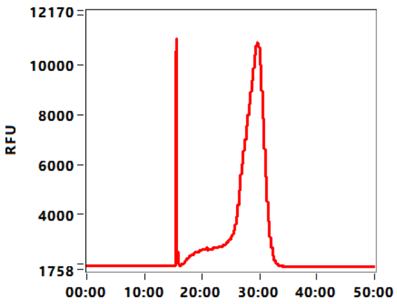

2019 06 18 13H 16M.raw
D7: 103613-001-052 (20x dil.)
GQN: 4.0

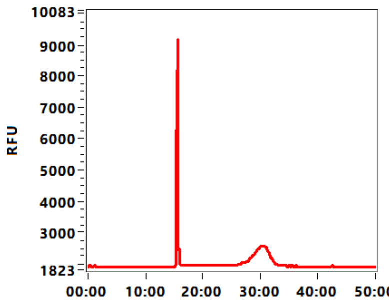

2019 06 18 13H 16M.raw
E7: 103613-001-053 (20x dil.)
GQN: 4.5

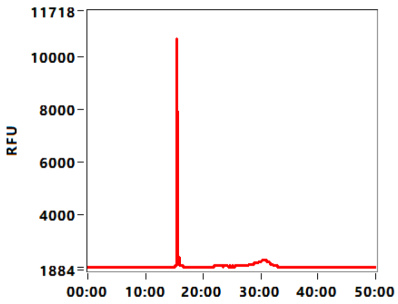

2019 06 18 13H 16M.raw
F7: 103613-001-054 (20x dil.)
GQN: 1.8

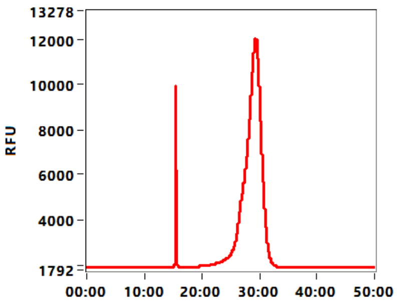

2019 06 18 13H 16M.raw
G7: 103613-001-055 (20x dil.)
GQN: 2.3

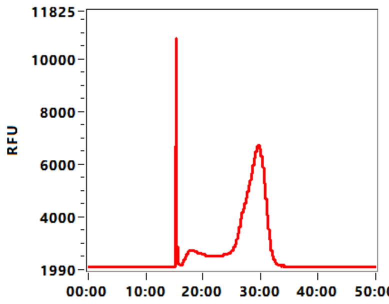

2019 06 18 13H 16M.raw
H7: 103613-001-056 (20x dil.)
GQN: 2.5

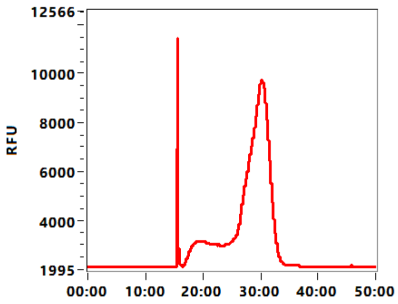

2019 06 18 13H 16M.raw
A8: 103613-001-057 (20x dil.)
GQN: 3.4

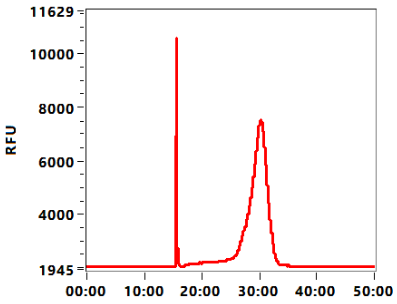

2019 06 18 13H 16M.raw
B8: 103613-001-058 (20x dil.)
GQN: 2.8

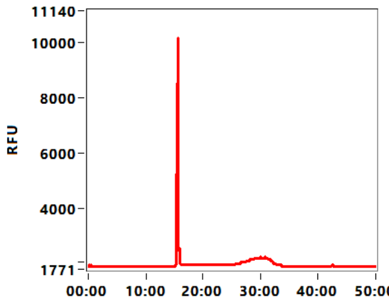

2019 06 18 13H 16M.raw
C8: 103613-001-059 (20x dil.)
GQN: 3.1

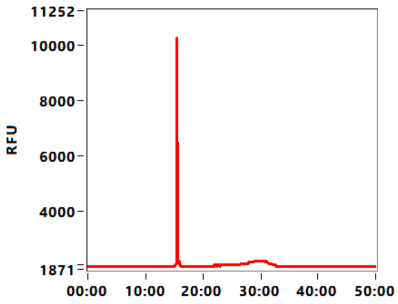

2019 06 18 13H 16M.raw
D8: 103613-001-060 (20x dil.)
GQN: 2.7

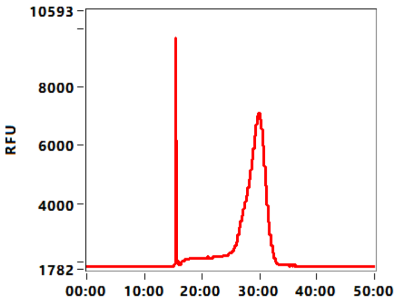

 Copyright 2015 Advanced Analytical Technologies, Inc.

2019 06 18 13H 16M.raw
E8: 103613-001-061 (20x dil.)
GQN: 2.5

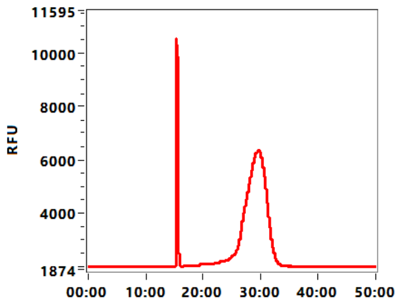

2019 06 18 13H 16M.raw
F8: 103613-001-062 (20x dil.)
GQN: 1.8

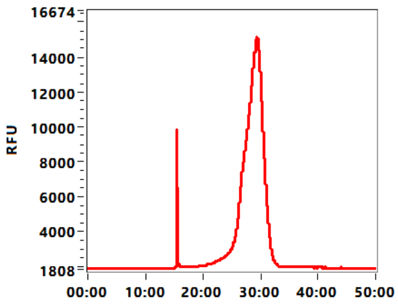

2019 06 18 13H 16M.raw
G8: 103613-001-063 (20x dil.)
GQN: 4.1

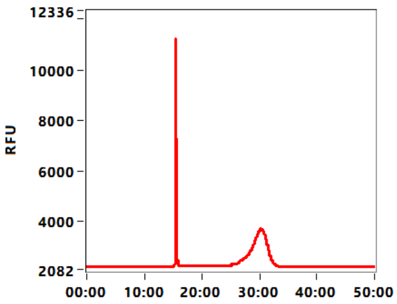

2019 06 18 13H 16M.raw
H8: 103613-001-064 (20x dil.)
GQN: 3.8

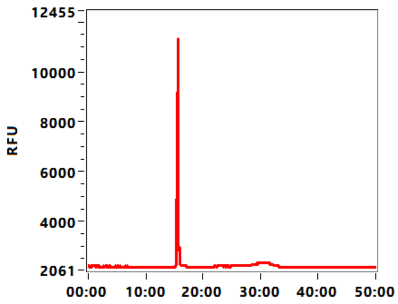

2019 06 18 13H 16M.raw
A9: 103613-001-065 (20x dil.)
GQN: 3.1

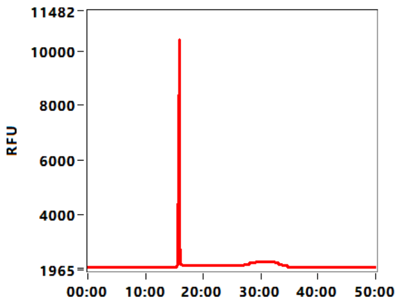

2019 06 18 13H 16M.raw
B9: 103613-001-066 (20x dil.)
GQN: 1.9

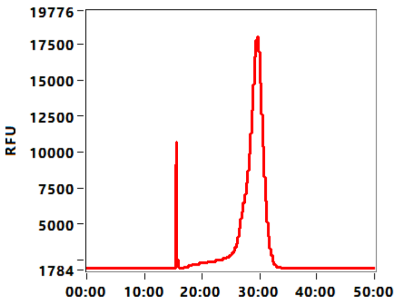

2019 06 18 13H 16M.raw
C9: 103613-001-067 (20x dil.)
GQN: 4.1

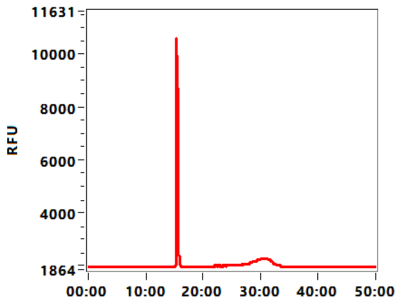

2019 06 18 13H 16M.raw
D9: 103613-001-068 (20x dil.)
GQN: 2.4

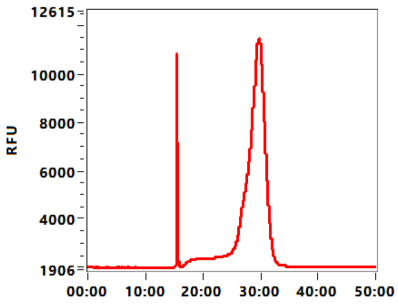

2019 06 18 13H 16M.raw
E9: 103613-001-069 (20x dil.)
GQN: 3.3

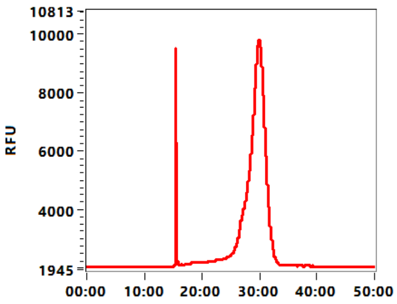

2019 06 18 13H 16M.raw
F9: 103613-001-070 (20x dil.)
GQN: 3.4

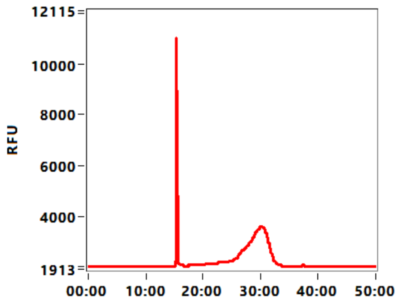

2019 06 18 13H 16M.raw
G9: 103613-001-071 (20x dil.)
GQN: 2.5

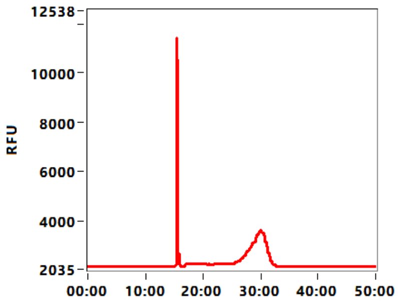

2019 06 18 13H 16M.raw
H9: 103613-001-072 (20x dil.)
GQN: 1.8

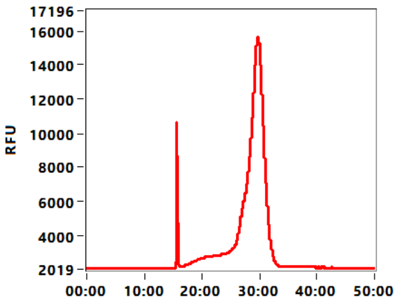

 Copyright 2015 Advanced Analytical Technologies, Inc.

2019 06 18 13H 16M.raw
A10: 103613-001-073 (20x dil.)
GQN: 1.8

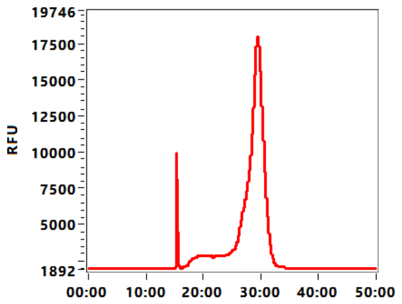

2019 06 18 13H 16M.raw
B10: 103613-001-074 (20x dil.)
GQN: 3.1

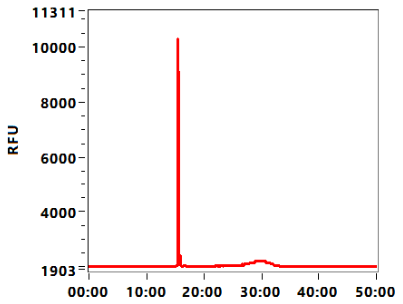

2019 06 18 13H 16M.raw
C10: 103613-001-075 (20x dil.)
GQN: 3.5

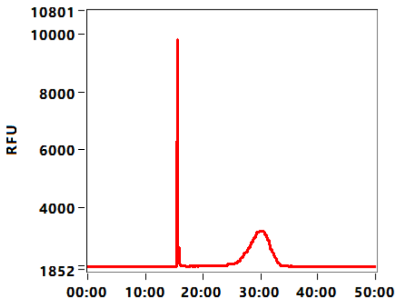

2019 06 18 13H 16M.raw
D10: 103613-001-076 (20x dil.)
GQN: 4.8

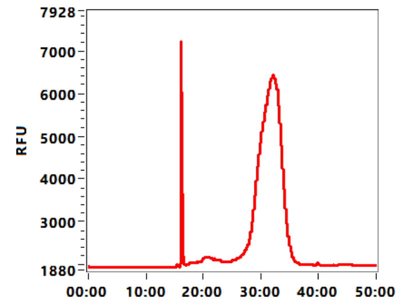

2019 06 18 13H 16M.raw
E10: 103613-001-077 (20x dil.)
GQN: 3.4

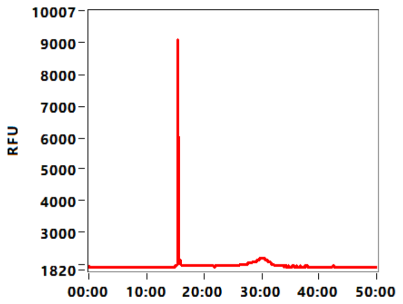

2019 06 18 13H 16M.raw
F10: 103613-001-078 (20x dil.)
GQN: 5.1

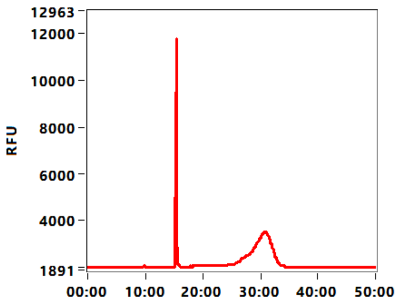

2019 06 18 13H 16M.raw
G10: 103613-001-079 (20x dil.)
GQN: 0.7

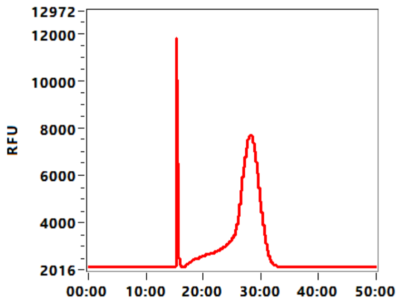

2019 06 18 13H 16M.raw
H10: 103613-001-080 (20x dil.)
GQN: 1.9

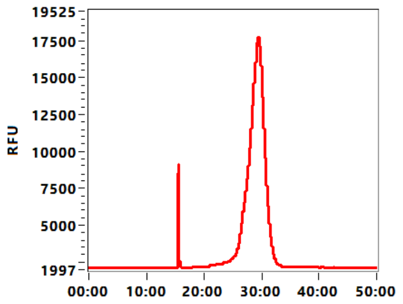

2019 06 18 13H 16M.raw
A11: 103613-001-081 (20x dil.)
GQN: 2.0

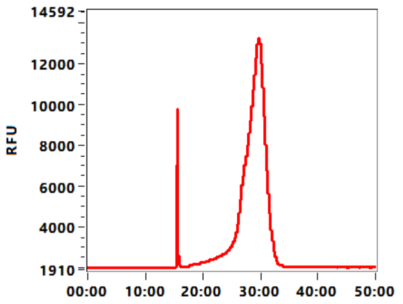

2019 06 18 13H 16M.raw
B11: 103613-001-082 (20x dil.)
GQN: 2.9

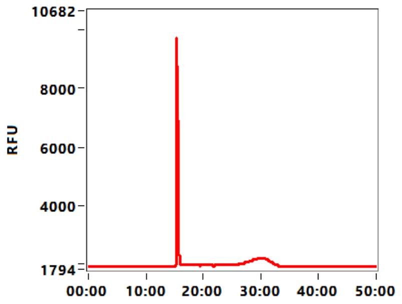

2019 06 18 13H 16M.raw
C11: 103613-001-083 (20x dil.)
GQN: 2.2

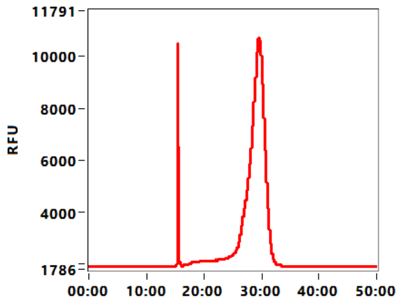

2019 06 18 13H 16M.raw
D11: 103613-001-084 (20x dil.)
GQN: 1.6

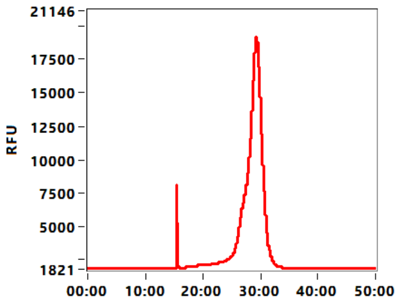

 Copyright 2015 Advanced Analytical Technologies, Inc.

2019 06 18 13H 16M.raw
E11: 103613-001-085 (20x dil.)
GQN: 3.2

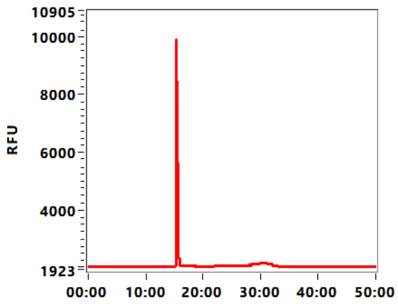

2019 06 18 13H 16M.raw
F11: 103613-001-086 (20x dil.)
GQN: 1.3

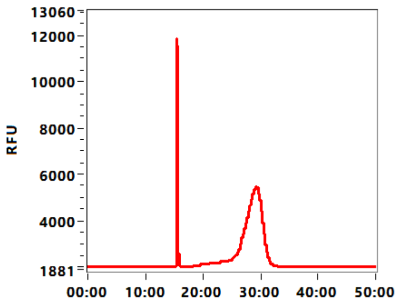

2019 06 18 13H 16M.raw
G11: 103613-001-087 (20x dil.)
GQN: 2.0

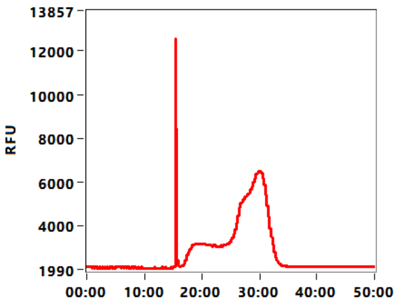

2019 06 18 13H 16M.raw
H11: 103613-001-088 (20x dil.)
GQN: 4.0

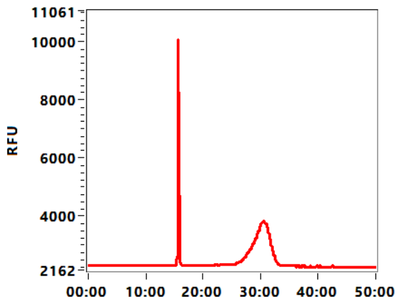

2019 06 18 13H 16M.raw
A12: 103613-001-089 (20x dil.)
GQN: 3.1

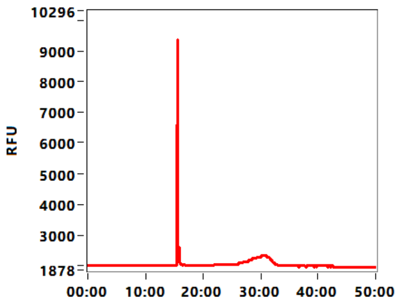

2019 06 18 13H 16M.raw
B12: 103613-001-090 (20x dil.)
GQN: 2.1

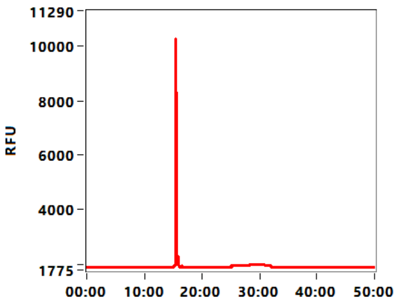

2019 06 18 13H 16M.raw
C12: 103613-001-091 (20x dil.)
GQN: 1.2

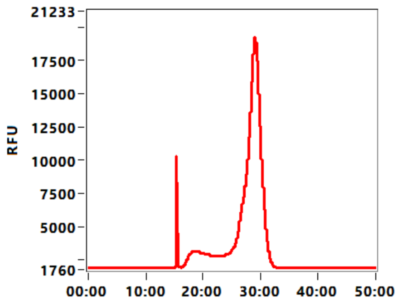

2019 06 18 13H 16M.raw
D12: 103613-001-092 (20x dil.)
GQN: 3.1

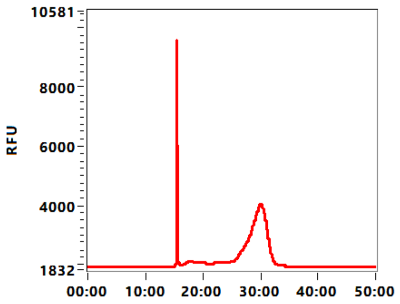

2019 06 18 13H 16M.raw
E12: 103613-001-093 (20x dil.)
GQN: 2.0

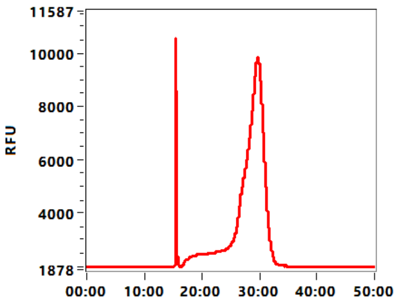

2019 06 18 13H 16M.raw
F12: 103613-001-094 (20x dil.)
GQN: 2.2

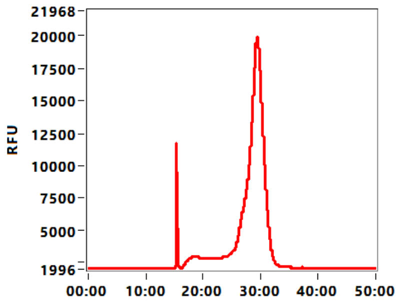

2019 06 18 13H 16M.raw
G12: 103613-001-095 (20x dil.)
GQN: 2.4

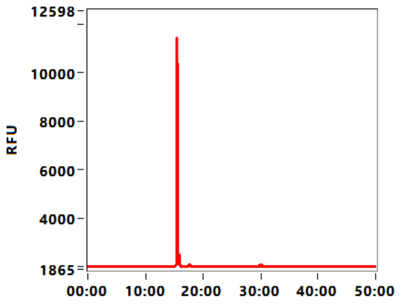

2019 06 18 13H 16M.raw
H12: 103613-001-096 (20x dil.)

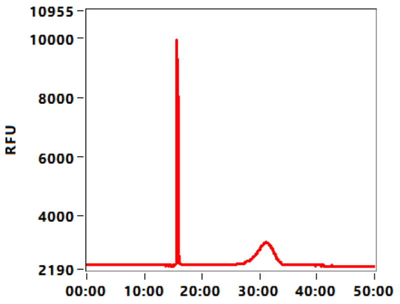

Copyright 2015 Advanced Analytical Technologies, Inc.

2019 06 18 14H 35M.raw
A1: 103613-001-097 (20x dil.)
GQN: 4.3

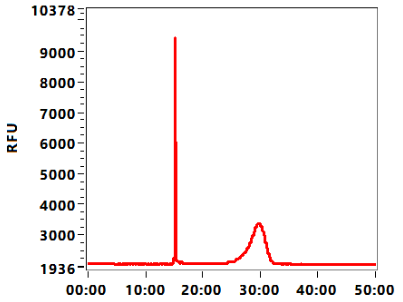

2019 06 18 14H 35M.raw
B1: 103613-001-098 (20x dil.)
GQN: 2.6

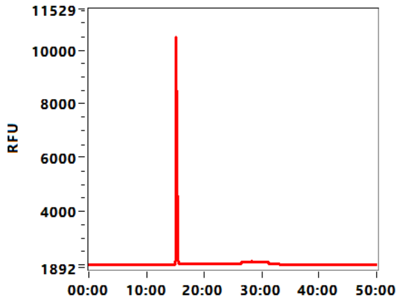

2019 06 18 14H 35M.raw
C1: 103613-001-099 (20x dil.)
GQN: 4.9

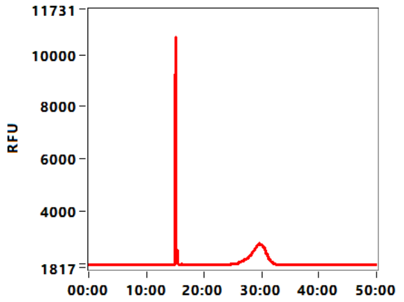

2019 06 18 14H 35M.raw
D1: 103613-001-100 (20x dil.)
GQN: 3.3

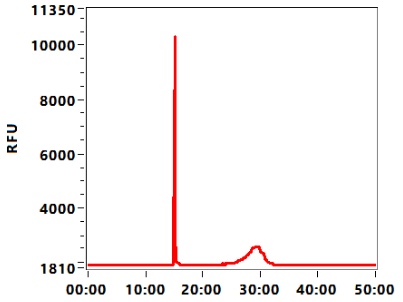

2019 06 18 14H 35M.raw
E1: 103613-001-101 (20x dil.)
GQN: 4.2

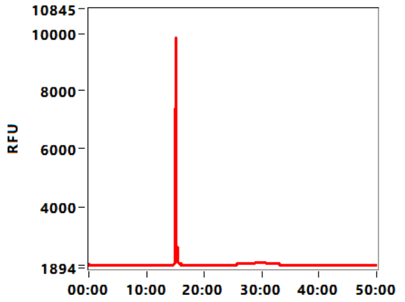

2019 06 18 14H 35M.raw
F1: 103613-001-102 (20x dil.)
GQN: 4.2

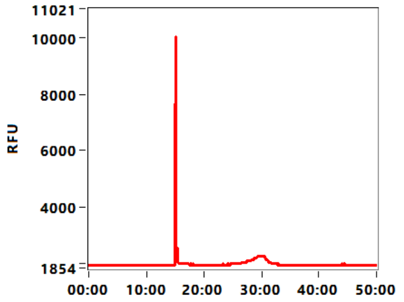

2019 06 18 14H 35M.raw
G1: 103613-001-103 (20x dil.)
GQN: 4.4

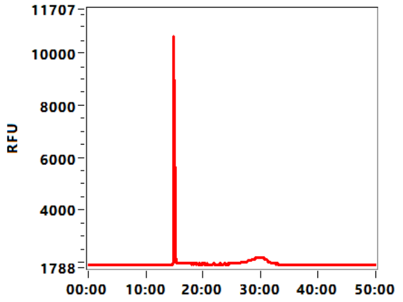

2019 06 18 14H 35M.raw
H1: 103613-001-104 (20x dil.)
GQN: 2.6

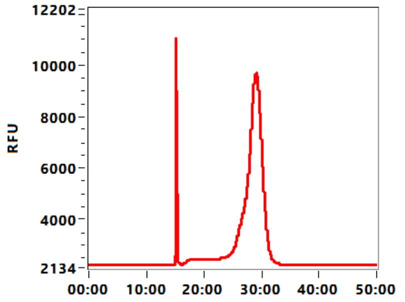

2019 06 18 14H 35M.raw
A2: 103613-001-105 (20x dil.)
GQN: 3.8

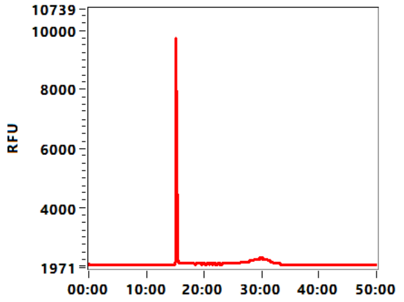

2019 06 18 14H 35M.raw
B2: 103613-001-106 (20x dil.)
GQN: 3.3

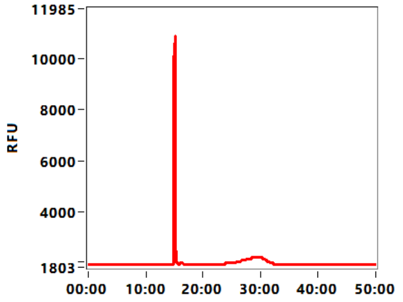

2019 06 18 14H 35M.raw
C2: 103613-001-107 (20x dil.)
GQN: 3.4

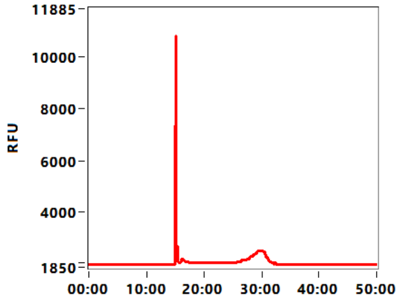

2019 06 18 14H 35M.raw
D2: 103613-001-108 (20x dil.)
GQN: 5.1

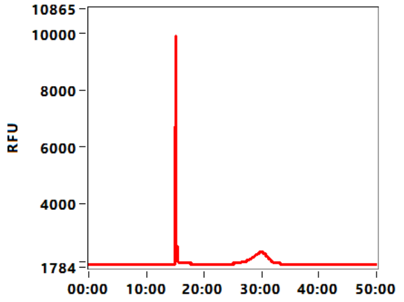

Copyright 2015 Advanced Analytical Technologies, Inc.

2019 06 18 14H 35M.raw
E2: 103613-001-109 (20x dil.)
GQN: 4.0

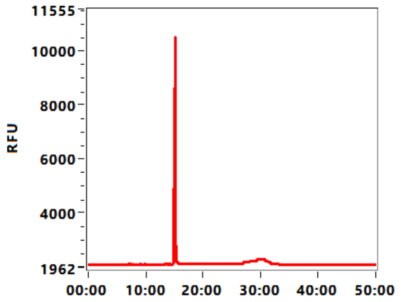

2019 06 18 14H 35M.raw
F2: 103613-001-110 (20x dil.)
GQN: 3.3

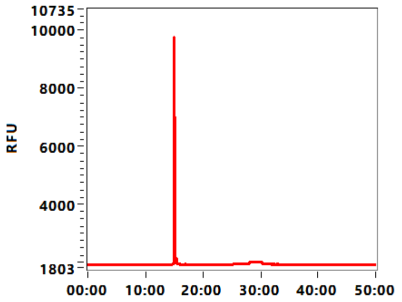

2019 06 18 14H 35M.raw
G2: 103613-001-111 (20x dil.)
GQN: 2.3

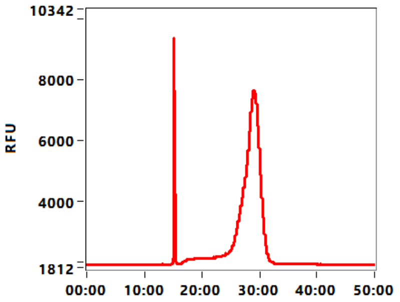

2019 06 18 14H 35M.raw
H2: 103613-001-112 (20x dil.)
GQN: 2.5

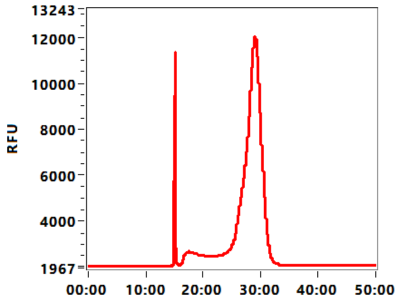

2019 06 18 14H 35M.raw
A3: 103613-001-113 (20x dil.)
GQN: 3.0

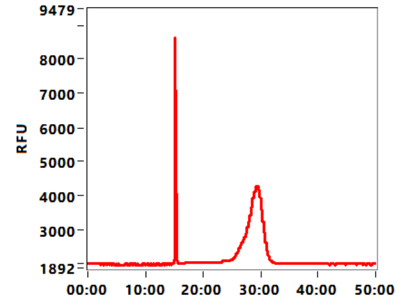

2019 06 18 14H 35M.raw
B3: 103613-001-114 (20x dil.)
GQN: 0.2

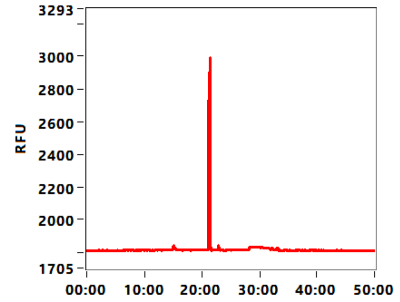

2019 06 18 14H 35M.raw
C3: 103613-001-115 (20x dil.)
GQN: 5.7

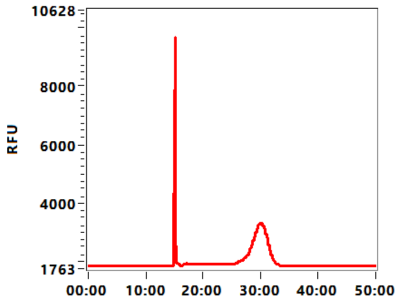

2019 06 18 14H 35M.raw
D3: 103613-001-116 (20x dil.)
GQN: 1.7

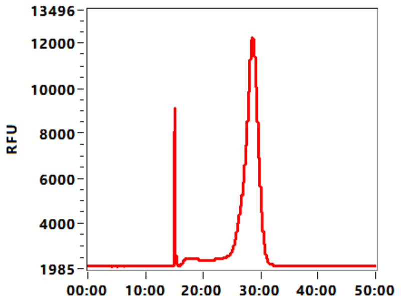

2019 06 18 14H 35M.raw
E3: 103613-001-117 (20x dil.)
GQN: 3.4

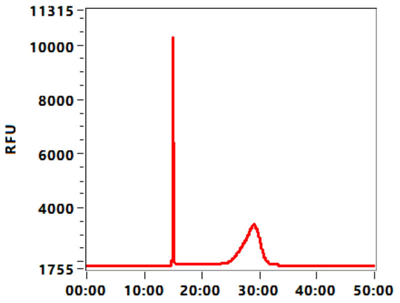

2019 06 18 14H 35M.raw
F3: 103613-001-118 (20x dil.)
GQN: 3.8

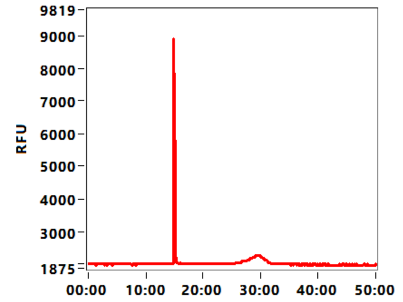

2019 06 18 14H 35M.raw
G3: 103613-001-119 (20x dil.)
GQN: 3.3

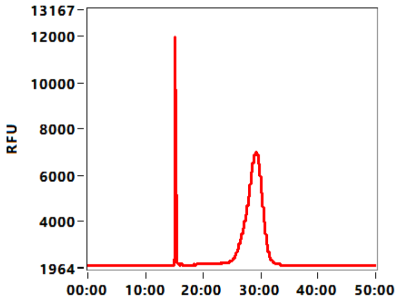

2019 06 18 14H 35M.raw
H3: 103613-001-120 (20x dil.)
GQN: 3.7

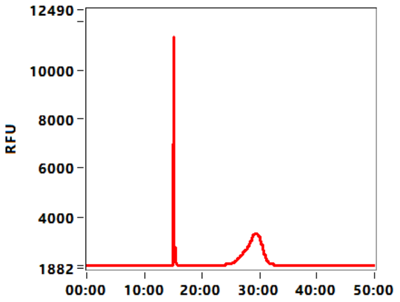

Copyright 2015 Advanced Analytical Technologies, Inc.

2019 06 18 14H 35M.raw
A4: 103613-001-121 (20x dil.)
GQN: 2.1

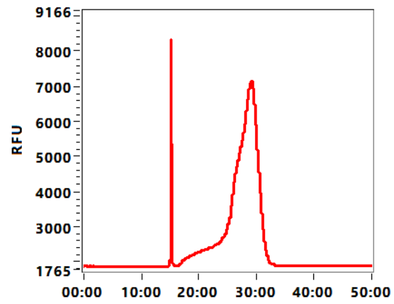

2019 06 18 14H 35M.raw
B4: 103613-001-122 (20x dil.)
GQN: 3.9

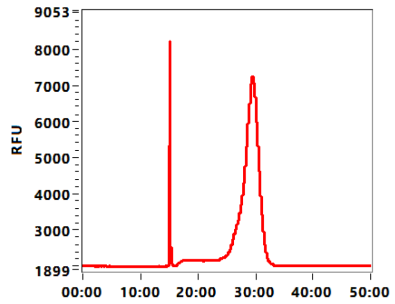

2019 06 18 14H 35M.raw
C4: 103613-001-123 (20x dil.)
GQN: 3.0

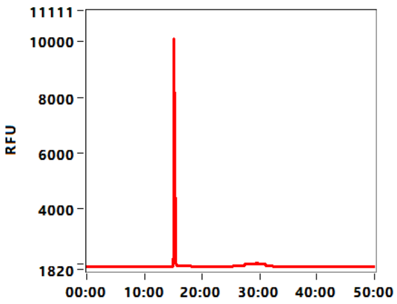

2019 06 18 14H 35M.raw
D4: 103613-001-124 (20x dil.)
GQN: 2.6

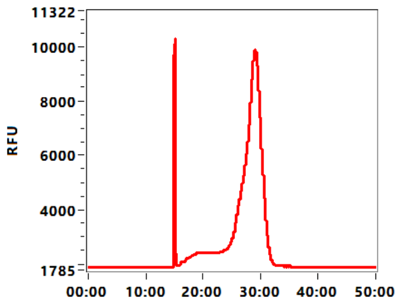

2019 06 18 14H 35M.raw
E4: 103613-001-125 (20x dil.)
GQN: 3.5

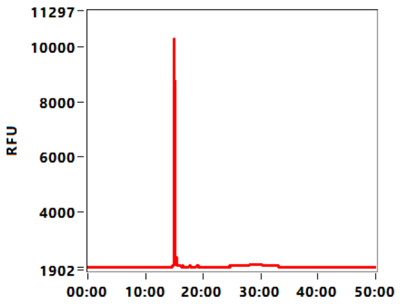

2019 06 18 14H 35M.raw
F4: 103613-001-126 (20x dil.)
GQN: 2.8

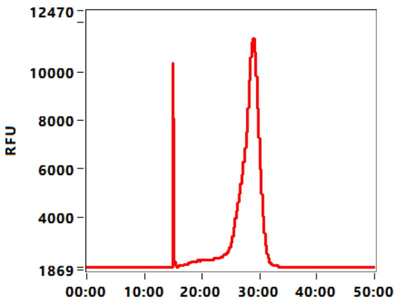

2019 06 18 14H 35M.raw
G4: 103613-001-127 (20x dil.)
GQN: 3.5

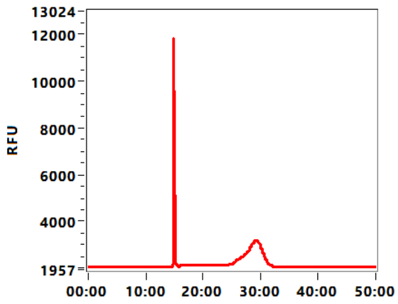

2019 06 18 14H 35M.raw
H4: 103613-001-128 (20x dil.)
GQN: 3.1

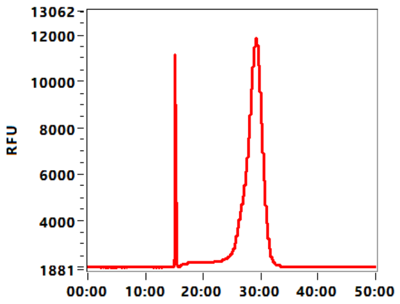

2019 06 18 14H 35M.raw
A5: 103613-001-129 (20x dil.)
GQN: 3.6

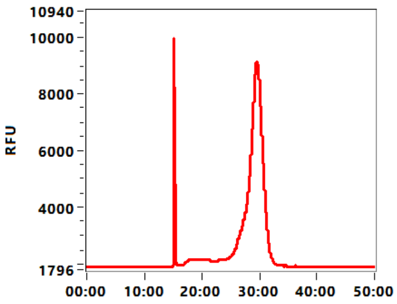

2019 06 18 14H 35M.raw
B5: 103613-001-130 (20x dil.)
GQN: 4.4

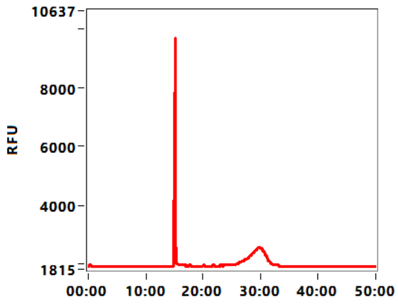

2019 06 18 14H 35M.raw
C5: 103613-001-131 (20x dil.)
GQN: 3.8

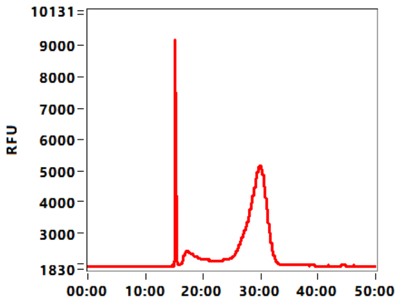

2019 06 18 14H 35M.raw
D5: 103613-001-132 (20x dil.)
GQN: 5.5

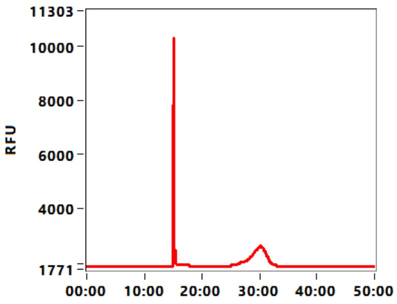

Copyright 2015 Advanced Analytical Technologies, Inc.

2019 06 18 14H 35M.raw
E5: 103613-001-133 (20x dil.)
GQN: 5.5

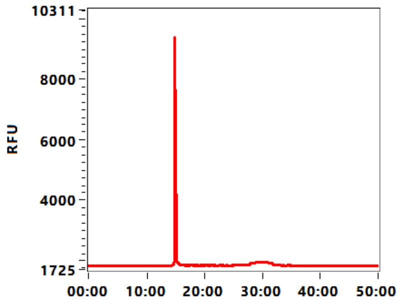

2019 06 18 14H 35M.raw
F5: 103613-001-134 (20x dil.)
GQN: 2.9

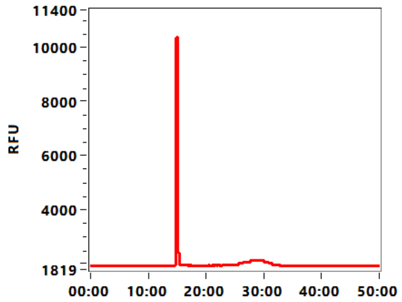

2019 06 18 14H 35M.raw
G5: 103613-001-135 (20x dil.)
GQN: 1.4

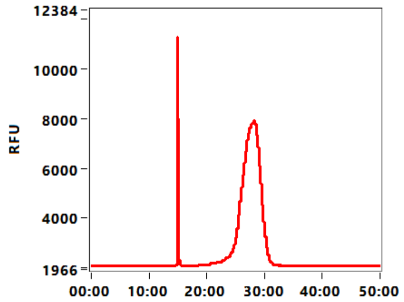

2019 06 18 14H 35M.raw
H5: 103613-001-136 (20x dil.)
GQN: 3.1

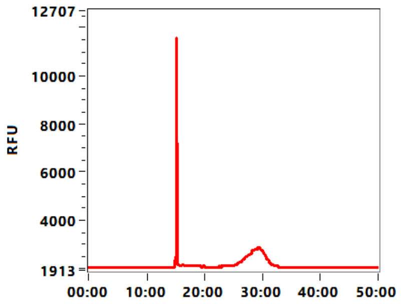

2019 06 18 14H 35M.raw
A6: 103613-001-137 (20x dil.)
GQN: 4.7

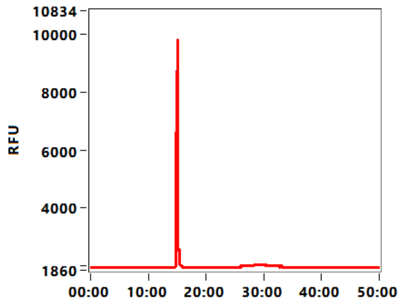

2019 06 18 14H 35M.raw
B6: 103613-001-138 (20x dil.)
GQN: 3.1

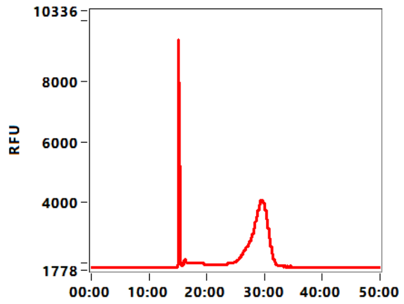

Copyright 2015 Advanced Analytical Technologies, Inc.

Data File: 2019 06 18 13H 16M.raw
Sample: 103613-001-001 (20x dil.)
Well Location: A1

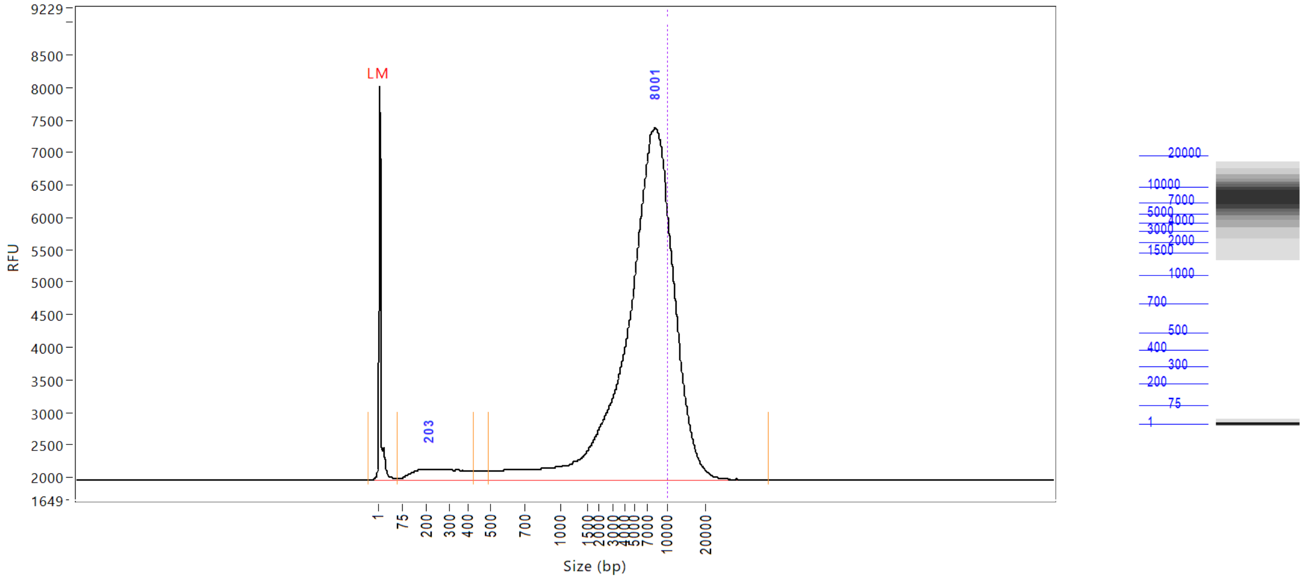

Peak	Size (bp)	Conc. (ng/uL)	From (bp)	To (bp)	Avg. Size (bp)	CV%	RFU	Corr. Peak Area
1	1 (LM)	0.0328	0	56	3	280.95	6056	42.247
2	203	0.2466	56	419	248	37.82	157	26.461
3	8001	5.0462	486	36480	7090	54.34	5427	541.573

TIC:	5.2927	ng/uL
TIM:	2.8068	nmole/L
Total Conc.:	5.3387	ng/uL
GQN:	1.9	

Sample Peak Width (sec): 50 Sample Min Peak Height: 50 Sample Baseline V to V?: Y Sample Baseline V to V pts: 3
Sample Filter: Binomial # of Pts for Filter: 3 Sample Start Region (min): 0 Sample End Region (min): 50
Manual Baseline Start (min): 6 Manual Baseline End (min): 48
Marker Peak Width (sec): 5 Marker Min Peak Height: 200 Marker Baseline V to V?: Y Marker Baseline V to V pts: 3
Lower Marker Selection: First Peak > 200 RFU Upper Marker Selection: Last Peak > 200 RFU
Ladder Size (bp): 1, 75, 200, 300, 400, 500, 700, 1000, 1500, 2000, 3000, 4000, 5000, 7000, 10000, 20000
Quantification Using: Ladder Final Concentration (ng/uL): 1.0417 Dilution Factor: 12.0
Size Threshold (b.p.): 10000

 Copyright 2015 Advanced Analytical Technologies, Inc.

Data File: 2019 06 18 13H 16M.raw
Sample: 103613-001-002 (20x dil.)
Well Location: B1

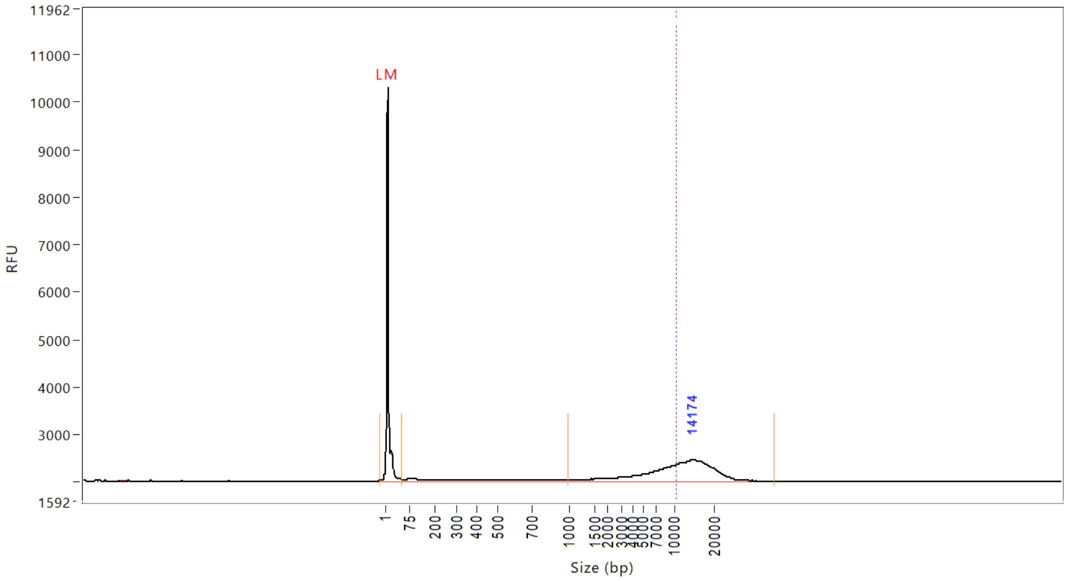

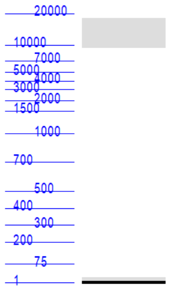

Peak	Size (bp)	Conc. (ng/uL)	From (bp)	To (bp)	Avg. Size (bp)	CV%	RFU	Corr. Peak Area
1	1 (LM)	0.0328	0	46	2	343.71	8281	57.356
2	14174	0.4549	997	36049	11681	53.01	440	66.280

TIC:	0.4549	ng/uL
TIM:	0.0641	nmole/L
Total Conc.:	0.5140	ng/uL
GQN:	5.0	

Sample Peak Width (sec): 50 Sample Min Peak Height: 50 Sample Baseline V to V?: Y Sample Baseline V to V pts: 3
Sample Filter: Binomial # of Pts for Filter: 3 Sample Start Region (min): 0 Sample End Region (min): 50
Manual Baseline Start (min): 6 Manual Baseline End (min): 48
Marker Peak Width (sec): 5 Marker Min Peak Height: 200 Marker Baseline V to V?: Y Marker Baseline V to V pts: 3
Lower Marker Selection: First Peak > 200 RFU Upper Marker Selection: Last Peak > 200 RFU
Ladder Size (bp): 1, 75, 200, 300, 400, 500, 700, 1000, 1500, 2000, 3000, 4000, 5000, 7000, 10000, 20000
Quantification Using: Ladder Final Concentration (ng/uL): 1.0417 Dilution Factor: 12.0
Size Threshold (b.p.): 10000

 Copyright 2015 Advanced Analytical Technologies, Inc.

Data File: 2019 06 18 13H 16M.raw
Sample: 103613-001-003 (20x dil.)
Well Location: C1

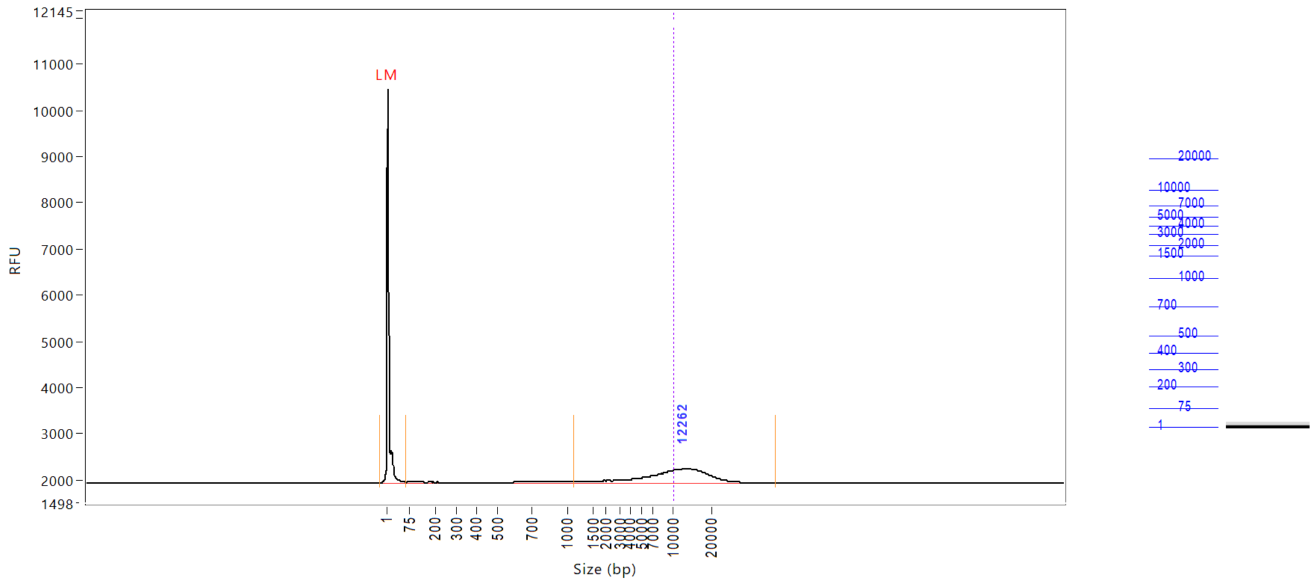

Peak	Size (bp)	Conc. (ng/uL)	From (bp)	To (bp)	Avg. Size (bp)	CV%	RFU	Corr. Peak Area
1	1 (LM)	0.0328	0	59	3	256.52	8511	60.187
2	12262	0.2970	1107	37084	11211	54.12	302	45.409

TIC:	0.2970	ng/uL
TIM:	0.0436	nmole/L
Total Conc.:	0.3388	ng/uL
GQN:	4.6	

Sample Peak Width (sec): 50 Sample Min Peak Height: 50 Sample Baseline V to V?: Y Sample Baseline V to V pts: 3
Sample Filter: Binomial # of Pts for Filter: 3 Sample Start Region (min): 0 Sample End Region (min): 50
Manual Baseline Start (min): 6 Manual Baseline End (min): 48
Marker Peak Width (sec): 5 Marker Min Peak Height: 200 Marker Baseline V to V?: Y Marker Baseline V to V pts: 3
Lower Marker Selection: First Peak > 200 RFU Upper Marker Selection: Last Peak > 200 RFU
Ladder Size (bp): 1, 75, 200, 300, 400, 500, 700, 1000, 1500, 2000, 3000, 4000, 5000, 7000, 10000, 20000
Quantification Using: Ladder Final Concentration (ng/uL): 1.0417 Dilution Factor: 12.0
Size Threshold (b.p.): 10000

 Copyright 2015 Advanced Analytical Technologies, Inc.

Data File: 2019 06 18 13H 16M.raw
Sample: 103613-001-004 (20x dil.)
Well Location: D1

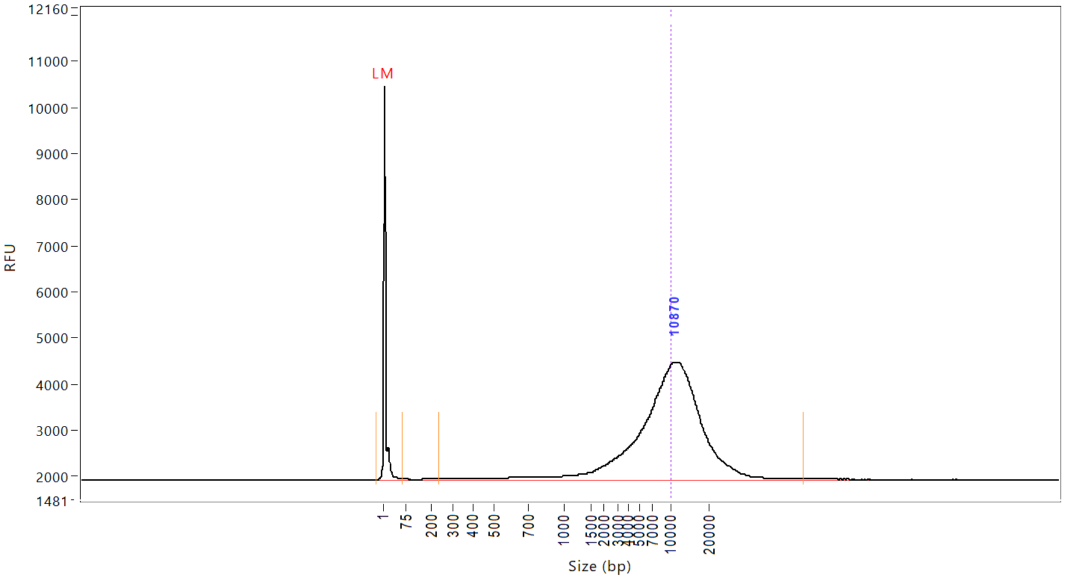

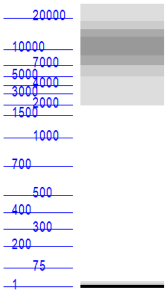

Peak	Size (bp)	Conc. (ng/uL)	From (bp)	To (bp)	Avg. Size (bp)	CV%	RFU	Corr. Peak Area
1	1 (LM)	0.0328	0	59	3	259.85	8534	60.185
2	10870	2.3670	231	44419	10369	60.76	2547	361.906

TIC:	2.3670	ng/uL
TIM:	0.3758	nmole/L
Total Conc.:	2.3934	ng/uL
GQN:	4.7	

Sample Peak Width (sec): 50 Sample Min Peak Height: 50 Sample Baseline V to V?: Y Sample Baseline V to V pts: 3
Sample Filter: Binomial # of Pts for Filter: 3 Sample Start Region (min): 0 Sample End Region (min): 50
Manual Baseline Start (min): 6 Manual Baseline End (min): 48
Marker Peak Width (sec): 5 Marker Min Peak Height: 200 Marker Baseline V to V?: Y Marker Baseline V to V pts: 3
Lower Marker Selection: First Peak > 200 RFU Upper Marker Selection: Last Peak > 200 RFU
Ladder Size (bp): 1, 75, 200, 300, 400, 500, 700, 1000, 1500, 2000, 3000, 4000, 5000, 7000, 10000, 20000
Quantification Using: Ladder Final Concentration (ng/uL): 1.0417 Dilution Factor: 12.0
Size Threshold (b.p.): 10000

Copyright 2015 Advanced Analytical Technologies, Inc.

Data File: 2019 06 18 13H 16M.raw
Sample: 103613-001-005 (20x dil.)
Well Location: E1

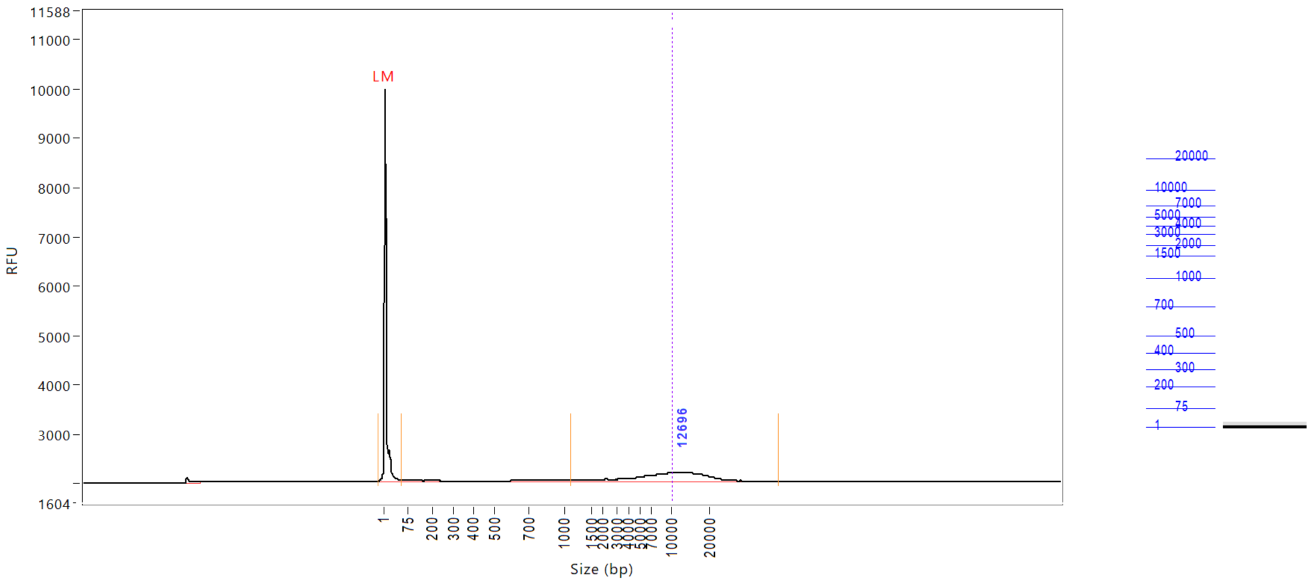

Peak	Size (bp)	Conc. (ng/uL)	From (bp)	To (bp)	Avg. Size (bp)	CV%	RFU	Corr. Peak Area
1	1 (LM)	0.0328	0	54	2	365.43	7947	55.740
2	12696	0.2140	1088	38034	10697	59.39	178	30.302

TIC:	0.2140	ng/uL
TIM:	0.0329	nmole/L
Total Conc.:	0.2547	ng/uL
GQN:	4.1	

Sample Peak Width (sec): 50 Sample Min Peak Height: 50 Sample Baseline V to V?: Y Sample Baseline V to V pts: 3
Sample Filter: Binomial # of Pts for Filter: 3 Sample Start Region (min): 0 Sample End Region (min): 50
Manual Baseline Start (min): 6 Manual Baseline End (min): 48
Marker Peak Width (sec): 5 Marker Min Peak Height: 200 Marker Baseline V to V?: Y Marker Baseline V to V pts: 3
Lower Marker Selection: First Peak > 200 RFU Upper Marker Selection: Last Peak > 200 RFU
Ladder Size (bp): 1, 75, 200, 300, 400, 500, 700, 1000, 1500, 2000, 3000, 4000, 5000, 7000, 10000, 20000
Quantification Using: Ladder Final Concentration (ng/uL): 1.0417 Dilution Factor: 12.0
Size Threshold (b.p.): 10000

 Copyright 2015 Advanced Analytical Technologies, Inc.

Data File: 2019 06 18 13H 16M.raw
Sample: 103613-001-006 (20x dil.)
Well Location: F1

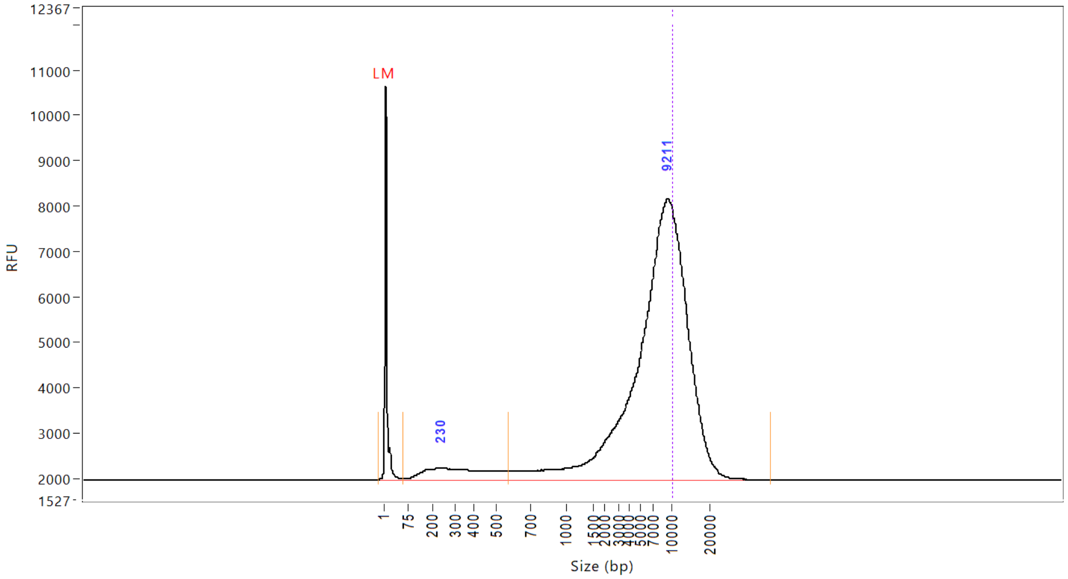

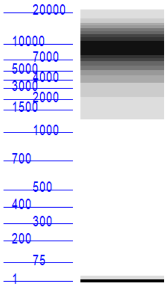

Peak	Size (bp)	Conc. (ng/uL)	From (bp)	To (bp)	Avg. Size (bp)	CV%	RFU	Corr. Peak Area
1	1 (LM)	0.0328	0	57	2	334.49	8665	60.067
2	230	0.3396	57	567	311	41.82	241	51.817
3	9211	4.4060	567	35704	8173	55.01	6185	672.339

TIC:	4.7456	ng/uL
TIM:	2.6836	nmole/L
Total Conc.:	4.7625	ng/uL
GQN:	2.9	

Sample Peak Width (sec): 50 Sample Min Peak Height: 50 Sample Baseline V to V?: Y Sample Baseline V to V pts: 3
Sample Filter: Binomial # of Pts for Filter: 3 Sample Start Region (min): 0 Sample End Region (min): 50
Manual Baseline Start (min): 6 Manual Baseline End (min): 48
Marker Peak Width (sec): 5 Marker Min Peak Height: 200 Marker Baseline V to V?: Y Marker Baseline V to V pts: 3
Lower Marker Selection: First Peak > 200 RFU Upper Marker Selection: Last Peak > 200 RFU
Ladder Size (bp): 1, 75, 200, 300, 400, 500, 700, 1000, 1500, 2000, 3000, 4000, 5000, 7000, 10000, 20000
Quantification Using: Ladder Final Concentration (ng/uL): 1.0417 Dilution Factor: 12.0
Size Threshold (b.p.): 10000

 Copyright 2015 Advanced Analytical Technologies, Inc.

Data File: 2019 06 18 13H 16M.raw
Sample: 103613-001-007 (20x dil.)
Well Location: G1

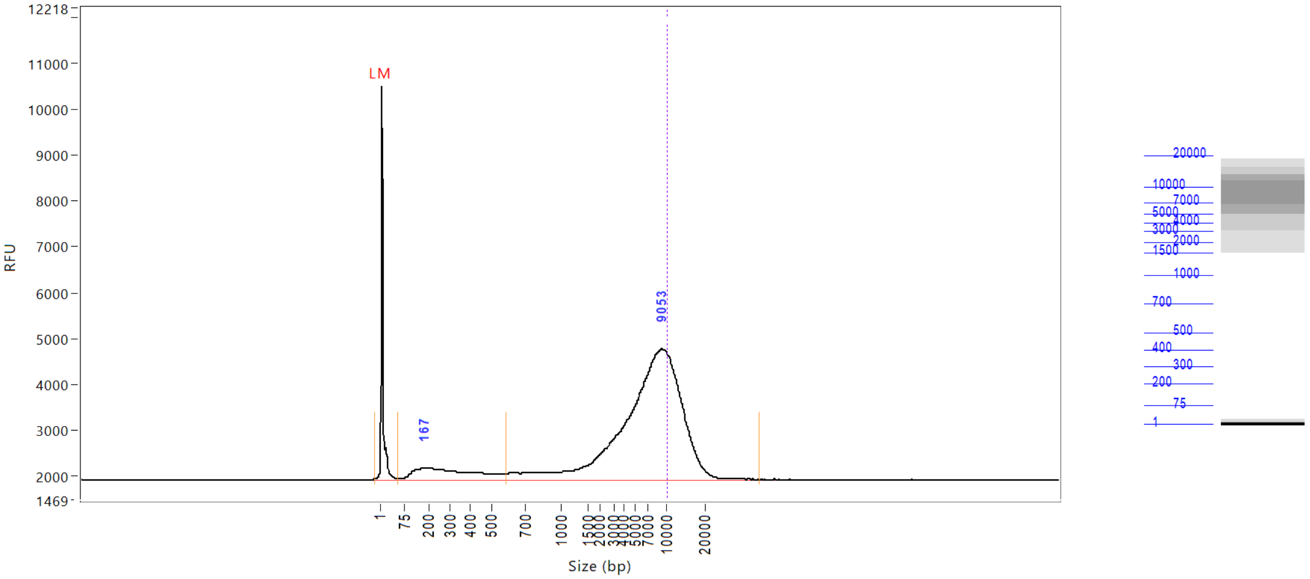

Peak	Size (bp)	Conc. (ng/uL)	From (bp)	To (bp)	Avg. Size (bp)	CV%	RFU	Corr. Peak Area
1	1 (LM)	0.0328	0	55	2	294.31	8583	59.634
2	167	0.3037	55	585	290	47.05	252	46.010
3	9053	2.3135	585	34582	7645	59.79	2849	350.487

TIC:	2.6172	ng/uL
TIM:	2.2208	nmole/L
Total Conc.:	2.6384	ng/uL
GQN:	2.4	

Sample Peak Width (sec): 50   Sample Min Peak Height: 50   Sample Baseline V to V?: Y   Sample Baseline V to V pts: 3
Sample Filter: Binomial   # of Pts for Filter: 3   Sample Start Region (min): 0   Sample End Region (min): 50
Manual Baseline Start (min): 6   Manual Baseline End (min): 48
Marker Peak Width (sec): 5   Marker Min Peak Height: 200   Marker Baseline V to V?: Y   Marker Baseline V to V pts: 3
Lower Marker Selection: First Peak > 200 RFU   Upper Marker Selection: Last Peak > 200 RFU
Ladder Size (bp): 1, 75, 200, 300, 400, 500, 700, 1000, 1500, 2000, 3000, 4000, 5000, 7000, 10000, 20000
Quantification Using: Ladder   Final Concentration (ng/uL): 1.0417   Dilution Factor: 12.0
Size Threshold (b.p.): 10000

Copyright 2015 Advanced Analytical Technologies, Inc.

Data File: 2019 06 18 13H 16M.raw
Sample: 103613-001-008 (20x dil.)
Well Location: H1

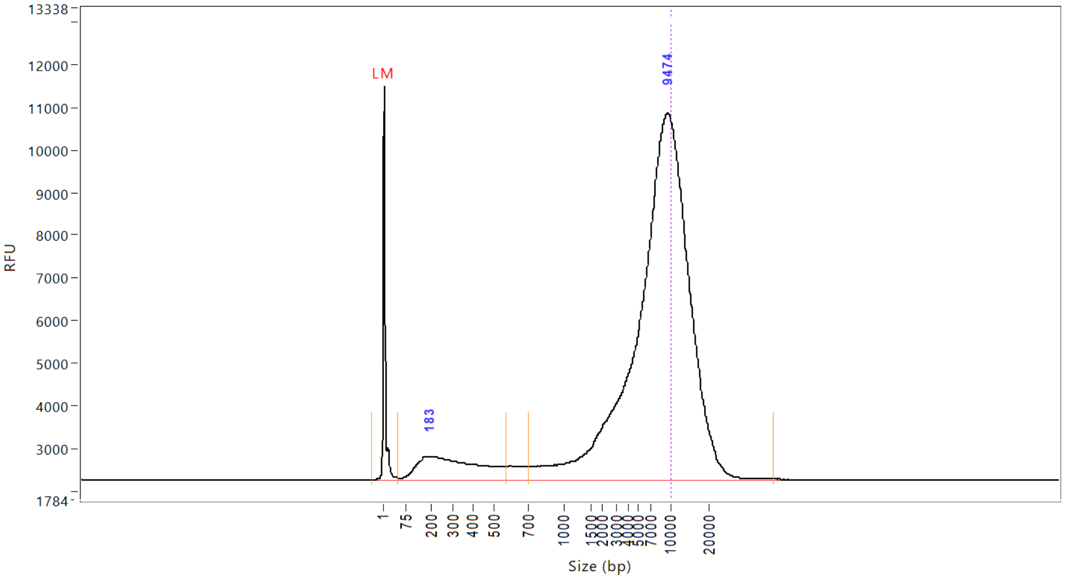

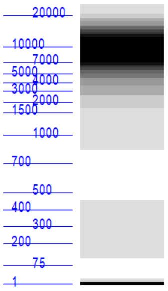

Peak	Size (bp)	Conc. (ng/uL)	From (bp)	To (bp)	Avg. Size (bp)	CV%	RFU	Corr. Peak Area
1	1 (LM)	0.0328	0	45	2	298.31	9229	64.057
2	183	0.6409	45	569	294	44.68	535	104.289
3	9474	6.0779	700	36912	8699	55.71	8601	989.062

TIC:	6.7187	ng/uL
TIM:	4.7358	nmole/L
Total Conc.:	6.8325	ng/uL
GQN:	3.1	

Sample Peak Width (sec): 50 Sample Min Peak Height: 50 Sample Baseline V to V?: Y Sample Baseline V to V pts: 3
Sample Filter: Binomial # of Pts for Filter: 3 Sample Start Region (min): 0 Sample End Region (min): 50
Manual Baseline Start (min): 6 Manual Baseline End (min): 48
Marker Peak Width (sec): 5 Marker Min Peak Height: 200 Marker Baseline V to V?: Y Marker Baseline V to V pts: 3
Lower Marker Selection: First Peak > 200 RFU Upper Marker Selection: Last Peak > 200 RFU
Ladder Size (bp): 1, 75, 200, 300, 400, 500, 700, 1000, 1500, 2000, 3000, 4000, 5000, 7000, 10000, 20000
Quantification Using: Ladder Final Concentration (ng/uL): 1.0417 Dilution Factor: 12.0
Size Threshold (b.p.): 10000

 Copyright 2015 Advanced Analytical Technologies, Inc.

Data File: 2019 06 18 13H 16M.raw
Sample: 103613-001-009 (20x dil.)
Well Location: A2

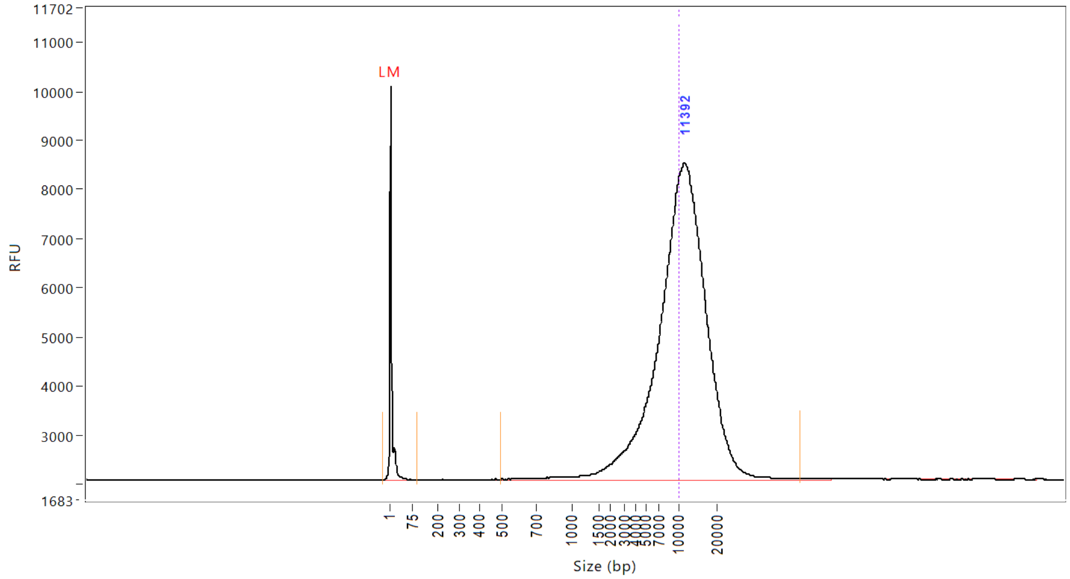

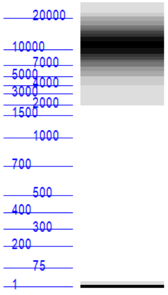

Peak	Size (bp)	Conc. (ng/uL)	From (bp)	To (bp)	Avg. Size (bp)	CV%	RFU	Corr. Peak Area
1	1 (LM)	0.0328	0	89	2	369.68	8004	55.378
2	11392	5.1074	497	41658	11116	46.31	6436	718.529

TIC:	5.1074	ng/uL
TIM:	0.7564	nmole/L
Total Conc.:	5.1300	ng/uL
GQN:	5.5	

Sample Peak Width (sec): 50 Sample Min Peak Height: 50 Sample Baseline V to V?: Y Sample Baseline V to V pts: 3
Sample Filter: Binomial # of Pts for Filter: 3 Sample Start Region (min): 0 Sample End Region (min): 50
Manual Baseline Start (min): 6 Manual Baseline End (min): 48
Marker Peak Width (sec): 5 Marker Min Peak Height: 200 Marker Baseline V to V?: Y Marker Baseline V to V pts: 3
Lower Marker Selection: First Peak > 200 RFU Upper Marker Selection: Last Peak > 200 RFU
Ladder Size (bp): 1, 75, 200, 300, 400, 500, 700, 1000, 1500, 2000, 3000, 4000, 5000, 7000, 10000, 20000
Quantification Using: Ladder Final Concentration (ng/uL): 1.0417 Dilution Factor: 12.0
Size Threshold (b.p.): 10000

 Copyright 2015 Advanced Analytical Technologies, Inc.

Data File: 2019 06 18 13H 16M.raw
Sample: 103613-001-010 (20x dil.)
Well Location: B2

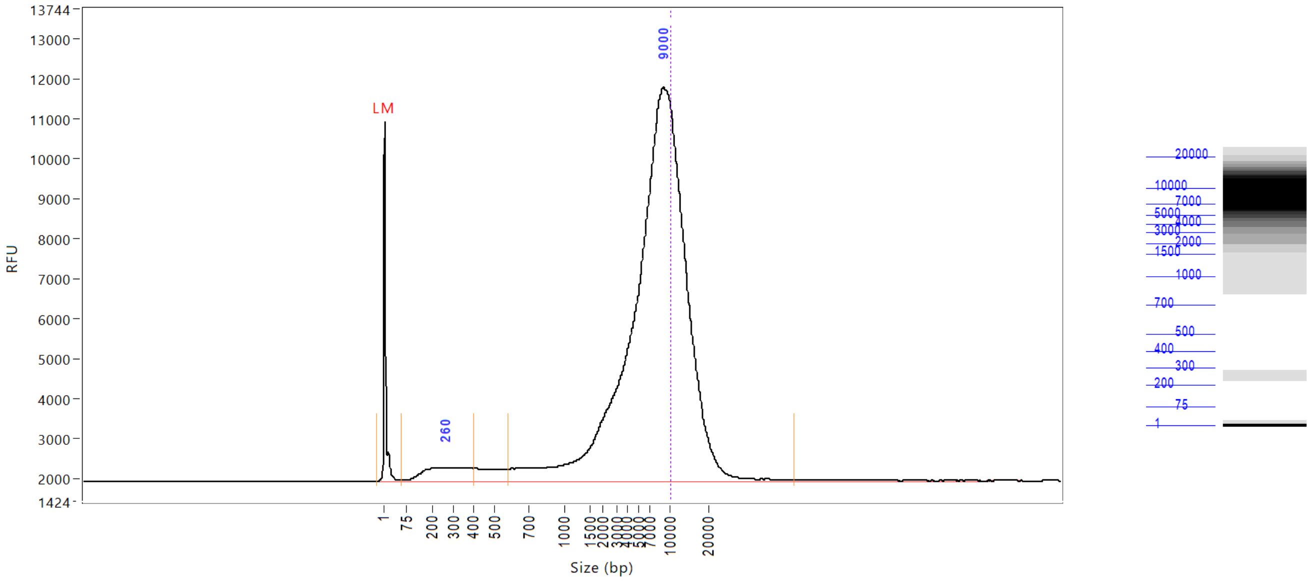

Peak	Size (bp)	Conc. (ng/uL)	From (bp)	To (bp)	Avg. Size (bp)	CV%	RFU	Corr. Peak Area
1	1 (LM)	0.0328	0	54	2	312.88	9012	62.003
2	260	0.3183	54	404	255	33.25	327	50.131
3	9000	7.2481	575	42520	8251	58.88	9839	1141.670

TIC:	7.5664	ng/uL
TIM:	3.4990	nmole/L
Total Conc.:	7.7485	ng/uL
GQN:	2.9	

```
Sample Peak Width (sec): 50   Sample Min Peak Height: 50    Sample Baseline V to V?: Y    Sample Baseline V to V pts: 3
Sample Filter: Binomial       # of Pts for Filter: 3        Sample Start Region (min): 0  Sample End Region (min): 50
Manual Baseline Start (min): 6     Manual Baseline End (min): 48
Marker Peak Width (sec): 5    Marker Min Peak Height: 200   Marker Baseline V to V?: Y    Marker Baseline V to V pts: 3
Lower Marker Selection: First Peak > 200 RFU                Upper Marker Selection: Last Peak > 200 RFU
Ladder Size (bp): 1, 75, 200, 300, 400, 500, 700, 1000, 1500, 2000, 3000, 4000, 5000, 7000, 10000, 20000
Quantification Using: Ladder          Final Concentration (ng/uL): 1.0417          Dilution Factor: 12.0
Size Threshold (b.p.): 10000
```

 Copyright 2015 Advanced Analytical Technologies, Inc.

Data File: 2019 06 18 13H 16M.raw
Sample: 103613-001-011 (20x dil.)
Well Location: C2

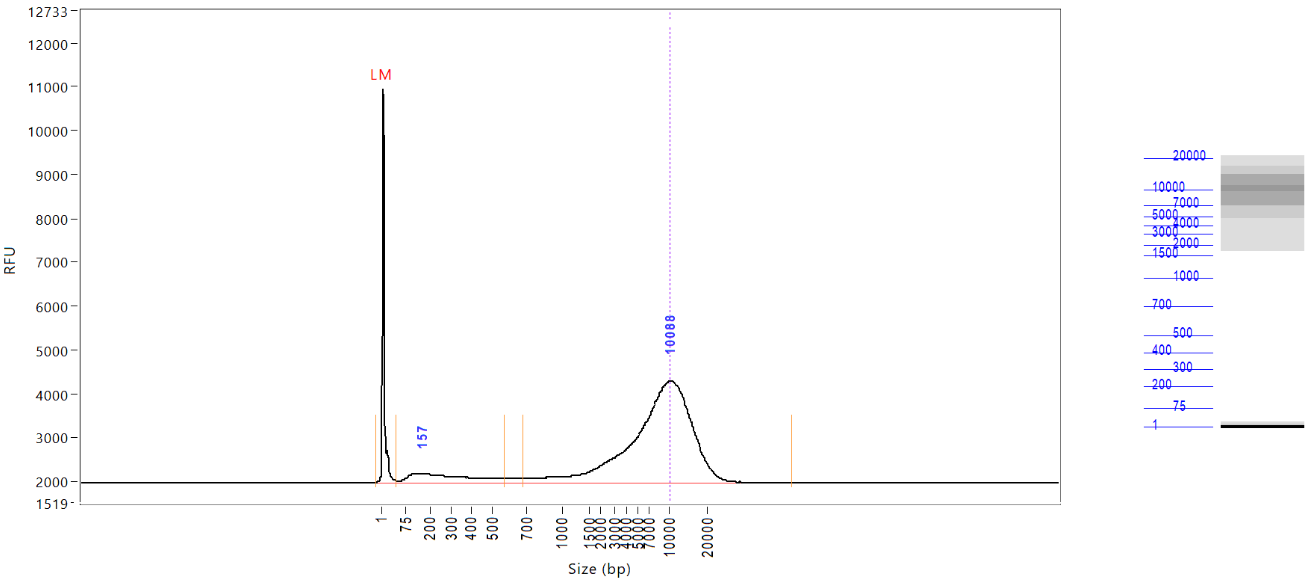

Peak	Size (bp)	Conc. (ng/uL)	From (bp)	To (bp)	Avg. Size (bp)	CV%	RFU	Corr. Peak Area
1	1 (LM)	0.0328	0	46	3	251.14	8963	63.299
2	157	0.2373	46	563	262	52.74	203	38.159
3	10088	1.8867	673	42089	8872	58.61	2315	303.398

TIC:	2.1240	ng/uL
TIM:	1.8398	nmole/L
Total Conc.:	2.1521	ng/uL
GQN:	3.3	

Sample Peak Width (sec): 50 Sample Min Peak Height: 50 Sample Baseline V to V?: Y Sample Baseline V to V pts: 3
Sample Filter: Binomial # of Pts for Filter: 3 Sample Start Region (min): 0 Sample End Region (min): 50
Manual Baseline Start (min): 6 Manual Baseline End (min): 48
Marker Peak Width (sec): 5 Marker Min Peak Height: 200 Marker Baseline V to V?: Y Marker Baseline V to V pts: 3
Lower Marker Selection: First Peak > 200 RFU Upper Marker Selection: Last Peak > 200 RFU
Ladder Size (bp): 1, 75, 200, 300, 400, 500, 700, 1000, 1500, 2000, 3000, 4000, 5000, 7000, 10000, 20000
Quantification Using: Ladder Final Concentration (ng/uL): 1.0417 Dilution Factor: 12.0
Size Threshold (b.p.): 10000

 Copyright 2015 Advanced Analytical Technologies, Inc.

Data File: 2019 06 18 13H 16M.raw
Sample: 103613-001-012 (20x dil.)
Well Location: D2

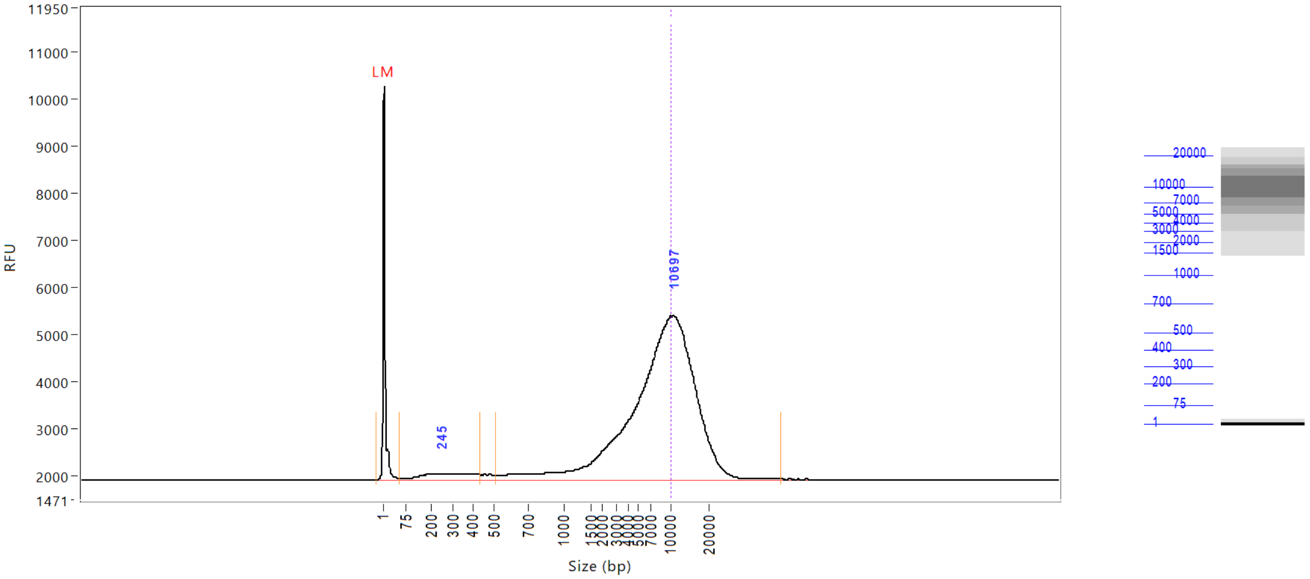

Peak	Size (bp)	Conc. (ng/uL)	From (bp)	To (bp)	Avg. Size (bp)	CV%	RFU	Corr. Peak Area
1	1 (LM)	0.0328	0	52	2	352.40	8377	58.911
2	245	0.1447	52	433	256	39.16	126	21.652
3	10697	3.2657	509	38983	9007	61.02	3497	488.745

TIC:	3.4104	ng/uL
TIM:	1.5264	nmole/L
Total Conc.:	3.4582	ng/uL
GQN:	3.7	

Sample Peak Width (sec): 50   Sample Min Peak Height: 50   Sample Baseline V to V?: Y   Sample Baseline V to V pts: 3
Sample Filter: Binomial   # of Pts for Filter: 3   Sample Start Region (min): 0   Sample End Region (min): 50
Manual Baseline Start (min): 6   Manual Baseline End (min): 48
Marker Peak Width (sec): 5   Marker Min Peak Height: 200   Marker Baseline V to V?: Y   Marker Baseline V to V pts: 3
Lower Marker Selection: First Peak > 200 RFU   Upper Marker Selection: Last Peak > 200 RFU
Ladder Size (bp): 1, 75, 200, 300, 400, 500, 700, 1000, 1500, 2000, 3000, 4000, 5000, 7000, 10000, 20000
Quantification Using: Ladder   Final Concentration (ng/uL): 1.0417   Dilution Factor: 12.0
Size Threshold (b.p.): 10000

 Copyright 2015 Advanced Analytical Technologies, Inc.

Data File: 2019 06 18 13H 16M.raw
Sample: 103613-001-013 (20x dil.)
Well Location: E2

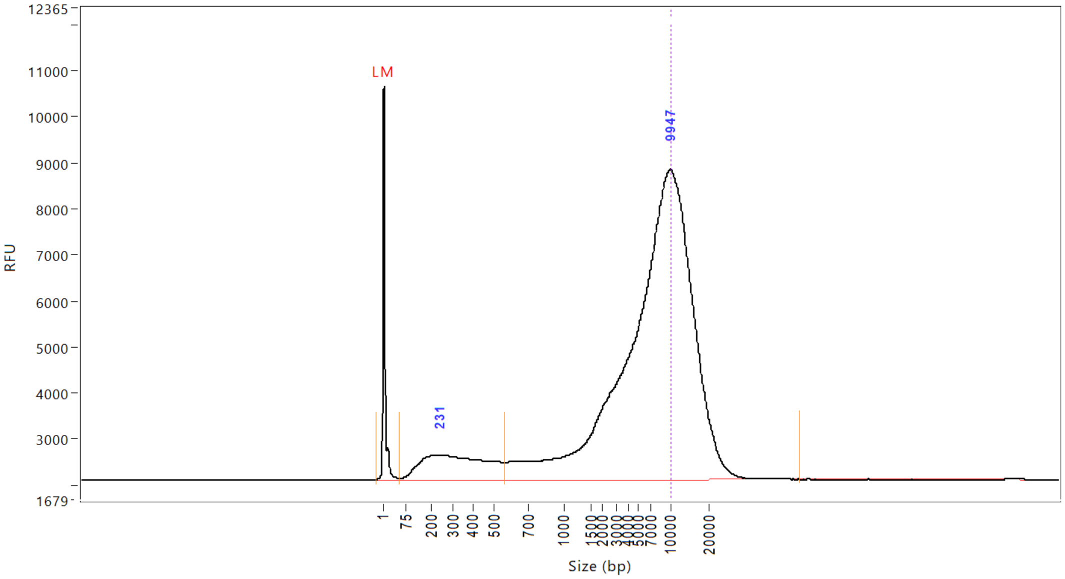

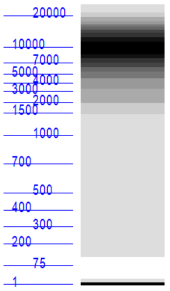

Peak	Size (bp)	Conc. (ng/uL)	From (bp)	To (bp)	Avg. Size (bp)	CV%	RFU	Corr. Peak Area
1	1 (LM)	0.0328	0	52	2	391.41	8535	60.521
2	231	0.7196	52	554	304	41.66	525	110.632
3	9947	6.2181	554	43556	8265	64.70	6748	956.017

TIC:	6.9376	ng/uL
TIM:	5.1322	nmole/L
Total Conc.:	6.9385	ng/uL
GQN:	3.1	

```
Sample Peak Width (sec): 50   Sample Min Peak Height: 50    Sample Baseline V to V?: Y    Sample Baseline V to V pts: 3
Sample Filter: Binomial       # of Pts for Filter: 3        Sample Start Region (min): 0  Sample End Region (min): 50
Manual Baseline Start (min): 6     Manual Baseline End (min): 48
Marker Peak Width (sec): 5    Marker Min Peak Height: 200   Marker Baseline V to V?: Y    Marker Baseline V to V pts: 3
Lower Marker Selection: First Peak > 200 RFU                Upper Marker Selection: Last Peak > 200 RFU
Ladder Size (bp): 1, 75, 200, 300, 400, 500, 700, 1000, 1500, 2000, 3000, 4000, 5000, 7000, 10000, 20000
Quantification Using: Ladder          Final Concentration (ng/uL): 1.0417          Dilution Factor: 12.0
Size Threshold (b.p.): 10000
```


Copyright 2015 Advanced Analytical Technologies, Inc.

Data File: 2019 06 18 13H 16M.raw
Sample: 103613-001-014 (20x dil.)
Well Location: F2

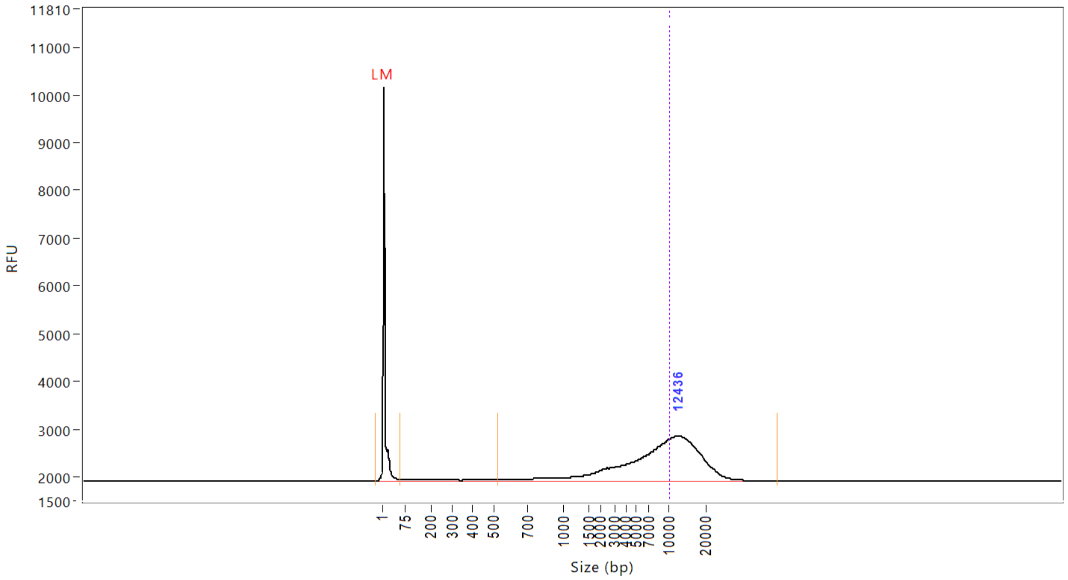

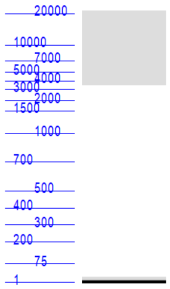

Peak	Size (bp)	Conc. (ng/uL)	From (bp)	To (bp)	Avg. Size (bp)	CV%	RFU	Corr. Peak Area
1	1 (LM)	0.0328	0	56	3	262.53	8240	57.864
2	12436	1.0381	524	38810	9648	63.95	927	152.600

TIC:	1.0381	ng/uL
TIM:	0.1771	nmole/L
Total Conc.:	1.0775	ng/uL
GQN:	4.3	

```
Sample Peak Width (sec): 50   Sample Min Peak Height: 50    Sample Baseline V to V?: Y    Sample Baseline V to V pts: 3
Sample Filter: Binomial       # of Pts for Filter: 3        Sample Start Region (min): 0  Sample End Region (min): 50
Manual Baseline Start (min): 6      Manual Baseline End (min): 48
Marker Peak Width (sec): 5    Marker Min Peak Height: 200   Marker Baseline V to V?: Y    Marker Baseline V to V pts: 3
Lower Marker Selection: First Peak > 200 RFU                Upper Marker Selection: Last Peak > 200 RFU
Ladder Size (bp): 1, 75, 200, 300, 400, 500, 700, 1000, 1500, 2000, 3000, 4000, 5000, 7000, 10000, 20000
Quantification Using: Ladder          Final Concentration (ng/uL): 1.0417          Dilution Factor: 12.0
Size Threshold (b.p.): 10000
```

 Copyright 2015 Advanced Analytical Technologies, Inc.

Data File: 2019 06 18 13H 16M.raw
Sample: 103613-001-015 (20x dil.)
Well Location: G2

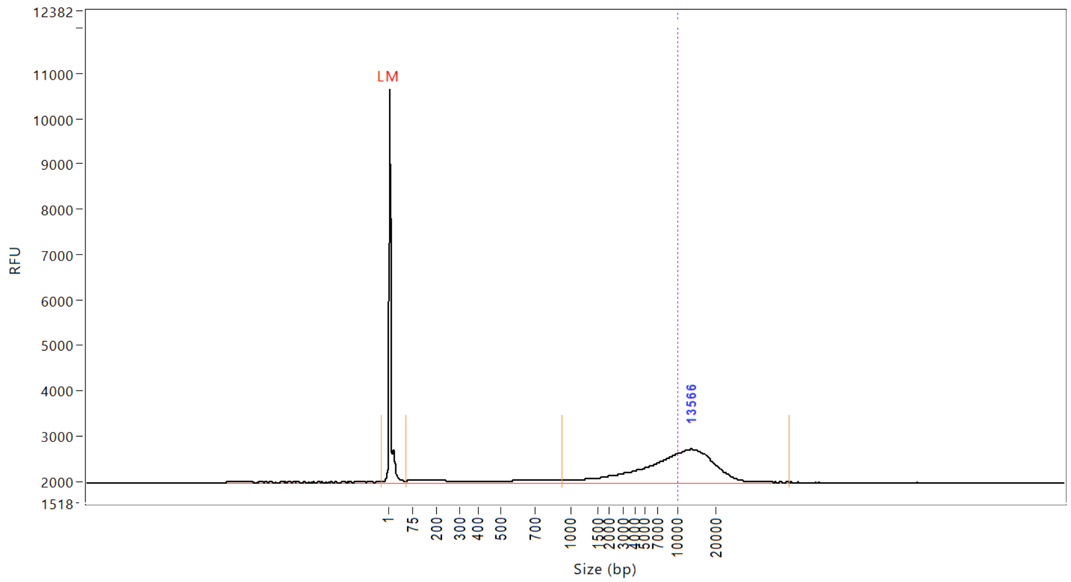

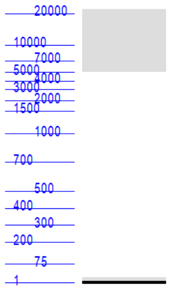

Peak	Size (bp)	Conc. (ng/uL)	From (bp)	To (bp)	Avg. Size (bp)	CV%	RFU	Corr. Peak Area
1	1 (LM)	0.0328	0	51	2	371.73	8680	62.575
2	13566	0.7907	927	39155	10868	61.21	740	125.697

TIC:	0.7907	ng/uL
TIM:	0.1198	nmole/L
Total Conc.:	0.9416	ng/uL
GQN:	4.4	

Sample Peak Width (sec): 50 Sample Min Peak Height: 50 Sample Baseline V to V?: Y Sample Baseline V to V pts: 3
Sample Filter: Binomial # of Pts for Filter: 3 Sample Start Region (min): 0 Sample End Region (min): 50
Manual Baseline Start (min): 6 Manual Baseline End (min): 48
Marker Peak Width (sec): 5 Marker Min Peak Height: 200 Marker Baseline V to V?: Y Marker Baseline V to V pts: 3
Lower Marker Selection: First Peak > 200 RFU Upper Marker Selection: Last Peak > 200 RFU
Ladder Size (bp): 1, 75, 200, 300, 400, 500, 700, 1000, 1500, 2000, 3000, 4000, 5000, 7000, 10000, 20000
Quantification Using: Ladder Final Concentration (ng/uL): 1.0417 Dilution Factor: 12.0
Size Threshold (b.p.): 10000

 Copyright 2015 Advanced Analytical Technologies, Inc.

Data File: 2019 06 18 13H 16M.raw
Sample: 103613-001-016 (20x dil.)
Well Location: H2

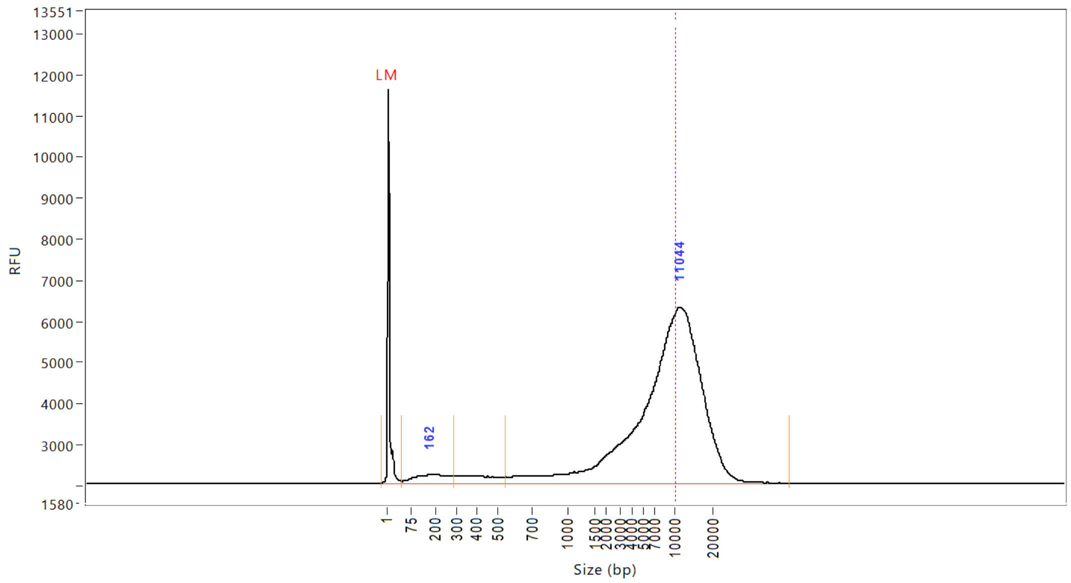

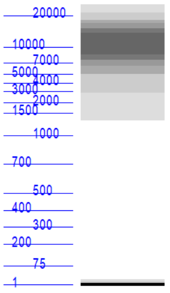

Peak	Size (bp)	Conc. (ng/uL)	From (bp)	To (bp)	Avg. Size (bp)	CV%	RFU	Corr. Peak Area
1	1 (LM)	0.0328	0	45	2	311.72	9570	66.534
2	162	0.1467	45	283	170	39.10	200	24.792
3	11044	3.4822	538	40191	9459	59.90	4274	588.573

TIC:	3.6289	ng/uL
TIM:	2.0244	nmole/L
Total Conc.:	3.7630	ng/uL
GQN:	4.0	

Sample Peak Width (sec): 50 Sample Min Peak Height: 50 Sample Baseline V to V?: Y Sample Baseline V to V pts: 3
Sample Filter: Binomial # of Pts for Filter: 3 Sample Start Region (min): 0 Sample End Region (min): 50
Manual Baseline Start (min): 6 Manual Baseline End (min): 48
Marker Peak Width (sec): 5 Marker Min Peak Height: 200 Marker Baseline V to V?: Y Marker Baseline V to V pts: 3
Lower Marker Selection: First Peak > 200 RFU Upper Marker Selection: Last Peak > 200 RFU
Ladder Size (bp): 1, 75, 200, 300, 400, 500, 700, 1000, 1500, 2000, 3000, 4000, 5000, 7000, 10000, 20000
Quantification Using: Ladder Final Concentration (ng/uL): 1.0417 Dilution Factor: 12.0
Size Threshold (b.p.): 10000

 Copyright 2015 Advanced Analytical Technologies, Inc.

Data File: 2019 06 18 13H 16M.raw
Sample: 103613-001-017 (20x dil.)
Well Location: A3

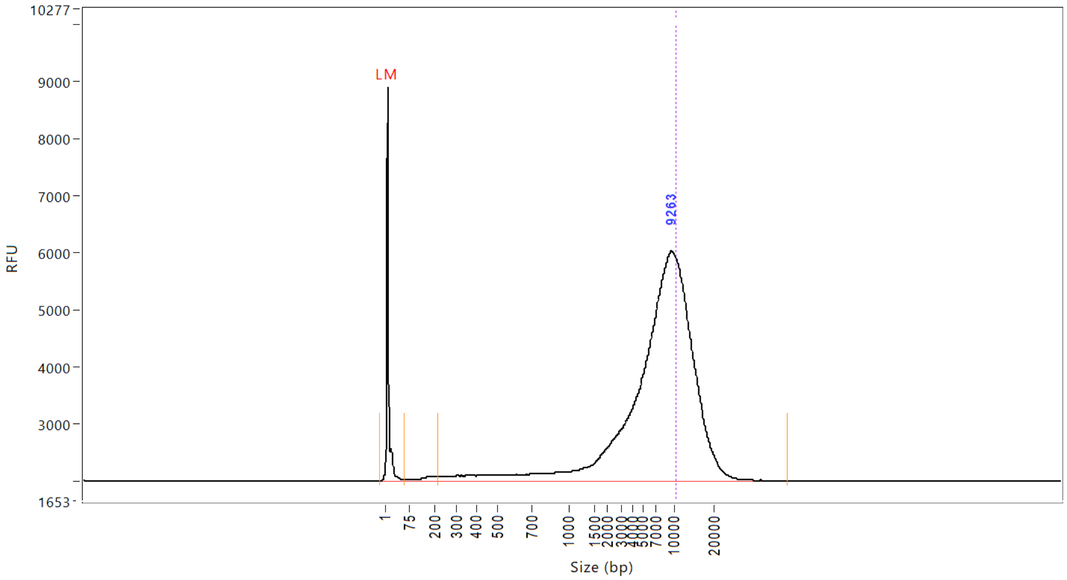

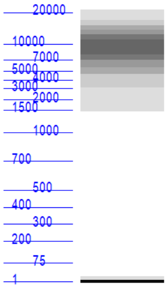

Peak	Size (bp)	Conc. (ng/uL)	From (bp)	To (bp)	Avg. Size (bp)	CV%	RFU	Corr. Peak Area
1	1 (LM)	0.0328	0	54	3	263.93	6891	48.261
2	9263	3.9261	209	39155	8146	60.07	4018	481.349

TIC:	3.9261	ng/uL
TIM:	0.7935	nmole/L
Total Conc.:	3.9684	ng/uL
GQN:	3.2	

Sample Peak Width (sec): 50   Sample Min Peak Height: 50   Sample Baseline V to V?: Y   Sample Baseline V to V pts: 3
Sample Filter: Binomial   # of Pts for Filter: 3   Sample Start Region (min): 0   Sample End Region (min): 50
Manual Baseline Start (min): 6   Manual Baseline End (min): 48
Marker Peak Width (sec): 5   Marker Min Peak Height: 200   Marker Baseline V to V?: Y   Marker Baseline V to V pts: 3
Lower Marker Selection: First Peak > 200 RFU   Upper Marker Selection: Last Peak > 200 RFU
Ladder Size (bp): 1, 75, 200, 300, 400, 500, 700, 1000, 1500, 2000, 3000, 4000, 5000, 7000, 10000, 20000
Quantification Using: Ladder   Final Concentration (ng/uL): 1.0417   Dilution Factor: 12.0
Size Threshold (b.p.): 10000

 Copyright 2015 Advanced Analytical Technologies, Inc.

Data File: 2019 06 18 13H 16M.raw
Sample: 103613-001-018 (20x dil.)
Well Location: B3

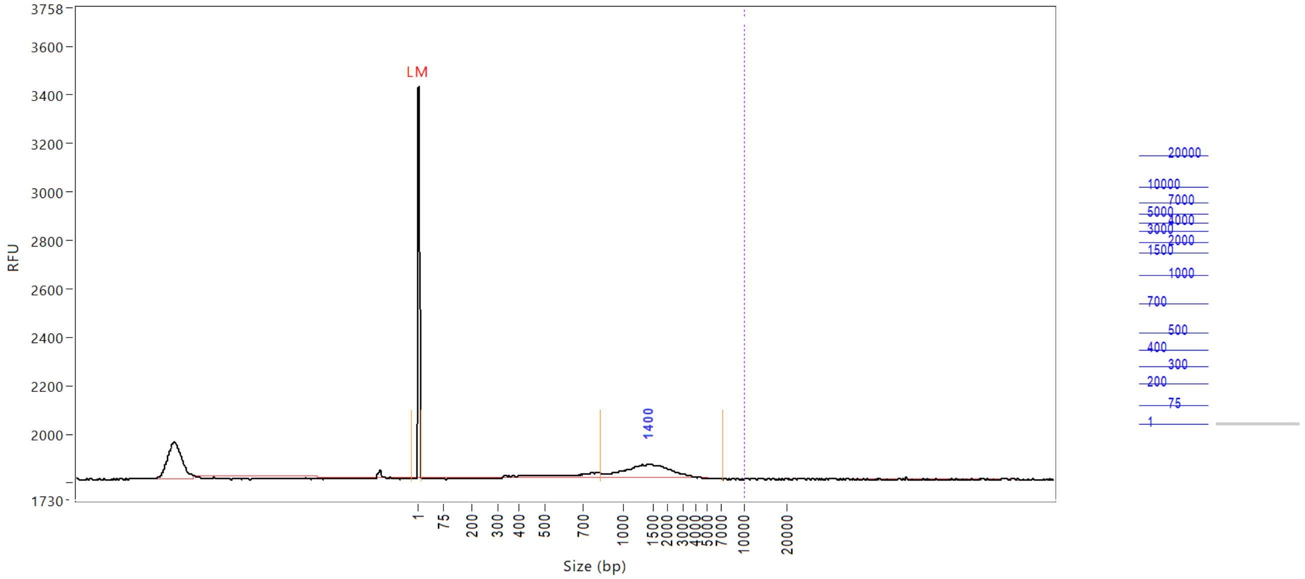

Peak	Size (bp)	Conc. (ng/uL)	From (bp)	To (bp)	Avg. Size (bp)	CV%	RFU	Corr. Peak Area
1	1 (LM)	0.0328	0	8	0	-311.40	1608	11.910
2	1400	0.1617	832	7159	1510	37.46	56	4.892

TIC:	0.1617	ng/uL
TIM:	0.1762	nmole/L
Total Conc.:	0.2158	ng/uL
GQN:	0.1	

Sample Peak Width (sec): 50   Sample Min Peak Height: 50   Sample Baseline V to V?: Y   Sample Baseline V to V pts: 3
Sample Filter: Binomial   # of Pts for Filter: 3   Sample Start Region (min): 0   Sample End Region (min): 50
Manual Baseline Start (min): 6   Manual Baseline End (min): 48
Marker Peak Width (sec): 5   Marker Min Peak Height: 200   Marker Baseline V to V?: Y   Marker Baseline V to V pts: 3
Lower Marker Selection: First Peak > 200 RFU   Upper Marker Selection: Last Peak > 200 RFU
Ladder Size (bp): 1, 75, 200, 300, 400, 500, 700, 1000, 1500, 2000, 3000, 4000, 5000, 7000, 10000, 20000
Quantification Using: Ladder   Final Concentration (ng/uL): 1.0417   Dilution Factor: 12.0
Size Threshold (b.p.): 10000

 Copyright 2015 Advanced Analytical Technologies, Inc.

Data File: 2019 06 18 13H 16M.raw
Sample: 103613-001-019 (20x dil.)
Well Location: C3

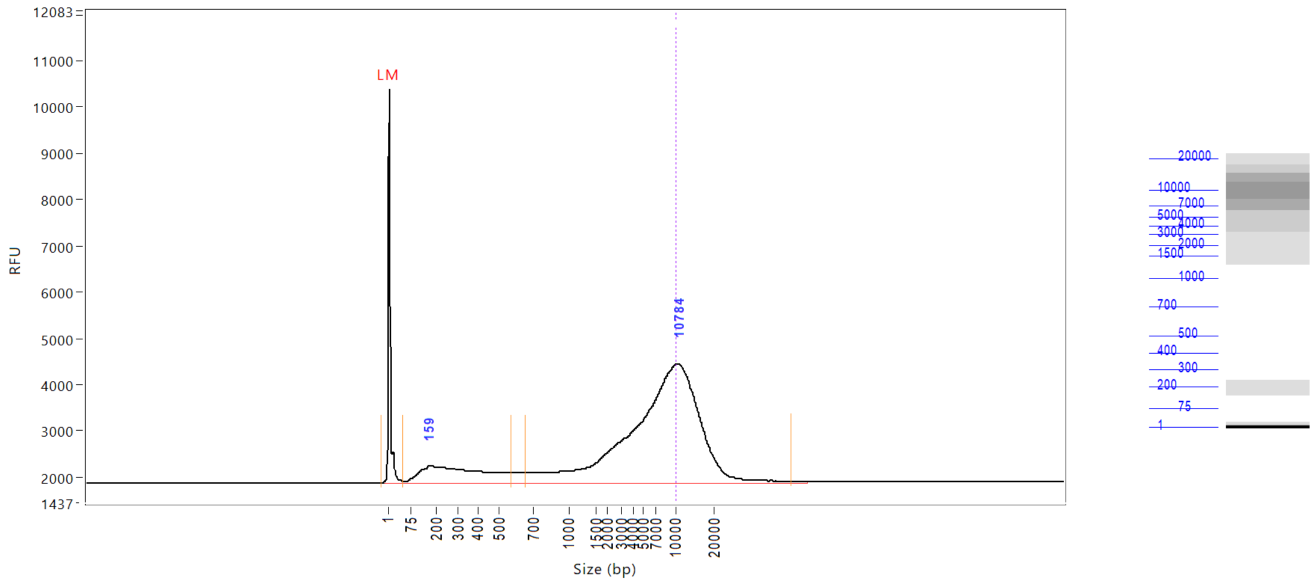

Peak	Size (bp)	Conc. (ng/uL)	From (bp)	To (bp)	Avg. Size (bp)	CV%	RFU	Corr. Peak Area
1	1 (LM)	0.0328	0	47	3	266.54	8507	59.207
2	159	0.4674	47	569	288	46.60	362	70.307
3	10784	2.5740	649	40277	8371	69.18	2556	387.155

TIC:	3.0414	ng/uL
TIM:	3.1759	nmole/L
Total Conc.:	3.1174	ng/uL
GQN:	2.9	

Sample Peak Width (sec): 50 Sample Min Peak Height: 50 Sample Baseline V to V?: Y Sample Baseline V to V pts: 3
Sample Filter: Binomial # of Pts for Filter: 3 Sample Start Region (min): 0 Sample End Region (min): 50
Manual Baseline Start (min): 6 Manual Baseline End (min): 48
Marker Peak Width (sec): 5 Marker Min Peak Height: 200 Marker Baseline V to V?: Y Marker Baseline V to V pts: 3
Lower Marker Selection: First Peak > 200 RFU Upper Marker Selection: Last Peak > 200 RFU
Ladder Size (bp): 1, 75, 200, 300, 400, 500, 700, 1000, 1500, 2000, 3000, 4000, 5000, 7000, 10000, 20000
Quantification Using: Ladder Final Concentration (ng/uL): 1.0417 Dilution Factor: 12.0
Size Threshold (b.p.): 10000

 Copyright 2015 Advanced Analytical Technologies, Inc.

Data File: 2019 06 18 13H 16M.raw
Sample: 103613-001-020 (20x dil.)
Well Location: D3

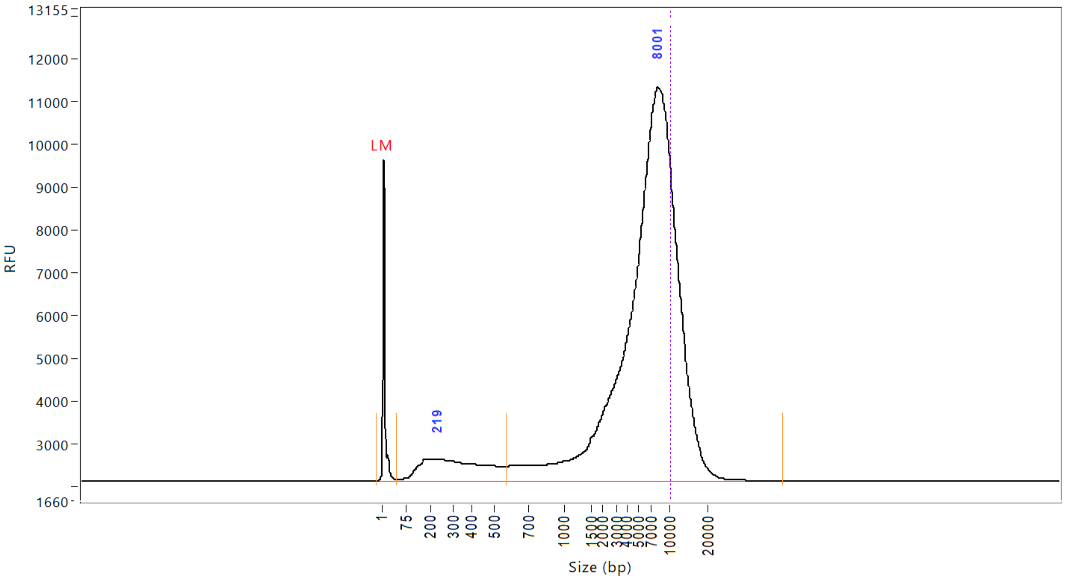

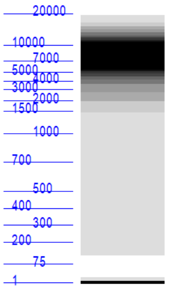

Peak	Size (bp)	Conc. (ng/uL)	From (bp)	To (bp)	Avg. Size (bp)	CV%	RFU	Corr. Peak Area
1	1 (LM)	0.0328	0	45	2	350.10	7499	52.311
2	219	0.7984	45	567	304	42.81	508	106.095
3	8001	7.1584	567	39414	6998	57.27	9188	951.291

TIC:	7.9568	ng/uL
TIM:	6.0040	nmole/L
Total Conc.:	7.9648	ng/uL
GQN:	1.8	

```
Sample Peak Width (sec): 50   Sample Min Peak Height: 50    Sample Baseline V to V?: Y    Sample Baseline V to V pts: 3
Sample Filter: Binomial       # of Pts for Filter: 3        Sample Start Region (min): 0  Sample End Region (min): 50
Manual Baseline Start (min): 6      Manual Baseline End (min): 48
Marker Peak Width (sec): 5    Marker Min Peak Height: 200   Marker Baseline V to V?: Y    Marker Baseline V to V pts: 3
Lower Marker Selection: First Peak > 200 RFU                Upper Marker Selection: Last Peak > 200 RFU
Ladder Size (bp): 1, 75, 200, 300, 400, 500, 700, 1000, 1500, 2000, 3000, 4000, 5000, 7000, 10000, 20000
Quantification Using: Ladder          Final Concentration (ng/uL): 1.0417          Dilution Factor: 12.0
Size Threshold (b.p.): 10000
```

 Copyright 2015 Advanced Analytical Technologies, Inc.

Data File: 2019 06 18 13H 16M.raw
Sample: 103613-001-021 (20x dil.)
Well Location: E3

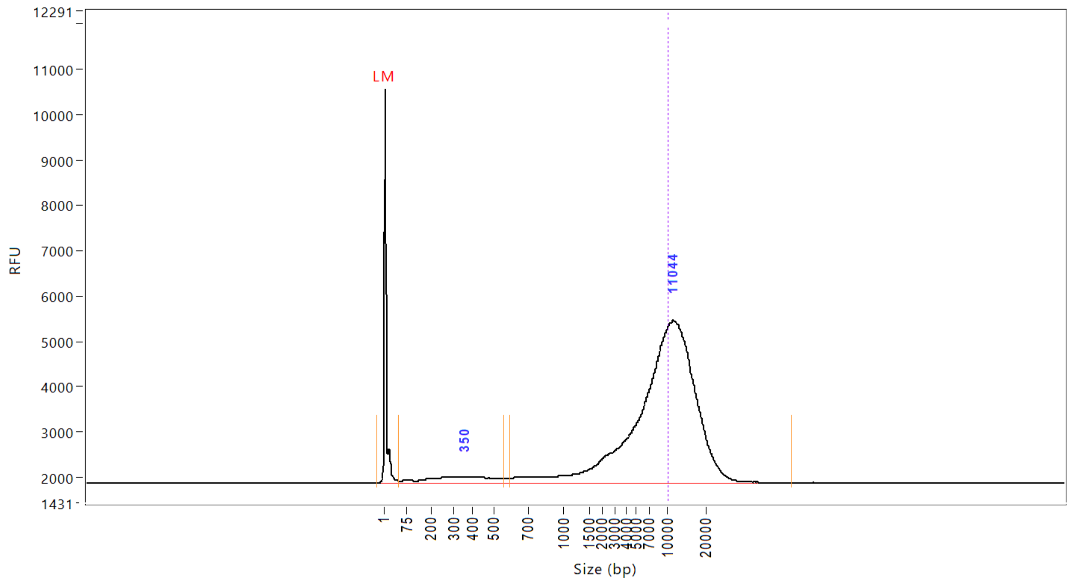

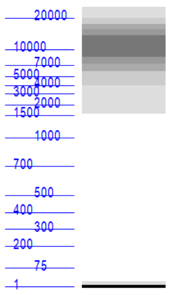

Peak	Size (bp)	Conc. (ng/uL)	From (bp)	To (bp)	Avg. Size (bp)	CV%	RFU	Corr. Peak Area
1	1 (LM)	0.0328	0	45	2	299.44	8682	60.687
2	350	0.1773	45	554	309	44.05	120	27.327
3	11044	3.1086	587	42866	9810	57.32	3575	479.257

TIC:	3.2859	ng/uL
TIM:	1.4653	nmole/L
Total Conc.:	3.3141	ng/uL
GQN:	4.3	

Sample Peak Width (sec): 50 Sample Min Peak Height: 50 Sample Baseline V to V?: Y Sample Baseline V to V pts: 3
Sample Filter: Binomial # of Pts for Filter: 3 Sample Start Region (min): 0 Sample End Region (min): 50
Manual Baseline Start (min): 6 Manual Baseline End (min): 48
Marker Peak Width (sec): 5 Marker Min Peak Height: 200 Marker Baseline V to V?: Y Marker Baseline V to V pts: 3
Lower Marker Selection: First Peak > 200 RFU Upper Marker Selection: Last Peak > 200 RFU
Ladder Size (bp): 1, 75, 200, 300, 400, 500, 700, 1000, 1500, 2000, 3000, 4000, 5000, 7000, 10000, 20000
Quantification Using: Ladder Final Concentration (ng/uL): 1.0417 Dilution Factor: 12.0
Size Threshold (b.p.): 10000

 Copyright 2015 Advanced Analytical Technologies, Inc.

Data File: 2019 06 18 13H 16M.raw
Sample: 103613-001-022 (20x dil.)
Well Location: F3

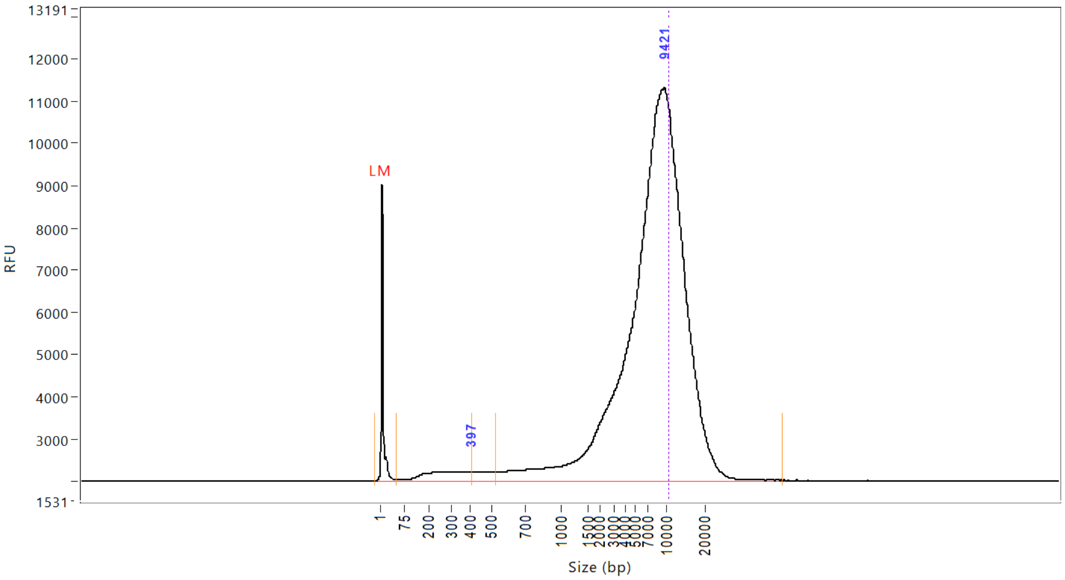

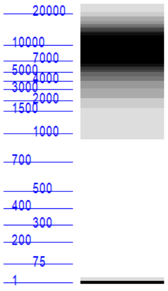

Peak	Size (bp)	Conc. (ng/uL)	From (bp)	To (bp)	Avg. Size (bp)	CV%	RFU	Corr. Peak Area
1	1 (LM)	0.0328	0	47	2	375.08	7043	50.374
2	397	0.2508	47	403	257	33.80	214	32.097
3	9421	8.3829	521	40104	8365	57.31	9321	1072.771

TIC:	8.6337	ng/uL
TIM:	3.2549	nmole/L
Total Conc.:	8.7568	ng/uL
GQN:	3.1	

Sample Peak Width (sec): 50 Sample Min Peak Height: 50 Sample Baseline V to V?: Y Sample Baseline V to V pts: 3
Sample Filter: Binomial # of Pts for Filter: 3 Sample Start Region (min): 0 Sample End Region (min): 50
Manual Baseline Start (min): 6 Manual Baseline End (min): 48
Marker Peak Width (sec): 5 Marker Min Peak Height: 200 Marker Baseline V to V?: Y Marker Baseline V to V pts: 3
Lower Marker Selection: First Peak > 200 RFU Upper Marker Selection: Last Peak > 200 RFU
Ladder Size (bp): 1, 75, 200, 300, 400, 500, 700, 1000, 1500, 2000, 3000, 4000, 5000, 7000, 10000, 20000
Quantification Using: Ladder Final Concentration (ng/uL): 1.0417 Dilution Factor: 12.0
Size Threshold (b.p.): 10000

 Copyright 2015 Advanced Analytical Technologies, Inc.

Data File: 2019 06 18 13H 16M.raw
Sample: 103613-001-023 (20x dil.)
Well Location: G3

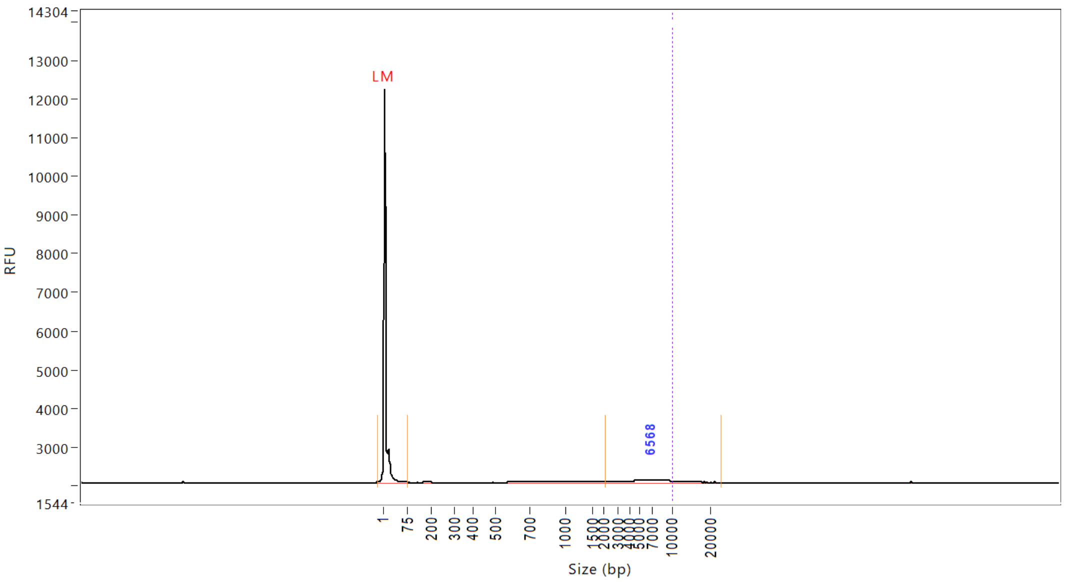

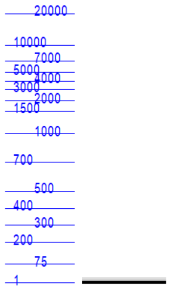

Peak	Size (bp)	Conc. (ng/uL)	From (bp)	To (bp)	Avg. Size (bp)	CV%	RFU	Corr. Peak Area
1	1 (LM)	0.0328	0	74	2	348.44	10190	71.690
2	6568	0.0446	2120	23020	7949	56.73	70	8.125

TIC:	0.0446	ng/uL
TIM:	0.0092	nmole/L
Total Conc.:	0.1179	ng/uL
GQN:	2.8	

Sample Peak Width (sec): 50 Sample Min Peak Height: 50 Sample Baseline V to V?: Y Sample Baseline V to V pts: 3
Sample Filter: Binomial # of Pts for Filter: 3 Sample Start Region (min): 0 Sample End Region (min): 50
Manual Baseline Start (min): 6 Manual Baseline End (min): 48
Marker Peak Width (sec): 5 Marker Min Peak Height: 200 Marker Baseline V to V?: Y Marker Baseline V to V pts: 3
Lower Marker Selection: First Peak > 200 RFU Upper Marker Selection: Last Peak > 200 RFU
Ladder Size (bp): 1, 75, 200, 300, 400, 500, 700, 1000, 1500, 2000, 3000, 4000, 5000, 7000, 10000, 20000
Quantification Using: Ladder Final Concentration (ng/uL): 1.0417 Dilution Factor: 12.0
Size Threshold (b.p.): 10000

 Copyright 2015 Advanced Analytical Technologies, Inc.

Data File: 2019 06 18 13H 16M.raw
Sample: 103613-001-024 (20x dil.)
Well Location: H3

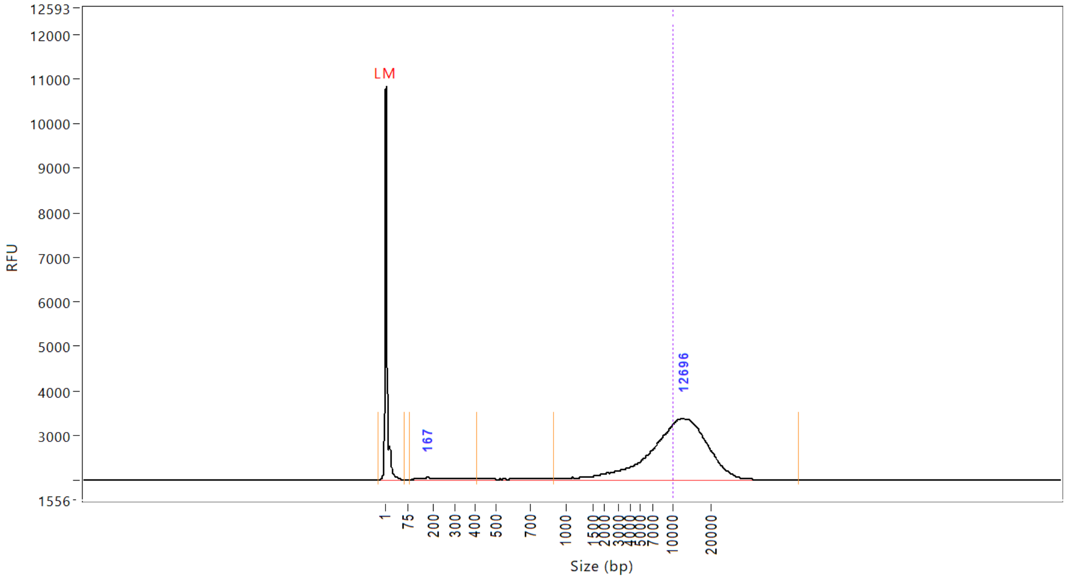

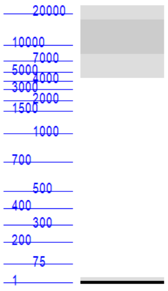

Peak	Size (bp)	Conc. (ng/uL)	From (bp)	To (bp)	Avg. Size (bp)	CV%	RFU	Corr. Peak Area
1	1 (LM)	0.0328	0	61	2	380.82	8820	62.806
2	167	0.0281	78	410	229	37.60	58	4.479
3	12696	1.2364	897	42866	11588	50.84	1376	197.275

TIC:	1.2645	ng/uL
TIM:	0.3773	nmole/L
Total Conc.:	1.2927	ng/uL
GQN:	5.4	

```
Sample Peak Width (sec): 50   Sample Min Peak Height: 50    Sample Baseline V to V?: Y    Sample Baseline V to V pts: 3
Sample Filter: Binomial       # of Pts for Filter: 3        Sample Start Region (min): 0  Sample End Region (min): 50
Manual Baseline Start (min): 6     Manual Baseline End (min): 48
Marker Peak Width (sec): 5    Marker Min Peak Height: 200   Marker Baseline V to V?: Y    Marker Baseline V to V pts: 3
Lower Marker Selection: First Peak > 200 RFU                Upper Marker Selection: Last Peak > 200 RFU
Ladder Size (bp): 1, 75, 200, 300, 400, 500, 700, 1000, 1500, 2000, 3000, 4000, 5000, 7000, 10000, 20000
Quantification Using: Ladder          Final Concentration (ng/uL): 1.0417          Dilution Factor: 12.0
Size Threshold (b.p.): 10000
```


Copyright 2015 Advanced Analytical Technologies, Inc.

Data File: 2019 06 18 13H 16M.raw
Sample: 103613-001-025 (20x dil.)
Well Location: A4

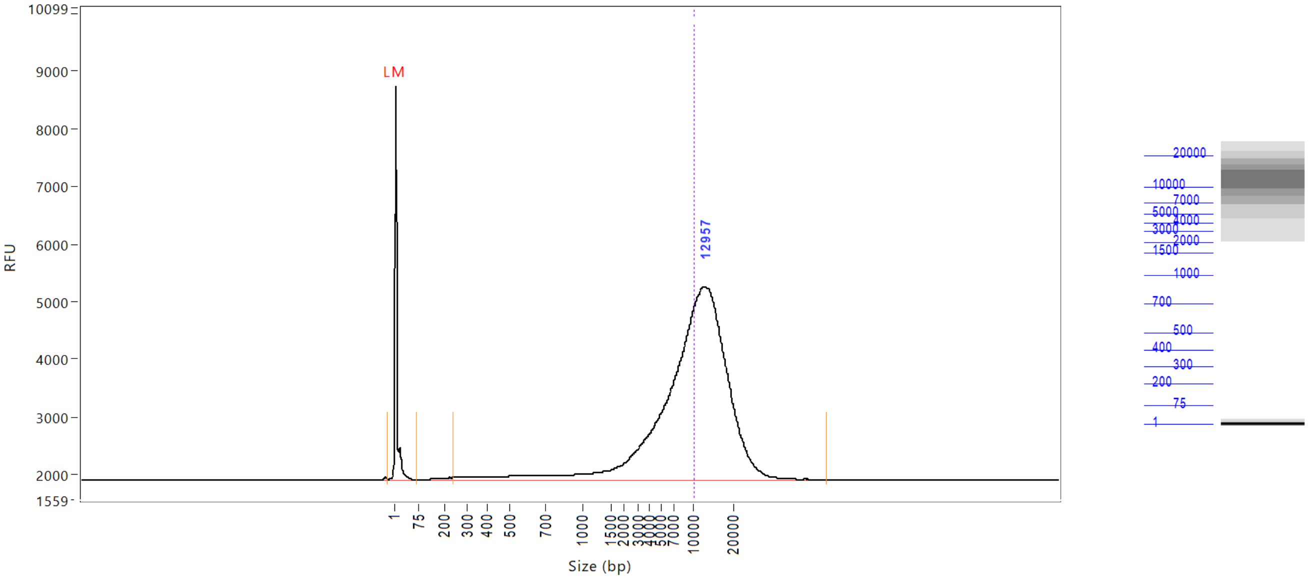

Peak	Size (bp)	Conc. (ng/uL)	From (bp)	To (bp)	Avg. Size (bp)	CV%	RFU	Corr. Peak Area
1	1 (LM)	0.0328	0	66	3	268.67	6825	48.574
2	12957	3.7897	233	43728	10741	55.98	3348	467.641

TIC:	3.7897	ng/uL
TIM:	0.5809	nmole/L
Total Conc.:	3.8122	ng/uL
GQN:	5.3	

Sample Peak Width (sec): 50 Sample Min Peak Height: 50 Sample Baseline V to V?: Y Sample Baseline V to V pts: 3
Sample Filter: Binomial # of Pts for Filter: 3 Sample Start Region (min): 0 Sample End Region (min): 50
Manual Baseline Start (min): 6 Manual Baseline End (min): 48
Marker Peak Width (sec): 5 Marker Min Peak Height: 200 Marker Baseline V to V?: Y Marker Baseline V to V pts: 3
Lower Marker Selection: First Peak > 200 RFU Upper Marker Selection: Last Peak > 200 RFU
Ladder Size (bp): 1, 75, 200, 300, 400, 500, 700, 1000, 1500, 2000, 3000, 4000, 5000, 7000, 10000, 20000
Quantification Using: Ladder Final Concentration (ng/uL): 1.0417 Dilution Factor: 12.0
Size Threshold (b.p.): 10000

 Copyright 2015 Advanced Analytical Technologies, Inc.

Data File: 2019 06 18 13H 16M.raw
Sample: 103613-001-026 (20x dil.)
Well Location: B4

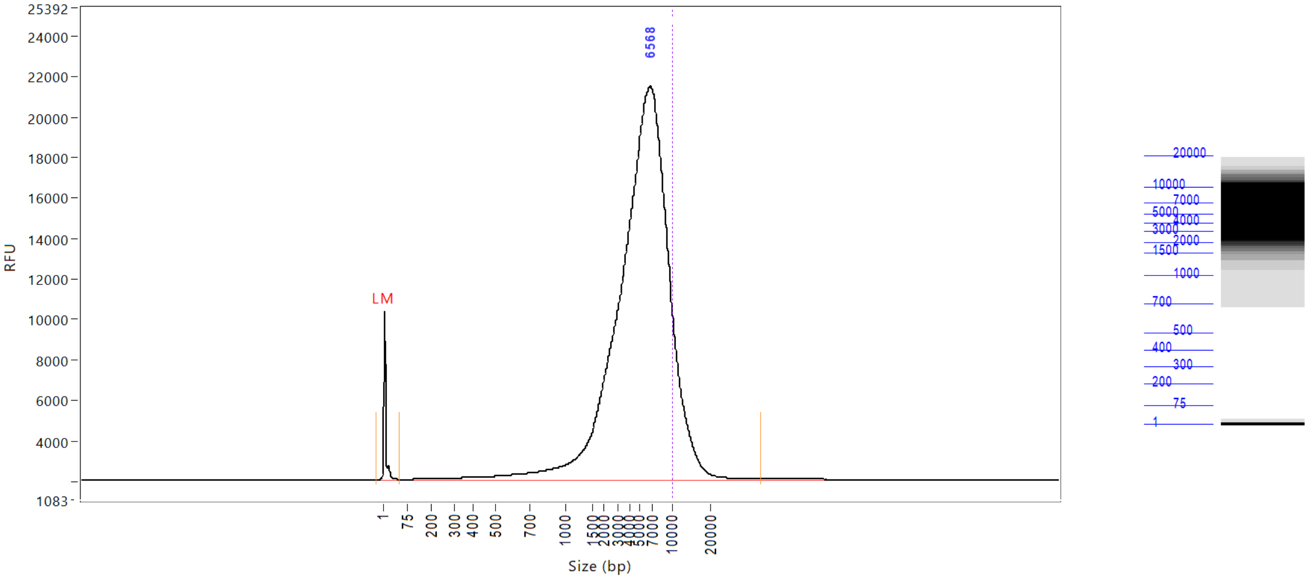

Peak	Size (bp)	Conc. (ng/uL)	From (bp)	To (bp)	Avg. Size (bp)	CV%	RFU	Corr. Peak Area
1	1 (LM)	0.0328	0	50	2	357.22	8350	58.035
2	6568	13.7462	50	33202	5774	60.03	19437	2026.654

TIC:	13.7462	ng/uL
TIM:	3.9193	nmole/L
Total Conc.:	13.8695	ng/uL
GQN:	1.0	

Sample Peak Width (sec): 50 Sample Min Peak Height: 50 Sample Baseline V to V?: Y Sample Baseline V to V pts: 3
Sample Filter: Binomial # of Pts for Filter: 3 Sample Start Region (min): 0 Sample End Region (min): 50
Manual Baseline Start (min): 6 Manual Baseline End (min): 48
Marker Peak Width (sec): 5 Marker Min Peak Height: 200 Marker Baseline V to V?: Y Marker Baseline V to V pts: 3
Lower Marker Selection: First Peak > 200 RFU Upper Marker Selection: Last Peak > 200 RFU
Ladder Size (bp): 1, 75, 200, 300, 400, 500, 700, 1000, 1500, 2000, 3000, 4000, 5000, 7000, 10000, 20000
Quantification Using: Ladder Final Concentration (ng/uL): 1.0417 Dilution Factor: 12.0
Size Threshold (b.p.): 10000

 Copyright 2015 Advanced Analytical Technologies, Inc.

Data File: 2019 06 18 13H 16M.raw
Sample: 103613-001-027 (20x dil.)
Well Location: C4

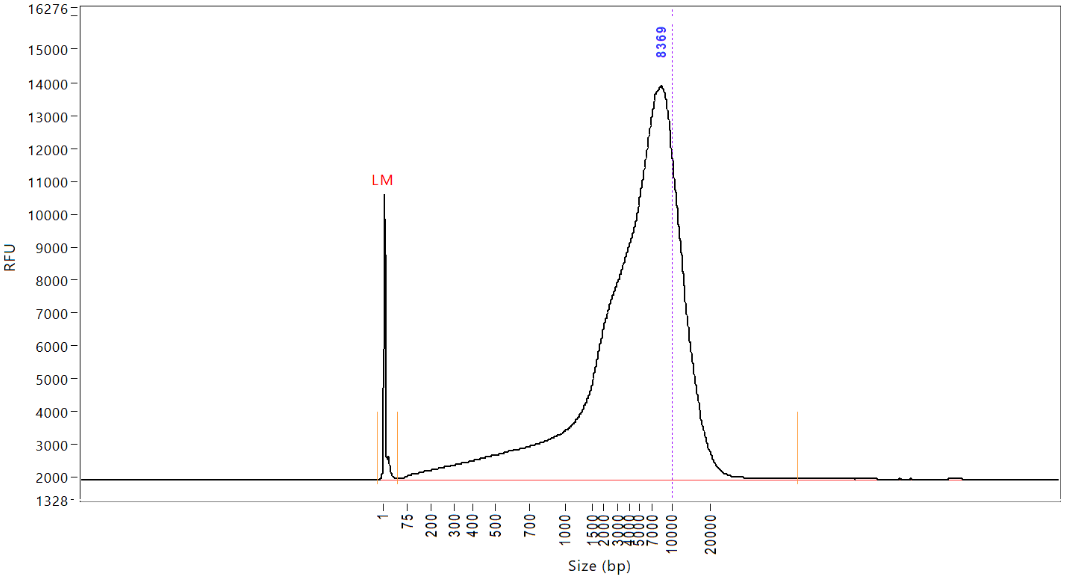

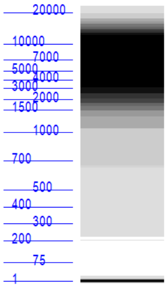

Peak	Size (bp)	Conc. (ng/uL)	From (bp)	To (bp)	Avg. Size (bp)	CV%	RFU	Corr. Peak Area
1	1 (LM)	0.0328	0	46	2	288.02	8672	59.823
2	8369	12.2727	46	42866	5887	79.50	11943	1865.130

TIC:	12.2727	ng/uL
TIM:	3.4320	nmole/L
Total Conc.:	12.3069	ng/uL
GQN:	1.7	

Sample Peak Width (sec): 50 Sample Min Peak Height: 50 Sample Baseline V to V?: Y Sample Baseline V to V pts: 3
Sample Filter: Binomial # of Pts for Filter: 3 Sample Start Region (min): 0 Sample End Region (min): 50
Manual Baseline Start (min): 6 Manual Baseline End (min): 48
Marker Peak Width (sec): 5 Marker Min Peak Height: 200 Marker Baseline V to V?: Y Marker Baseline V to V pts: 3
Lower Marker Selection: First Peak > 200 RFU Upper Marker Selection: Last Peak > 200 RFU
Ladder Size (bp): 1, 75, 200, 300, 400, 500, 700, 1000, 1500, 2000, 3000, 4000, 5000, 7000, 10000, 20000
Quantification Using: Ladder Final Concentration (ng/uL): 1.0417 Dilution Factor: 12.0
Size Threshold (b.p.): 10000

 Copyright 2015 Advanced Analytical Technologies, Inc.

Data File: 2019 06 18 13H 16M.raw
Sample: 103613-001-028 (20x dil.)
Well Location: D4

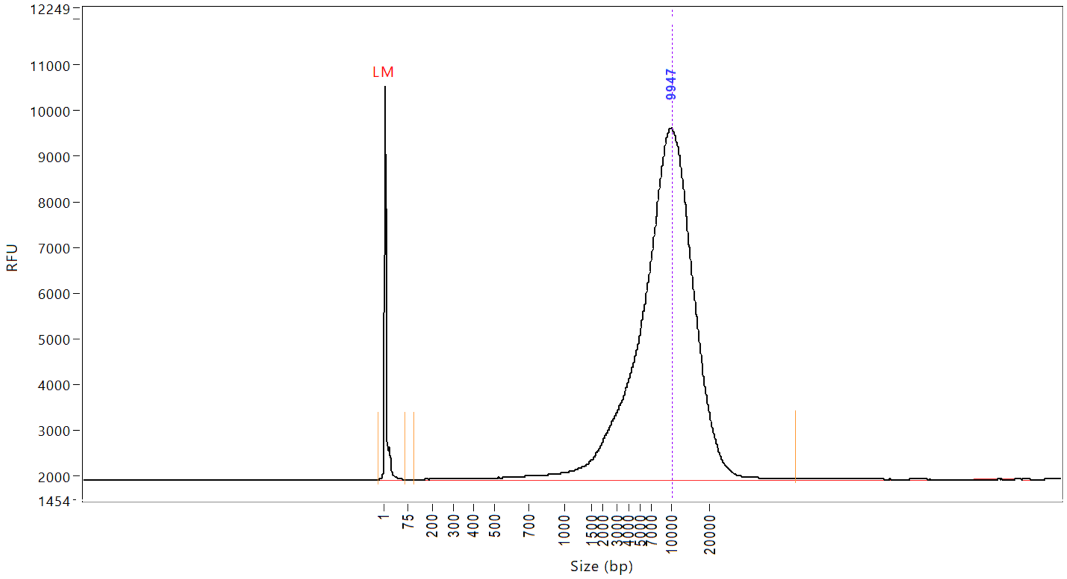

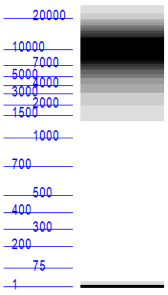

Peak	Size (bp)	Conc. (ng/uL)	From (bp)	To (bp)	Avg. Size (bp)	CV%	RFU	Corr. Peak Area
1	1 (LM)	0.0328	0	68	2	353.83	8626	60.059
2	9947	6.0662	106	42607	9448	54.47	7707	925.538

TIC:	6.0662	ng/uL
TIM:	1.0570	nmole/L
Total Conc.:	6.0911	ng/uL
GQN:	4.1	

Sample Peak Width (sec): 50 Sample Min Peak Height: 50 Sample Baseline V to V?: Y Sample Baseline V to V pts: 3
Sample Filter: Binomial # of Pts for Filter: 3 Sample Start Region (min): 0 Sample End Region (min): 50
Manual Baseline Start (min): 6 Manual Baseline End (min): 48
Marker Peak Width (sec): 5 Marker Min Peak Height: 200 Marker Baseline V to V?: Y Marker Baseline V to V pts: 3
Lower Marker Selection: First Peak > 200 RFU Upper Marker Selection: Last Peak > 200 RFU
Ladder Size (bp): 1, 75, 200, 300, 400, 500, 700, 1000, 1500, 2000, 3000, 4000, 5000, 7000, 10000, 20000
Quantification Using: Ladder Final Concentration (ng/uL): 1.0417 Dilution Factor: 12.0
Size Threshold (b.p.): 10000

 Copyright 2015 Advanced Analytical Technologies, Inc.

Data File: 2019 06 18 13H 16M.raw
Sample: 103613-001-029 (20x dil.)
Well Location: E4

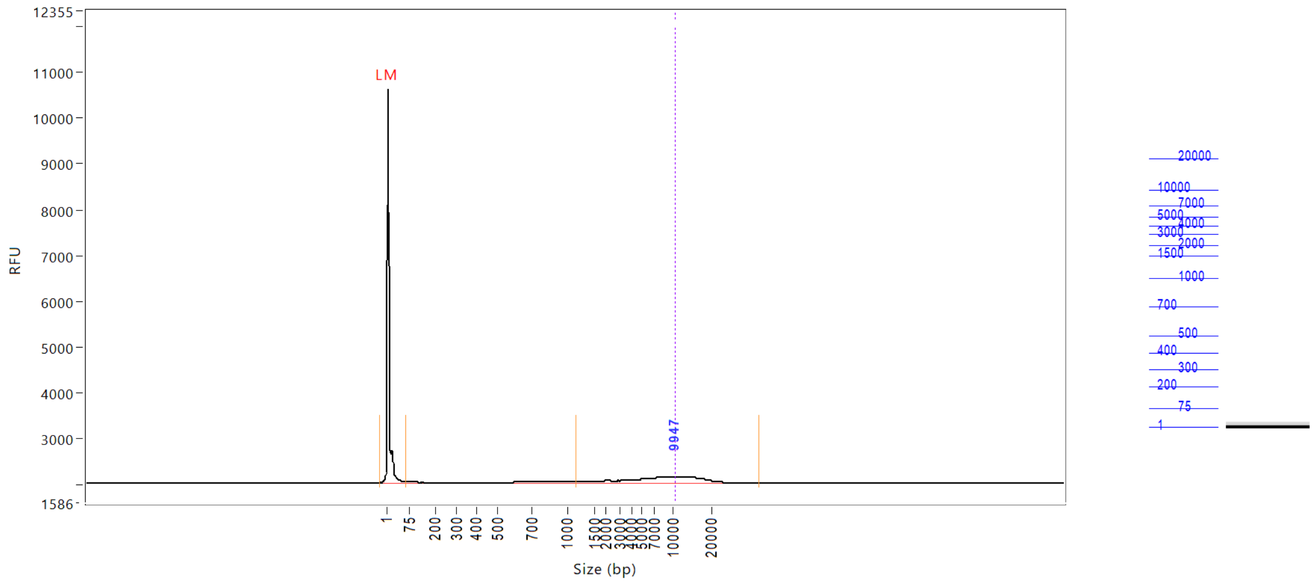

Peak	Size (bp)	Conc. (ng/uL)	From (bp)	To (bp)	Avg. Size (bp)	CV%	RFU	Corr. Peak Area
1	1 (LM)	0.0328	0	58	3	282.58	8603	59.757
2	9947	0.1426	1169	32511	8987	60.49	134	21.650

TIC:	0.1426	ng/uL
TIM:	0.0261	nmole/L
Total Conc.:	0.1760	ng/uL
GQN:	3.1	

Sample Peak Width (sec): 50 Sample Min Peak Height: 50 Sample Baseline V to V?: Y Sample Baseline V to V pts: 3
Sample Filter: Binomial # of Pts for Filter: 3 Sample Start Region (min): 0 Sample End Region (min): 50
Manual Baseline Start (min): 6 Manual Baseline End (min): 48
Marker Peak Width (sec): 5 Marker Min Peak Height: 200 Marker Baseline V to V?: Y Marker Baseline V to V pts: 3
Lower Marker Selection: First Peak > 200 RFU Upper Marker Selection: Last Peak > 200 RFU
Ladder Size (bp): 1, 75, 200, 300, 400, 500, 700, 1000, 1500, 2000, 3000, 4000, 5000, 7000, 10000, 20000
Quantification Using: Ladder Final Concentration (ng/uL): 1.0417 Dilution Factor: 12.0
Size Threshold (b.p.): 10000

 Copyright 2015 Advanced Analytical Technologies, Inc.

Data File: 2019 06 18 13H 16M.raw
Sample: 103613-001-030 (20x dil.)
Well Location: F4

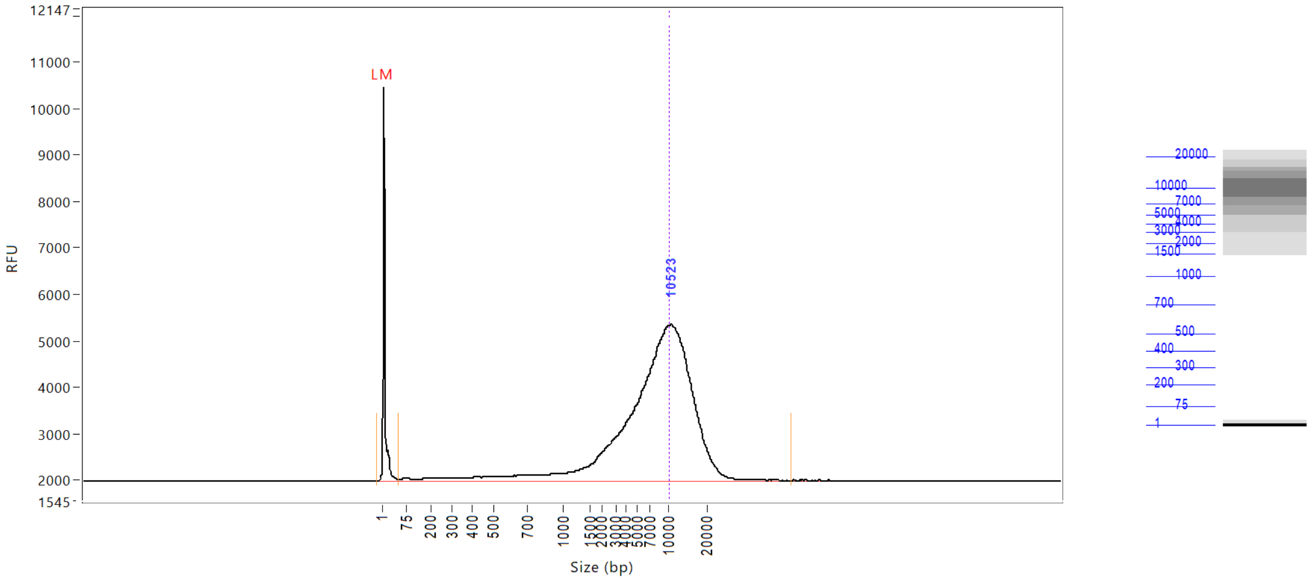

Peak	Size (bp)	Conc. (ng/uL)	From (bp)	To (bp)	Avg. Size (bp)	CV%	RFU	Corr. Peak Area
1	1 (LM)	0.0328	0	49	2	304.91	8473	58.820
2	10523	3.1891	49	42262	8549	64.35	3376	476.530

TIC:	3.1891	ng/uL
TIM:	0.6141	nmole/L
Total Conc.:	3.2003	ng/uL
GQN:	3.7	

Sample Peak Width (sec): 50 Sample Min Peak Height: 50 Sample Baseline V to V?: Y Sample Baseline V to V pts: 3
Sample Filter: Binomial # of Pts for Filter: 3 Sample Start Region (min): 0 Sample End Region (min): 50
Manual Baseline Start (min): 6 Manual Baseline End (min): 48
Marker Peak Width (sec): 5 Marker Min Peak Height: 200 Marker Baseline V to V?: Y Marker Baseline V to V pts: 3
Lower Marker Selection: First Peak > 200 RFU Upper Marker Selection: Last Peak > 200 RFU
Ladder Size (bp): 1, 75, 200, 300, 400, 500, 700, 1000, 1500, 2000, 3000, 4000, 5000, 7000, 10000, 20000
Quantification Using: Ladder Final Concentration (ng/uL): 1.0417 Dilution Factor: 12.0
Size Threshold (b.p.): 10000

 Copyright 2015 Advanced Analytical Technologies, Inc.

Data File: 2019 06 18 13H 16M.raw
Sample: 103613-001-031 (20x dil.)
Well Location: G4

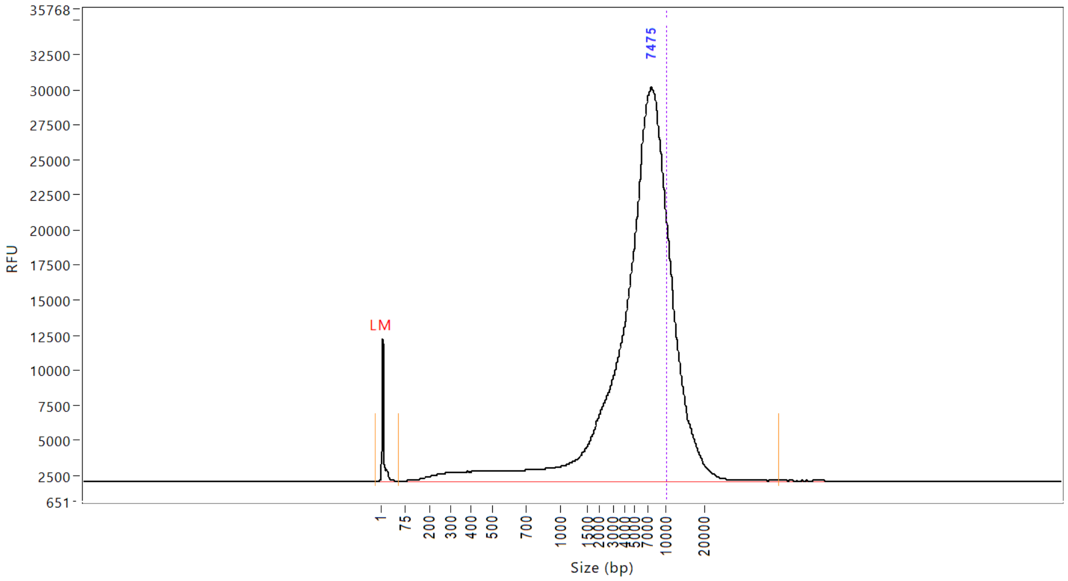

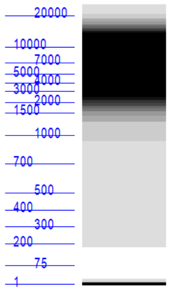

Peak	Size (bp)	Conc. (ng/uL)	From (bp)	To (bp)	Avg. Size (bp)	CV%	RFU	Corr. Peak Area
1	1 (LM)	0.0328	0	53	3	274.11	10198	70.844
2	7475	16.3699	53	39932	6638	63.17	28073	2946.118

TIC:	16.3699	ng/uL
TIM:	4.0599	nmole/L
Total Conc.:	16.4484	ng/uL
GQN:	1.7	

Sample Peak Width (sec): 50 Sample Min Peak Height: 50 Sample Baseline V to V?: Y Sample Baseline V to V pts: 3
Sample Filter: Binomial # of Pts for Filter: 3 Sample Start Region (min): 0 Sample End Region (min): 50
Manual Baseline Start (min): 6 Manual Baseline End (min): 48
Marker Peak Width (sec): 5 Marker Min Peak Height: 200 Marker Baseline V to V?: Y Marker Baseline V to V pts: 3
Lower Marker Selection: First Peak > 200 RFU Upper Marker Selection: Last Peak > 200 RFU
Ladder Size (bp): 1, 75, 200, 300, 400, 500, 700, 1000, 1500, 2000, 3000, 4000, 5000, 7000, 10000, 20000
Quantification Using: Ladder Final Concentration (ng/uL): 1.0417 Dilution Factor: 12.0
Size Threshold (b.p.): 10000

Copyright 2015 Advanced Analytical Technologies, Inc.

Data File: 2019 06 18 13H 16M.raw
Sample: 103613-001-032 (20x dil.)
Well Location: H4

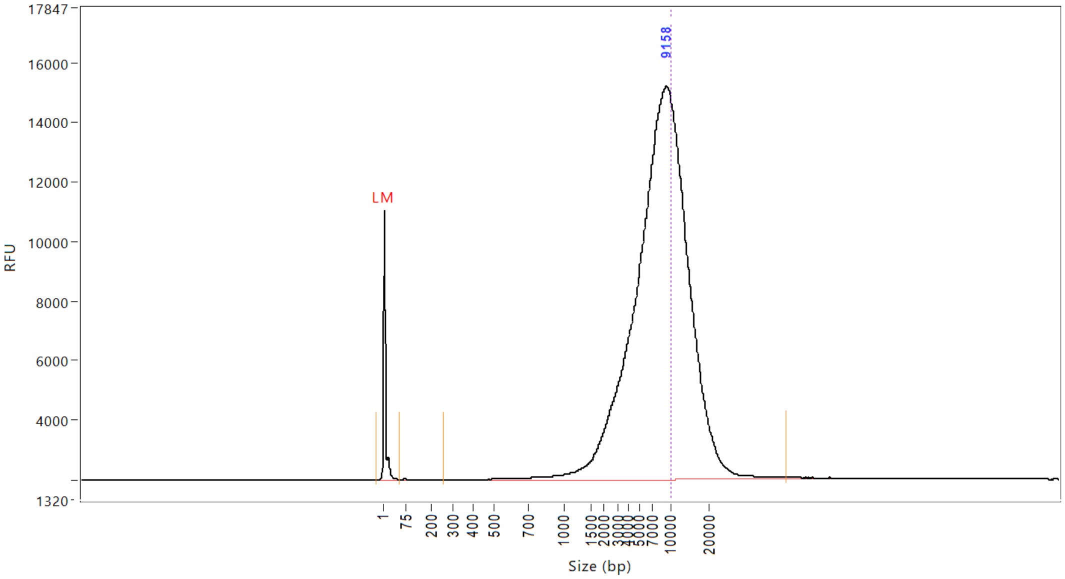

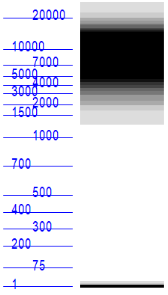

Peak	Size (bp)	Conc. (ng/uL)	From (bp)	To (bp)	Avg. Size (bp)	CV%	RFU	Corr. Peak Area
1	1 (LM)	0.0328	0	50	2	301.08	9030	61.905
2	9158	9.6901	253	40018	8832	54.26	13188	1523.895

TIC:	9.6901	ng/uL
TIM:	1.8063	nmole/L
Total Conc.:	9.7610	ng/uL
GQN:	3.4	

```
Sample Peak Width (sec): 50   Sample Min Peak Height: 50    Sample Baseline V to V?: Y    Sample Baseline V to V pts: 3
Sample Filter: Binomial       # of Pts for Filter: 3        Sample Start Region (min): 0  Sample End Region (min): 50
Manual Baseline Start (min): 6     Manual Baseline End (min): 48
Marker Peak Width (sec): 5    Marker Min Peak Height: 200   Marker Baseline V to V?: Y    Marker Baseline V to V pts: 3
Lower Marker Selection: First Peak > 200 RFU                Upper Marker Selection: Last Peak > 200 RFU
Ladder Size (bp): 1, 75, 200, 300, 400, 500, 700, 1000, 1500, 2000, 3000, 4000, 5000, 7000, 10000, 20000
Quantification Using: Ladder          Final Concentration (ng/uL): 1.0417          Dilution Factor: 12.0
Size Threshold (b.p.): 10000
```


Copyright 2015 Advanced Analytical Technologies, Inc.

Data File: 2019 06 18 13H 16M.raw
Sample: 103613-001-033 (20x dil.)
Well Location: A5

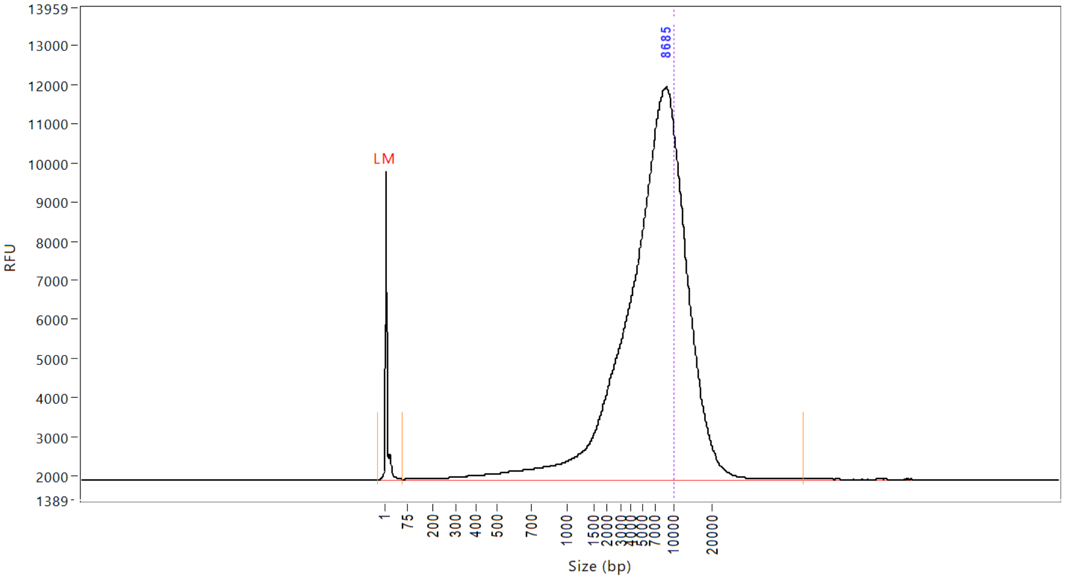

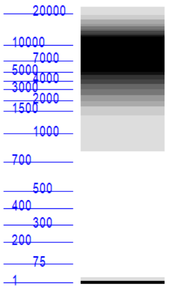

Peak	Size (bp)	Conc. (ng/uL)	From (bp)	To (bp)	Avg. Size (bp)	CV%	RFU	Corr. Peak Area
1	1 (LM)	0.0328	0	55	2	318.46	7874	54.094
2	8685	8.9815	55	43556	7314	62.80	10042	1234.258

TIC:	8.9815	ng/uL
TIM:	2.0216	nmole/L
Total Conc.:	9.0065	ng/uL
GQN:	2.4	

Sample Peak Width (sec): 50 Sample Min Peak Height: 50 Sample Baseline V to V?: Y Sample Baseline V to V pts: 3
Sample Filter: Binomial # of Pts for Filter: 3 Sample Start Region (min): 0 Sample End Region (min): 50
Manual Baseline Start (min): 6 Manual Baseline End (min): 48
Marker Peak Width (sec): 5 Marker Min Peak Height: 200 Marker Baseline V to V?: Y Marker Baseline V to V pts: 3
Lower Marker Selection: First Peak > 200 RFU Upper Marker Selection: Last Peak > 200 RFU
Ladder Size (bp): 1, 75, 200, 300, 400, 500, 700, 1000, 1500, 2000, 3000, 4000, 5000, 7000, 10000, 20000
Quantification Using: Ladder Final Concentration (ng/uL): 1.0417 Dilution Factor: 12.0
Size Threshold (b.p.): 10000

 Copyright 2015 Advanced Analytical Technologies, Inc.

Data File: 2019 06 18 13H 16M.raw
Sample: 103613-001-034 (20x dil.)
Well Location: B5

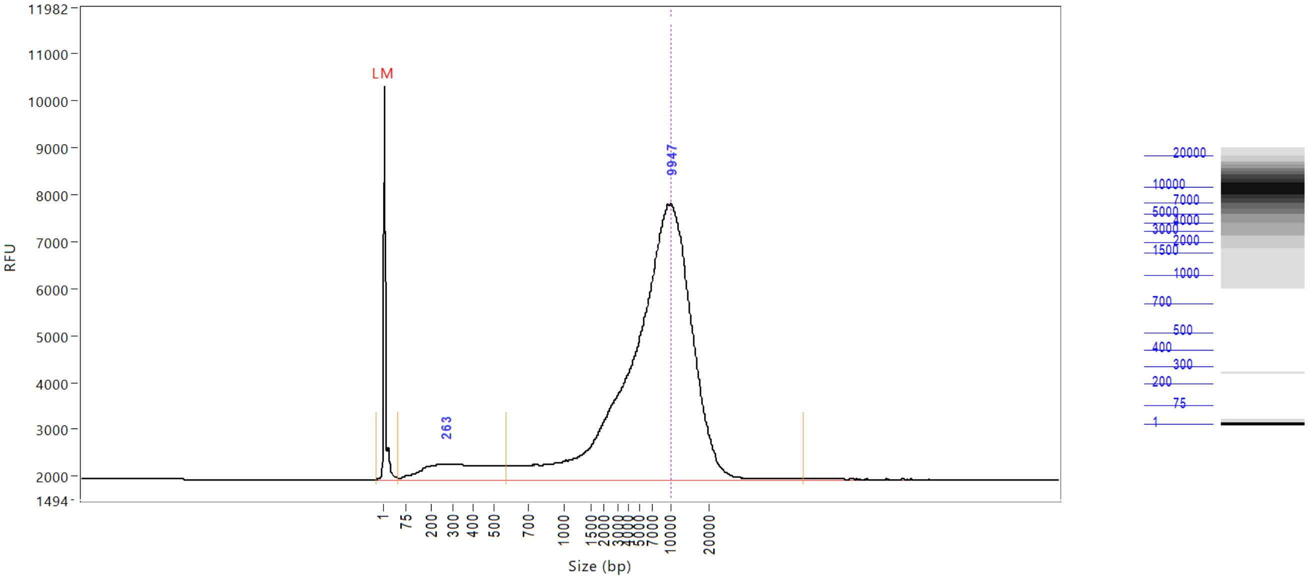

Peak	Size (bp)	Conc. (ng/uL)	From (bp)	To (bp)	Avg. Size (bp)	CV%	RFU	Corr. Peak Area
1	1 (LM)	0.0328	0	44	2	297.83	8374	57.654
2	263	0.4928	44	558	317	41.14	325	72.173
3	9947	5.4007	558	44591	8195	63.71	5880	791.017

TIC:	5.8935	ng/uL
TIM:	3.6421	nmole/L
Total Conc.:	5.9084	ng/uL
GQN:	3.0	

Sample Peak Width (sec): 50 Sample Min Peak Height: 50 Sample Baseline V to V?: Y Sample Baseline V to V pts: 3
Sample Filter: Binomial # of Pts for Filter: 3 Sample Start Region (min): 0 Sample End Region (min): 50
Manual Baseline Start (min): 6 Manual Baseline End (min): 48
Marker Peak Width (sec): 5 Marker Min Peak Height: 200 Marker Baseline V to V?: Y Marker Baseline V to V pts: 3
Lower Marker Selection: First Peak > 200 RFU Upper Marker Selection: Last Peak > 200 RFU
Ladder Size (bp): 1, 75, 200, 300, 400, 500, 700, 1000, 1500, 2000, 3000, 4000, 5000, 7000, 10000, 20000
Quantification Using: Ladder Final Concentration (ng/uL): 1.0417 Dilution Factor: 12.0
Size Threshold (b.p.): 10000

 Copyright 2015 Advanced Analytical Technologies, Inc.

Data File: 2019 06 18 13H 16M.raw
Sample: 103613-001-035 (20x dil.)
Well Location: C5

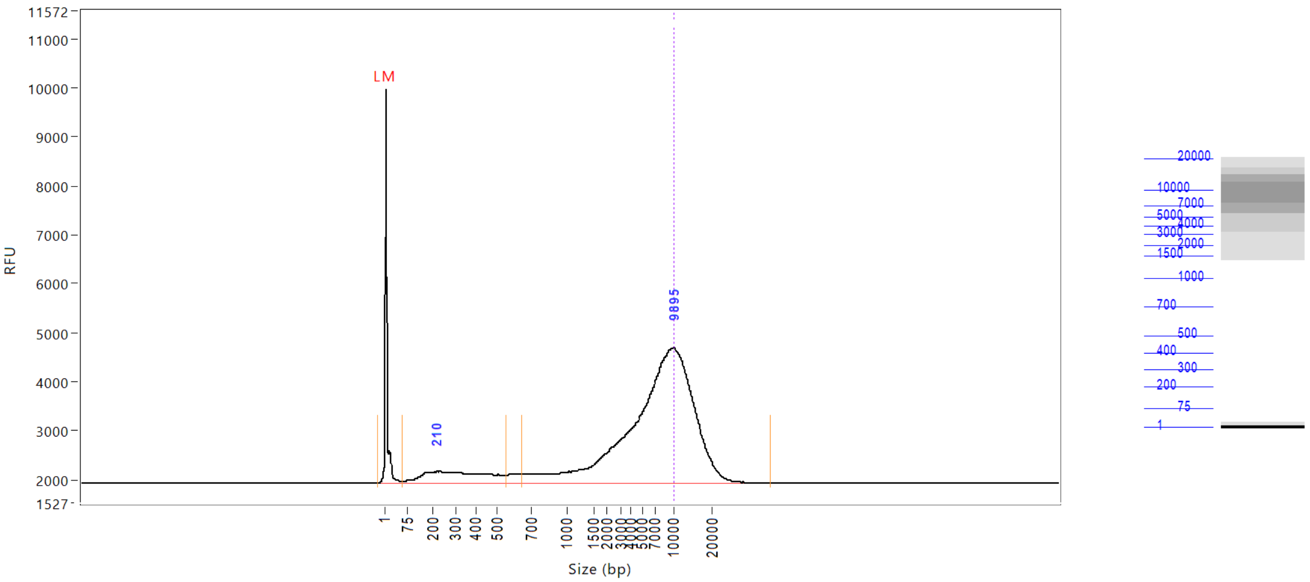

Peak	Size (bp)	Conc. (ng/uL)	From (bp)	To (bp)	Avg. Size (bp)	CV%	RFU	Corr. Peak Area
1	1 (LM)	0.0328	0	54	2	383.43	8030	57.469
2	210	0.3148	54	552	301	42.50	222	45.966
3	9895	2.5254	643	35186	8001	62.29	2771	368.686

TIC:	2.8402	ng/uL
TIM:	2.2402	nmole/L
Total Conc.:	2.8892	ng/uL
GQN:	2.8	

Sample Peak Width (sec): 50 Sample Min Peak Height: 50 Sample Baseline V to V?: Y Sample Baseline V to V pts: 3
Sample Filter: Binomial # of Pts for Filter: 3 Sample Start Region (min): 0 Sample End Region (min): 50
Manual Baseline Start (min): 6 Manual Baseline End (min): 48
Marker Peak Width (sec): 5 Marker Min Peak Height: 200 Marker Baseline V to V?: Y Marker Baseline V to V pts: 3
Lower Marker Selection: First Peak > 200 RFU Upper Marker Selection: Last Peak > 200 RFU
Ladder Size (bp): 1, 75, 200, 300, 400, 500, 700, 1000, 1500, 2000, 3000, 4000, 5000, 7000, 10000, 20000
Quantification Using: Ladder Final Concentration (ng/uL): 1.0417 Dilution Factor: 12.0
Size Threshold (b.p.): 10000

Data File: 2019 06 18 13H 16M.raw
Sample: 103613-001-036 (20x dil.)
Well Location: D5

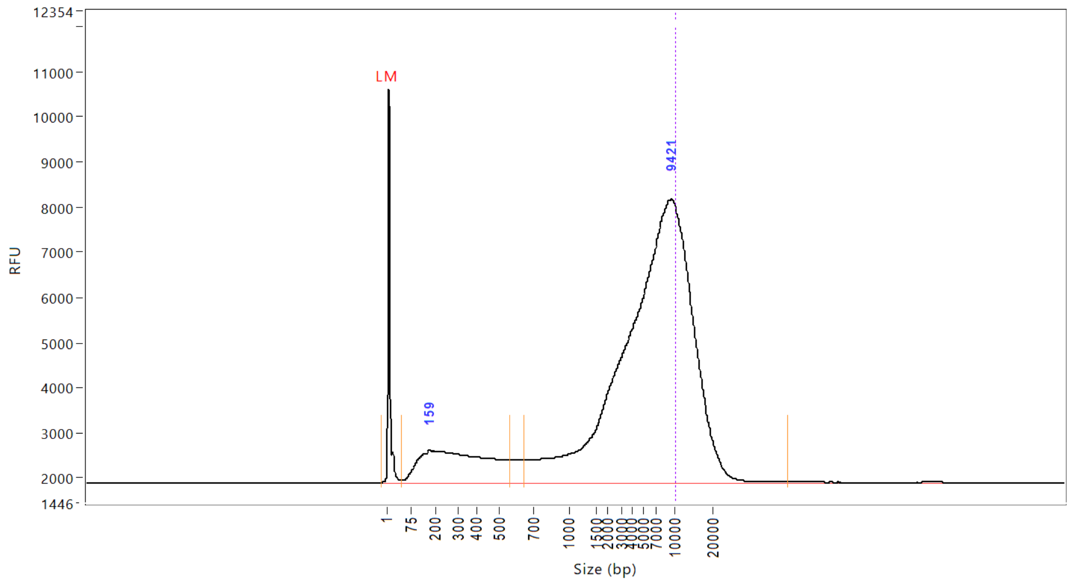

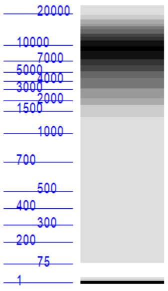

Peak	Size (bp)	Conc. (ng/uL)	From (bp)	To (bp)	Avg. Size (bp)	CV%	RFU	Corr. Peak Area
1	1 (LM)	0.0328	0	45	3	282.44	8718	60.522
2	159	1.0341	45	560	289	46.89	722	159.000
3	9421	6.1431	643	39500	7365	68.17	6297	944.511

TIC:	7.1773	ng/uL
TIM:	7.2591	nmole/L
Total Conc.:	7.3029	ng/uL
GQN:	2.3	

Sample Peak Width (sec): 50   Sample Min Peak Height: 50   Sample Baseline V to V?: Y   Sample Baseline V to V pts: 3
Sample Filter: Binomial   # of Pts for Filter: 3   Sample Start Region (min): 0   Sample End Region (min): 50
Manual Baseline Start (min): 6   Manual Baseline End (min): 48
Marker Peak Width (sec): 5   Marker Min Peak Height: 200   Marker Baseline V to V?: Y   Marker Baseline V to V pts: 3
Lower Marker Selection: First Peak > 200 RFU   Upper Marker Selection: Last Peak > 200 RFU
Ladder Size (bp): 1, 75, 200, 300, 400, 500, 700, 1000, 1500, 2000, 3000, 4000, 5000, 7000, 10000, 20000
Quantification Using: Ladder   Final Concentration (ng/uL): 1.0417   Dilution Factor: 12.0
Size Threshold (b.p.): 10000

Copyright 2015 Advanced Analytical Technologies, Inc.

Data File: 2019 06 18 13H 16M.raw
Sample: 103613-001-037 (20x dil.)
Well Location: E5

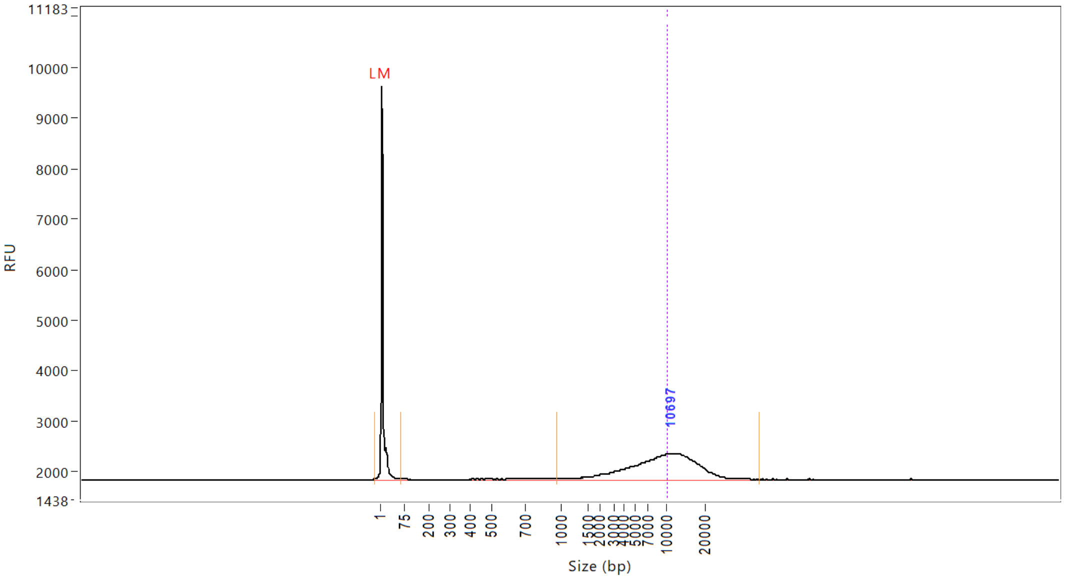

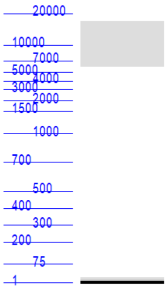

Peak	Size (bp)	Conc. (ng/uL)	From (bp)	To (bp)	Avg. Size (bp)	CV%	RFU	Corr. Peak Area
1	1 (LM)	0.0328	0	62	3	270.74	7783	54.599
2	10697	0.5855	966	34582	10189	57.98	513	81.216

TIC:	0.5855	ng/uL
TIM:	0.0946	nmole/L
Total Conc.:	0.6478	ng/uL
GQN:	4.5	

```
Sample Peak Width (sec): 50   Sample Min Peak Height: 50    Sample Baseline V to V?: Y    Sample Baseline V to V pts: 3
Sample Filter: Binomial       # of Pts for Filter: 3        Sample Start Region (min): 0  Sample End Region (min): 50
Manual Baseline Start (min): 6     Manual Baseline End (min): 48
Marker Peak Width (sec): 5    Marker Min Peak Height: 200   Marker Baseline V to V?: Y    Marker Baseline V to V pts: 3
Lower Marker Selection: First Peak > 200 RFU                Upper Marker Selection: Last Peak > 200 RFU
Ladder Size (bp): 1, 75, 200, 300, 400, 500, 700, 1000, 1500, 2000, 3000, 4000, 5000, 7000, 10000, 20000
Quantification Using: Ladder          Final Concentration (ng/uL): 1.0417          Dilution Factor: 12.0
Size Threshold (b.p.): 10000
```

 Copyright 2015 Advanced Analytical Technologies, Inc.

Data File: 2019 06 18 13H 16M.raw
Sample: 103613-001-038 (20x dil.)
Well Location: F5

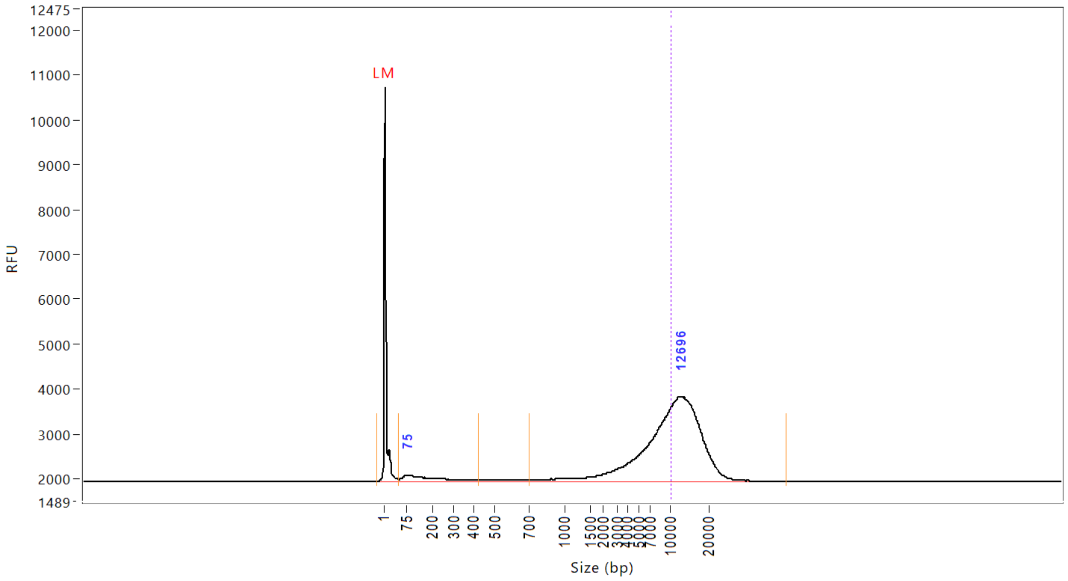

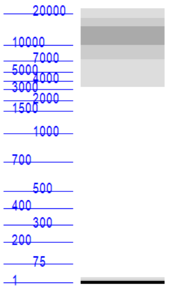

Peak	Size (bp)	Conc. (ng/uL)	From (bp)	To (bp)	Avg. Size (bp)	CV%	RFU	Corr. Peak Area
1	1 (LM)	0.0328	0	46	3	258.08	8782	62.008
2	75	0.0939	46	422	175	58.71	140	14.791
3	12696	1.5318	700	40450	10907	50.53	1881	241.300

TIC:	1.6257	ng/uL
TIM:	1.1132	nmole/L
Total Conc.:	1.6615	ng/uL
GQN:	4.9	

Sample Peak Width (sec): 50   Sample Min Peak Height: 50   Sample Baseline V to V?: Y   Sample Baseline V to V pts: 3
Sample Filter: Binomial   # of Pts for Filter: 3   Sample Start Region (min): 0   Sample End Region (min): 50
Manual Baseline Start (min): 6   Manual Baseline End (min): 48
Marker Peak Width (sec): 5   Marker Min Peak Height: 200   Marker Baseline V to V?: Y   Marker Baseline V to V pts: 3
Lower Marker Selection: First Peak > 200 RFU   Upper Marker Selection: Last Peak > 200 RFU
Ladder Size (bp): 1, 75, 200, 300, 400, 500, 700, 1000, 1500, 2000, 3000, 4000, 5000, 7000, 10000, 20000
Quantification Using: Ladder   Final Concentration (ng/uL): 1.0417   Dilution Factor: 12.0
Size Threshold (b.p.): 10000

 Copyright 2015 Advanced Analytical Technologies, Inc.

Data File: 2019 06 18 13H 16M.raw
Sample: 103613-001-039 (20x dil.)
Well Location: G5

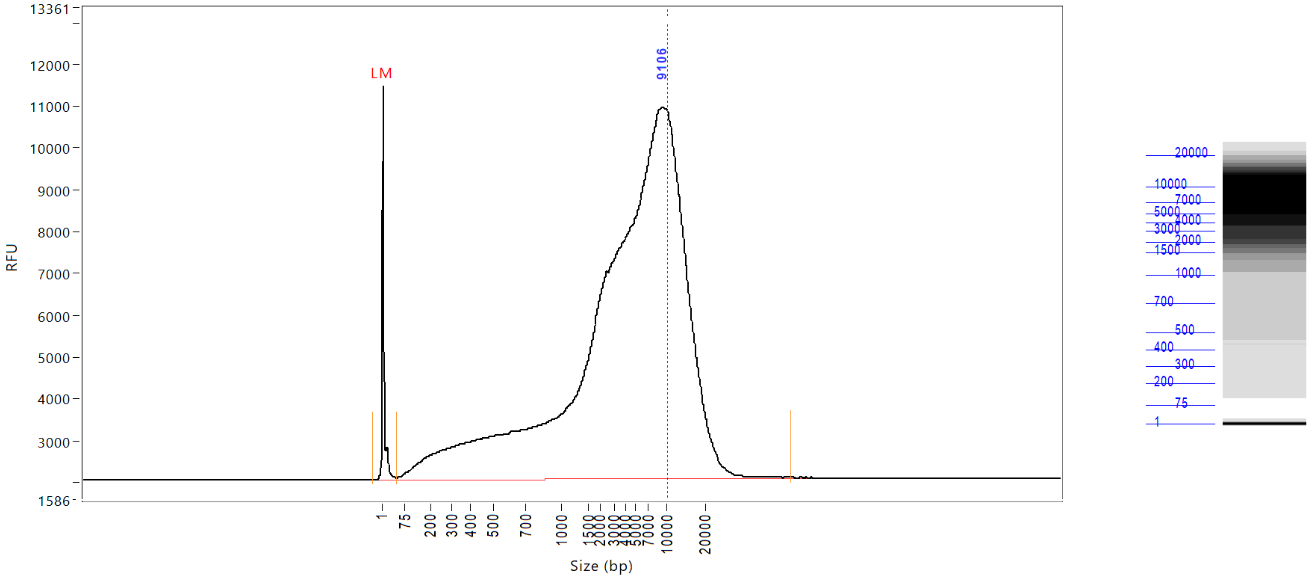

Peak	Size (bp)	Conc. (ng/uL)	From (bp)	To (bp)	Avg. Size (bp)	CV%	RFU	Corr. Peak Area
1	1 (LM)	0.0328	0	45	2	321.85	9403	65.200
2	9106	10.9912	45	42607	5914	90.91	8883	1820.521

TIC:	10.9912	ng/uL
TIM:	3.0596	nmole/L
Total Conc.:	11.0192	ng/uL
GQN:	2.1	

Sample Peak Width (sec): 50 Sample Min Peak Height: 50 Sample Baseline V to V?: Y Sample Baseline V to V pts: 3
Sample Filter: Binomial # of Pts for Filter: 3 Sample Start Region (min): 0 Sample End Region (min): 50
Manual Baseline Start (min): 6 Manual Baseline End (min): 48
Marker Peak Width (sec): 5 Marker Min Peak Height: 200 Marker Baseline V to V?: Y Marker Baseline V to V pts: 3
Lower Marker Selection: First Peak > 200 RFU Upper Marker Selection: Last Peak > 200 RFU
Ladder Size (bp): 1, 75, 200, 300, 400, 500, 700, 1000, 1500, 2000, 3000, 4000, 5000, 7000, 10000, 20000
Quantification Using: Ladder Final Concentration (ng/uL): 1.0417 Dilution Factor: 12.0
Size Threshold (b.p.): 10000

 Copyright 2015 Advanced Analytical Technologies, Inc.

Data File: 2019 06 18 13H 16M.raw
Sample: 103613-001-040 (20x dil.)
Well Location: H5

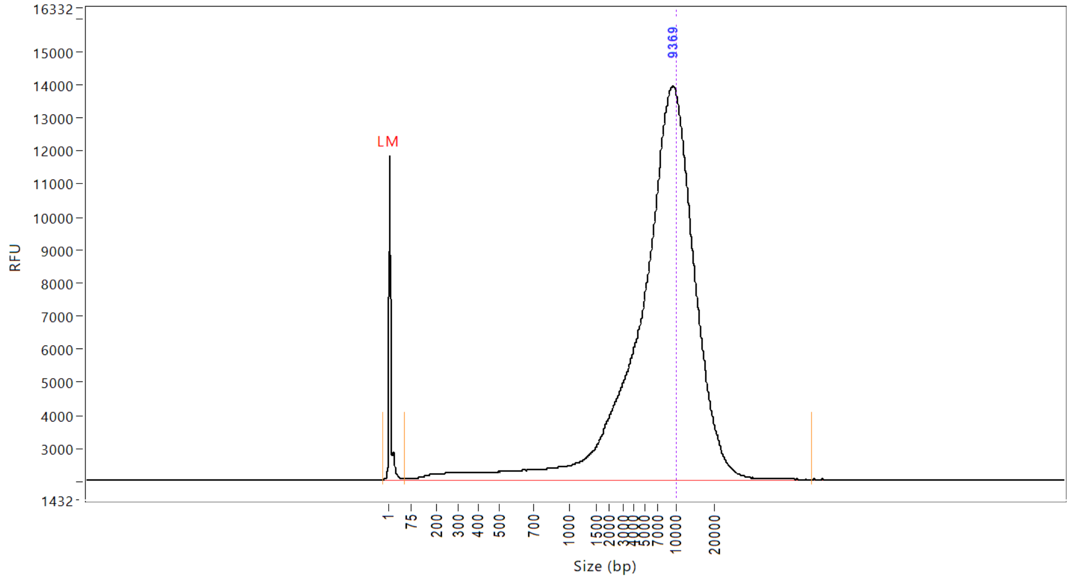

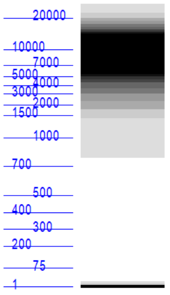

Peak	Size (bp)	Conc. (ng/uL)	From (bp)	To (bp)	Avg. Size (bp)	CV%	RFU	Corr. Peak Area
1	1 (LM)	0.0328	0	49	3	274.98	9799	68.313
2	9369	8.4587	49	45454	8205	62.76	11913	1467.936

TIC:	8.4587	ng/uL
TIM:	1.6972	nmole/L
Total Conc.:	8.4827	ng/uL
GQN:	3.2	

```
Sample Peak Width (sec): 50   Sample Min Peak Height: 50    Sample Baseline V to V?: Y    Sample Baseline V to V pts: 3
Sample Filter: Binomial       # of Pts for Filter: 3        Sample Start Region (min): 0  Sample End Region (min): 50
Manual Baseline Start (min): 6     Manual Baseline End (min): 48
Marker Peak Width (sec): 5    Marker Min Peak Height: 200   Marker Baseline V to V?: Y    Marker Baseline V to V pts: 3
Lower Marker Selection: First Peak > 200 RFU                Upper Marker Selection: Last Peak > 200 RFU
Ladder Size (bp): 1, 75, 200, 300, 400, 500, 700, 1000, 1500, 2000, 3000, 4000, 5000, 7000, 10000, 20000
Quantification Using: Ladder          Final Concentration (ng/uL): 1.0417          Dilution Factor: 12.0
Size Threshold (b.p.): 10000
```

 Copyright 2015 Advanced Analytical Technologies, Inc.

Data File: 2019 06 18 13H 16M.raw
Sample: 103613-001-041 (20x dil.)
Well Location: A6

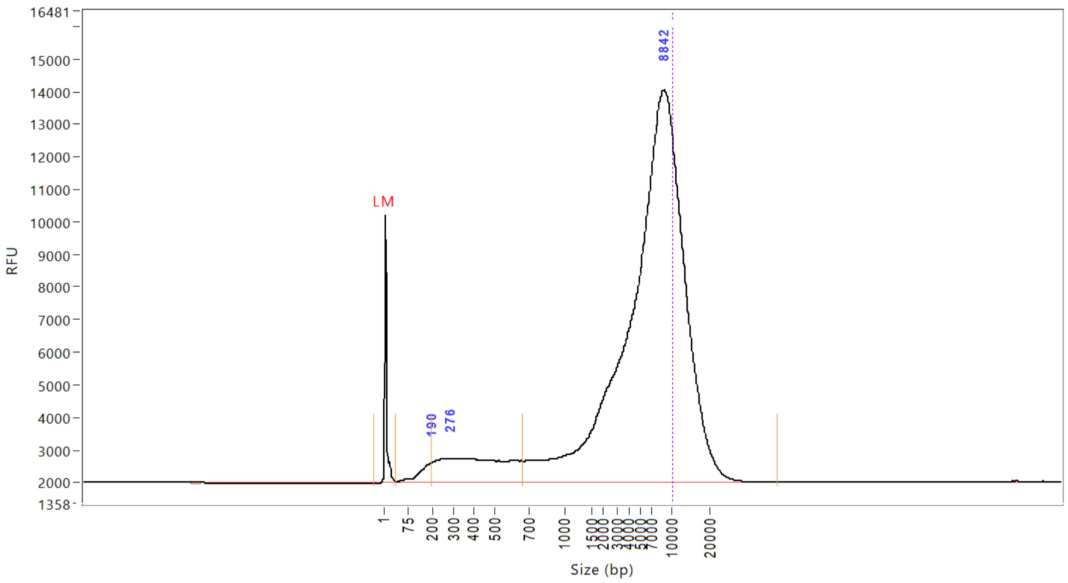

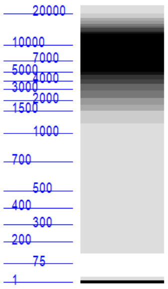

Peak	Size (bp)	Conc. (ng/uL)	From (bp)	To (bp)	Avg. Size (bp)	CV%	RFU	Corr. Peak Area
1	1 (LM)	0.0328	0	35	2	280.15	8174	53.964
2	190	0.1712	35	192	145	25.40	588	23.469
3	276	1.1303	192	657	400	32.78	717	154.948
4	8842	10.3208	657	37602	7423	60.51	12036	1414.884

TIC:	11.6222	ng/uL
TIM:	8.8783	nmole/L
Total Conc.:	11.6176	ng/uL
GQN:	2.2	

Sample Peak Width (sec): 50 Sample Min Peak Height: 50 Sample Baseline V to V?: Y Sample Baseline V to V pts: 3
Sample Filter: Binomial # of Pts for Filter: 3 Sample Start Region (min): 0 Sample End Region (min): 50
Manual Baseline Start (min): 6 Manual Baseline End (min): 48
Marker Peak Width (sec): 5 Marker Min Peak Height: 200 Marker Baseline V to V?: Y Marker Baseline V to V pts: 3
Lower Marker Selection: First Peak > 200 RFU Upper Marker Selection: Last Peak > 200 RFU
Ladder Size (bp): 1, 75, 200, 300, 400, 500, 700, 1000, 1500, 2000, 3000, 4000, 5000, 7000, 10000, 20000
Quantification Using: Ladder Final Concentration (ng/uL): 1.0417 Dilution Factor: 12.0
Size Threshold (b.p.): 10000

 Copyright 2015 Advanced Analytical Technologies, Inc.

Data File: 2019 06 18 13H 16M.raw
Sample: 103613-001-042 (20x dil.)
Well Location: B6

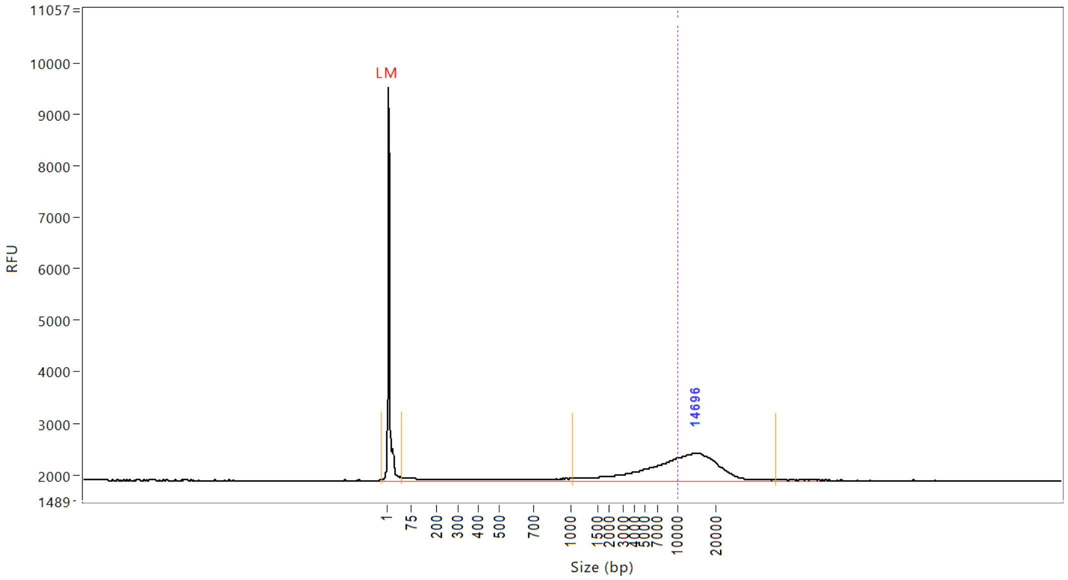

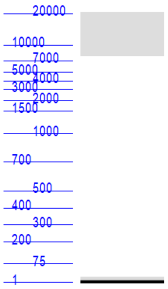

Peak	Size (bp)	Conc. (ng/uL)	From (bp)	To (bp)	Avg. Size (bp)	CV%	RFU	Corr. Peak Area
1	1 (LM)	0.0328	0	45	3	272.13	7638	53.675
2	14696	0.6437	1020	35790	11614	55.76	532	87.769

TIC:	0.6437	ng/uL
TIM:	0.0912	nmole/L
Total Conc.:	0.7446	ng/uL
GQN:	5.0	

Sample Peak Width (sec): 50 Sample Min Peak Height: 50 Sample Baseline V to V?: Y Sample Baseline V to V pts: 3
Sample Filter: Binomial # of Pts for Filter: 3 Sample Start Region (min): 0 Sample End Region (min): 50
Manual Baseline Start (min): 6 Manual Baseline End (min): 48
Marker Peak Width (sec): 5 Marker Min Peak Height: 200 Marker Baseline V to V?: Y Marker Baseline V to V pts: 3
Lower Marker Selection: First Peak > 200 RFU Upper Marker Selection: Last Peak > 200 RFU
Ladder Size (bp): 1, 75, 200, 300, 400, 500, 700, 1000, 1500, 2000, 3000, 4000, 5000, 7000, 10000, 20000
Quantification Using: Ladder Final Concentration (ng/uL): 1.0417 Dilution Factor: 12.0
Size Threshold (b.p.): 10000

 Copyright 2015 Advanced Analytical Technologies, Inc.

Data File: 2019 06 18 13H 16M.raw
Sample: 103613-001-043 (20x dil.)
Well Location: C6

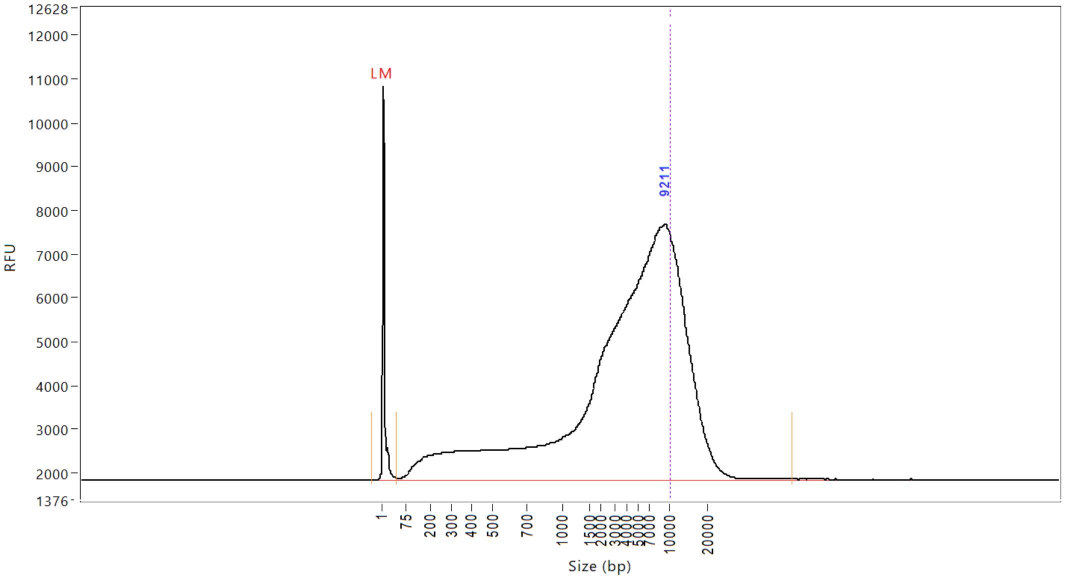

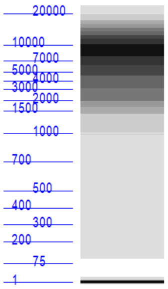

Peak	Size (bp)	Conc. (ng/uL)	From (bp)	To (bp)	Avg. Size (bp)	CV%	RFU	Corr. Peak Area
1	1 (LM)	0.0328	0	46	3	259.35	8995	63.234
2	9211	7.4777	46	42262	5643	91.65	5841	1201.227

TIC:	7.4777	ng/uL
TIM:	2.1816	nmole/L
Total Conc.:	7.5011	ng/uL
GQN:	1.9	

Sample Peak Width (sec): 50 Sample Min Peak Height: 50 Sample Baseline V to V?: Y Sample Baseline V to V pts: 3
Sample Filter: Binomial # of Pts for Filter: 3 Sample Start Region (min): 0 Sample End Region (min): 50
Manual Baseline Start (min): 6 Manual Baseline End (min): 48
Marker Peak Width (sec): 5 Marker Min Peak Height: 200 Marker Baseline V to V?: Y Marker Baseline V to V pts: 3
Lower Marker Selection: First Peak > 200 RFU Upper Marker Selection: Last Peak > 200 RFU
Ladder Size (bp): 1, 75, 200, 300, 400, 500, 700, 1000, 1500, 2000, 3000, 4000, 5000, 7000, 10000, 20000
Quantification Using: Ladder Final Concentration (ng/uL): 1.0417 Dilution Factor: 12.0
Size Threshold (b.p.): 10000

 Copyright 2015 Advanced Analytical Technologies, Inc.

Data File: 2019 06 18 13H 16M.raw
Sample: 103613-001-044 (20x dil.)
Well Location: D6

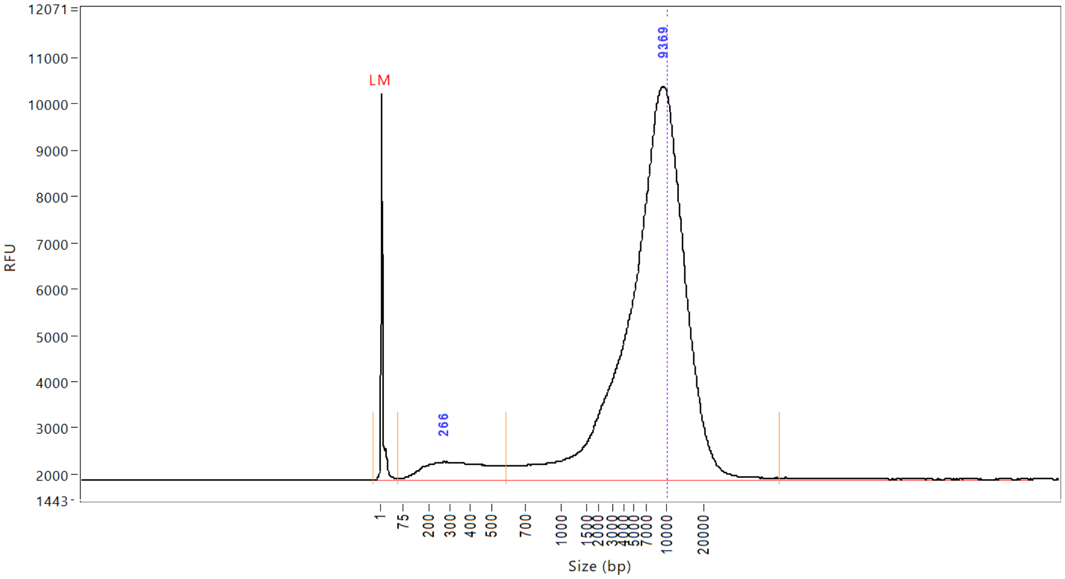

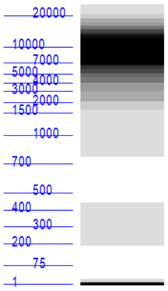

Peak	Size (bp)	Conc. (ng/uL)	From (bp)	To (bp)	Avg. Size (bp)	CV%	RFU	Corr. Peak Area
1	1 (LM)	0.0328	0	54	3	262.68	8332	58.386
2	266	0.5573	54	587	333	39.28	385	82.665
3	9369	7.0463	587	39759	8408	60.15	8489	1045.140

TIC:	7.6036	ng/uL
TIM:	4.1329	nmole/L
Total Conc.:	7.6465	ng/uL
GQN:	3.1	

Sample Peak Width (sec): 50   Sample Min Peak Height: 50   Sample Baseline V to V?: Y   Sample Baseline V to V pts: 3
Sample Filter: Binomial   # of Pts for Filter: 3   Sample Start Region (min): 0   Sample End Region (min): 50
Manual Baseline Start (min): 6   Manual Baseline End (min): 48
Marker Peak Width (sec): 5   Marker Min Peak Height: 200   Marker Baseline V to V?: Y   Marker Baseline V to V pts: 3
Lower Marker Selection: First Peak > 200 RFU   Upper Marker Selection: Last Peak > 200 RFU
Ladder Size (bp): 1, 75, 200, 300, 400, 500, 700, 1000, 1500, 2000, 3000, 4000, 5000, 7000, 10000, 20000
Quantification Using: Ladder   Final Concentration (ng/uL): 1.0417   Dilution Factor: 12.0
Size Threshold (b.p.): 10000

 Copyright 2015 Advanced Analytical Technologies, Inc.

Data File: 2019 06 18 13H 16M.raw
Sample: 103613-001-045 (20x dil.)
Well Location: E6

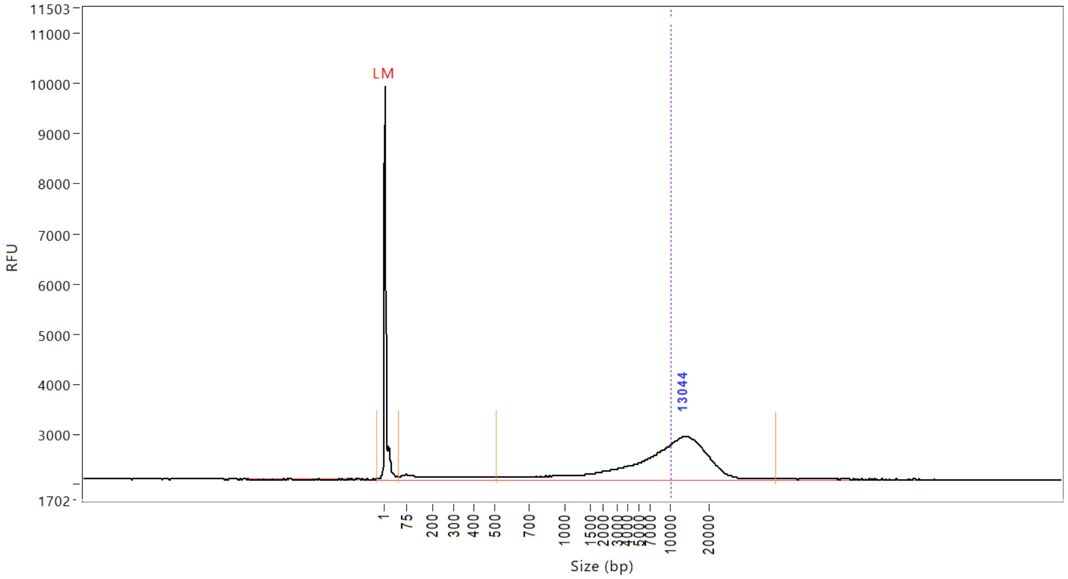

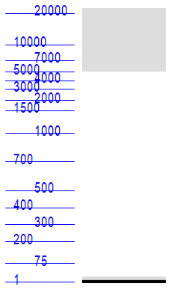

Peak	Size (bp)	Conc. (ng/uL)	From (bp)	To (bp)	Avg. Size (bp)	CV%	RFU	Corr. Peak Area
1	1 (LM)	0.0328	0	46	3	250.98	7822	55.407
2	13044	1.0278	503	37947	10348	65.67	843	144.667

TIC:	1.0278	ng/uL
TIM:	0.1635	nmole/L
Total Conc.:	1.1634	ng/uL
GQN:	4.5	

Sample Peak Width (sec): 50 Sample Min Peak Height: 50 Sample Baseline V to V?: Y Sample Baseline V to V pts: 3
Sample Filter: Binomial # of Pts for Filter: 3 Sample Start Region (min): 0 Sample End Region (min): 50
Manual Baseline Start (min): 6 Manual Baseline End (min): 48
Marker Peak Width (sec): 5 Marker Min Peak Height: 200 Marker Baseline V to V?: Y Marker Baseline V to V pts: 3
Lower Marker Selection: First Peak > 200 RFU Upper Marker Selection: Last Peak > 200 RFU
Ladder Size (bp): 1, 75, 200, 300, 400, 500, 700, 1000, 1500, 2000, 3000, 4000, 5000, 7000, 10000, 20000
Quantification Using: Ladder Final Concentration (ng/uL): 1.0417 Dilution Factor: 12.0
Size Threshold (b.p.): 10000

 Copyright 2015 Advanced Analytical Technologies, Inc.

Data File: 2019 06 18 13H 16M.raw
Sample: 103613-001-046 (20x dil.)
Well Location: F6

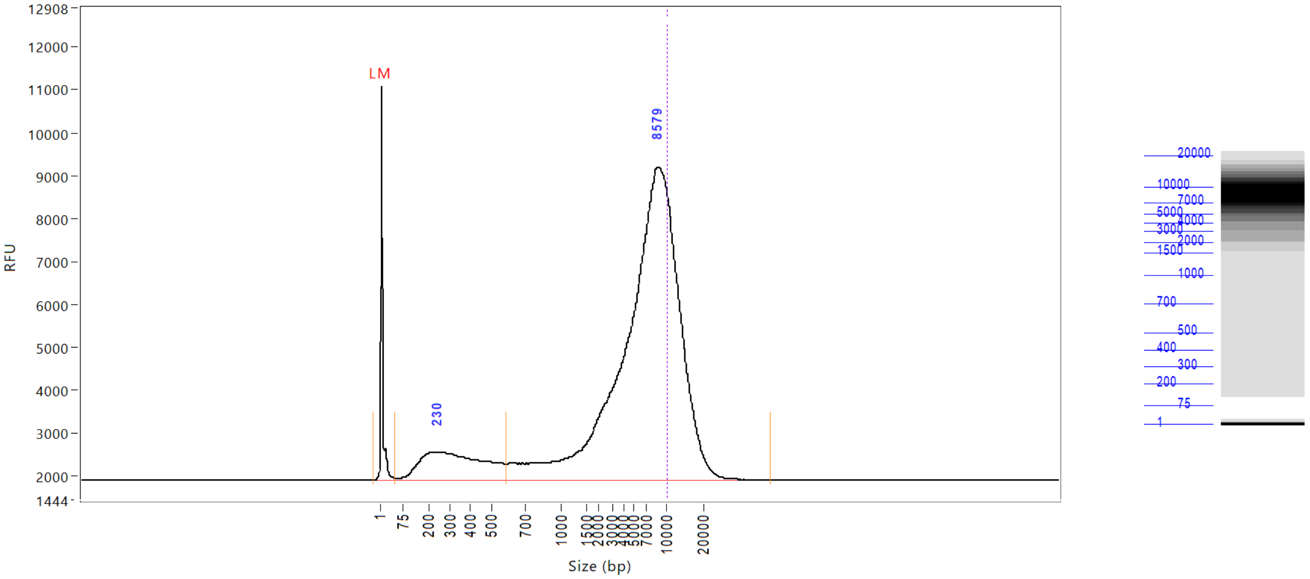

Peak	Size (bp)	Conc. (ng/uL)	From (bp)	To (bp)	Avg. Size (bp)	CV%	RFU	Corr. Peak Area
1	1 (LM)	0.0328	0	46	2	303.26	9165	63.509
2	230	0.8117	46	587	315	41.49	643	130.953
3	8579	5.4094	587	37688	7439	60.99	7288	872.752

TIC:	6.2211	ng/uL
TIM:	5.4358	nmole/L
Total Conc.:	6.2318	ng/uL
GQN:	2.2	

```
Sample Peak Width (sec): 50   Sample Min Peak Height: 50    Sample Baseline V to V?: Y    Sample Baseline V to V pts: 3
Sample Filter: Binomial       # of Pts for Filter: 3        Sample Start Region (min): 0  Sample End Region (min): 50
Manual Baseline Start (min): 6     Manual Baseline End (min): 48
Marker Peak Width (sec): 5    Marker Min Peak Height: 200   Marker Baseline V to V?: Y    Marker Baseline V to V pts: 3
Lower Marker Selection: First Peak > 200 RFU                Upper Marker Selection: Last Peak > 200 RFU
Ladder Size (bp): 1, 75, 200, 300, 400, 500, 700, 1000, 1500, 2000, 3000, 4000, 5000, 7000, 10000, 20000
Quantification Using: Ladder         Final Concentration (ng/uL): 1.0417         Dilution Factor: 12.0
Size Threshold (b.p.): 10000
```


Copyright 2015 Advanced Analytical Technologies, Inc.

Data File: 2019 06 18 13H 16M.raw
Sample: 103613-001-047 (20x dil.)
Well Location: G6

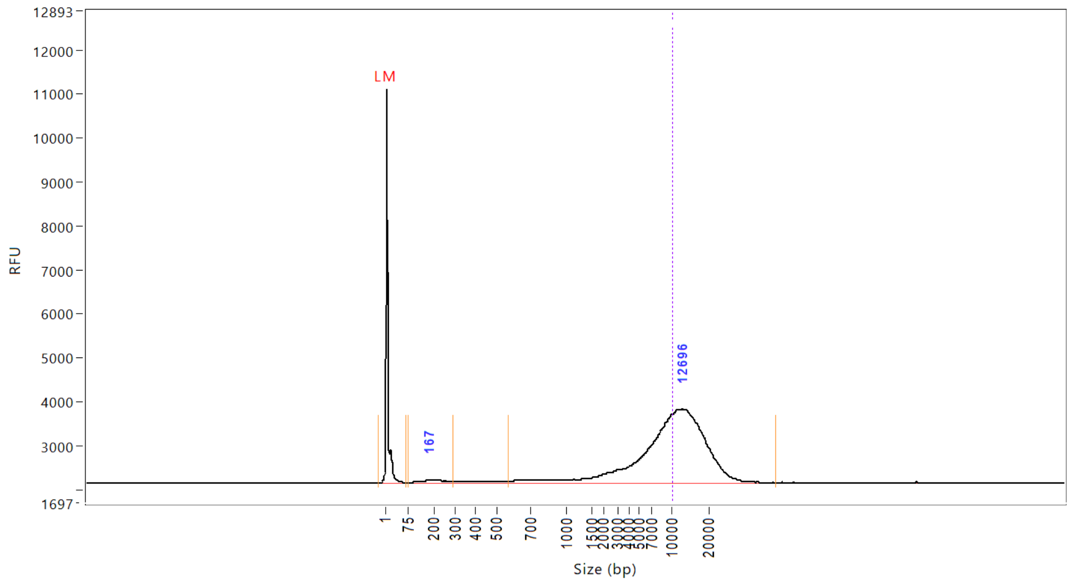

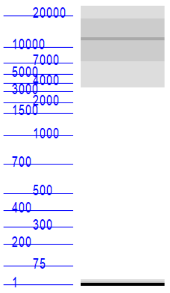

Peak	Size (bp)	Conc. (ng/uL)	From (bp)	To (bp)	Avg. Size (bp)	CV%	RFU	Corr. Peak Area
1	1 (LM)	0.0328	0	62	2	279.10	8944	62.212
2	167	0.0306	71	283	189	27.73	72	4.829
3	12696	1.5506	563	37775	10961	54.60	1666	245.065

TIC:	1.5812	ng/uL
TIM:	0.4987	nmole/L
Total Conc.:	1.6183	ng/uL
GQN:	5.1	

Sample Peak Width (sec): 50 Sample Min Peak Height: 50 Sample Baseline V to V?: Y Sample Baseline V to V pts: 3
Sample Filter: Binomial # of Pts for Filter: 3 Sample Start Region (min): 0 Sample End Region (min): 50
Manual Baseline Start (min): 6 Manual Baseline End (min): 48
Marker Peak Width (sec): 5 Marker Min Peak Height: 200 Marker Baseline V to V?: Y Marker Baseline V to V pts: 3
Lower Marker Selection: First Peak > 200 RFU Upper Marker Selection: Last Peak > 200 RFU
Ladder Size (bp): 1, 75, 200, 300, 400, 500, 700, 1000, 1500, 2000, 3000, 4000, 5000, 7000, 10000, 20000
Quantification Using: Ladder Final Concentration (ng/uL): 1.0417 Dilution Factor: 12.0
Size Threshold (b.p.): 10000

 Copyright 2015 Advanced Analytical Technologies, Inc.

Data File: 2019 06 18 13H 16M.raw
Sample: 103613-001-048 (20x dil.)
Well Location: H6

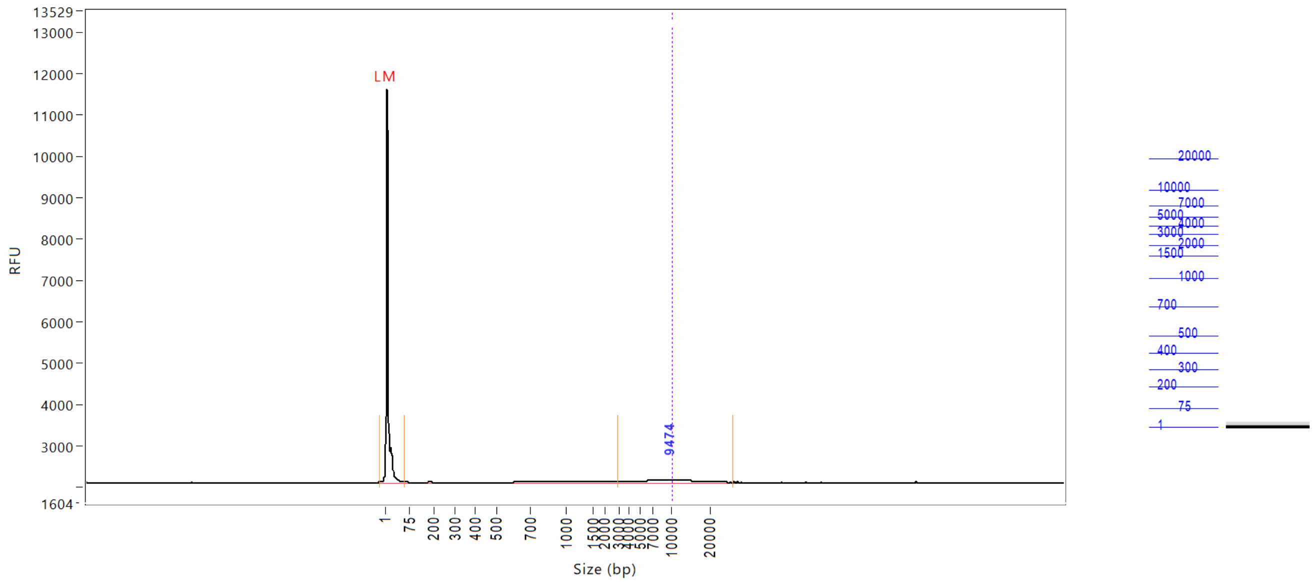

Peak	Size (bp)	Conc. (ng/uL)	From (bp)	To (bp)	Avg. Size (bp)	CV%	RFU	Corr. Peak Area
1	1 (LM)	0.0328	0	57	3	241.17	9522	68.529
2	9474	0.0508	2881	26299	10538	49.46	70	8.851

TIC:	0.0508	ng/uL
TIM:	0.0079	nmole/L
Total Conc.:	0.1010	ng/uL
GQN:	3.8	

```
Sample Peak Width (sec): 50   Sample Min Peak Height: 50    Sample Baseline V to V?: Y    Sample Baseline V to V pts: 3
Sample Filter: Binomial       # of Pts for Filter: 3        Sample Start Region (min): 0  Sample End Region (min): 50
Manual Baseline Start (min): 6     Manual Baseline End (min): 48
Marker Peak Width (sec): 5    Marker Min Peak Height: 200   Marker Baseline V to V?: Y    Marker Baseline V to V pts: 3
Lower Marker Selection: First Peak > 200 RFU                Upper Marker Selection: Last Peak > 200 RFU
Ladder Size (bp): 1, 75, 200, 300, 400, 500, 700, 1000, 1500, 2000, 3000, 4000, 5000, 7000, 10000, 20000
Quantification Using: Ladder          Final Concentration (ng/uL): 1.0417          Dilution Factor: 12.0
Size Threshold (b.p.): 10000
```


Copyright 2015 Advanced Analytical Technologies, Inc.

Data File: 2019 06 18 13H 16M.raw
Sample: 103613-001-049 (20x dil.)
Well Location: A7

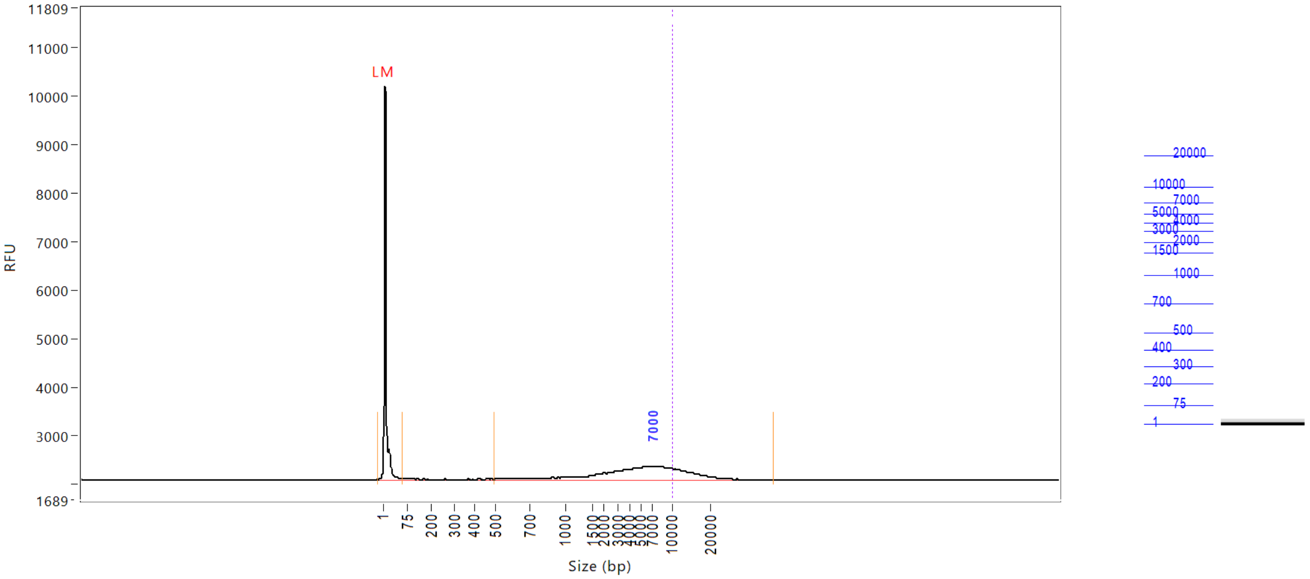

Peak	Size (bp)	Conc. (ng/uL)	From (bp)	To (bp)	Avg. Size (bp)	CV%	RFU	Corr. Peak Area
1	1 (LM)	0.0328	0	57	3	261.15	8084	57.246
2	7000	0.3474	492	36567	6479	81.68	261	50.522

TIC:	0.3474	ng/uL
TIM:	0.0883	nmole/L
Total Conc.:	0.3670	ng/uL
GQN:	2.0	

Sample Peak Width (sec): 50   Sample Min Peak Height: 50   Sample Baseline V to V?: Y   Sample Baseline V to V pts: 3
Sample Filter: Binomial   # of Pts for Filter: 3   Sample Start Region (min): 0   Sample End Region (min): 50
Manual Baseline Start (min): 6   Manual Baseline End (min): 48
Marker Peak Width (sec): 5   Marker Min Peak Height: 200   Marker Baseline V to V?: Y   Marker Baseline V to V pts: 3
Lower Marker Selection: First Peak > 200 RFU   Upper Marker Selection: Last Peak > 200 RFU
Ladder Size (bp): 1, 75, 200, 300, 400, 500, 700, 1000, 1500, 2000, 3000, 4000, 5000, 7000, 10000, 20000
Quantification Using: Ladder   Final Concentration (ng/uL): 1.0417   Dilution Factor: 12.0
Size Threshold (b.p.): 10000

 Copyright 2015 Advanced Analytical Technologies, Inc.

Data File: 2019 06 18 13H 16M.raw
Sample: 103613-001-050 (20x dil.)
Well Location: B7

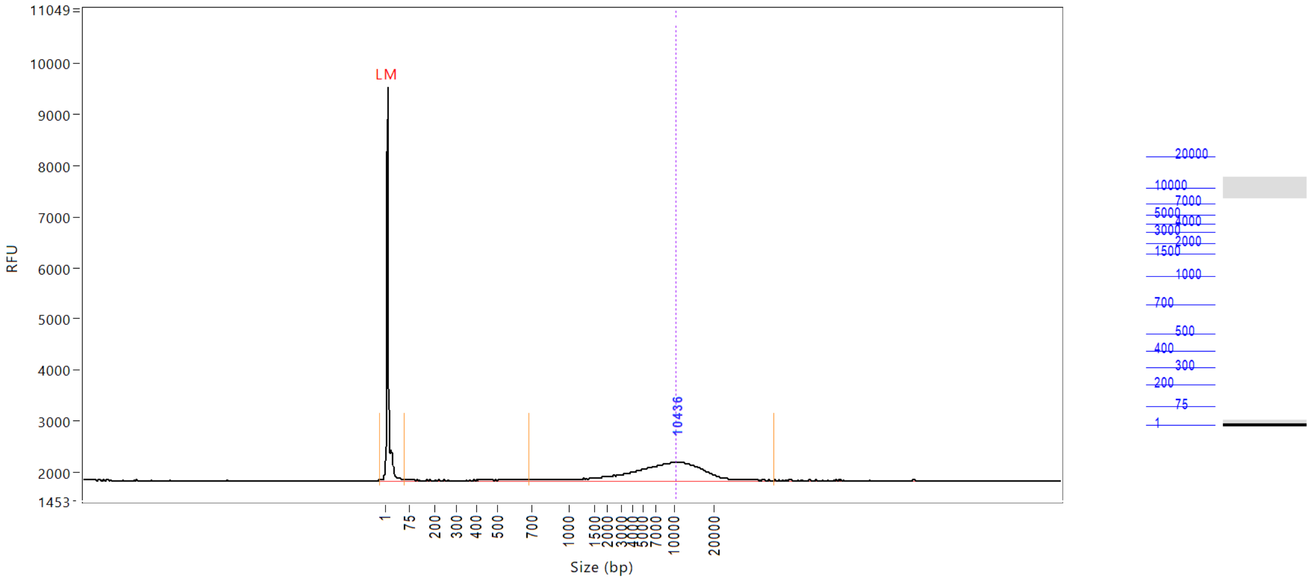

Peak	Size (bp)	Conc. (ng/uL)	From (bp)	To (bp)	Avg. Size (bp)	CV%	RFU	Corr. Peak Area
1	1 (LM)	0.0328	0	57	3	274.44	7663	53.430
2	10436	0.4405	679	35876	9239	67.15	367	59.796

TIC:	0.4405	ng/uL
TIM:	0.0785	nmole/L
Total Conc.:	0.5167	ng/uL
GQN:	4.1	

```
Sample Peak Width (sec): 50    Sample Min Peak Height: 50    Sample Baseline V to V?: Y    Sample Baseline V to V pts: 3
Sample Filter: Binomial        # of Pts for Filter: 3        Sample Start Region (min): 0  Sample End Region (min): 50
Manual Baseline Start (min): 6      Manual Baseline End (min): 48
Marker Peak Width (sec): 5     Marker Min Peak Height: 200   Marker Baseline V to V?: Y    Marker Baseline V to V pts: 3
Lower Marker Selection: First Peak > 200 RFU                 Upper Marker Selection: Last Peak > 200 RFU
Ladder Size (bp): 1, 75, 200, 300, 400, 500, 700, 1000, 1500, 2000, 3000, 4000, 5000, 7000, 10000, 20000
Quantification Using: Ladder          Final Concentration (ng/uL): 1.0417          Dilution Factor: 12.0
Size Threshold (b.p.): 10000
```

 Copyright 2015 Advanced Analytical Technologies, Inc.

Data File: 2019 06 18 13H 16M.raw
Sample: 103613-001-051 (20x dil.)
Well Location: C7

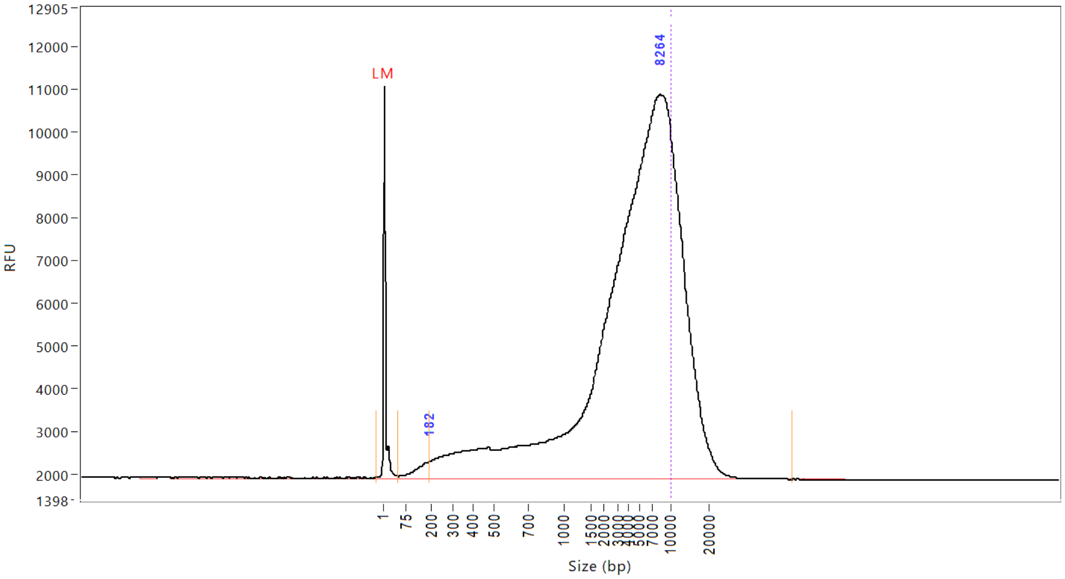

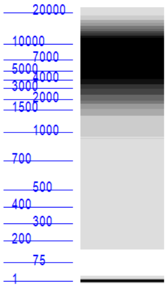

Peak	Size (bp)	Conc. (ng/uL)	From (bp)	To (bp)	Avg. Size (bp)	CV%	RFU	Corr. Peak Area
1	1 (LM)	0.0328	0	45	2	301.79	9159	63.777
2	182	0.1080	45	183	133	28.06	392	17.491
3	8264	9.2401	183	41571	5817	78.24	8986	1497.085

TIC:	9.3481	ng/uL
TIM:	3.9488	nmole/L
Total Conc.:	9.3473	ng/uL
GQN:	1.7	

```
Sample Peak Width (sec): 50   Sample Min Peak Height: 50    Sample Baseline V to V?: Y    Sample Baseline V to V pts: 3
Sample Filter: Binomial       # of Pts for Filter: 3        Sample Start Region (min): 0  Sample End Region (min): 50
Manual Baseline Start (min): 6     Manual Baseline End (min): 48
Marker Peak Width (sec): 5    Marker Min Peak Height: 200   Marker Baseline V to V?: Y    Marker Baseline V to V pts: 3
Lower Marker Selection: First Peak > 200 RFU                Upper Marker Selection: Last Peak > 200 RFU
Ladder Size (bp): 1, 75, 200, 300, 400, 500, 700, 1000, 1500, 2000, 3000, 4000, 5000, 7000, 10000, 20000
Quantification Using: Ladder          Final Concentration (ng/uL): 1.0417          Dilution Factor: 12.0
Size Threshold (b.p.): 10000
```

 Copyright 2015 Advanced Analytical Technologies, Inc.

Data File: 2019 06 18 13H 16M.raw
Sample: 103613-001-052 (20x dil.)
Well Location: D7

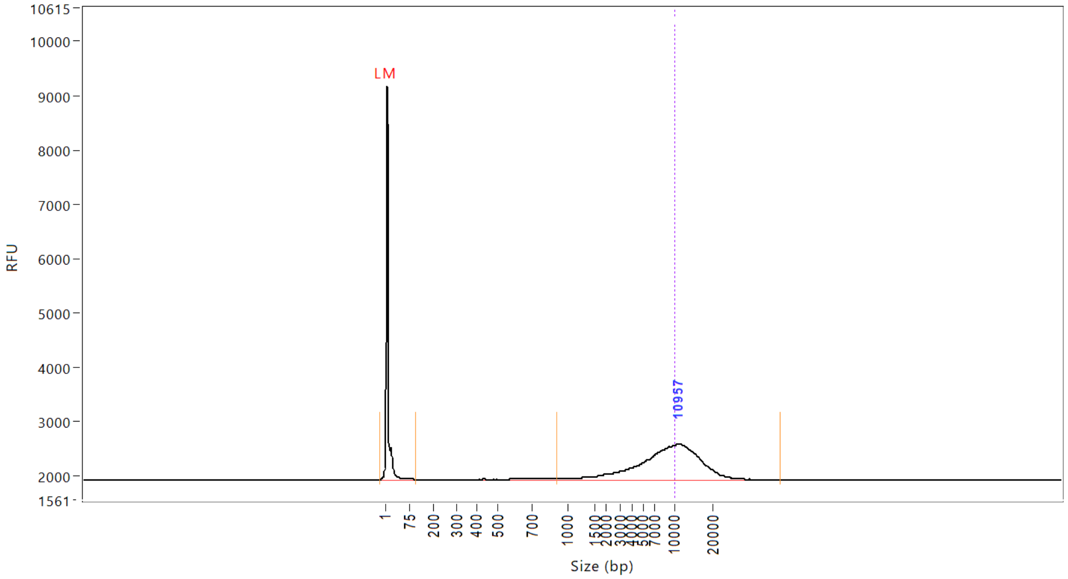

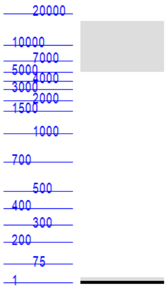

Peak	Size (bp)	Conc. (ng/uL)	From (bp)	To (bp)	Avg. Size (bp)	CV%	RFU	Corr. Peak Area
1	1 (LM)	0.0328	0	102	3	392.87	7238	52.168
2	10957	0.6769	902	37516	9670	57.10	656	89.713

TIC:	0.6769	ng/uL
TIM:	0.1152	nmole/L
Total Conc.:	0.7245	ng/uL
GQN:	4.0	

Sample Peak Width (sec): 50   Sample Min Peak Height: 50   Sample Baseline V to V?: Y   Sample Baseline V to V pts: 3
Sample Filter: Binomial   # of Pts for Filter: 3   Sample Start Region (min): 0   Sample End Region (min): 50
Manual Baseline Start (min): 6   Manual Baseline End (min): 48
Marker Peak Width (sec): 5   Marker Min Peak Height: 200   Marker Baseline V to V?: Y   Marker Baseline V to V pts: 3
Lower Marker Selection: First Peak > 200 RFU   Upper Marker Selection: Last Peak > 200 RFU
Ladder Size (bp): 1, 75, 200, 300, 400, 500, 700, 1000, 1500, 2000, 3000, 4000, 5000, 7000, 10000, 20000
Quantification Using: Ladder   Final Concentration (ng/uL): 1.0417   Dilution Factor: 12.0
Size Threshold (b.p.): 10000

 Copyright 2015 Advanced Analytical Technologies, Inc.

Data File: 2019 06 18 13H 16M.raw
Sample: 103613-001-053 (20x dil.)
Well Location: E7

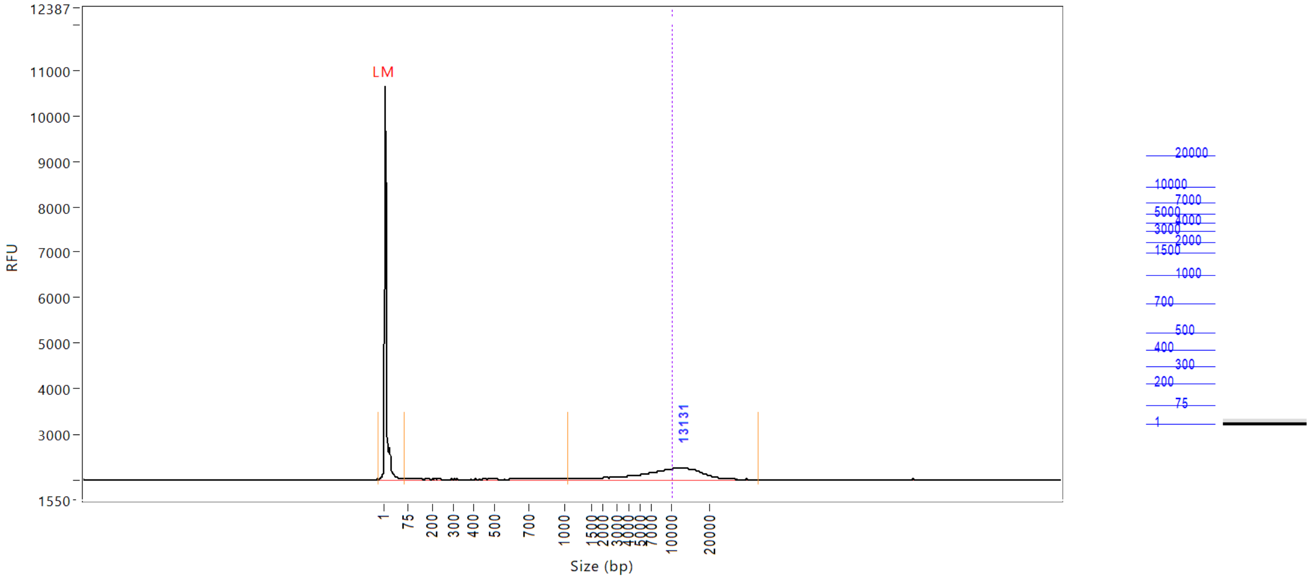

Peak	Size (bp)	Conc. (ng/uL)	From (bp)	To (bp)	Avg. Size (bp)	CV%	RFU	Corr. Peak Area
1	1 (LM)	0.0328	0	64	3	303.95	8653	60.176
2	13131	0.2364	1045	33115	10427	56.04	247	36.144

TIC:	0.2364	ng/uL
TIM:	0.0373	nmole/L
Total Conc.:	0.2938	ng/uL
GQN:	4.5	

Sample Peak Width (sec): 50 Sample Min Peak Height: 50 Sample Baseline V to V?: Y Sample Baseline V to V pts: 3
Sample Filter: Binomial # of Pts for Filter: 3 Sample Start Region (min): 0 Sample End Region (min): 50
Manual Baseline Start (min): 6 Manual Baseline End (min): 48
Marker Peak Width (sec): 5 Marker Min Peak Height: 200 Marker Baseline V to V?: Y Marker Baseline V to V pts: 3
Lower Marker Selection: First Peak > 200 RFU Upper Marker Selection: Last Peak > 200 RFU
Ladder Size (bp): 1, 75, 200, 300, 400, 500, 700, 1000, 1500, 2000, 3000, 4000, 5000, 7000, 10000, 20000
Quantification Using: Ladder Final Concentration (ng/uL): 1.0417 Dilution Factor: 12.0
Size Threshold (b.p.): 10000

 Copyright 2015 Advanced Analytical Technologies, Inc.

Data File: 2019 06 18 13H 16M.raw
Sample: 103613-001-054 (20x dil.)
Well Location: F7

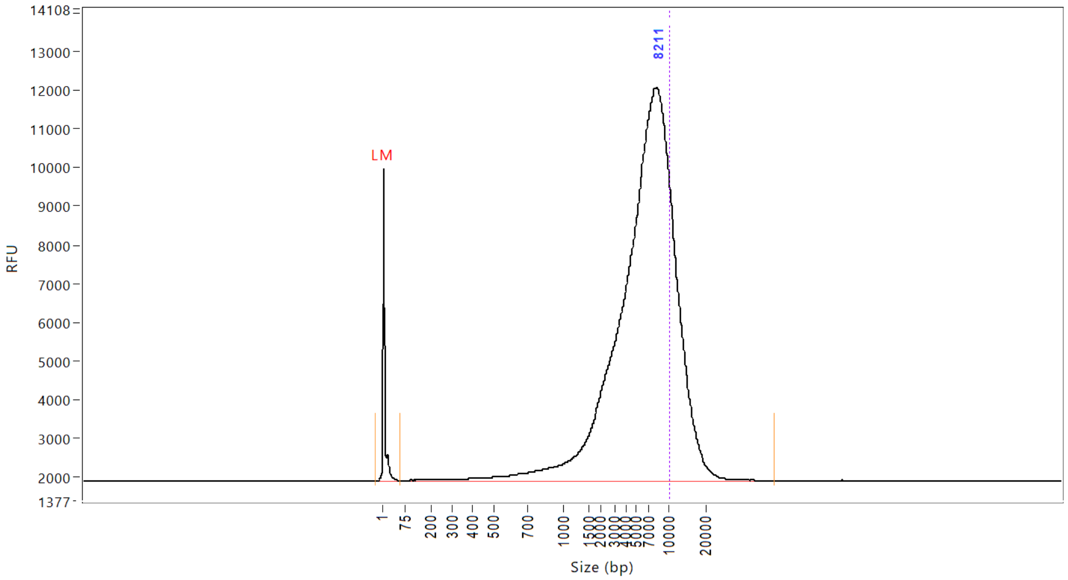

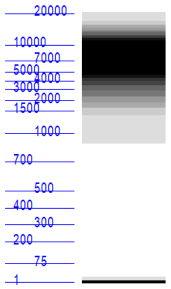

Peak	Size (bp)	Conc. (ng/uL)	From (bp)	To (bp)	Avg. Size (bp)	CV%	RFU	Corr. Peak Area
1	1 (LM)	0.0328	0	56	2	340.52	8095	56.639
2	8211	8.0243	56	38206	6816	59.27	10174	1154.586

TIC:	8.0243	ng/uL
TIM:	1.9382	nmole/L
Total Conc.:	8.0393	ng/uL
GQN:	1.8	

Sample Peak Width (sec): 50 Sample Min Peak Height: 50 Sample Baseline V to V?: Y Sample Baseline V to V pts: 3
Sample Filter: Binomial # of Pts for Filter: 3 Sample Start Region (min): 0 Sample End Region (min): 50
Manual Baseline Start (min): 6 Manual Baseline End (min): 48
Marker Peak Width (sec): 5 Marker Min Peak Height: 200 Marker Baseline V to V?: Y Marker Baseline V to V pts: 3
Lower Marker Selection: First Peak > 200 RFU Upper Marker Selection: Last Peak > 200 RFU
Ladder Size (bp): 1, 75, 200, 300, 400, 500, 700, 1000, 1500, 2000, 3000, 4000, 5000, 7000, 10000, 20000
Quantification Using: Ladder Final Concentration (ng/uL): 1.0417 Dilution Factor: 12.0
Size Threshold (b.p.): 10000

 Copyright 2015 Advanced Analytical Technologies, Inc.

Data File: 2019 06 18 13H 16M.raw
Sample: 103613-001-055 (20x dil.)
Well Location: G7

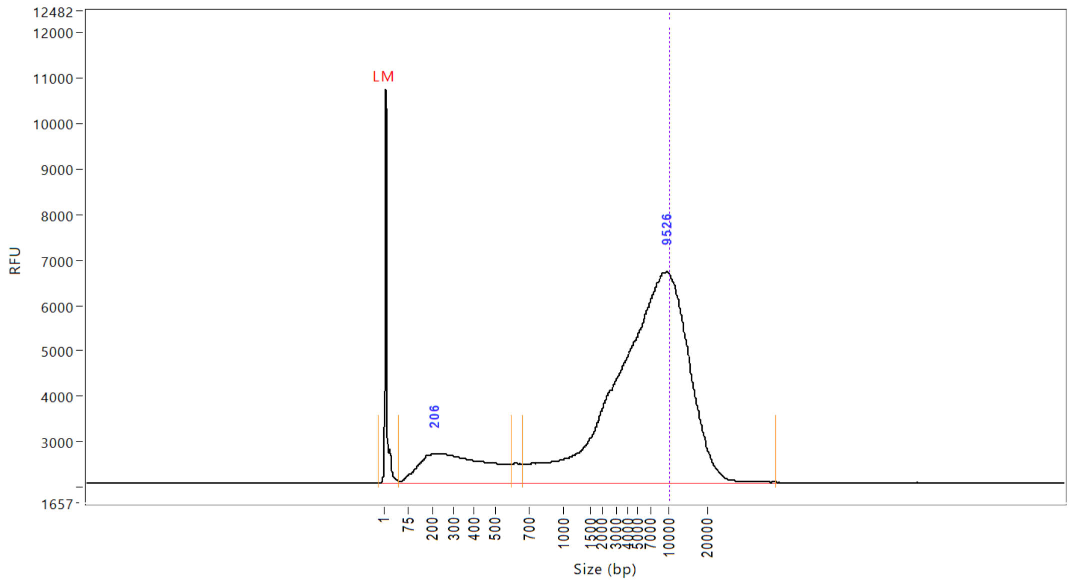

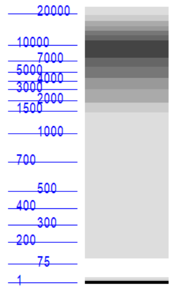

Peak	Size (bp)	Conc. (ng/uL)	From (bp)	To (bp)	Avg. Size (bp)	CV%	RFU	Corr. Peak Area
1	1 (LM)	0.0328	0	45	3	281.28	8652	60.785
2	206	0.8996	45	595	308	45.14	634	138.912
3	9526	4.8578	657	38120	7366	69.79	4642	750.131

TIC:	5.7574	ng/uL
TIM:	5.8902	nmole/L
Total Conc.:	5.8487	ng/uL
GQN:	2.3	

Sample Peak Width (sec): 50 Sample Min Peak Height: 50 Sample Baseline V to V?: Y Sample Baseline V to V pts: 3
Sample Filter: Binomial # of Pts for Filter: 3 Sample Start Region (min): 0 Sample End Region (min): 50
Manual Baseline Start (min): 6 Manual Baseline End (min): 48
Marker Peak Width (sec): 5 Marker Min Peak Height: 200 Marker Baseline V to V?: Y Marker Baseline V to V pts: 3
Lower Marker Selection: First Peak > 200 RFU Upper Marker Selection: Last Peak > 200 RFU
Ladder Size (bp): 1, 75, 200, 300, 400, 500, 700, 1000, 1500, 2000, 3000, 4000, 5000, 7000, 10000, 20000
Quantification Using: Ladder Final Concentration (ng/uL): 1.0417 Dilution Factor: 12.0
Size Threshold (b.p.): 10000

 Copyright 2015 Advanced Analytical Technologies, Inc.

Data File: 2019 06 18 13H 16M.raw
Sample: 103613-001-056 (20x dil.)
Well Location: H7

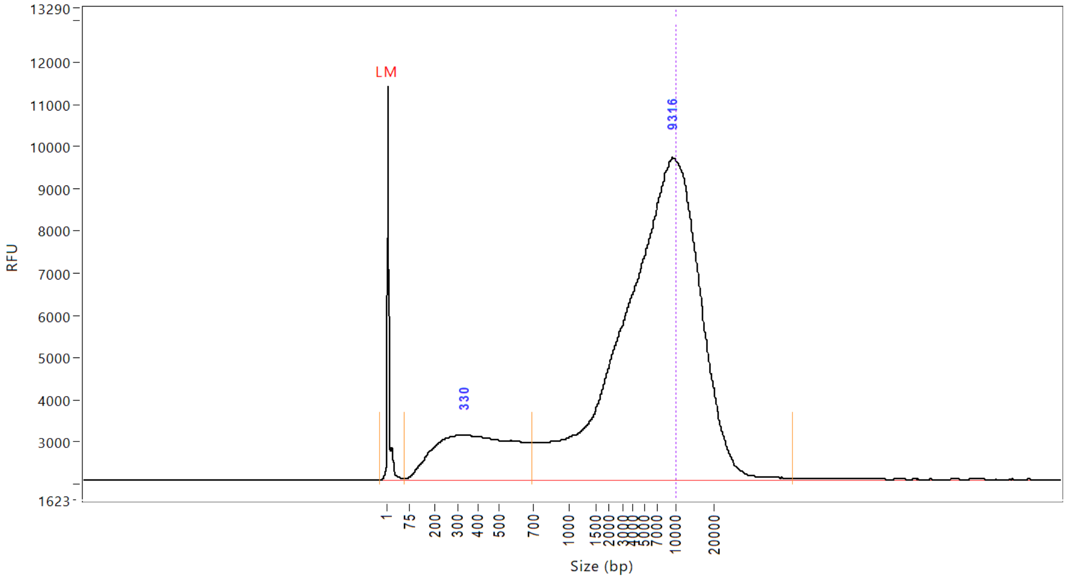

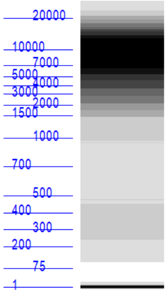

Peak	Size (bp)	Conc. (ng/uL)	From (bp)	To (bp)	Avg. Size (bp)	CV%	RFU	Corr. Peak Area
1	1 (LM)	0.0328	0	52	2	381.72	9325	66.014
2	330	1.5903	52	688	380	41.54	1044	266.704
3	9316	7.9449	688	40191	7851	72.04	7640	1332.394

TIC:	9.5352	ng/uL
TIM:	8.5514	nmole/L
Total Conc.:	9.5807	ng/uL
GQN:	2.5	

Sample Peak Width (sec): 50   Sample Min Peak Height: 50   Sample Baseline V to V?: Y   Sample Baseline V to V pts: 3
Sample Filter: Binomial   # of Pts for Filter: 3   Sample Start Region (min): 0   Sample End Region (min): 50
Manual Baseline Start (min): 6   Manual Baseline End (min): 48
Marker Peak Width (sec): 5   Marker Min Peak Height: 200   Marker Baseline V to V?: Y   Marker Baseline V to V pts: 3
Lower Marker Selection: First Peak > 200 RFU   Upper Marker Selection: Last Peak > 200 RFU
Ladder Size (bp): 1, 75, 200, 300, 400, 500, 700, 1000, 1500, 2000, 3000, 4000, 5000, 7000, 10000, 20000
Quantification Using: Ladder   Final Concentration (ng/uL): 1.0417   Dilution Factor: 12.0
Size Threshold (b.p.): 10000

 Copyright 2015 Advanced Analytical Technologies, Inc.

Data File: 2019 06 18 13H 16M.raw
Sample: 103613-001-057 (20x dil.)
Well Location: A8

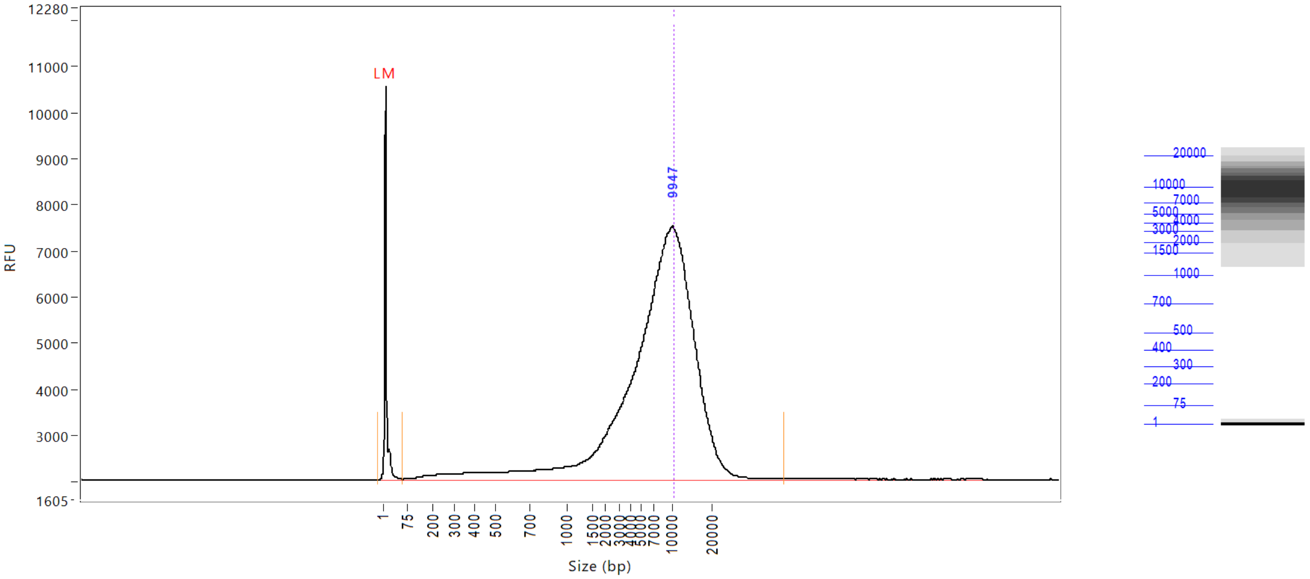

Peak	Size (bp)	Conc. (ng/uL)	From (bp)	To (bp)	Avg. Size (bp)	CV%	RFU	Corr. Peak Area
1	1 (LM)	0.0328	0	54	2	296.17	8530	58.585
2	9947	5.0735	54	38810	8187	64.92	5494	755.082

TIC:	5.0735	ng/uL
TIM:	1.0202	nmole/L
Total Conc.:	5.0992	ng/uL
GQN:	3.4	

Sample Peak Width (sec): 50 Sample Min Peak Height: 50 Sample Baseline V to V?: Y Sample Baseline V to V pts: 3
Sample Filter: Binomial # of Pts for Filter: 3 Sample Start Region (min): 0 Sample End Region (min): 50
Manual Baseline Start (min): 6 Manual Baseline End (min): 48
Marker Peak Width (sec): 5 Marker Min Peak Height: 200 Marker Baseline V to V?: Y Marker Baseline V to V pts: 3
Lower Marker Selection: First Peak > 200 RFU Upper Marker Selection: Last Peak > 200 RFU
Ladder Size (bp): 1, 75, 200, 300, 400, 500, 700, 1000, 1500, 2000, 3000, 4000, 5000, 7000, 10000, 20000
Quantification Using: Ladder Final Concentration (ng/uL): 1.0417 Dilution Factor: 12.0
Size Threshold (b.p.): 10000

 Copyright 2015 Advanced Analytical Technologies, Inc.

Data File: 2019 06 18 13H 16M.raw
Sample: 103613-001-058 (20x dil.)
Well Location: B8

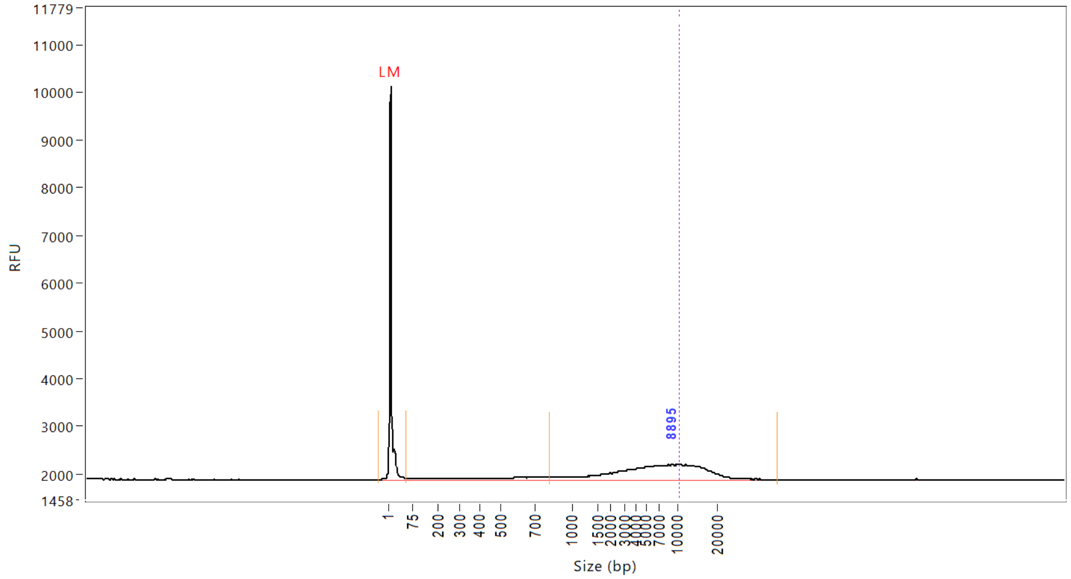

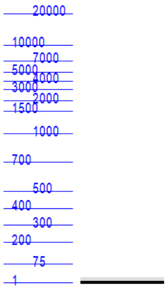

Peak	Size (bp)	Conc. (ng/uL)	From (bp)	To (bp)	Avg. Size (bp)	CV%	RFU	Corr. Peak Area
1	1 (LM)	0.0328	0	53	2	360.04	8238	57.529
2	8895	0.4232	810	36049	8142	72.10	312	61.850

TIC:	0.4232	ng/uL
TIM:	0.0856	nmole/L
Total Conc.:	0.4956	ng/uL
GQN:	2.8	

Sample Peak Width (sec): 50   Sample Min Peak Height: 50   Sample Baseline V to V?: Y   Sample Baseline V to V pts: 3
Sample Filter: Binomial   # of Pts for Filter: 3   Sample Start Region (min): 0   Sample End Region (min): 50
Manual Baseline Start (min): 6   Manual Baseline End (min): 48
Marker Peak Width (sec): 5   Marker Min Peak Height: 200   Marker Baseline V to V?: Y   Marker Baseline V to V pts: 3
Lower Marker Selection: First Peak > 200 RFU   Upper Marker Selection: Last Peak > 200 RFU
Ladder Size (bp): 1, 75, 200, 300, 400, 500, 700, 1000, 1500, 2000, 3000, 4000, 5000, 7000, 10000, 20000
Quantification Using: Ladder   Final Concentration (ng/uL): 1.0417   Dilution Factor: 12.0
Size Threshold (b.p.): 10000

Copyright 2015 Advanced Analytical Technologies, Inc.

Data File: 2019 06 18 13H 16M.raw
Sample: 103613-001-059 (20x dil.)
Well Location: C8

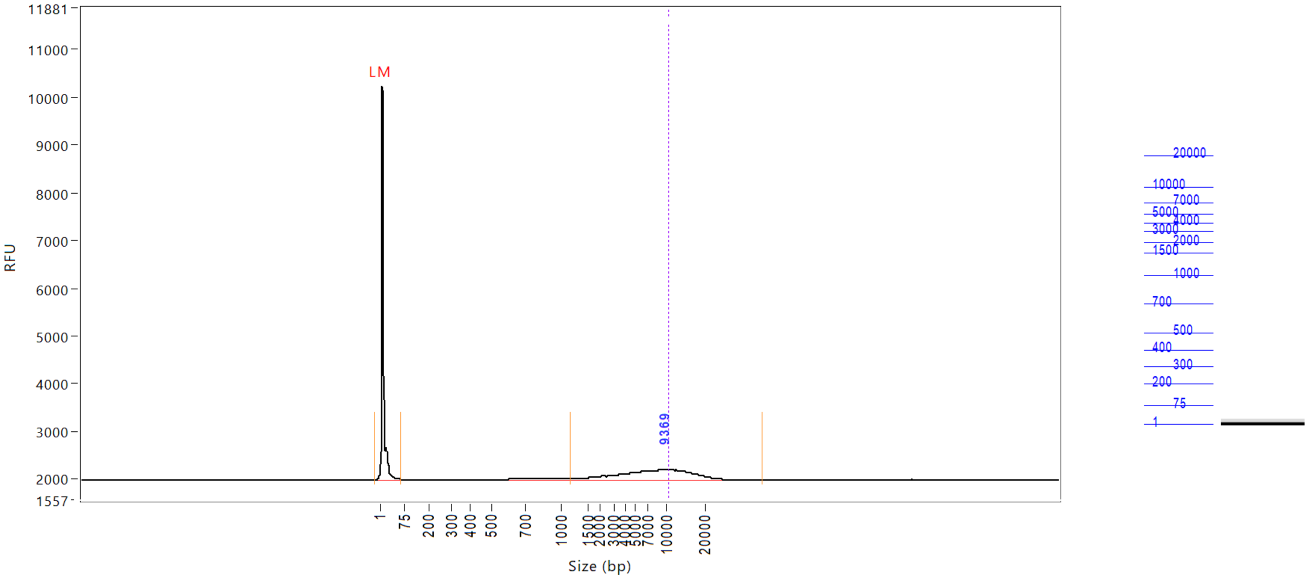

Peak	Size (bp)	Conc. (ng/uL)	From (bp)	To (bp)	Avg. Size (bp)	CV%	RFU	Corr. Peak Area
1	1 (LM)	0.0328	0	60	3	255.52	8252	58.864
2	9369	0.2326	1144	34927	8678	63.06	207	34.782

TIC:	0.2326	ng/uL
TIM:	0.0441	nmole/L
Total Conc.:	0.2641	ng/uL
GQN:	3.1	

Sample Peak Width (sec): 50 Sample Min Peak Height: 50 Sample Baseline V to V?: Y Sample Baseline V to V pts: 3
Sample Filter: Binomial # of Pts for Filter: 3 Sample Start Region (min): 0 Sample End Region (min): 50
Manual Baseline Start (min): 6 Manual Baseline End (min): 48
Marker Peak Width (sec): 5 Marker Min Peak Height: 200 Marker Baseline V to V?: Y Marker Baseline V to V pts: 3
Lower Marker Selection: First Peak > 200 RFU Upper Marker Selection: Last Peak > 200 RFU
Ladder Size (bp): 1, 75, 200, 300, 400, 500, 700, 1000, 1500, 2000, 3000, 4000, 5000, 7000, 10000, 20000
Quantification Using: Ladder Final Concentration (ng/uL): 1.0417 Dilution Factor: 12.0
Size Threshold (b.p.): 10000

 Copyright 2015 Advanced Analytical Technologies, Inc.

Data File: 2019 06 18 13H 16M.raw
Sample: 103613-001-060 (20x dil.)
Well Location: D8

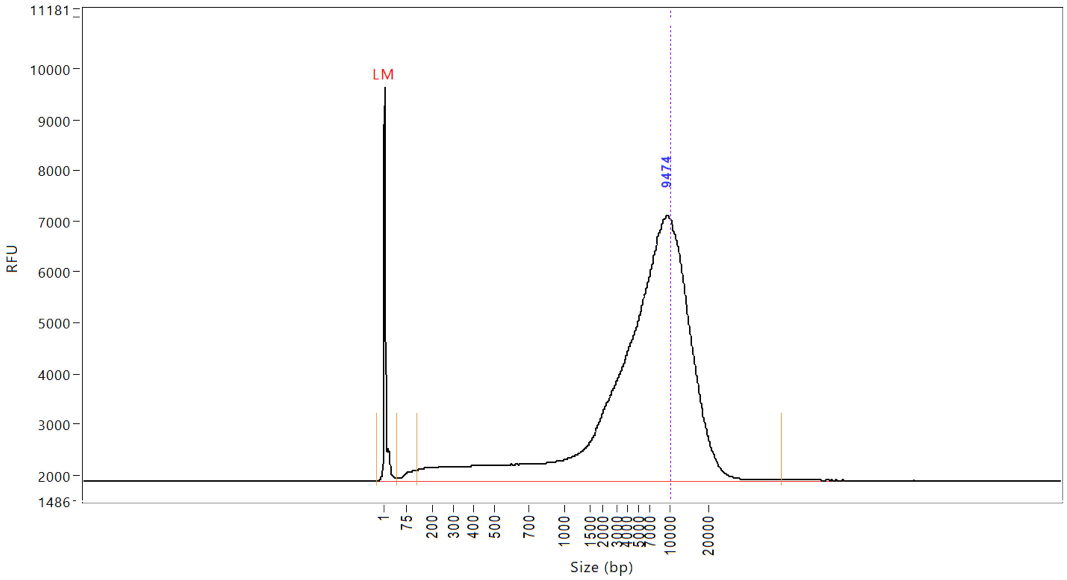

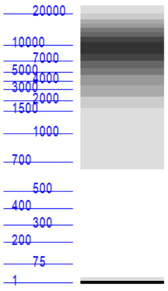

Peak	Size (bp)	Conc. (ng/uL)	From (bp)	To (bp)	Avg. Size (bp)	CV%	RFU	Corr. Peak Area
1	1 (LM)	0.0328	0	43	2	303.18	7748	53.663
2	9474	6.1033	122	39155	7179	75.19	5231	832.046

TIC:	6.1033	ng/uL
TIM:	1.3996	nmole/L
Total Conc.:	6.1841	ng/uL
GQN:	2.7	

Sample Peak Width (sec): 50 Sample Min Peak Height: 50 Sample Baseline V to V?: Y Sample Baseline V to V pts: 3
Sample Filter: Binomial # of Pts for Filter: 3 Sample Start Region (min): 0 Sample End Region (min): 50
Manual Baseline Start (min): 6 Manual Baseline End (min): 48
Marker Peak Width (sec): 5 Marker Min Peak Height: 200 Marker Baseline V to V?: Y Marker Baseline V to V pts: 3
Lower Marker Selection: First Peak > 200 RFU Upper Marker Selection: Last Peak > 200 RFU
Ladder Size (bp): 1, 75, 200, 300, 400, 500, 700, 1000, 1500, 2000, 3000, 4000, 5000, 7000, 10000, 20000
Quantification Using: Ladder Final Concentration (ng/uL): 1.0417 Dilution Factor: 12.0
Size Threshold (b.p.): 10000

 Copyright 2015 Advanced Analytical Technologies, Inc.

Data File: 2019 06 18 13H 16M.raw
Sample: 103613-001-061 (20x dil.)
Well Location: E8

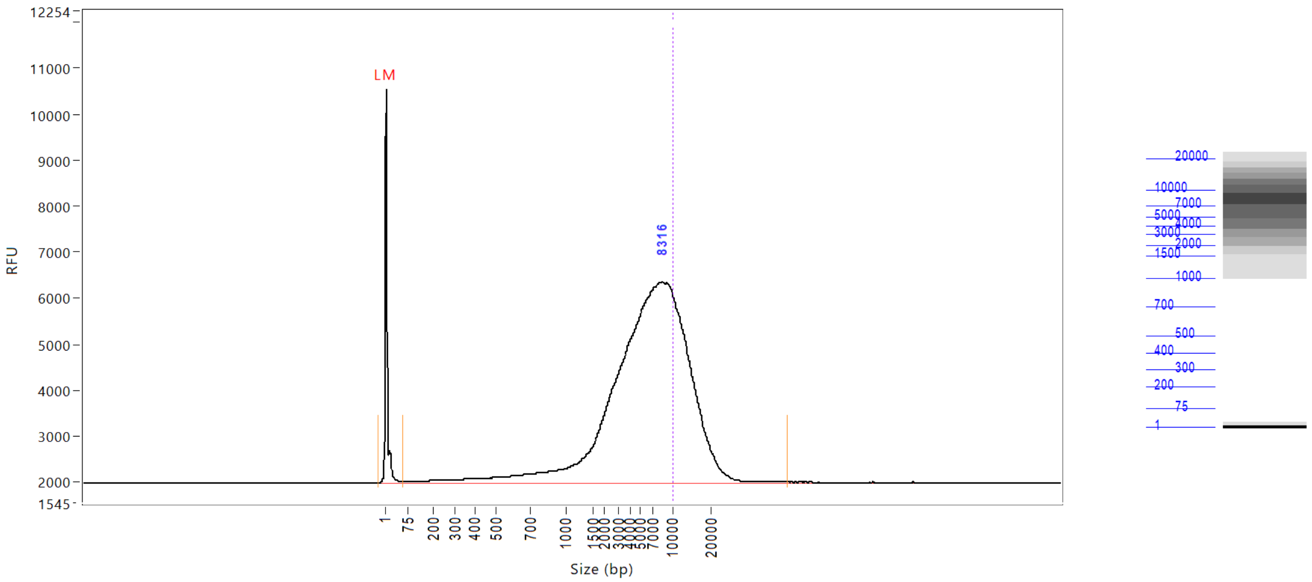

Peak	Size (bp)	Conc. (ng/uL)	From (bp)	To (bp)	Avg. Size (bp)	CV%	RFU	Corr. Peak Area
1	1 (LM)	0.0328	0	53	2	295.07	8561	59.499
2	8316	4.8282	53	39932	7150	71.71	4364	729.801

TIC:	4.8282	ng/uL
TIM:	1.1117	nmole/L
Total Conc.:	4.8671	ng/uL
GQN:	2.5	

Sample Peak Width (sec): 50 Sample Min Peak Height: 50 Sample Baseline V to V?: Y Sample Baseline V to V pts: 3
Sample Filter: Binomial # of Pts for Filter: 3 Sample Start Region (min): 0 Sample End Region (min): 50
Manual Baseline Start (min): 6 Manual Baseline End (min): 48
Marker Peak Width (sec): 5 Marker Min Peak Height: 200 Marker Baseline V to V?: Y Marker Baseline V to V pts: 3
Lower Marker Selection: First Peak > 200 RFU Upper Marker Selection: Last Peak > 200 RFU
Ladder Size (bp): 1, 75, 200, 300, 400, 500, 700, 1000, 1500, 2000, 3000, 4000, 5000, 7000, 10000, 20000
Quantification Using: Ladder Final Concentration (ng/uL): 1.0417 Dilution Factor: 12.0
Size Threshold (b.p.): 10000

 Copyright 2015 Advanced Analytical Technologies, Inc.

Data File: 2019 06 18 13H 16M.raw
Sample: 103613-001-062 (20x dil.)
Well Location: F8

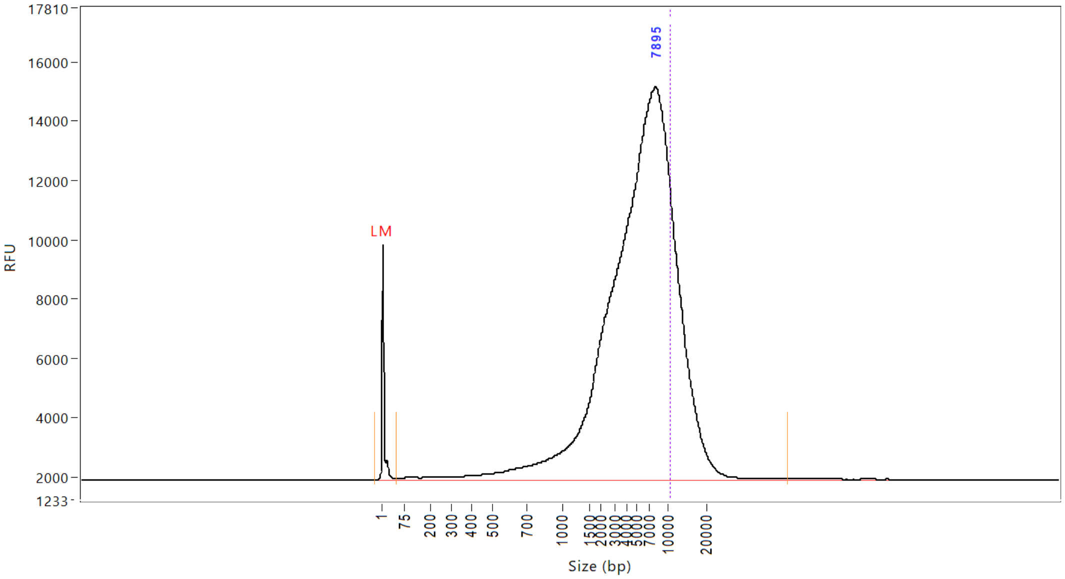

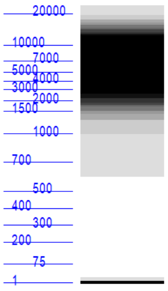

Peak	Size (bp)	Conc. (ng/uL)	From (bp)	To (bp)	Avg. Size (bp)	CV%	RFU	Corr. Peak Area
1	1 (LM)	0.0328	0	46	2	328.22	7952	55.401
2	7895	12.9344	46	41312	6404	69.12	13247	1820.399

TIC:	12.9344	ng/uL
TIM:	3.3251	nmole/L
Total Conc.:	12.9809	ng/uL
GQN:	1.8	

Sample Peak Width (sec): 50   Sample Min Peak Height: 50   Sample Baseline V to V?: Y   Sample Baseline V to V pts: 3
Sample Filter: Binomial   # of Pts for Filter: 3   Sample Start Region (min): 0   Sample End Region (min): 50
Manual Baseline Start (min): 6   Manual Baseline End (min): 48
Marker Peak Width (sec): 5   Marker Min Peak Height: 200   Marker Baseline V to V?: Y   Marker Baseline V to V pts: 3
Lower Marker Selection: First Peak > 200 RFU   Upper Marker Selection: Last Peak > 200 RFU
Ladder Size (bp): 1, 75, 200, 300, 400, 500, 700, 1000, 1500, 2000, 3000, 4000, 5000, 7000, 10000, 20000
Quantification Using: Ladder   Final Concentration (ng/uL): 1.0417   Dilution Factor: 12.0
Size Threshold (b.p.): 10000

 Copyright 2015 Advanced Analytical Technologies, Inc.

Data File: 2019 06 18 13H 16M.raw
Sample: 103613-001-063 (20x dil.)
Well Location: G8

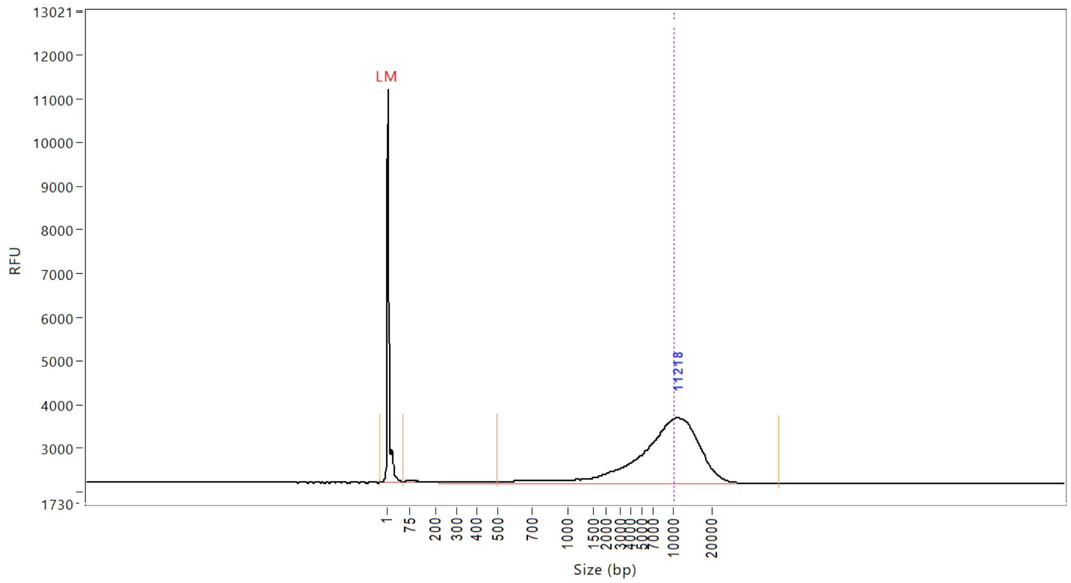

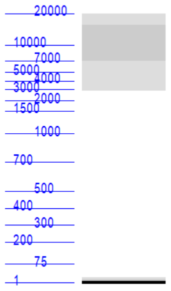

Peak	Size (bp)	Conc. (ng/uL)	From (bp)	To (bp)	Avg. Size (bp)	CV%	RFU	Corr. Peak Area
1	1 (LM)	0.0328	0	53	3	264.71	9009	64.040
2	11218	1.2195	495	37688	9360	55.46	1506	198.396

TIC:	1.2195	ng/uL
TIM:	0.2145	nmole/L
Total Conc.:	1.2608	ng/uL
GQN:	4.1	

Sample Peak Width (sec): 50 Sample Min Peak Height: 50 Sample Baseline V to V?: Y Sample Baseline V to V pts: 3
Sample Filter: Binomial # of Pts for Filter: 3 Sample Start Region (min): 0 Sample End Region (min): 50
Manual Baseline Start (min): 6 Manual Baseline End (min): 48
Marker Peak Width (sec): 5 Marker Min Peak Height: 200 Marker Baseline V to V?: Y Marker Baseline V to V pts: 3
Lower Marker Selection: First Peak > 200 RFU Upper Marker Selection: Last Peak > 200 RFU
Ladder Size (bp): 1, 75, 200, 300, 400, 500, 700, 1000, 1500, 2000, 3000, 4000, 5000, 7000, 10000, 20000
Quantification Using: Ladder Final Concentration (ng/uL): 1.0417 Dilution Factor: 12.0
Size Threshold (b.p.): 10000

 Copyright 2015 Advanced Analytical Technologies, Inc.

Data File: 2019 06 18 13H 16M.raw
Sample: 103613-001-064 (20x dil.)
Well Location: H8

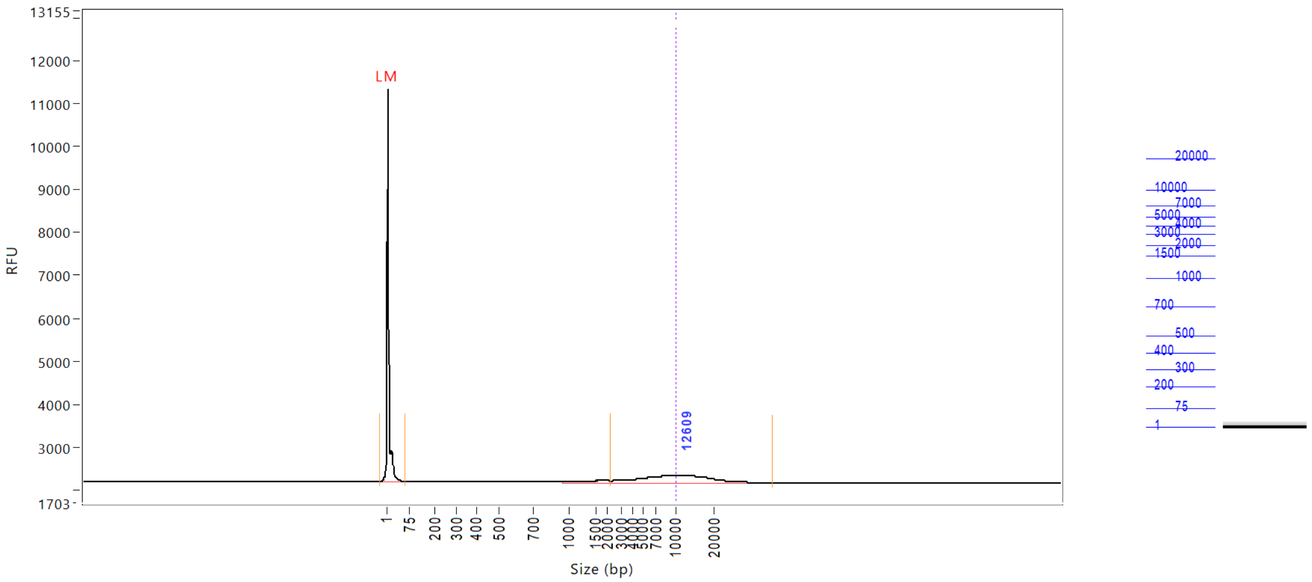

Peak	Size (bp)	Conc. (ng/uL)	From (bp)	To (bp)	Avg. Size (bp)	CV%	RFU	Corr. Peak Area
1	1 (LM)	0.0328	0	58	2	300.49	9132	63.645
2	12609	0.1563	2168	35359	10838	53.22	151	25.267

TIC:	0.1563	ng/uL
TIM:	0.0237	nmole/L
Total Conc.:	0.2017	ng/uL
GQN:	3.8	

Sample Peak Width (sec): 50   Sample Min Peak Height: 50   Sample Baseline V to V?: Y   Sample Baseline V to V pts: 3
Sample Filter: Binomial   # of Pts for Filter: 3   Sample Start Region (min): 0   Sample End Region (min): 50
Manual Baseline Start (min): 6   Manual Baseline End (min): 48
Marker Peak Width (sec): 5   Marker Min Peak Height: 200   Marker Baseline V to V?: Y   Marker Baseline V to V pts: 3
Lower Marker Selection: First Peak > 200 RFU   Upper Marker Selection: Last Peak > 200 RFU
Ladder Size (bp): 1, 75, 200, 300, 400, 500, 700, 1000, 1500, 2000, 3000, 4000, 5000, 7000, 10000, 20000
Quantification Using: Ladder   Final Concentration (ng/uL): 1.0417   Dilution Factor: 12.0
Size Threshold (b.p.): 10000

Copyright 2015 Advanced Analytical Technologies, Inc.

Data File: 2019 06 18 13H 16M.raw
Sample: 103613-001-065 (20x dil.)
Well Location: A9

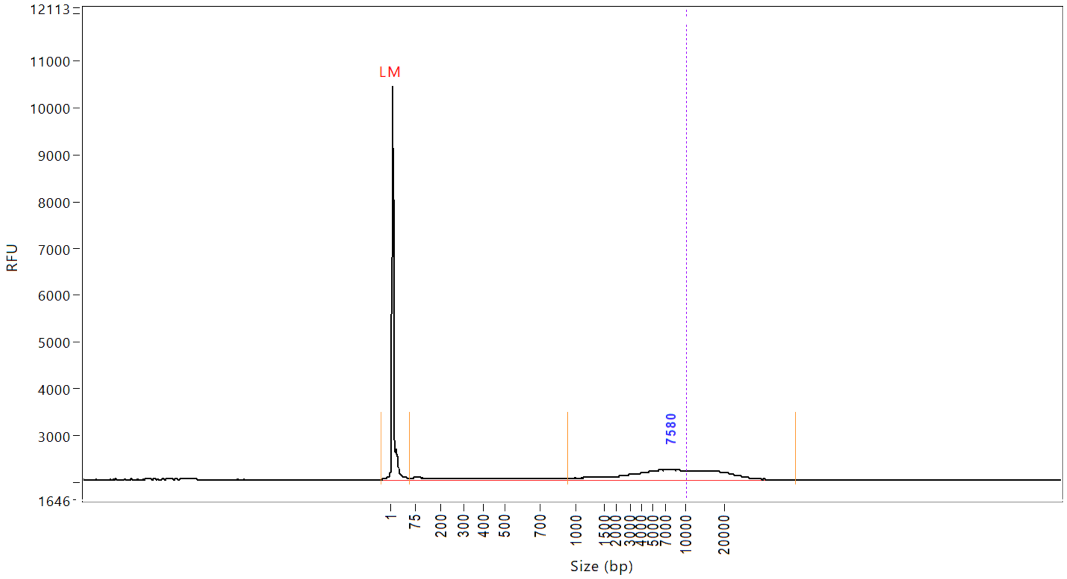

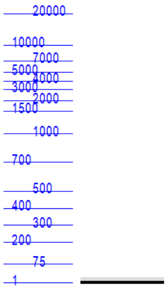

Peak	Size (bp)	Conc. (ng/uL)	From (bp)	To (bp)	Avg. Size (bp)	CV%	RFU	Corr. Peak Area
1	1 (LM)	0.0328	0	56	2	397.20	8360	58.688
2	7580	0.3220	927	38206	9759	72.17	203	48.007

TIC:	0.3220	ng/uL
TIM:	0.0543	nmole/L
Total Conc.:	0.3952	ng/uL
GQN:	3.1	

Sample Peak Width (sec): 50   Sample Min Peak Height: 50   Sample Baseline V to V?: Y   Sample Baseline V to V pts: 3
Sample Filter: Binomial   # of Pts for Filter: 3   Sample Start Region (min): 0   Sample End Region (min): 50
Manual Baseline Start (min): 6   Manual Baseline End (min): 48
Marker Peak Width (sec): 5   Marker Min Peak Height: 200   Marker Baseline V to V?: Y   Marker Baseline V to V pts: 3
Lower Marker Selection: First Peak > 200 RFU   Upper Marker Selection: Last Peak > 200 RFU
Ladder Size (bp): 1, 75, 200, 300, 400, 500, 700, 1000, 1500, 2000, 3000, 4000, 5000, 7000, 10000, 20000
Quantification Using: Ladder   Final Concentration (ng/uL): 1.0417   Dilution Factor: 12.0
Size Threshold (b.p.): 10000

 Copyright 2015 Advanced Analytical Technologies, Inc.

Data File: 2019 06 18 13H 16M.raw
Sample: 103613-001-066 (20x dil.)
Well Location: B9

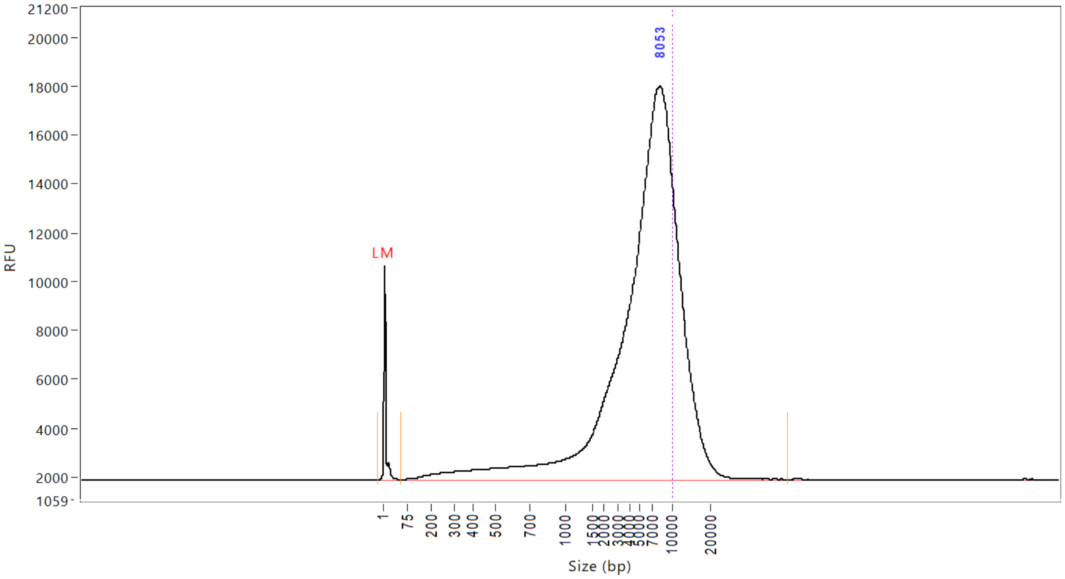

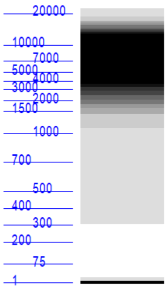

Peak	Size (bp)	Conc. (ng/uL)	From (bp)	To (bp)	Avg. Size (bp)	CV%	RFU	Corr. Peak Area
1	1 (LM)	0.0328	0	55	2	316.35	8748	60.227
2	8053	12.1277	55	40450	6641	64.10	16094	1855.558

TIC:	12.1277	ng/uL
TIM:	3.0065	nmole/L
Total Conc.:	12.1430	ng/uL
GQN:	1.9	

Sample Peak Width (sec): 50   Sample Min Peak Height: 50   Sample Baseline V to V?: Y   Sample Baseline V to V pts: 3
Sample Filter: Binomial   # of Pts for Filter: 3   Sample Start Region (min): 0   Sample End Region (min): 50
Manual Baseline Start (min): 6   Manual Baseline End (min): 48
Marker Peak Width (sec): 5   Marker Min Peak Height: 200   Marker Baseline V to V?: Y   Marker Baseline V to V pts: 3
Lower Marker Selection: First Peak > 200 RFU   Upper Marker Selection: Last Peak > 200 RFU
Ladder Size (bp): 1, 75, 200, 300, 400, 500, 700, 1000, 1500, 2000, 3000, 4000, 5000, 7000, 10000, 20000
Quantification Using: Ladder   Final Concentration (ng/uL): 1.0417   Dilution Factor: 12.0
Size Threshold (b.p.): 10000

 Copyright 2015 Advanced Analytical Technologies, Inc.

Data File: 2019 06 18 13H 16M.raw
Sample: 103613-001-067 (20x dil.)
Well Location: C9

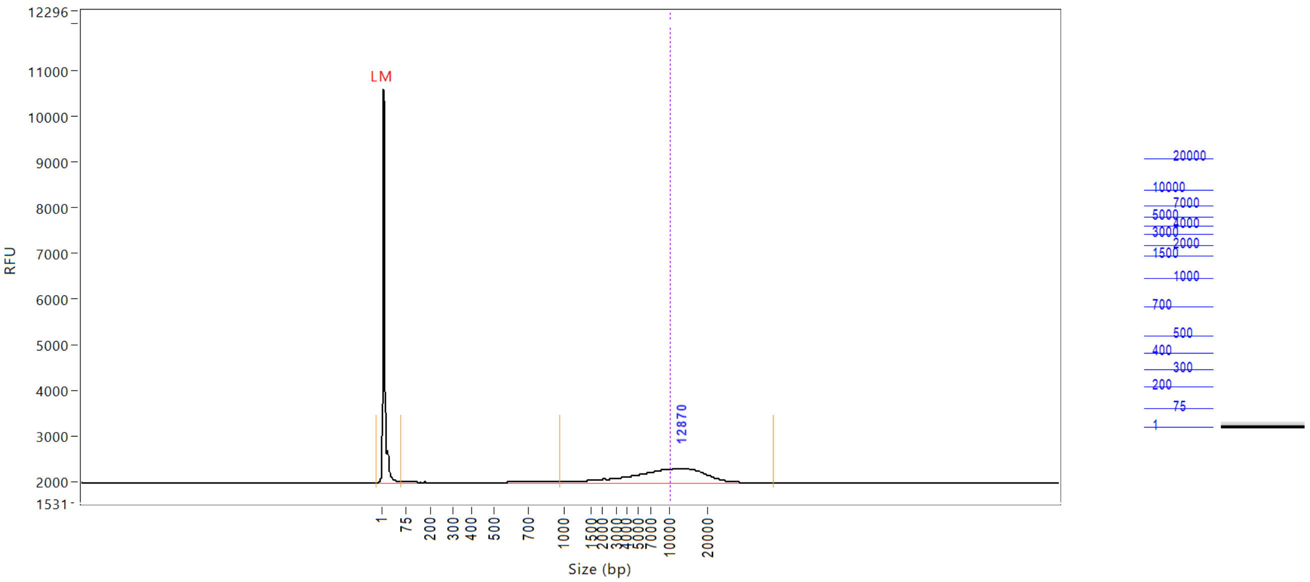

Peak	Size (bp)	Conc. (ng/uL)	From (bp)	To (bp)	Avg. Size (bp)	CV%	RFU	Corr. Peak Area
1	1 (LM)	0.0328	0	56	3	273.68	8606	59.830
2	12870	0.3682	961	37343	10354	62.73	316	55.971

TIC:	0.3682	ng/uL
TIM:	0.0586	nmole/L
Total Conc.:	0.4240	ng/uL
GQN:	4.1	

Sample Peak Width (sec): 50 Sample Min Peak Height: 50 Sample Baseline V to V?: Y Sample Baseline V to V pts: 3
Sample Filter: Binomial # of Pts for Filter: 3 Sample Start Region (min): 0 Sample End Region (min): 50
Manual Baseline Start (min): 6 Manual Baseline End (min): 48
Marker Peak Width (sec): 5 Marker Min Peak Height: 200 Marker Baseline V to V?: Y Marker Baseline V to V pts: 3
Lower Marker Selection: First Peak > 200 RFU Upper Marker Selection: Last Peak > 200 RFU
Ladder Size (bp): 1, 75, 200, 300, 400, 500, 700, 1000, 1500, 2000, 3000, 4000, 5000, 7000, 10000, 20000
Quantification Using: Ladder Final Concentration (ng/uL): 1.0417 Dilution Factor: 12.0
Size Threshold (b.p.): 10000

 Copyright 2015 Advanced Analytical Technologies, Inc.

Data File: 2019 06 18 13H 16M.raw
Sample: 103613-001-068 (20x dil.)
Well Location: D9

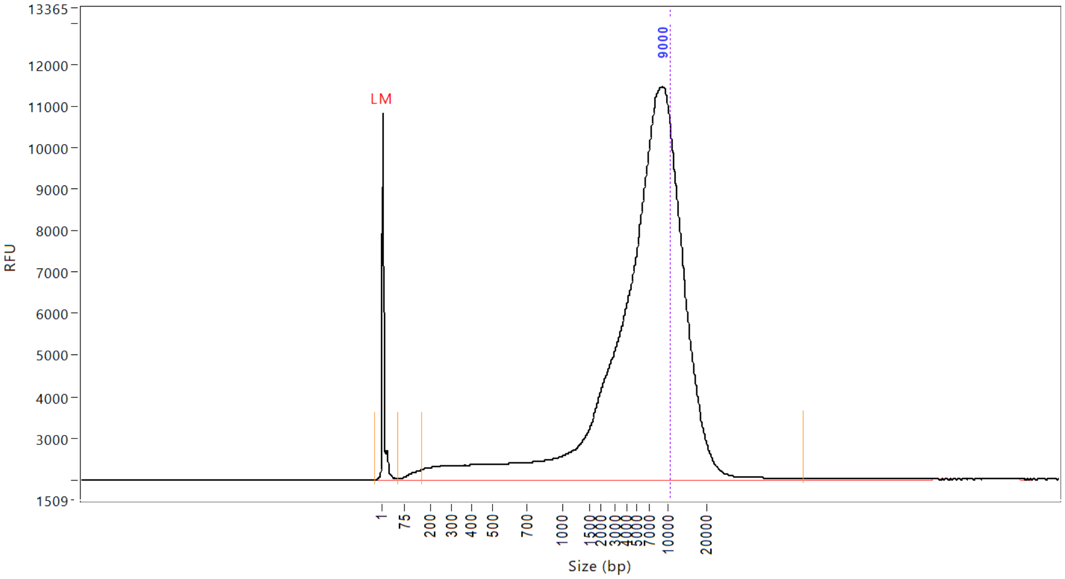

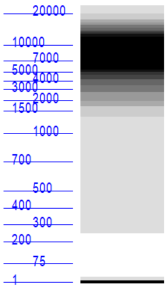

Peak	Size (bp)	Conc. (ng/uL)	From (bp)	To (bp)	Avg. Size (bp)	CV%	RFU	Corr. Peak Area
1	1 (LM)	0.0328	0	50	2	323.94	8844	60.674
2	9000	8.2598	154	45368	7115	70.56	9467	1273.141

TIC:	8.2598	ng/uL
TIM:	1.9112	nmole/L
Total Conc.:	8.3488	ng/uL
GQN:	2.4	

Sample Peak Width (sec): 50 Sample Min Peak Height: 50 Sample Baseline V to V?: Y Sample Baseline V to V pts: 3
Sample Filter: Binomial # of Pts for Filter: 3 Sample Start Region (min): 0 Sample End Region (min): 50
Manual Baseline Start (min): 6 Manual Baseline End (min): 48
Marker Peak Width (sec): 5 Marker Min Peak Height: 200 Marker Baseline V to V?: Y Marker Baseline V to V pts: 3
Lower Marker Selection: First Peak > 200 RFU Upper Marker Selection: Last Peak > 200 RFU
Ladder Size (bp): 1, 75, 200, 300, 400, 500, 700, 1000, 1500, 2000, 3000, 4000, 5000, 7000, 10000, 20000
Quantification Using: Ladder Final Concentration (ng/uL): 1.0417 Dilution Factor: 12.0
Size Threshold (b.p.): 10000

 Copyright 2015 Advanced Analytical Technologies, Inc.

Data File: 2019 06 18 13H 16M.raw
Sample: 103613-001-069 (20x dil.)
Well Location: E9

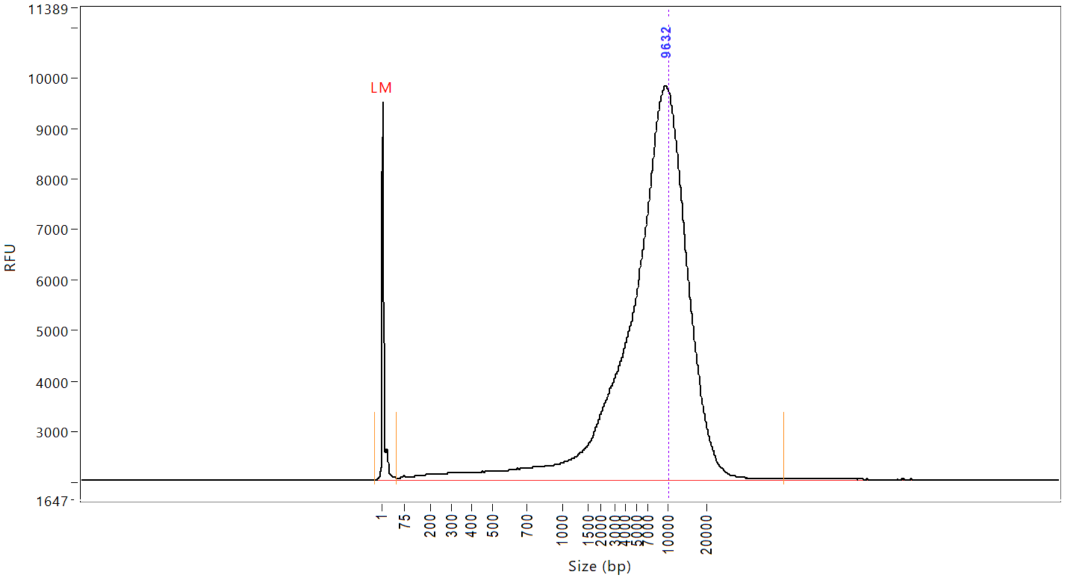

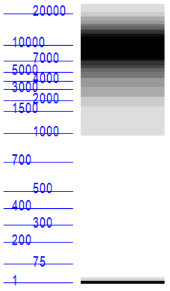

Peak	Size (bp)	Conc. (ng/uL)	From (bp)	To (bp)	Avg. Size (bp)	CV%	RFU	Corr. Peak Area
1	1 (LM)	0.0328	0	46	3	254.98	7487	52.609
2	9632	7.4061	46	40277	8227	62.62	7780	989.823

TIC:	7.4061	ng/uL
TIM:	1.4820	nmole/L
Total Conc.:	7.4291	ng/uL
GQN:	3.3	

Sample Peak Width (sec): 50 Sample Min Peak Height: 50 Sample Baseline V to V?: Y Sample Baseline V to V pts: 3
Sample Filter: Binomial # of Pts for Filter: 3 Sample Start Region (min): 0 Sample End Region (min): 50
Manual Baseline Start (min): 6 Manual Baseline End (min): 48
Marker Peak Width (sec): 5 Marker Min Peak Height: 200 Marker Baseline V to V?: Y Marker Baseline V to V pts: 3
Lower Marker Selection: First Peak > 200 RFU Upper Marker Selection: Last Peak > 200 RFU
Ladder Size (bp): 1, 75, 200, 300, 400, 500, 700, 1000, 1500, 2000, 3000, 4000, 5000, 7000, 10000, 20000
Quantification Using: Ladder Final Concentration (ng/uL): 1.0417 Dilution Factor: 12.0
Size Threshold (b.p.): 10000

 Copyright 2015 Advanced Analytical Technologies, Inc.

Data File: 2019 06 18 13H 16M.raw
Sample: 103613-001-070 (20x dil.)
Well Location: F9

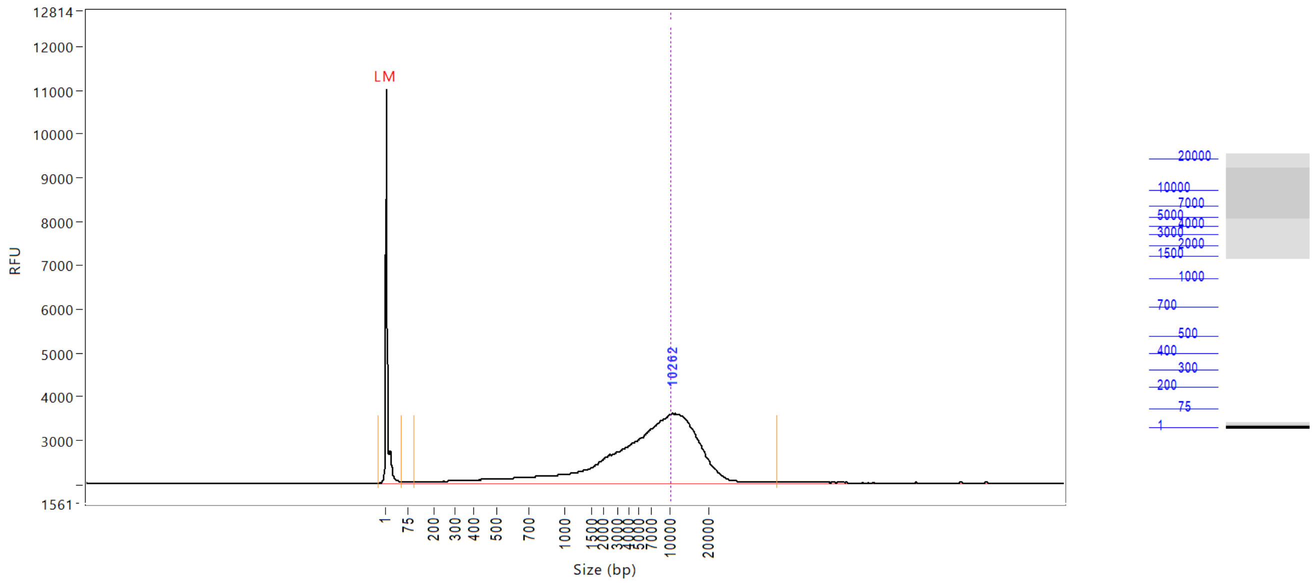

Peak	Size (bp)	Conc. (ng/uL)	From (bp)	To (bp)	Avg. Size (bp)	CV%	RFU	Corr. Peak Area
1	1 (LM)	0.0328	0	50	3	278.67	8991	62.398
2	10262	1.9288	101	38034	7903	77.88	1596	305.755

TIC:	1.9288	ng/uL
TIM:	0.4018	nmole/L
Total Conc.:	1.9781	ng/uL
GQN:	3.4	

Sample Peak Width (sec): 50 Sample Min Peak Height: 50 Sample Baseline V to V?: Y Sample Baseline V to V pts: 3
Sample Filter: Binomial # of Pts for Filter: 3 Sample Start Region (min): 0 Sample End Region (min): 50
Manual Baseline Start (min): 6 Manual Baseline End (min): 48
Marker Peak Width (sec): 5 Marker Min Peak Height: 200 Marker Baseline V to V?: Y Marker Baseline V to V pts: 3
Lower Marker Selection: First Peak > 200 RFU Upper Marker Selection: Last Peak > 200 RFU
Ladder Size (bp): 1, 75, 200, 300, 400, 500, 700, 1000, 1500, 2000, 3000, 4000, 5000, 7000, 10000, 20000
Quantification Using: Ladder Final Concentration (ng/uL): 1.0417 Dilution Factor: 12.0
Size Threshold (b.p.): 10000

 Copyright 2015 Advanced Analytical Technologies, Inc.

Data File: 2019 06 18 13H 16M.raw
Sample: 103613-001-071 (20x dil.)
Well Location: G9

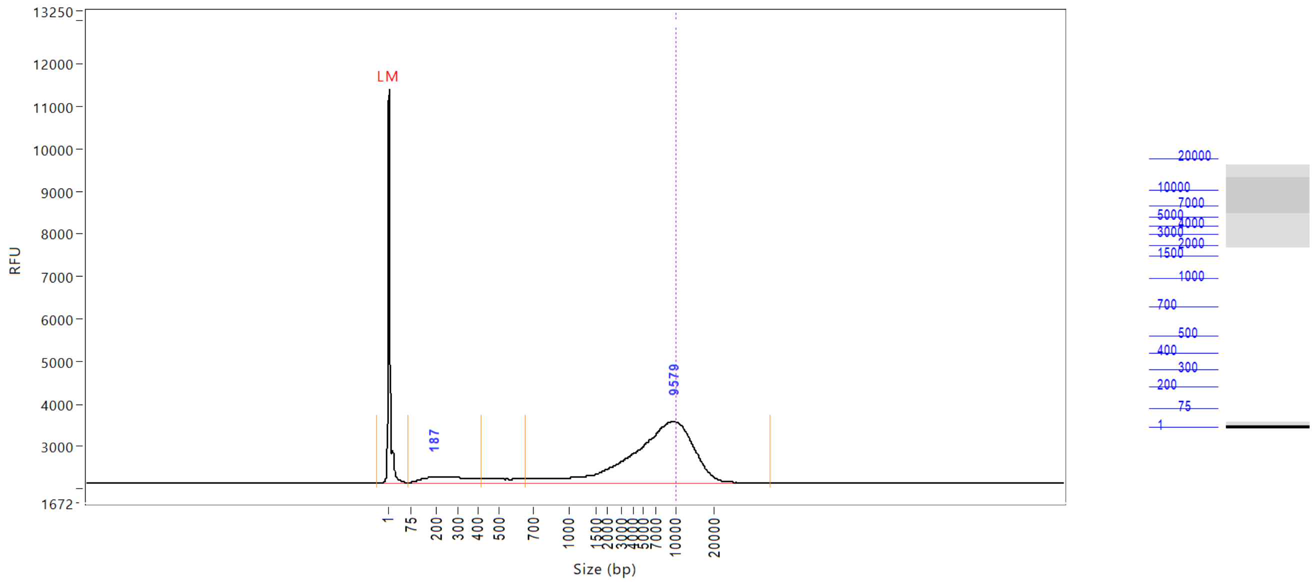

Peak	Size (bp)	Conc. (ng/uL)	From (bp)	To (bp)	Avg. Size (bp)	CV%	RFU	Corr. Peak Area
1	1 (LM)	0.0328	0	64	2	371.97	9255	65.471
2	187	0.1296	64	416	246	36.31	139	21.554
3	9579	1.1824	651	34582	7696	63.02	1455	196.655

TIC:	1.3119	ng/uL
TIM:	1.1193	nmole/L
Total Conc.:	1.3810	ng/uL
GQN:	2.5	

Sample Peak Width (sec): 50 Sample Min Peak Height: 50 Sample Baseline V to V?: Y Sample Baseline V to V pts: 3
Sample Filter: Binomial # of Pts for Filter: 3 Sample Start Region (min): 0 Sample End Region (min): 50
Manual Baseline Start (min): 6 Manual Baseline End (min): 48
Marker Peak Width (sec): 5 Marker Min Peak Height: 200 Marker Baseline V to V?: Y Marker Baseline V to V pts: 3
Lower Marker Selection: First Peak > 200 RFU Upper Marker Selection: Last Peak > 200 RFU
Ladder Size (bp): 1, 75, 200, 300, 400, 500, 700, 1000, 1500, 2000, 3000, 4000, 5000, 7000, 10000, 20000
Quantification Using: Ladder Final Concentration (ng/uL): 1.0417 Dilution Factor: 12.0
Size Threshold (b.p.): 10000

 Copyright 2015 Advanced Analytical Technologies, Inc.

Data File: 2019 06 18 13H 16M.raw
Sample: 103613-001-072 (20x dil.)
Well Location: H9

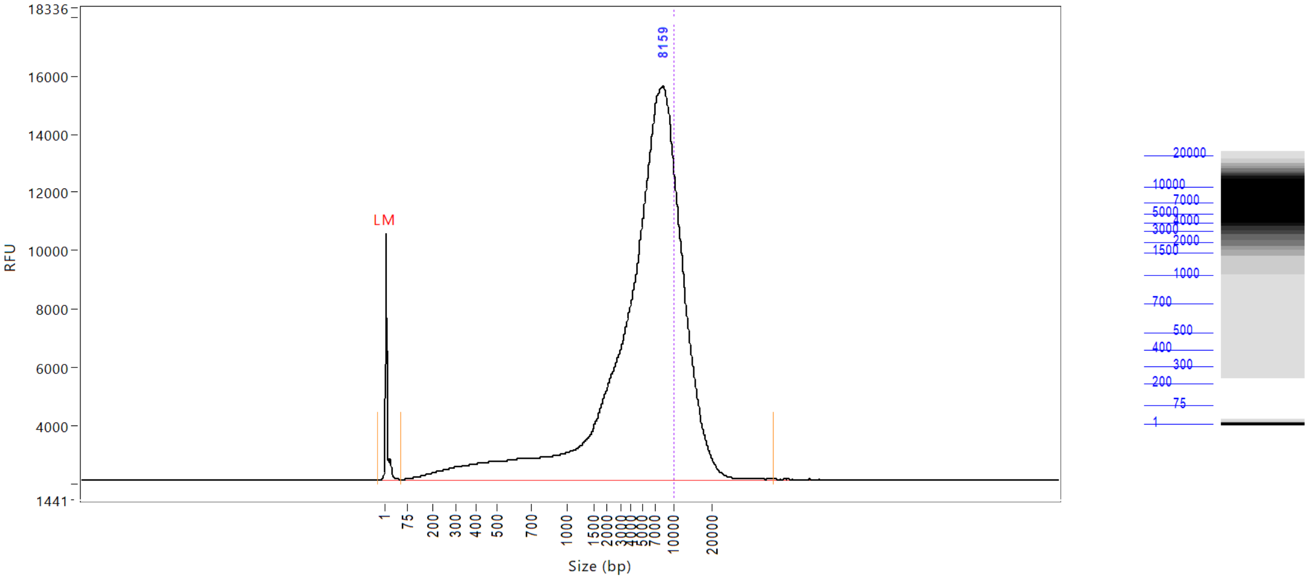

Peak	Size (bp)	Conc. (ng/uL)	From (bp)	To (bp)	Avg. Size (bp)	CV%	RFU	Corr. Peak Area
1	1 (LM)	0.0328	0	50	2	294.31	8473	58.404
2	8159	11.4566	50	35876	6321	69.46	13507	1699.829

TIC:	11.4566	ng/uL
TIM:	2.9838	nmole/L
Total Conc.:	11.5051	ng/uL
GQN:	1.8	

Sample Peak Width (sec): 50   Sample Min Peak Height: 50   Sample Baseline V to V?: Y   Sample Baseline V to V pts: 3
Sample Filter: Binomial   # of Pts for Filter: 3   Sample Start Region (min): 0   Sample End Region (min): 50
Manual Baseline Start (min): 6   Manual Baseline End (min): 48
Marker Peak Width (sec): 5   Marker Min Peak Height: 200   Marker Baseline V to V?: Y   Marker Baseline V to V pts: 3
Lower Marker Selection: First Peak > 200 RFU   Upper Marker Selection: Last Peak > 200 RFU
Ladder Size (bp): 1, 75, 200, 300, 400, 500, 700, 1000, 1500, 2000, 3000, 4000, 5000, 7000, 10000, 20000
Quantification Using: Ladder   Final Concentration (ng/uL): 1.0417   Dilution Factor: 12.0
Size Threshold (b.p.): 10000

Copyright 2015 Advanced Analytical Technologies, Inc.

Data File: 2019 06 18 13H 16M.raw
Sample: 103613-001-073 (20x dil.)
Well Location: A10

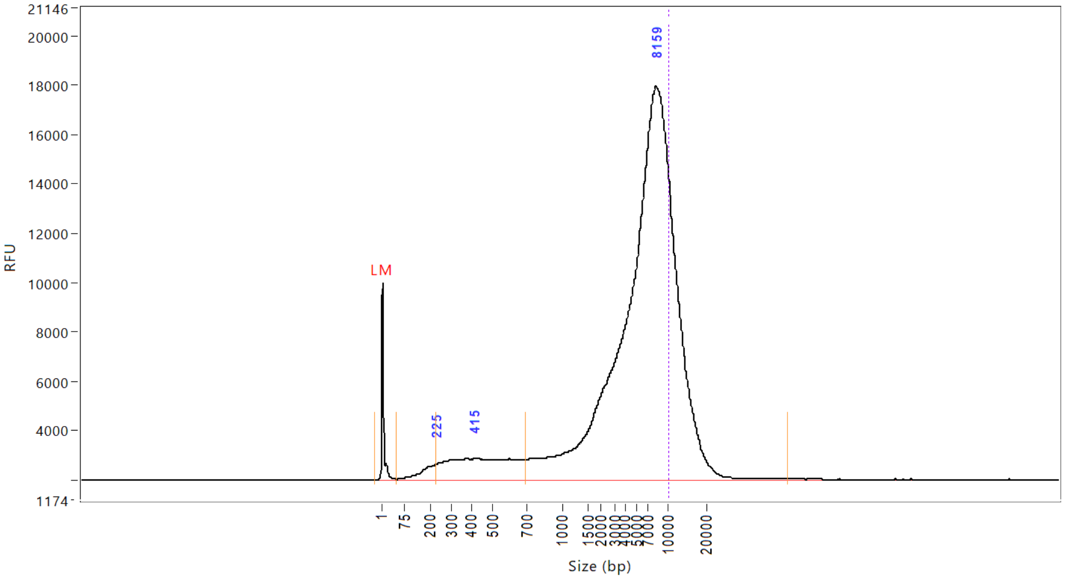

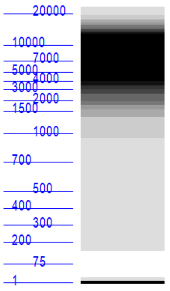

Peak	Size (bp)	Conc. (ng/uL)	From (bp)	To (bp)	Avg. Size (bp)	CV%	RFU	Corr. Peak Area
1	1 (LM)	0.0328	0	45	2	329.85	7962	55.243
2	225	0.2354	45	227	168	27.25	652	33.029
3	415	1.3025	227	694	444	29.75	841	182.788
4	8159	12.3491	694	41658	7044	60.93	15958	1733.077

TIC:	13.8869	ng/uL
TIM:	10.0158	nmole/L
Total Conc.:	13.9100	ng/uL
GQN:	1.8	

Sample Peak Width (sec): 50 Sample Min Peak Height: 50 Sample Baseline V to V?: Y Sample Baseline V to V pts: 3
Sample Filter: Binomial # of Pts for Filter: 3 Sample Start Region (min): 0 Sample End Region (min): 50
Manual Baseline Start (min): 6 Manual Baseline End (min): 48
Marker Peak Width (sec): 5 Marker Min Peak Height: 200 Marker Baseline V to V?: Y Marker Baseline V to V pts: 3
Lower Marker Selection: First Peak > 200 RFU Upper Marker Selection: Last Peak > 200 RFU
Ladder Size (bp): 1, 75, 200, 300, 400, 500, 700, 1000, 1500, 2000, 3000, 4000, 5000, 7000, 10000, 20000
Quantification Using: Ladder Final Concentration (ng/uL): 1.0417 Dilution Factor: 12.0
Size Threshold (b.p.): 10000

 Copyright 2015 Advanced Analytical Technologies, Inc.

Data File: 2019 06 18 13H 16M.raw
Sample: 103613-001-074 (20x dil.)
Well Location: B10

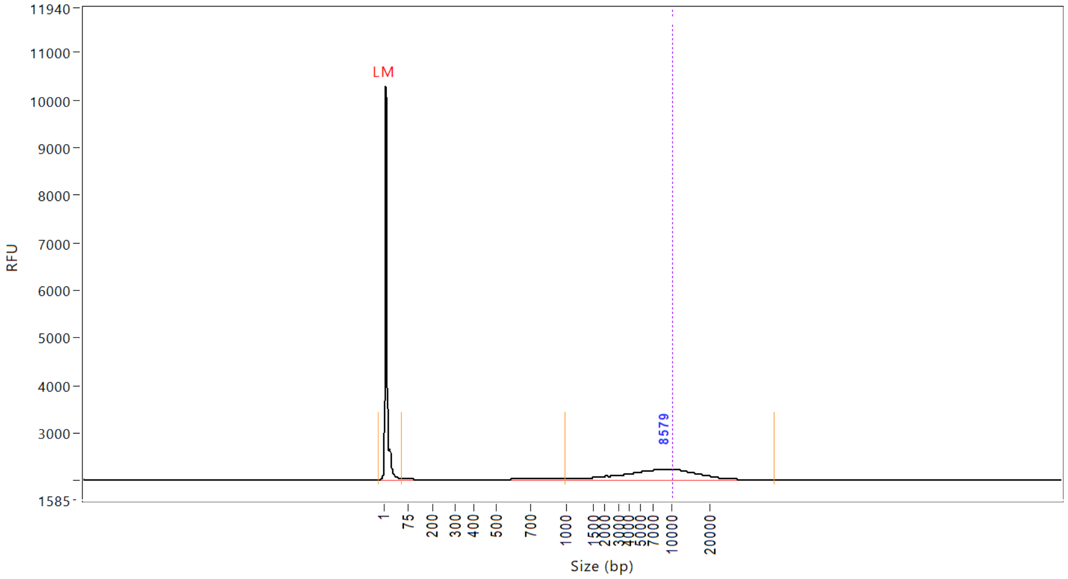

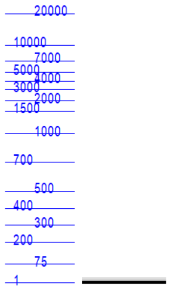

Peak	Size (bp)	Conc. (ng/uL)	From (bp)	To (bp)	Avg. Size (bp)	CV%	RFU	Corr. Peak Area
1	1 (LM)	0.0328	0	52	3	269.51	8277	57.647
2	8579	0.2579	992	36998	8927	69.58	217	37.762

TIC:	0.2579	ng/uL
TIM:	0.0476	nmole/L
Total Conc.:	0.3045	ng/uL
GQN:	3.1	

Sample Peak Width (sec): 50 Sample Min Peak Height: 50 Sample Baseline V to V?: Y Sample Baseline V to V pts: 3
Sample Filter: Binomial # of Pts for Filter: 3 Sample Start Region (min): 0 Sample End Region (min): 50
Manual Baseline Start (min): 6 Manual Baseline End (min): 48
Marker Peak Width (sec): 5 Marker Min Peak Height: 200 Marker Baseline V to V?: Y Marker Baseline V to V pts: 3
Lower Marker Selection: First Peak > 200 RFU Upper Marker Selection: Last Peak > 200 RFU
Ladder Size (bp): 1, 75, 200, 300, 400, 500, 700, 1000, 1500, 2000, 3000, 4000, 5000, 7000, 10000, 20000
Quantification Using: Ladder Final Concentration (ng/uL): 1.0417 Dilution Factor: 12.0
Size Threshold (b.p.): 10000

 Copyright 2015 Advanced Analytical Technologies, Inc.

Data File: 2019 06 18 13H 16M.raw
Sample: 103613-001-075 (20x dil.)
Well Location: C10

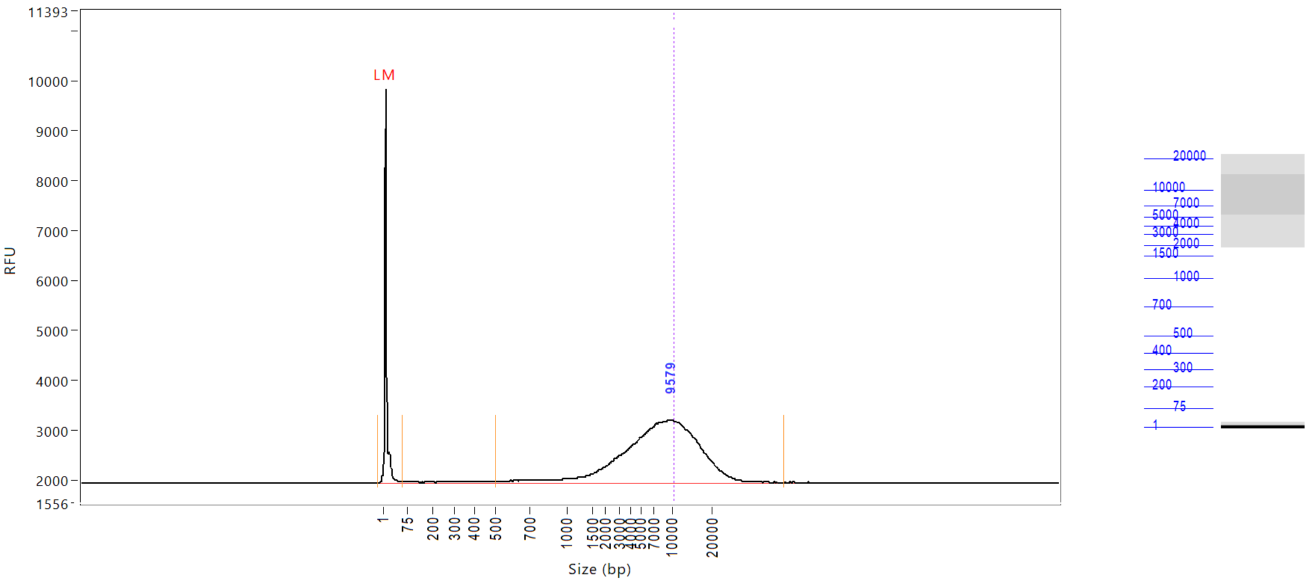

Peak	Size (bp)	Conc. (ng/uL)	From (bp)	To (bp)	Avg. Size (bp)	CV%	RFU	Corr. Peak Area
1	1 (LM)	0.0328	0	57	3	265.17	7863	55.838
2	9579	1.5377	498	38810	8757	67.30	1255	218.119

TIC:	1.5377	ng/uL
TIM:	0.2891	nmole/L
Total Conc.:	1.5879	ng/uL
GQN:	3.5	

```
Sample Peak Width (sec): 50    Sample Min Peak Height: 50     Sample Baseline V to V?: Y     Sample Baseline V to V pts: 3
Sample Filter: Binomial        # of Pts for Filter: 3         Sample Start Region (min): 0   Sample End Region (min): 50
Manual Baseline Start (min): 6      Manual Baseline End (min): 48
Marker Peak Width (sec): 5     Marker Min Peak Height: 200    Marker Baseline V to V?: Y     Marker Baseline V to V pts: 3
Lower Marker Selection: First Peak > 200 RFU                  Upper Marker Selection: Last Peak > 200 RFU
Ladder Size (bp): 1, 75, 200, 300, 400, 500, 700, 1000, 1500, 2000, 3000, 4000, 5000, 7000, 10000, 20000
Quantification Using: Ladder          Final Concentration (ng/uL): 1.0417          Dilution Factor: 12.0
Size Threshold (b.p.): 10000
```


Copyright 2015 Advanced Analytical Technologies, Inc.

Data File: 2019 06 18 13H 16M.raw
Sample: 103613-001-076 (20x dil.)
Well Location: D10

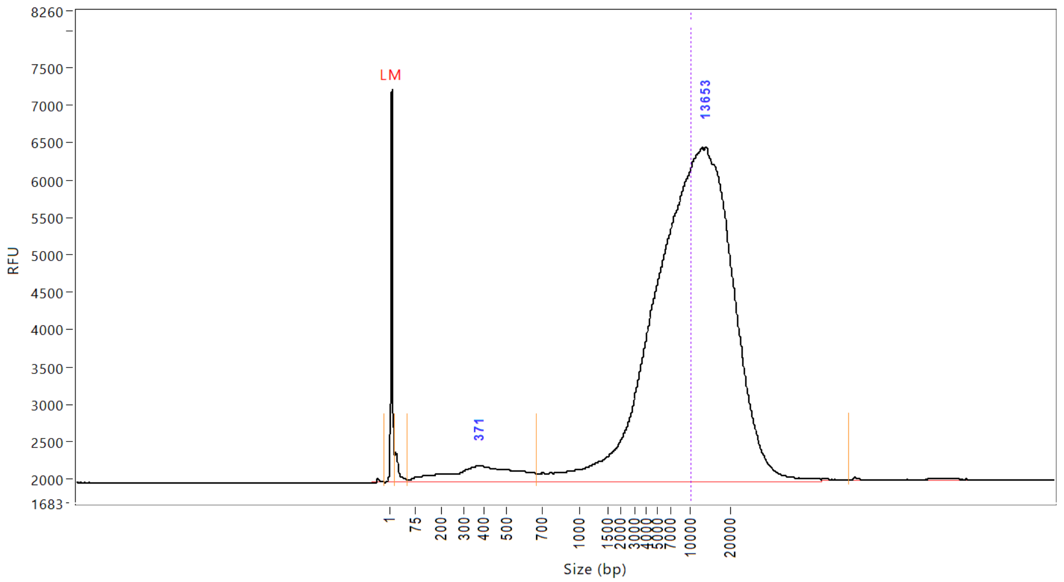

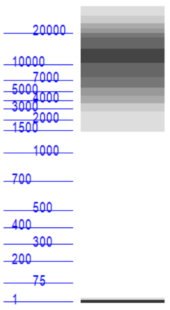

Peak	Size (bp)	Conc. (ng/uL)	From (bp)	To (bp)	Avg. Size (bp)	CV%	RFU	Corr. Peak Area
1	1 (LM)	0.0328	0	9	0	-1709.09	5248	32.840
2	371	0.4908	49	665	365	42.35	218	40.942
3	13653	10.1617	665	49596	11288	59.55	4461	847.763

TIC:	10.6525	ng/uL
TIM:	3.6941	nmole/L
Total Conc.:	10.7700	ng/uL
GQN:	4.8	

```
Sample Peak Width (sec): 50   Sample Min Peak Height: 50    Sample Baseline V to V?: Y    Sample Baseline V to V pts: 3
Sample Filter: Binomial       # of Pts for Filter: 3        Sample Start Region (min): 0  Sample End Region (min): 50
Manual Baseline Start (min): 6     Manual Baseline End (min): 48
Marker Peak Width (sec): 5    Marker Min Peak Height: 200   Marker Baseline V to V?: Y    Marker Baseline V to V pts: 3
Lower Marker Selection: First Peak > 200 RFU                Upper Marker Selection: Last Peak > 200 RFU
Ladder Size (bp): 1, 75, 200, 300, 400, 500, 700, 1000, 1500, 2000, 3000, 4000, 5000, 7000, 10000, 20000
Quantification Using: Ladder          Final Concentration (ng/uL): 1.0417          Dilution Factor: 12.0
Size Threshold (b.p.): 10000
```

 Copyright 2015 Advanced Analytical Technologies, Inc.

Data File: 2019 06 18 13H 16M.raw
Sample: 103613-001-077 (20x dil.)
Well Location: E10

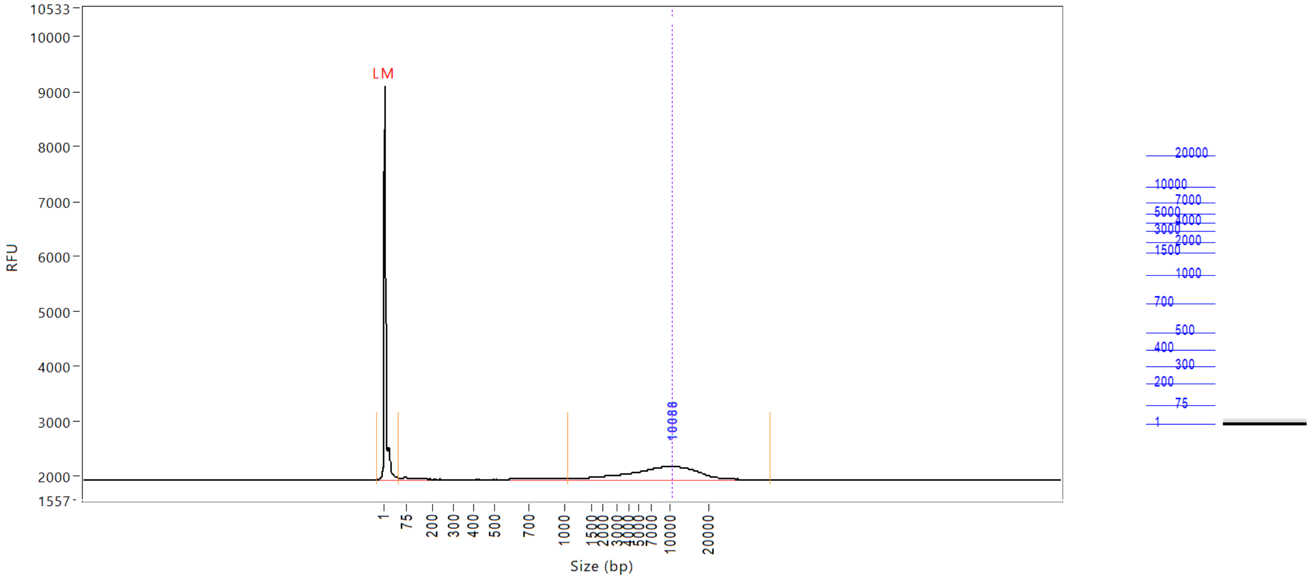

Peak	Size (bp)	Conc. (ng/uL)	From (bp)	To (bp)	Avg. Size (bp)	CV%	RFU	Corr. Peak Area
1	1 (LM)	0.0328	0	45	2	374.94	7174	51.276
2	10088	0.2924	1038	36222	9344	63.31	241	38.090

TIC:	0.2924	ng/uL
TIM:	0.0515	nmole/L
Total Conc.:	0.3605	ng/uL
GQN:	3.4	

```
Sample Peak Width (sec): 50   Sample Min Peak Height: 50    Sample Baseline V to V?: Y    Sample Baseline V to V pts: 3
Sample Filter: Binomial       # of Pts for Filter: 3        Sample Start Region (min): 0  Sample End Region (min): 50
Manual Baseline Start (min): 6     Manual Baseline End (min): 48
Marker Peak Width (sec): 5    Marker Min Peak Height: 200   Marker Baseline V to V?: Y    Marker Baseline V to V pts: 3
Lower Marker Selection: First Peak > 200 RFU                Upper Marker Selection: Last Peak > 200 RFU
Ladder Size (bp): 1, 75, 200, 300, 400, 500, 700, 1000, 1500, 2000, 3000, 4000, 5000, 7000, 10000, 20000
Quantification Using: Ladder          Final Concentration (ng/uL): 1.0417          Dilution Factor: 12.0
Size Threshold (b.p.): 10000
```

 Copyright 2015 Advanced Analytical Technologies, Inc.

Data File: 2019 06 18 13H 16M.raw
Sample: 103613-001-078 (20x dil.)
Well Location: F10

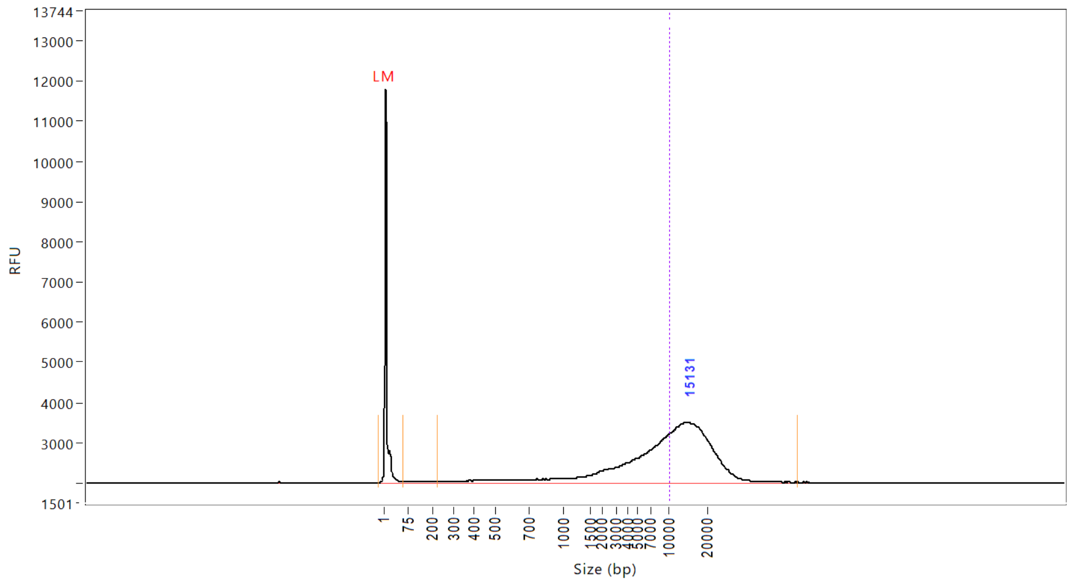

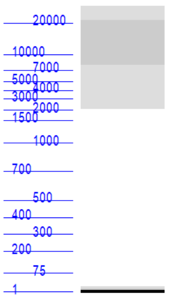

Peak	Size (bp)	Conc. (ng/uL)	From (bp)	To (bp)	Avg. Size (bp)	CV%	RFU	Corr. Peak Area
1	1 (LM)	0.0328	0	57	2	332.82	9783	68.736
2	15131	1.6756	215	43901	10871	68.35	1516	292.598

TIC:	1.6756	ng/uL
TIM:	0.2538	nmole/L
Total Conc.:	1.7106	ng/uL
GQN:	5.1	

Sample Peak Width (sec): 50 Sample Min Peak Height: 50 Sample Baseline V to V?: Y Sample Baseline V to V pts: 3
Sample Filter: Binomial # of Pts for Filter: 3 Sample Start Region (min): 0 Sample End Region (min): 50
Manual Baseline Start (min): 6 Manual Baseline End (min): 48
Marker Peak Width (sec): 5 Marker Min Peak Height: 200 Marker Baseline V to V?: Y Marker Baseline V to V pts: 3
Lower Marker Selection: First Peak > 200 RFU Upper Marker Selection: Last Peak > 200 RFU
Ladder Size (bp): 1, 75, 200, 300, 400, 500, 700, 1000, 1500, 2000, 3000, 4000, 5000, 7000, 10000, 20000
Quantification Using: Ladder Final Concentration (ng/uL): 1.0417 Dilution Factor: 12.0
Size Threshold (b.p.): 10000

 Copyright 2015 Advanced Analytical Technologies, Inc.

Data File: 2019 06 18 13H 16M.raw
Sample: 103613-001-079 (20x dil.)
Well Location: G10

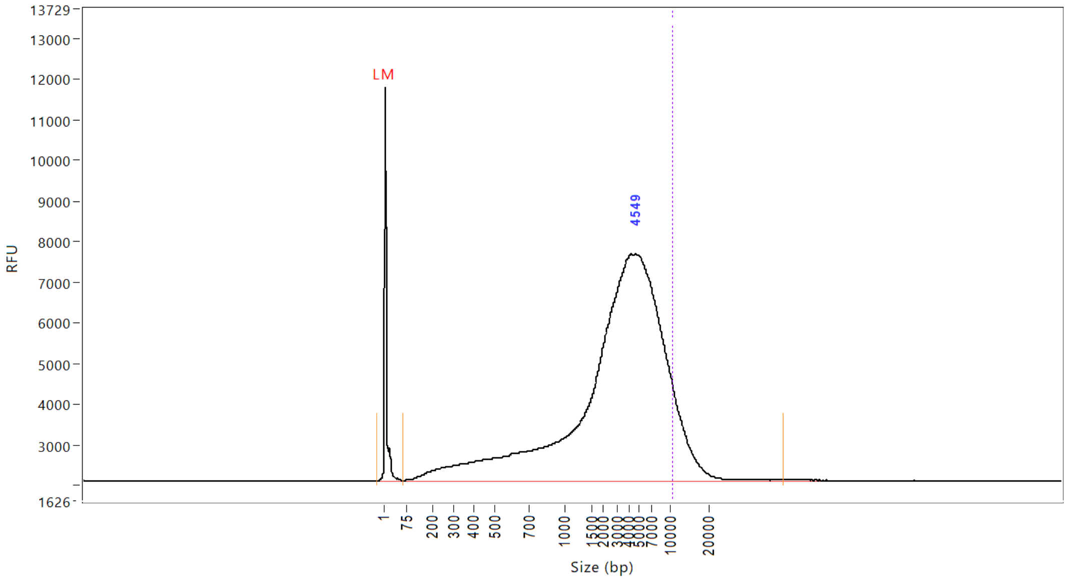

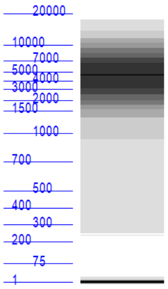

Peak	Size (bp)	Conc. (ng/uL)	From (bp)	To (bp)	Avg. Size (bp)	CV%	RFU	Corr. Peak Area
1	1 (LM)	0.0328	0	60	3	248.31	9673	68.696
2	4549	5.5618	60	39414	4161	89.85	5587	970.619

TIC:	5.5618	ng/uL
TIM:	2.2005	nmole/L
Total Conc.:	5.5820	ng/uL
GQN:	0.7	

Sample Peak Width (sec): 50   Sample Min Peak Height: 50   Sample Baseline V to V?: Y   Sample Baseline V to V pts: 3
Sample Filter: Binomial   # of Pts for Filter: 3   Sample Start Region (min): 0   Sample End Region (min): 50
Manual Baseline Start (min): 6   Manual Baseline End (min): 48
Marker Peak Width (sec): 5   Marker Min Peak Height: 200   Marker Baseline V to V?: Y   Marker Baseline V to V pts: 3
Lower Marker Selection: First Peak > 200 RFU   Upper Marker Selection: Last Peak > 200 RFU
Ladder Size (bp): 1, 75, 200, 300, 400, 500, 700, 1000, 1500, 2000, 3000, 4000, 5000, 7000, 10000, 20000
Quantification Using: Ladder   Final Concentration (ng/uL): 1.0417   Dilution Factor: 12.0
Size Threshold (b.p.): 10000

 Copyright 2015 Advanced Analytical Technologies, Inc.

Data File: 2019 06 18 13H 16M.raw
Sample: 103613-001-080 (20x dil.)
Well Location: H10

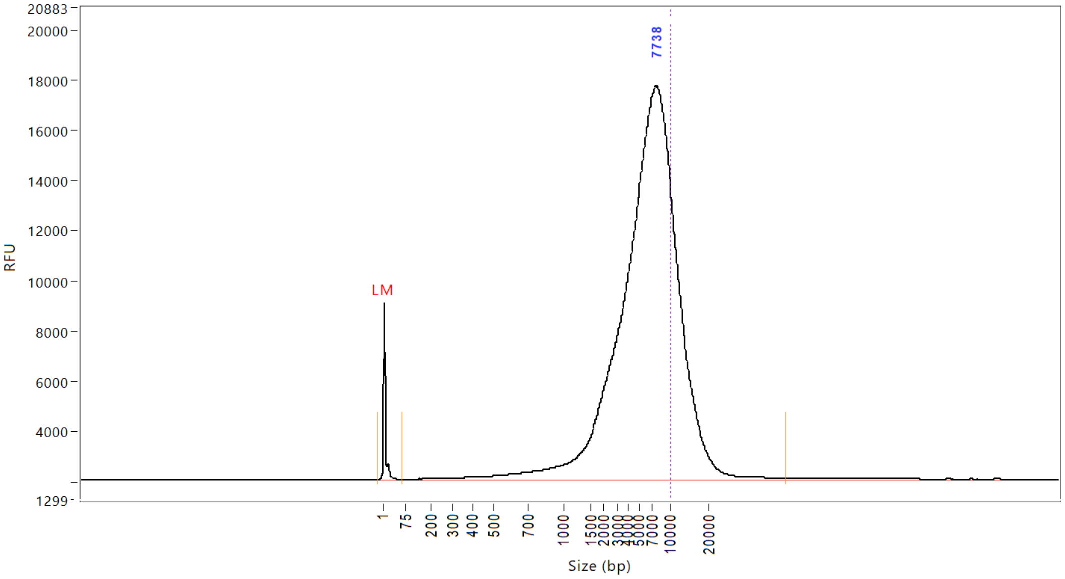

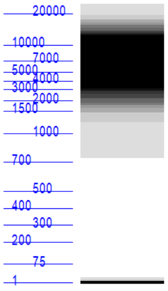

Peak	Size (bp)	Conc. (ng/uL)	From (bp)	To (bp)	Avg. Size (bp)	CV%	RFU	Corr. Peak Area
1	1 (LM)	0.0328	0	59	2	254.60	7033	48.964
2	7738	14.5603	59	40277	6925	62.86	15646	1811.145

TIC:	14.5603	ng/uL
TIM:	3.4615	nmole/L
Total Conc.:	14.6748	ng/uL
GQN:	1.9	

Sample Peak Width (sec): 50 Sample Min Peak Height: 50 Sample Baseline V to V?: Y Sample Baseline V to V pts: 3
Sample Filter: Binomial # of Pts for Filter: 3 Sample Start Region (min): 0 Sample End Region (min): 50
Manual Baseline Start (min): 6 Manual Baseline End (min): 48
Marker Peak Width (sec): 5 Marker Min Peak Height: 200 Marker Baseline V to V?: Y Marker Baseline V to V pts: 3
Lower Marker Selection: First Peak > 200 RFU Upper Marker Selection: Last Peak > 200 RFU
Ladder Size (bp): 1, 75, 200, 300, 400, 500, 700, 1000, 1500, 2000, 3000, 4000, 5000, 7000, 10000, 20000
Quantification Using: Ladder Final Concentration (ng/uL): 1.0417 Dilution Factor: 12.0
Size Threshold (b.p.): 10000

 Copyright 2015 Advanced Analytical Technologies, Inc.

Data File: 2019 06 18 13H 16M.raw
Sample: 103613-001-081 (20x dil.)
Well Location: A11

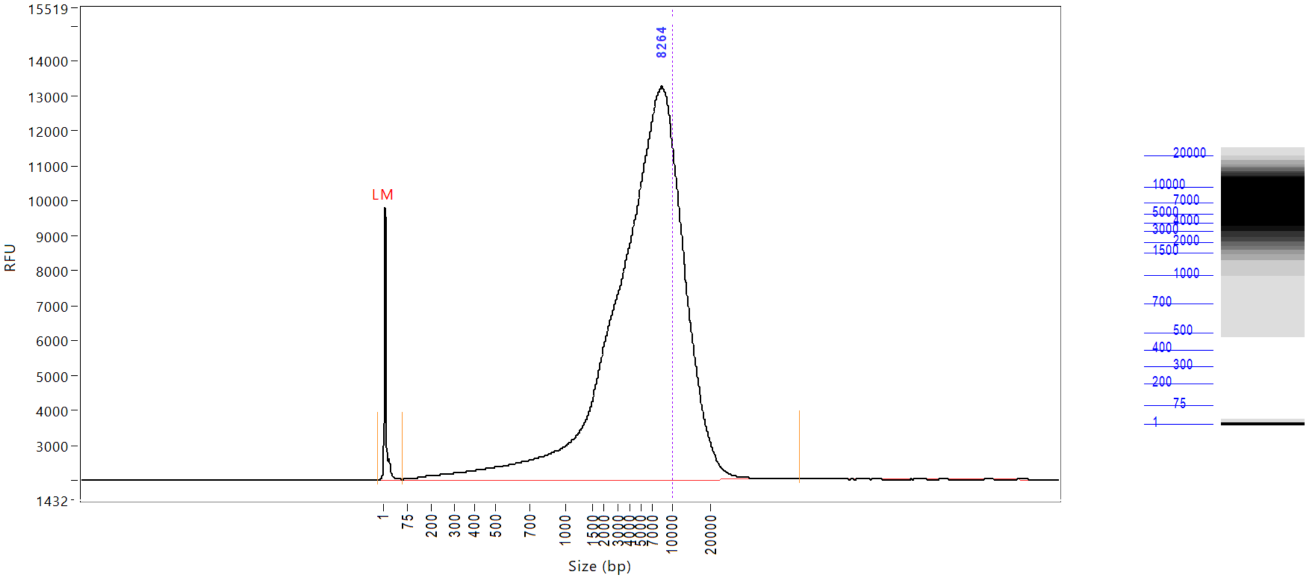

Peak	Size (bp)	Conc. (ng/uL)	From (bp)	To (bp)	Avg. Size (bp)	CV%	RFU	Corr. Peak Area
1	1 (LM)	0.0328	0	55	2	280.31	7782	53.318
2	8264	12.0904	55	43124	6539	71.34	11243	1637.631

TIC:	12.0904	ng/uL
TIM:	3.0439	nmole/L
Total Conc.:	12.1026	ng/uL
GQN:	2.0	

Sample Peak Width (sec): 50 Sample Min Peak Height: 50 Sample Baseline V to V?: Y Sample Baseline V to V pts: 3
Sample Filter: Binomial # of Pts for Filter: 3 Sample Start Region (min): 0 Sample End Region (min): 50
Manual Baseline Start (min): 6 Manual Baseline End (min): 48
Marker Peak Width (sec): 5 Marker Min Peak Height: 200 Marker Baseline V to V?: Y Marker Baseline V to V pts: 3
Lower Marker Selection: First Peak > 200 RFU Upper Marker Selection: Last Peak > 200 RFU
Ladder Size (bp): 1, 75, 200, 300, 400, 500, 700, 1000, 1500, 2000, 3000, 4000, 5000, 7000, 10000, 20000
Quantification Using: Ladder Final Concentration (ng/uL): 1.0417 Dilution Factor: 12.0
Size Threshold (b.p.): 10000

 Copyright 2015 Advanced Analytical Technologies, Inc.

Data File: 2019 06 18 13H 16M.raw
Sample: 103613-001-082 (20x dil.)
Well Location: B11

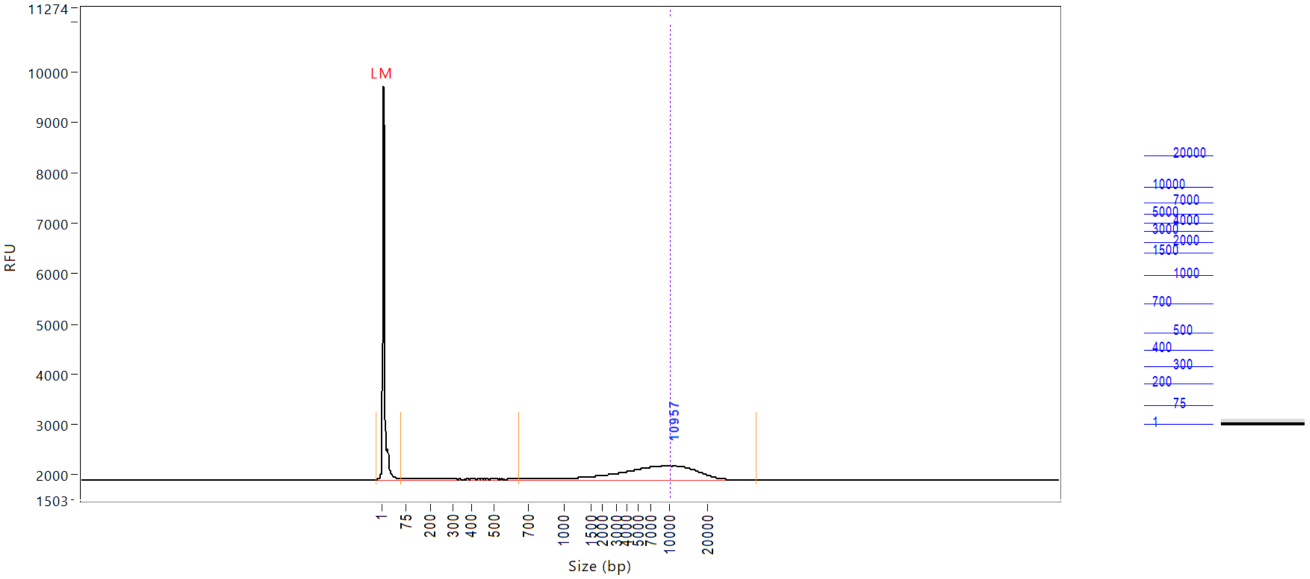

Peak	Size (bp)	Conc. (ng/uL)	From (bp)	To (bp)	Avg. Size (bp)	CV%	RFU	Corr. Peak Area
1	1 (LM)	0.0328	0	55	2	371.45	7811	55.244
2	10957	0.3598	643	32943	8092	69.53	279	50.491

TIC:	0.3598	ng/uL
TIM:	0.0732	nmole/L
Total Conc.:	0.4116	ng/uL
GQN:	2.9	

Sample Peak Width (sec): 50 Sample Min Peak Height: 50 Sample Baseline V to V?: Y Sample Baseline V to V pts: 3
Sample Filter: Binomial # of Pts for Filter: 3 Sample Start Region (min): 0 Sample End Region (min): 50
Manual Baseline Start (min): 6 Manual Baseline End (min): 48
Marker Peak Width (sec): 5 Marker Min Peak Height: 200 Marker Baseline V to V?: Y Marker Baseline V to V pts: 3
Lower Marker Selection: First Peak > 200 RFU Upper Marker Selection: Last Peak > 200 RFU
Ladder Size (bp): 1, 75, 200, 300, 400, 500, 700, 1000, 1500, 2000, 3000, 4000, 5000, 7000, 10000, 20000
Quantification Using: Ladder Final Concentration (ng/uL): 1.0417 Dilution Factor: 12.0
Size Threshold (b.p.): 10000

 Copyright 2015 Advanced Analytical Technologies, Inc.

Data File: 2019 06 18 13H 16M.raw
Sample: 103613-001-083 (20x dil.)
Well Location: C11

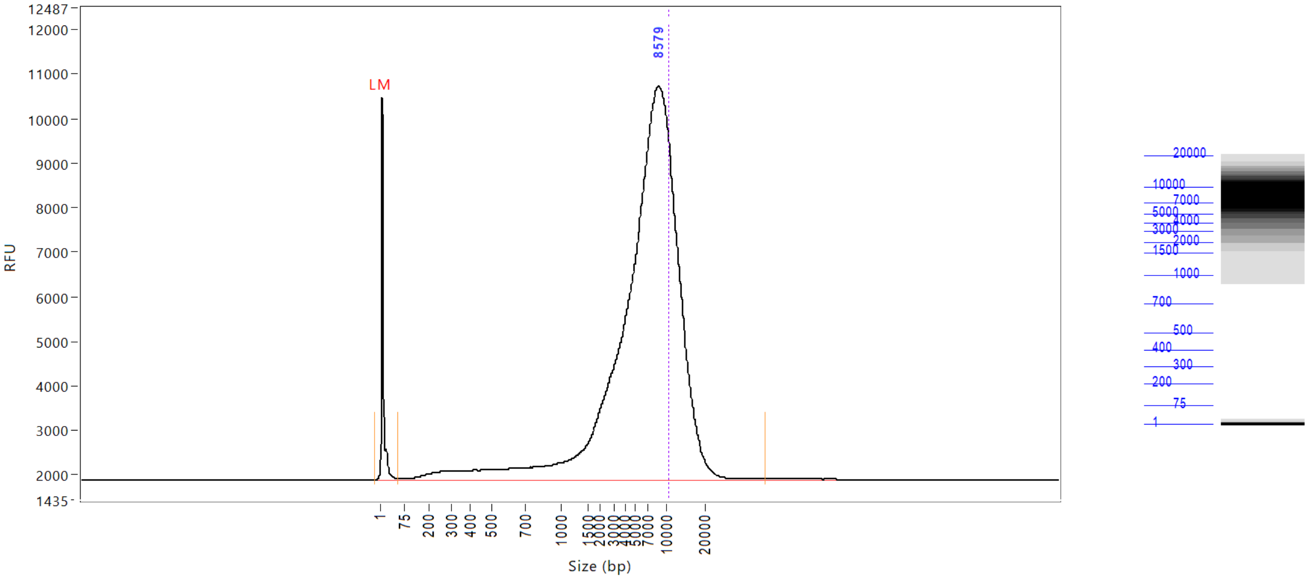

Peak	Size (bp)	Conc. (ng/uL)	From (bp)	To (bp)	Avg. Size (bp)	CV%	RFU	Corr. Peak Area
1	1 (LM)	0.0328	0	54	3	283.33	8597	60.022
2	8579	6.7014	54	35790	7069	61.73	8831	1021.830

TIC:	6.7014	ng/uL
TIM:	1.5607	nmole/L
Total Conc.:	6.7278	ng/uL
GQN:	2.2	

Sample Peak Width (sec): 50   Sample Min Peak Height: 50   Sample Baseline V to V?: Y   Sample Baseline V to V pts: 3
Sample Filter: Binomial   # of Pts for Filter: 3   Sample Start Region (min): 0   Sample End Region (min): 50
Manual Baseline Start (min): 6   Manual Baseline End (min): 48
Marker Peak Width (sec): 5   Marker Min Peak Height: 200   Marker Baseline V to V?: Y   Marker Baseline V to V pts: 3
Lower Marker Selection: First Peak > 200 RFU   Upper Marker Selection: Last Peak > 200 RFU
Ladder Size (bp): 1, 75, 200, 300, 400, 500, 700, 1000, 1500, 2000, 3000, 4000, 5000, 7000, 10000, 20000
Quantification Using: Ladder   Final Concentration (ng/uL): 1.0417   Dilution Factor: 12.0
Size Threshold (b.p.): 10000

Copyright 2015 Advanced Analytical Technologies, Inc.

Data File: 2019 06 18 13H 16M.raw
Sample: 103613-001-084 (20x dil.)
Well Location: D11

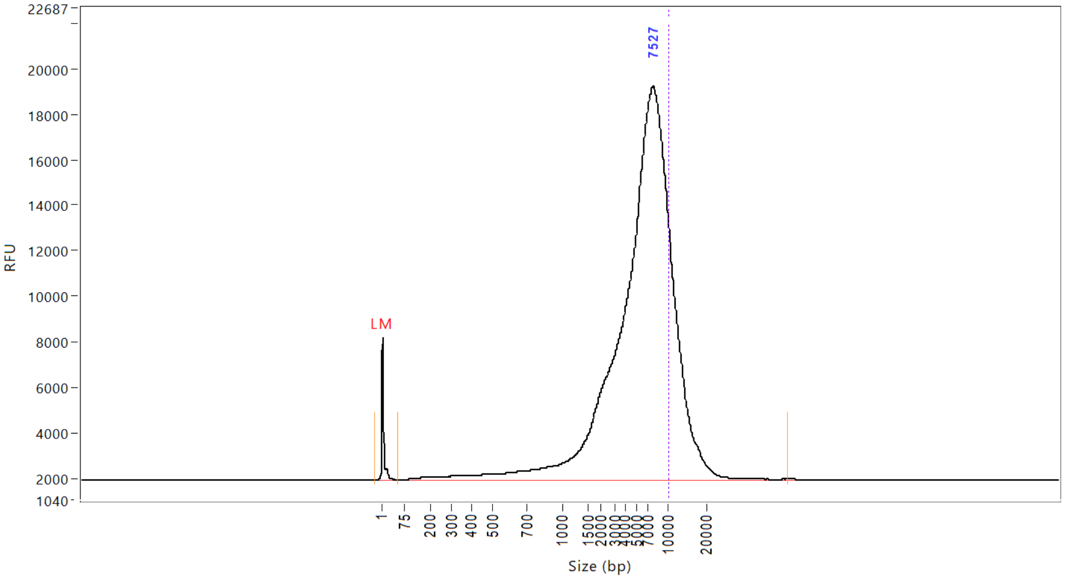

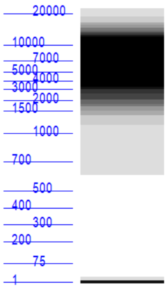

Peak	Size (bp)	Conc. (ng/uL)	From (bp)	To (bp)	Avg. Size (bp)	CV%	RFU	Corr. Peak Area
1	1 (LM)	0.0328	0	52	2	315.00	6245	43.060
2	7527	16.7949	52	41485	6567	61.80	17300	1837.206

TIC:	16.7949	ng/uL
TIM:	4.2104	nmole/L
Total Conc.:	16.8271	ng/uL
GQN:	1.6	

```
Sample Peak Width (sec): 50   Sample Min Peak Height: 50    Sample Baseline V to V?: Y    Sample Baseline V to V pts: 3
Sample Filter: Binomial       # of Pts for Filter: 3        Sample Start Region (min): 0  Sample End Region (min): 50
Manual Baseline Start (min): 6     Manual Baseline End (min): 48
Marker Peak Width (sec): 5    Marker Min Peak Height: 200   Marker Baseline V to V?: Y    Marker Baseline V to V pts: 3
Lower Marker Selection: First Peak > 200 RFU                Upper Marker Selection: Last Peak > 200 RFU
Ladder Size (bp): 1, 75, 200, 300, 400, 500, 700, 1000, 1500, 2000, 3000, 4000, 5000, 7000, 10000, 20000
Quantification Using: Ladder          Final Concentration (ng/uL): 1.0417          Dilution Factor: 12.0
Size Threshold (b.p.): 10000
```


Copyright 2015 Advanced Analytical Technologies, Inc.

Data File: 2019 06 18 13H 16M.raw
Sample: 103613-001-085 (20x dil.)
Well Location: E11

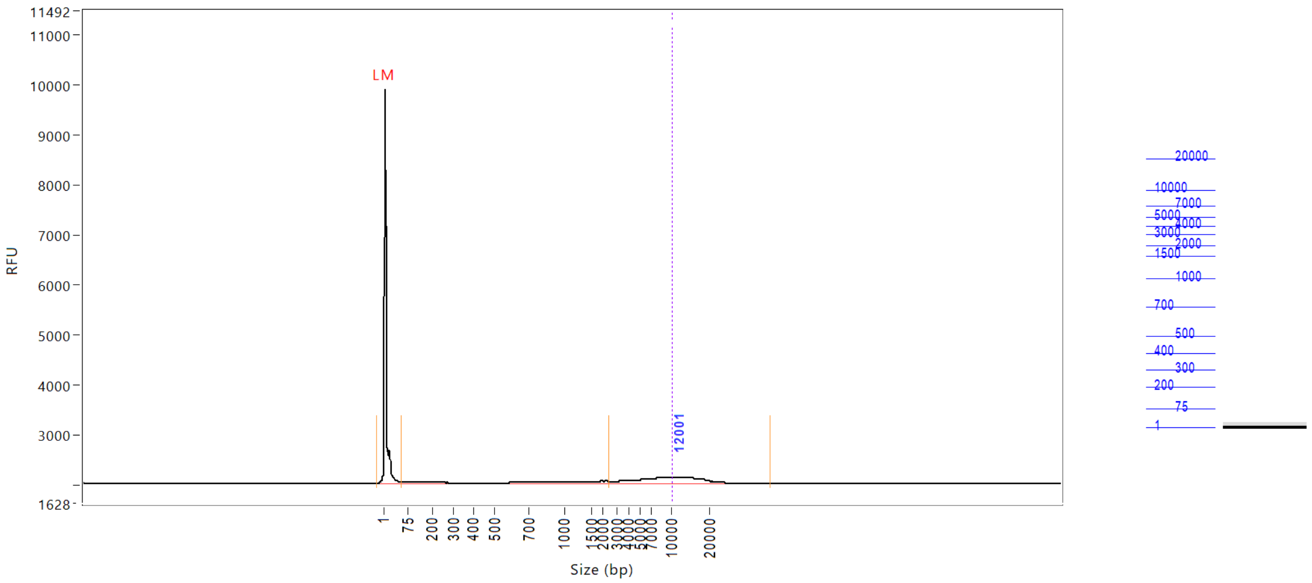

Peak	Size (bp)	Conc. (ng/uL)	From (bp)	To (bp)	Avg. Size (bp)	CV%	RFU	Corr. Peak Area
1	1 (LM)	0.0328	0	53	2	375.32	7881	55.810
2	12001	0.1329	2310	35963	10688	54.42	120	18.841

TIC:	0.1329	ng/uL
TIM:	0.0205	nmole/L
Total Conc.:	0.2048	ng/uL
GQN:	3.2	

Sample Peak Width (sec): 50 Sample Min Peak Height: 50 Sample Baseline V to V?: Y Sample Baseline V to V pts: 3
Sample Filter: Binomial # of Pts for Filter: 3 Sample Start Region (min): 0 Sample End Region (min): 50
Manual Baseline Start (min): 6 Manual Baseline End (min): 48
Marker Peak Width (sec): 5 Marker Min Peak Height: 200 Marker Baseline V to V?: Y Marker Baseline V to V pts: 3
Lower Marker Selection: First Peak > 200 RFU Upper Marker Selection: Last Peak > 200 RFU
Ladder Size (bp): 1, 75, 200, 300, 400, 500, 700, 1000, 1500, 2000, 3000, 4000, 5000, 7000, 10000, 20000
Quantification Using: Ladder Final Concentration (ng/uL): 1.0417 Dilution Factor: 12.0
Size Threshold (b.p.): 10000

 Copyright 2015 Advanced Analytical Technologies, Inc.

Data File: 2019 06 18 13H 16M.raw
Sample: 103613-001-086 (20x dil.)
Well Location: F11

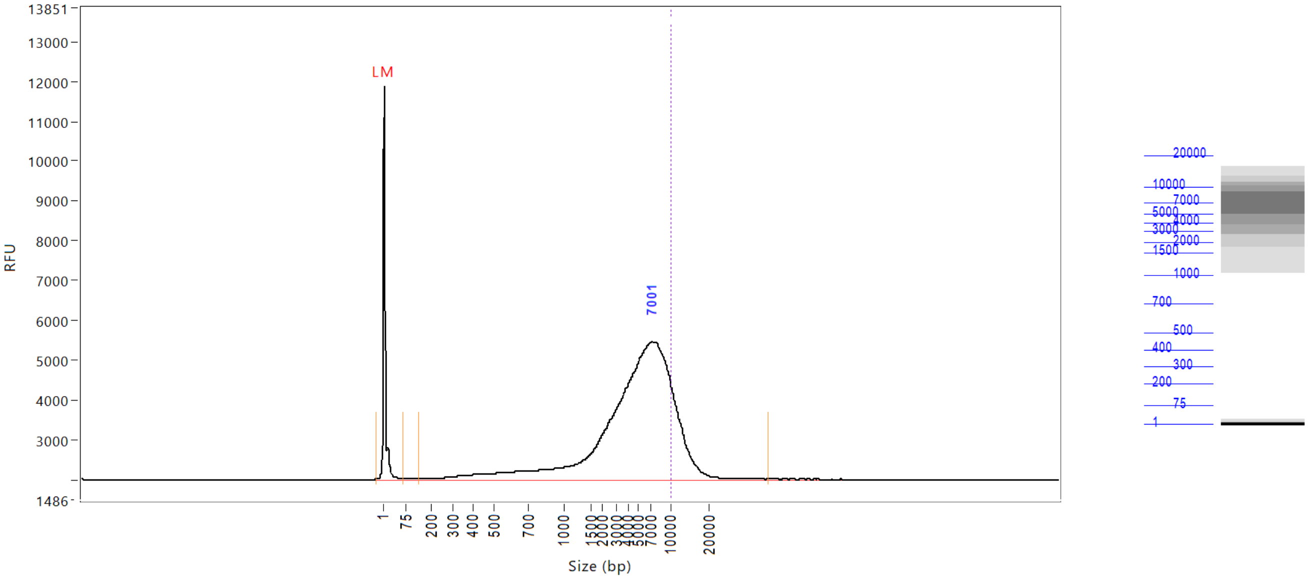

Peak	Size (bp)	Conc. (ng/uL)	From (bp)	To (bp)	Avg. Size (bp)	CV%	RFU	Corr. Peak Area
1	1 (LM)	0.0328	0	64	3	282.89	9874	69.253
2	7001	2.6811	139	35359	5711	71.43	3470	471.682

TIC:	2.6811	ng/uL
TIM:	0.7729	nmole/L
Total Conc.:	2.7210	ng/uL
GQN:	1.3	

Sample Peak Width (sec): 50 Sample Min Peak Height: 50 Sample Baseline V to V?: Y Sample Baseline V to V pts: 3
Sample Filter: Binomial # of Pts for Filter: 3 Sample Start Region (min): 0 Sample End Region (min): 50
Manual Baseline Start (min): 6 Manual Baseline End (min): 48
Marker Peak Width (sec): 5 Marker Min Peak Height: 200 Marker Baseline V to V?: Y Marker Baseline V to V pts: 3
Lower Marker Selection: First Peak > 200 RFU Upper Marker Selection: Last Peak > 200 RFU
Ladder Size (bp): 1, 75, 200, 300, 400, 500, 700, 1000, 1500, 2000, 3000, 4000, 5000, 7000, 10000, 20000
Quantification Using: Ladder Final Concentration (ng/uL): 1.0417 Dilution Factor: 12.0
Size Threshold (b.p.): 10000

 Copyright 2015 Advanced Analytical Technologies, Inc.

Data File: 2019 06 18 13H 16M.raw
Sample: 103613-001-087 (20x dil.)
Well Location: G11

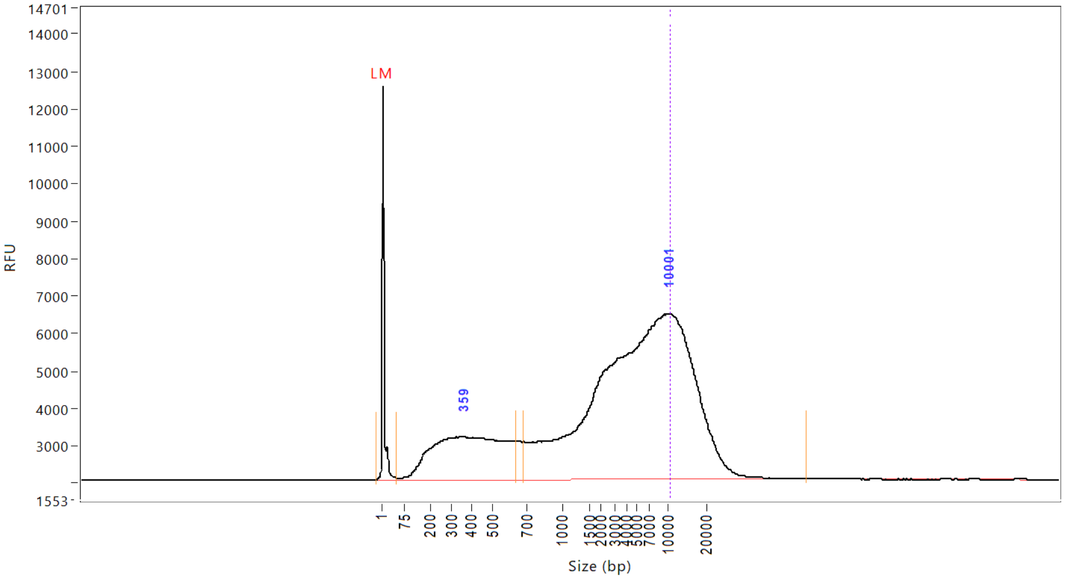

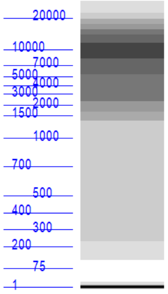

Peak	Size (bp)	Conc. (ng/uL)	From (bp)	To (bp)	Avg. Size (bp)	CV%	RFU	Corr. Peak Area
1	1 (LM)	0.0328	0	46	2	270.92	10501	72.250
2	359	1.4282	46	630	367	38.03	1120	262.129
3	10001	5.4513	671	46403	7061	84.19	4401	1000.550

TIC:	6.8794	ng/uL
TIM:	7.6731	nmole/L
Total Conc.:	6.9641	ng/uL
GQN:	2.0	

Sample Peak Width (sec): 50   Sample Min Peak Height: 50   Sample Baseline V to V?: Y   Sample Baseline V to V pts: 3
Sample Filter: Binomial   # of Pts for Filter: 3   Sample Start Region (min): 0   Sample End Region (min): 50
Manual Baseline Start (min): 6   Manual Baseline End (min): 48
Marker Peak Width (sec): 5   Marker Min Peak Height: 200   Marker Baseline V to V?: Y   Marker Baseline V to V pts: 3
Lower Marker Selection: First Peak > 200 RFU   Upper Marker Selection: Last Peak > 200 RFU
Ladder Size (bp): 1, 75, 200, 300, 400, 500, 700, 1000, 1500, 2000, 3000, 4000, 5000, 7000, 10000, 20000
Quantification Using: Ladder   Final Concentration (ng/uL): 1.0417   Dilution Factor: 12.0
Size Threshold (b.p.): 10000

 Copyright 2015 Advanced Analytical Technologies, Inc.

Data File: 2019 06 18 13H 16M.raw
Sample: 103613-001-088 (20x dil.)
Well Location: H11

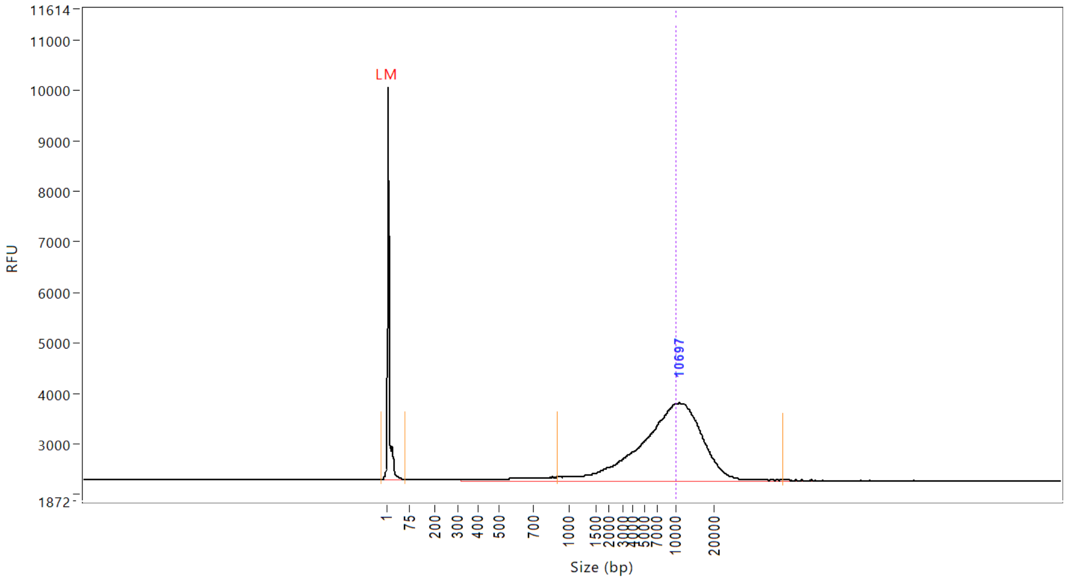

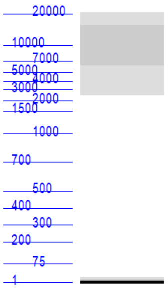

Peak	Size (bp)	Conc. (ng/uL)	From (bp)	To (bp)	Avg. Size (bp)	CV%	RFU	Corr. Peak Area
1	1 (LM)	0.0328	0	59	3	262.91	7772	55.043
2	10697	1.5150	902	37602	9451	56.88	1533	211.843

TIC:	1.5150	ng/uL
TIM:	0.2639	nmole/L
Total Conc.:	1.6095	ng/uL
GQN:	4.0	

Sample Peak Width (sec): 50   Sample Min Peak Height: 50   Sample Baseline V to V?: Y   Sample Baseline V to V pts: 3
Sample Filter: Binomial   # of Pts for Filter: 3   Sample Start Region (min): 0   Sample End Region (min): 50
Manual Baseline Start (min): 6   Manual Baseline End (min): 48
Marker Peak Width (sec): 5   Marker Min Peak Height: 200   Marker Baseline V to V?: Y   Marker Baseline V to V pts: 3
Lower Marker Selection: First Peak > 200 RFU   Upper Marker Selection: Last Peak > 200 RFU
Ladder Size (bp): 1, 75, 200, 300, 400, 500, 700, 1000, 1500, 2000, 3000, 4000, 5000, 7000, 10000, 20000
Quantification Using: Ladder   Final Concentration (ng/uL): 1.0417   Dilution Factor: 12.0
Size Threshold (b.p.): 10000

 Copyright 2015 Advanced Analytical Technologies, Inc.

Data File: 2019 06 18 13H 16M.raw
Sample: 103613-001-089 (20x dil.)
Well Location: A12

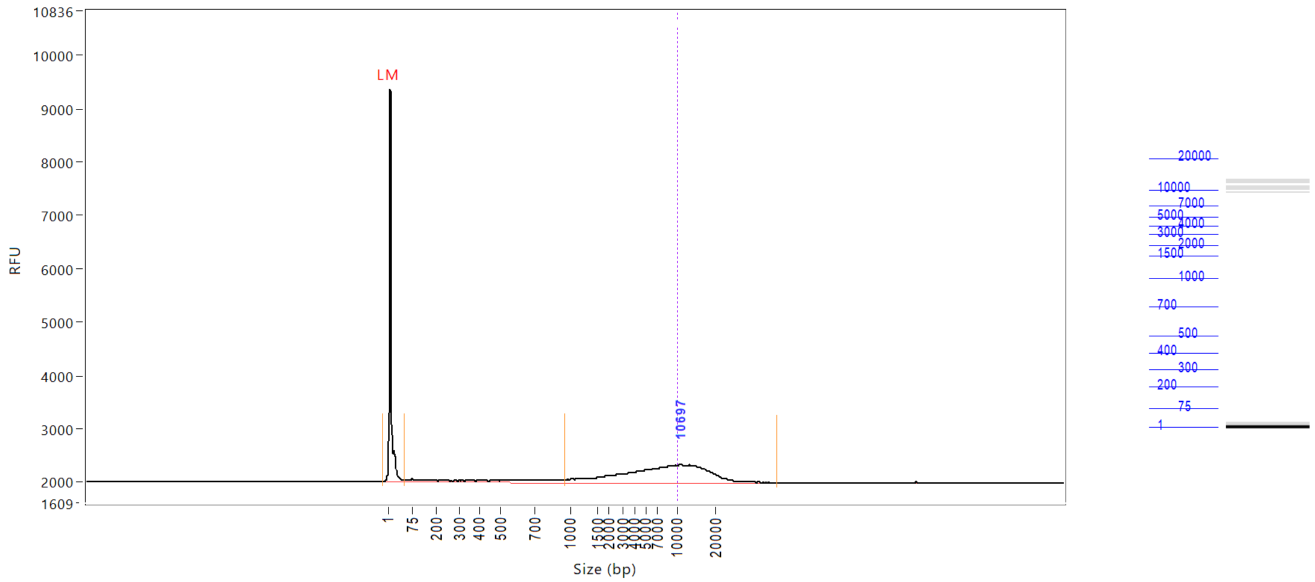

Peak	Size (bp)	Conc. (ng/uL)	From (bp)	To (bp)	Avg. Size (bp)	CV%	RFU	Corr. Peak Area
1	1 (LM)	0.0328	0	46	3	251.85	7360	52.402
2	10697	0.4756	944	35876	8872	69.25	335	63.314

TIC:	0.4756	ng/uL
TIM:	0.0883	nmole/L
Total Conc.:	0.5975	ng/uL
GQN:	3.1	

```
Sample Peak Width (sec): 50   Sample Min Peak Height: 50    Sample Baseline V to V?: Y    Sample Baseline V to V pts: 3
Sample Filter: Binomial       # of Pts for Filter: 3        Sample Start Region (min): 0  Sample End Region (min): 50
Manual Baseline Start (min): 6     Manual Baseline End (min): 48
Marker Peak Width (sec): 5    Marker Min Peak Height: 200   Marker Baseline V to V?: Y    Marker Baseline V to V pts: 3
Lower Marker Selection: First Peak > 200 RFU                Upper Marker Selection: Last Peak > 200 RFU
Ladder Size (bp): 1, 75, 200, 300, 400, 500, 700, 1000, 1500, 2000, 3000, 4000, 5000, 7000, 10000, 20000
Quantification Using: Ladder          Final Concentration (ng/uL): 1.0417          Dilution Factor: 12.0
Size Threshold (b.p.): 10000
```


Copyright 2015 Advanced Analytical Technologies, Inc.

Data File: 2019 06 18 13H 16M.raw
Sample: 103613-001-090 (20x dil.)
Well Location: B12

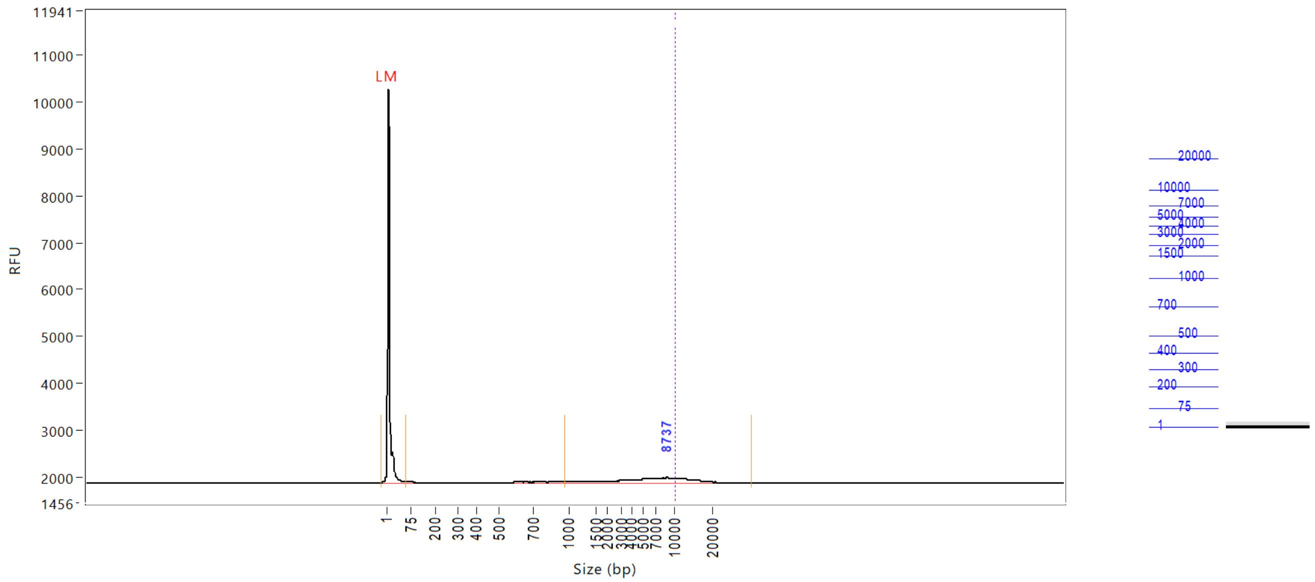

Peak	Size (bp)	Conc. (ng/uL)	From (bp)	To (bp)	Avg. Size (bp)	CV%	RFU	Corr. Peak Area
1	1 (LM)	0.0328	0	54	2	364.65	8384	59.169
2	8737	0.1167	969	30268	7501	69.37	111	17.540

TIC:	0.1167	ng/uL
TIM:	0.0256	nmole/L
Total Conc.:	0.1545	ng/uL
GQN:	2.1	

Sample Peak Width (sec): 50 Sample Min Peak Height: 50 Sample Baseline V to V?: Y Sample Baseline V to V pts: 3
Sample Filter: Binomial # of Pts for Filter: 3 Sample Start Region (min): 0 Sample End Region (min): 50
Manual Baseline Start (min): 6 Manual Baseline End (min): 48
Marker Peak Width (sec): 5 Marker Min Peak Height: 200 Marker Baseline V to V?: Y Marker Baseline V to V pts: 3
Lower Marker Selection: First Peak > 200 RFU Upper Marker Selection: Last Peak > 200 RFU
Ladder Size (bp): 1, 75, 200, 300, 400, 500, 700, 1000, 1500, 2000, 3000, 4000, 5000, 7000, 10000, 20000
Quantification Using: Ladder Final Concentration (ng/uL): 1.0417 Dilution Factor: 12.0
Size Threshold (b.p.): 10000

 Copyright 2015 Advanced Analytical Technologies, Inc.

Data File: 2019 06 18 13H 16M.raw
Sample: 103613-001-091 (20x dil.)
Well Location: C12

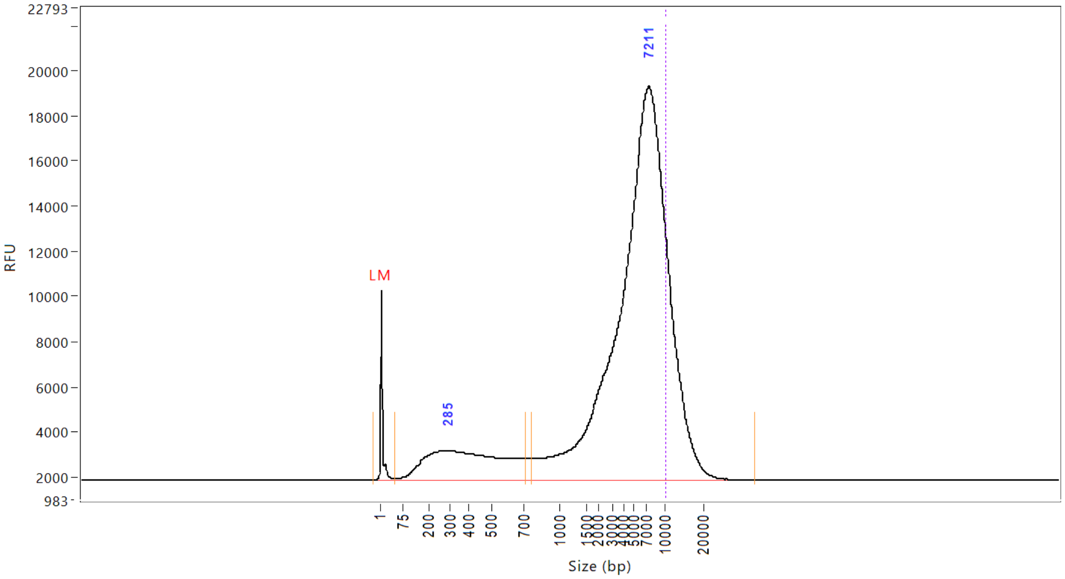

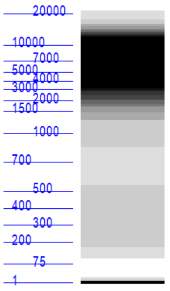

Peak	Size (bp)	Conc. (ng/uL)	From (bp)	To (bp)	Avg. Size (bp)	CV%	RFU	Corr. Peak Area
1	1 (LM)	0.0328	0	46	3	247.86	8387	59.915
2	285	2.0884	46	707	374	43.51	1274	317.868
3	7211	11.9762	751	33719	6441	57.91	17438	1822.872

TIC:	14.0646	ng/uL
TIM:	12.2478	nmole/L
Total Conc.:	14.1562	ng/uL
GQN:	1.2	

Sample Peak Width (sec): 50 Sample Min Peak Height: 50 Sample Baseline V to V?: Y Sample Baseline V to V pts: 3
Sample Filter: Binomial # of Pts for Filter: 3 Sample Start Region (min): 0 Sample End Region (min): 50
Manual Baseline Start (min): 6 Manual Baseline End (min): 48
Marker Peak Width (sec): 5 Marker Min Peak Height: 200 Marker Baseline V to V?: Y Marker Baseline V to V pts: 3
Lower Marker Selection: First Peak > 200 RFU Upper Marker Selection: Last Peak > 200 RFU
Ladder Size (bp): 1, 75, 200, 300, 400, 500, 700, 1000, 1500, 2000, 3000, 4000, 5000, 7000, 10000, 20000
Quantification Using: Ladder Final Concentration (ng/uL): 1.0417 Dilution Factor: 12.0
Size Threshold (b.p.): 10000

 Copyright 2015 Advanced Analytical Technologies, Inc.

Data File: 2019 06 18 13H 16M.raw
Sample: 103613-001-092 (20x dil.)
Well Location: D12

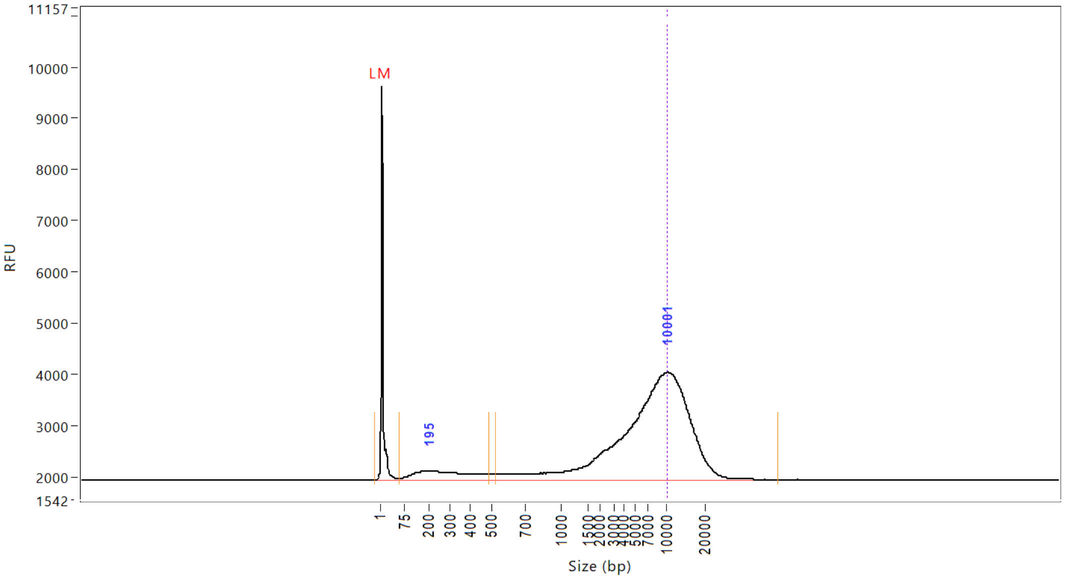

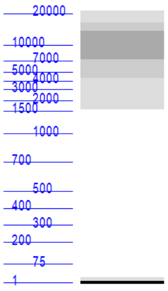

Peak	Size (bp)	Conc. (ng/uL)	From (bp)	To (bp)	Avg. Size (bp)	CV%	RFU	Corr. Peak Area
1	1 (LM)	0.0328	0	56	3	278.75	7687	53.677
2	195	0.2237	56	483	263	43.22	163	30.502
3	10001	2.2690	522	39155	8323	65.36	2112	309.404

TIC:	2.4927	ng/uL
TIM:	1.8477	nmole/L
Total Conc.:	2.5263	ng/uL
GQN:	3.1	

Sample Peak Width (sec): 50 Sample Min Peak Height: 50 Sample Baseline V to V?: Y Sample Baseline V to V pts: 3
Sample Filter: Binomial # of Pts for Filter: 3 Sample Start Region (min): 0 Sample End Region (min): 50
Manual Baseline Start (min): 6 Manual Baseline End (min): 48
Marker Peak Width (sec): 5 Marker Min Peak Height: 200 Marker Baseline V to V?: Y Marker Baseline V to V pts: 3
Lower Marker Selection: First Peak > 200 RFU Upper Marker Selection: Last Peak > 200 RFU
Ladder Size (bp): 1, 75, 200, 300, 400, 500, 700, 1000, 1500, 2000, 3000, 4000, 5000, 7000, 10000, 20000
Quantification Using: Ladder Final Concentration (ng/uL): 1.0417 Dilution Factor: 12.0
Size Threshold (b.p.): 10000

 Copyright 2015 Advanced Analytical Technologies, Inc.

Data File: 2019 06 18 13H 16M.raw
Sample: 103613-001-093 (20x dil.)
Well Location: E12

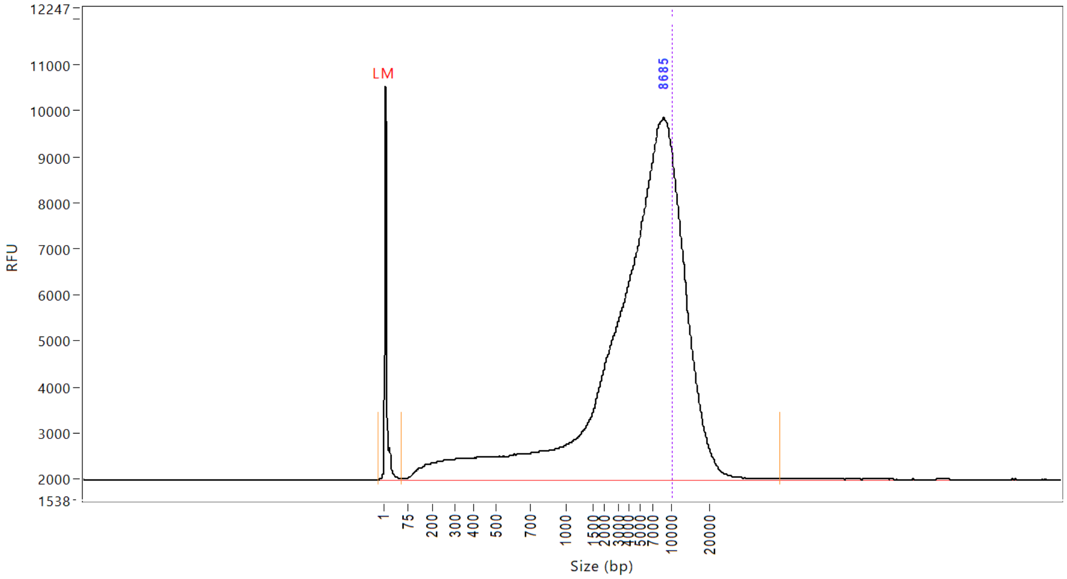

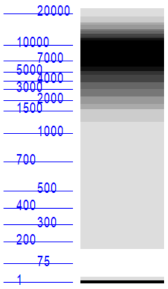

Peak	Size (bp)	Conc. (ng/uL)	From (bp)	To (bp)	Avg. Size (bp)	CV%	RFU	Corr. Peak Area
1	1 (LM)	0.0328	0	51	2	351.69	8558	59.862
2	8685	7.9380	51	38206	6170	77.84	7866	1207.153

TIC:	7.9380	ng/uL
TIM:	2.1180	nmole/L
Total Conc.:	7.9732	ng/uL
GQN:	2.0	

Sample Peak Width (sec): 50 Sample Min Peak Height: 50 Sample Baseline V to V?: Y Sample Baseline V to V pts: 3
Sample Filter: Binomial # of Pts for Filter: 3 Sample Start Region (min): 0 Sample End Region (min): 50
Manual Baseline Start (min): 6 Manual Baseline End (min): 48
Marker Peak Width (sec): 5 Marker Min Peak Height: 200 Marker Baseline V to V?: Y Marker Baseline V to V pts: 3
Lower Marker Selection: First Peak > 200 RFU Upper Marker Selection: Last Peak > 200 RFU
Ladder Size (bp): 1, 75, 200, 300, 400, 500, 700, 1000, 1500, 2000, 3000, 4000, 5000, 7000, 10000, 20000
Quantification Using: Ladder Final Concentration (ng/uL): 1.0417 Dilution Factor: 12.0
Size Threshold (b.p.): 10000

 Copyright 2015 Advanced Analytical Technologies, Inc.

Data File: 2019 06 18 13H 16M.raw
Sample: 103613-001-094 (20x dil.)
Well Location: F12

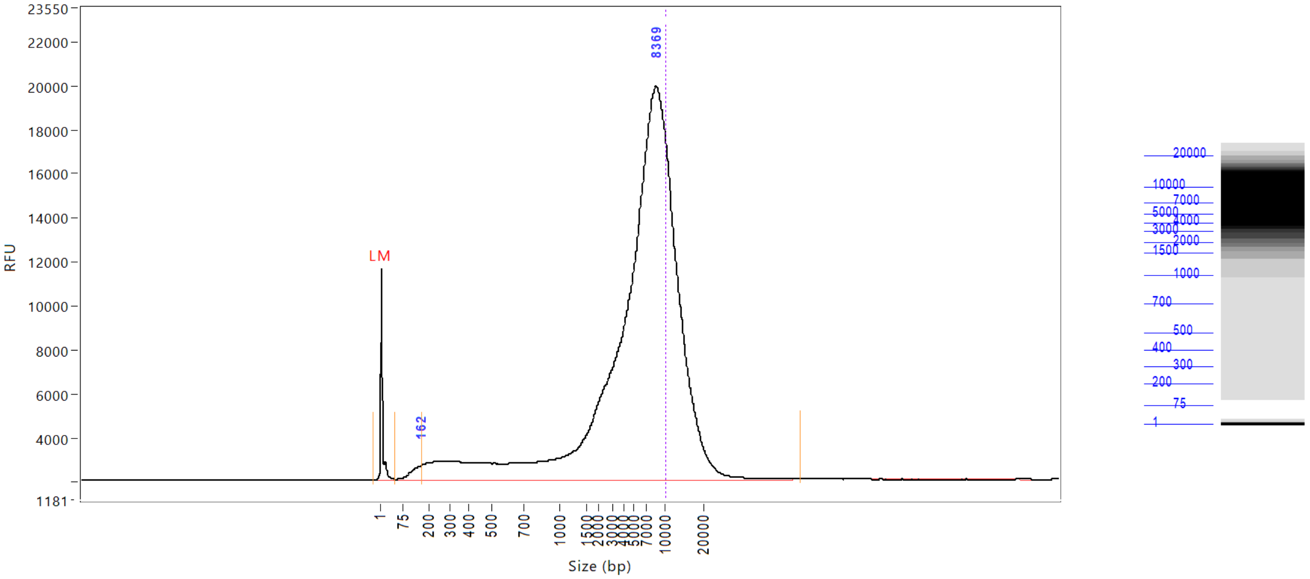

Peak	Size (bp)	Conc. (ng/uL)	From (bp)	To (bp)	Avg. Size (bp)	CV%	RFU	Corr. Peak Area
1	1 (LM)	0.0328	0	46	3	244.55	9594	67.293
2	162	0.1396	46	164	125	22.29	693	23.873
3	8369	13.0397	164	45713	6912	70.60	17859	2229.174

TIC:	13.1794	ng/uL
TIM:	4.9413	nmole/L
Total Conc.:	13.1890	ng/uL
GQN:	2.2	

Sample Peak Width (sec): 50 Sample Min Peak Height: 50 Sample Baseline V to V?: Y Sample Baseline V to V pts: 3
Sample Filter: Binomial # of Pts for Filter: 3 Sample Start Region (min): 0 Sample End Region (min): 50
Manual Baseline Start (min): 6 Manual Baseline End (min): 48
Marker Peak Width (sec): 5 Marker Min Peak Height: 200 Marker Baseline V to V?: Y Marker Baseline V to V pts: 3
Lower Marker Selection: First Peak > 200 RFU Upper Marker Selection: Last Peak > 200 RFU
Ladder Size (bp): 1, 75, 200, 300, 400, 500, 700, 1000, 1500, 2000, 3000, 4000, 5000, 7000, 10000, 20000
Quantification Using: Ladder Final Concentration (ng/uL): 1.0417 Dilution Factor: 12.0
Size Threshold (b.p.): 10000

 Copyright 2015 Advanced Analytical Technologies, Inc.

Data File: 2019 06 18 13H 16M.raw
Sample: 103613-001-095 (20x dil.)
Well Location: G12

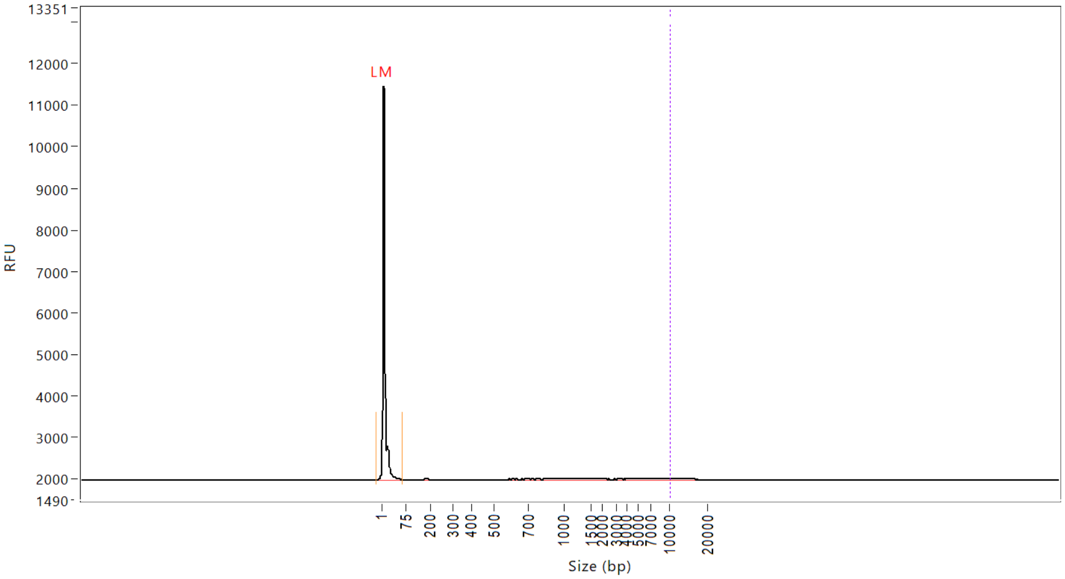

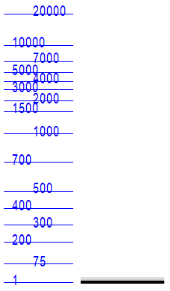

Peak	Size (bp)	Conc. (ng/uL)	From (bp)	To (bp)	Avg. Size (bp)	CV%	RFU	Corr. Peak Area
1	1 (LM)	0.0328	0	62	3	253.78	9481	67.743

TIC:	0.0000	ng/uL
TIM:	0.0000	nmole/L
Total Conc.:	0.0780	ng/uL
GQN:	2.4	

```
Sample Peak Width (sec): 50   Sample Min Peak Height: 50    Sample Baseline V to V?: Y    Sample Baseline V to V pts: 3
Sample Filter: Binomial       # of Pts for Filter: 3        Sample Start Region (min): 0  Sample End Region (min): 50
Manual Baseline Start (min): 6     Manual Baseline End (min): 48
Marker Peak Width (sec): 5    Marker Min Peak Height: 200   Marker Baseline V to V?: Y    Marker Baseline V to V pts: 3
Lower Marker Selection: First Peak > 200 RFU                Upper Marker Selection: Last Peak > 200 RFU
Ladder Size (bp): 1, 75, 200, 300, 400, 500, 700, 1000, 1500, 2000, 3000, 4000, 5000, 7000, 10000, 20000
Quantification Using: Ladder          Final Concentration (ng/uL): 1.0417          Dilution Factor: 12.0
Size Threshold (b.p.): 10000
```

 Copyright 2015 Advanced Analytical Technologies, Inc.

Data File: 2019 06 18 13H 16M.raw
Sample: 103613-001-096 (20x dil.)
Well Location: H12

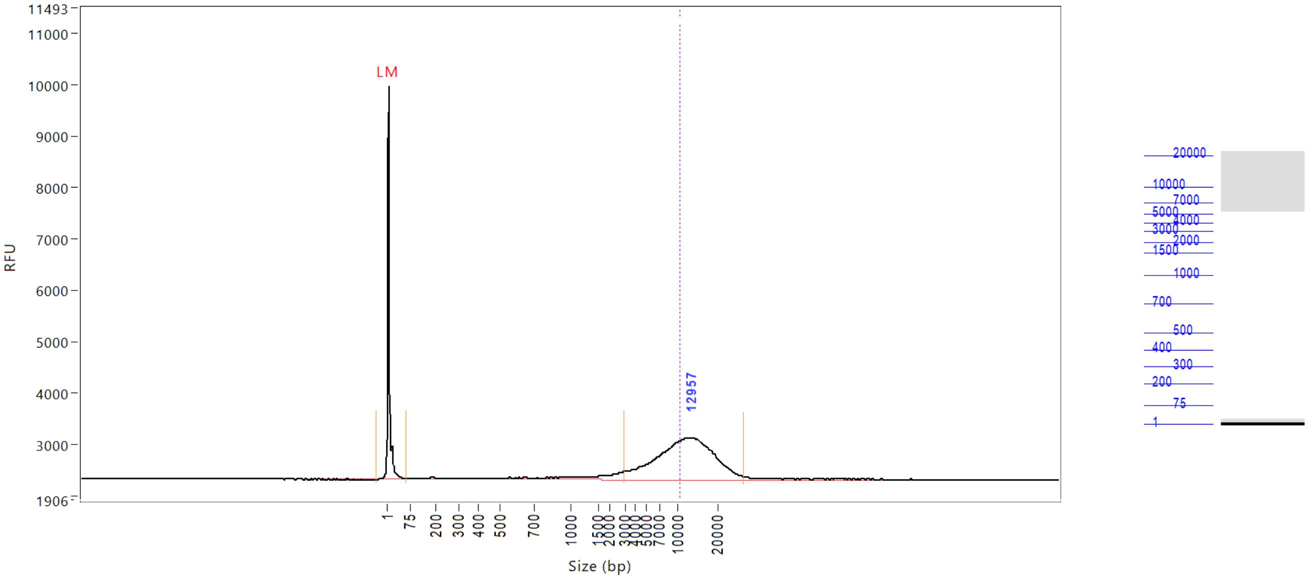

Peak	Size (bp)	Conc. (ng/uL)	From (bp)	To (bp)	Avg. Size (bp)	CV%	RFU	Corr. Peak Area
1	1 (LM)	0.0328	0	59	2	292.54	7644	52.892
2	12957	0.8468	2976	26816	12000	44.47	807	113.785

TIC:	0.8468	ng/uL
TIM:	0.1162	nmole/L
Total Conc.:	0.9735	ng/uL

Sample Peak Width (sec): 8 Sample Min Peak Height: 200 Sample Baseline V to V?: Y Sample Baseline V to V pts: 3
Sample Filter: Binomial # of Pts for Filter: 3 Sample Start Region (min): 0 Sample End Region (min): 50
Manual Baseline Start (min): 6 Manual Baseline End (min): 48
Marker Peak Width (sec): 5 Marker Min Peak Height: 200 Marker Baseline V to V?: Y Marker Baseline V to V pts: 3
Lower Marker Selection: First Peak > 200 RFU Upper Marker Selection: Last Peak > 200 RFU
Ladder Size (bp): 1, 75, 200, 300, 400, 500, 700, 1000, 1500, 2000, 3000, 4000, 5000, 7000, 10000, 20000
Quantification Using: Ladder Final Concentration (ng/uL): 1.0417 Dilution Factor: 12.0
Size Threshold (b.p.): 10000

 Copyright 2015 Advanced Analytical Technologies, Inc.

Data File: 2019 06 18 14H 35M.raw
Sample: 103613-001-097 (20x dil.)
Well Location: A1

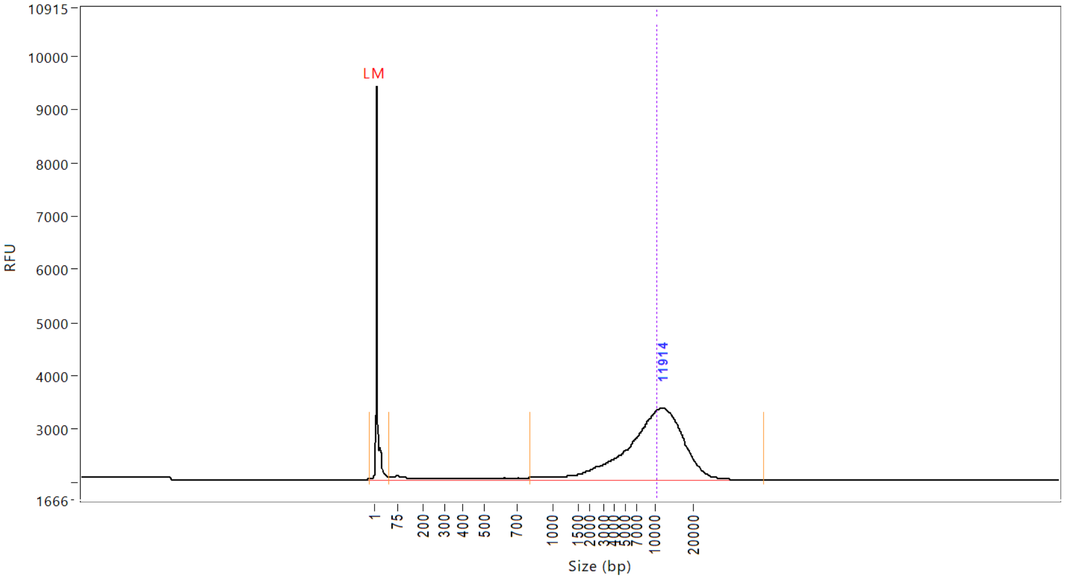

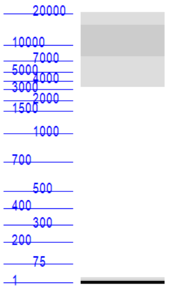

Peak	Size (bp)	Conc. (ng/uL)	From (bp)	To (bp)	Avg. Size (bp)	CV%	RFU	Corr. Peak Area
1	1 (LM)	0.0328	0	46	3	249.21	7390	53.009
2	11914	1.3483	807	38896	10067	54.80	1346	181.573

TIC:	1.3483	ng/uL
TIM:	0.2205	nmole/L
Total Conc.:	1.4657	ng/uL
GQN:	4.3	

Sample Peak Width (sec): 50 Sample Min Peak Height: 50 Sample Baseline V to V?: Y Sample Baseline V to V pts: 3
Sample Filter: Binomial # of Pts for Filter: 3 Sample Start Region (min): 0 Sample End Region (min): 50
Manual Baseline Start (min): 6 Manual Baseline End (min): 48
Marker Peak Width (sec): 5 Marker Min Peak Height: 200 Marker Baseline V to V?: Y Marker Baseline V to V pts: 3
Lower Marker Selection: First Peak > 200 RFU Upper Marker Selection: Last Peak > 200 RFU
Ladder Size (bp): 1, 75, 200, 300, 400, 500, 700, 1000, 1500, 2000, 3000, 4000, 5000, 7000, 10000, 20000
Quantification Using: Ladder Final Concentration (ng/uL): 1.0417 Dilution Factor: 12.0
Size Threshold (b.p.): 10000

 Copyright 2015 Advanced Analytical Technologies, Inc.

Data File: 2019 06 18 14H 35M.raw
Sample: 103613-001-098 (20x dil.)
Well Location: B1

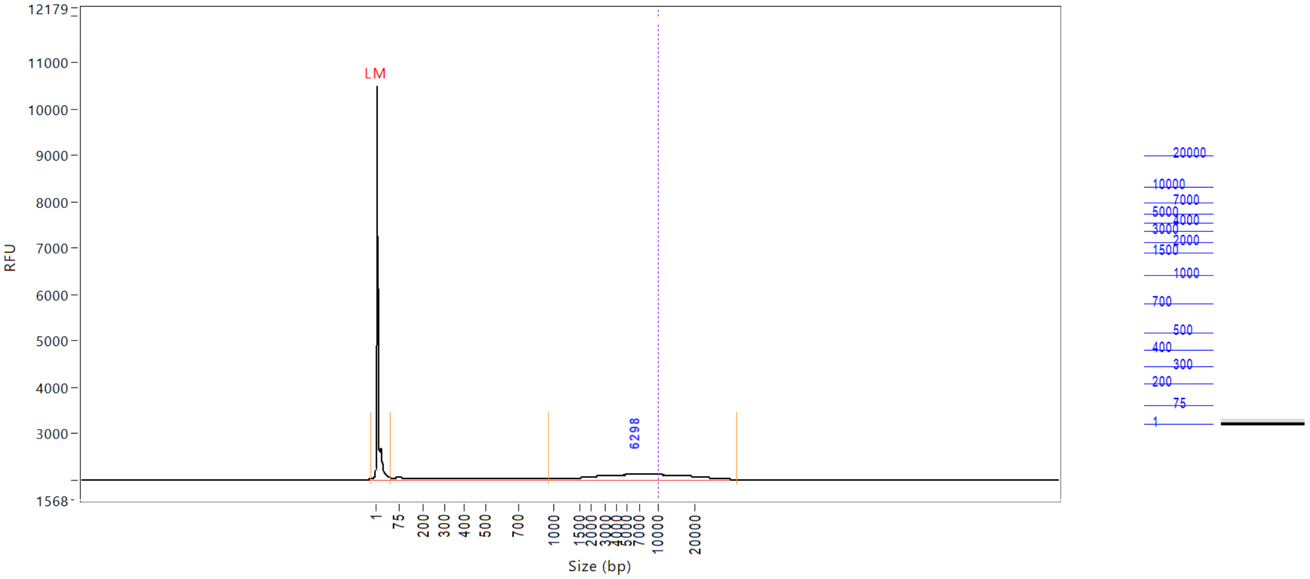

Peak	Size (bp)	Conc. (ng/uL)	From (bp)	To (bp)	Avg. Size (bp)	CV%	RFU	Corr. Peak Area
1	1 (LM)	0.0328	0	47	2	310.49	8478	58.950
2	6298	0.1938	950	31217	9011	78.47	123	29.023

TIC:	0.1938	ng/uL
TIM:	0.0354	nmole/L
Total Conc.:	0.3066	ng/uL
GQN:	2.6	

Sample Peak Width (sec): 50 Sample Min Peak Height: 50 Sample Baseline V to V?: Y Sample Baseline V to V pts: 3
Sample Filter: Binomial # of Pts for Filter: 3 Sample Start Region (min): 0 Sample End Region (min): 50
Manual Baseline Start (min): 6 Manual Baseline End (min): 48
Marker Peak Width (sec): 5 Marker Min Peak Height: 200 Marker Baseline V to V?: Y Marker Baseline V to V pts: 3
Lower Marker Selection: First Peak > 200 RFU Upper Marker Selection: Last Peak > 200 RFU
Ladder Size (bp): 1, 75, 200, 300, 400, 500, 700, 1000, 1500, 2000, 3000, 4000, 5000, 7000, 10000, 20000
Quantification Using: Ladder Final Concentration (ng/uL): 1.0417 Dilution Factor: 12.0
Size Threshold (b.p.): 10000

Copyright 2015 Advanced Analytical Technologies, Inc.

Data File: 2019 06 18 14H 35M.raw
Sample: 103613-001-099 (20x dil.)
Well Location: C1

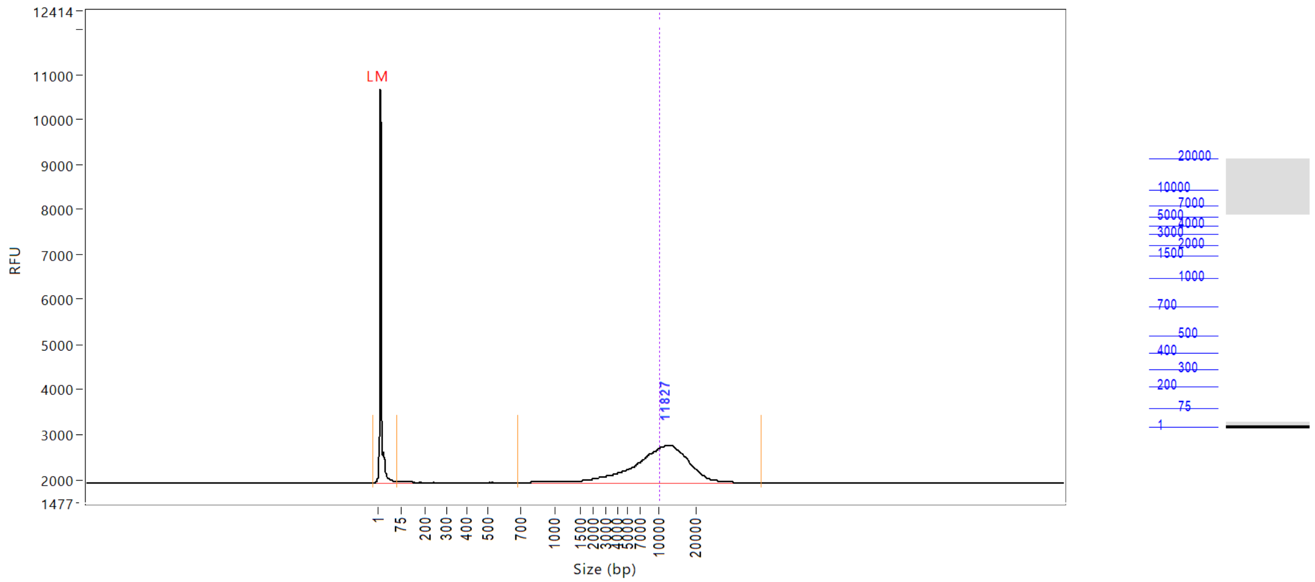

Peak	Size (bp)	Conc. (ng/uL)	From (bp)	To (bp)	Avg. Size (bp)	CV%	RFU	Corr. Peak Area
1	1 (LM)	0.0328	0	57	2	336.98	8747	61.198
2	11827	0.7416	688	38034	10778	54.18	829	115.298

TIC:	0.7416	ng/uL
TIM:	0.1133	nmole/L
Total Conc.:	0.7962	ng/uL
GQN:	4.9	

Sample Peak Width (sec): 50   Sample Min Peak Height: 50   Sample Baseline V to V?: Y   Sample Baseline V to V pts: 3
Sample Filter: Binomial   # of Pts for Filter: 3   Sample Start Region (min): 0   Sample End Region (min): 50
Manual Baseline Start (min): 6   Manual Baseline End (min): 48
Marker Peak Width (sec): 5   Marker Min Peak Height: 200   Marker Baseline V to V?: Y   Marker Baseline V to V pts: 3
Lower Marker Selection: First Peak > 200 RFU   Upper Marker Selection: Last Peak > 200 RFU
Ladder Size (bp): 1, 75, 200, 300, 400, 500, 700, 1000, 1500, 2000, 3000, 4000, 5000, 7000, 10000, 20000
Quantification Using: Ladder   Final Concentration (ng/uL): 1.0417   Dilution Factor: 12.0
Size Threshold (b.p.): 10000

 Copyright 2015 Advanced Analytical Technologies, Inc.

Data File: 2019 06 18 14H 35M.raw
Sample: 103613-001-100 (20x dil.)
Well Location: D1

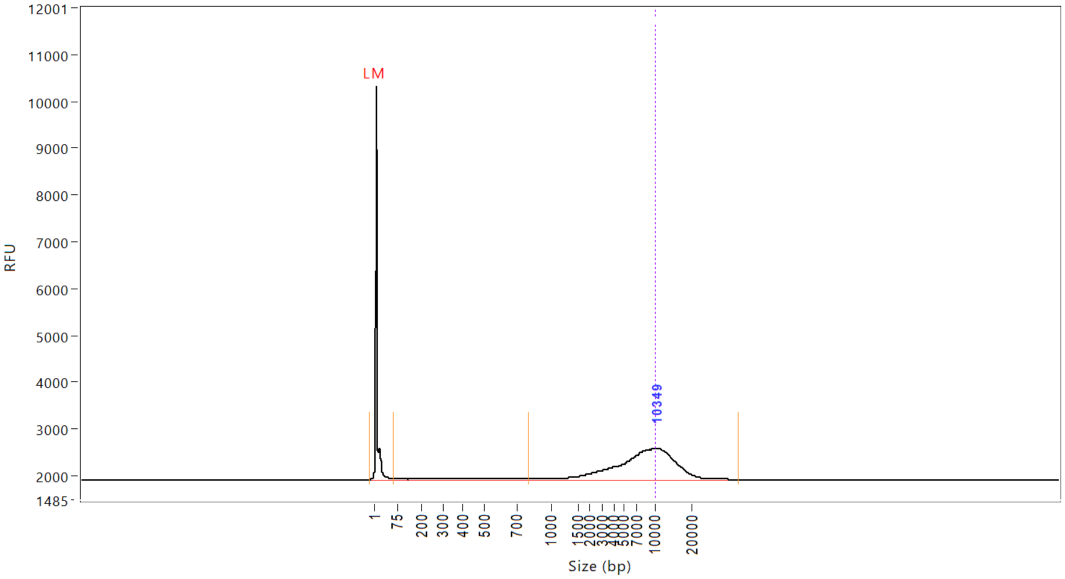

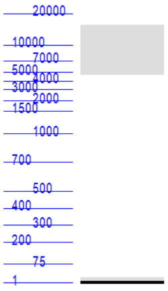

Peak	Size (bp)	Conc. (ng/uL)	From (bp)	To (bp)	Avg. Size (bp)	CV%	RFU	Corr. Peak Area
1	1 (LM)	0.0328	0	57	2	343.07	8408	58.824
2	10349	0.5912	804	32080	8714	58.62	674	88.353

TIC:	0.5912	ng/uL
TIM:	0.1117	nmole/L
Total Conc.:	0.6646	ng/uL
GQN:	3.3	

Sample Peak Width (sec): 50 Sample Min Peak Height: 50 Sample Baseline V to V?: Y Sample Baseline V to V pts: 3
Sample Filter: Binomial # of Pts for Filter: 3 Sample Start Region (min): 0 Sample End Region (min): 50
Manual Baseline Start (min): 6 Manual Baseline End (min): 48
Marker Peak Width (sec): 5 Marker Min Peak Height: 200 Marker Baseline V to V?: Y Marker Baseline V to V pts: 3
Lower Marker Selection: First Peak > 200 RFU Upper Marker Selection: Last Peak > 200 RFU
Ladder Size (bp): 1, 75, 200, 300, 400, 500, 700, 1000, 1500, 2000, 3000, 4000, 5000, 7000, 10000, 20000
Quantification Using: Ladder Final Concentration (ng/uL): 1.0417 Dilution Factor: 12.0
Size Threshold (b.p.): 10000

 Copyright 2015 Advanced Analytical Technologies, Inc.

Data File: 2019 06 18 14H 35M.raw
Sample: 103613-001-101 (20x dil.)
Well Location: E1

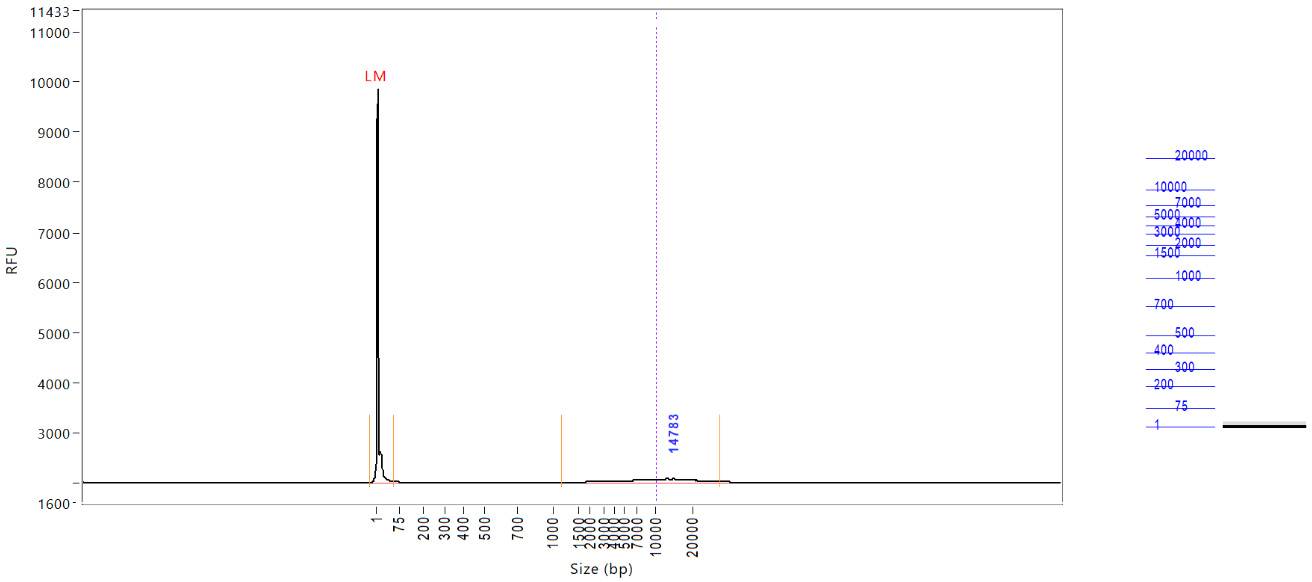

Peak	Size (bp)	Conc. (ng/uL)	From (bp)	To (bp)	Avg. Size (bp)	CV%	RFU	Corr. Peak Area
1	1 (LM)	0.0328	0	58	2	333.23	7864	55.421
2	14783	0.1062	1169	27248	11118	60.39	77	14.953

TIC:	0.1062	ng/uL
TIM:	0.0157	nmole/L
Total Conc.:	0.1635	ng/uL
GQN:	4.2	

```
Sample Peak Width (sec): 50   Sample Min Peak Height: 50    Sample Baseline V to V?: Y    Sample Baseline V to V pts: 3
Sample Filter: Binomial       # of Pts for Filter: 3        Sample Start Region (min): 0  Sample End Region (min): 50
Manual Baseline Start (min): 6     Manual Baseline End (min): 48
Marker Peak Width (sec): 5    Marker Min Peak Height: 200   Marker Baseline V to V?: Y    Marker Baseline V to V pts: 3
Lower Marker Selection: First Peak > 200 RFU                Upper Marker Selection: Last Peak > 200 RFU
Ladder Size (bp): 1, 75, 200, 300, 400, 500, 700, 1000, 1500, 2000, 3000, 4000, 5000, 7000, 10000, 20000
Quantification Using: Ladder          Final Concentration (ng/uL): 1.0417          Dilution Factor: 12.0
Size Threshold (b.p.): 10000
```

 Copyright 2015 Advanced Analytical Technologies, Inc.

Data File: 2019 06 18 14H 35M.raw
Sample: 103613-001-102 (20x dil.)
Well Location: F1

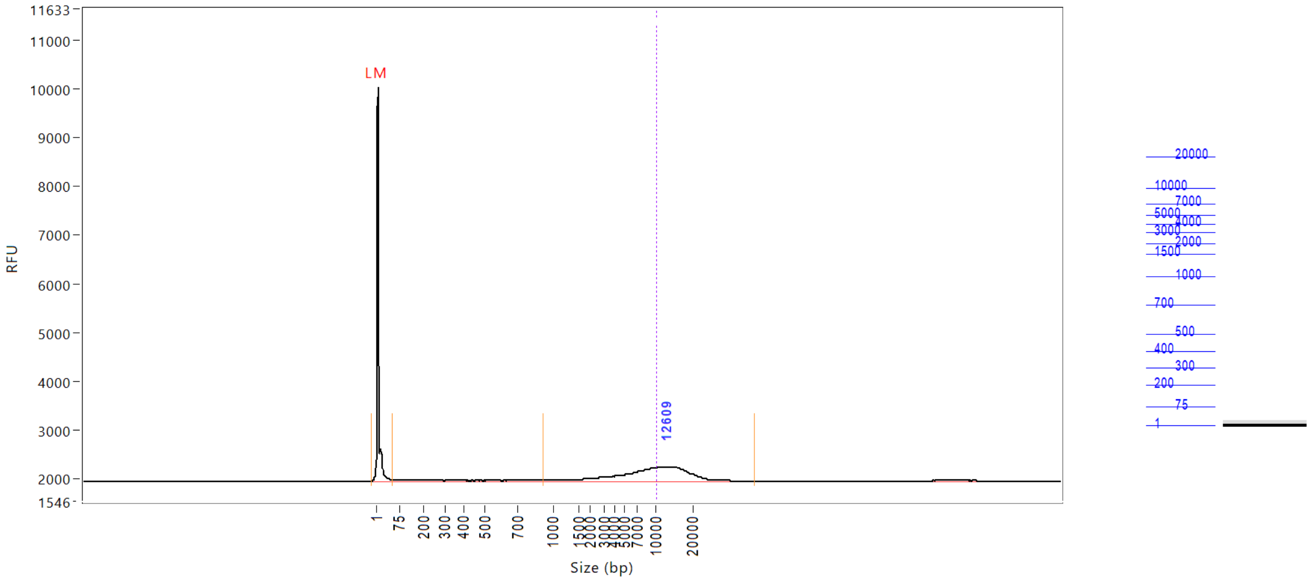

Peak	Size (bp)	Conc. (ng/uL)	From (bp)	To (bp)	Avg. Size (bp)	CV%	RFU	Corr. Peak Area
1	1 (LM)	0.0328	0	54	2	354.68	8065	56.716
2	12609	0.3388	916	36480	10497	59.79	303	48.815

TIC:	0.3388	ng/uL
TIM:	0.0531	nmole/L
Total Conc.:	0.4222	ng/uL
GQN:	4.2	

Sample Peak Width (sec): 50 Sample Min Peak Height: 50 Sample Baseline V to V?: Y Sample Baseline V to V pts: 3
Sample Filter: Binomial # of Pts for Filter: 3 Sample Start Region (min): 0 Sample End Region (min): 50
Manual Baseline Start (min): 6 Manual Baseline End (min): 48
Marker Peak Width (sec): 5 Marker Min Peak Height: 200 Marker Baseline V to V?: Y Marker Baseline V to V pts: 3
Lower Marker Selection: First Peak > 200 RFU Upper Marker Selection: Last Peak > 200 RFU
Ladder Size (bp): 1, 75, 200, 300, 400, 500, 700, 1000, 1500, 2000, 3000, 4000, 5000, 7000, 10000, 20000
Quantification Using: Ladder Final Concentration (ng/uL): 1.0417 Dilution Factor: 12.0
Size Threshold (b.p.): 10000

 Copyright 2015 Advanced Analytical Technologies, Inc.

Data File: 2019 06 18 14H 35M.raw
Sample: 103613-001-103 (20x dil.)
Well Location: G1

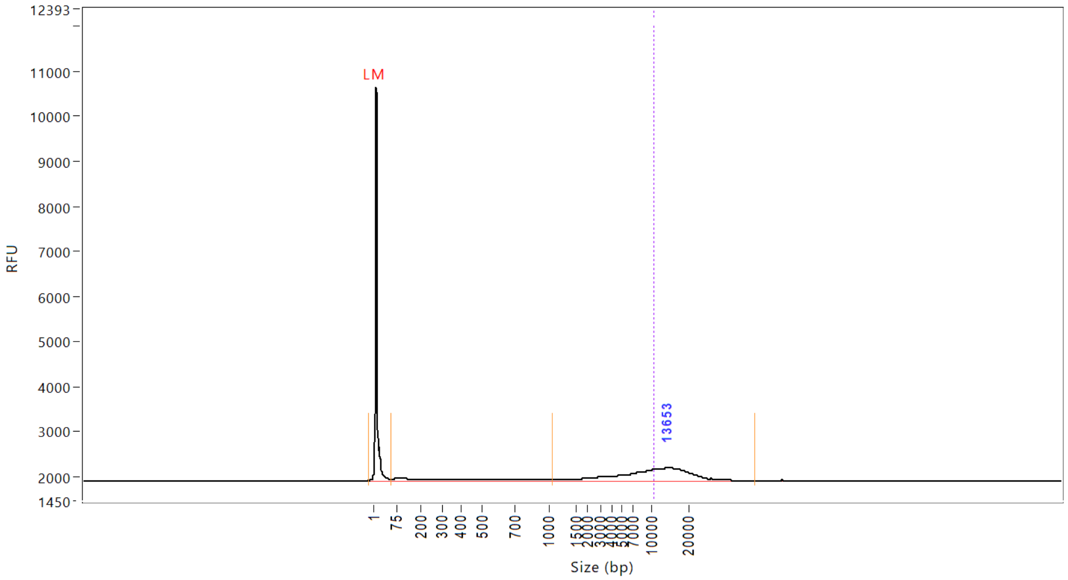

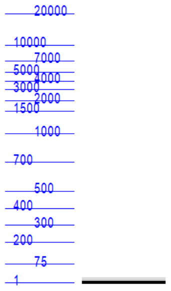

Peak	Size (bp)	Conc. (ng/uL)	From (bp)	To (bp)	Avg. Size (bp)	CV%	RFU	Corr. Peak Area
1	1 (LM)	0.0328	0	52	3	256.57	8743	62.491
2	13653	0.3150	1038	37861	11562	61.66	274	50.001

TIC:	0.3150	ng/uL
TIM:	0.0448	nmole/L
Total Conc.:	0.4098	ng/uL
GQN:	4.4	

Sample Peak Width (sec): 50   Sample Min Peak Height: 50   Sample Baseline V to V?: Y   Sample Baseline V to V pts: 3
Sample Filter: Binomial   # of Pts for Filter: 3   Sample Start Region (min): 0   Sample End Region (min): 50
Manual Baseline Start (min): 6   Manual Baseline End (min): 48
Marker Peak Width (sec): 5   Marker Min Peak Height: 200   Marker Baseline V to V?: Y   Marker Baseline V to V pts: 3
Lower Marker Selection: First Peak > 200 RFU   Upper Marker Selection: Last Peak > 200 RFU
Ladder Size (bp): 1, 75, 200, 300, 400, 500, 700, 1000, 1500, 2000, 3000, 4000, 5000, 7000, 10000, 20000
Quantification Using: Ladder   Final Concentration (ng/uL): 1.0417   Dilution Factor: 12.0
Size Threshold (b.p.): 10000

Copyright 2015 Advanced Analytical Technologies, Inc.

Data File: 2019 06 18 14H 35M.raw
Sample: 103613-001-104 (20x dil.)
Well Location: H1

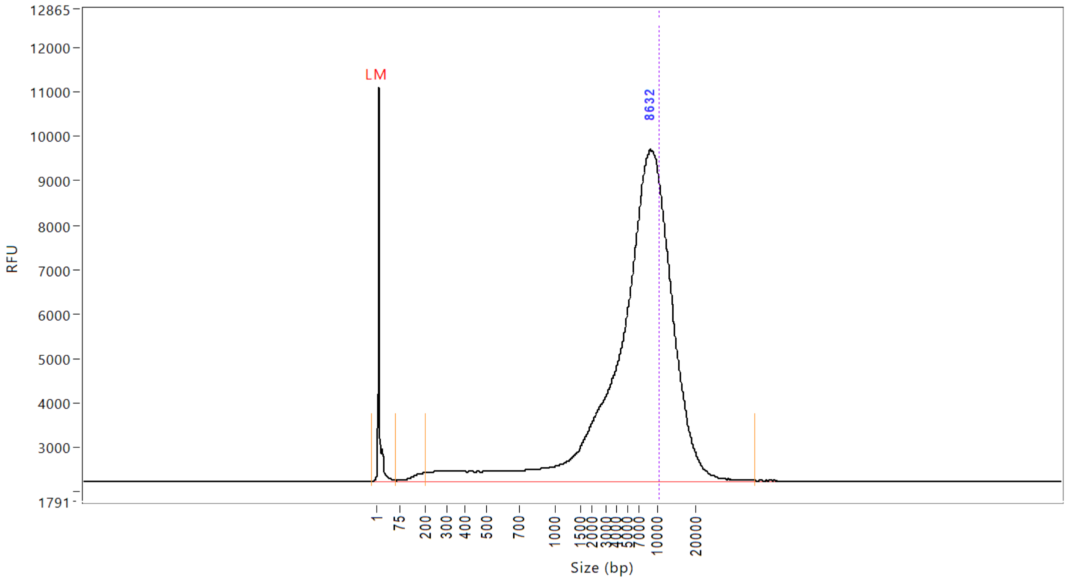

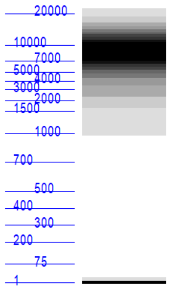

Peak	Size (bp)	Conc. (ng/uL)	From (bp)	To (bp)	Avg. Size (bp)	CV%	RFU	Corr. Peak Area
1	1 (LM)	0.0328	0	60	3	303.10	8855	61.602
2	8632	5.7375	200	36049	7487	64.03	7468	897.894

TIC:	5.7375	ng/uL
TIM:	1.2616	nmole/L
Total Conc.:	5.8172	ng/uL
GQN:	2.6	

Sample Peak Width (sec): 50 Sample Min Peak Height: 50 Sample Baseline V to V?: Y Sample Baseline V to V pts: 3
Sample Filter: Binomial # of Pts for Filter: 3 Sample Start Region (min): 0 Sample End Region (min): 50
Manual Baseline Start (min): 6 Manual Baseline End (min): 48
Marker Peak Width (sec): 5 Marker Min Peak Height: 200 Marker Baseline V to V?: Y Marker Baseline V to V pts: 3
Lower Marker Selection: First Peak > 200 RFU Upper Marker Selection: Last Peak > 200 RFU
Ladder Size (bp): 1, 75, 200, 300, 400, 500, 700, 1000, 1500, 2000, 3000, 4000, 5000, 7000, 10000, 20000
Quantification Using: Ladder Final Concentration (ng/uL): 1.0417 Dilution Factor: 12.0
Size Threshold (b.p.): 10000

 Copyright 2015 Advanced Analytical Technologies, Inc.

Data File: 2019 06 18 14H 35M.raw
Sample: 103613-001-105 (20x dil.)
Well Location: A2

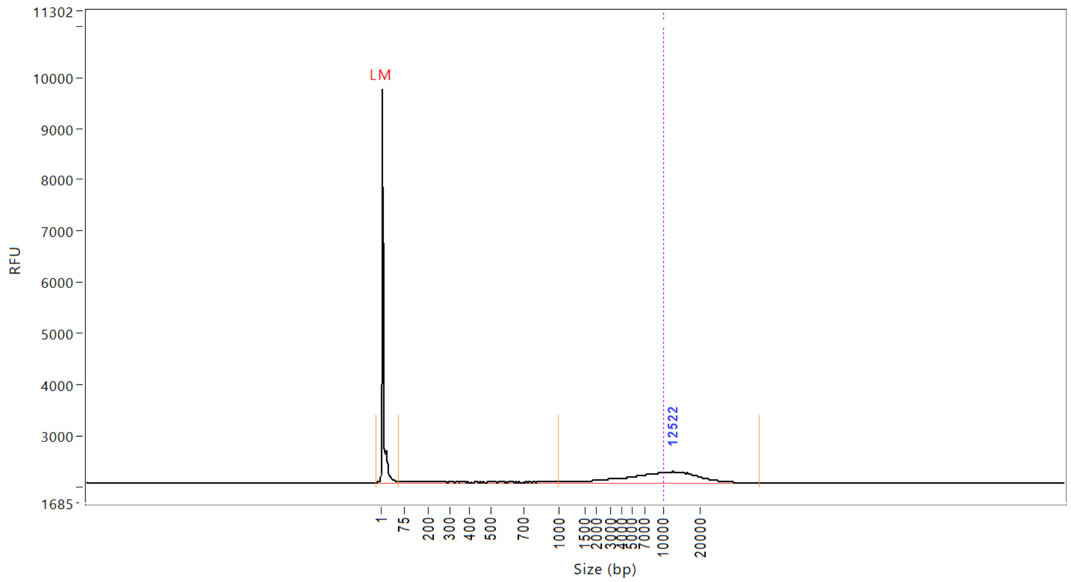

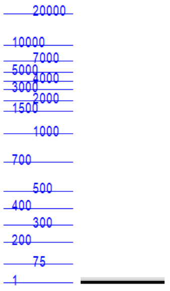

Peak	Size (bp)	Conc. (ng/uL)	From (bp)	To (bp)	Avg. Size (bp)	CV%	RFU	Corr. Peak Area
1	1 (LM)	0.0328	0	57	3	263.97	7691	54.682
2	12522	0.2802	994	35790	10398	64.21	213	38.920

TIC:	0.2802	ng/uL
TIM:	0.0444	nmole/L
Total Conc.:	0.3532	ng/uL
GQN:	3.8	

Sample Peak Width (sec): 50 Sample Min Peak Height: 50 Sample Baseline V to V?: Y Sample Baseline V to V pts: 3
Sample Filter: Binomial # of Pts for Filter: 3 Sample Start Region (min): 0 Sample End Region (min): 50
Manual Baseline Start (min): 6 Manual Baseline End (min): 48
Marker Peak Width (sec): 5 Marker Min Peak Height: 200 Marker Baseline V to V?: Y Marker Baseline V to V pts: 3
Lower Marker Selection: First Peak > 200 RFU Upper Marker Selection: Last Peak > 200 RFU
Ladder Size (bp): 1, 75, 200, 300, 400, 500, 700, 1000, 1500, 2000, 3000, 4000, 5000, 7000, 10000, 20000
Quantification Using: Ladder Final Concentration (ng/uL): 1.0417 Dilution Factor: 12.0
Size Threshold (b.p.): 10000

 Copyright 2015 Advanced Analytical Technologies, Inc.

Data File: 2019 06 18 14H 35M.raw
Sample: 103613-001-106 (20x dil.)
Well Location: B2

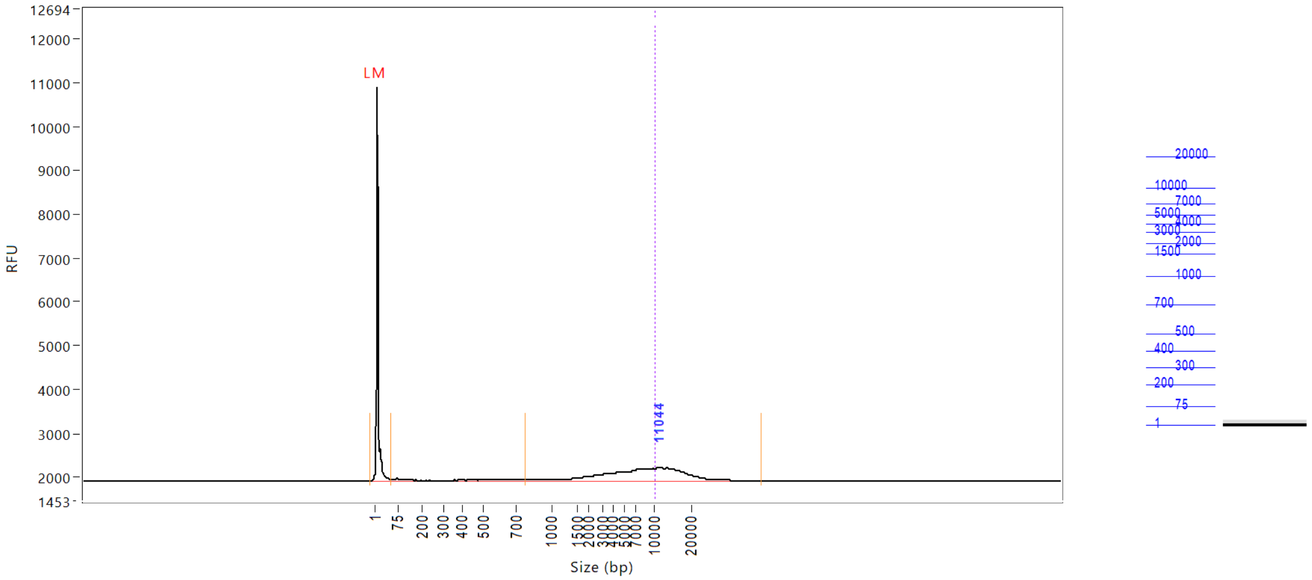

Peak	Size (bp)	Conc. (ng/uL)	From (bp)	To (bp)	Avg. Size (bp)	CV%	RFU	Corr. Peak Area
1	1 (LM)	0.0328	0	48	2	303.30	8991	62.461
2	11044	0.3659	762	38896	9065	73.32	287	58.055

TIC:	0.3659	ng/uL
TIM:	0.0664	nmole/L
Total Conc.:	0.4397	ng/uL
GQN:	3.3	

Sample Peak Width (sec): 50 Sample Min Peak Height: 50 Sample Baseline V to V?: Y Sample Baseline V to V pts: 3
Sample Filter: Binomial # of Pts for Filter: 3 Sample Start Region (min): 0 Sample End Region (min): 50
Manual Baseline Start (min): 6 Manual Baseline End (min): 48
Marker Peak Width (sec): 5 Marker Min Peak Height: 200 Marker Baseline V to V?: Y Marker Baseline V to V pts: 3
Lower Marker Selection: First Peak > 200 RFU Upper Marker Selection: Last Peak > 200 RFU
Ladder Size (bp): 1, 75, 200, 300, 400, 500, 700, 1000, 1500, 2000, 3000, 4000, 5000, 7000, 10000, 20000
Quantification Using: Ladder Final Concentration (ng/uL): 1.0417 Dilution Factor: 12.0
Size Threshold (b.p.): 10000

 Copyright 2015 Advanced Analytical Technologies, Inc.

Data File: 2019 06 18 14H 35M.raw
Sample: 103613-001-107 (20x dil.)
Well Location: C2

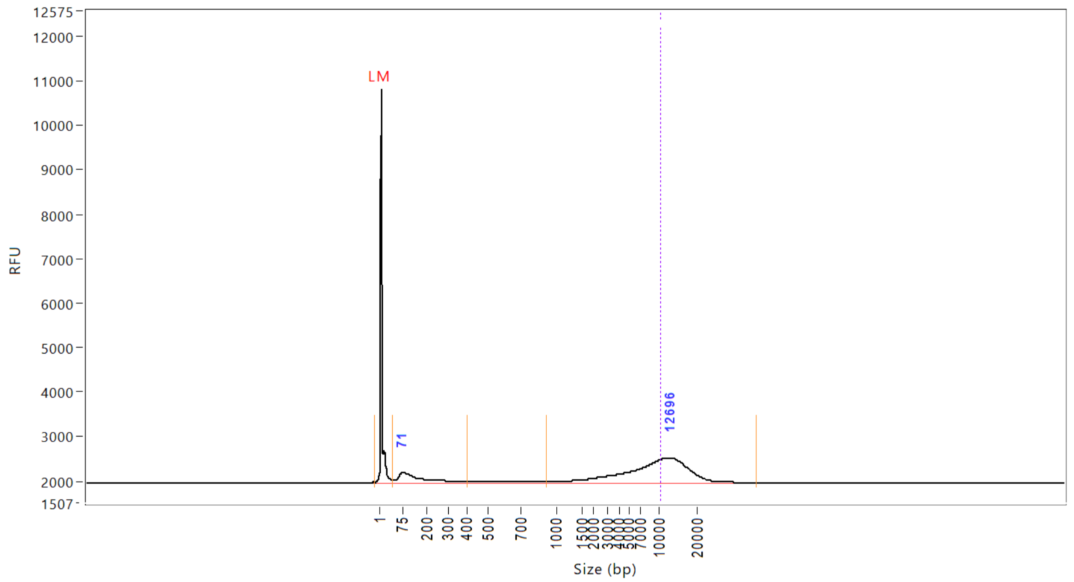

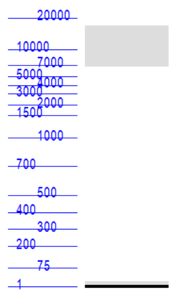

Peak	Size (bp)	Conc. (ng/uL)	From (bp)	To (bp)	Avg. Size (bp)	CV%	RFU	Corr. Peak Area
1	1 (LM)	0.0328	0	44	2	376.11	8847	63.024
2	71	0.1195	44	393	153	60.61	221	19.126
3	12696	0.5004	919	36049	9916	58.66	557	80.118

TIC:	0.6199	ng/uL
TIM:	1.3663	nmole/L
Total Conc.:	0.6727	ng/uL
GQN:	3.4	

Sample Peak Width (sec): 50 Sample Min Peak Height: 50 Sample Baseline V to V?: Y Sample Baseline V to V pts: 3
Sample Filter: Binomial # of Pts for Filter: 3 Sample Start Region (min): 0 Sample End Region (min): 50
Manual Baseline Start (min): 6 Manual Baseline End (min): 48
Marker Peak Width (sec): 5 Marker Min Peak Height: 200 Marker Baseline V to V?: Y Marker Baseline V to V pts: 3
Lower Marker Selection: First Peak > 200 RFU Upper Marker Selection: Last Peak > 200 RFU
Ladder Size (bp): 1, 75, 200, 300, 400, 500, 700, 1000, 1500, 2000, 3000, 4000, 5000, 7000, 10000, 20000
Quantification Using: Ladder Final Concentration (ng/uL): 1.0417 Dilution Factor: 12.0
Size Threshold (b.p.): 10000

 Copyright 2015 Advanced Analytical Technologies, Inc.

Data File: 2019 06 18 14H 35M.raw
Sample: 103613-001-108 (20x dil.)
Well Location: D2

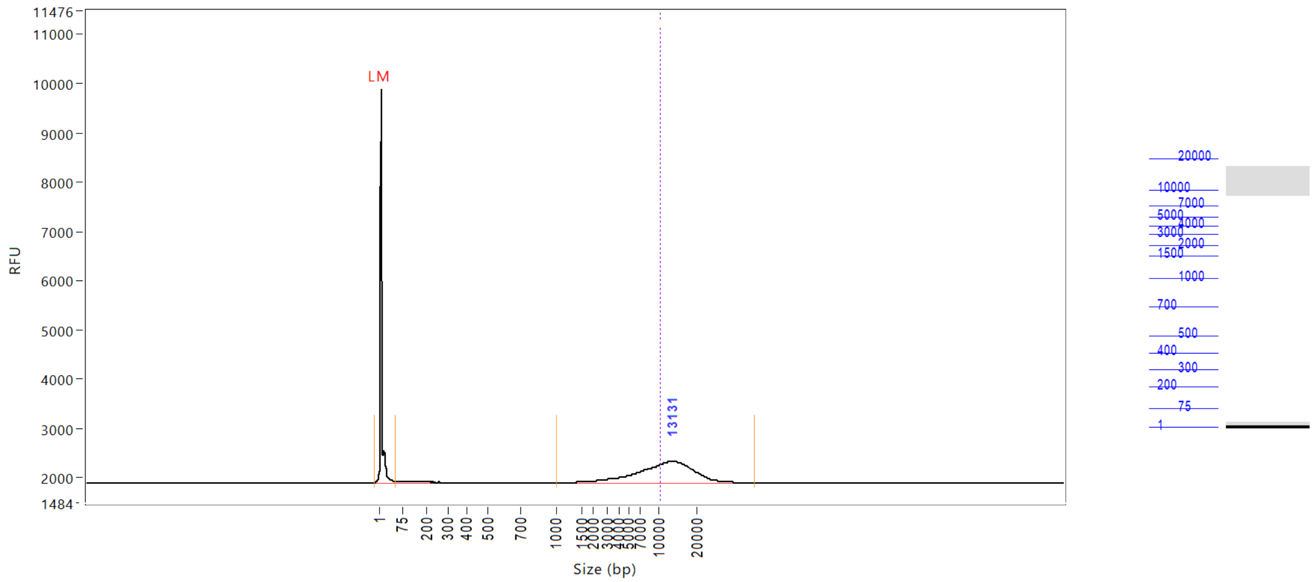

Peak	Size (bp)	Conc. (ng/uL)	From (bp)	To (bp)	Avg. Size (bp)	CV%	RFU	Corr. Peak Area
1	1 (LM)	0.0328	0	52	2	376.46	7992	56.961
2	13131	0.4454	1007	35531	11689	52.48	440	64.446

TIC:	0.4454	ng/uL
TIM:	0.0627	nmole/L
Total Conc.:	0.5156	ng/uL
GQN:	5.1	

Sample Peak Width (sec): 50   Sample Min Peak Height: 50   Sample Baseline V to V?: Y   Sample Baseline V to V pts: 3
Sample Filter: Binomial   # of Pts for Filter: 3   Sample Start Region (min): 0   Sample End Region (min): 50
Manual Baseline Start (min): 6   Manual Baseline End (min): 48
Marker Peak Width (sec): 5   Marker Min Peak Height: 200   Marker Baseline V to V?: Y   Marker Baseline V to V pts: 3
Lower Marker Selection: First Peak > 200 RFU   Upper Marker Selection: Last Peak > 200 RFU
Ladder Size (bp): 1, 75, 200, 300, 400, 500, 700, 1000, 1500, 2000, 3000, 4000, 5000, 7000, 10000, 20000
Quantification Using: Ladder   Final Concentration (ng/uL): 1.0417   Dilution Factor: 12.0
Size Threshold (b.p.): 10000

 Copyright 2015 Advanced Analytical Technologies, Inc.

Data File: 2019 06 18 14H 35M.raw
Sample: 103613-001-109 (20x dil.)
Well Location: E2

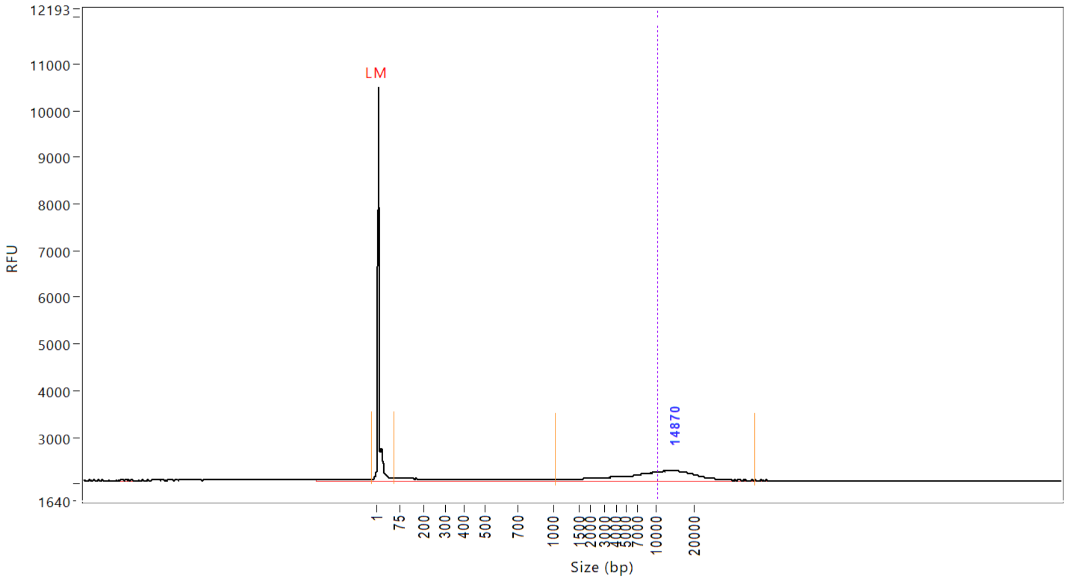

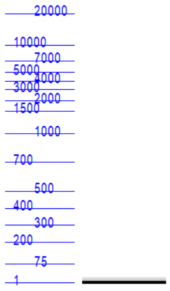

Peak	Size (bp)	Conc. (ng/uL)	From (bp)	To (bp)	Avg. Size (bp)	CV%	RFU	Corr. Peak Area
1	1 (LM)	0.0328	0	54	3	282.26	8423	59.451
2	14870	0.2481	1026	36135	11311	63.15	205	37.471

TIC:	0.2481	ng/uL
TIM:	0.0361	nmole/L
Total Conc.:	0.3472	ng/uL
GQN:	4.0	

Sample Peak Width (sec): 50 Sample Min Peak Height: 50 Sample Baseline V to V?: Y Sample Baseline V to V pts: 3
Sample Filter: Binomial # of Pts for Filter: 3 Sample Start Region (min): 0 Sample End Region (min): 50
Manual Baseline Start (min): 6 Manual Baseline End (min): 48
Marker Peak Width (sec): 5 Marker Min Peak Height: 200 Marker Baseline V to V?: Y Marker Baseline V to V pts: 3
Lower Marker Selection: First Peak > 200 RFU Upper Marker Selection: Last Peak > 200 RFU
Ladder Size (bp): 1, 75, 200, 300, 400, 500, 700, 1000, 1500, 2000, 3000, 4000, 5000, 7000, 10000, 20000
Quantification Using: Ladder Final Concentration (ng/uL): 1.0417 Dilution Factor: 12.0
Size Threshold (b.p.): 10000

 Copyright 2015 Advanced Analytical Technologies, Inc.

Data File: 2019 06 18 14H 35M.raw
Sample: 103613-001-110 (20x dil.)
Well Location: F2

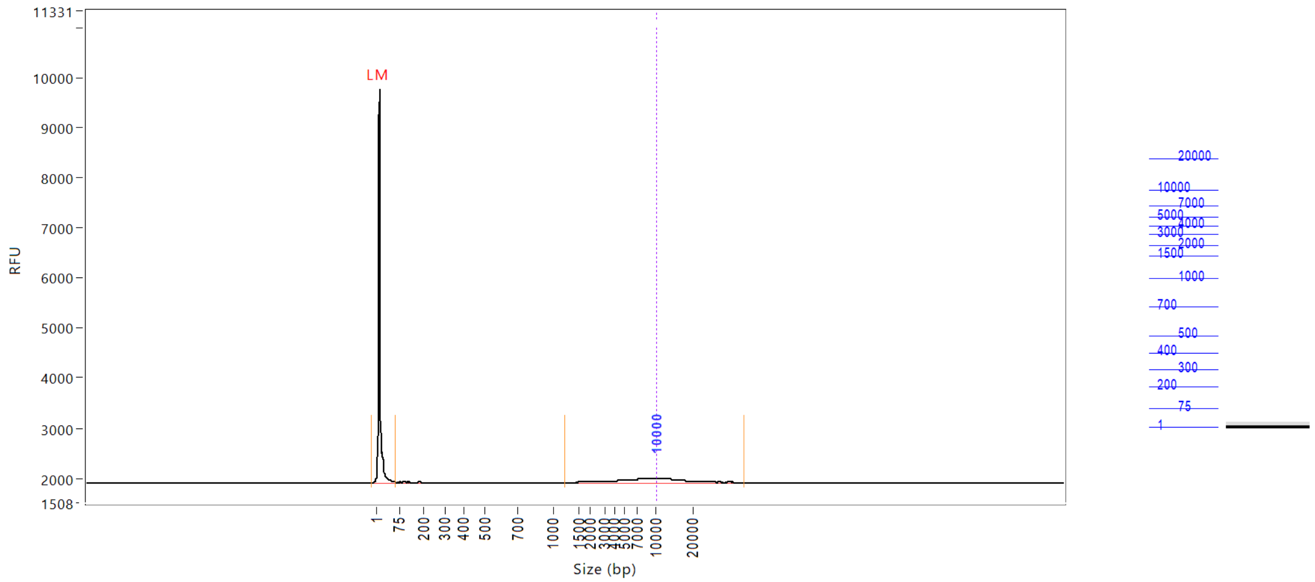

Peak	Size (bp)	Conc. (ng/uL)	From (bp)	To (bp)	Avg. Size (bp)	CV%	RFU	Corr. Peak Area
1	1 (LM)	0.0328	0	59	2	335.84	7855	55.308
2	10000	0.1114	1226	34151	10249	68.27	88	15.654

TIC:	0.1114	ng/uL
TIM:	0.0179	nmole/L
Total Conc.:	0.1563	ng/uL
GQN:	3.3	

```
Sample Peak Width (sec): 50   Sample Min Peak Height: 50    Sample Baseline V to V?: Y    Sample Baseline V to V pts: 3
Sample Filter: Binomial       # of Pts for Filter: 3        Sample Start Region (min): 0  Sample End Region (min): 50
Manual Baseline Start (min): 6     Manual Baseline End (min): 48
Marker Peak Width (sec): 5    Marker Min Peak Height: 200   Marker Baseline V to V?: Y    Marker Baseline V to V pts: 3
Lower Marker Selection: First Peak > 200 RFU                Upper Marker Selection: Last Peak > 200 RFU
Ladder Size (bp): 1, 75, 200, 300, 400, 500, 700, 1000, 1500, 2000, 3000, 4000, 5000, 7000, 10000, 20000
Quantification Using: Ladder         Final Concentration (ng/uL): 1.0417         Dilution Factor: 12.0
Size Threshold (b.p.): 10000
```


Copyright 2015 Advanced Analytical Technologies, Inc.

Data File: 2019 06 18 14H 35M.raw
Sample: 103613-001-111 (20x dil.)
Well Location: G2

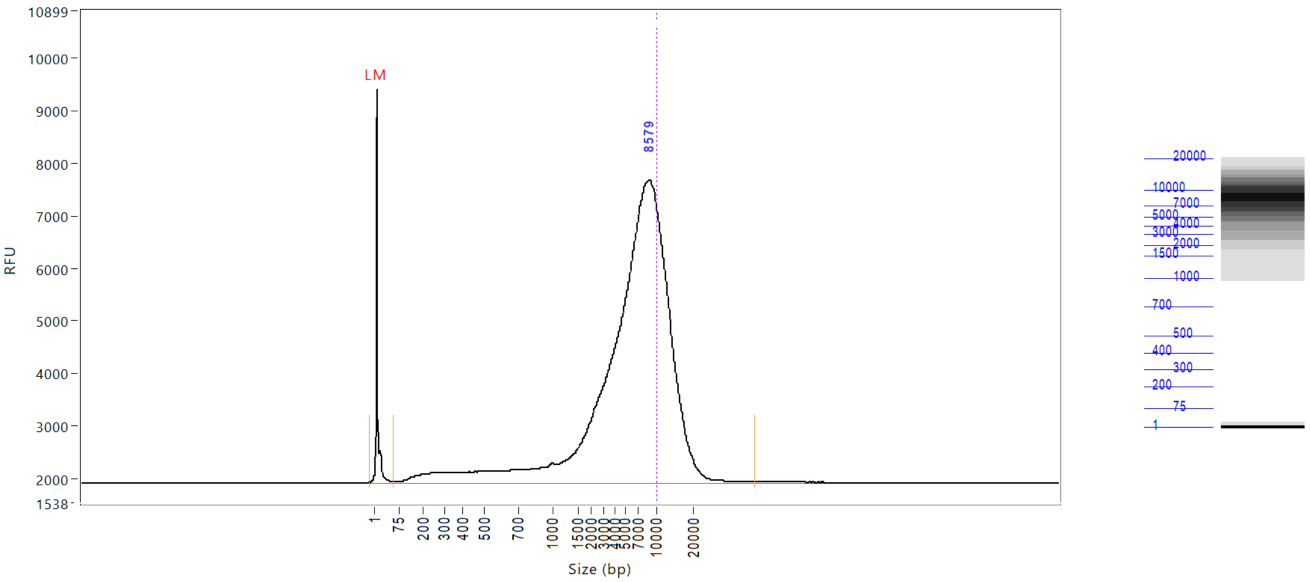

Peak	Size (bp)	Conc. (ng/uL)	From (bp)	To (bp)	Avg. Size (bp)	CV%	RFU	Corr. Peak Area
1	1 (LM)	0.0328	0	55	2	355.71	7482	52.584
2	8579	5.6168	55	36567	6925	67.95	5752	750.319

TIC:	5.6168	ng/uL
TIM:	1.3353	nmole/L
Total Conc.:	5.6588	ng/uL
GQN:	2.3	

Sample Peak Width (sec): 50 Sample Min Peak Height: 50 Sample Baseline V to V?: Y Sample Baseline V to V pts: 3
Sample Filter: Binomial # of Pts for Filter: 3 Sample Start Region (min): 0 Sample End Region (min): 50
Manual Baseline Start (min): 6 Manual Baseline End (min): 48
Marker Peak Width (sec): 5 Marker Min Peak Height: 200 Marker Baseline V to V?: Y Marker Baseline V to V pts: 3
Lower Marker Selection: First Peak > 200 RFU Upper Marker Selection: Last Peak > 200 RFU
Ladder Size (bp): 1, 75, 200, 300, 400, 500, 700, 1000, 1500, 2000, 3000, 4000, 5000, 7000, 10000, 20000
Quantification Using: Ladder Final Concentration (ng/uL): 1.0417 Dilution Factor: 12.0
Size Threshold (b.p.): 10000

 Copyright 2015 Advanced Analytical Technologies, Inc.

Data File: 2019 06 18 14H 35M.raw
Sample: 103613-001-112 (20x dil.)
Well Location: H2

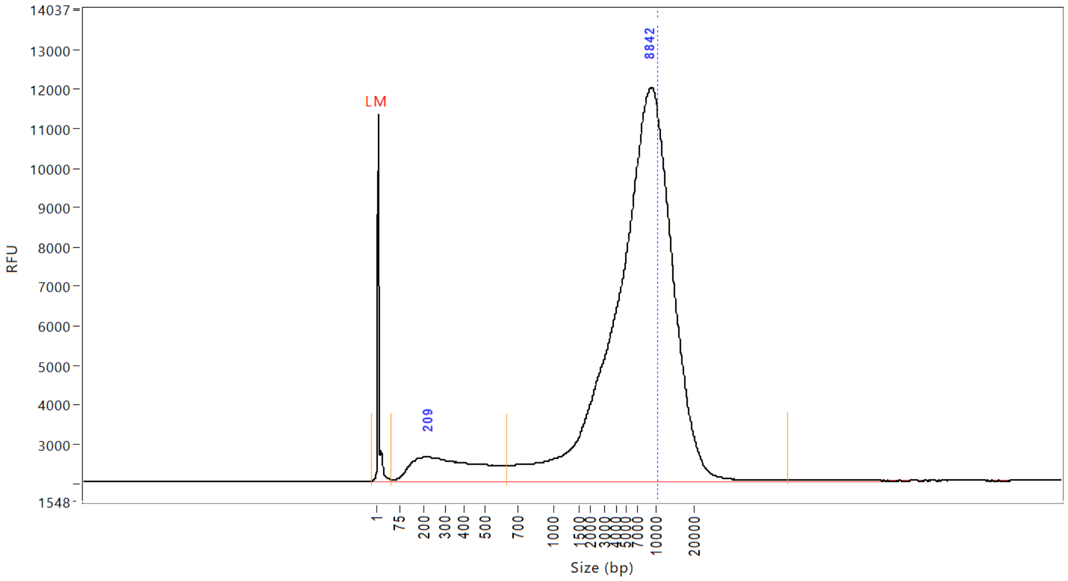

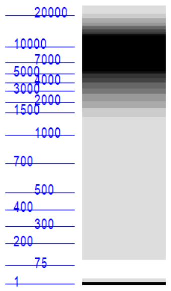

Peak	Size (bp)	Conc. (ng/uL)	From (bp)	To (bp)	Avg. Size (bp)	CV%	RFU	Corr. Peak Area
1	1 (LM)	0.0328	0	46	2	360.05	9301	65.533
2	209	0.8236	46	626	320	46.27	605	137.108
3	8842	7.5495	626	45109	7855	61.97	9975	1256.851

TIC:	8.3731	ng/uL
TIM:	5.8160	nmole/L
Total Conc.:	8.3880	ng/uL
GQN:	2.5	

Sample Peak Width (sec): 50 Sample Min Peak Height: 50 Sample Baseline V to V?: Y Sample Baseline V to V pts: 3
Sample Filter: Binomial # of Pts for Filter: 3 Sample Start Region (min): 0 Sample End Region (min): 50
Manual Baseline Start (min): 6 Manual Baseline End (min): 48
Marker Peak Width (sec): 5 Marker Min Peak Height: 200 Marker Baseline V to V?: Y Marker Baseline V to V pts: 3
Lower Marker Selection: First Peak > 200 RFU Upper Marker Selection: Last Peak > 200 RFU
Ladder Size (bp): 1, 75, 200, 300, 400, 500, 700, 1000, 1500, 2000, 3000, 4000, 5000, 7000, 10000, 20000
Quantification Using: Ladder Final Concentration (ng/uL): 1.0417 Dilution Factor: 12.0
Size Threshold (b.p.): 10000

 Copyright 2015 Advanced Analytical Technologies, Inc.

Data File: 2019 06 18 14H 35M.raw
Sample: 103613-001-113 (20x dil.)
Well Location: A3

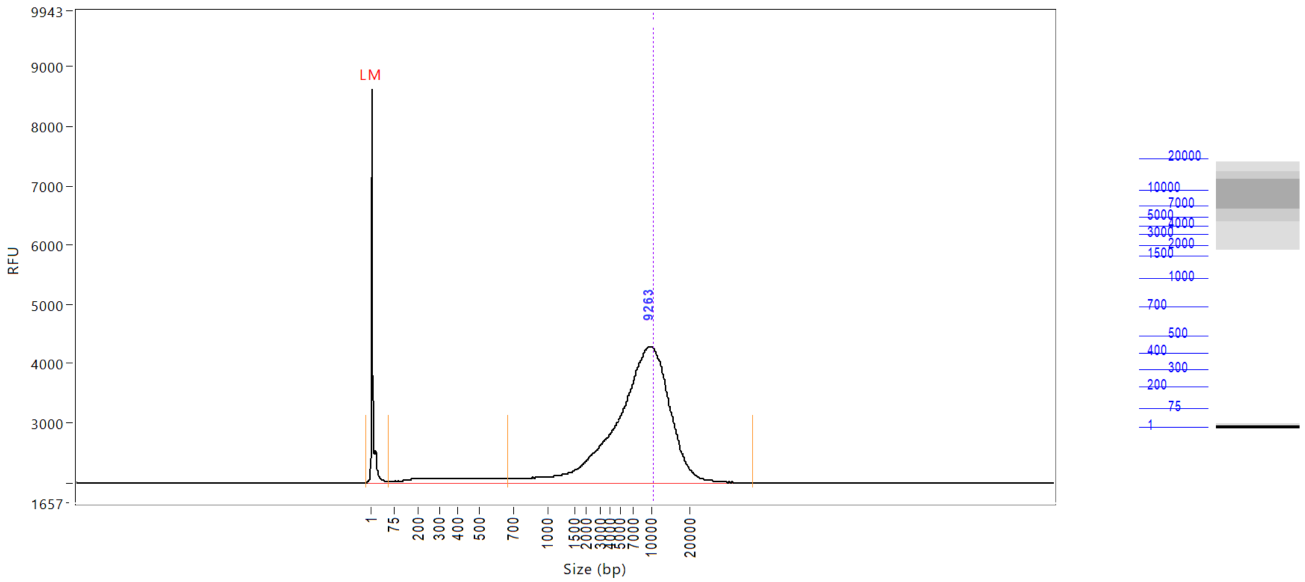

Peak	Size (bp)	Conc. (ng/uL)	From (bp)	To (bp)	Avg. Size (bp)	CV%	RFU	Corr. Peak Area
1	1 (LM)	0.0328	0	56	2	381.39	6626	47.710
2	9263	2.2790	663	36912	8358	57.36	2290	276.223

TIC:	2.2790	ng/uL
TIM:	0.4489	nmole/L
Total Conc.:	2.4589	ng/uL
GQN:	3.0	

Sample Peak Width (sec): 50 Sample Min Peak Height: 50 Sample Baseline V to V?: Y Sample Baseline V to V pts: 3
Sample Filter: Binomial # of Pts for Filter: 3 Sample Start Region (min): 0 Sample End Region (min): 50
Manual Baseline Start (min): 6 Manual Baseline End (min): 48
Marker Peak Width (sec): 5 Marker Min Peak Height: 200 Marker Baseline V to V?: Y Marker Baseline V to V pts: 3
Lower Marker Selection: First Peak > 200 RFU Upper Marker Selection: Last Peak > 200 RFU
Ladder Size (bp): 1, 75, 200, 300, 400, 500, 700, 1000, 1500, 2000, 3000, 4000, 5000, 7000, 10000, 20000
Quantification Using: Ladder Final Concentration (ng/uL): 1.0417 Dilution Factor: 12.0
Size Threshold (b.p.): 10000

 Copyright 2015 Advanced Analytical Technologies, Inc.

Data File: 2019 06 18 14H 35M.raw
Sample: 103613-001-114 (20x dil.)
Well Location: B3

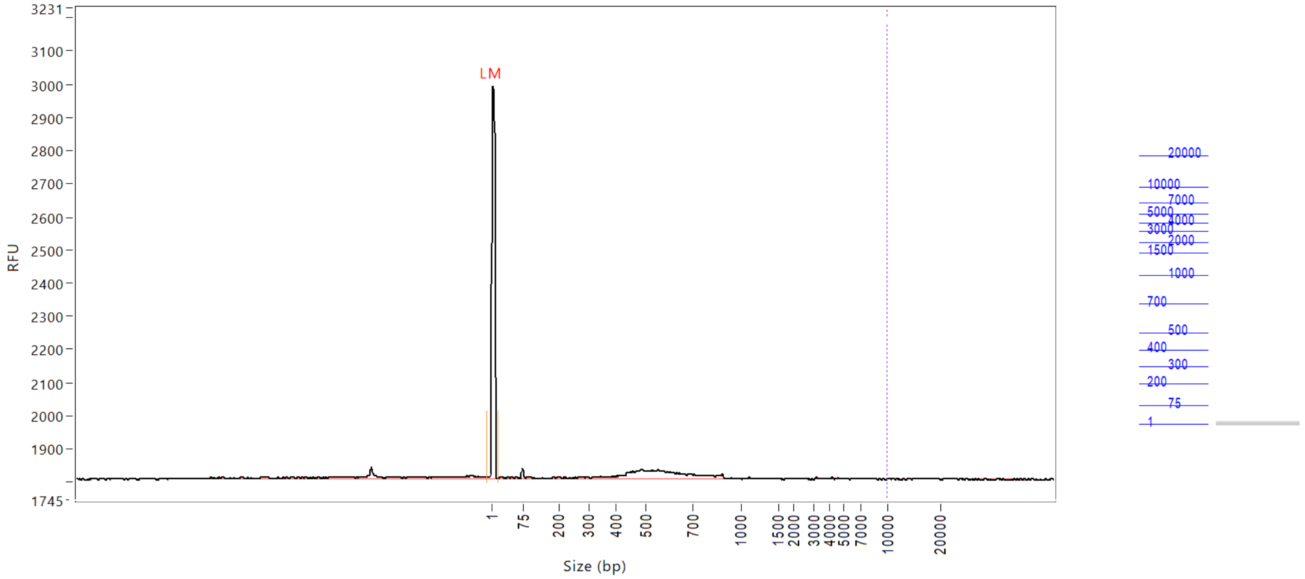

Peak	Size (bp)	Conc. (ng/uL)	From (bp)	To (bp)	Avg. Size (bp)	CV%	RFU	Corr. Peak Area
1	1 (LM)	0.0328	0	13	1	281.11	1185	10.579

TIC:	0.0000	ng/uL
TIM:	0.0000	nmole/L
Total Conc.:	0.1725	ng/uL
GQN:	0.2	

Sample Peak Width (sec): 50 Sample Min Peak Height: 50 Sample Baseline V to V?: Y Sample Baseline V to V pts: 3
Sample Filter: Binomial # of Pts for Filter: 3 Sample Start Region (min): 0 Sample End Region (min): 50
Manual Baseline Start (min): 6 Manual Baseline End (min): 48
Marker Peak Width (sec): 5 Marker Min Peak Height: 200 Marker Baseline V to V?: Y Marker Baseline V to V pts: 3
Lower Marker Selection: First Peak > 200 RFU Upper Marker Selection: Last Peak > 200 RFU
Ladder Size (bp): 1, 75, 200, 300, 400, 500, 700, 1000, 1500, 2000, 3000, 4000, 5000, 7000, 10000, 20000
Quantification Using: Ladder Final Concentration (ng/uL): 1.0417 Dilution Factor: 12.0
Size Threshold (b.p.): 10000

 Copyright 2015 Advanced Analytical Technologies, Inc.

Data File: 2019 06 18 14H 35M.raw
Sample: 103613-001-115 (20x dil.)
Well Location: C3

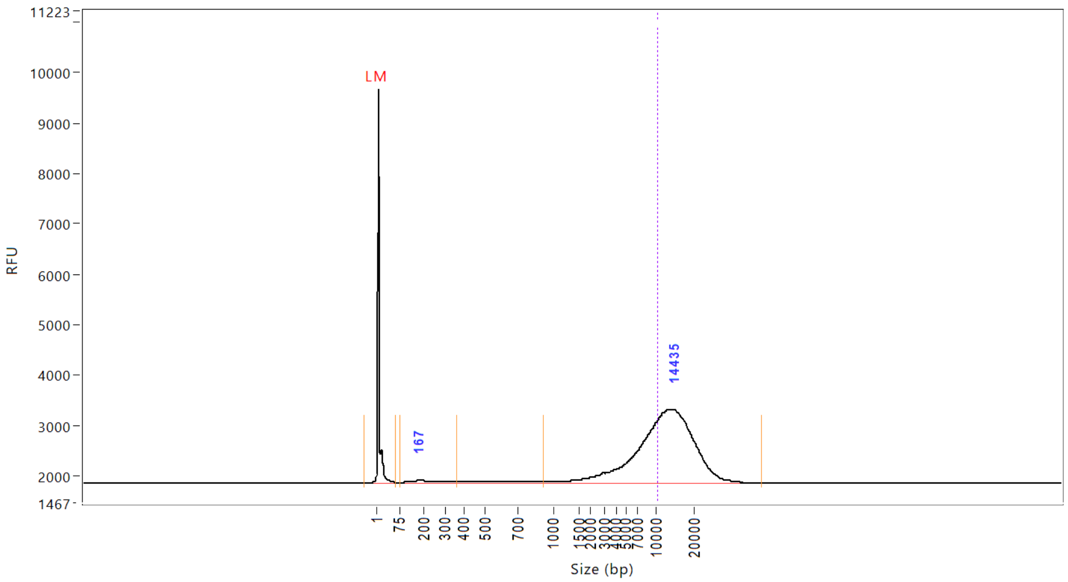

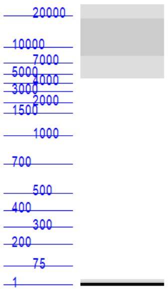

Peak	Size (bp)	Conc. (ng/uL)	From (bp)	To (bp)	Avg. Size (bp)	CV%	RFU	Corr. Peak Area
1	1 (LM)	0.0328	0	62	3	271.67	7802	56.136
2	167	0.0434	74	357	215	34.48	71	6.193
3	14435	1.4953	911	38292	12253	48.84	1455	213.247

TIC:	1.5388	ng/uL
TIM:	0.5330	nmole/L
Total Conc.:	1.5931	ng/uL
GQN:	5.7	

Sample Peak Width (sec): 50 Sample Min Peak Height: 50 Sample Baseline V to V?: Y Sample Baseline V to V pts: 3
Sample Filter: Binomial # of Pts for Filter: 3 Sample Start Region (min): 0 Sample End Region (min): 50
Manual Baseline Start (min): 6 Manual Baseline End (min): 48
Marker Peak Width (sec): 5 Marker Min Peak Height: 200 Marker Baseline V to V?: Y Marker Baseline V to V pts: 3
Lower Marker Selection: First Peak > 200 RFU Upper Marker Selection: Last Peak > 200 RFU
Ladder Size (bp): 1, 75, 200, 300, 400, 500, 700, 1000, 1500, 2000, 3000, 4000, 5000, 7000, 10000, 20000
Quantification Using: Ladder Final Concentration (ng/uL): 1.0417 Dilution Factor: 12.0
Size Threshold (b.p.): 10000

 Copyright 2015 Advanced Analytical Technologies, Inc.

Data File: 2019 06 18 14H 35M.raw
Sample: 103613-001-116 (20x dil.)
Well Location: D3

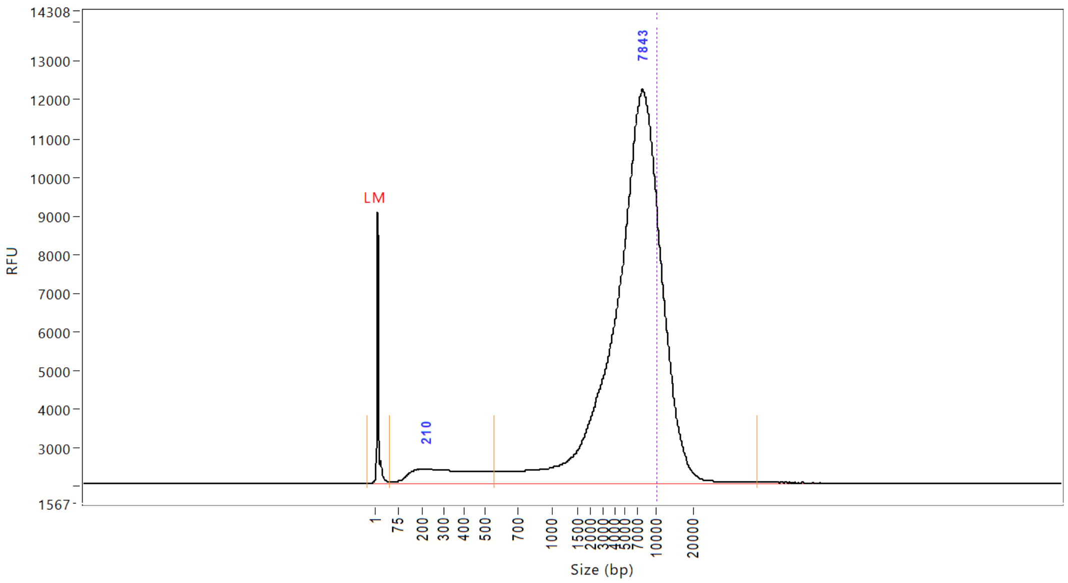

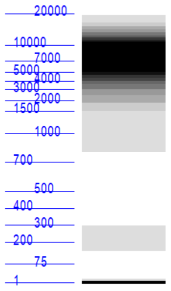

Peak	Size (bp)	Conc. (ng/uL)	From (bp)	To (bp)	Avg. Size (bp)	CV%	RFU	Corr. Peak Area
1	1 (LM)	0.0328	0	45	2	366.55	7034	49.417
2	210	0.6276	45	550	300	43.45	364	78.792
3	7843	8.2263	550	37602	6952	56.82	10184	1032.726

TIC:	8.8540	ng/uL
TIM:	5.3894	nmole/L
Total Conc.:	8.8871	ng/uL
GQN:	1.7	

Sample Peak Width (sec): 50 Sample Min Peak Height: 50 Sample Baseline V to V?: Y Sample Baseline V to V pts: 3
Sample Filter: Binomial # of Pts for Filter: 3 Sample Start Region (min): 0 Sample End Region (min): 50
Manual Baseline Start (min): 6 Manual Baseline End (min): 48
Marker Peak Width (sec): 5 Marker Min Peak Height: 200 Marker Baseline V to V?: Y Marker Baseline V to V pts: 3
Lower Marker Selection: First Peak > 200 RFU Upper Marker Selection: Last Peak > 200 RFU
Ladder Size (bp): 1, 75, 200, 300, 400, 500, 700, 1000, 1500, 2000, 3000, 4000, 5000, 7000, 10000, 20000
Quantification Using: Ladder Final Concentration (ng/uL): 1.0417 Dilution Factor: 12.0
Size Threshold (b.p.): 10000

 Copyright 2015 Advanced Analytical Technologies, Inc.

Data File: 2019 06 18 14H 35M.raw
Sample: 103613-001-117 (20x dil.)
Well Location: E3

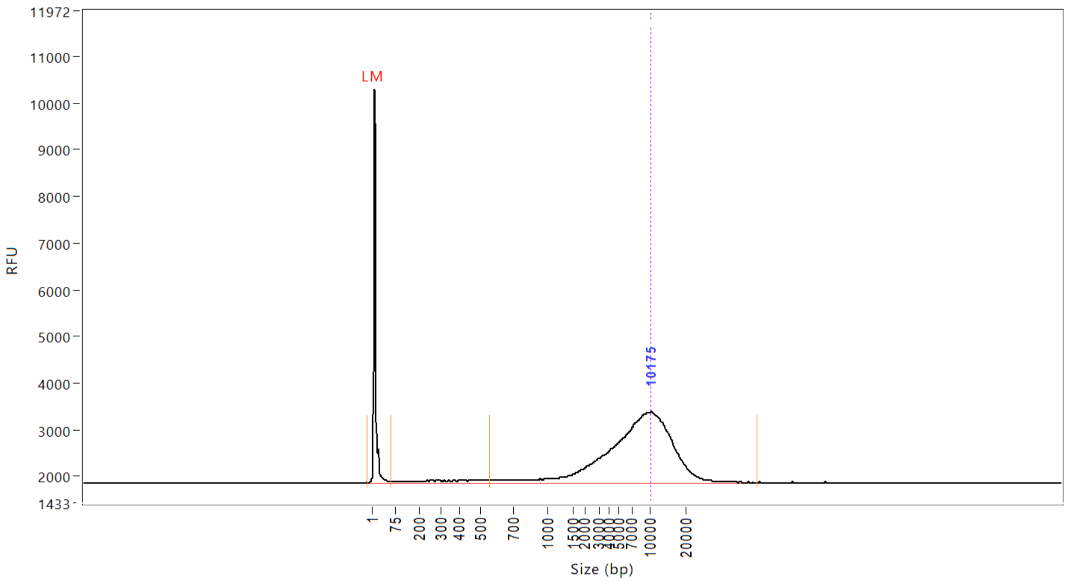

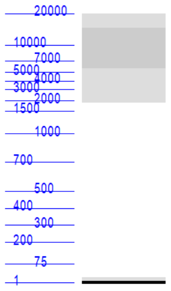

Peak	Size (bp)	Conc. (ng/uL)	From (bp)	To (bp)	Avg. Size (bp)	CV%	RFU	Corr. Peak Area
1	1 (LM)	0.0328	0	56	2	329.74	8427	59.756
2	10175	1.4780	556	39414	8666	65.30	1518	224.363

TIC:	1.4780	ng/uL
TIM:	0.2808	nmole/L
Total Conc.:	1.5914	ng/uL
GQN:	3.4	

Sample Peak Width (sec): 50 Sample Min Peak Height: 50 Sample Baseline V to V?: Y Sample Baseline V to V pts: 3
Sample Filter: Binomial # of Pts for Filter: 3 Sample Start Region (min): 0 Sample End Region (min): 50
Manual Baseline Start (min): 6 Manual Baseline End (min): 48
Marker Peak Width (sec): 5 Marker Min Peak Height: 200 Marker Baseline V to V?: Y Marker Baseline V to V pts: 3
Lower Marker Selection: First Peak > 200 RFU Upper Marker Selection: Last Peak > 200 RFU
Ladder Size (bp): 1, 75, 200, 300, 400, 500, 700, 1000, 1500, 2000, 3000, 4000, 5000, 7000, 10000, 20000
Quantification Using: Ladder Final Concentration (ng/uL): 1.0417 Dilution Factor: 12.0
Size Threshold (b.p.): 10000

 Copyright 2015 Advanced Analytical Technologies, Inc.

Data File: 2019 06 18 14H 35M.raw
Sample: 103613-001-118 (20x dil.)
Well Location: F3

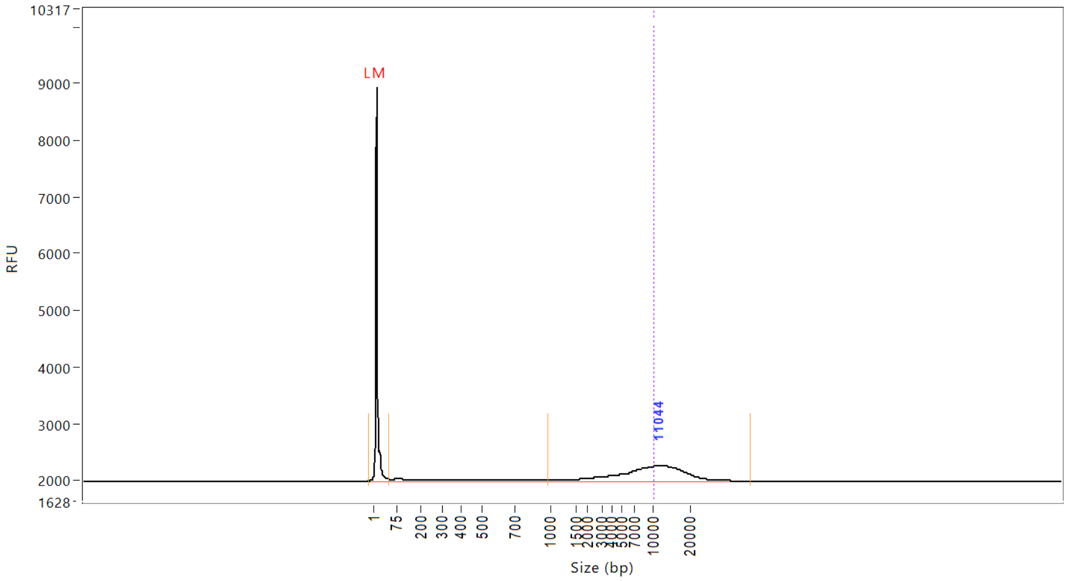

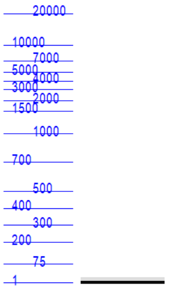

Peak	Size (bp)	Conc. (ng/uL)	From (bp)	To (bp)	Avg. Size (bp)	CV%	RFU	Corr. Peak Area
1	1 (LM)	0.0328	0	46	3	280.03	6942	48.700
2	11044	0.3470	983	36480	10470	58.80	273	42.931

TIC:	0.3470	ng/uL
TIM:	0.0546	nmole/L
Total Conc.:	0.4243	ng/uL
GQN:	3.8	

Sample Peak Width (sec): 50   Sample Min Peak Height: 50   Sample Baseline V to V?: Y   Sample Baseline V to V pts: 3
Sample Filter: Binomial   # of Pts for Filter: 3   Sample Start Region (min): 0   Sample End Region (min): 50
Manual Baseline Start (min): 6   Manual Baseline End (min): 48
Marker Peak Width (sec): 5   Marker Min Peak Height: 200   Marker Baseline V to V?: Y   Marker Baseline V to V pts: 3
Lower Marker Selection: First Peak > 200 RFU   Upper Marker Selection: Last Peak > 200 RFU
Ladder Size (bp): 1, 75, 200, 300, 400, 500, 700, 1000, 1500, 2000, 3000, 4000, 5000, 7000, 10000, 20000
Quantification Using: Ladder   Final Concentration (ng/uL): 1.0417   Dilution Factor: 12.0
Size Threshold (b.p.): 10000

 Copyright 2015 Advanced Analytical Technologies, Inc.

Data File: 2019 06 18 14H 35M.raw
Sample: 103613-001-119 (20x dil.)
Well Location: G3

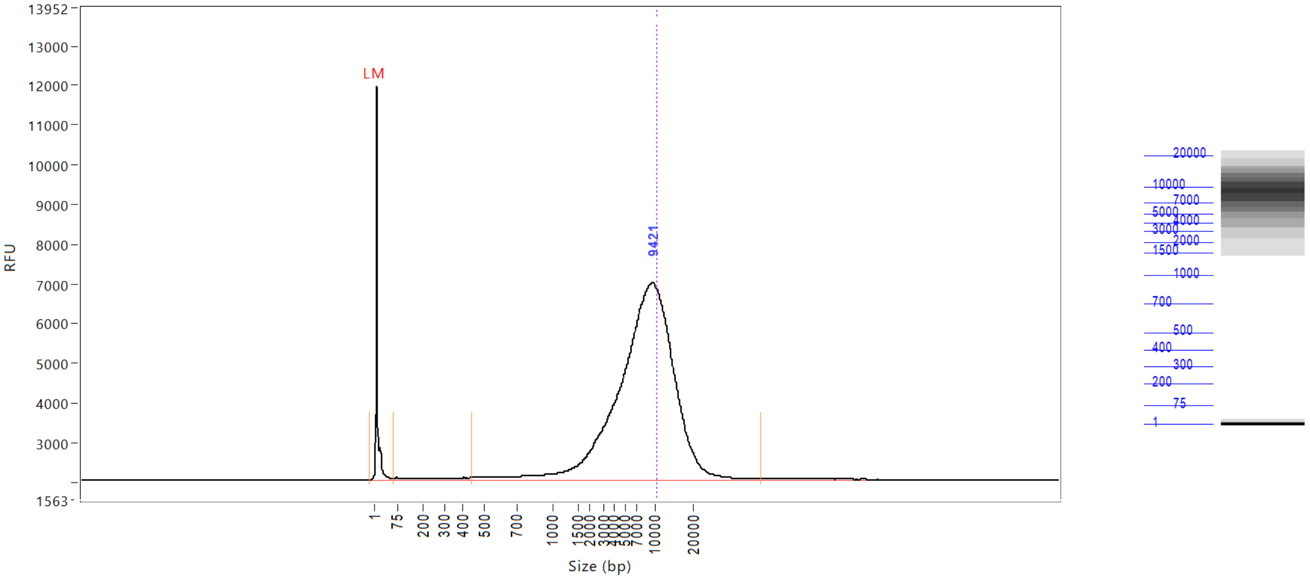

Peak	Size (bp)	Conc. (ng/uL)	From (bp)	To (bp)	Avg. Size (bp)	CV%	RFU	Corr. Peak Area
1	1 (LM)	0.0328	0	56	3	265.97	9905	70.450
2	9421	3.4545	439	38206	8547	58.74	4952	618.253

TIC:	3.4545	ng/uL
TIM:	0.6654	nmole/L
Total Conc.:	3.5509	ng/uL
GQN:	3.3	

Sample Peak Width (sec): 50 Sample Min Peak Height: 50 Sample Baseline V to V?: Y Sample Baseline V to V pts: 3
Sample Filter: Binomial # of Pts for Filter: 3 Sample Start Region (min): 0 Sample End Region (min): 50
Manual Baseline Start (min): 6 Manual Baseline End (min): 48
Marker Peak Width (sec): 5 Marker Min Peak Height: 200 Marker Baseline V to V?: Y Marker Baseline V to V pts: 3
Lower Marker Selection: First Peak > 200 RFU Upper Marker Selection: Last Peak > 200 RFU
Ladder Size (bp): 1, 75, 200, 300, 400, 500, 700, 1000, 1500, 2000, 3000, 4000, 5000, 7000, 10000, 20000
Quantification Using: Ladder Final Concentration (ng/uL): 1.0417 Dilution Factor: 12.0
Size Threshold (b.p.): 10000

 Copyright 2015 Advanced Analytical Technologies, Inc.

Data File: 2019 06 18 14H 35M.raw
Sample: 103613-001-120 (20x dil.)
Well Location: H3

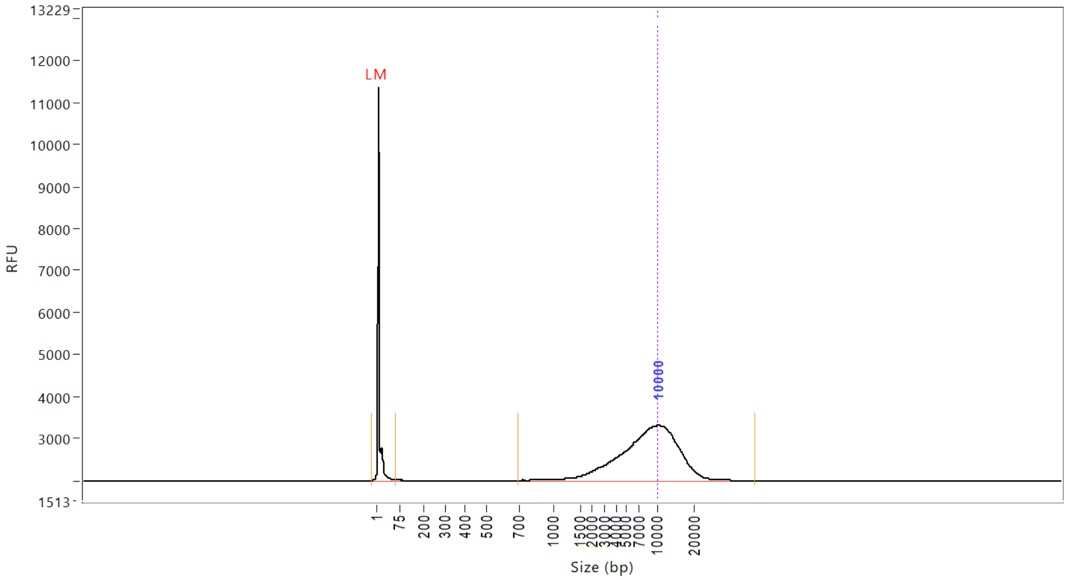

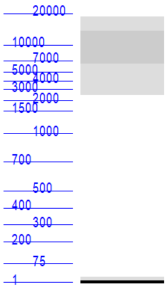

Peak	Size (bp)	Conc. (ng/uL)	From (bp)	To (bp)	Avg. Size (bp)	CV%	RFU	Corr. Peak Area
1	1 (LM)	0.0328	0	57	2	342.35	9367	65.698
2	10000	1.0526	690	36308	8974	56.50	1325	175.684

TIC:	1.0526	ng/uL
TIM:	0.1931	nmole/L
Total Conc.:	1.0879	ng/uL
GQN:	3.7	

Sample Peak Width (sec): 50 Sample Min Peak Height: 50 Sample Baseline V to V?: Y Sample Baseline V to V pts: 3
Sample Filter: Binomial # of Pts for Filter: 3 Sample Start Region (min): 0 Sample End Region (min): 50
Manual Baseline Start (min): 6 Manual Baseline End (min): 48
Marker Peak Width (sec): 5 Marker Min Peak Height: 200 Marker Baseline V to V?: Y Marker Baseline V to V pts: 3
Lower Marker Selection: First Peak > 200 RFU Upper Marker Selection: Last Peak > 200 RFU
Ladder Size (bp): 1, 75, 200, 300, 400, 500, 700, 1000, 1500, 2000, 3000, 4000, 5000, 7000, 10000, 20000
Quantification Using: Ladder Final Concentration (ng/uL): 1.0417 Dilution Factor: 12.0
Size Threshold (b.p.): 10000

 Copyright 2015 Advanced Analytical Technologies, Inc.

Data File: 2019 06 18 14H 35M.raw
Sample: 103613-001-121 (20x dil.)
Well Location: A4

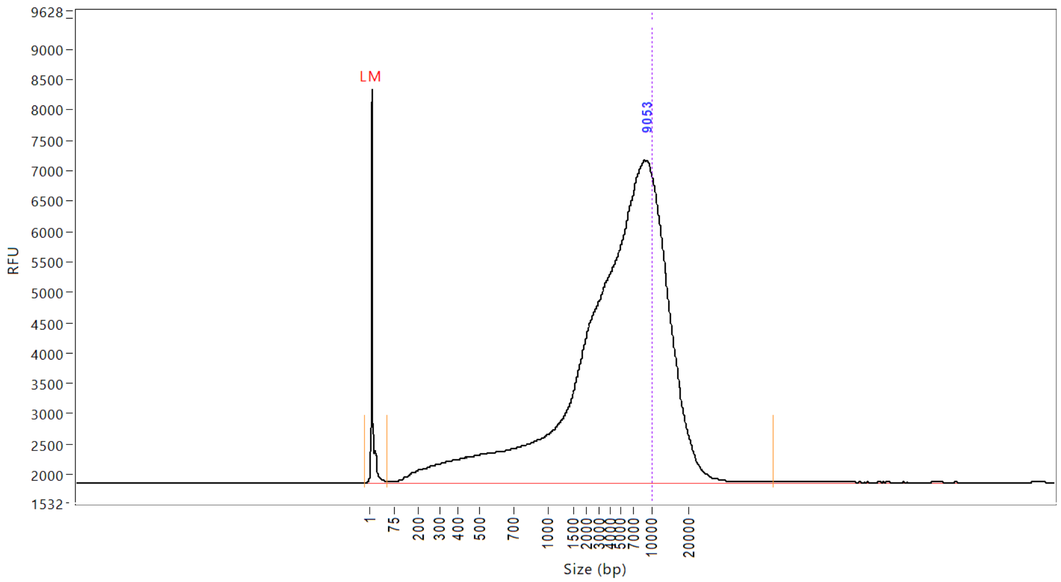

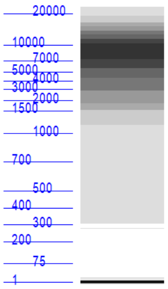

Peak	Size (bp)	Conc. (ng/uL)	From (bp)	To (bp)	Avg. Size (bp)	CV%	RFU	Corr. Peak Area
1	1 (LM)	0.0328	0	52	2	363.80	6473	45.886
2	9053	8.4676	52	42434	6094	84.79	5303	987.049

TIC:	8.4676	ng/uL
TIM:	2.2875	nmole/L
Total Conc.:	8.5093	ng/uL
GQN:	2.1	

Sample Peak Width (sec): 50 Sample Min Peak Height: 50 Sample Baseline V to V?: Y Sample Baseline V to V pts: 3
Sample Filter: Binomial # of Pts for Filter: 3 Sample Start Region (min): 0 Sample End Region (min): 50
Manual Baseline Start (min): 6 Manual Baseline End (min): 48
Marker Peak Width (sec): 5 Marker Min Peak Height: 200 Marker Baseline V to V?: Y Marker Baseline V to V pts: 3
Lower Marker Selection: First Peak > 200 RFU Upper Marker Selection: Last Peak > 200 RFU
Ladder Size (bp): 1, 75, 200, 300, 400, 500, 700, 1000, 1500, 2000, 3000, 4000, 5000, 7000, 10000, 20000
Quantification Using: Ladder Final Concentration (ng/uL): 1.0417 Dilution Factor: 12.0
Size Threshold (b.p.): 10000

 Copyright 2015 Advanced Analytical Technologies, Inc.

Data File: 2019 06 18 14H 35M.raw
Sample: 103613-001-122 (20x dil.)
Well Location: B4

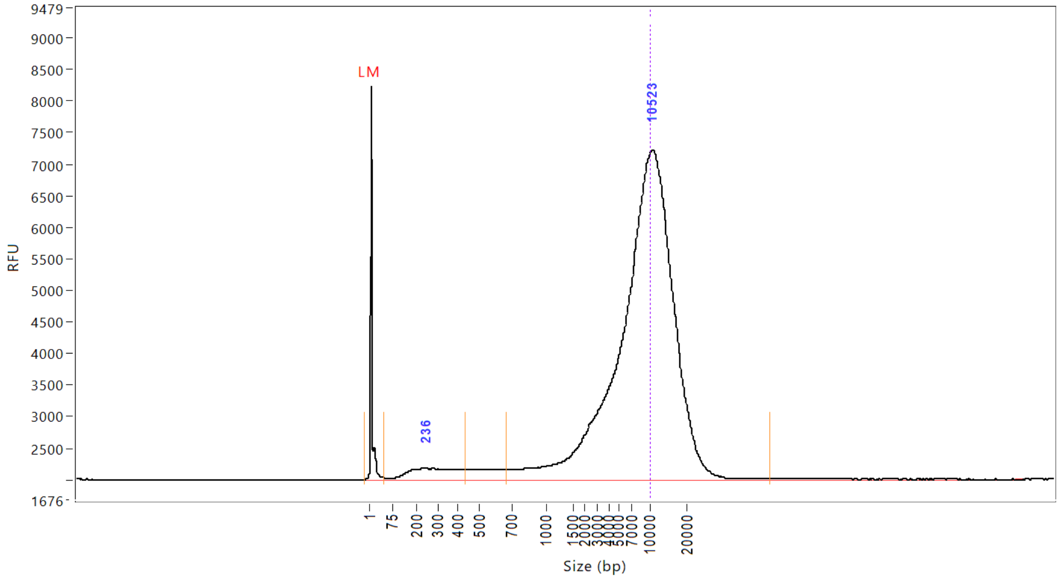

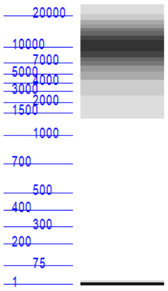

Peak	Size (bp)	Conc. (ng/uL)	From (bp)	To (bp)	Avg. Size (bp)	CV%	RFU	Corr. Peak Area
1	1 (LM)	0.0328	0	45	2	358.19	6238	43.621
2	236	0.2708	45	435	263	37.21	172	30.004
3	10523	5.8320	665	42089	9420	56.33	5224	646.277

TIC:	6.1027	ng/uL
TIM:	2.7125	nmole/L
Total Conc.:	6.2705	ng/uL
GQN:	3.9	

Sample Peak Width (sec): 50 Sample Min Peak Height: 50 Sample Baseline V to V?: Y Sample Baseline V to V pts: 3
Sample Filter: Binomial # of Pts for Filter: 3 Sample Start Region (min): 0 Sample End Region (min): 50
Manual Baseline Start (min): 6 Manual Baseline End (min): 48
Marker Peak Width (sec): 5 Marker Min Peak Height: 200 Marker Baseline V to V?: Y Marker Baseline V to V pts: 3
Lower Marker Selection: First Peak > 200 RFU Upper Marker Selection: Last Peak > 200 RFU
Ladder Size (bp): 1, 75, 200, 300, 400, 500, 700, 1000, 1500, 2000, 3000, 4000, 5000, 7000, 10000, 20000
Quantification Using: Ladder Final Concentration (ng/uL): 1.0417 Dilution Factor: 12.0
Size Threshold (b.p.): 10000

 Copyright 2015 Advanced Analytical Technologies, Inc.

Data File: 2019 06 18 14H 35M.raw
Sample: 103613-001-123 (20x dil.)
Well Location: C4

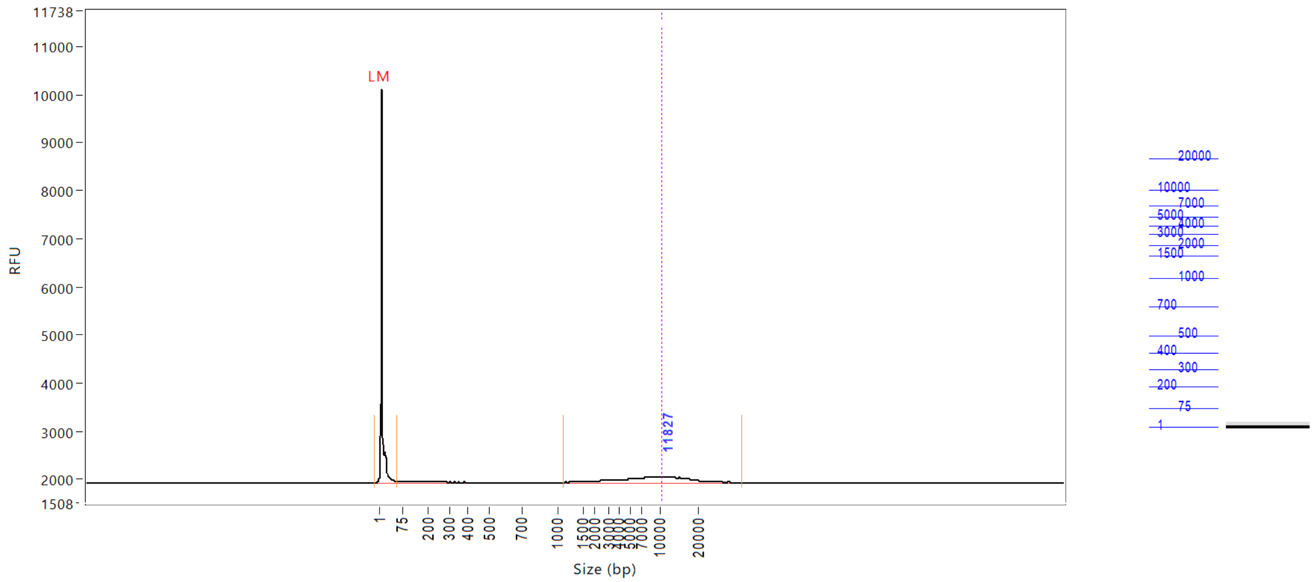

Peak	Size (bp)	Conc. (ng/uL)	From (bp)	To (bp)	Avg. Size (bp)	CV%	RFU	Corr. Peak Area
1	1 (LM)	0.0328	0	52	3	282.86	8181	57.085
2	11827	0.1566	1120	31735	9578	66.37	128	22.714

TIC:	0.1566	ng/uL
TIM:	0.0269	nmole/L
Total Conc.:	0.2246	ng/uL
GQN:	3.0	

Sample Peak Width (sec): 50 Sample Min Peak Height: 50 Sample Baseline V to V?: Y Sample Baseline V to V pts: 3
Sample Filter: Binomial # of Pts for Filter: 3 Sample Start Region (min): 0 Sample End Region (min): 50
Manual Baseline Start (min): 6 Manual Baseline End (min): 48
Marker Peak Width (sec): 5 Marker Min Peak Height: 200 Marker Baseline V to V?: Y Marker Baseline V to V pts: 3
Lower Marker Selection: First Peak > 200 RFU Upper Marker Selection: Last Peak > 200 RFU
Ladder Size (bp): 1, 75, 200, 300, 400, 500, 700, 1000, 1500, 2000, 3000, 4000, 5000, 7000, 10000, 20000
Quantification Using: Ladder Final Concentration (ng/uL): 1.0417 Dilution Factor: 12.0
Size Threshold (b.p.): 10000

 Copyright 2015 Advanced Analytical Technologies, Inc.

Data File: 2019 06 18 14H 35M.raw
Sample: 103613-001-124 (20x dil.)
Well Location: D4

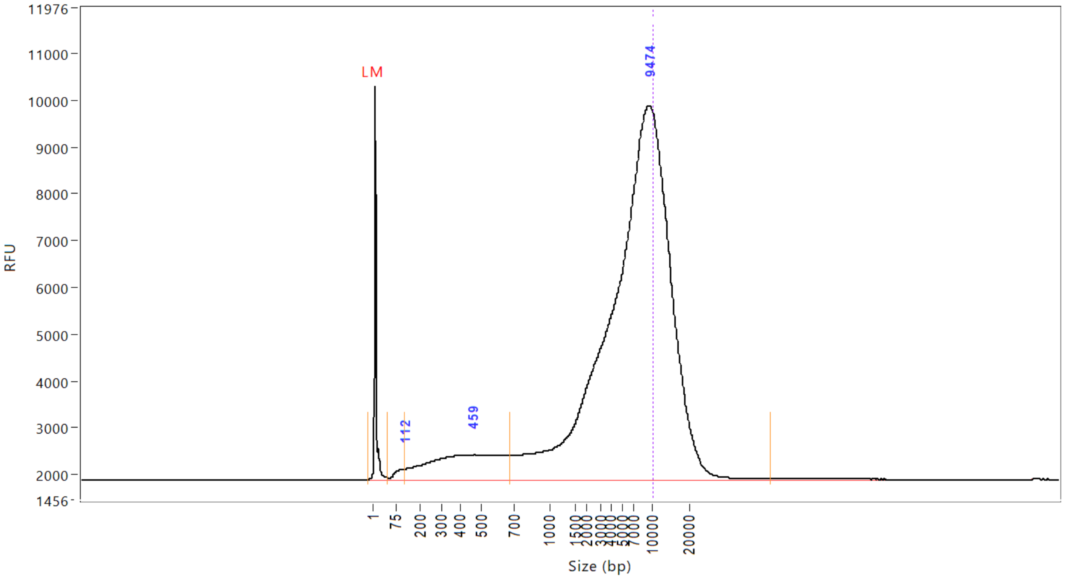

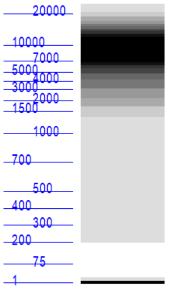

Peak	Size (bp)	Conc. (ng/uL)	From (bp)	To (bp)	Avg. Size (bp)	CV%	RFU	Corr. Peak Area
1	1 (LM)	0.0328	0	44	2	321.26	8411	58.455
2	112	0.0550	44	114	83	22.22	224	8.165
3	459	0.8562	114	673	400	37.40	535	127.147
4	9474	7.3636	673	41830	7848	65.09	7996	1093.489

TIC:	8.2748	ng/uL
TIM:	6.1538	nmole/L
Total Conc.:	8.3127	ng/uL
GQN:	2.6	

Sample Peak Width (sec): 50 Sample Min Peak Height: 50 Sample Baseline V to V?: Y Sample Baseline V to V pts: 3
Sample Filter: Binomial # of Pts for Filter: 3 Sample Start Region (min): 0 Sample End Region (min): 50
Manual Baseline Start (min): 6 Manual Baseline End (min): 48
Marker Peak Width (sec): 5 Marker Min Peak Height: 200 Marker Baseline V to V?: Y Marker Baseline V to V pts: 3
Lower Marker Selection: First Peak > 200 RFU Upper Marker Selection: Last Peak > 200 RFU
Ladder Size (bp): 1, 75, 200, 300, 400, 500, 700, 1000, 1500, 2000, 3000, 4000, 5000, 7000, 10000, 20000
Quantification Using: Ladder Final Concentration (ng/uL): 1.0417 Dilution Factor: 12.0
Size Threshold (b.p.): 10000

 Copyright 2015 Advanced Analytical Technologies, Inc.

Data File: 2019 06 18 14H 35M.raw
Sample: 103613-001-125 (20x dil.)
Well Location: E4

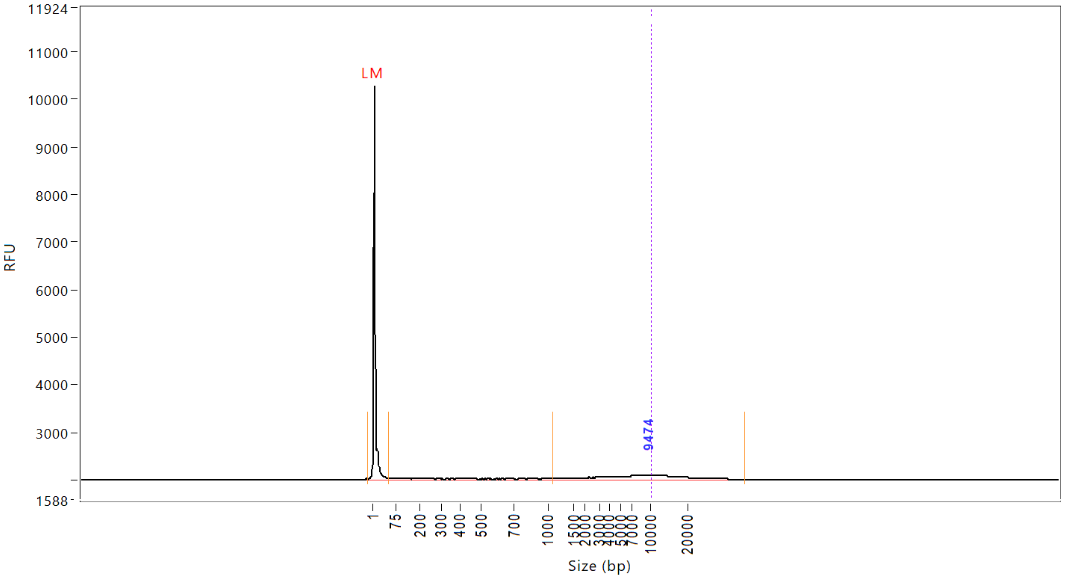

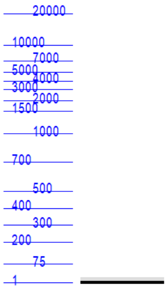

Peak	Size (bp)	Conc. (ng/uL)	From (bp)	To (bp)	Avg. Size (bp)	CV%	RFU	Corr. Peak Area
1	1 (LM)	0.0328	0	52	2	298.28	8258	57.498
2	9474	0.1268	1088	35445	10375	74.28	85	18.528

TIC:	0.1268	ng/uL
TIM:	0.0201	nmole/L
Total Conc.:	0.1861	ng/uL
GQN:	3.5	

Sample Peak Width (sec): 50 Sample Min Peak Height: 50 Sample Baseline V to V?: Y Sample Baseline V to V pts: 3
Sample Filter: Binomial # of Pts for Filter: 3 Sample Start Region (min): 0 Sample End Region (min): 50
Manual Baseline Start (min): 6 Manual Baseline End (min): 48
Marker Peak Width (sec): 5 Marker Min Peak Height: 200 Marker Baseline V to V?: Y Marker Baseline V to V pts: 3
Lower Marker Selection: First Peak > 200 RFU Upper Marker Selection: Last Peak > 200 RFU
Ladder Size (bp): 1, 75, 200, 300, 400, 500, 700, 1000, 1500, 2000, 3000, 4000, 5000, 7000, 10000, 20000
Quantification Using: Ladder Final Concentration (ng/uL): 1.0417 Dilution Factor: 12.0
Size Threshold (b.p.): 10000

 Copyright 2015 Advanced Analytical Technologies, Inc.

Data File: 2019 06 18 14H 35M.raw
Sample: 103613-001-126 (20x dil.)
Well Location: F4

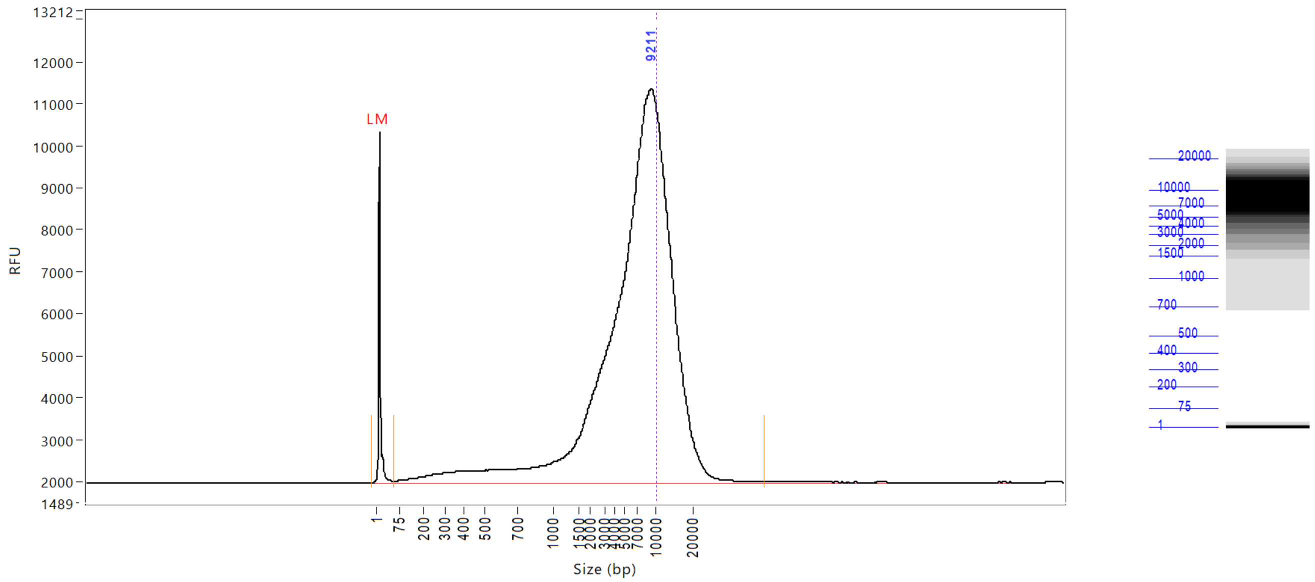

Peak	Size (bp)	Conc. (ng/uL)	From (bp)	To (bp)	Avg. Size (bp)	CV%	RFU	Corr. Peak Area
1	1 (LM)	0.0328	0	52	3	257.35	8369	59.035
2	9211	8.1644	52	39328	7511	66.33	9366	1224.450

TIC:	8.1644	ng/uL
TIM:	1.7895	nmole/L
Total Conc.:	8.1902	ng/uL
GQN:	2.8	

```
Sample Peak Width (sec): 50   Sample Min Peak Height: 50    Sample Baseline V to V?: Y    Sample Baseline V to V pts: 3
Sample Filter: Binomial       # of Pts for Filter: 3        Sample Start Region (min): 0  Sample End Region (min): 50
Manual Baseline Start (min): 6     Manual Baseline End (min): 48
Marker Peak Width (sec): 5    Marker Min Peak Height: 200   Marker Baseline V to V?: Y    Marker Baseline V to V pts: 3
Lower Marker Selection: First Peak > 200 RFU                Upper Marker Selection: Last Peak > 200 RFU
Ladder Size (bp): 1, 75, 200, 300, 400, 500, 700, 1000, 1500, 2000, 3000, 4000, 5000, 7000, 10000, 20000
Quantification Using: Ladder          Final Concentration (ng/uL): 1.0417          Dilution Factor: 12.0
Size Threshold (b.p.): 10000
```


Copyright 2015 Advanced Analytical Technologies, Inc.

Data File: 2019 06 18 14H 35M.raw
Sample: 103613-001-127 (20x dil.)
Well Location: G4

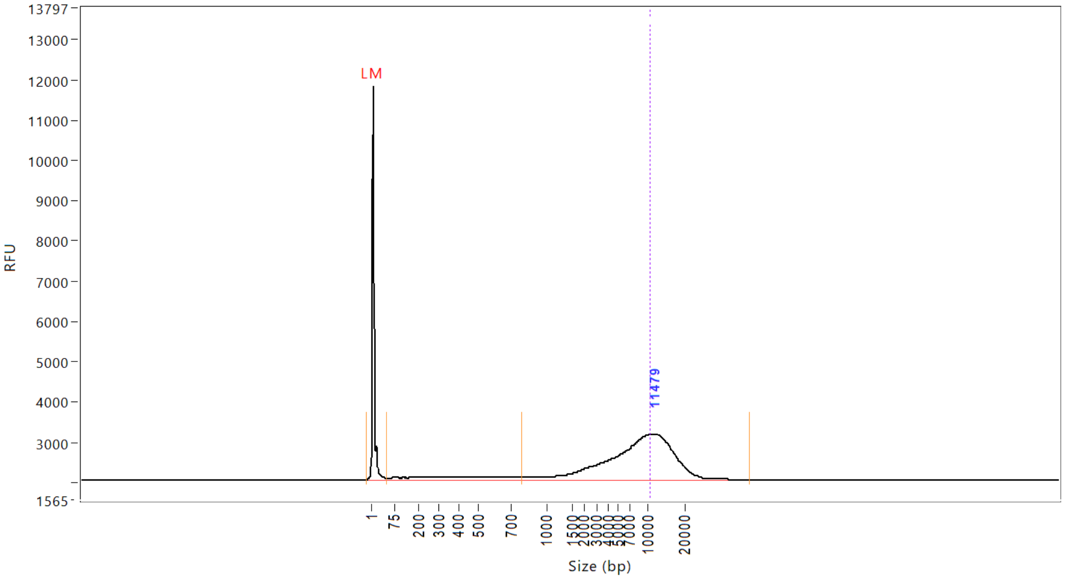

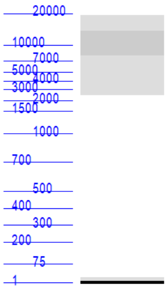

Peak	Size (bp)	Conc. (ng/uL)	From (bp)	To (bp)	Avg. Size (bp)	CV%	RFU	Corr. Peak Area
1	1 (LM)	0.0328	0	49	2	369.96	9781	69.998
2	11479	0.9595	779	37516	9026	62.76	1149	170.622

TIC:	0.9595	ng/uL
TIM:	0.1750	nmole/L
Total Conc.:	1.0997	ng/uL
GQN:	3.5	

Sample Peak Width (sec): 50   Sample Min Peak Height: 50   Sample Baseline V to V?: Y   Sample Baseline V to V pts: 3
Sample Filter: Binomial   # of Pts for Filter: 3   Sample Start Region (min): 0   Sample End Region (min): 50
Manual Baseline Start (min): 6   Manual Baseline End (min): 48
Marker Peak Width (sec): 5   Marker Min Peak Height: 200   Marker Baseline V to V?: Y   Marker Baseline V to V pts: 3
Lower Marker Selection: First Peak > 200 RFU   Upper Marker Selection: Last Peak > 200 RFU
Ladder Size (bp): 1, 75, 200, 300, 400, 500, 700, 1000, 1500, 2000, 3000, 4000, 5000, 7000, 10000, 20000
Quantification Using: Ladder   Final Concentration (ng/uL): 1.0417   Dilution Factor: 12.0
Size Threshold (b.p.): 10000

 Copyright 2015 Advanced Analytical Technologies, Inc.

Data File: 2019 06 18 14H 35M.raw
Sample: 103613-001-128 (20x dil.)
Well Location: H4

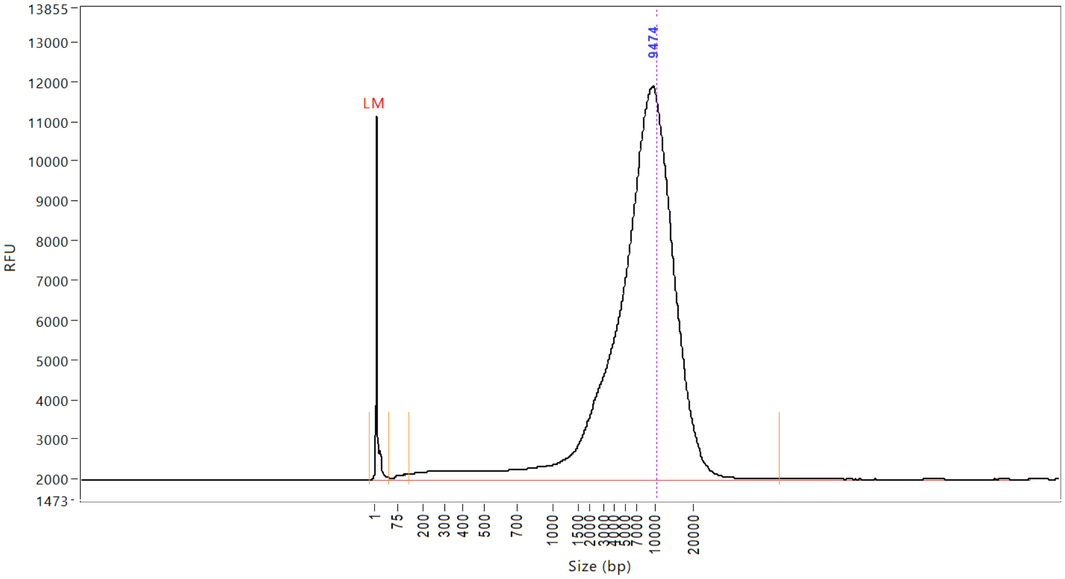

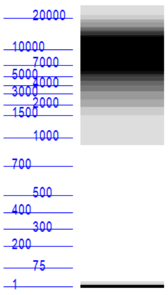

Peak	Size (bp)	Conc. (ng/uL)	From (bp)	To (bp)	Avg. Size (bp)	CV%	RFU	Corr. Peak Area
1	1 (LM)	0.0328	0	46	2	277.85	9150	63.888
2	9474	7.7474	129	43038	8129	63.34	9894	1257.417

TIC:	7.7474	ng/uL
TIM:	1.5690	nmole/L
Total Conc.:	7.8234	ng/uL
GQN:	3.1	

Sample Peak Width (sec): 50 Sample Min Peak Height: 50 Sample Baseline V to V?: Y Sample Baseline V to V pts: 3
Sample Filter: Binomial # of Pts for Filter: 3 Sample Start Region (min): 0 Sample End Region (min): 50
Manual Baseline Start (min): 6 Manual Baseline End (min): 48
Marker Peak Width (sec): 5 Marker Min Peak Height: 200 Marker Baseline V to V?: Y Marker Baseline V to V pts: 3
Lower Marker Selection: First Peak > 200 RFU Upper Marker Selection: Last Peak > 200 RFU
Ladder Size (bp): 1, 75, 200, 300, 400, 500, 700, 1000, 1500, 2000, 3000, 4000, 5000, 7000, 10000, 20000
Quantification Using: Ladder Final Concentration (ng/uL): 1.0417 Dilution Factor: 12.0
Size Threshold (b.p.): 10000

 Copyright 2015 Advanced Analytical Technologies, Inc.

Data File: 2019 06 18 14H 35M.raw
Sample: 103613-001-129 (20x dil.)
Well Location: A5

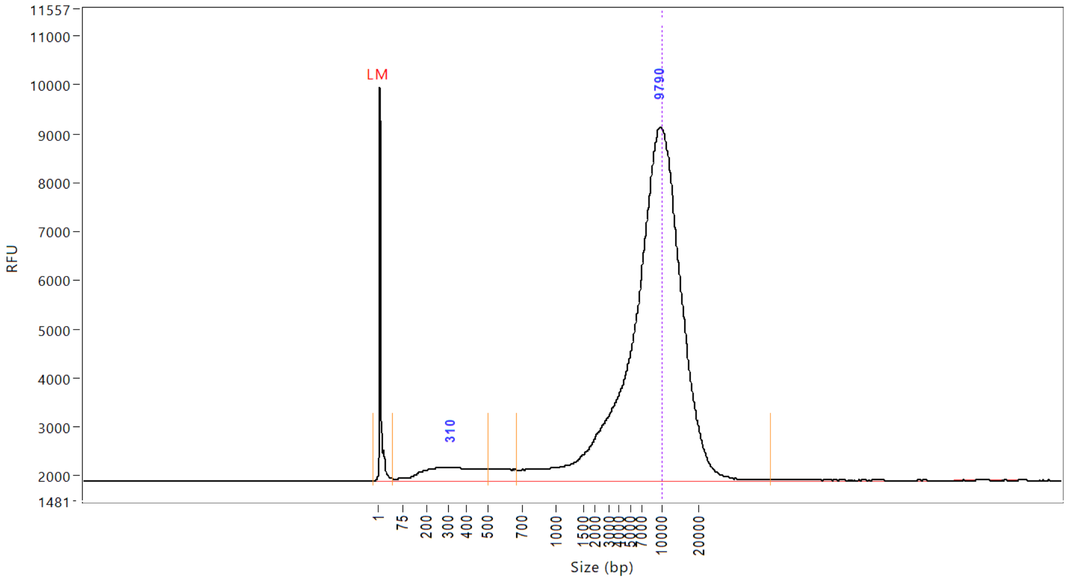

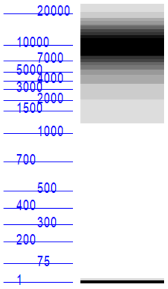

Peak	Size (bp)	Conc. (ng/uL)	From (bp)	To (bp)	Avg. Size (bp)	CV%	RFU	Corr. Peak Area
1	1 (LM)	0.0328	0	43	2	344.59	8055	54.245
2	310	0.3609	43	494	298	38.09	255	49.739
3	9790	5.8692	667	38896	9099	54.38	7229	808.802

TIC:	6.2301	ng/uL
TIM:	3.0543	nmole/L
Total Conc.:	6.3598	ng/uL
GQN:	3.6	

Sample Peak Width (sec): 50 Sample Min Peak Height: 50 Sample Baseline V to V?: Y Sample Baseline V to V pts: 3
Sample Filter: Binomial # of Pts for Filter: 3 Sample Start Region (min): 0 Sample End Region (min): 50
Manual Baseline Start (min): 6 Manual Baseline End (min): 48
Marker Peak Width (sec): 5 Marker Min Peak Height: 200 Marker Baseline V to V?: Y Marker Baseline V to V pts: 3
Lower Marker Selection: First Peak > 200 RFU Upper Marker Selection: Last Peak > 200 RFU
Ladder Size (bp): 1, 75, 200, 300, 400, 500, 700, 1000, 1500, 2000, 3000, 4000, 5000, 7000, 10000, 20000
Quantification Using: Ladder Final Concentration (ng/uL): 1.0417 Dilution Factor: 12.0
Size Threshold (b.p.): 10000

 Copyright 2015 Advanced Analytical Technologies, Inc.

Data File: 2019 06 18 14H 35M.raw
Sample: 103613-001-130 (20x dil.)
Well Location: B5

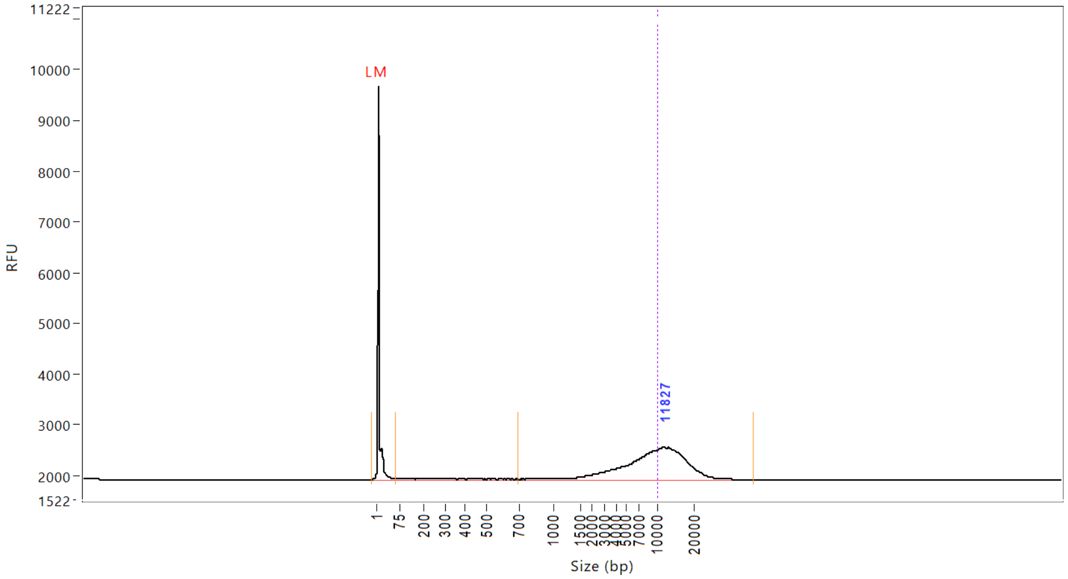

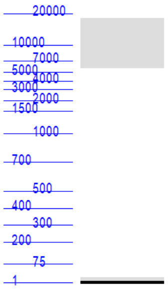

Peak	Size (bp)	Conc. (ng/uL)	From (bp)	To (bp)	Avg. Size (bp)	CV%	RFU	Corr. Peak Area
1	1 (LM)	0.0328	0	57	2	311.14	7755	53.879
2	11827	0.6749	688	35790	10128	57.25	644	92.375

TIC:	0.6749	ng/uL
TIM:	0.1097	nmole/L
Total Conc.:	0.7293	ng/uL

GQN: 4.4

```
Sample Peak Width (sec): 50    Sample Min Peak Height: 50    Sample Baseline V to V?: Y    Sample Baseline V to V pts: 3
Sample Filter: Binomial        # of Pts for Filter: 3        Sample Start Region (min): 0  Sample End Region (min): 50
Manual Baseline Start (min): 6      Manual Baseline End (min): 48
Marker Peak Width (sec): 5    Marker Min Peak Height: 200    Marker Baseline V to V?: Y    Marker Baseline V to V pts: 3
Lower Marker Selection: First Peak > 200 RFU                  Upper Marker Selection: Last Peak > 200 RFU
Ladder Size (bp): 1, 75, 200, 300, 400, 500, 700, 1000, 1500, 2000, 3000, 4000, 5000, 7000, 10000, 20000
Quantification Using: Ladder          Final Concentration (ng/uL): 1.0417          Dilution Factor: 12.0
Size Threshold (b.p.): 10000
```

 Copyright 2015 Advanced Analytical Technologies, Inc.

Data File: 2019 06 18 14H 35M.raw
Sample: 103613-001-131 (20x dil.)
Well Location: C5

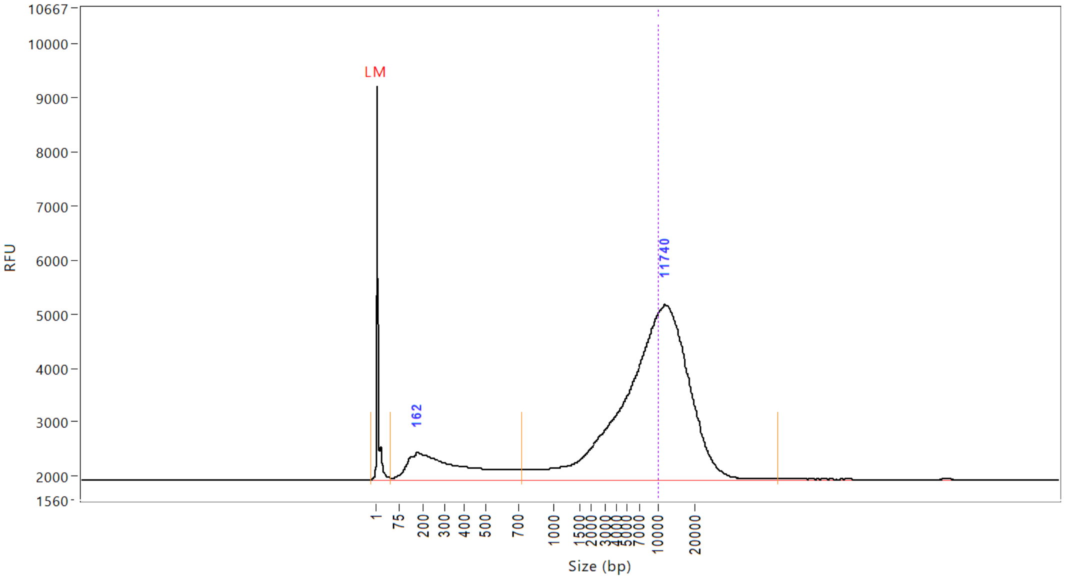

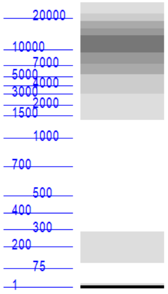

Peak	Size (bp)	Conc. (ng/uL)	From (bp)	To (bp)	Avg. Size (bp)	CV%	RFU	Corr. Peak Area
1	1 (LM)	0.0328	0	44	2	395.22	7283	52.157
2	162	0.6937	44	718	311	54.40	499	91.916
3	11740	3.8993	718	42262	9943	62.05	3238	516.658

TIC:	4.5930	ng/uL
TIM:	4.3149	nmole/L
Total Conc.:	4.6247	ng/uL
GQN:	3.8	

Sample Peak Width (sec): 50   Sample Min Peak Height: 50   Sample Baseline V to V?: Y   Sample Baseline V to V pts: 3
Sample Filter: Binomial   # of Pts for Filter: 3   Sample Start Region (min): 0   Sample End Region (min): 50
Manual Baseline Start (min): 6   Manual Baseline End (min): 48
Marker Peak Width (sec): 5   Marker Min Peak Height: 200   Marker Baseline V to V?: Y   Marker Baseline V to V pts: 3
Lower Marker Selection: First Peak > 200 RFU   Upper Marker Selection: Last Peak > 200 RFU
Ladder Size (bp): 1, 75, 200, 300, 400, 500, 700, 1000, 1500, 2000, 3000, 4000, 5000, 7000, 10000, 20000
Quantification Using: Ladder   Final Concentration (ng/uL): 1.0417   Dilution Factor: 12.0
Size Threshold (b.p.): 10000

 Copyright 2015 Advanced Analytical Technologies, Inc.

Data File: 2019 06 18 14H 35M.raw
Sample: 103613-001-132 (20x dil.)
Well Location: D5

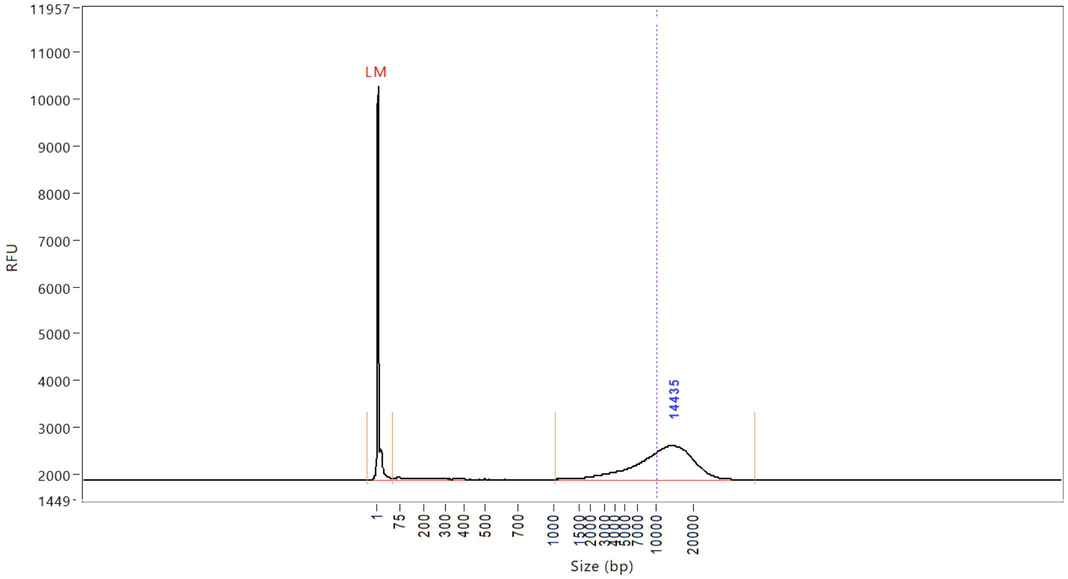

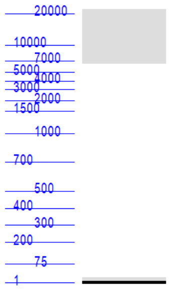

Peak	Size (bp)	Conc. (ng/uL)	From (bp)	To (bp)	Avg. Size (bp)	CV%	RFU	Corr. Peak Area
1	1 (LM)	0.0328	0	52	2	367.93	8401	59.306
2	14435	0.7239	1038	36308	12162	49.76	742	109.062

TIC:	0.7239	ng/uL
TIM:	0.0980	nmole/L
Total Conc.:	0.7954	ng/uL
GQN:	5.5	

Sample Peak Width (sec): 50   Sample Min Peak Height: 50   Sample Baseline V to V?: Y   Sample Baseline V to V pts: 3
Sample Filter: Binomial   # of Pts for Filter: 3   Sample Start Region (min): 0   Sample End Region (min): 50
Manual Baseline Start (min): 6   Manual Baseline End (min): 48
Marker Peak Width (sec): 5   Marker Min Peak Height: 200   Marker Baseline V to V?: Y   Marker Baseline V to V pts: 3
Lower Marker Selection: First Peak > 200 RFU   Upper Marker Selection: Last Peak > 200 RFU
Ladder Size (bp): 1, 75, 200, 300, 400, 500, 700, 1000, 1500, 2000, 3000, 4000, 5000, 7000, 10000, 20000
Quantification Using: Ladder   Final Concentration (ng/uL): 1.0417   Dilution Factor: 12.0
Size Threshold (b.p.): 10000

 Copyright 2015 Advanced Analytical Technologies, Inc.

Data File: 2019 06 18 14H 35M.raw
Sample: 103613-001-133 (20x dil.)
Well Location: E5

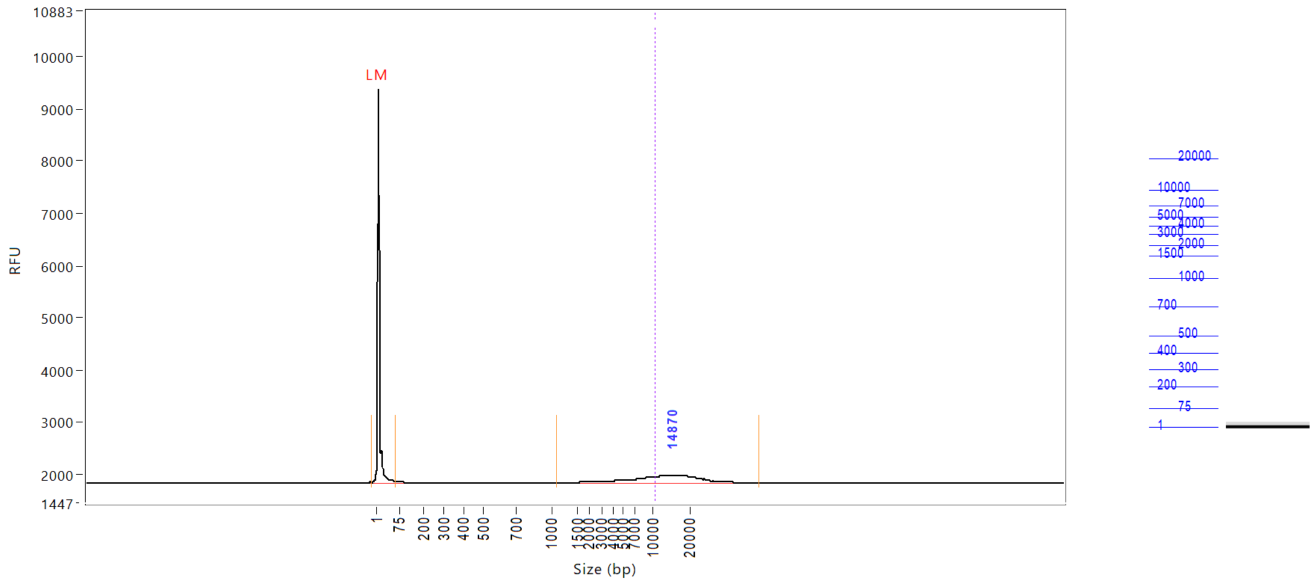

Peak	Size (bp)	Conc. (ng/uL)	From (bp)	To (bp)	Avg. Size (bp)	CV%	RFU	Corr. Peak Area
1	1 (LM)	0.0328	0	60	3	262.71	7541	54.432
2	14870	0.1952	1101	38638	13416	55.82	146	26.986

TIC:	0.1952	ng/uL
TIM:	0.0239	nmole/L
Total Conc.:	0.2387	ng/uL
GQN:	5.5	

Sample Peak Width (sec): 50 Sample Min Peak Height: 50 Sample Baseline V to V?: Y Sample Baseline V to V pts: 3
Sample Filter: Binomial # of Pts for Filter: 3 Sample Start Region (min): 0 Sample End Region (min): 50
Manual Baseline Start (min): 6 Manual Baseline End (min): 48
Marker Peak Width (sec): 5 Marker Min Peak Height: 200 Marker Baseline V to V?: Y Marker Baseline V to V pts: 3
Lower Marker Selection: First Peak > 200 RFU Upper Marker Selection: Last Peak > 200 RFU
Ladder Size (bp): 1, 75, 200, 300, 400, 500, 700, 1000, 1500, 2000, 3000, 4000, 5000, 7000, 10000, 20000
Quantification Using: Ladder Final Concentration (ng/uL): 1.0417 Dilution Factor: 12.0
Size Threshold (b.p.): 10000

 Copyright 2015 Advanced Analytical Technologies, Inc.

Data File: 2019 06 18 14H 35M.raw
Sample: 103613-001-134 (20x dil.)
Well Location: F5

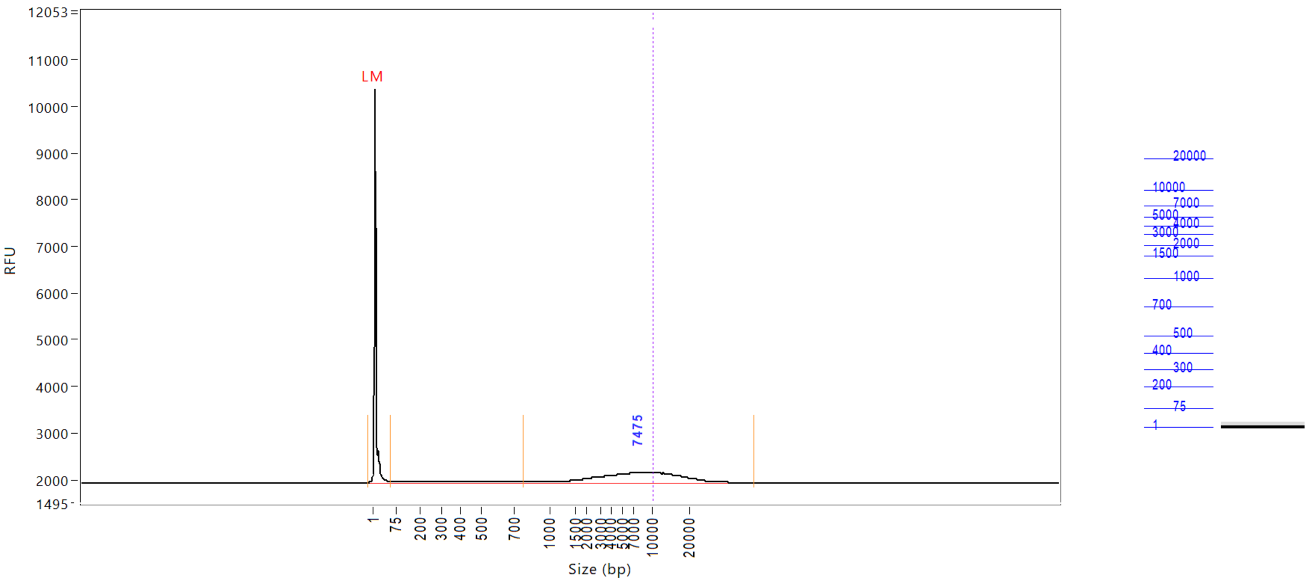

Peak	Size (bp)	Conc. (ng/uL)	From (bp)	To (bp)	Avg. Size (bp)	CV%	RFU	Corr. Peak Area
1	1 (LM)	0.0328	0	55	2	377.58	8443	60.643
2	7475	0.3142	768	37343	8782	77.22	226	48.400

TIC:	0.3142	ng/uL
TIM:	0.0589	nmole/L
Total Conc.:	0.3825	ng/uL
GQN:	2.9	

Sample Peak Width (sec): 50   Sample Min Peak Height: 50   Sample Baseline V to V?: Y   Sample Baseline V to V pts: 3
Sample Filter: Binomial   # of Pts for Filter: 3   Sample Start Region (min): 0   Sample End Region (min): 50
Manual Baseline Start (min): 6   Manual Baseline End (min): 48
Marker Peak Width (sec): 5   Marker Min Peak Height: 200   Marker Baseline V to V?: Y   Marker Baseline V to V pts: 3
Lower Marker Selection: First Peak > 200 RFU   Upper Marker Selection: Last Peak > 200 RFU
Ladder Size (bp): 1, 75, 200, 300, 400, 500, 700, 1000, 1500, 2000, 3000, 4000, 5000, 7000, 10000, 20000
Quantification Using: Ladder   Final Concentration (ng/uL): 1.0417   Dilution Factor: 12.0
Size Threshold (b.p.): 10000

Data File: 2019 06 18 14H 35M.raw
Sample: 103613-001-135 (20x dil.)
Well Location: G5

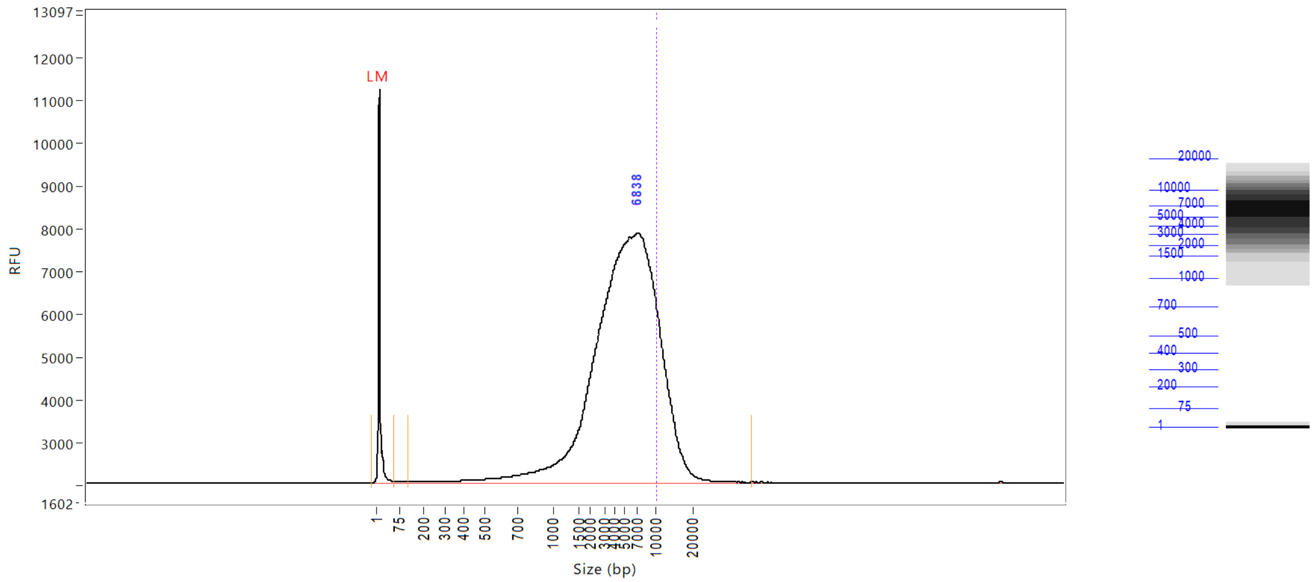

Peak	Size (bp)	Conc. (ng/uL)	From (bp)	To (bp)	Avg. Size (bp)	CV%	RFU	Corr. Peak Area
1	1 (LM)	0.0328	0	51	2	351.35	9192	65.005
2	6838	5.1028	116	35876	5850	67.46	5847	842.672

TIC:	5.1028	ng/uL
TIM:	1.4360	nmole/L
Total Conc.:	5.1356	ng/uL
GQN:	1.4	

Sample Peak Width (sec): 50 Sample Min Peak Height: 50 Sample Baseline V to V?: Y Sample Baseline V to V pts: 3
Sample Filter: Binomial # of Pts for Filter: 3 Sample Start Region (min): 0 Sample End Region (min): 50
Manual Baseline Start (min): 6 Manual Baseline End (min): 48
Marker Peak Width (sec): 5 Marker Min Peak Height: 200 Marker Baseline V to V?: Y Marker Baseline V to V pts: 3
Lower Marker Selection: First Peak > 200 RFU Upper Marker Selection: Last Peak > 200 RFU
Ladder Size (bp): 1, 75, 200, 300, 400, 500, 700, 1000, 1500, 2000, 3000, 4000, 5000, 7000, 10000, 20000
Quantification Using: Ladder Final Concentration (ng/uL): 1.0417 Dilution Factor: 12.0
Size Threshold (b.p.): 10000

 Copyright 2015 Advanced Analytical Technologies, Inc.

Data File: 2019 06 18 14H 35M.raw
Sample: 103613-001-136 (20x dil.)
Well Location: H5

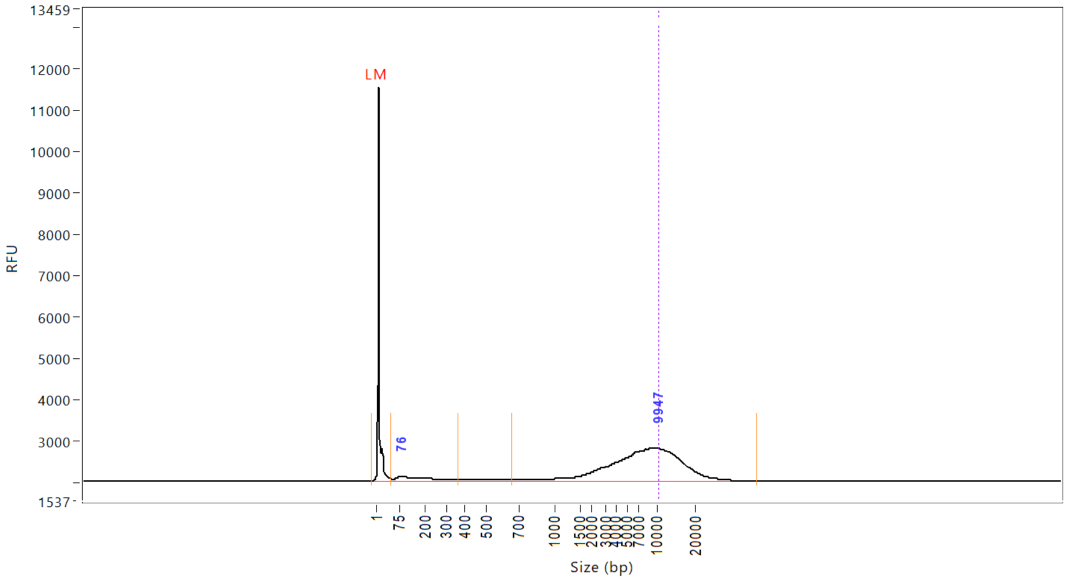

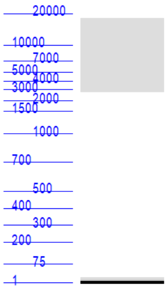

Peak	Size (bp)	Conc. (ng/uL)	From (bp)	To (bp)	Avg. Size (bp)	CV%	RFU	Corr. Peak Area
1	1 (LM)	0.0328	0	45	2	306.22	9533	66.455
2	76	0.0715	45	362	169	53.22	105	12.066
3	9947	0.7928	657	36739	8665	67.28	795	133.842

TIC:	0.8643	ng/uL
TIM:	0.8458	nmole/L
Total Conc.:	0.9127	ng/uL
GQN:	3.1	

```
Sample Peak Width (sec): 50   Sample Min Peak Height: 50    Sample Baseline V to V?: Y    Sample Baseline V to V pts: 3
Sample Filter: Binomial       # of Pts for Filter: 3        Sample Start Region (min): 0  Sample End Region (min): 50
Manual Baseline Start (min): 6     Manual Baseline End (min): 48
Marker Peak Width (sec): 5    Marker Min Peak Height: 200   Marker Baseline V to V?: Y    Marker Baseline V to V pts: 3
Lower Marker Selection: First Peak > 200 RFU                Upper Marker Selection: Last Peak > 200 RFU
Ladder Size (bp): 1, 75, 200, 300, 400, 500, 700, 1000, 1500, 2000, 3000, 4000, 5000, 7000, 10000, 20000
Quantification Using: Ladder         Final Concentration (ng/uL): 1.0417          Dilution Factor: 12.0
Size Threshold (b.p.): 10000
```

 Copyright 2015 Advanced Analytical Technologies, Inc.

Data File: 2019 06 18 14H 35M.raw
Sample: 103613-001-137 (20x dil.)
Well Location: A6

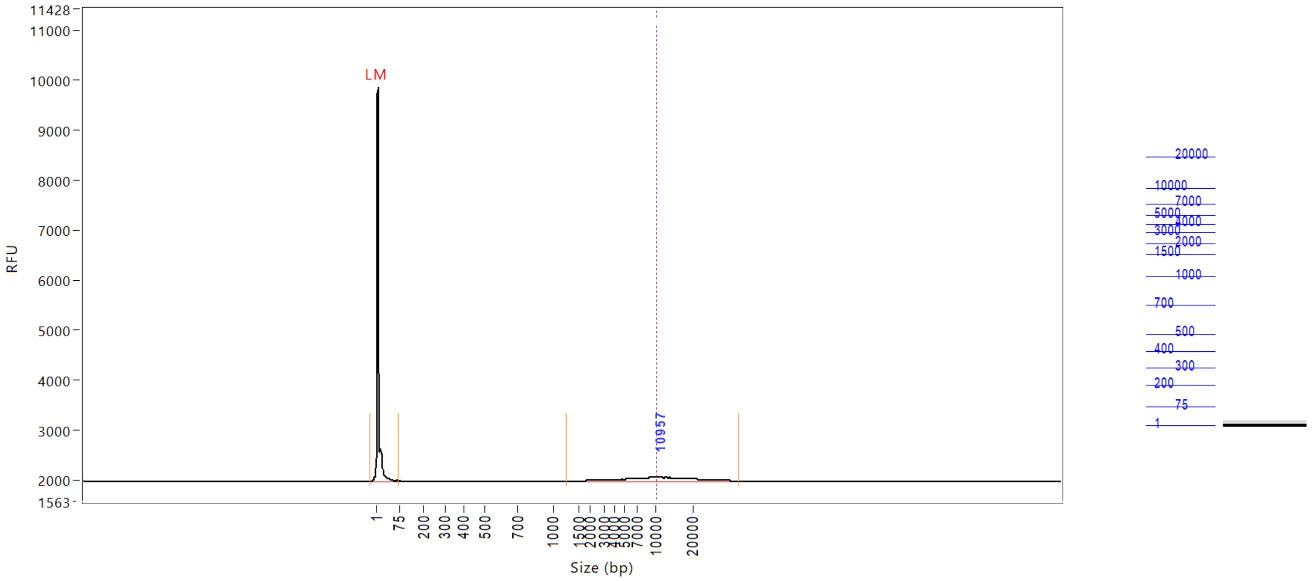

Peak	Size (bp)	Conc. (ng/uL)	From (bp)	To (bp)	Avg. Size (bp)	CV%	RFU	Corr. Peak Area
1	1 (LM)	0.0328	0	71	2	375.78	7888	56.511
2	10957	0.1085	1269	32339	11501	61.96	77	15.579

TIC:	0.1085	ng/uL
TIM:	0.0155	nmole/L
Total Conc.:	0.1295	ng/uL
GQN:	4.7	

Sample Peak Width (sec): 50   Sample Min Peak Height: 50   Sample Baseline V to V?: Y   Sample Baseline V to V pts: 3
Sample Filter: Binomial   # of Pts for Filter: 3   Sample Start Region (min): 0   Sample End Region (min): 50
Manual Baseline Start (min): 6   Manual Baseline End (min): 48
Marker Peak Width (sec): 5   Marker Min Peak Height: 200   Marker Baseline V to V?: Y   Marker Baseline V to V pts: 3
Lower Marker Selection: First Peak > 200 RFU   Upper Marker Selection: Last Peak > 200 RFU
Ladder Size (bp): 1, 75, 200, 300, 400, 500, 700, 1000, 1500, 2000, 3000, 4000, 5000, 7000, 10000, 20000
Quantification Using: Ladder   Final Concentration (ng/uL): 1.0417   Dilution Factor: 12.0
Size Threshold (b.p.): 10000

 Copyright 2015 Advanced Analytical Technologies, Inc.

Data File: 2019 06 18 14H 35M.raw
Sample: 103613-001-138 (20x dil.)
Well Location: B6

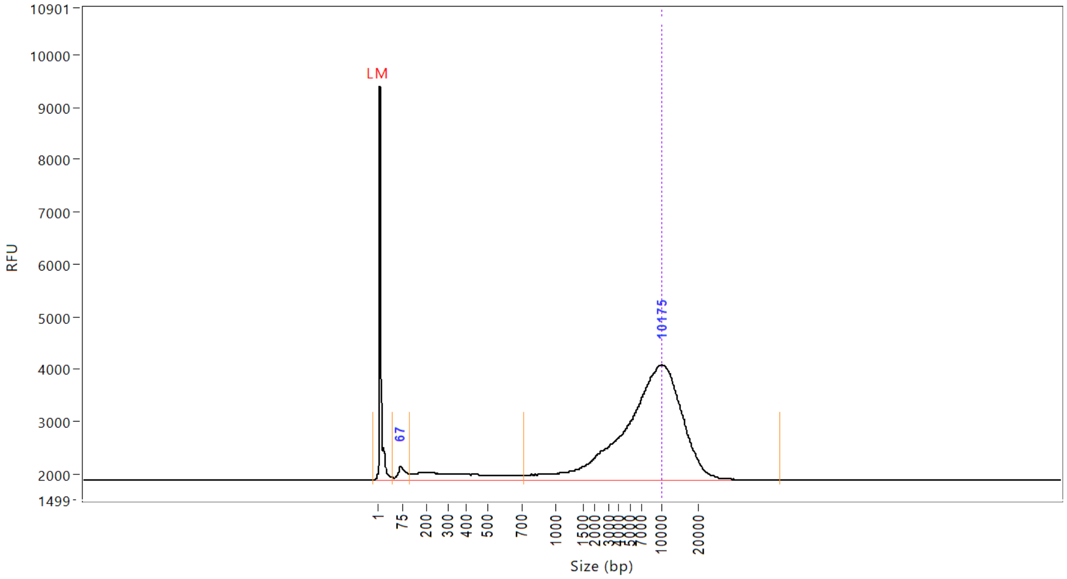

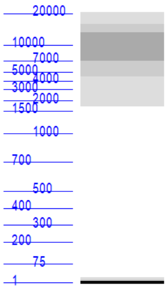

Peak	Size (bp)	Conc. (ng/uL)	From (bp)	To (bp)	Avg. Size (bp)	CV%	RFU	Corr. Peak Area
1	1 (LM)	0.0328	0	44	3	255.39	7517	52.938
2	67	0.0550	44	111	74	21.93	245	7.403
3	10175	2.1444	709	41830	8627	59.97	2195	288.384

TIC:	2.1994	ng/uL
TIM:	1.6297	nmole/L
Total Conc.:	2.4550	ng/uL
GQN:	3.1	

Sample Peak Width (sec): 50 Sample Min Peak Height: 50 Sample Baseline V to V?: Y Sample Baseline V to V pts: 3
Sample Filter: Binomial # of Pts for Filter: 3 Sample Start Region (min): 0 Sample End Region (min): 50
Manual Baseline Start (min): 6 Manual Baseline End (min): 48
Marker Peak Width (sec): 5 Marker Min Peak Height: 200 Marker Baseline V to V?: Y Marker Baseline V to V pts: 3
Lower Marker Selection: First Peak > 200 RFU Upper Marker Selection: Last Peak > 200 RFU
Ladder Size (bp): 1, 75, 200, 300, 400, 500, 700, 1000, 1500, 2000, 3000, 4000, 5000, 7000, 10000, 20000
Quantification Using: Ladder Final Concentration (ng/uL): 1.0417 Dilution Factor: 12.0
Size Threshold (b.p.): 10000

 Copyright 2015 Advanced Analytical Technologies, Inc.

Fragment Analyzer Run Summary:

Filename and Data Path: X:\Lopende Opdrachten\GAII\103613\PrepQC_NGS HS_103665_103165-031_103613_103724_103641 15-56-34\2019 06 21 15H 56M.raw
Created: Friday, June 21, 2019 4:25:48 PM
# of Capillaries: 42
Array Serial #: 112118-01SFS
Effect Length: 33 cm
Array Usage Count: 228
FA Version #: 1.2.0.11
Device Serial #: 3003

METHOD INFORMATION
Method Name: DNF-474-33 - HS NGS Fragment 1-6000bp.mthds
Gel Prime: No
Full Conditioning: Yes
Gel Prime to Buffer: No
Gel Selection: Gel 1
Perform Prerun: 6.0 kV, 30 sec.
Rinse: No
Marker 1: No
Rinse: Tray: 3, Row: A, # Dips: 1
Sample Injection: 5.0 kV, 30 sec.
Separation: 6.0 kV, 50.0 min.
Tray Name: PrepQC_NGS HS_103665_103165-031_103613_103724_103641

Analysis Mode: NGS

NOTES

Copyright 2015 Advanced Analytical Technologies, Inc.

Gel Image

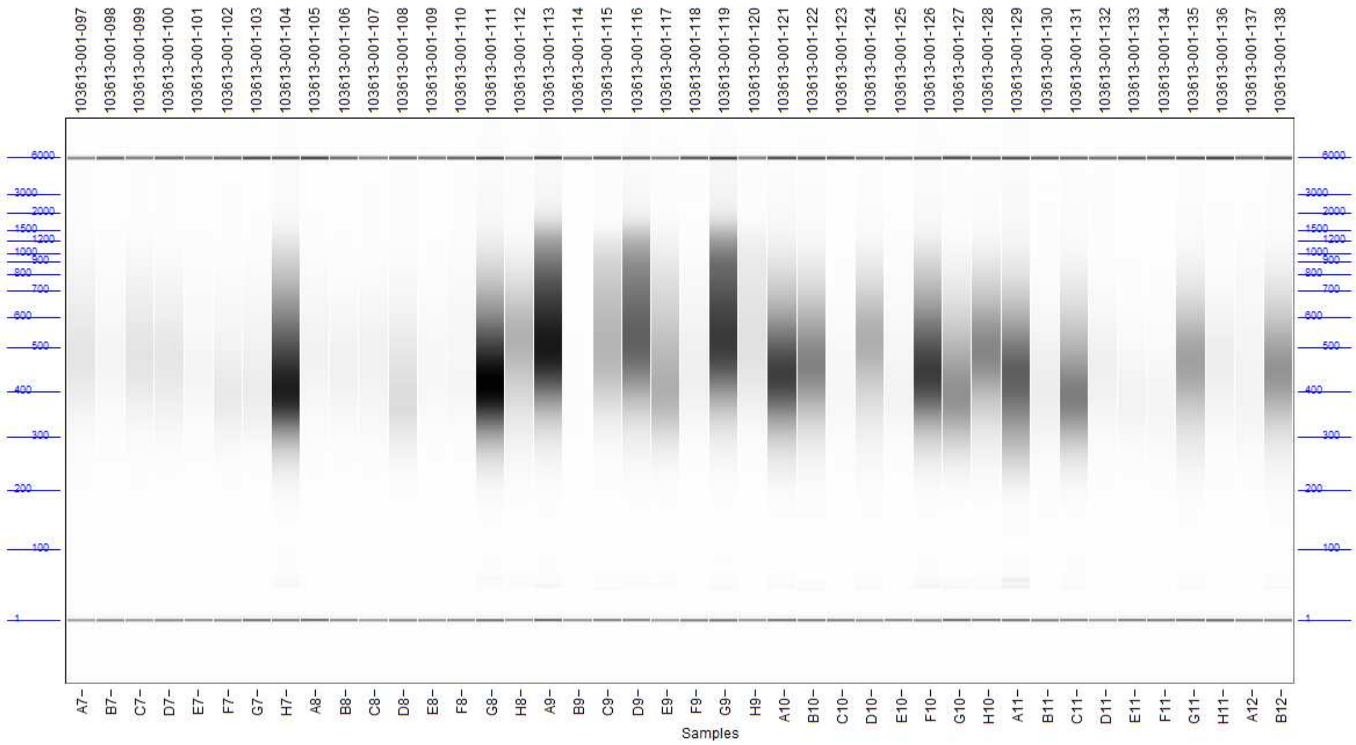

Copyright 2015 Advanced Analytical Technologies, Inc.

Filename and Data Path: X:\Lopende Opdrachten\GAII\103613\PrepQC_NGS HS_103665_103165-031_103613_103724_103641 15-56-34\2019 06 21 15H 56M.raw

A7: 103613-001-097

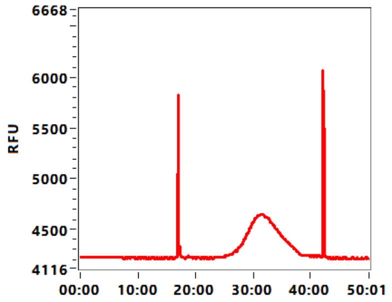

B7: 103613-001-098

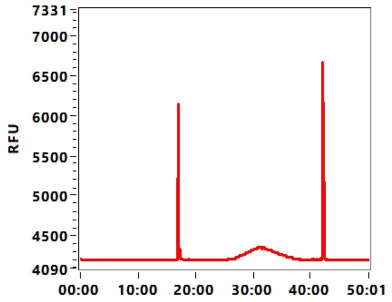

C7: 103613-001-099

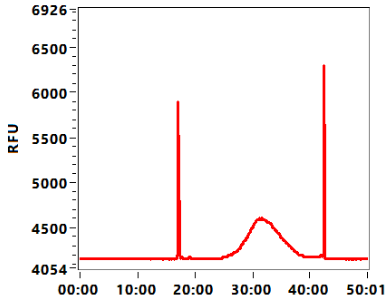

D7: 103613-001-100

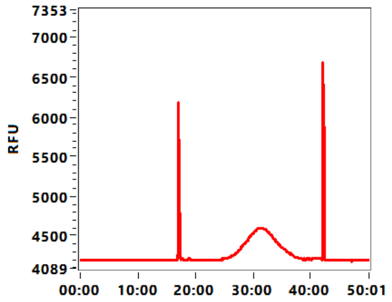

E7: 103613-001-101

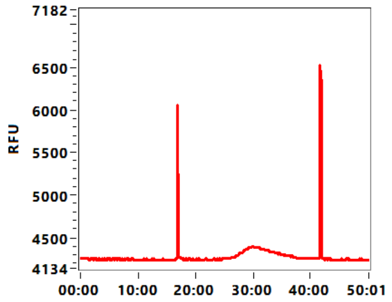

F7: 103613-001-102

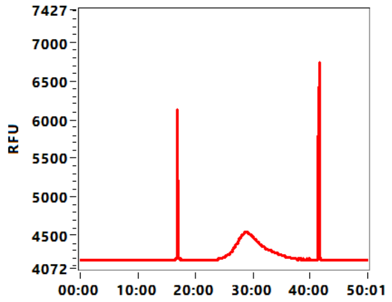

G7: 103613-001-103

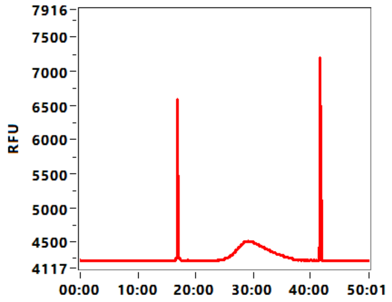

H7: 103613-001-104

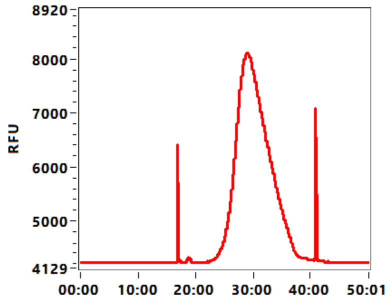

A8: 103613-001-105

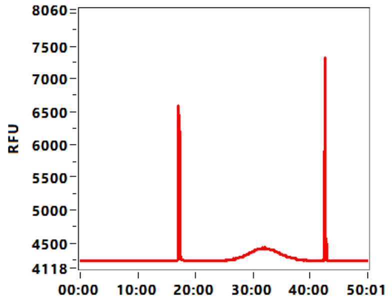

B8: 103613-001-106

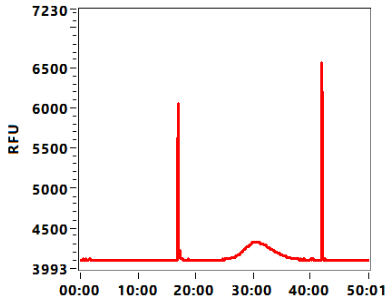

C8: 103613-001-107

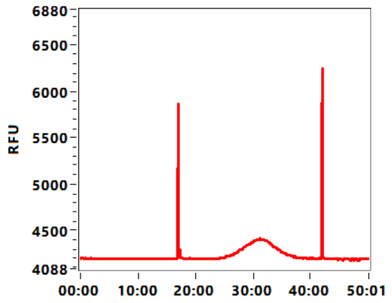

D8: 103613-001-108

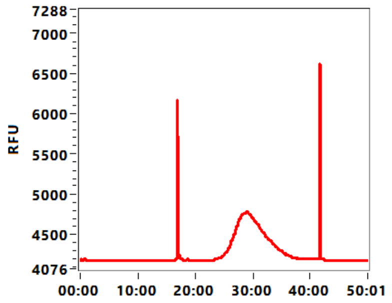

Copyright 2015 Advanced Analytical Technologies, Inc.

Filename and Data Path: X:\Lopende Opdrachten\GAII\103613\PrepQC_NGS HS_103665_103165-031_103613_103724_103641 15-56-34\2019 06 21 15H 56M.raw

E8: 103613-001-109

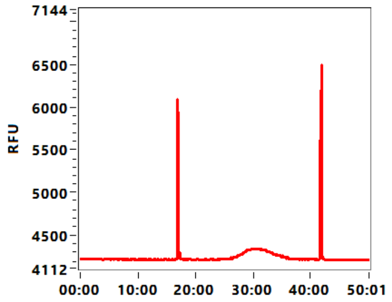

F8: 103613-001-110

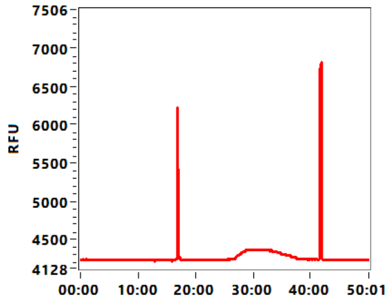

G8: 103613-001-111

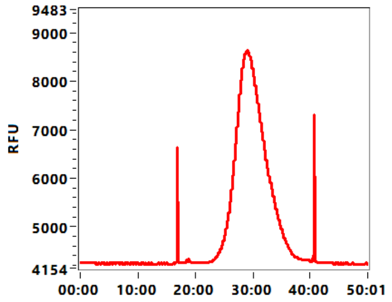

H8: 103613-001-112

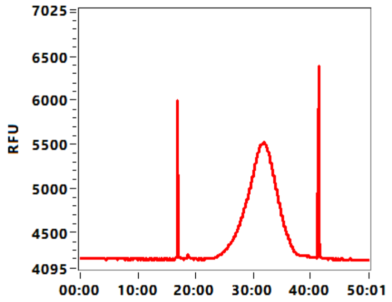

A9: 103613-001-113

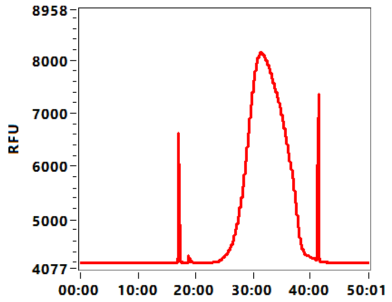

B9: 103613-001-114

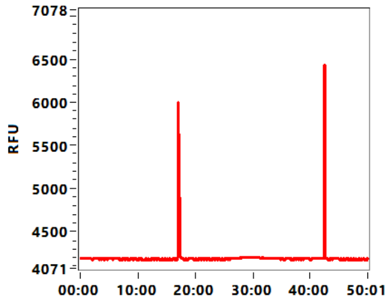

C9: 103613-001-115

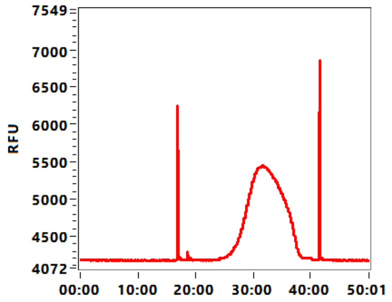

D9: 103613-001-116

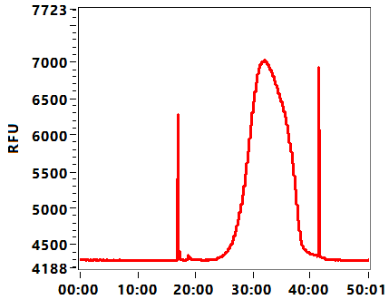

E9: 103613-001-117

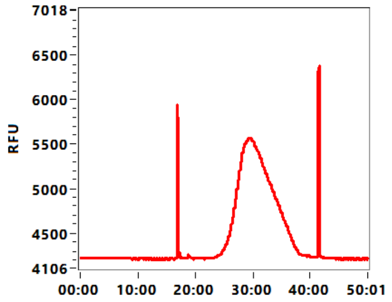

F9: 103613-001-118

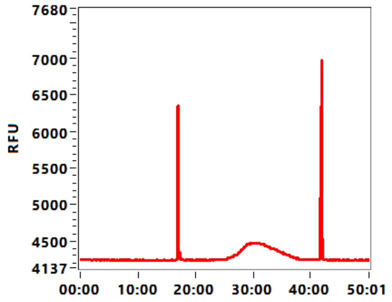

G9: 103613-001-119

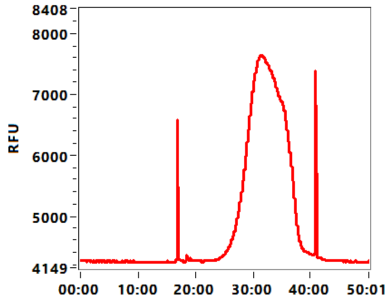

H9: 103613-001-120

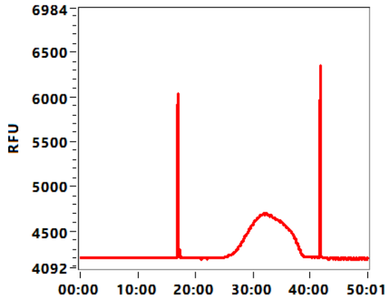

Copyright 2015 Advanced Analytical Technologies, Inc.

Filename and Data Path: X:\Lopende Opdrachten\GAII\103613\PrepQC_NGS HS_103665_103165-031_103613_103724_103641 15-56-34\2019 06 21 15H 56M.raw

A10: 103613-001-121

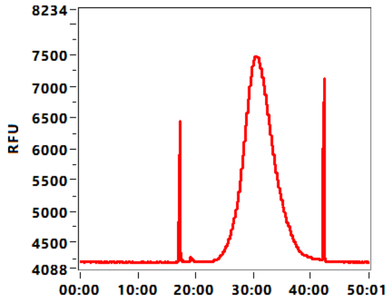

B10: 103613-001-122

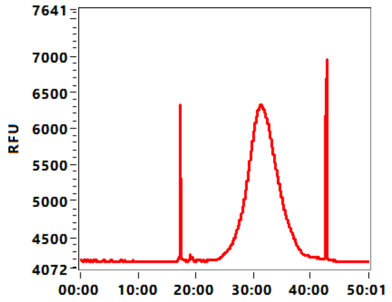

C10: 103613-001-123

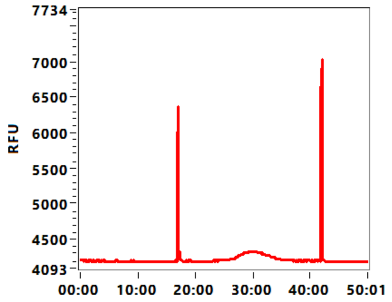

D10: 103613-001-124

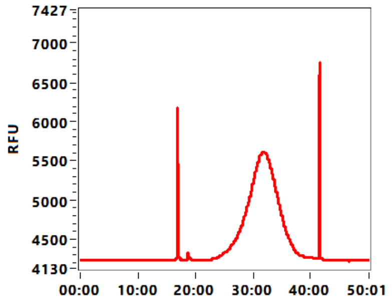

E10: 103613-001-125

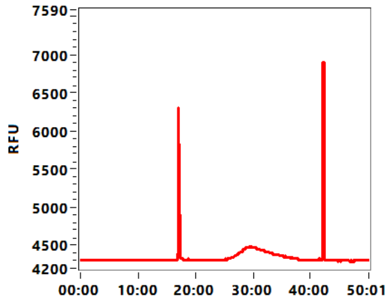

F10: 103613-001-126

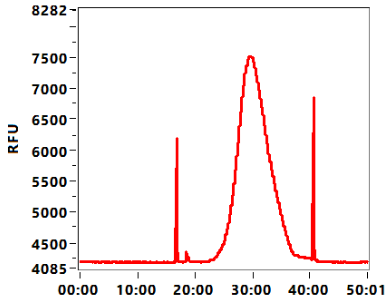

G10: 103613-001-127

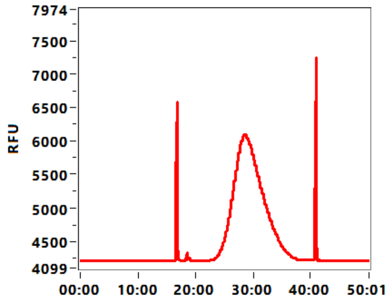

H10: 103613-001-128

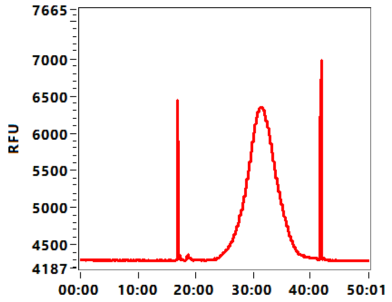

A11: 103613-001-129

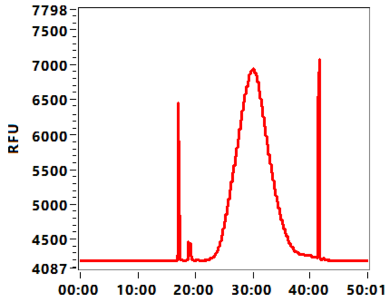

B11: 103613-001-130

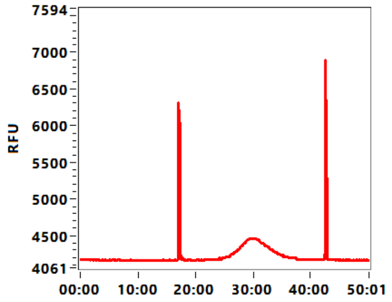

C11: 103613-001-131

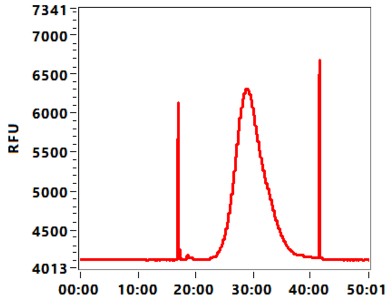

D11: 103613-001-132

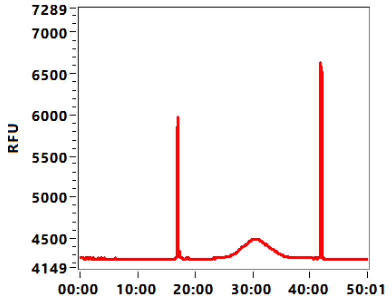

Copyright 2015 Advanced Analytical Technologies, Inc.

Filename and Data Path: X:\Lopende Opdrachten\GAII\103613\PrepQC_NGS HS_103665_103165-031_103613_103724_103641 15-56-34\2019 06 21 15H 56M.raw

E11: 103613-001-133

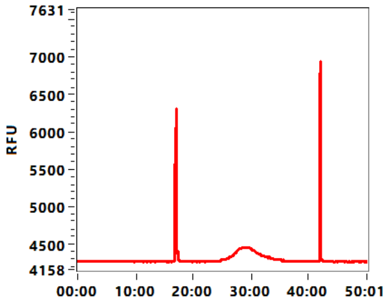

F11: 103613-001-134

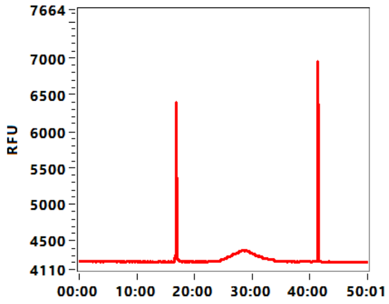

G11: 103613-001-135

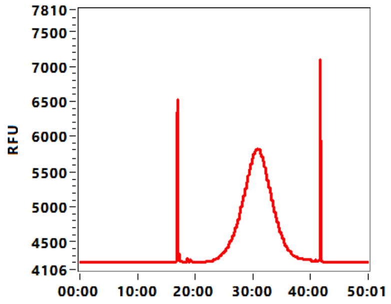

H11: 103613-001-136

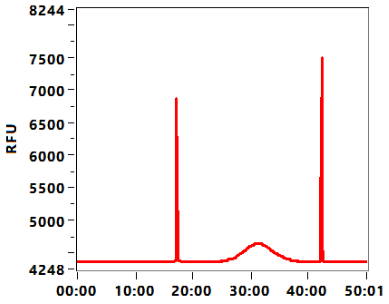

A12: 103613-001-137

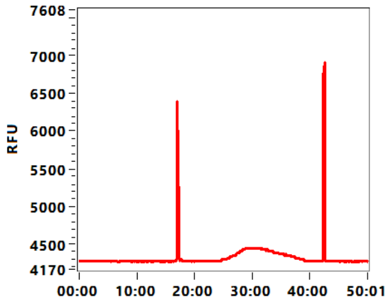

B12: 103613-001-138

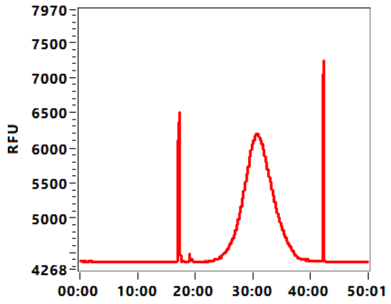

Copyright 2015 Advanced Analytical Technologies, Inc.

Sample: 103613-001-097
Well Location: A7
Created: Friday, June 21, 2019 4:25:48 PM

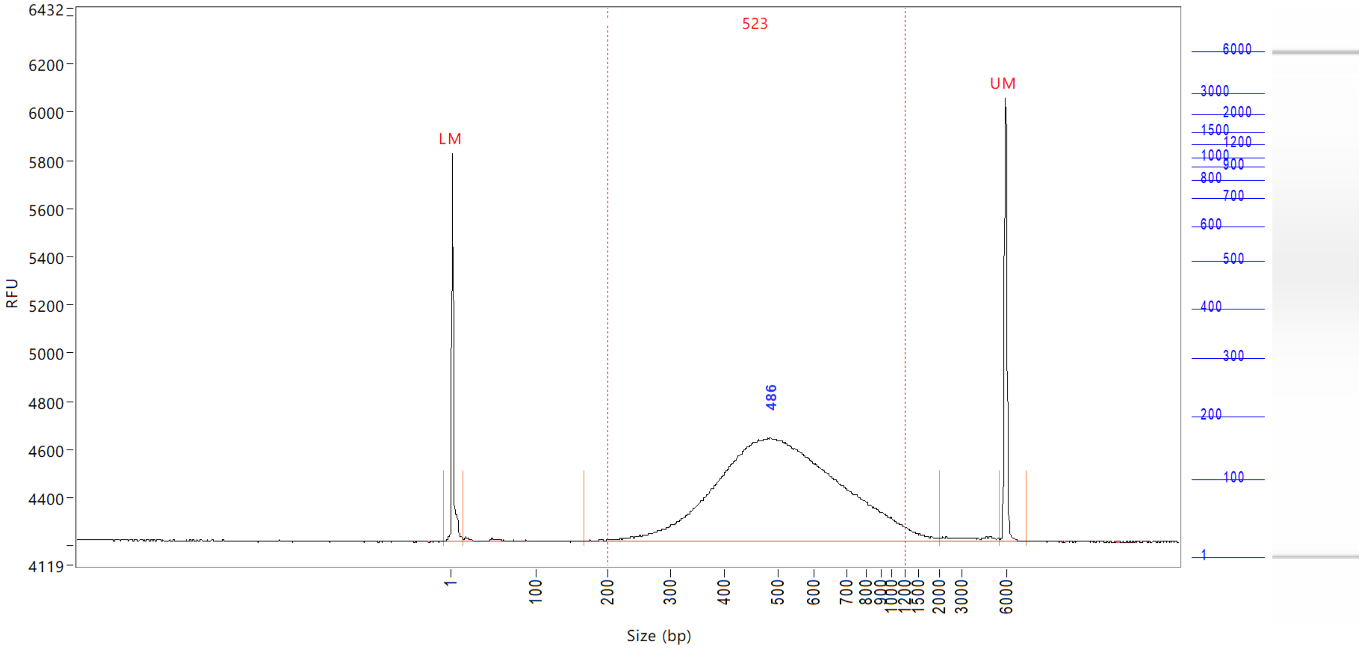

Peak	Size (bp)	Conc. (ng/uL)	From (bp)	To (bp)	RFU
1	1 (LM)	0.0103	0	14	1611
2	486	1.1042	166	2018	431
3	6000 (UM)	0.0067	5593	7364	1846

TIC:	1.1042	ng/uL
TIM:	3.3965	nmole/L
Total Conc.:	1.1316	ng/uL

Smear Analysis	200 bp to 1200 bp	1.0862 ng/ul	96.0 %Total	3.4192 nmole/L	523 Avg. Size (b.p.)	30.14 %CV

Sample Peak Width (sec): 50 Sample Min Peak Height: 25 Sample Baseline V to V?: Y Sample Baseline V to V pts: 3
Sample Filter: Binomial # of Pts for Filter: 3 Sample Start Region (min): 0 Sample End Region (min): 50
Manual Baseline Start (min): 10 Manual Baseline End (min): 48
Marker Peak Width (sec): 5 Marker Min Peak Height: 200 Marker Baseline V to V?: Y Marker Baseline V to V pts: 3
Lower Marker Selection: First Peak > 200 RFU Upper Marker Selection: Last Peak > 200 RFU
Ladder Size (bp): 1, 100, 200, 300, 400, 500, 600, 700, 800, 900, 1000, 1200, 1500, 2000, 3000, 6000
Quantification Using: Ladder Final Concentration (ng/uL): 0.0830 Dilution Factor: 12.0

 Copyright 2015 Advanced Analytical Technologies, Inc.

Sample: 103613-001-098
Well Location: B7
Created: Friday, June 21, 2019 4:25:48 PM

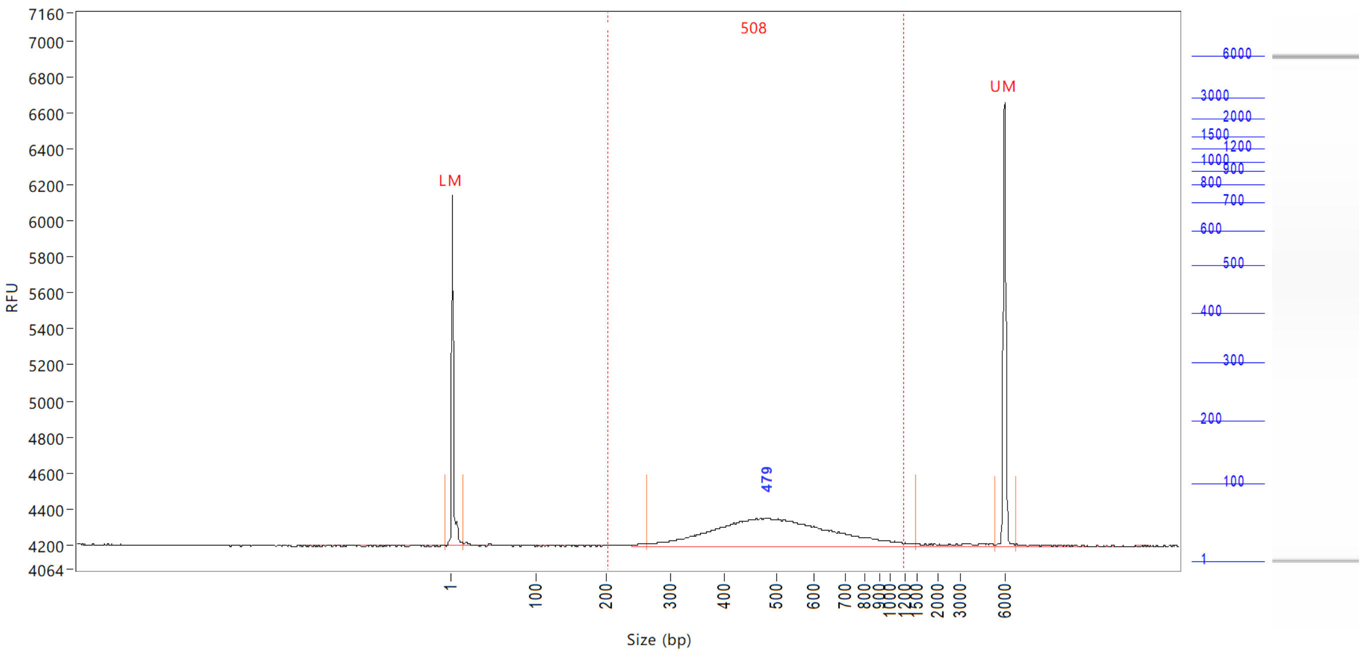

Peak	Size (bp)	Conc. (ng/uL)	From (bp)	To (bp)	RFU
1	1 (LM)	0.0103	0	15	1950
2	479	0.3104	262	1473	155
3	6000 (UM)	0.0073	5365	6732	2473
	TIC:	0.3104	ng/uL		
	TIM:	0.9879	nmole/L		
	Total Conc.:	0.3304	ng/uL		

Smear Analysis 200 bp to 1200 bp 0.3125 ng/ul 94.6 %Total 1.0120 nmole/L 508 Avg. Size (b.p.) 29.60 %CV

Sample Peak Width (sec): 50 Sample Min Peak Height: 25 Sample Baseline V to V?: Y Sample Baseline V to V pts: 3
Sample Filter: Binomial # of Pts for Filter: 3 Sample Start Region (min): 0 Sample End Region (min): 50
Manual Baseline Start (min): 10 Manual Baseline End (min): 48
Marker Peak Width (sec): 5 Marker Min Peak Height: 200 Marker Baseline V to V?: Y Marker Baseline V to V pts: 3
Lower Marker Selection: First Peak > 200 RFU Upper Marker Selection: Last Peak > 200 RFU
Ladder Size (bp): 1, 100, 200, 300, 400, 500, 600, 700, 800, 900, 1000, 1200, 1500, 2000, 3000, 6000
Quantification Using: Ladder Final Concentration (ng/uL): 0.0830 Dilution Factor: 12.0

 Copyright 2015 Advanced Analytical Technologies, Inc.

Sample: 103613-001-099
Well Location: C7
Created: Friday, June 21, 2019 4:25:48 PM

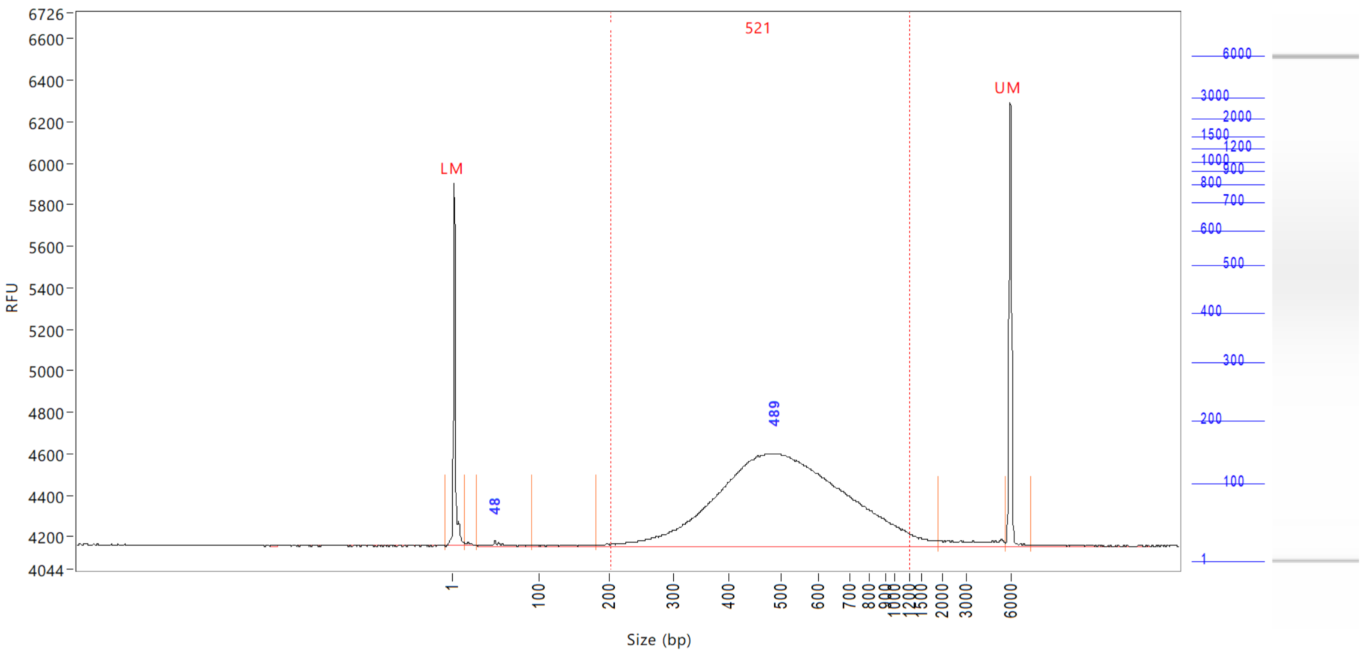

Peak	Size (bp)	Conc. (ng/uL)	From (bp)	To (bp)	RFU
1	1 (LM)	0.0103	0	14	1748
2	48	0.0067	28	91	25
3	489	1.0969	180	1889	447
4	6000 (UM)	0.0072	5695	7389	2142

TIC:	1.1035	ng/uL
TIM:	3.5813	nmole/L
Total Conc.:	1.1303	ng/uL

Smear Analysis	200 bp to 1200 bp	1.0808 ng/ul	95.6 %Total	3.4132 nmole/L	521 Avg. Size (b.p.)	30.19 %CV

```
Sample Peak Width (sec): 50   Sample Min Peak Height: 25    Sample Baseline V to V?: Y    Sample Baseline V to V pts: 3
Sample Filter: Binomial       # of Pts for Filter: 3        Sample Start Region (min): 0  Sample End Region (min): 50
Manual Baseline Start (min): 10     Manual Baseline End (min): 48
Marker Peak Width (sec): 5    Marker Min Peak Height: 200   Marker Baseline V to V?: Y    Marker Baseline V to V pts: 3
Lower Marker Selection: First Peak > 200 RFU                Upper Marker Selection: Last Peak > 200 RFU
Ladder Size (bp): 1, 100, 200, 300, 400, 500, 600, 700, 800, 900, 1000, 1200, 1500, 2000, 3000, 6000
Quantification Using: Ladder          Final Concentration (ng/uL): 0.0830          Dilution Factor: 12.0
```


Copyright 2015 Advanced Analytical Technologies, Inc.

Sample: 103613-001-100
Well Location: D7
Created: Friday, June 21, 2019 4:25:48 PM

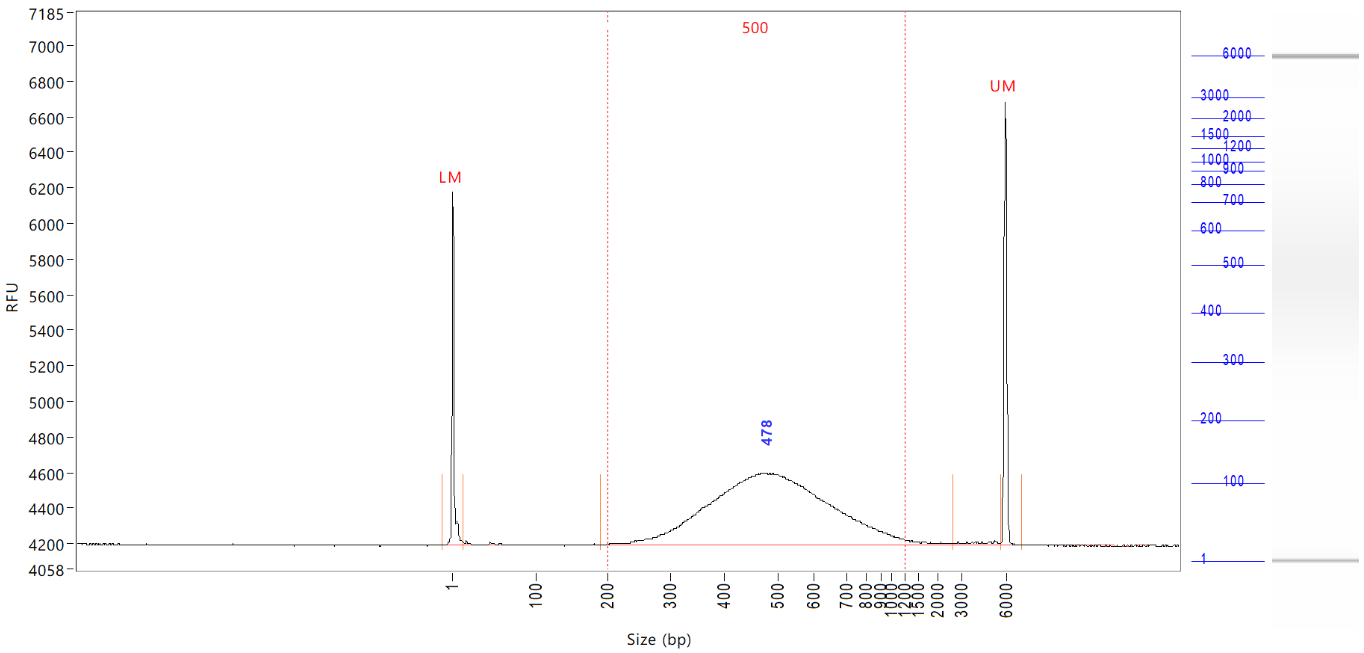

Peak	Size (bp)	Conc. (ng/uL)	From (bp)	To (bp)	RFU
1	1 (LM)	0.0103	0	15	1988
2	478	0.8526	190	2634	408
3	6000 (UM)	0.0074	5695	7061	2496

TIC:	0.8526	ng/uL
TIM:	2.7294	nmole/L
Total Conc.:	0.8696	ng/uL

Smear Analysis 200 bp to 1200 bp 0.8419 ng/ul 96.8 %Total 2.7685 nmole/L 500 Avg. Size (b.p.) 29.08 %CV

Sample Peak Width (sec): 50 Sample Min Peak Height: 25 Sample Baseline V to V?: Y Sample Baseline V to V pts: 3
Sample Filter: Binomial # of Pts for Filter: 3 Sample Start Region (min): 0 Sample End Region (min): 50
Manual Baseline Start (min): 10 Manual Baseline End (min): 48
Marker Peak Width (sec): 5 Marker Min Peak Height: 200 Marker Baseline V to V?: Y Marker Baseline V to V pts: 3
Lower Marker Selection: First Peak > 200 RFU Upper Marker Selection: Last Peak > 200 RFU
Ladder Size (bp): 1, 100, 200, 300, 400, 500, 600, 700, 800, 900, 1000, 1200, 1500, 2000, 3000, 6000
Quantification Using: Ladder Final Concentration (ng/uL): 0.0830 Dilution Factor: 12.0

Copyright 2015 Advanced Analytical Technologies, Inc.

Sample: 103613-001-101
Well Location: E7
Created: Friday, June 21, 2019 4:25:48 PM

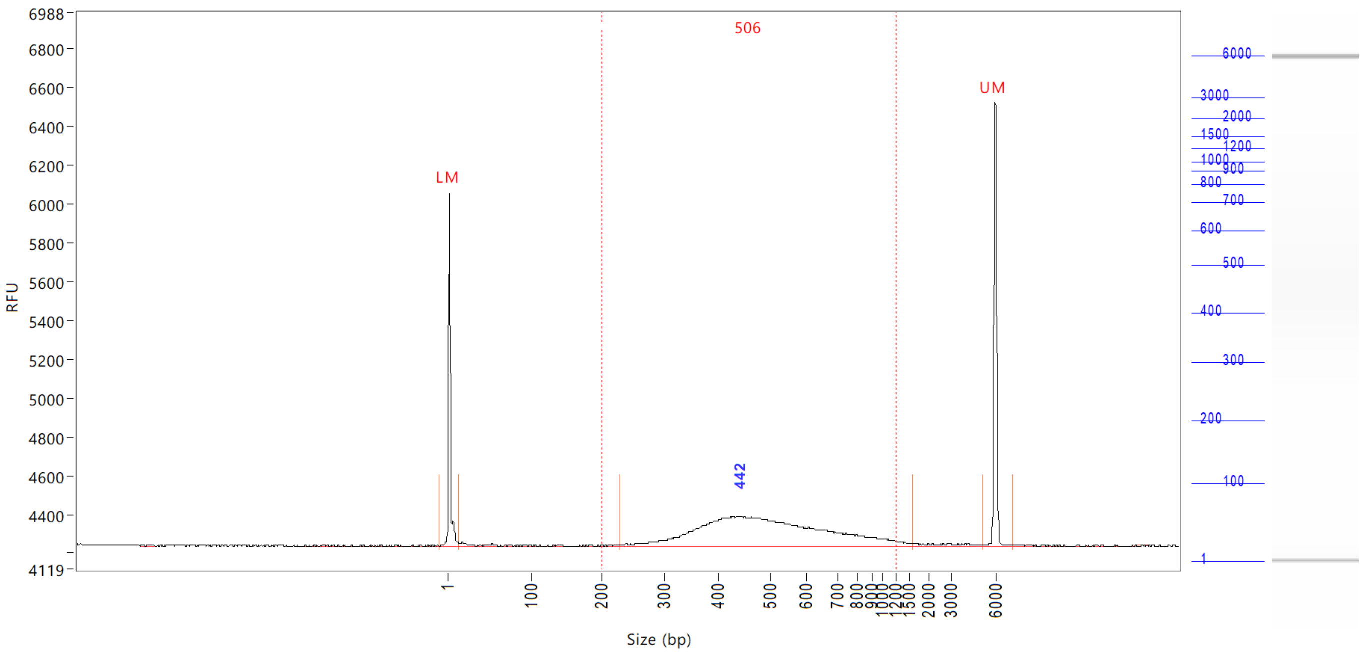

Peak	Size (bp)	Conc. (ng/uL)	From (bp)	To (bp)	RFU
1	1 (LM)	0.0103	0	15	1816
2	442	0.3537	230	1612	154
3	6000 (UM)	0.0074	5187	7162	2291
	TIC:	0.3537	ng/uL		
	TIM:	1.1259	nmole/L		
	Total Conc.:	0.3679	ng/uL		

Smear Analysis 200 bp to 1200 bp 0.3507 ng/ul 95.3 %Total 1.1397 nmole/L 506 Avg. Size (b.p.) 31.80 %CV

Sample Peak Width (sec): 50 Sample Min Peak Height: 25 Sample Baseline V to V?: Y Sample Baseline V to V pts: 3
Sample Filter: Binomial # of Pts for Filter: 3 Sample Start Region (min): 0 Sample End Region (min): 50
Manual Baseline Start (min): 10 Manual Baseline End (min): 48
Marker Peak Width (sec): 5 Marker Min Peak Height: 200 Marker Baseline V to V?: Y Marker Baseline V to V pts: 3
Lower Marker Selection: First Peak > 200 RFU Upper Marker Selection: Last Peak > 200 RFU
Ladder Size (bp): 1, 100, 200, 300, 400, 500, 600, 700, 800, 900, 1000, 1200, 1500, 2000, 3000, 6000
Quantification Using: Ladder Final Concentration (ng/uL): 0.0830 Dilution Factor: 12.0

 Copyright 2015 Advanced Analytical Technologies, Inc.

Sample: 103613-001-102
Well Location: F7
Created: Friday, June 21, 2019 4:25:48 PM

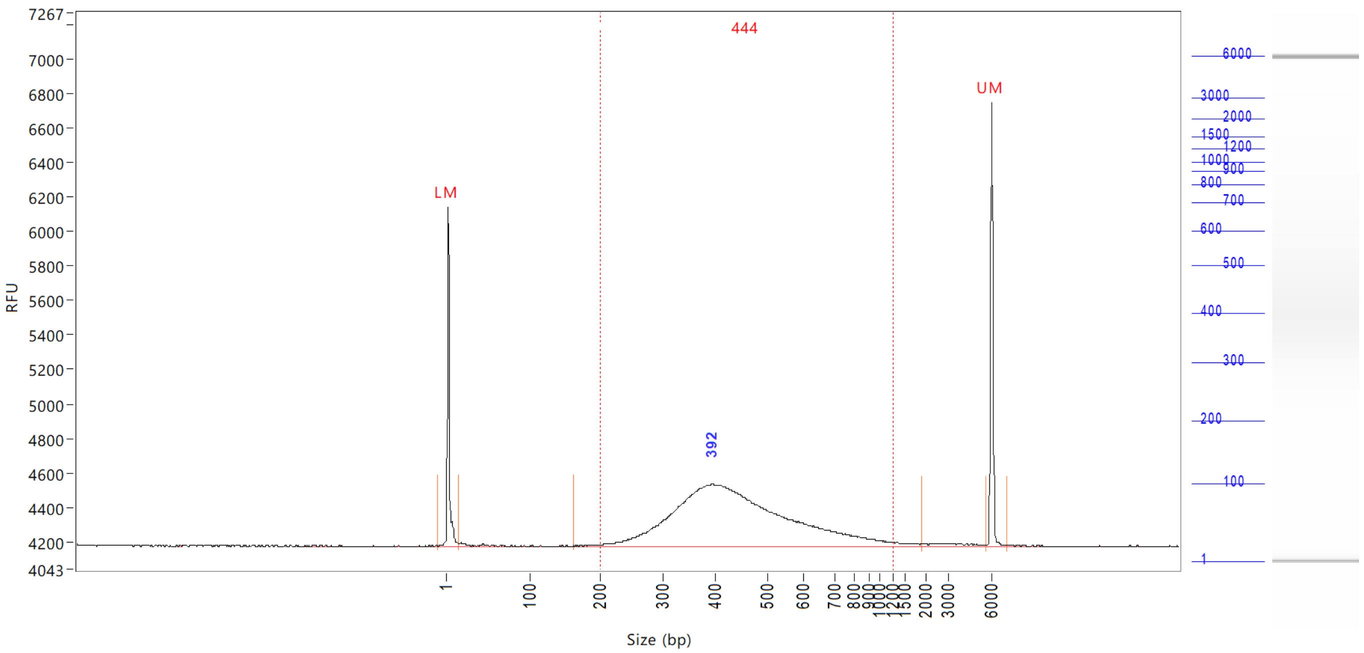

Peak	Size (bp)	Conc. (ng/uL)	From (bp)	To (bp)	RFU
1	1 (LM)	0.0103	0	14	1960
2	392	0.7038	161	1889	359
3	6000 (UM)	0.0077	5619	7010	2576
	TIC:	0.7038	ng/uL		
	TIM:	2.5678	nmole/L		
	Total Conc.:	0.7172	ng/uL		

Smear Analysis 200 bp to 1200 bp 0.6972 ng/ul 97.2 %Total 2.5838 nmole/L 444 Avg. Size (b.p.) 31.00 %CV

Sample Peak Width (sec): 50 Sample Min Peak Height: 25 Sample Baseline V to V?: Y Sample Baseline V to V pts: 3
Sample Filter: Binomial # of Pts for Filter: 3 Sample Start Region (min): 0 Sample End Region (min): 50
Manual Baseline Start (min): 10 Manual Baseline End (min): 48
Marker Peak Width (sec): 5 Marker Min Peak Height: 200 Marker Baseline V to V?: Y Marker Baseline V to V pts: 3
Lower Marker Selection: First Peak > 200 RFU Upper Marker Selection: Last Peak > 200 RFU
Ladder Size (bp): 1, 100, 200, 300, 400, 500, 600, 700, 800, 900, 1000, 1200, 1500, 2000, 3000, 6000
Quantification Using: Ladder Final Concentration (ng/uL): 0.0830 Dilution Factor: 12.0

Copyright 2015 Advanced Analytical Technologies, Inc.

Sample: 103613-001-103
Well Location: G7
Created: Friday, June 21, 2019 4:25:48 PM

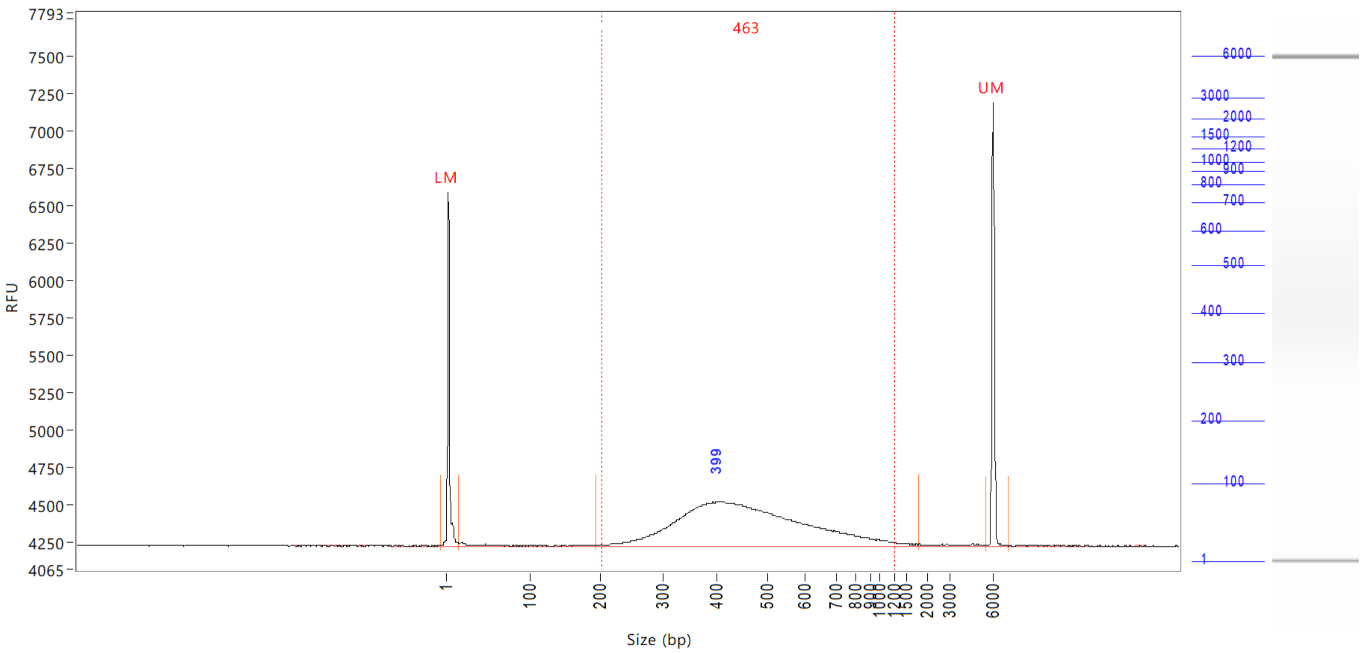

Peak	Size (bp)	Conc. (ng/uL)	From (bp)	To (bp)	RFU
1	1 (LM)	0.0103	0	15	2372
2	399	0.5467	193	1806	299
3	6000 (UM)	0.0074	5543	7061	2978

TIC:	0.5467	ng/uL
TIM:	1.9140	nmole/L
Total Conc.:	0.5615	ng/uL

Smear Analysis 200 bp to 1200 bp 0.5421 ng/ul 96.6 %Total 1.9257 nmole/L 463 Avg. Size (b.p.) 31.67 %CV

Sample Peak Width (sec): 50 Sample Min Peak Height: 25 Sample Baseline V to V?: Y Sample Baseline V to V pts: 3
Sample Filter: Binomial # of Pts for Filter: 3 Sample Start Region (min): 0 Sample End Region (min): 50
Manual Baseline Start (min): 10 Manual Baseline End (min): 48
Marker Peak Width (sec): 5 Marker Min Peak Height: 200 Marker Baseline V to V?: Y Marker Baseline V to V pts: 3
Lower Marker Selection: First Peak > 200 RFU Upper Marker Selection: Last Peak > 200 RFU
Ladder Size (bp): 1, 100, 200, 300, 400, 500, 600, 700, 800, 900, 1000, 1200, 1500, 2000, 3000, 6000
Quantification Using: Ladder Final Concentration (ng/uL): 0.0830 Dilution Factor: 12.0

 Copyright 2015 Advanced Analytical Technologies, Inc.

Sample: 103613-001-104
Well Location: H7
Created: Friday, June 21, 2019 4:25:48 PM

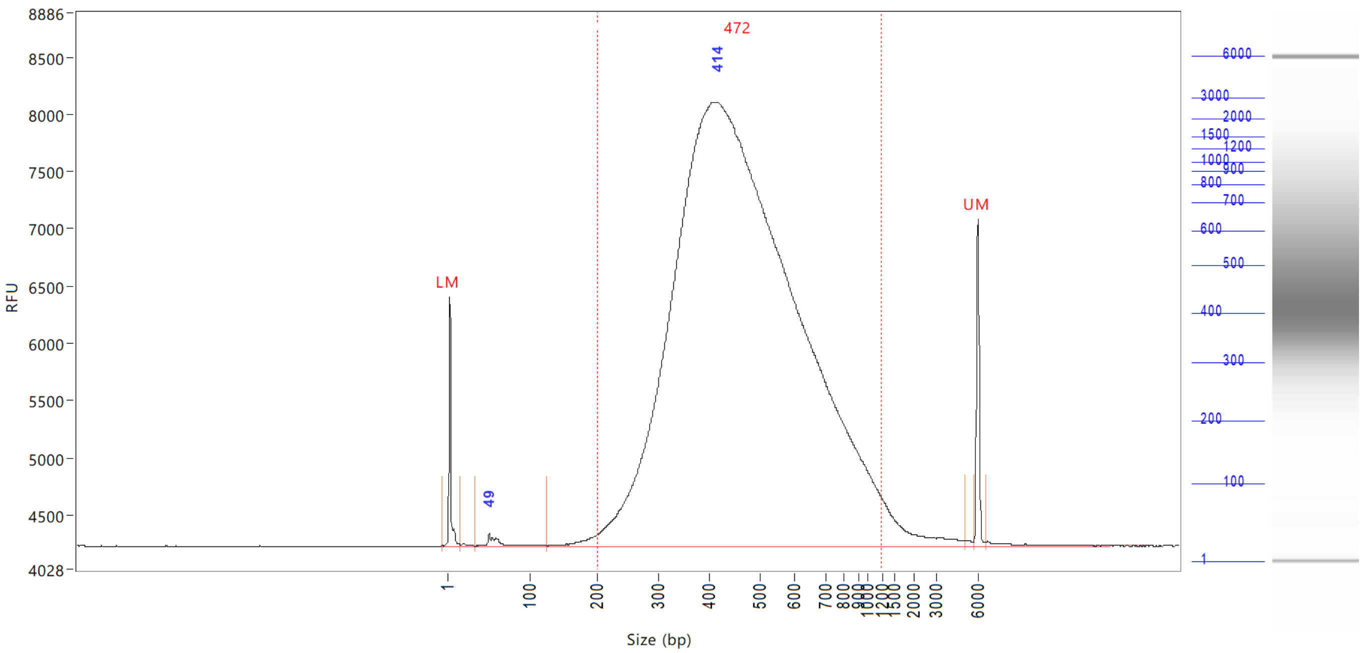

Peak	Size (bp)	Conc. (ng/uL)	From (bp)	To (bp)	RFU
1	1 (LM)	0.0103	0	15	2180
2	49	0.0343	33	125	114
3	414	7.9371	125	5085	3882
4	6000 (UM)	0.0079	5695	6556	2853

TIC:	7.9714	ng/uL
TIM:	27.3027	nmole/L
Total Conc.:	7.9796	ng/uL

Smear Analysis 200 bp to 1200 bp 7.7867 ng/ul 97.6 %Total 27.1562 nmole/L 472 Avg. Size (b.p.) 32.40 %CV

Sample Peak Width (sec): 50 Sample Min Peak Height: 25 Sample Baseline V to V?: Y Sample Baseline V to V pts: 3
Sample Filter: Binomial # of Pts for Filter: 3 Sample Start Region (min): 0 Sample End Region (min): 50
Manual Baseline Start (min): 10 Manual Baseline End (min): 48
Marker Peak Width (sec): 5 Marker Min Peak Height: 200 Marker Baseline V to V?: Y Marker Baseline V to V pts: 3
Lower Marker Selection: First Peak > 200 RFU Upper Marker Selection: Last Peak > 200 RFU
Ladder Size (bp): 1, 100, 200, 300, 400, 500, 600, 700, 800, 900, 1000, 1200, 1500, 2000, 3000, 6000
Quantification Using: Ladder Final Concentration (ng/uL): 0.0830 Dilution Factor: 12.0

 Copyright 2015 Advanced Analytical Technologies, Inc.

Sample: 103613-001-105
Well Location: A8
Created: Friday, June 21, 2019 4:25:48 PM

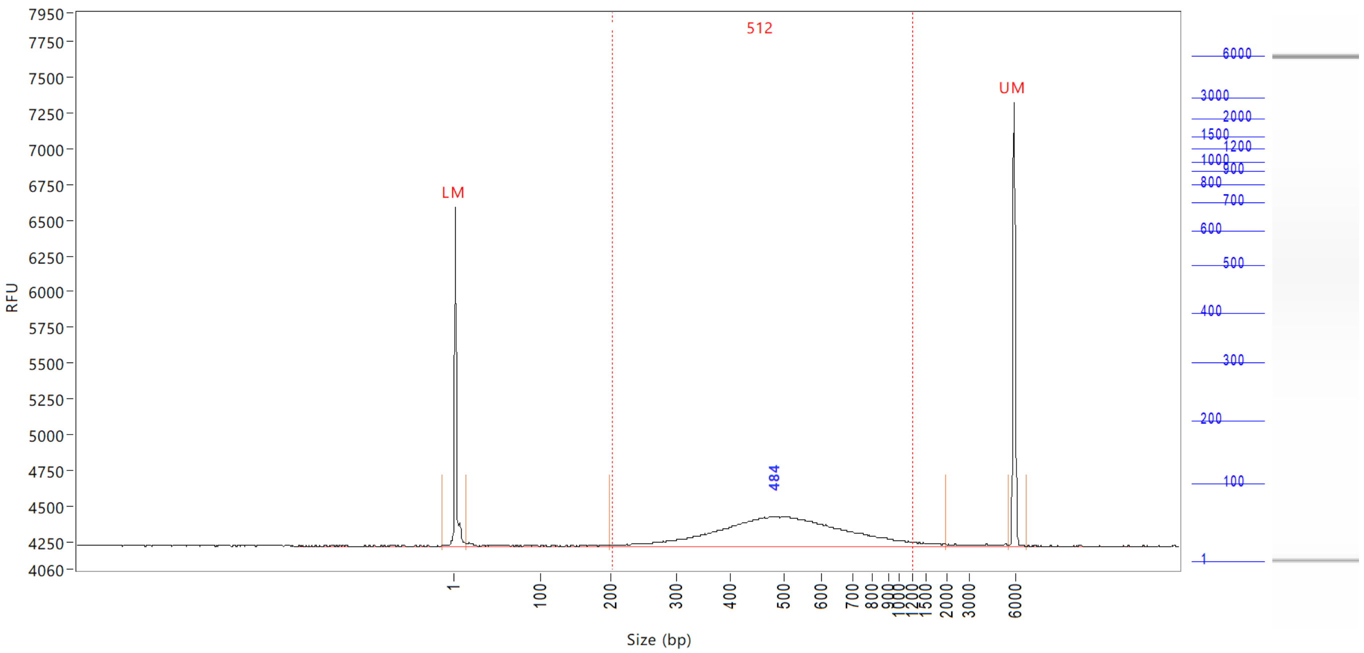

Peak	Size (bp)	Conc. (ng/uL)	From (bp)	To (bp)	RFU
1	1 (LM)	0.0103	0	15	2365
2	484	0.3623	198	1972	206
3	6000 (UM)	0.0073	5593	6783	3107

TIC:	0.3623	ng/uL
TIM:	1.1291	nmole/L
Total Conc.:	0.3763	ng/uL

Smear Analysis	200 bp to 1200 bp	0.3563 ng/ul	94.7 %Total	1.1458 nmole/L	512 Avg. Size (b.p.)	30.60 %CV

Sample Peak Width (sec): 50 Sample Min Peak Height: 25 Sample Baseline V to V?: Y Sample Baseline V to V pts: 3
Sample Filter: Binomial # of Pts for Filter: 3 Sample Start Region (min): 0 Sample End Region (min): 50
Manual Baseline Start (min): 10 Manual Baseline End (min): 48
Marker Peak Width (sec): 5 Marker Min Peak Height: 200 Marker Baseline V to V?: Y Marker Baseline V to V pts: 3
Lower Marker Selection: First Peak > 200 RFU Upper Marker Selection: Last Peak > 200 RFU
Ladder Size (bp): 1, 100, 200, 300, 400, 500, 600, 700, 800, 900, 1000, 1200, 1500, 2000, 3000, 6000
Quantification Using: Ladder Final Concentration (ng/uL): 0.0830 Dilution Factor: 12.0

 Copyright 2015 Advanced Analytical Technologies, Inc.

Sample: 103613-001-106
Well Location: B8
Created: Friday, June 21, 2019 4:25:48 PM

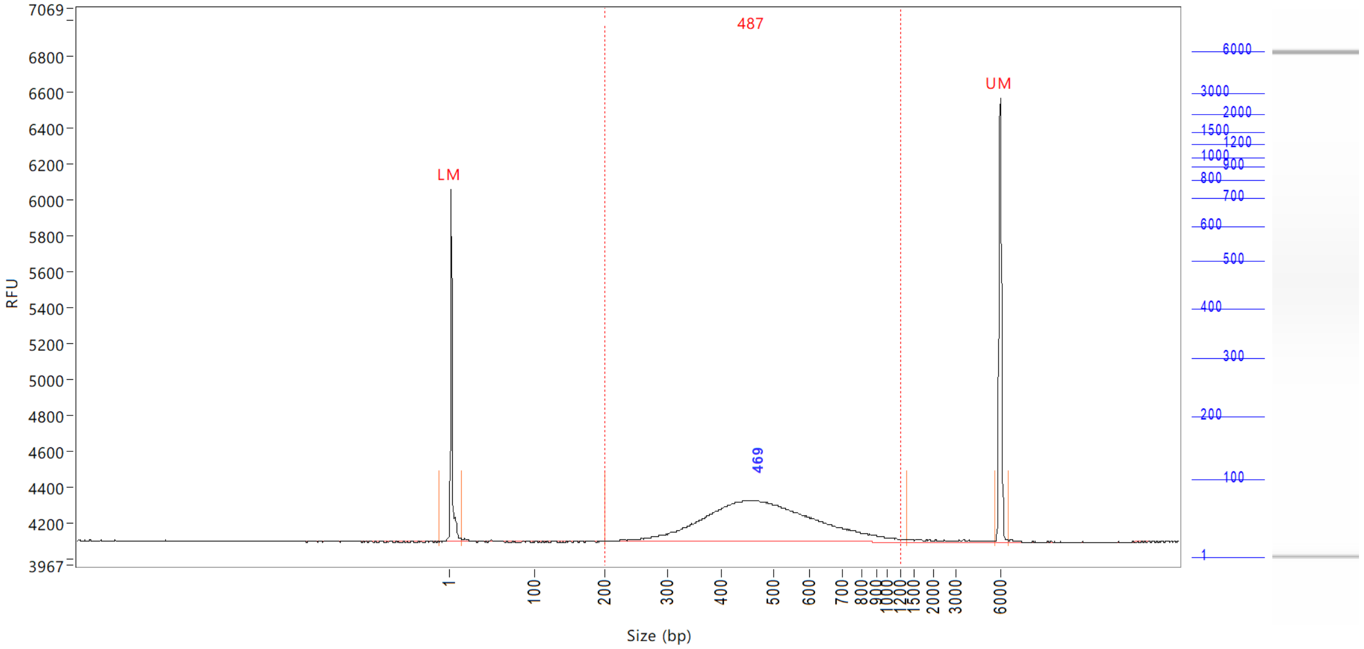

Peak	Size (bp)	Conc. (ng/uL)	From (bp)	To (bp)	RFU
1	1 (LM)	0.0103	0	15	1960
2	469	0.4609	199	1346	232
3	6000 (UM)	0.0075	5670	6581	2477

TIC:	0.4609	ng/uL
TIM:	1.5509	nmole/L
Total Conc.:	0.4726	ng/uL

Smear Analysis	200 bp to 1200 bp	0.4598 ng/ul	97.3 %Total	1.5535 nmole/L	487 Avg. Size (b.p.)	28.21 %CV

Sample Peak Width (sec): 50 Sample Min Peak Height: 25 Sample Baseline V to V?: Y Sample Baseline V to V pts: 3
Sample Filter: Binomial # of Pts for Filter: 3 Sample Start Region (min): 0 Sample End Region (min): 50
Manual Baseline Start (min): 10 Manual Baseline End (min): 48
Marker Peak Width (sec): 5 Marker Min Peak Height: 200 Marker Baseline V to V?: Y Marker Baseline V to V pts: 3
Lower Marker Selection: First Peak > 200 RFU Upper Marker Selection: Last Peak > 200 RFU
Ladder Size (bp): 1, 100, 200, 300, 400, 500, 600, 700, 800, 900, 1000, 1200, 1500, 2000, 3000, 6000
Quantification Using: Ladder Final Concentration (ng/uL): 0.0830 Dilution Factor: 12.0

 Copyright 2015 Advanced Analytical Technologies, Inc.

Sample: 103613-001-107
Well Location: C8
Created: Friday, June 21, 2019 4:25:48 PM

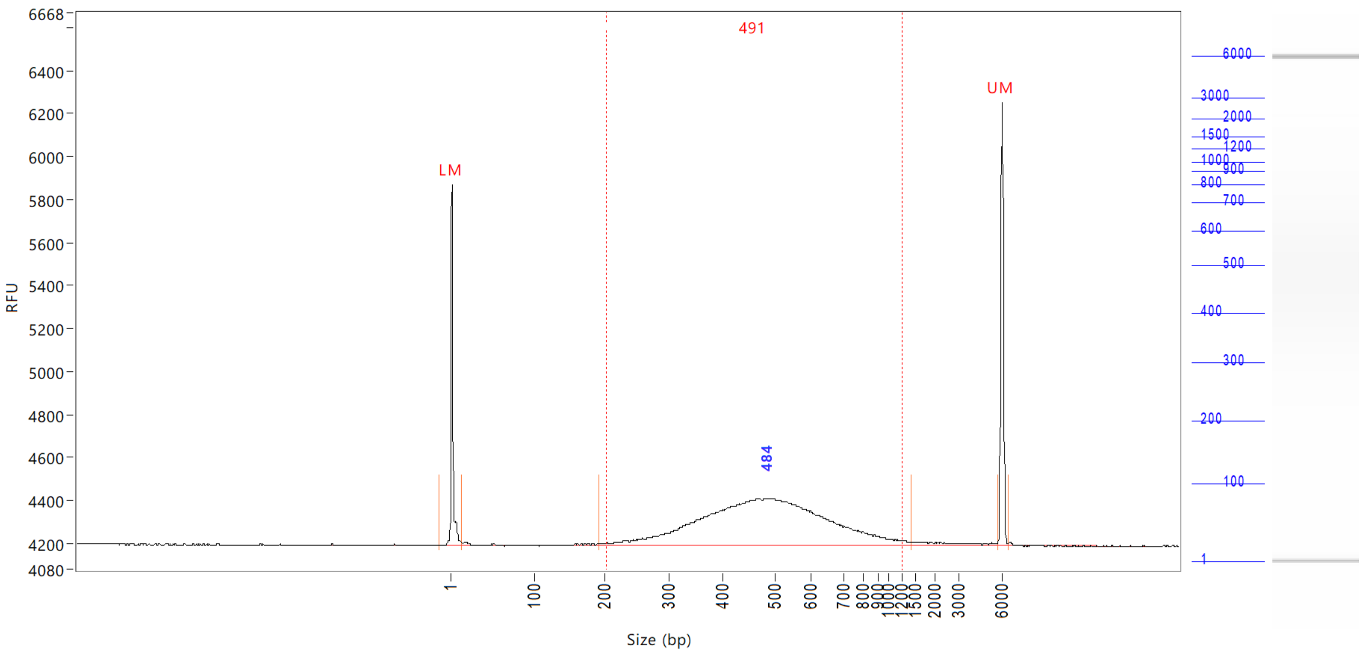

Peak	Size (bp)	Conc. (ng/uL)	From (bp)	To (bp)	RFU
1	1 (LM)	0.0103	0	14	1674
2	484	0.5788	191	1428	219
3	6000 (UM)	0.0073	5695	6404	2066

TIC:	0.5788	ng/uL
TIM:	1.9281	nmole/L
Total Conc.:	0.5984	ng/uL

Smear Analysis 200 bp to 1200 bp 0.5750 ng/ul 96.1 %Total 1.9288 nmole/L 491 Avg. Size (b.p.) 30.60 %CV

Sample Peak Width (sec): 50 Sample Min Peak Height: 25 Sample Baseline V to V?: Y Sample Baseline V to V pts: 3
Sample Filter: Binomial # of Pts for Filter: 3 Sample Start Region (min): 0 Sample End Region (min): 50
Manual Baseline Start (min): 10 Manual Baseline End (min): 48
Marker Peak Width (sec): 5 Marker Min Peak Height: 200 Marker Baseline V to V?: Y Marker Baseline V to V pts: 3
Lower Marker Selection: First Peak > 200 RFU Upper Marker Selection: Last Peak > 200 RFU
Ladder Size (bp): 1, 100, 200, 300, 400, 500, 600, 700, 800, 900, 1000, 1200, 1500, 2000, 3000, 6000
Quantification Using: Ladder Final Concentration (ng/uL): 0.0830 Dilution Factor: 12.0

 Copyright 2015 Advanced Analytical Technologies, Inc.

Sample: 103613-001-108
Well Location: D8
Created: Friday, June 21, 2019 4:25:48 PM

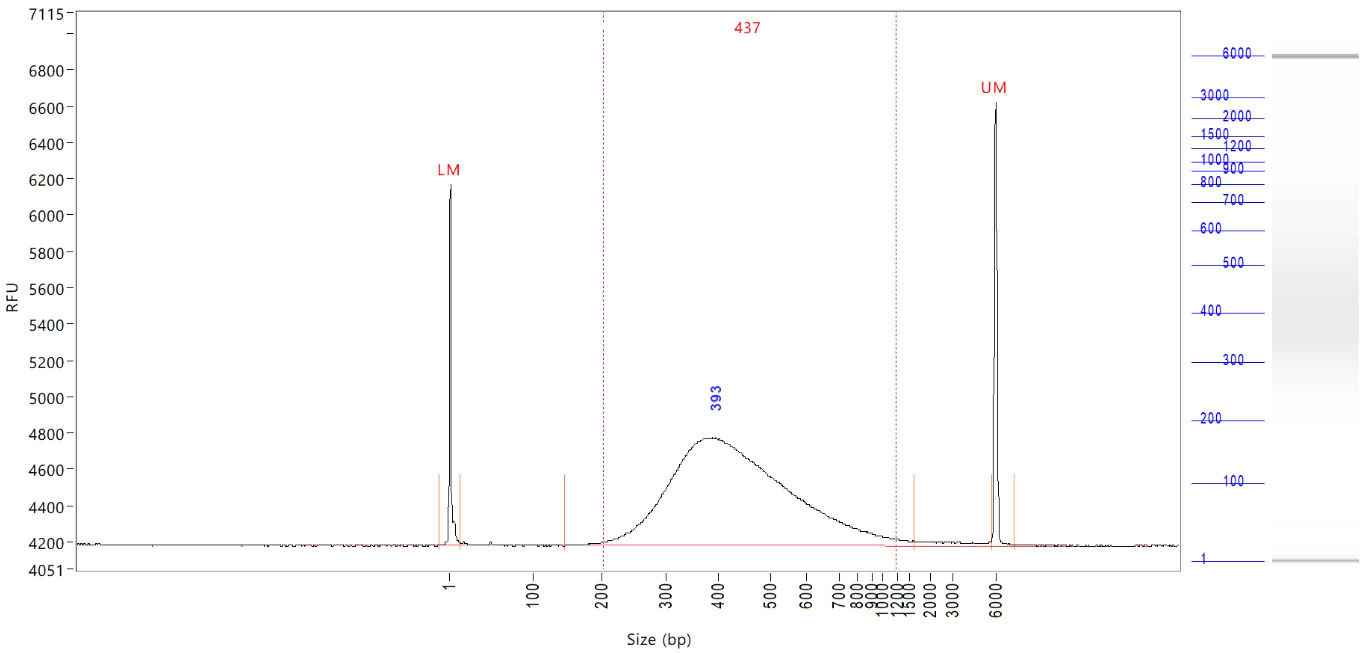

Peak	Size (bp)	Conc. (ng/uL)	From (bp)	To (bp)	RFU
1	1 (LM)	0.0103	0	14	1987
2	393	1.2724	145	1630	593
3	6000 (UM)	0.0074	5720	7288	2447

TIC:	1.2724	ng/uL
TIM:	4.7475	nmole/L
Total Conc.:	1.2929	ng/uL

Smear Analysis	200 bp to 1200 bp	1.2621 ng/ul	97.6 %Total	4.7532 nmole/L	437 Avg. Size (b.p.)	31.09 %CV

Sample Peak Width (sec): 50 Sample Min Peak Height: 25 Sample Baseline V to V?: Y Sample Baseline V to V pts: 3
Sample Filter: Binomial # of Pts for Filter: 3 Sample Start Region (min): 0 Sample End Region (min): 50
Manual Baseline Start (min): 10 Manual Baseline End (min): 48
Marker Peak Width (sec): 5 Marker Min Peak Height: 200 Marker Baseline V to V?: Y Marker Baseline V to V pts: 3
Lower Marker Selection: First Peak > 200 RFU Upper Marker Selection: Last Peak > 200 RFU
Ladder Size (bp): 1, 100, 200, 300, 400, 500, 600, 700, 800, 900, 1000, 1200, 1500, 2000, 3000, 6000
Quantification Using: Ladder Final Concentration (ng/uL): 0.0830 Dilution Factor: 12.0

Copyright 2015 Advanced Analytical Technologies, Inc.

Sample: 103613-001-109
Well Location: E8
Created: Friday, June 21, 2019 4:25:48 PM

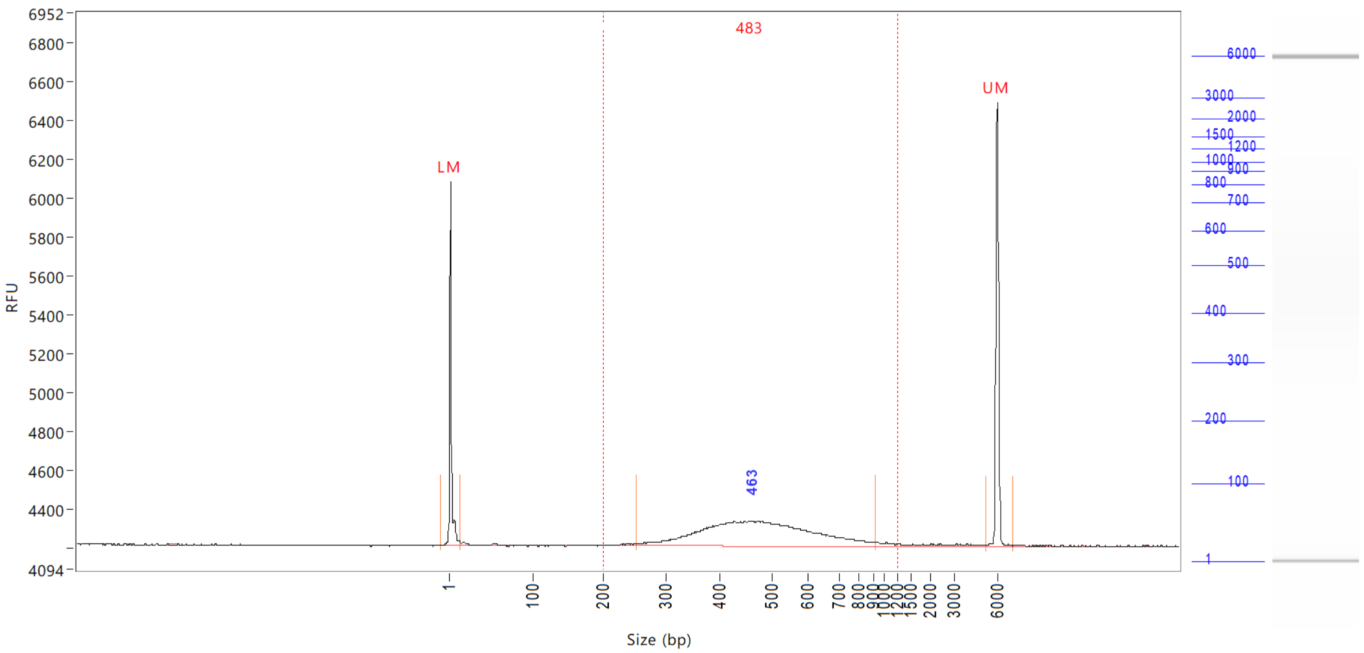

Peak	Size (bp)	Conc. (ng/uL)	From (bp)	To (bp)	RFU
1	1 (LM)	0.0103	0	15	1868
2	463	0.2710	251	920	127
3	6000 (UM)	0.0074	5187	7061	2283

TIC:	0.2710	ng/uL
TIM:	0.9310	nmole/L
Total Conc.:	0.2960	ng/uL

Smear Analysis 200 bp to 1200 bp 0.2791 ng/ul 94.3 %Total 0.9508 nmole/L 483 Avg. Size (b.p.) 29.09 %CV

Sample Peak Width (sec): 50 Sample Min Peak Height: 25 Sample Baseline V to V?: Y Sample Baseline V to V pts: 3
Sample Filter: Binomial # of Pts for Filter: 3 Sample Start Region (min): 0 Sample End Region (min): 50
Manual Baseline Start (min): 10 Manual Baseline End (min): 48
Marker Peak Width (sec): 5 Marker Min Peak Height: 200 Marker Baseline V to V?: Y Marker Baseline V to V pts: 3
Lower Marker Selection: First Peak > 200 RFU Upper Marker Selection: Last Peak > 200 RFU
Ladder Size (bp): 1, 100, 200, 300, 400, 500, 600, 700, 800, 900, 1000, 1200, 1500, 2000, 3000, 6000
Quantification Using: Ladder Final Concentration (ng/uL): 0.0830 Dilution Factor: 12.0

 Copyright 2015 Advanced Analytical Technologies, Inc.

Sample: 103613-001-110
Well Location: F8
Created: Friday, June 21, 2019 4:25:48 PM

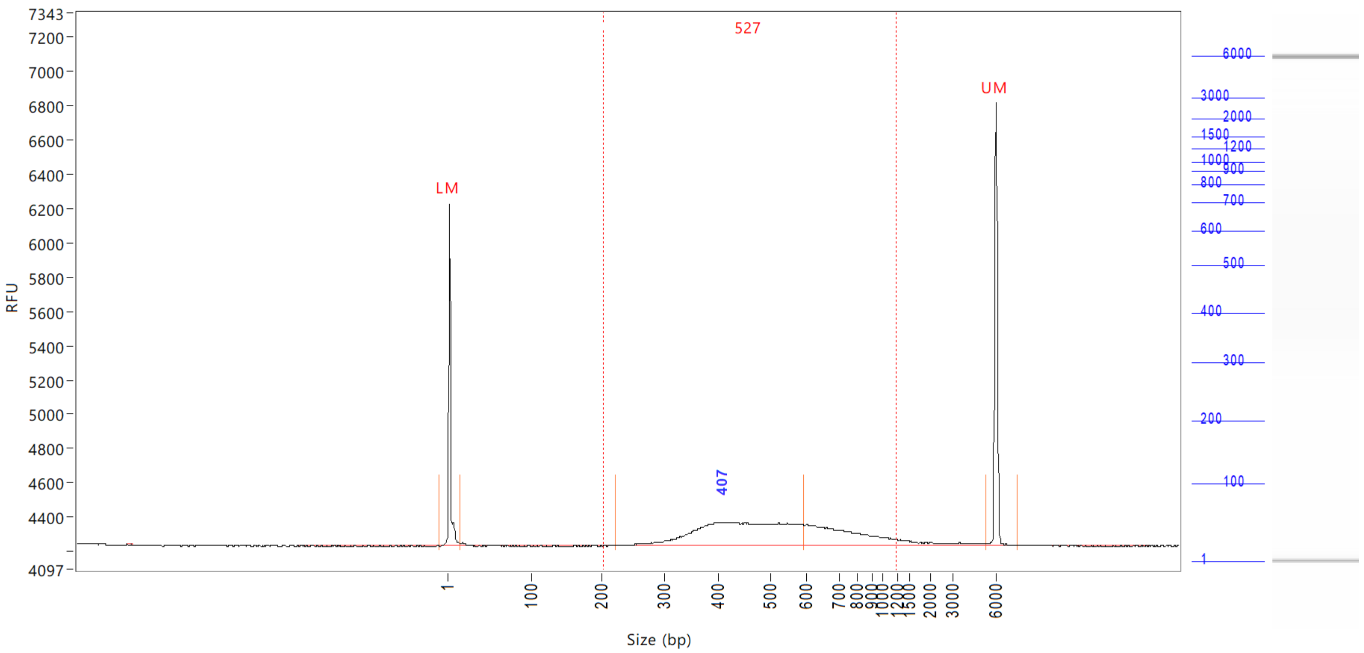

Peak	Size (bp)	Conc. (ng/uL)	From (bp)	To (bp)	RFU
1	1 (LM)	0.0103	0	15	1992
2	407	0.2479	222	593	137
3	6000 (UM)	0.0079	5339	7465	2591

TIC:	0.2479	ng/uL
TIM:	0.9248	nmole/L
Total Conc.:	0.3688	ng/uL

Smear Analysis 200 bp to 1200 bp 0.3466 ng/ul 94.0 %Total 1.0828 nmole/L 527 Avg. Size (b.p.) 32.45 %CV

Sample Peak Width (sec): 50 Sample Min Peak Height: 25 Sample Baseline V to V?: Y Sample Baseline V to V pts: 3
Sample Filter: Binomial # of Pts for Filter: 3 Sample Start Region (min): 0 Sample End Region (min): 50
Manual Baseline Start (min): 10 Manual Baseline End (min): 48
Marker Peak Width (sec): 5 Marker Min Peak Height: 200 Marker Baseline V to V?: Y Marker Baseline V to V pts: 3
Lower Marker Selection: First Peak > 200 RFU Upper Marker Selection: Last Peak > 200 RFU
Ladder Size (bp): 1, 100, 200, 300, 400, 500, 600, 700, 800, 900, 1000, 1200, 1500, 2000, 3000, 6000
Quantification Using: Ladder Final Concentration (ng/uL): 0.0830 Dilution Factor: 12.0

 Copyright 2015 Advanced Analytical Technologies, Inc.

Sample: 103613-001-111
Well Location: G8
Created: Friday, June 21, 2019 4:25:48 PM

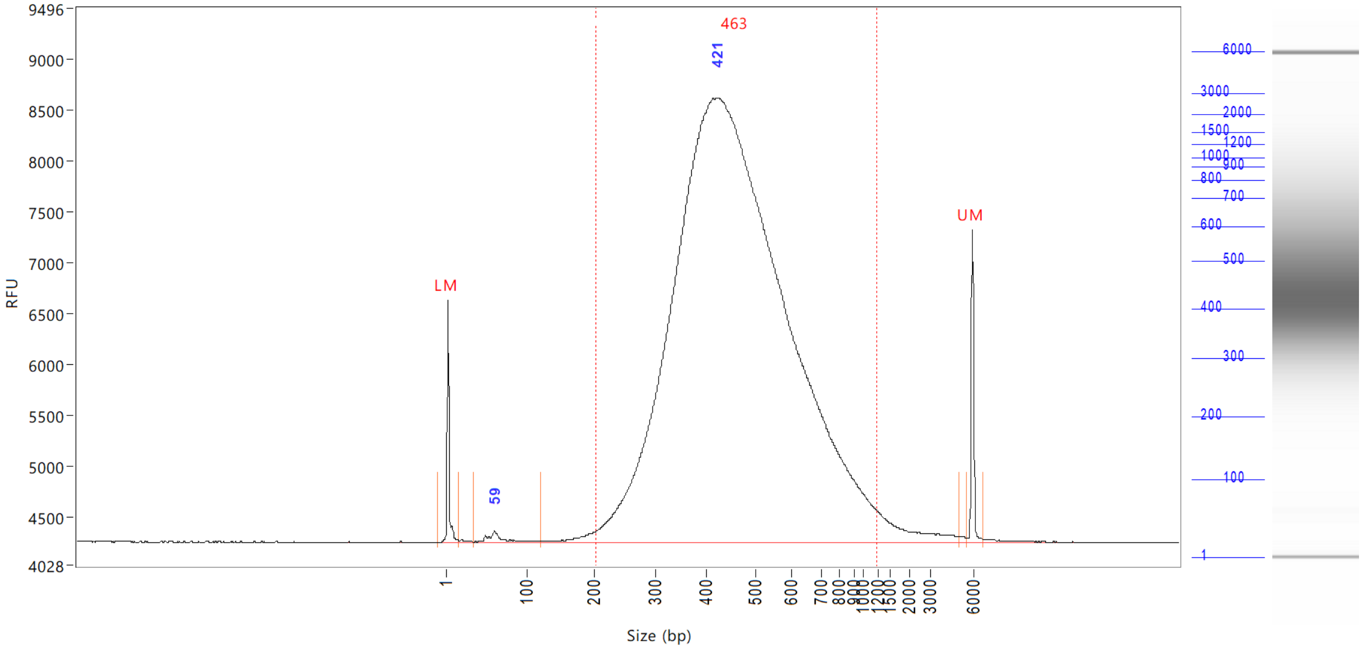

Peak	Size (bp)	Conc. (ng/uL)	From (bp)	To (bp)	RFU
1	1 (LM)	0.0103	0	16	2377
2	59	0.0339	33	120	107
3	421	7.5651	120	5085	4370
4	6000 (UM)	0.0079	5543	6758	3064
	TIC:	7.5990	ng/uL		
	TIM:	26.5339	nmole/L		
	Total Conc.:	7.6053	ng/uL		

Smear Analysis 200 bp to 1200 bp 7.4332 ng/ul 97.7 %Total 26.4241 nmole/L 463 Avg. Size (b.p.) 30.25 %CV

Sample Peak Width (sec): 50 Sample Min Peak Height: 25 Sample Baseline V to V?: Y Sample Baseline V to V pts: 3
Sample Filter: Binomial # of Pts for Filter: 3 Sample Start Region (min): 0 Sample End Region (min): 50
Manual Baseline Start (min): 10 Manual Baseline End (min): 48
Marker Peak Width (sec): 5 Marker Min Peak Height: 200 Marker Baseline V to V?: Y Marker Baseline V to V pts: 3
Lower Marker Selection: First Peak > 200 RFU Upper Marker Selection: Last Peak > 200 RFU
Ladder Size (bp): 1, 100, 200, 300, 400, 500, 600, 700, 800, 900, 1000, 1200, 1500, 2000, 3000, 6000
Quantification Using: Ladder Final Concentration (ng/uL): 0.0830 Dilution Factor: 12.0

Copyright 2015 Advanced Analytical Technologies, Inc.

Sample: 103613-001-112
Well Location: H8
Created: Friday, June 21, 2019 4:25:48 PM

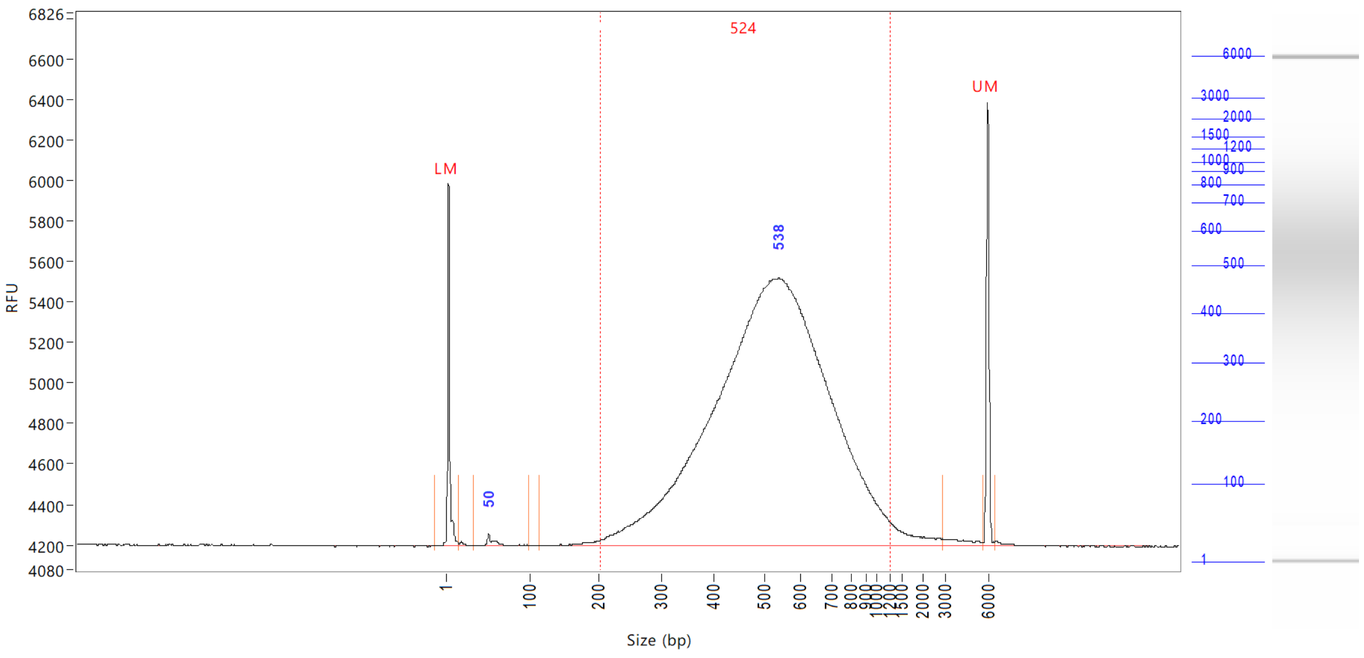

Peak	Size (bp)	Conc. (ng/uL)	From (bp)	To (bp)	RFU
1	1 (LM)	0.0103	0	15	1787
2	50	0.0129	32	97	57
3	538	2.8673	114	2900	1323
4	6000 (UM)	0.0069	5670	6480	2191

TIC:	2.8802	ng/uL
TIM:	9.1238	nmole/L
Total Conc.:	2.8969	ng/uL

Smear Analysis 200 bp to 1200 bp 2.8186 ng/ul 97.3 %Total 8.8479 nmole/L 524 Avg. Size (b.p.) 29.04 %CV

Sample Peak Width (sec): 50 Sample Min Peak Height: 25 Sample Baseline V to V?: Y Sample Baseline V to V pts: 3
Sample Filter: Binomial # of Pts for Filter: 3 Sample Start Region (min): 0 Sample End Region (min): 50
Manual Baseline Start (min): 10 Manual Baseline End (min): 48
Marker Peak Width (sec): 5 Marker Min Peak Height: 200 Marker Baseline V to V?: Y Marker Baseline V to V pts: 3
Lower Marker Selection: First Peak > 200 RFU Upper Marker Selection: Last Peak > 200 RFU
Ladder Size (bp): 1, 100, 200, 300, 400, 500, 600, 700, 800, 900, 1000, 1200, 1500, 2000, 3000, 6000
Quantification Using: Ladder Final Concentration (ng/uL): 0.0830 Dilution Factor: 12.0

Copyright 2015 Advanced Analytical Technologies, Inc.

Sample: 103613-001-113
Well Location: A9
Created: Friday, June 21, 2019 4:25:48 PM

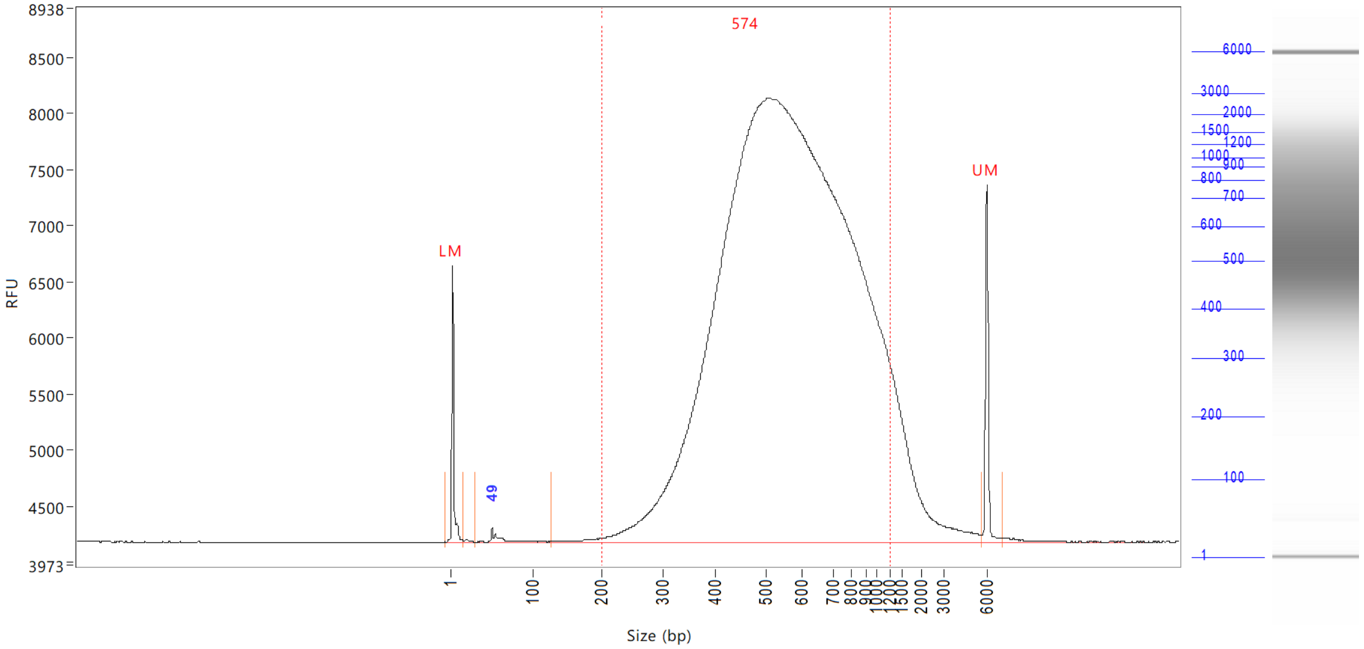

Peak	Size (bp)	Conc. (ng/uL)	From (bp)	To (bp)	RFU
1	1 (LM)	0.0103	0	14	2472
2	49	0.0234	30	125	134
3	6000 (UM)	0.0080	5695	7162	3188
	TIC:	0.0234	ng/uL		
	TIM:	0.5998	nmole/L		
	Total Conc.:	7.6600	ng/uL		

Smear Analysis 200 bp to 1200 bp 7.2382 ng/ul 94.5 %Total 20.7624 nmole/L 574 Avg. Size (b.p.) 31.91 %CV

Sample Peak Width (sec): 50 Sample Min Peak Height: 25 Sample Baseline V to V?: Y Sample Baseline V to V pts: 3
Sample Filter: Binomial # of Pts for Filter: 3 Sample Start Region (min): 0 Sample End Region (min): 50
Manual Baseline Start (min): 10 Manual Baseline End (min): 48
Marker Peak Width (sec): 5 Marker Min Peak Height: 200 Marker Baseline V to V?: Y Marker Baseline V to V pts: 3
Lower Marker Selection: First Peak > 200 RFU Upper Marker Selection: Last Peak > 200 RFU
Ladder Size (bp): 1, 100, 200, 300, 400, 500, 600, 700, 800, 900, 1000, 1200, 1500, 2000, 3000, 6000
Quantification Using: Ladder Final Concentration (ng/uL): 0.0830 Dilution Factor: 12.0

 Copyright 2015 Advanced Analytical Technologies, Inc.

Sample: 103613-001-114
Well Location: B9
Created: Friday, June 21, 2019 4:25:48 PM

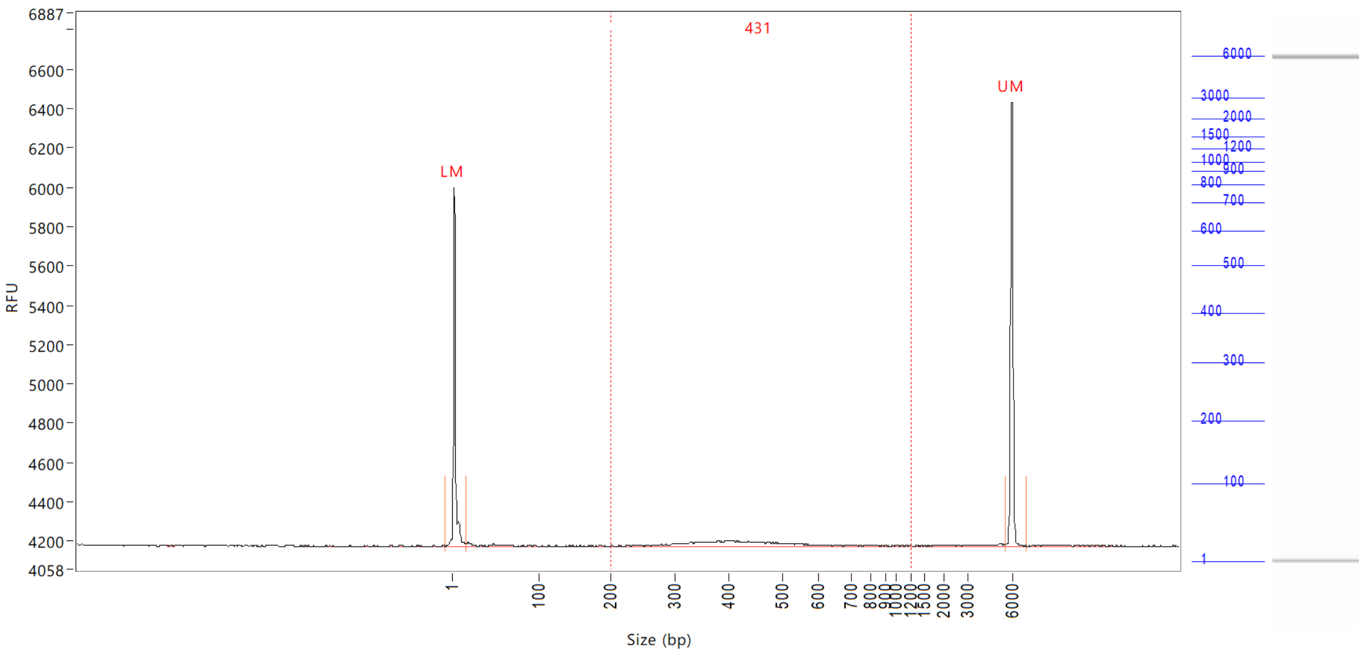

Peak	Size (bp)	Conc. (ng/uL)	From (bp)	To (bp)	RFU
1	1 (LM)	0.0103	0	15	1821
2	6000 (UM)	0.0073	5543	6985	2261

TIC:	0.0000	ng/uL
TIM:	0.0000	nmole/L
Total Conc.:	0.0698	ng/uL

Smear Analysis	200 bp to 1200 bp	0.0545 ng/ul	78.2 %Total	0.2080 nmole/L	431 Avg. Size (b.p.)	31.39 %CV

Sample Peak Width (sec): 50 Sample Min Peak Height: 25 Sample Baseline V to V?: Y Sample Baseline V to V pts: 3
Sample Filter: Binomial # of Pts for Filter: 3 Sample Start Region (min): 0 Sample End Region (min): 50
Manual Baseline Start (min): 10 Manual Baseline End (min): 48
Marker Peak Width (sec): 5 Marker Min Peak Height: 200 Marker Baseline V to V?: Y Marker Baseline V to V pts: 3
Lower Marker Selection: First Peak > 200 RFU Upper Marker Selection: Last Peak > 200 RFU
Ladder Size (bp): 1, 100, 200, 300, 400, 500, 600, 700, 800, 900, 1000, 1200, 1500, 2000, 3000, 6000
Quantification Using: Ladder Final Concentration (ng/uL): 0.0830 Dilution Factor: 12.0

 Copyright 2015 Advanced Analytical Technologies, Inc.

Sample: 103613-001-115
Well Location: C9
Created: Friday, June 21, 2019 4:25:48 PM

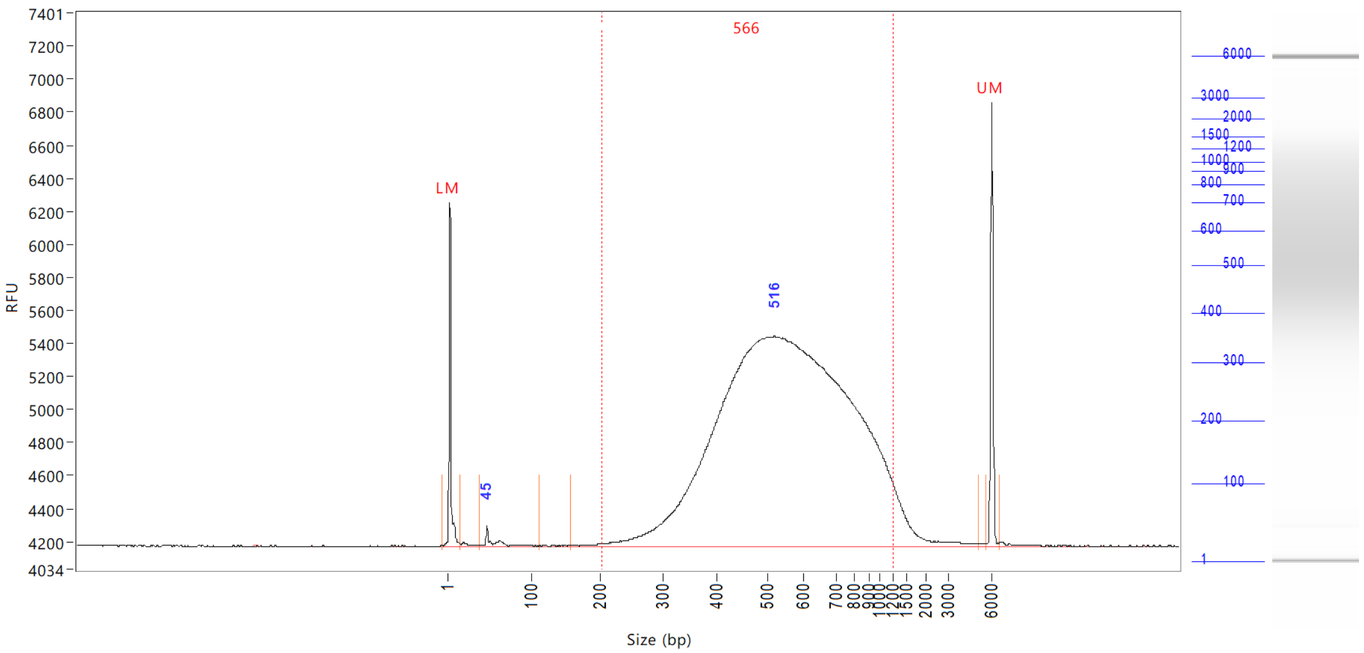

Peak	Size (bp)	Conc. (ng/uL)	From (bp)	To (bp)	RFU
1	1 (LM)	0.0103	0	15	2083
2	45	0.0166	38	110	124
3	516	2.8638	156	5085	1272
4	6000 (UM)	0.0076	5644	6505	2689

TIC:	2.8803	ng/uL
TIM:	8.3968	nmole/L
Total Conc.:	2.8860	ng/uL

Smear Analysis	200 bp to 1200 bp	2.7880 ng/ul	96.6 %Total	8.1035 nmole/L	566 Avg. Size (b.p.)	31.35 %CV

Sample Peak Width (sec): 50 Sample Min Peak Height: 25 Sample Baseline V to V?: Y Sample Baseline V to V pts: 3
Sample Filter: Binomial # of Pts for Filter: 3 Sample Start Region (min): 0 Sample End Region (min): 50
Manual Baseline Start (min): 10 Manual Baseline End (min): 48
Marker Peak Width (sec): 5 Marker Min Peak Height: 200 Marker Baseline V to V?: Y Marker Baseline V to V pts: 3
Lower Marker Selection: First Peak > 200 RFU Upper Marker Selection: Last Peak > 200 RFU
Ladder Size (bp): 1, 100, 200, 300, 400, 500, 600, 700, 800, 900, 1000, 1200, 1500, 2000, 3000, 6000
Quantification Using: Ladder Final Concentration (ng/uL): 0.0830 Dilution Factor: 12.0

Copyright 2015 Advanced Analytical Technologies, Inc.

Sample: 103613-001-116
Well Location: D9
Created: Friday, June 21, 2019 4:25:48 PM

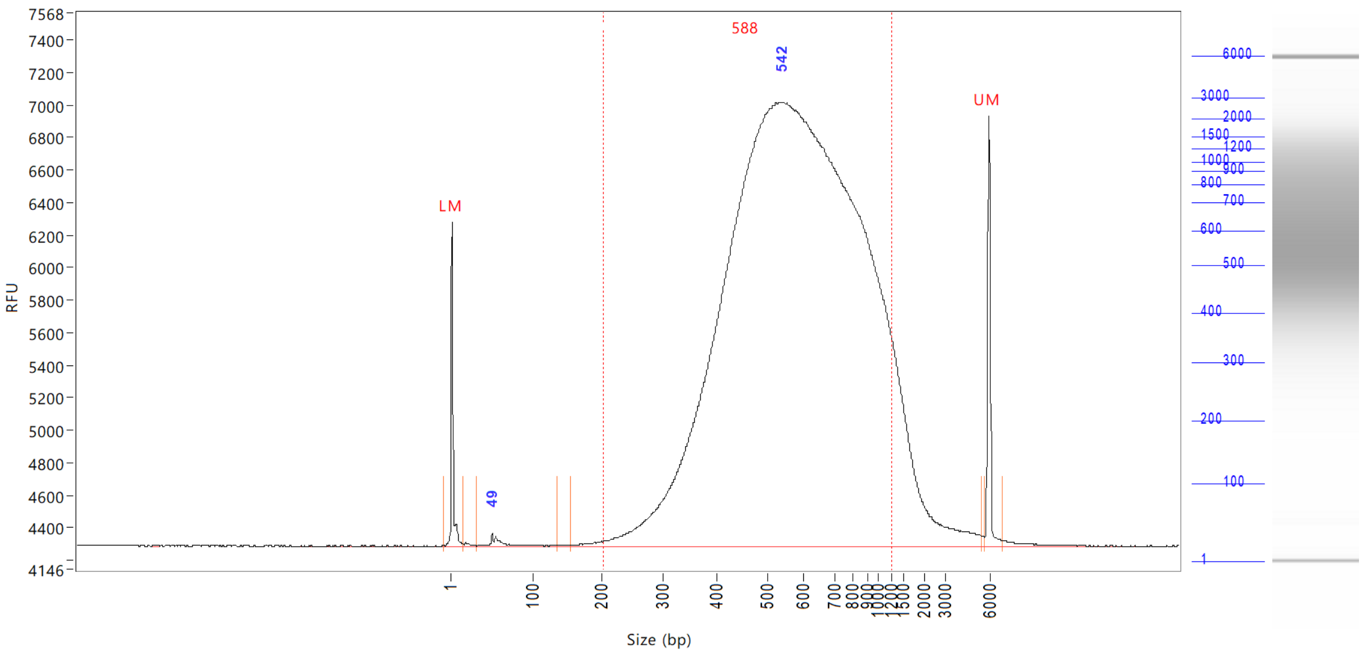

Peak	Size (bp)	Conc. (ng/uL)	From (bp)	To (bp)	RFU
1	1 (LM)	0.0103	0	15	1997
2	49	0.0227	31	134	76
3	542	6.5410	153	5517	2734
4	6000 (UM)	0.0083	5720	6985	2647

TIC:	6.5637	ng/uL
TIM:	16.9484	nmole/L
Total Conc.:	6.5706	ng/uL

Smear Analysis 200 bp to 1200 bp 6.1711 ng/ul 93.9 %Total 17.2703 nmole/L 588 Avg. Size (b.p.) 32.07 %CV

Sample Peak Width (sec): 50 Sample Min Peak Height: 25 Sample Baseline V to V?: Y Sample Baseline V to V pts: 3
Sample Filter: Binomial # of Pts for Filter: 3 Sample Start Region (min): 0 Sample End Region (min): 50
Manual Baseline Start (min): 10 Manual Baseline End (min): 48
Marker Peak Width (sec): 5 Marker Min Peak Height: 200 Marker Baseline V to V?: Y Marker Baseline V to V pts: 3
Lower Marker Selection: First Peak > 200 RFU Upper Marker Selection: Last Peak > 200 RFU
Ladder Size (bp): 1, 100, 200, 300, 400, 500, 600, 700, 800, 900, 1000, 1200, 1500, 2000, 3000, 6000
Quantification Using: Ladder Final Concentration (ng/uL): 0.0830 Dilution Factor: 12.0

 Copyright 2015 Advanced Analytical Technologies, Inc.

Sample: 103613-001-117
Well Location: E9
Created: Friday, June 21, 2019 4:25:48 PM

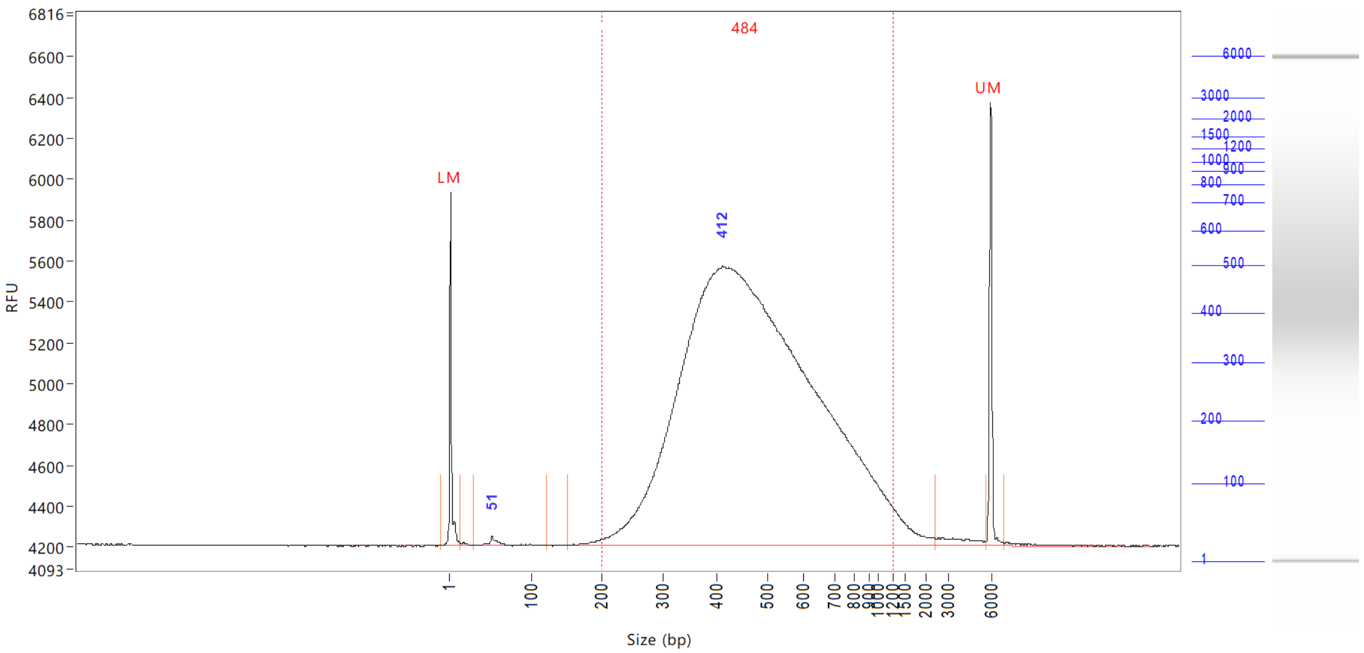

Peak	Size (bp)	Conc. (ng/uL)	From (bp)	To (bp)	RFU
1	1 (LM)	0.0103	0	14	1728
2	51	0.0148	30	120	48
3	412	3.7697	151	2467	1369
4	6000 (UM)	0.0077	5644	6960	2174

TIC:	3.7845	ng/uL
TIM:	12.8606	nmole/L
Total Conc.:	3.8090	ng/uL

Smear Analysis 200 bp to 1200 bp 3.7087 ng/ul 97.4 %Total 12.6095 nmole/L 484 Avg. Size (b.p.) 33.07 %CV

Sample Peak Width (sec): 50 Sample Min Peak Height: 25 Sample Baseline V to V?: Y Sample Baseline V to V pts: 3
Sample Filter: Binomial # of Pts for Filter: 3 Sample Start Region (min): 0 Sample End Region (min): 50
Manual Baseline Start (min): 10 Manual Baseline End (min): 48
Marker Peak Width (sec): 5 Marker Min Peak Height: 200 Marker Baseline V to V?: Y Marker Baseline V to V pts: 3
Lower Marker Selection: First Peak > 200 RFU Upper Marker Selection: Last Peak > 200 RFU
Ladder Size (bp): 1, 100, 200, 300, 400, 500, 600, 700, 800, 900, 1000, 1200, 1500, 2000, 3000, 6000
Quantification Using: Ladder Final Concentration (ng/uL): 0.0830 Dilution Factor: 12.0

 Copyright 2015 Advanced Analytical Technologies, Inc.

Sample: 103613-001-118
Well Location: F9
Created: Friday, June 21, 2019 4:25:48 PM

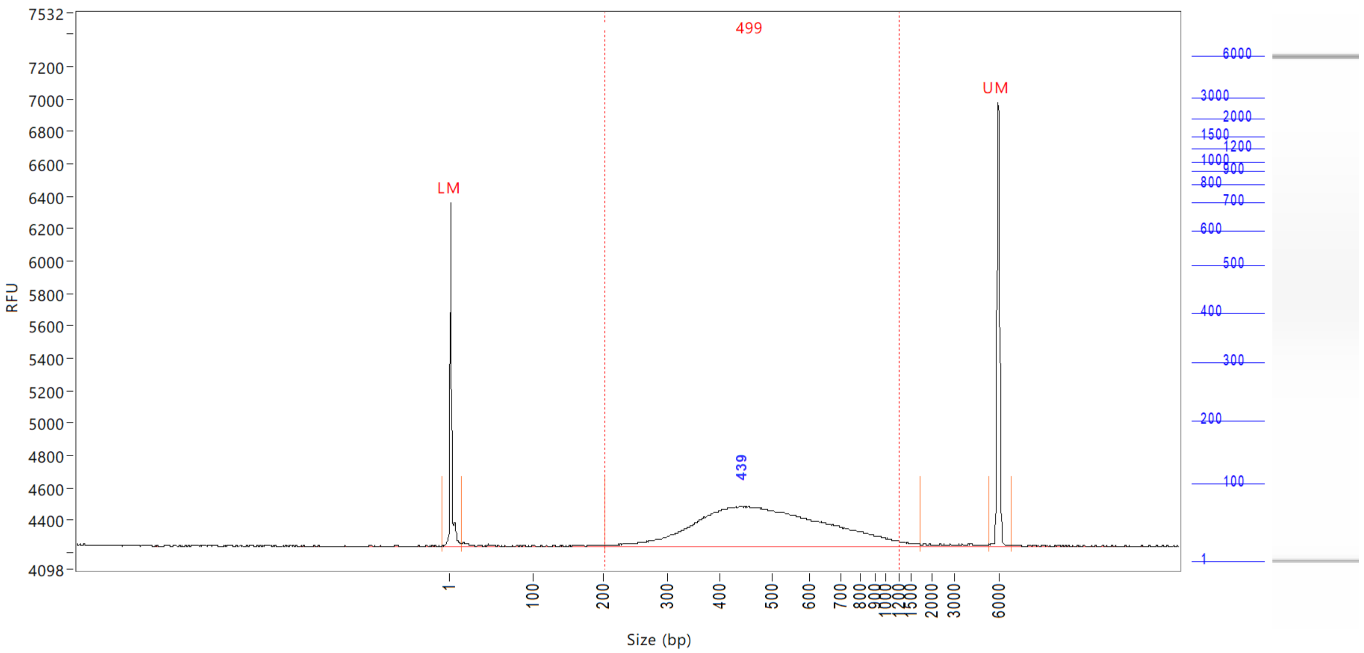

Peak	Size (bp)	Conc. (ng/uL)	From (bp)	To (bp)	RFU
1	1 (LM)	0.0103	0	15	2123
2	439	0.5148	202	1714	246
3	6000 (UM)	0.0075	5390	6884	2743
	TIC:	0.5148	ng/uL		
	TIM:	1.6709	nmole/L		
	Total Conc.:	0.5317	ng/uL		

Smear Analysis 200 bp to 1200 bp 0.5104 ng/ul 96.0 %Total 1.6830 nmole/L 499 Avg. Size (b.p.) 31.43 %CV

Sample Peak Width (sec): 50 Sample Min Peak Height: 25 Sample Baseline V to V?: Y Sample Baseline V to V pts: 3
Sample Filter: Binomial # of Pts for Filter: 3 Sample Start Region (min): 0 Sample End Region (min): 50
Manual Baseline Start (min): 10 Manual Baseline End (min): 48
Marker Peak Width (sec): 5 Marker Min Peak Height: 200 Marker Baseline V to V?: Y Marker Baseline V to V pts: 3
Lower Marker Selection: First Peak > 200 RFU Upper Marker Selection: Last Peak > 200 RFU
Ladder Size (bp): 1, 100, 200, 300, 400, 500, 600, 700, 800, 900, 1000, 1200, 1500, 2000, 3000, 6000
Quantification Using: Ladder Final Concentration (ng/uL): 0.0830 Dilution Factor: 12.0

Copyright 2015 Advanced Analytical Technologies, Inc.

Sample: 103613-001-119
Well Location: G9
Created: Friday, June 21, 2019 4:25:48 PM

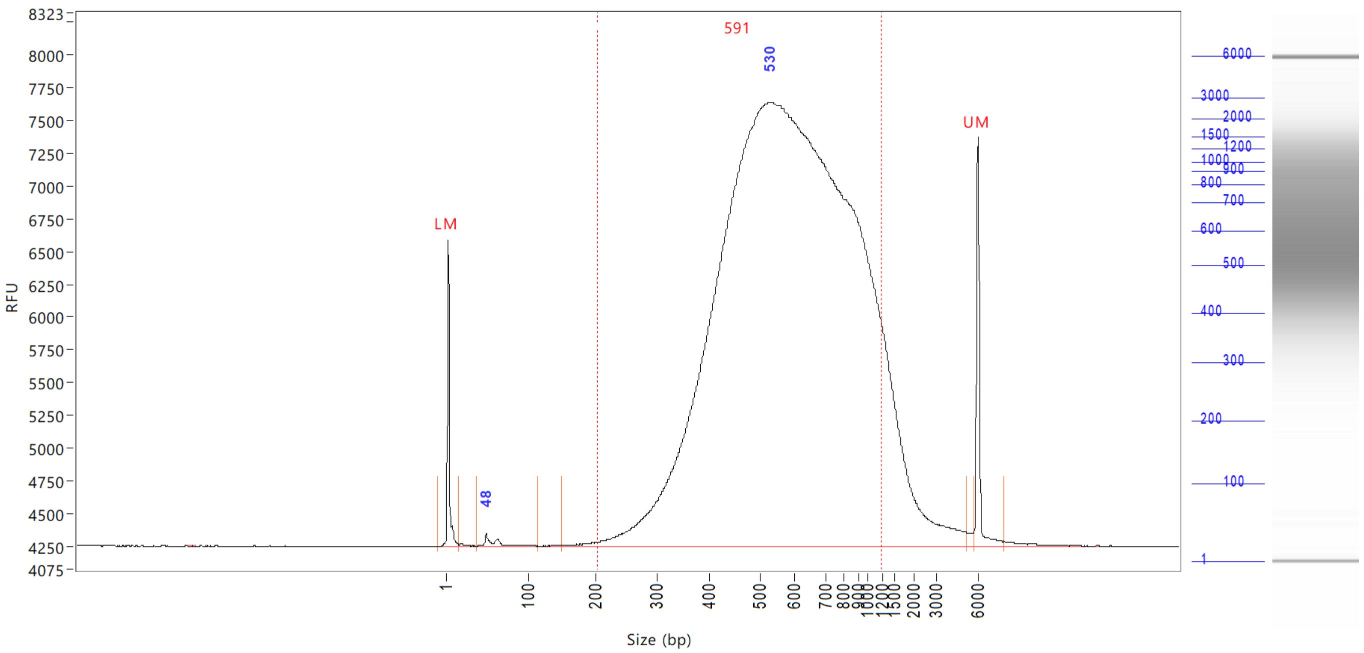

Peak	Size (bp)	Conc. (ng/uL)	From (bp)	To (bp)	RFU
1	1 (LM)	0.0103	0	15	2336
2	48	0.0215	37	114	99
3	530	7.0081	148	5187	3394
4	6000 (UM)	0.0090	5695	7844	3131

TIC:	7.0296	ng/uL
TIM:	17.9214	nmole/L
Total Conc.:	7.0413	ng/uL

Smear Analysis 200 bp to 1200 bp 6.5801 ng/ul 93.4 %Total 18.3244 nmole/L 591 Avg. Size (b.p.) 32.19 %CV

Sample Peak Width (sec): 50 Sample Min Peak Height: 25 Sample Baseline V to V?: Y Sample Baseline V to V pts: 3
Sample Filter: Binomial # of Pts for Filter: 3 Sample Start Region (min): 0 Sample End Region (min): 50
Manual Baseline Start (min): 10 Manual Baseline End (min): 48
Marker Peak Width (sec): 5 Marker Min Peak Height: 200 Marker Baseline V to V?: Y Marker Baseline V to V pts: 3
Lower Marker Selection: First Peak > 200 RFU Upper Marker Selection: Last Peak > 200 RFU
Ladder Size (bp): 1, 100, 200, 300, 400, 500, 600, 700, 800, 900, 1000, 1200, 1500, 2000, 3000, 6000
Quantification Using: Ladder Final Concentration (ng/uL): 0.0830 Dilution Factor: 12.0

Copyright 2015 Advanced Analytical Technologies, Inc.

Sample: 103613-001-120
Well Location: H9
Created: Friday, June 21, 2019 4:25:48 PM

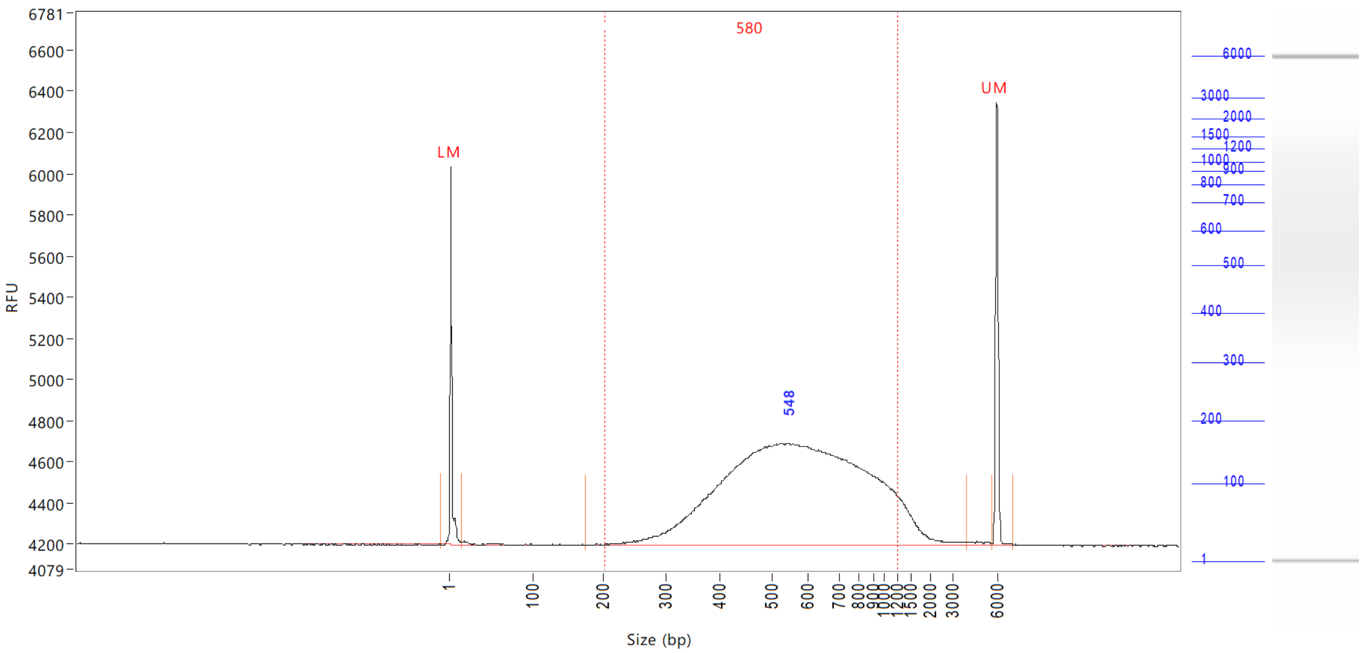

Peak	Size (bp)	Conc. (ng/uL)	From (bp)	To (bp)	RFU
1	1 (LM)	0.0103	0	15	1836
2	548	1.3774	174	3941	494
3	6000 (UM)	0.0070	5644	7137	2156

TIC:	1.3774	ng/uL
TIM:	3.6094	nmole/L
Total Conc.:	1.3858	ng/uL

Smear Analysis	200 bp to 1200 bp	1.3136 ng/ul	94.8 %Total	3.7300 nmole/L	580 Avg. Size (b.p.)	32.72 %CV

Sample Peak Width (sec): 50 Sample Min Peak Height: 25 Sample Baseline V to V?: Y Sample Baseline V to V pts: 3
Sample Filter: Binomial # of Pts for Filter: 3 Sample Start Region (min): 0 Sample End Region (min): 50
Manual Baseline Start (min): 10 Manual Baseline End (min): 48
Marker Peak Width (sec): 5 Marker Min Peak Height: 200 Marker Baseline V to V?: Y Marker Baseline V to V pts: 3
Lower Marker Selection: First Peak > 200 RFU Upper Marker Selection: Last Peak > 200 RFU
Ladder Size (bp): 1, 100, 200, 300, 400, 500, 600, 700, 800, 900, 1000, 1200, 1500, 2000, 3000, 6000
Quantification Using: Ladder Final Concentration (ng/uL): 0.0830 Dilution Factor: 12.0

 Copyright 2015 Advanced Analytical Technologies, Inc.

Sample: 103613-001-121
Well Location: A10
Created: Friday, June 21, 2019 4:25:48 PM

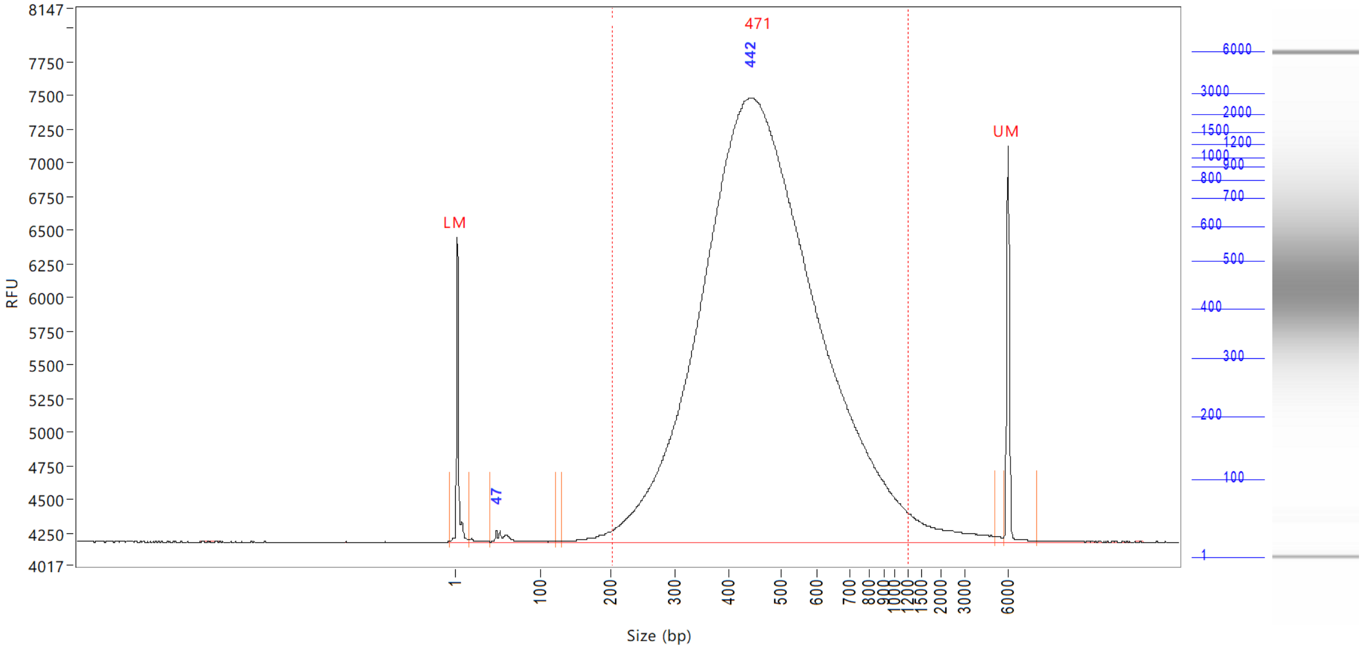

Peak	Size (bp)	Conc. (ng/uL)	From (bp)	To (bp)	RFU
1	1 (LM)	0.0103	0	15	2261
2	47	0.0232	40	121	87
3	442	5.9690	130	5110	3298
4	6000 (UM)	0.0079	5695	7995	2936

TIC:	5.9921	ng/uL
TIM:	20.4450	nmole/L
Total Conc.:	5.9995	ng/uL

Smear Analysis	200 bp to 1200 bp	5.8582 ng/ul	97.6 %Total	20.4507 nmole/L	471 Avg. Size (b.p.)	29.40 %CV

Sample Peak Width (sec): 50 Sample Min Peak Height: 25 Sample Baseline V to V?: Y Sample Baseline V to V pts: 3
Sample Filter: Binomial # of Pts for Filter: 3 Sample Start Region (min): 0 Sample End Region (min): 50
Manual Baseline Start (min): 10 Manual Baseline End (min): 48
Marker Peak Width (sec): 5 Marker Min Peak Height: 200 Marker Baseline V to V?: Y Marker Baseline V to V pts: 3
Lower Marker Selection: First Peak > 200 RFU Upper Marker Selection: Last Peak > 200 RFU
Ladder Size (bp): 1, 100, 200, 300, 400, 500, 600, 700, 800, 900, 1000, 1200, 1500, 2000, 3000, 6000
Quantification Using: Ladder Final Concentration (ng/uL): 0.0830 Dilution Factor: 12.0

Copyright 2015 Advanced Analytical Technologies, Inc.

Sample: 103613-001-122
Well Location: B10
Created: Friday, June 21, 2019 4:25:48 PM

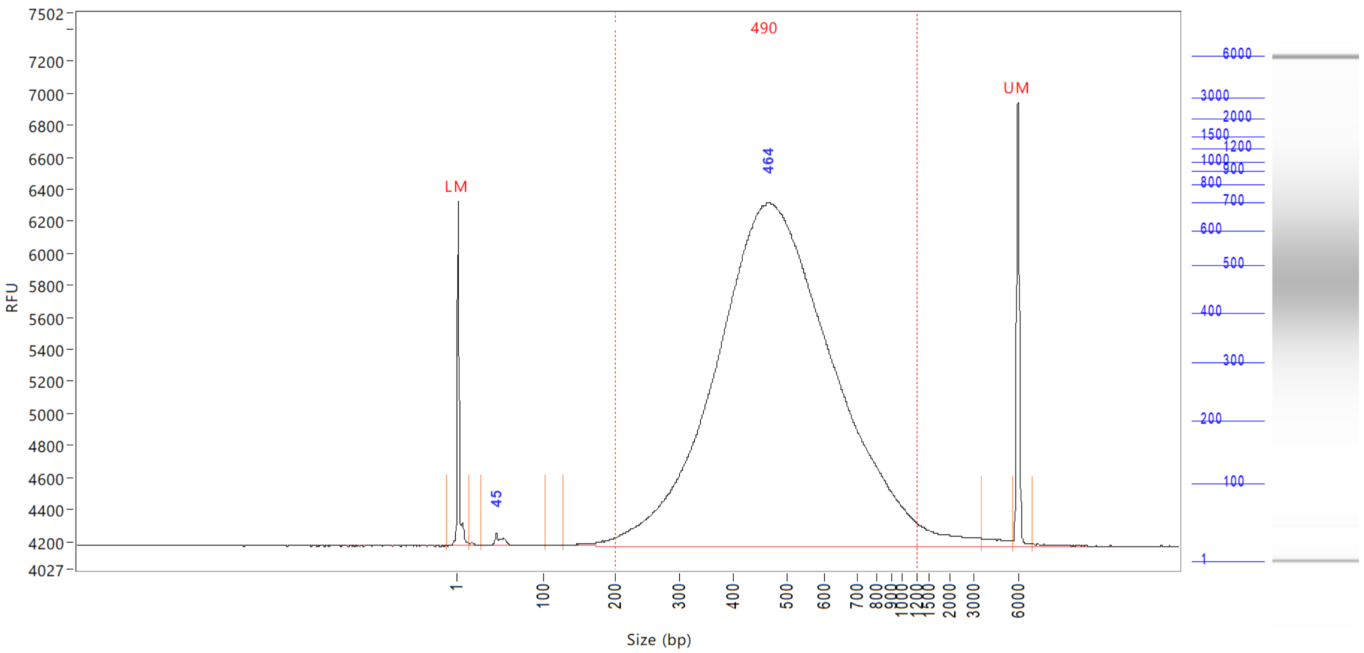

Peak	Size (bp)	Conc. (ng/uL)	From (bp)	To (bp)	RFU
1	1 (LM)	0.0103	0	15	2155
2	45	0.0189	28	104	81
3	464	4.0377	129	3586	2146
4	6000 (UM)	0.0081	5670	6985	2777
	TIC:	4.0565	ng/uL		
	TIM:	13.6411	nmole/L		
	Total Conc.:	4.0735	ng/uL		

Smear Analysis 200 bp to 1200 bp 3.9645 ng/ul 97.3 %Total 13.3230 nmole/L 490 Avg. Size (b.p.) 28.86 %CV

Sample Peak Width (sec): 50 Sample Min Peak Height: 25 Sample Baseline V to V?: Y Sample Baseline V to V pts: 3
Sample Filter: Binomial # of Pts for Filter: 3 Sample Start Region (min): 0 Sample End Region (min): 50
Manual Baseline Start (min): 10 Manual Baseline End (min): 48
Marker Peak Width (sec): 5 Marker Min Peak Height: 200 Marker Baseline V to V?: Y Marker Baseline V to V pts: 3
Lower Marker Selection: First Peak > 200 RFU Upper Marker Selection: Last Peak > 200 RFU
Ladder Size (bp): 1, 100, 200, 300, 400, 500, 600, 700, 800, 900, 1000, 1200, 1500, 2000, 3000, 6000
Quantification Using: Ladder Final Concentration (ng/uL): 0.0830 Dilution Factor: 12.0

Copyright 2015 Advanced Analytical Technologies, Inc.

Sample: 103613-001-123
Well Location: C10
Created: Friday, June 21, 2019 4:25:48 PM

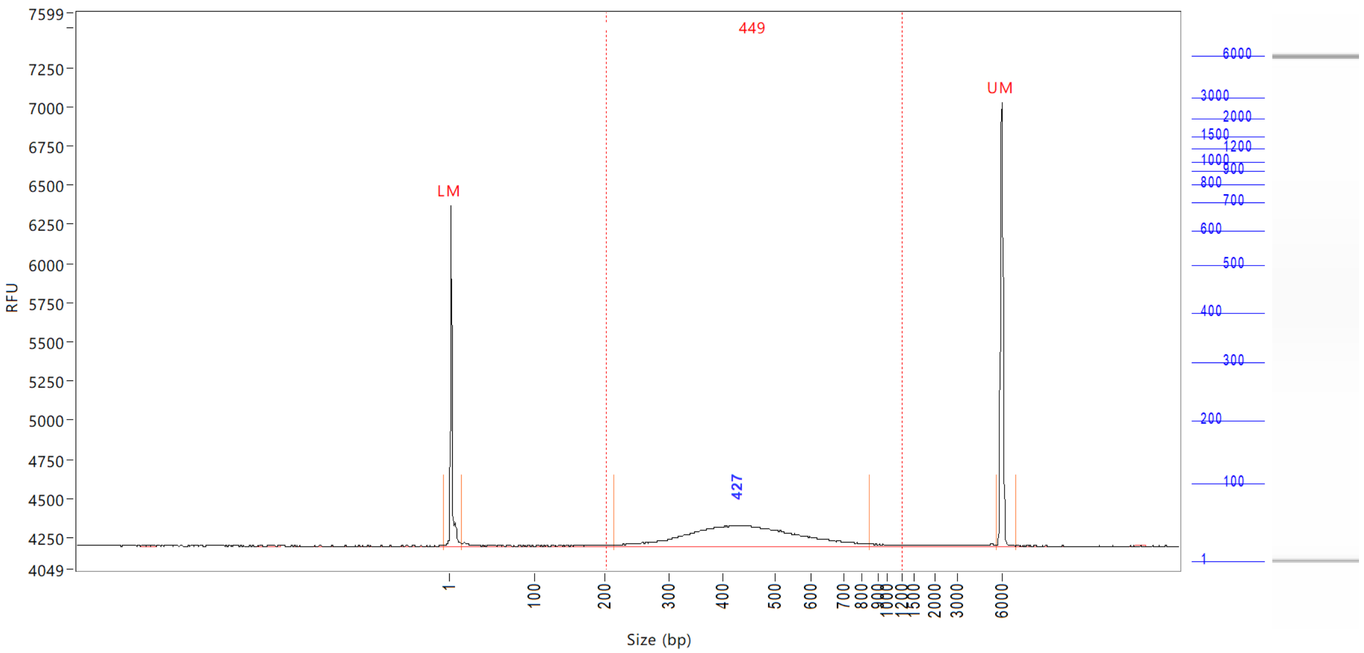

Peak	Size (bp)	Conc. (ng/uL)	From (bp)	To (bp)	RFU
1	1 (LM)	0.0103	0	15	2175
2	427	0.2365	214	853	131
3	6000 (UM)	0.0077	5670	6935	2835

TIC:	0.2365	ng/uL
TIM:	0.8786	nmole/L
Total Conc.:	0.2545	ng/uL

Smear Analysis 200 bp to 1200 bp 0.2406 ng/ul 94.5 %Total 0.8825 nmole/L 449 Avg. Size (b.p.) 28.29 %CV

Sample Peak Width (sec): 50 Sample Min Peak Height: 25 Sample Baseline V to V?: Y Sample Baseline V to V pts: 3
Sample Filter: Binomial # of Pts for Filter: 3 Sample Start Region (min): 0 Sample End Region (min): 50
Manual Baseline Start (min): 10 Manual Baseline End (min): 48
Marker Peak Width (sec): 5 Marker Min Peak Height: 200 Marker Baseline V to V?: Y Marker Baseline V to V pts: 3
Lower Marker Selection: First Peak > 200 RFU Upper Marker Selection: Last Peak > 200 RFU
Ladder Size (bp): 1, 100, 200, 300, 400, 500, 600, 700, 800, 900, 1000, 1200, 1500, 2000, 3000, 6000
Quantification Using: Ladder Final Concentration (ng/uL): 0.0830 Dilution Factor: 12.0

 Copyright 2015 Advanced Analytical Technologies, Inc.

Sample: 103613-001-124
Well Location: D10
Created: Friday, June 21, 2019 4:25:48 PM

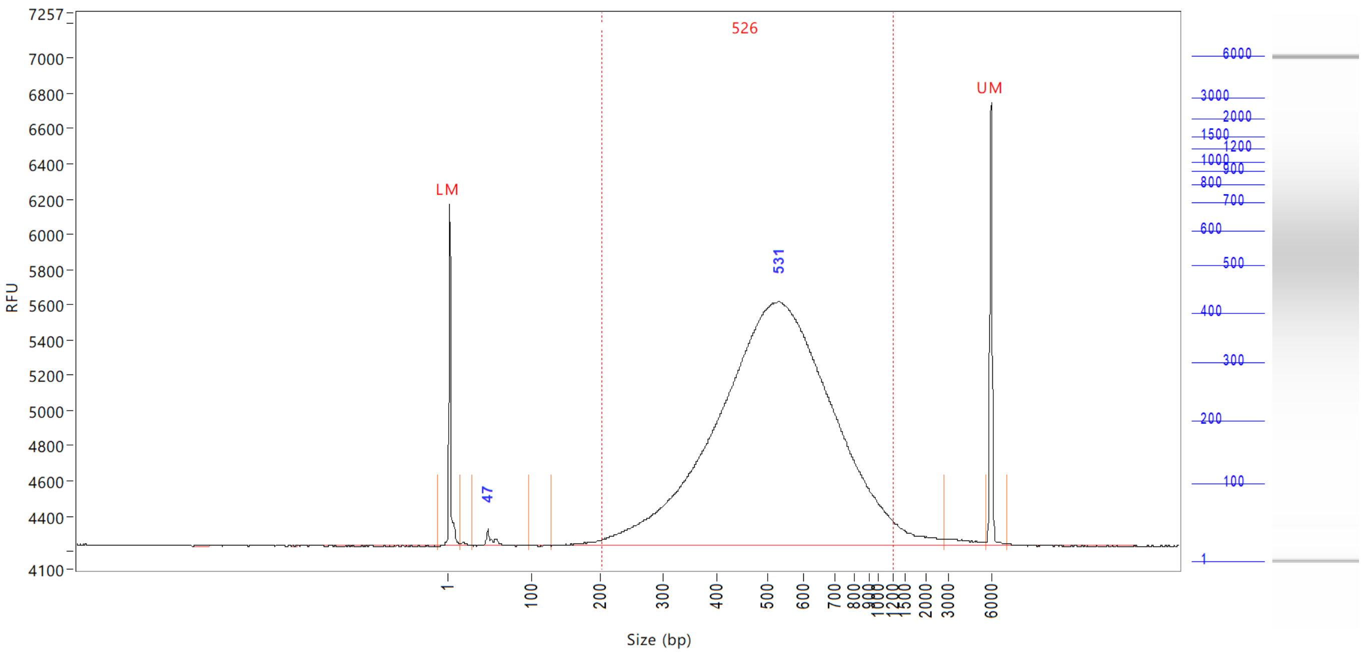

Peak	Size (bp)	Conc. (ng/uL)	From (bp)	To (bp)	RFU
1	1 (LM)	0.0103	0	15	1941
2	47	0.0169	29	96	94
3	531	2.7944	127	2834	1386
4	6000 (UM)	0.0077	5644	7086	2520

TIC:	2.8114	ng/uL
TIM:	8.9977	nmole/L
Total Conc.:	2.8295	ng/uL

Smear Analysis 200 bp to 1200 bp 2.7393 ng/ul 96.8 %Total 8.5715 nmole/L 526 Avg. Size (b.p.) 29.15 %CV

Sample Peak Width (sec): 50 Sample Min Peak Height: 25 Sample Baseline V to V?: Y Sample Baseline V to V pts: 3
Sample Filter: Binomial # of Pts for Filter: 3 Sample Start Region (min): 0 Sample End Region (min): 50
Manual Baseline Start (min): 10 Manual Baseline End (min): 48
Marker Peak Width (sec): 5 Marker Min Peak Height: 200 Marker Baseline V to V?: Y Marker Baseline V to V pts: 3
Lower Marker Selection: First Peak > 200 RFU Upper Marker Selection: Last Peak > 200 RFU
Ladder Size (bp): 1, 100, 200, 300, 400, 500, 600, 700, 800, 900, 1000, 1200, 1500, 2000, 3000, 6000
Quantification Using: Ladder Final Concentration (ng/uL): 0.0830 Dilution Factor: 12.0

Copyright 2015 Advanced Analytical Technologies, Inc.

Sample: 103613-001-125
Well Location: E10
Created: Friday, June 21, 2019 4:25:48 PM

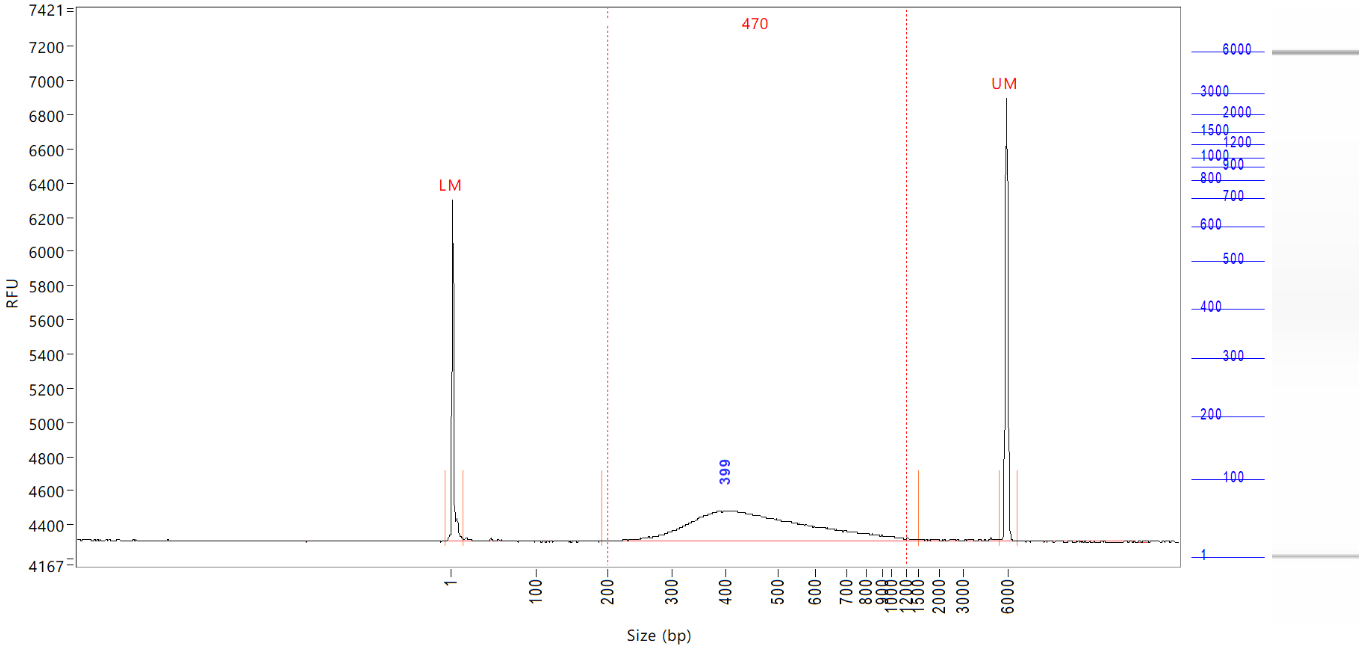

Peak	Size (bp)	Conc. (ng/uL)	From (bp)	To (bp)	RFU
1	1 (LM)	0.0103	0	15	1992
2	399	0.3730	193	1510	177
3	6000 (UM)	0.0077	5466	6657	2596
	TIC:	0.3730	ng/uL		
	TIM:	1.2921	nmole/L		
	Total Conc.:	0.3870	ng/uL		

Smear Analysis 200 bp to 1200 bp 0.3707 ng/ul 95.8 %Total 1.2970 nmole/L 470 Avg. Size (b.p.) 31.68 %CV

Sample Peak Width (sec): 50 Sample Min Peak Height: 25 Sample Baseline V to V?: Y Sample Baseline V to V pts: 3
Sample Filter: Binomial # of Pts for Filter: 3 Sample Start Region (min): 0 Sample End Region (min): 50
Manual Baseline Start (min): 10 Manual Baseline End (min): 48
Marker Peak Width (sec): 5 Marker Min Peak Height: 200 Marker Baseline V to V?: Y Marker Baseline V to V pts: 3
Lower Marker Selection: First Peak > 200 RFU Upper Marker Selection: Last Peak > 200 RFU
Ladder Size (bp): 1, 100, 200, 300, 400, 500, 600, 700, 800, 900, 1000, 1200, 1500, 2000, 3000, 6000
Quantification Using: Ladder Final Concentration (ng/uL): 0.0830 Dilution Factor: 12.0

 Copyright 2015 Advanced Analytical Technologies, Inc.

Sample: 103613-001-126
Well Location: F10
Created: Friday, June 21, 2019 4:25:48 PM

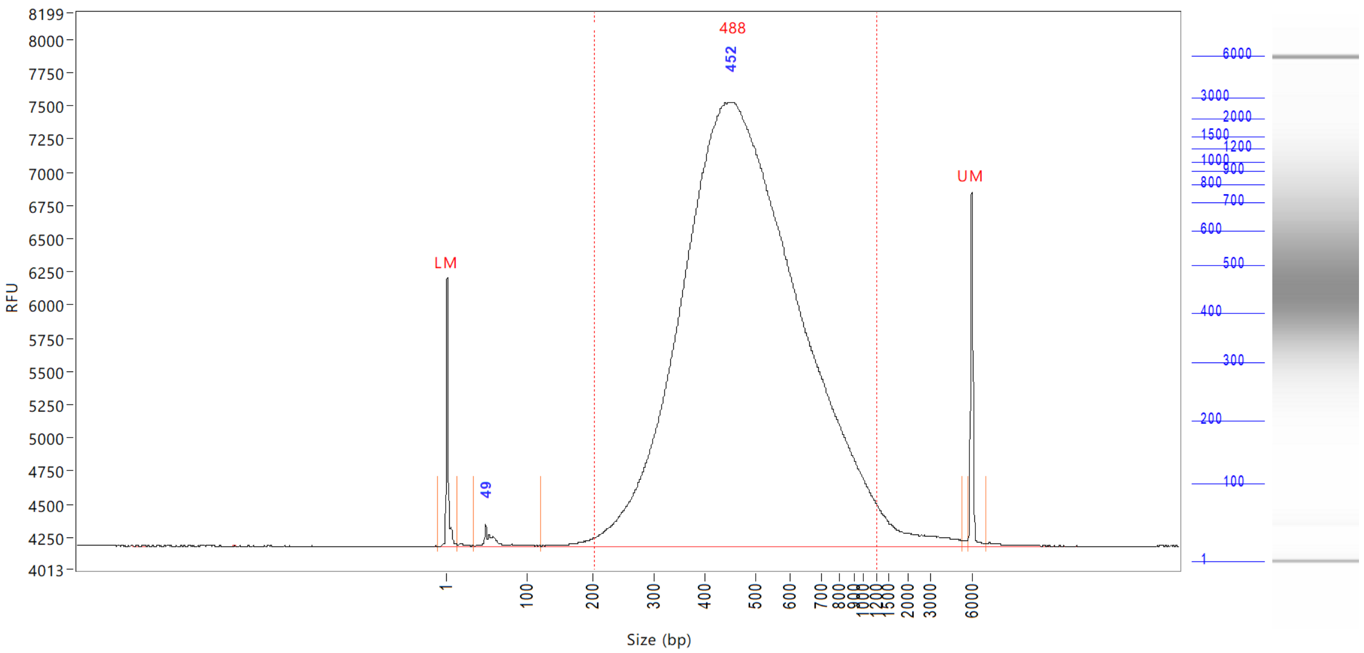

Peak	Size (bp)	Conc. (ng/uL)	From (bp)	To (bp)	RFU
1	1 (LM)	0.0103	0	15	2020
2	49	0.0360	35	121	166
3	452	6.8553	121	5288	3346
4	6000 (UM)	0.0080	5695	6960	2665

TIC:	6.8913	ng/uL
TIM:	22.8721	nmole/L
Total Conc.:	6.8977	ng/uL

Smear Analysis 200 bp to 1200 bp 6.7234 ng/ul 97.5 %Total 22.6689 nmole/L 488 Avg. Size (b.p.) 30.42 %CV

Sample Peak Width (sec): 50 Sample Min Peak Height: 25 Sample Baseline V to V?: Y Sample Baseline V to V pts: 3
Sample Filter: Binomial # of Pts for Filter: 3 Sample Start Region (min): 0 Sample End Region (min): 50
Manual Baseline Start (min): 10 Manual Baseline End (min): 48
Marker Peak Width (sec): 5 Marker Min Peak Height: 200 Marker Baseline V to V?: Y Marker Baseline V to V pts: 3
Lower Marker Selection: First Peak > 200 RFU Upper Marker Selection: Last Peak > 200 RFU
Ladder Size (bp): 1, 100, 200, 300, 400, 500, 600, 700, 800, 900, 1000, 1200, 1500, 2000, 3000, 6000
Quantification Using: Ladder Final Concentration (ng/uL): 0.0830 Dilution Factor: 12.0

 Copyright 2015 Advanced Analytical Technologies, Inc.

Sample: 103613-001-127
Well Location: G10
Created: Friday, June 21, 2019 4:25:48 PM

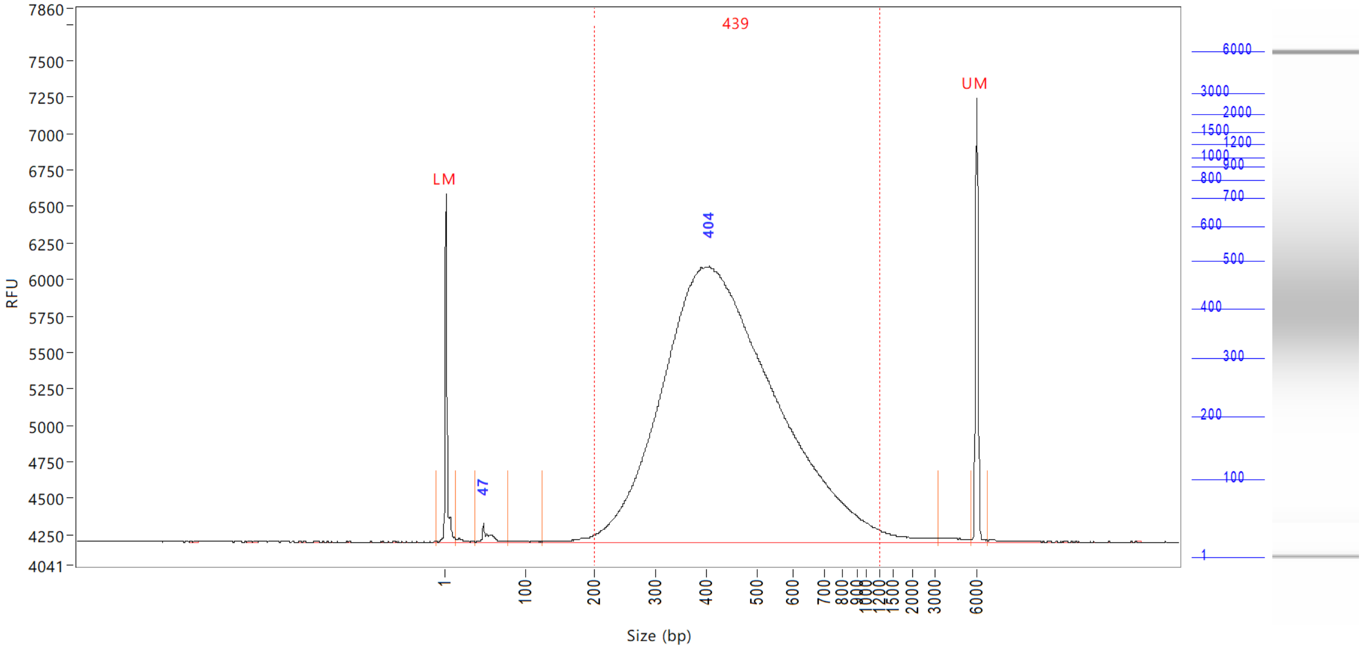

Peak	Size (bp)	Conc. (ng/uL)	From (bp)	To (bp)	RFU
1	1 (LM)	0.0103	0	15	2391
2	47	0.0165	38	77	132
3	404	3.2161	123	3331	1890
4	6000 (UM)	0.0075	5568	6808	3049

TIC:	3.2326	ng/uL
TIM:	12.3230	nmole/L
Total Conc.:	3.2456	ng/uL

Smear Analysis 200 bp to 1200 bp 3.1823 ng/ul 98.0 %Total 11.9212 nmole/L 439 Avg. Size (b.p.) 29.85 %CV

Sample Peak Width (sec): 50 Sample Min Peak Height: 25 Sample Baseline V to V?: Y Sample Baseline V to V pts: 3
Sample Filter: Binomial # of Pts for Filter: 3 Sample Start Region (min): 0 Sample End Region (min): 50
Manual Baseline Start (min): 10 Manual Baseline End (min): 48
Marker Peak Width (sec): 5 Marker Min Peak Height: 200 Marker Baseline V to V?: Y Marker Baseline V to V pts: 3
Lower Marker Selection: First Peak > 200 RFU Upper Marker Selection: Last Peak > 200 RFU
Ladder Size (bp): 1, 100, 200, 300, 400, 500, 600, 700, 800, 900, 1000, 1200, 1500, 2000, 3000, 6000
Quantification Using: Ladder Final Concentration (ng/uL): 0.0830 Dilution Factor: 12.0

 Copyright 2015 Advanced Analytical Technologies, Inc.

Sample: 103613-001-128
Well Location: H10
Created: Friday, June 21, 2019 4:25:48 PM

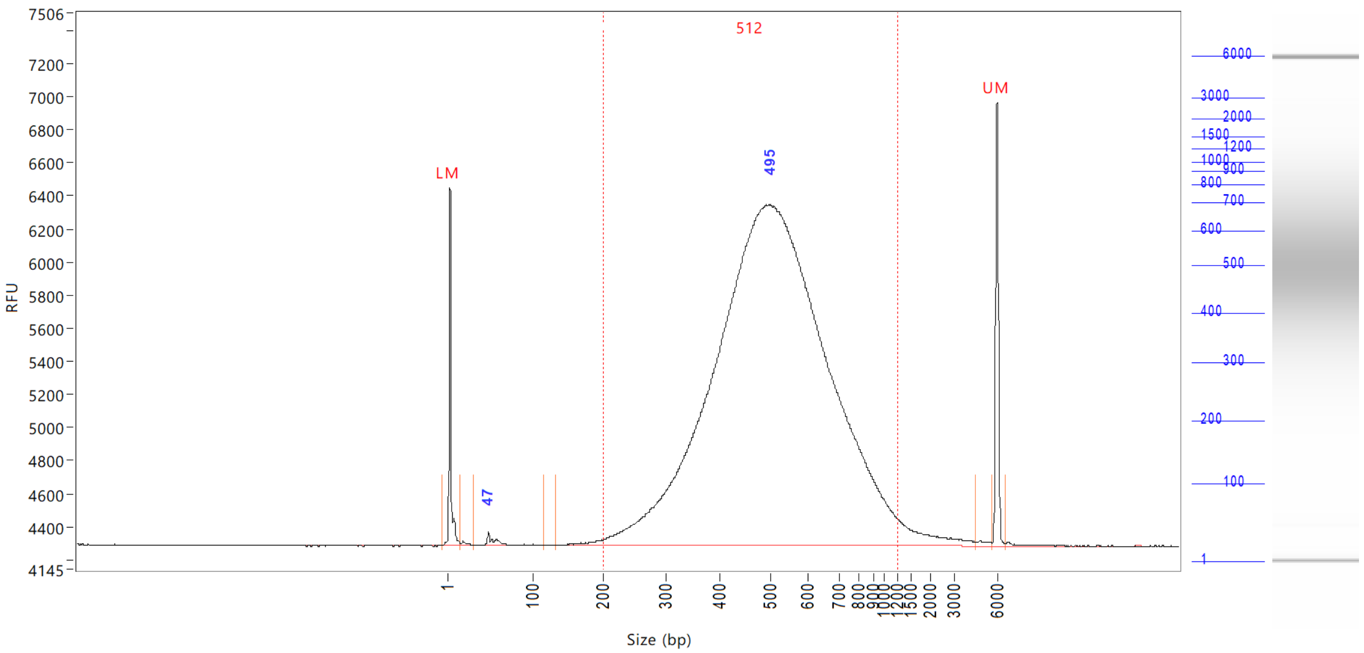

Peak	Size (bp)	Conc. (ng/uL)	From (bp)	To (bp)	RFU
1	1 (LM)	0.0103	0	15	2156
2	47	0.0142	30	115	84
3	495	3.6702	132	4551	2061
4	6000 (UM)	0.0075	5644	6530	2684

TIC:	3.6845	ng/uL
TIM:	11.7267	nmole/L
Total Conc.:	3.6913	ng/uL

Smear Analysis 200 bp to 1200 bp 3.6053 ng/ul 97.7 %Total 11.5819 nmole/L 512 Avg. Size (b.p.) 28.36 %CV

Sample Peak Width (sec): 50 Sample Min Peak Height: 25 Sample Baseline V to V?: Y Sample Baseline V to V pts: 3
Sample Filter: Binomial # of Pts for Filter: 3 Sample Start Region (min): 0 Sample End Region (min): 50
Manual Baseline Start (min): 10 Manual Baseline End (min): 48
Marker Peak Width (sec): 5 Marker Min Peak Height: 200 Marker Baseline V to V?: Y Marker Baseline V to V pts: 3
Lower Marker Selection: First Peak > 200 RFU Upper Marker Selection: Last Peak > 200 RFU
Ladder Size (bp): 1, 100, 200, 300, 400, 500, 600, 700, 800, 900, 1000, 1200, 1500, 2000, 3000, 6000
Quantification Using: Ladder Final Concentration (ng/uL): 0.0830 Dilution Factor: 12.0

Sample: 103613-001-129
Well Location: A11
Created: Friday, June 21, 2019 4:25:48 PM

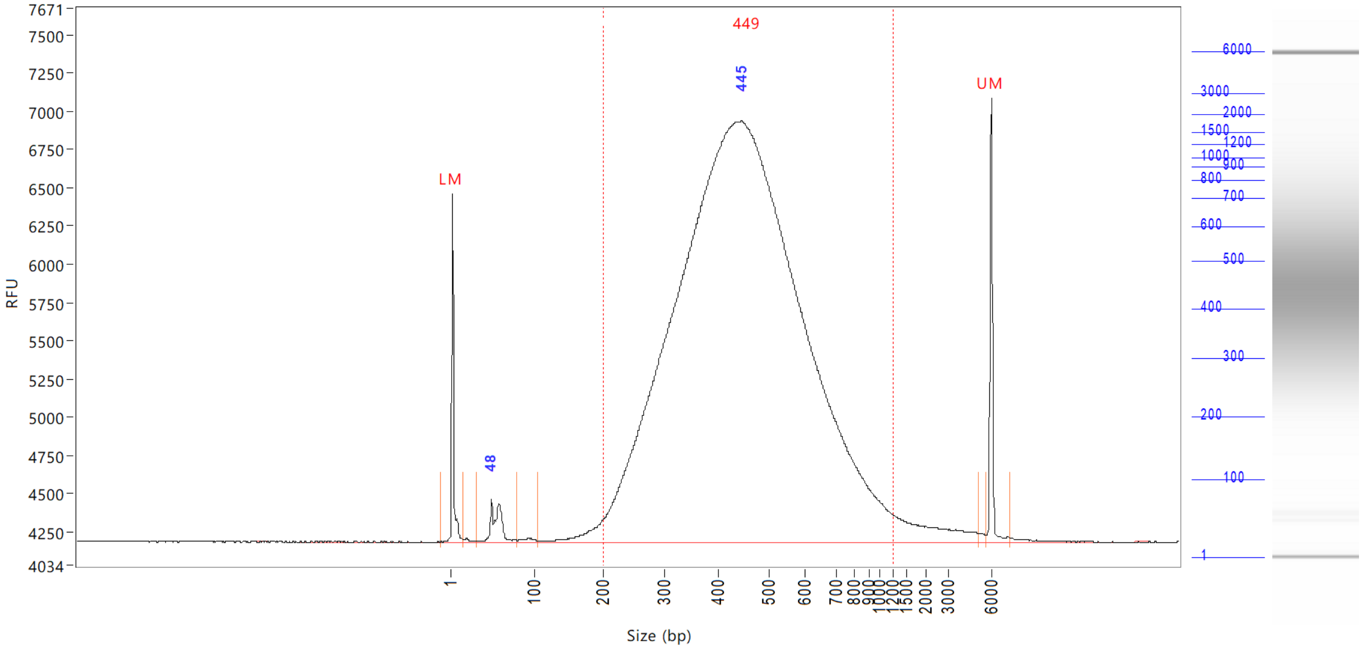

Peak	Size (bp)	Conc. (ng/uL)	From (bp)	To (bp)	RFU
1	1 (LM)	0.0103	0	15	2273
2	48	0.0620	31	78	283
3	445	5.6399	106	5110	2752
4	6000 (UM)	0.0080	5619	7313	2906

TIC:	5.7018	ng/uL
TIM:	21.3763	nmole/L
Total Conc.:	5.7164	ng/uL

Smear Analysis	200 bp to 1200 bp	5.5045 ng/ul	96.3 %Total	20.1892 nmole/L	449 Avg. Size (b.p.)	31.42 %CV

```
Sample Peak Width (sec): 50   Sample Min Peak Height: 25    Sample Baseline V to V?: Y    Sample Baseline V to V pts: 3
Sample Filter: Binomial       # of Pts for Filter: 3        Sample Start Region (min): 0  Sample End Region (min): 50
Manual Baseline Start (min): 10     Manual Baseline End (min): 48
Marker Peak Width (sec): 5    Marker Min Peak Height: 200   Marker Baseline V to V?: Y    Marker Baseline V to V pts: 3
Lower Marker Selection: First Peak > 200 RFU                Upper Marker Selection: Last Peak > 200 RFU
Ladder Size (bp): 1, 100, 200, 300, 400, 500, 600, 700, 800, 900, 1000, 1200, 1500, 2000, 3000, 6000
Quantification Using: Ladder          Final Concentration (ng/uL): 0.0830          Dilution Factor: 12.0
```


Copyright 2015 Advanced Analytical Technologies, Inc.

Sample: 103613-001-130
Well Location: B11
Created: Friday, June 21, 2019 4:25:48 PM

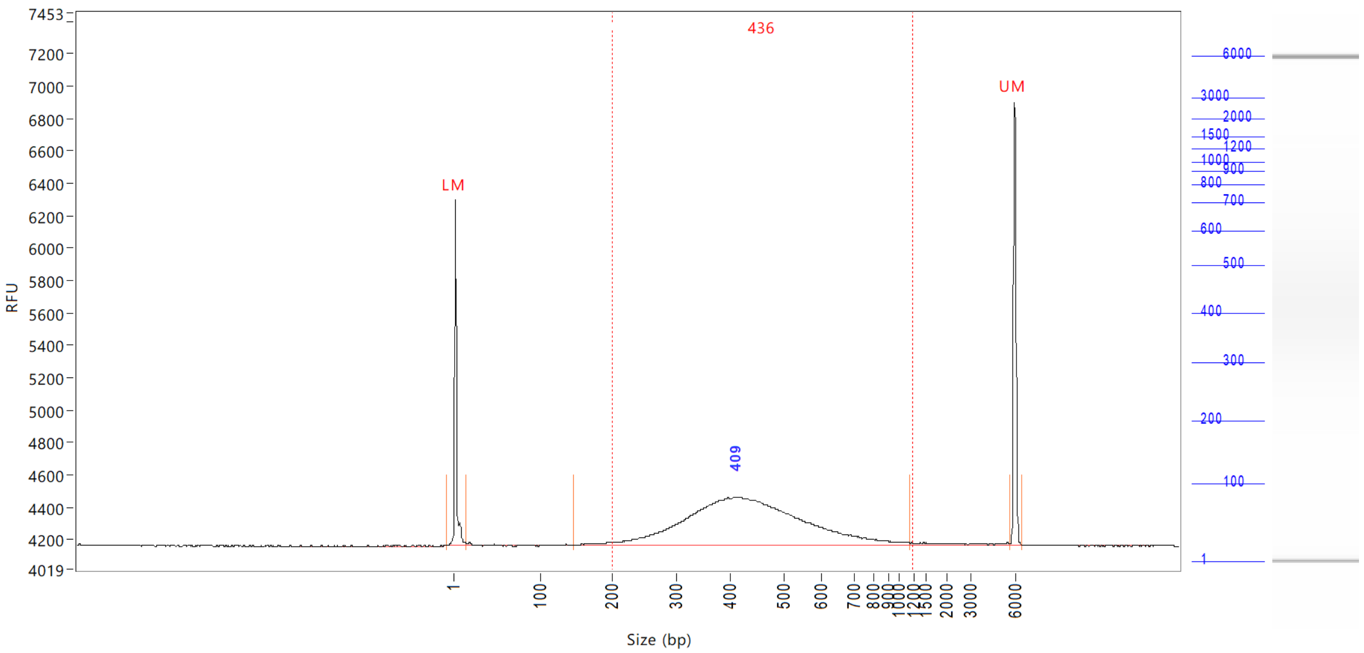

Peak	Size (bp)	Conc. (ng/uL)	From (bp)	To (bp)	RFU
1	1 (LM)	0.0103	0	14	2140
2	409	0.5889	145	1157	299
3	6000 (UM)	0.0074	5695	6505	2739

TIC:	0.5889	ng/uL
TIM:	2.2480	nmole/L
Total Conc.:	0.6089	ng/uL

Smear Analysis 200 bp to 1200 bp 0.5804 ng/ul 95.3 %Total 2.1895 nmole/L 436 Avg. Size (b.p.) 30.24 %CV

Sample Peak Width (sec): 50 Sample Min Peak Height: 25 Sample Baseline V to V?: Y Sample Baseline V to V pts: 3
Sample Filter: Binomial # of Pts for Filter: 3 Sample Start Region (min): 0 Sample End Region (min): 50
Manual Baseline Start (min): 10 Manual Baseline End (min): 48
Marker Peak Width (sec): 5 Marker Min Peak Height: 200 Marker Baseline V to V?: Y Marker Baseline V to V pts: 3
Lower Marker Selection: First Peak > 200 RFU Upper Marker Selection: Last Peak > 200 RFU
Ladder Size (bp): 1, 100, 200, 300, 400, 500, 600, 700, 800, 900, 1000, 1200, 1500, 2000, 3000, 6000
Quantification Using: Ladder Final Concentration (ng/uL): 0.0830 Dilution Factor: 12.0

 Copyright 2015 Advanced Analytical Technologies, Inc.

Sample: 103613-001-131
Well Location: C11
Created: Friday, June 21, 2019 4:25:48 PM

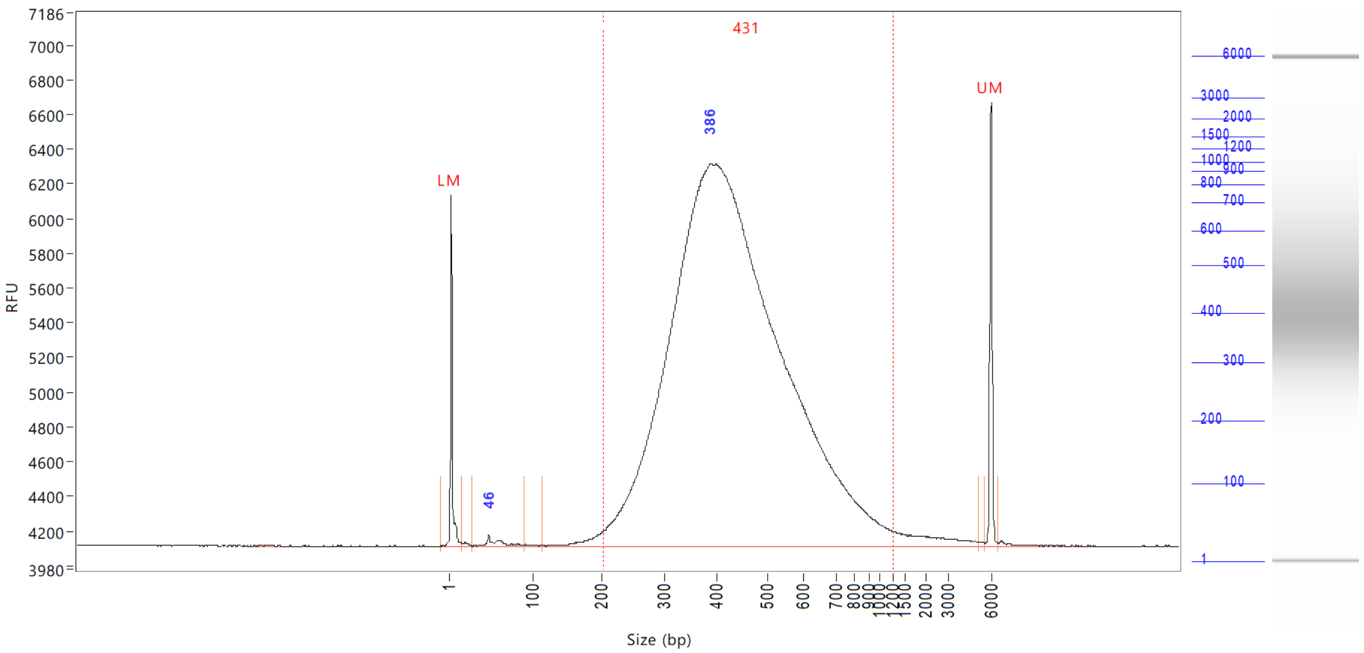

Peak	Size (bp)	Conc. (ng/uL)	From (bp)	To (bp)	RFU
1	1 (LM)	0.0103	0	15	2019
2	46	0.0180	27	89	68
3	386	4.4189	113	5110	2205
4	6000 (UM)	0.0075	5568	6505	2561

TIC:	4.4370	ng/uL
TIM:	16.6407	nmole/L
Total Conc.:	4.4441	ng/uL

Smear Analysis	200 bp to 1200 bp	4.3422 ng/ul	97.7 %Total	16.5758 nmole/L	431 Avg. Size (b.p.)	29.74 %CV

Sample Peak Width (sec): 50 Sample Min Peak Height: 25 Sample Baseline V to V?: Y Sample Baseline V to V pts: 3
Sample Filter: Binomial # of Pts for Filter: 3 Sample Start Region (min): 0 Sample End Region (min): 50
Manual Baseline Start (min): 10 Manual Baseline End (min): 48
Marker Peak Width (sec): 5 Marker Min Peak Height: 200 Marker Baseline V to V?: Y Marker Baseline V to V pts: 3
Lower Marker Selection: First Peak > 200 RFU Upper Marker Selection: Last Peak > 200 RFU
Ladder Size (bp): 1, 100, 200, 300, 400, 500, 600, 700, 800, 900, 1000, 1200, 1500, 2000, 3000, 6000
Quantification Using: Ladder Final Concentration (ng/uL): 0.0830 Dilution Factor: 12.0

Copyright 2015 Advanced Analytical Technologies, Inc.

Sample: 103613-001-132
Well Location: D11
Created: Friday, June 21, 2019 4:25:48 PM

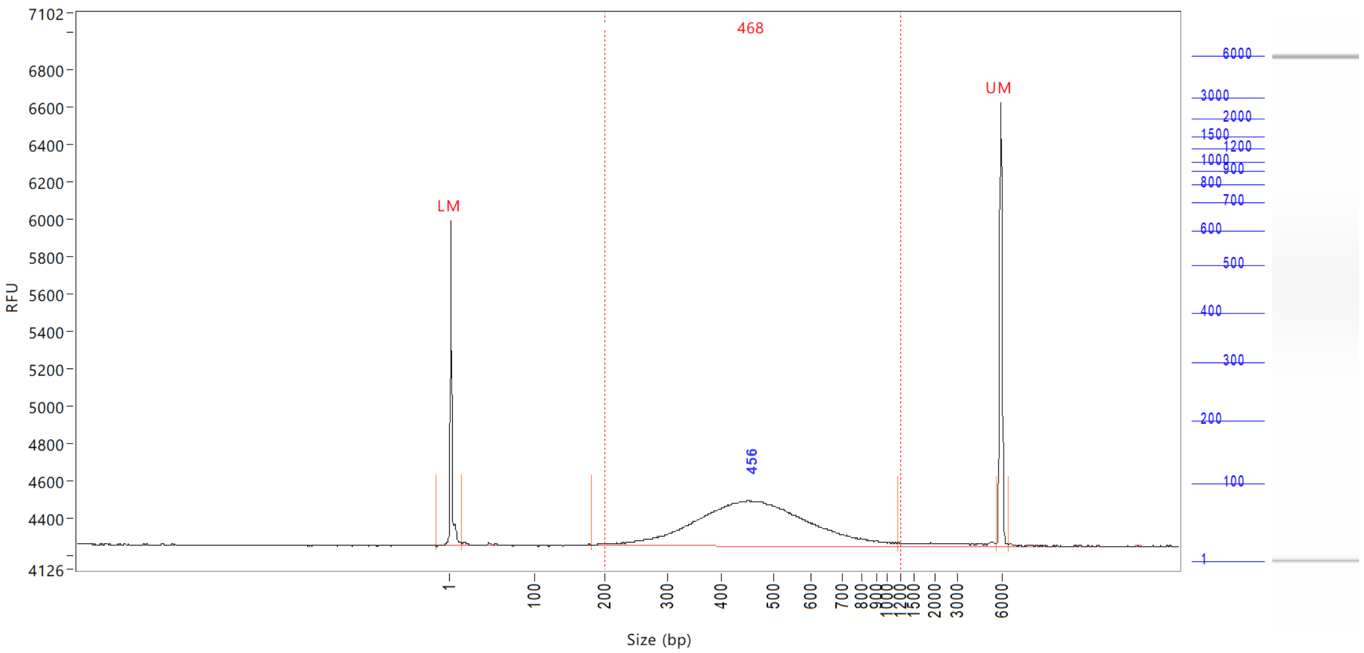

Peak	Size (bp)	Conc. (ng/uL)	From (bp)	To (bp)	RFU
1	1 (LM)	0.0103	0	15	1734
2	456	0.5554	182	1162	241
3	6000 (UM)	0.0080	5695	6505	2378

TIC:	0.5554	ng/uL
TIM:	1.9610	nmole/L
Total Conc.:	0.5839	ng/uL

Smear Analysis 200 bp to 1200 bp 0.5539 ng/ul 94.9 %Total 1.9489 nmole/L 468 Avg. Size (b.p.) 28.61 %CV

Sample Peak Width (sec): 50 Sample Min Peak Height: 25 Sample Baseline V to V?: Y Sample Baseline V to V pts: 3
Sample Filter: Binomial # of Pts for Filter: 3 Sample Start Region (min): 0 Sample End Region (min): 50
Manual Baseline Start (min): 10 Manual Baseline End (min): 48
Marker Peak Width (sec): 5 Marker Min Peak Height: 200 Marker Baseline V to V?: Y Marker Baseline V to V pts: 3
Lower Marker Selection: First Peak > 200 RFU Upper Marker Selection: Last Peak > 200 RFU
Ladder Size (bp): 1, 100, 200, 300, 400, 500, 600, 700, 800, 900, 1000, 1200, 1500, 2000, 3000, 6000
Quantification Using: Ladder Final Concentration (ng/uL): 0.0830 Dilution Factor: 12.0

 Copyright 2015 Advanced Analytical Technologies, Inc.

Sample: 103613-001-133
Well Location: E11
Created: Friday, June 21, 2019 4:25:48 PM

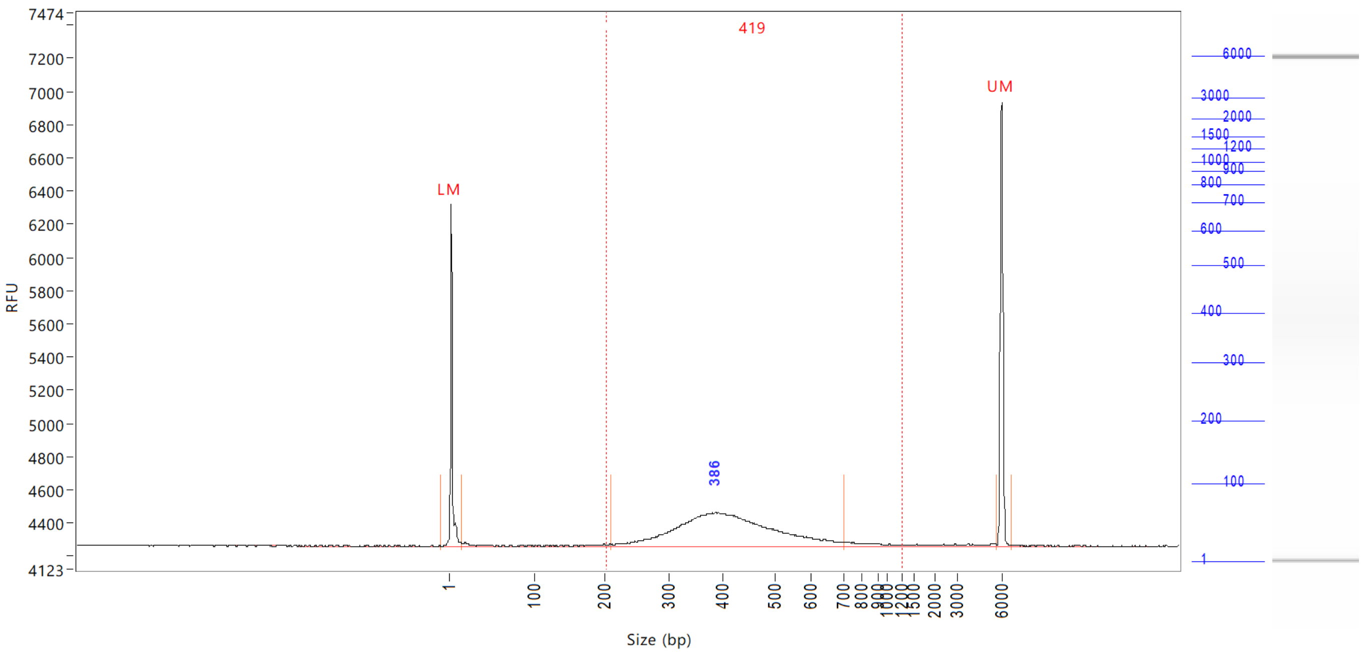

Peak	Size (bp)	Conc. (ng/uL)	From (bp)	To (bp)	RFU
1	1 (LM)	0.0103	0	15	2058
2	386	0.3297	209	703	201
3	6000 (UM)	0.0076	5670	6657	2676

TIC:	0.3297	ng/uL
TIM:	1.3297	nmole/L
Total Conc.:	0.3558	ng/uL

Smear Analysis	200 bp to 1200 bp	0.3394 ng/ul	95.4 %Total	1.3331 nmole/L	419 Avg. Size (b.p.)	28.11 %CV

Sample Peak Width (sec): 50   Sample Min Peak Height: 25   Sample Baseline V to V?: Y   Sample Baseline V to V pts: 3
Sample Filter: Binomial   # of Pts for Filter: 3   Sample Start Region (min): 0   Sample End Region (min): 50
Manual Baseline Start (min): 10   Manual Baseline End (min): 48
Marker Peak Width (sec): 5   Marker Min Peak Height: 200   Marker Baseline V to V?: Y   Marker Baseline V to V pts: 3
Lower Marker Selection: First Peak > 200 RFU   Upper Marker Selection: Last Peak > 200 RFU
Ladder Size (bp): 1, 100, 200, 300, 400, 500, 600, 700, 800, 900, 1000, 1200, 1500, 2000, 3000, 6000
Quantification Using: Ladder   Final Concentration (ng/uL): 0.0830   Dilution Factor: 12.0

Copyright 2015 Advanced Analytical Technologies, Inc.

Sample: 103613-001-134
Well Location: F11
Created: Friday, June 21, 2019 4:25:48 PM

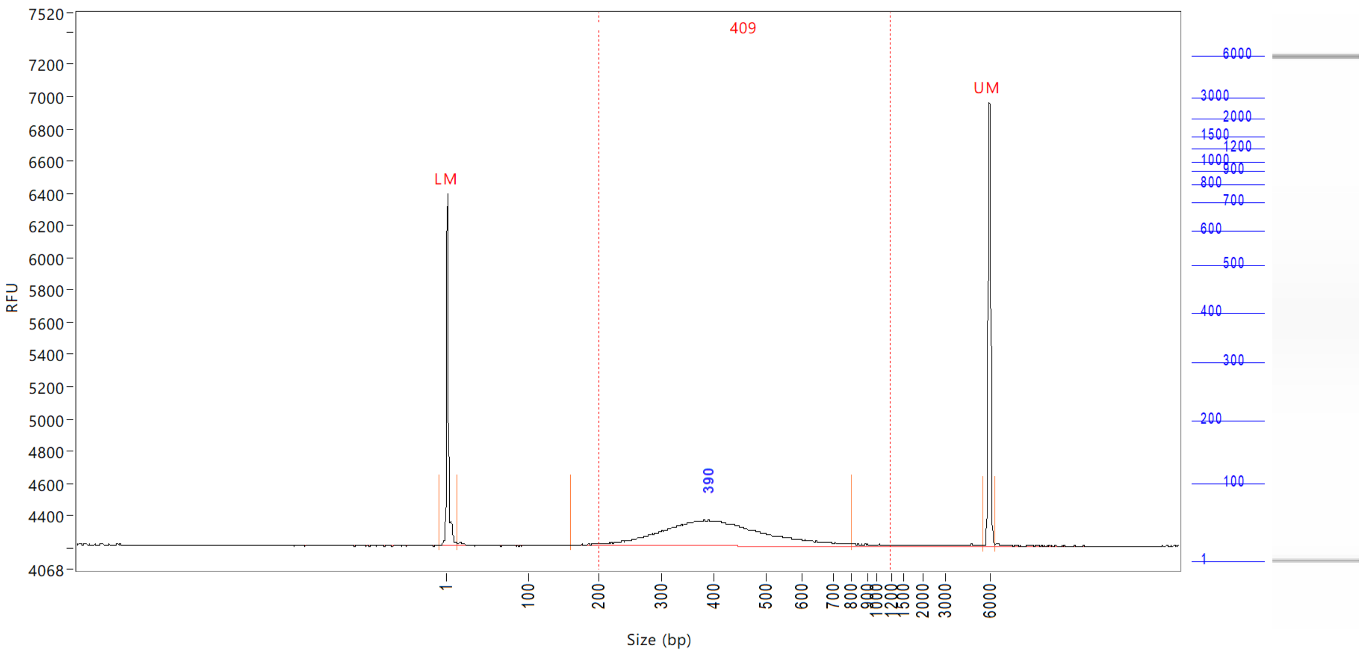

Peak	Size (bp)	Conc. (ng/uL)	From (bp)	To (bp)	RFU
1	1 (LM)	0.0103	0	14	2180
2	390	0.2804	160	801	159
3	6000 (UM)	0.0075	5619	6429	2757
	TIC:	0.2804	ng/uL		
	TIM:	1.1563	nmole/L		
	Total Conc.:	0.2981	ng/uL		

Smear Analysis 200 bp to 1200 bp 0.2804 ng/ul 94.1 %Total 1.1290 nmole/L 409 Avg. Size (b.p.) 29.62 %CV

Sample Peak Width (sec): 50 Sample Min Peak Height: 25 Sample Baseline V to V?: Y Sample Baseline V to V pts: 3
Sample Filter: Binomial # of Pts for Filter: 3 Sample Start Region (min): 0 Sample End Region (min): 50
Manual Baseline Start (min): 10 Manual Baseline End (min): 48
Marker Peak Width (sec): 5 Marker Min Peak Height: 200 Marker Baseline V to V?: Y Marker Baseline V to V pts: 3
Lower Marker Selection: First Peak > 200 RFU Upper Marker Selection: Last Peak > 200 RFU
Ladder Size (bp): 1, 100, 200, 300, 400, 500, 600, 700, 800, 900, 1000, 1200, 1500, 2000, 3000, 6000
Quantification Using: Ladder Final Concentration (ng/uL): 0.0830 Dilution Factor: 12.0

 Copyright 2015 Advanced Analytical Technologies, Inc.

Sample: 103613-001-135
Well Location: G11
Created: Friday, June 21, 2019 4:25:48 PM

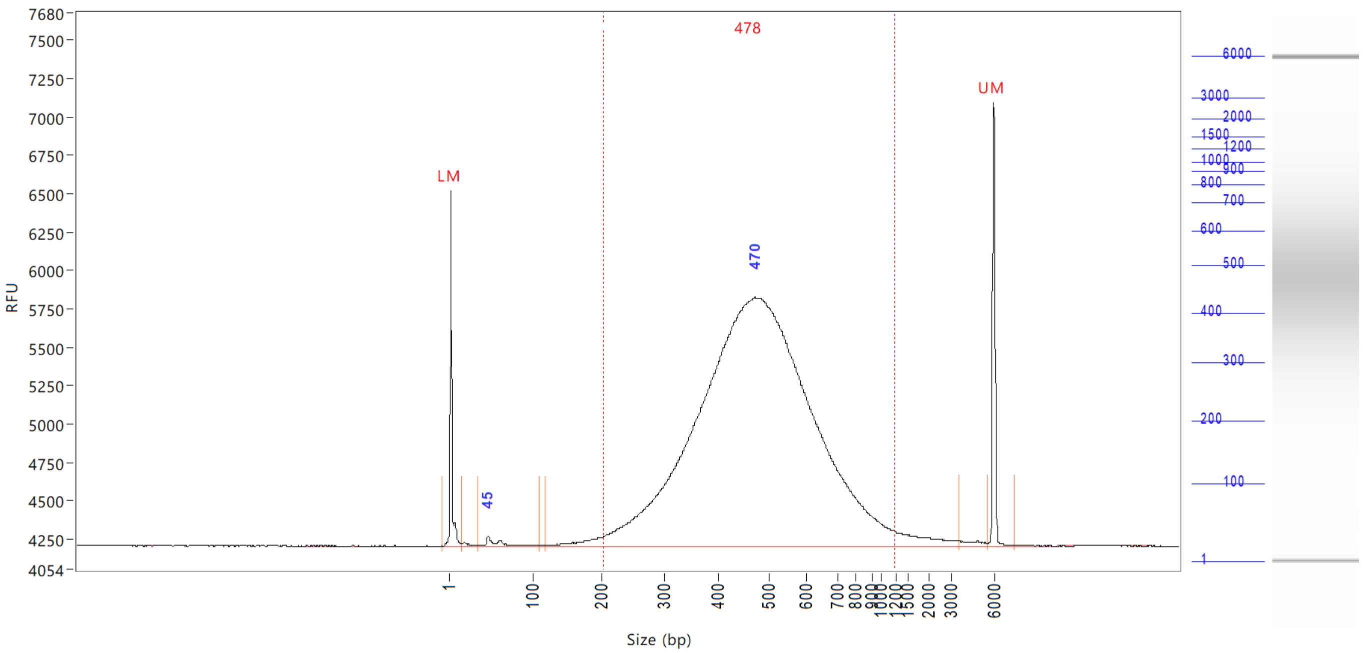

Peak	Size (bp)	Conc. (ng/uL)	From (bp)	To (bp)	RFU
1	1 (LM)	0.0103	0	15	2314
2	45	0.0170	35	108	69
3	470	2.8513	117	3586	1621
4	6000 (UM)	0.0074	5543	7389	2895

TIC:	2.8682	ng/uL
TIM:	9.9611	nmole/L
Total Conc.:	2.8796	ng/uL

Smear Analysis 200 bp to 1200 bp 2.7907 ng/ul 96.9 %Total 9.6042 nmole/L 478 Avg. Size (b.p.) 28.66 %CV

Sample Peak Width (sec): 50 Sample Min Peak Height: 25 Sample Baseline V to V?: Y Sample Baseline V to V pts: 3
Sample Filter: Binomial # of Pts for Filter: 3 Sample Start Region (min): 0 Sample End Region (min): 50
Manual Baseline Start (min): 10 Manual Baseline End (min): 48
Marker Peak Width (sec): 5 Marker Min Peak Height: 200 Marker Baseline V to V?: Y Marker Baseline V to V pts: 3
Lower Marker Selection: First Peak > 200 RFU Upper Marker Selection: Last Peak > 200 RFU
Ladder Size (bp): 1, 100, 200, 300, 400, 500, 600, 700, 800, 900, 1000, 1200, 1500, 2000, 3000, 6000
Quantification Using: Ladder Final Concentration (ng/uL): 0.0830 Dilution Factor: 12.0

 Copyright 2015 Advanced Analytical Technologies, Inc.

Sample: 103613-001-136
Well Location: H11
Created: Friday, June 21, 2019 4:25:48 PM

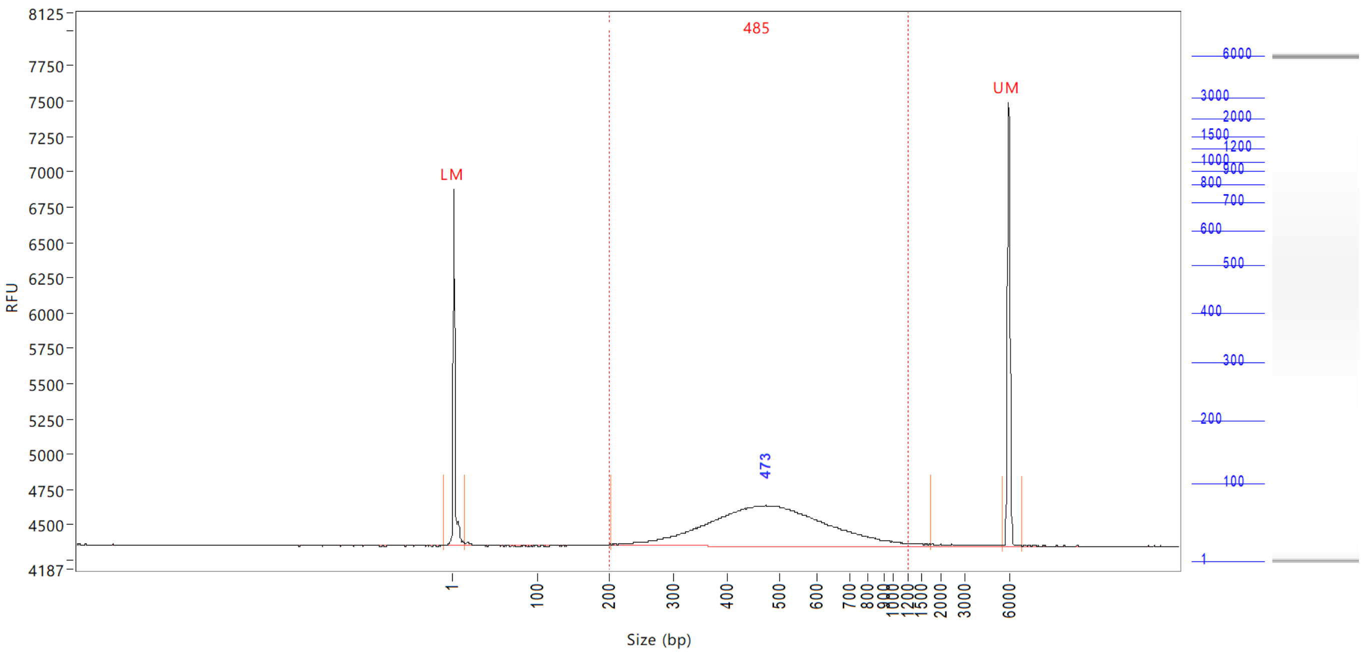

Peak	Size (bp)	Conc. (ng/uL)	From (bp)	To (bp)	RFU
1	1 (LM)	0.0103	0	14	2520
2	473	0.4661	203	1760	287
3	6000 (UM)	0.0073	5593	6935	3145

TIC:	0.4661	ng/uL
TIM:	1.5588	nmole/L
Total Conc.:	0.4789	ng/uL

Smear Analysis	200 bp to 1200 bp	0.4631 ng/ul	96.7 %Total	1.5713 nmole/L	485 Avg. Size (b.p.)	28.83 %CV

Sample Peak Width (sec): 50   Sample Min Peak Height: 25   Sample Baseline V to V?: Y   Sample Baseline V to V pts: 3
Sample Filter: Binomial   # of Pts for Filter: 3   Sample Start Region (min): 0   Sample End Region (min): 50
Manual Baseline Start (min): 10   Manual Baseline End (min): 48
Marker Peak Width (sec): 5   Marker Min Peak Height: 200   Marker Baseline V to V?: Y   Marker Baseline V to V pts: 3
Lower Marker Selection: First Peak > 200 RFU   Upper Marker Selection: Last Peak > 200 RFU
Ladder Size (bp): 1, 100, 200, 300, 400, 500, 600, 700, 800, 900, 1000, 1200, 1500, 2000, 3000, 6000
Quantification Using: Ladder   Final Concentration (ng/uL): 0.0830   Dilution Factor: 12.0

Copyright 2015 Advanced Analytical Technologies, Inc.

Sample: 103613-001-137
Well Location: A12
Created: Friday, June 21, 2019 4:25:48 PM

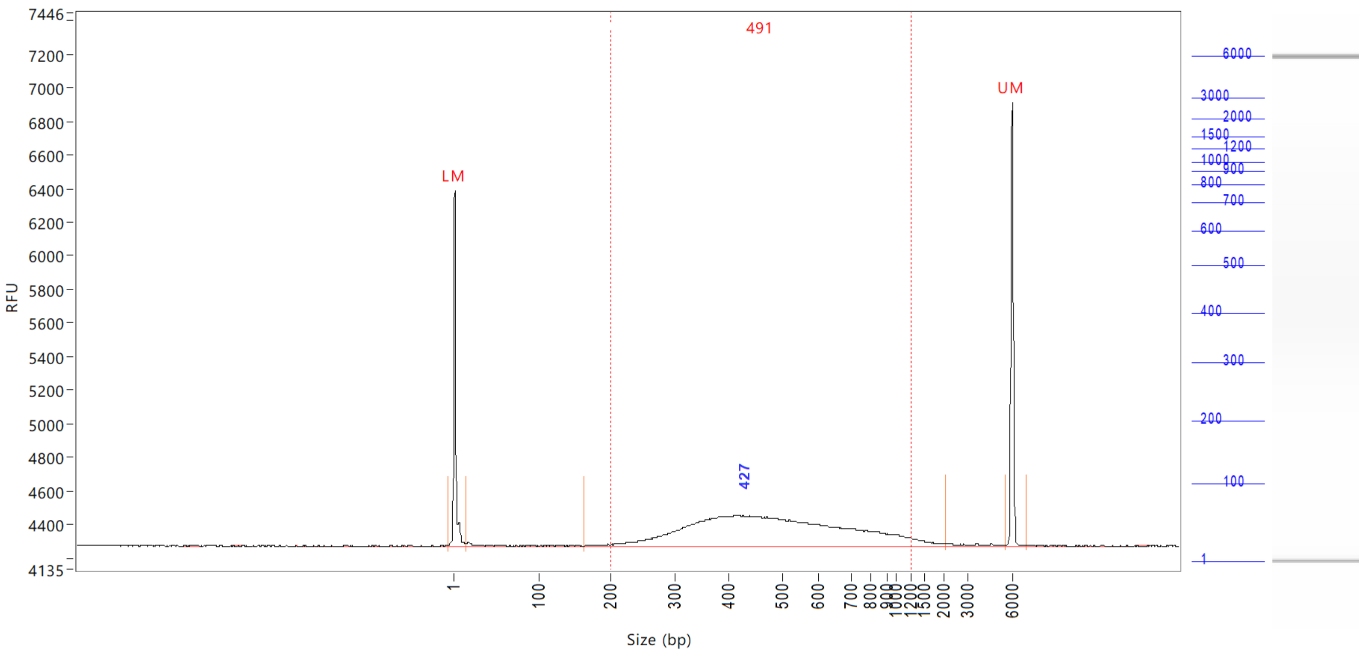

Peak	Size (bp)	Conc. (ng/uL)	From (bp)	To (bp)	RFU
1	1 (LM)	0.0103	0	15	2121
2	427	0.4689	162	2051	183
3	6000 (UM)	0.0071	5543	6960	2644
	TIC:	0.4689	ng/uL		
	TIM:	1.5159	nmole/L		
	Total Conc.:	0.4850	ng/uL		

Smear Analysis 200 bp to 1200 bp 0.4555 ng/ul 93.9 %Total 1.5279 nmole/L 491 Avg. Size (b.p.) 36.52 %CV

Sample Peak Width (sec): 50 Sample Min Peak Height: 25 Sample Baseline V to V?: Y Sample Baseline V to V pts: 3
Sample Filter: Binomial # of Pts for Filter: 3 Sample Start Region (min): 0 Sample End Region (min): 50
Manual Baseline Start (min): 10 Manual Baseline End (min): 48
Marker Peak Width (sec): 5 Marker Min Peak Height: 200 Marker Baseline V to V?: Y Marker Baseline V to V pts: 3
Lower Marker Selection: First Peak > 200 RFU Upper Marker Selection: Last Peak > 200 RFU
Ladder Size (bp): 1, 100, 200, 300, 400, 500, 600, 700, 800, 900, 1000, 1200, 1500, 2000, 3000, 6000
Quantification Using: Ladder Final Concentration (ng/uL): 0.0830 Dilution Factor: 12.0

 Copyright 2015 Advanced Analytical Technologies, Inc.

Sample: 103613-001-138
Well Location: B12
Created: Friday, June 21, 2019 4:25:48 PM

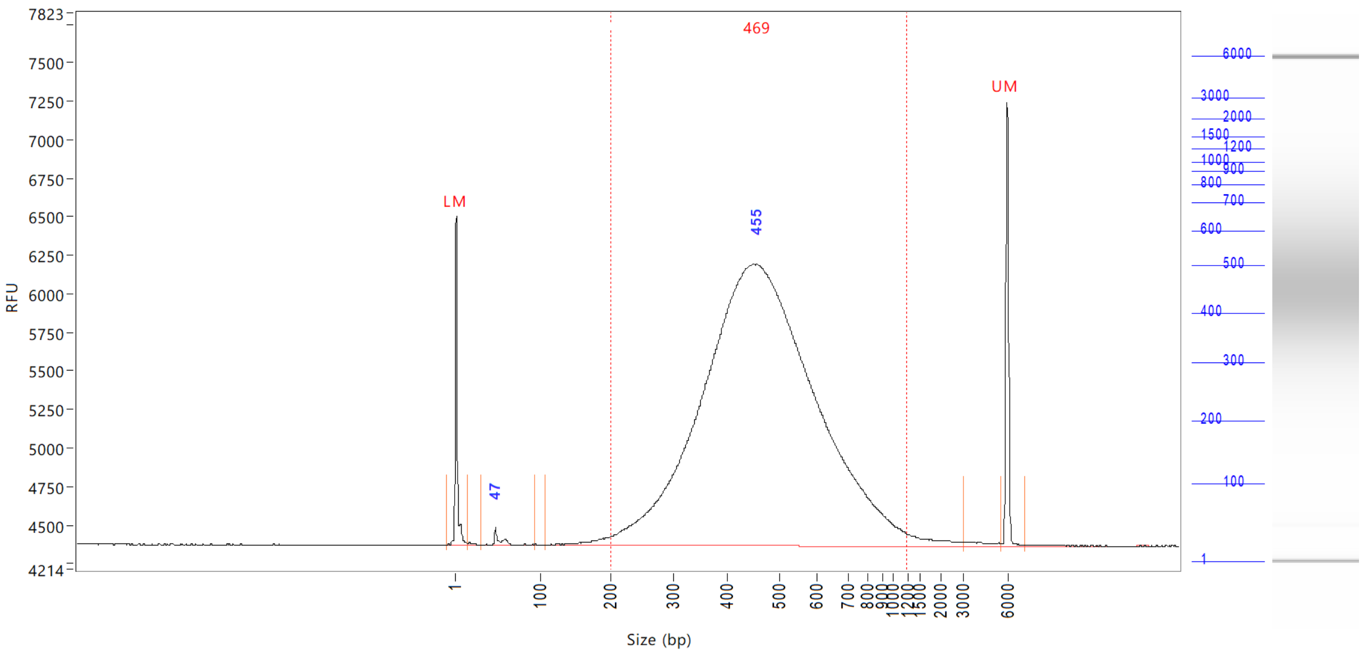

Peak	Size (bp)	Conc. (ng/uL)	From (bp)	To (bp)	RFU
1	1 (LM)	0.0103	0	15	2131
2	47	0.0162	31	94	122
3	455	3.4042	106	3001	1827
4	6000 (UM)	0.0081	5619	7238	2883

TIC:	3.4204	ng/uL
TIM:	12.2271	nmole/L
Total Conc.:	3.4326	ng/uL

Smear Analysis 200 bp to 1200 bp 3.3532 ng/ul 97.7 %Total 11.7588 nmole/L 469 Avg. Size (b.p.) 28.33 %CV

Sample Peak Width (sec): 50 Sample Min Peak Height: 25 Sample Baseline V to V?: Y Sample Baseline V to V pts: 3
Sample Filter: Binomial # of Pts for Filter: 3 Sample Start Region (min): 0 Sample End Region (min): 50
Manual Baseline Start (min): 10 Manual Baseline End (min): 48
Marker Peak Width (sec): 5 Marker Min Peak Height: 200 Marker Baseline V to V?: Y Marker Baseline V to V pts: 3
Lower Marker Selection: First Peak > 200 RFU Upper Marker Selection: Last Peak > 200 RFU
Ladder Size (bp): 1, 100, 200, 300, 400, 500, 600, 700, 800, 900, 1000, 1200, 1500, 2000, 3000, 6000
Quantification Using: Ladder Final Concentration (ng/uL): 0.0830 Dilution Factor: 12.0

 Copyright 2015 Advanced Analytical Technologies, Inc.

Sample: ladder
Well Location: G6
Created: Friday, June 21, 2019 4:25:48 PM
Fit Type: Point to Point

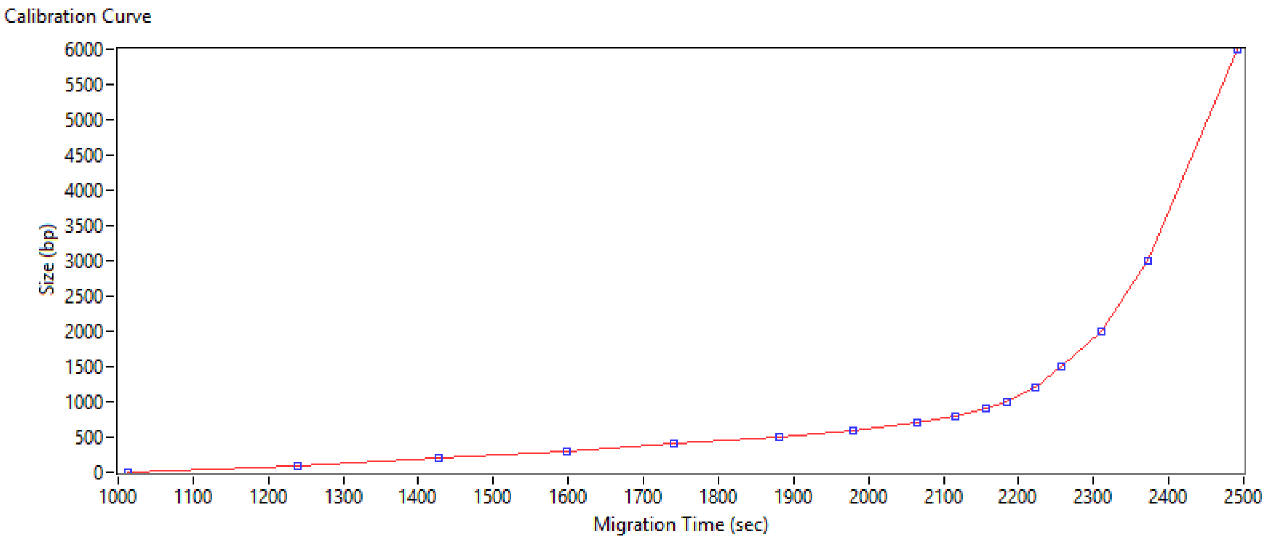

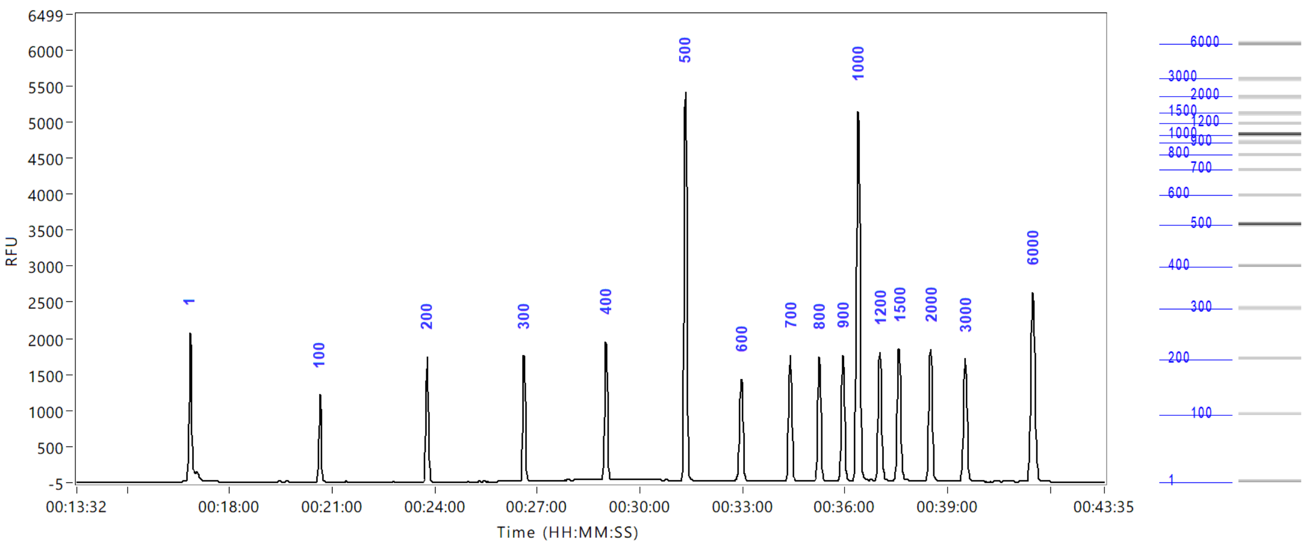

Copyright 2015 Advanced Analytical Technologies, Inc.

Fragment Analyzer Run Summary:

Filename and Data Path: X:\Lopende Opdrachten\GAII\103613\PrepQC_103613-NGS_HS 13-42-08\2019 06 21 13H 42M.raw
Created: Friday, June 21, 2019 2:11:36 PM
# of Capillaries: 96
Array Serial #: 112118-01SFS
Effect Length: 33 cm
Array Usage Count: 227
FA Version #: 1.2.0.11
Device Serial #: 3003

METHOD INFORMATION
Method Name: DNF-474-33 - HS NGS Fragment 1-6000bp.mthds
Gel Prime: No
Full Conditioning: Yes
Gel Prime to Buffer: No
Gel Selection: Gel 1
Perform Prerun: 6.0 kV, 30 sec.
Rinse: No
Marker 1: No
Rinse: Tray: 3, Row: A, # Dips: 1
Sample Injection: 5.0 kV, 30 sec.
Separation: 6.0 kV, 50.0 min.
Tray Name: PrepQC_103613-NGS_HS

Analysis Mode: NGS

NOTES

Copyright 2015 Advanced Analytical Technologies, Inc.

Gel Image

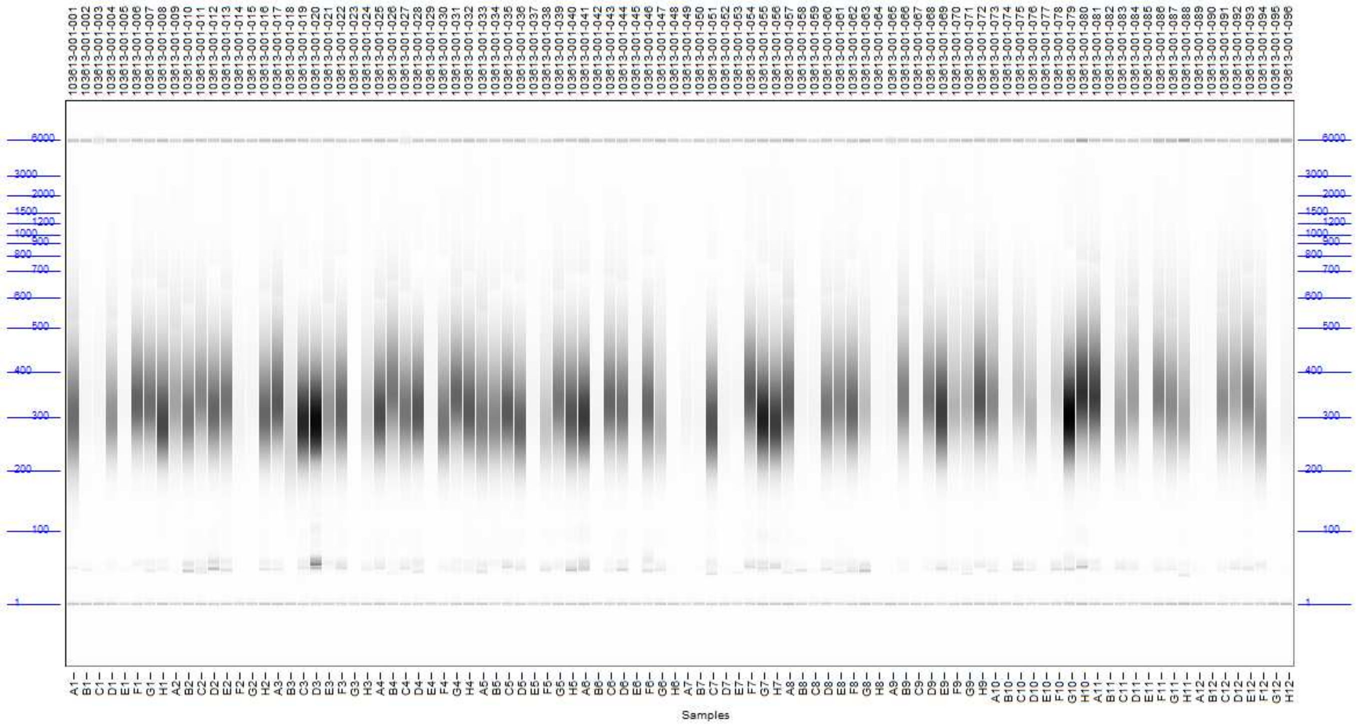

Copyright 2015 Advanced Analytical Technologies, Inc.

Filename and Data Path: X:\Lopende Opdrachten\GAII\103613\PrepQC_103613-NGS_HS 13-42-08\2019 06 21 13H 42M.raw

A1: 103613-001-001

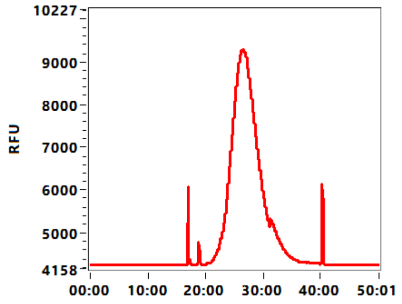

B1: 103613-001-002

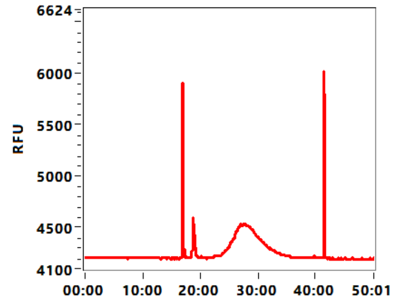

C1: 103613-001-003

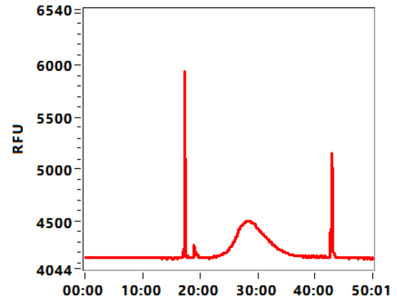

D1: 103613-001-004

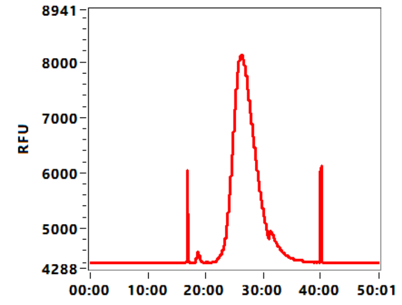

E1: 103613-001-005

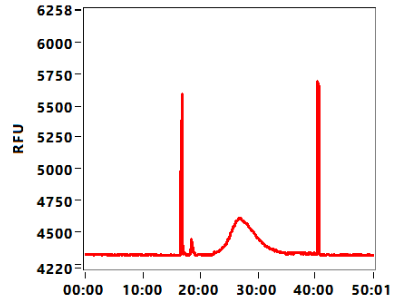

F1: 103613-001-006

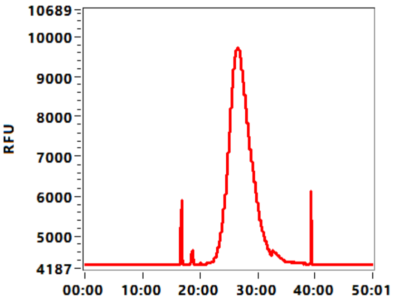

G1: 103613-001-007

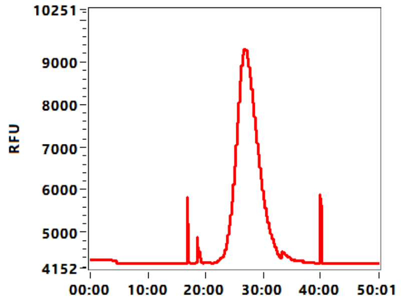

H1: 103613-001-008

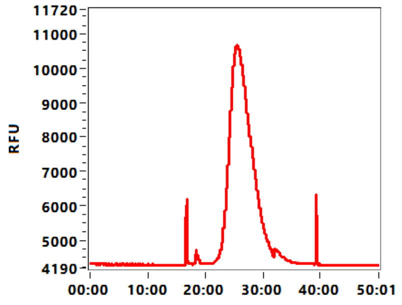

A2: 103613-001-009

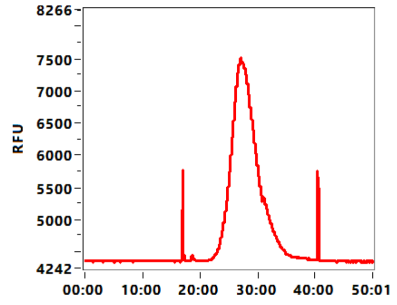

B2: 103613-001-010

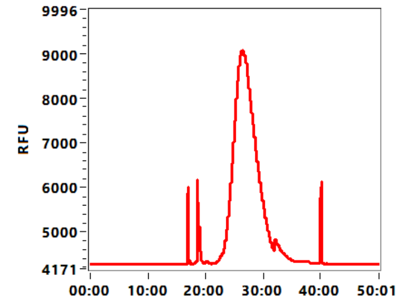

C2: 103613-001-011

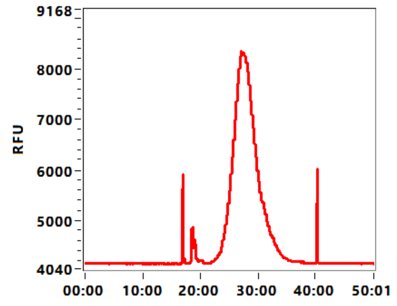

D2: 103613-001-012

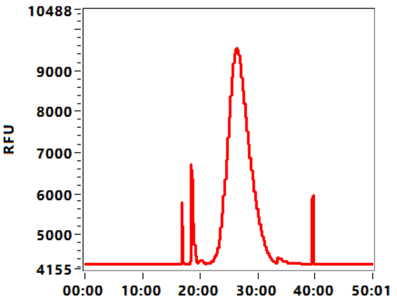

Copyright 2015 Advanced Analytical Technologies, Inc.

Filename and Data Path: X:\Lopende Opdrachten\GAII\103613\PrepQC_103613-NGS_HS 13-42-08\2019 06 21 13H 42M.raw

E2: 103613-001-013

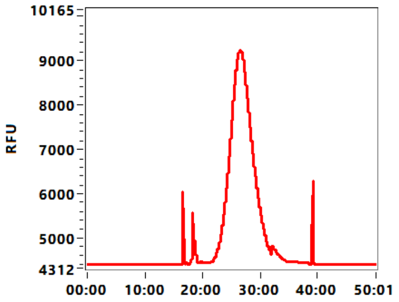

F2: 103613-001-014

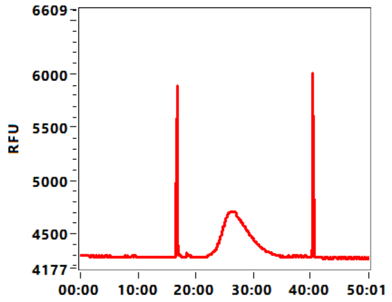

G2: 103613-001-015

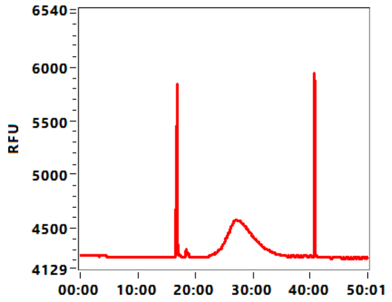

H2: 103613-001-016

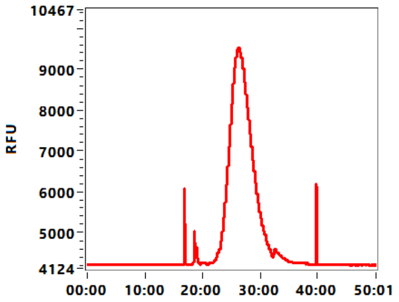

A3: 103613-001-017

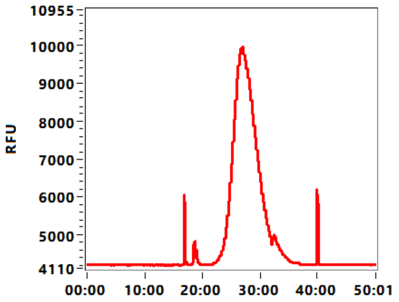

B3: 103613-001-018

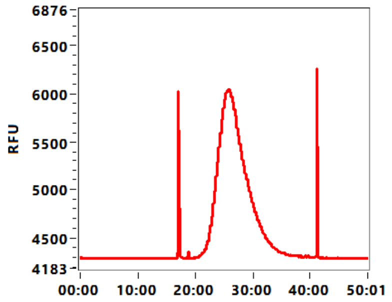

C3: 103613-001-019

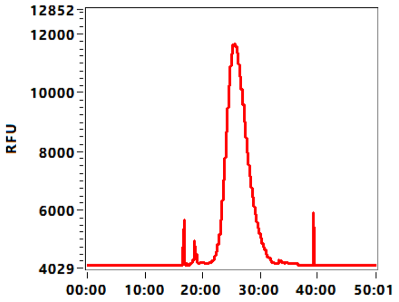

D3: 103613-001-020

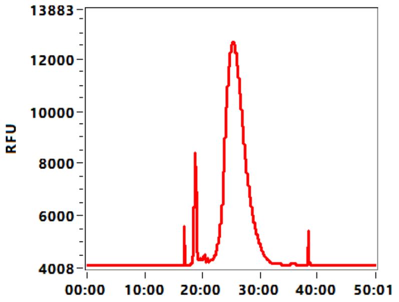

E3: 103613-001-021

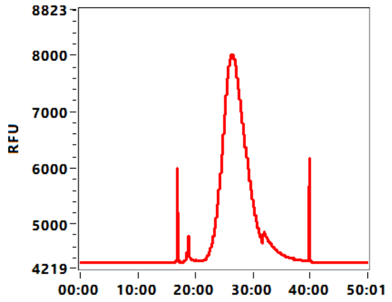

F3: 103613-001-022

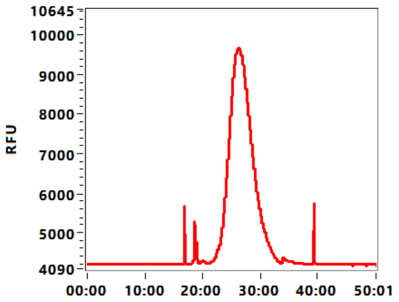

G3: 103613-001-023

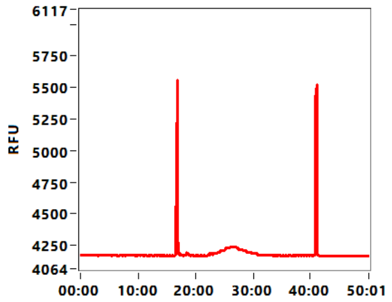

H3: 103613-001-024

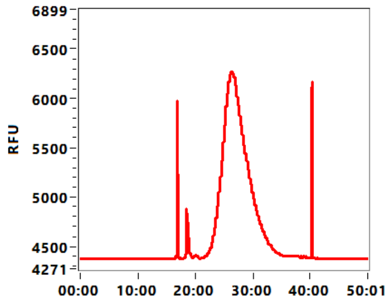

Copyright 2015 Advanced Analytical Technologies, Inc.

Filename and Data Path: X:\Lopende Opdrachten\GAII\103613\PrepQC_103613-NGS_HS 13-42-08\2019 06 21 13H 42M.raw

A4: 103613-001-025

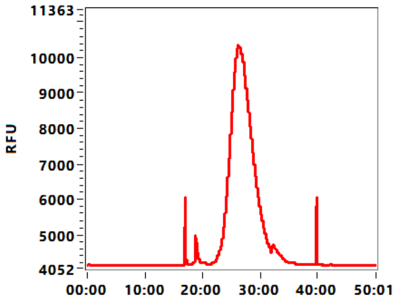

B4: 103613-001-026

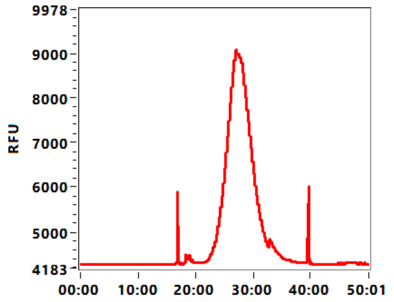

C4: 103613-001-027

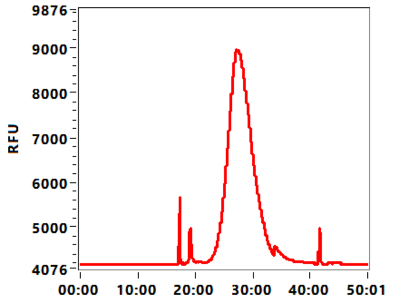

D4: 103613-001-028

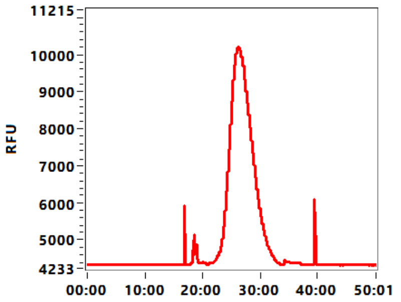

E4: 103613-001-029

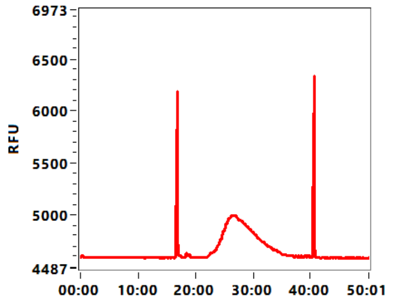

F4: 103613-001-030

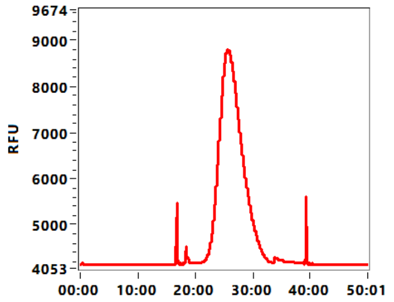

G4: 103613-001-031

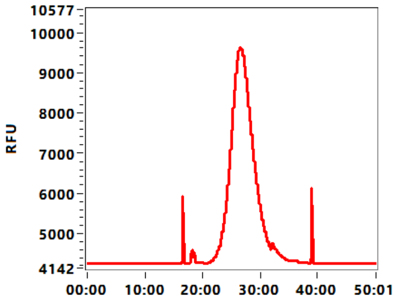

H4: 103613-001-032

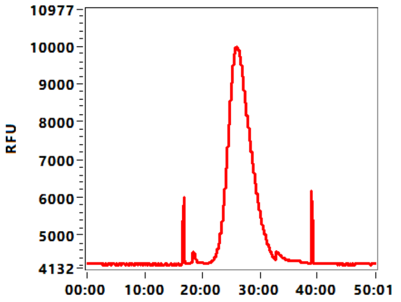

A5: 103613-001-033

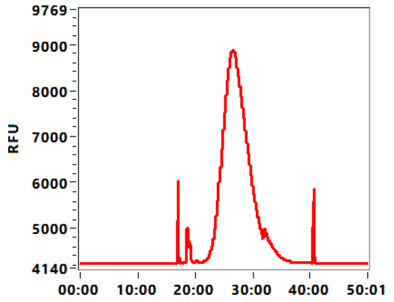

B5: 103613-001-034

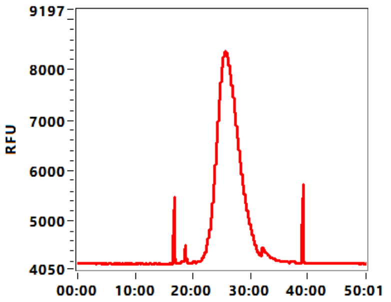

C5: 103613-001-035

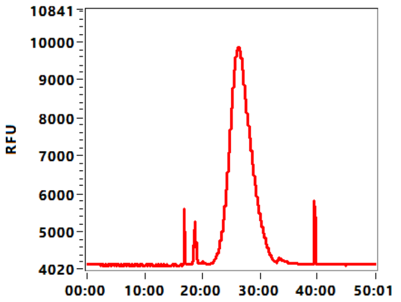

D5: 103613-001-036

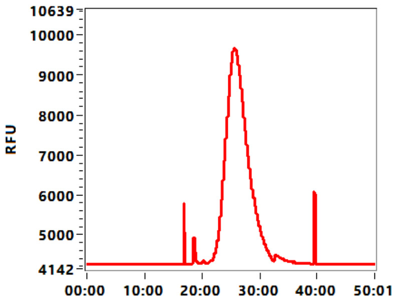

Copyright 2015 Advanced Analytical Technologies, Inc.

Filename and Data Path: X:\Lopende Opdrachten\GAII\103613\PrepQC_103613-NGS_HS 13-42-08\2019 06 21 13H 42M.raw

E5: 103613-001-037

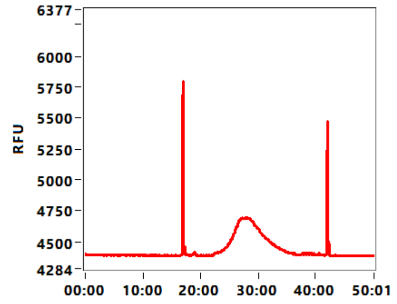

F5: 103613-001-038

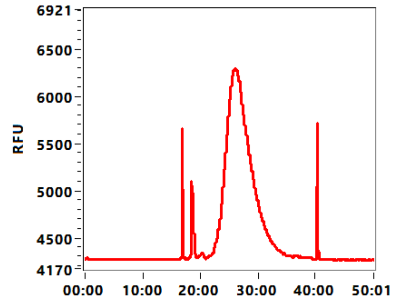

G5: 103613-001-039

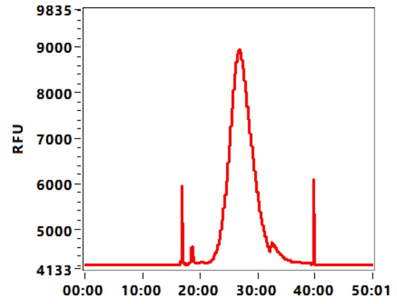

H5: 103613-001-040

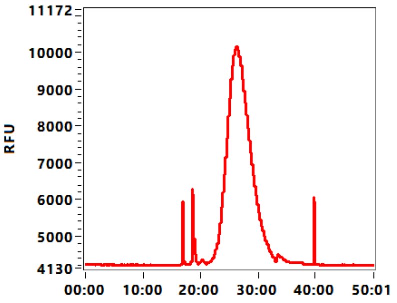

A6: 103613-001-041

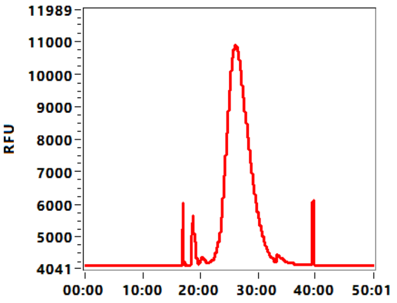

B6: 103613-001-042

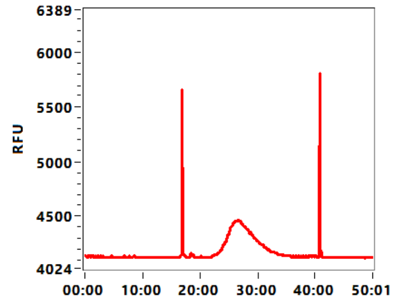

C6: 103613-001-043

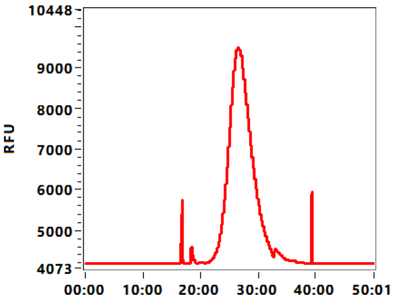

D6: 103613-001-044

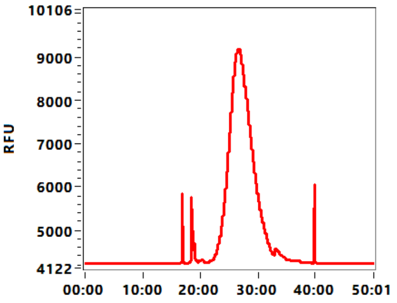

E6: 103613-001-045

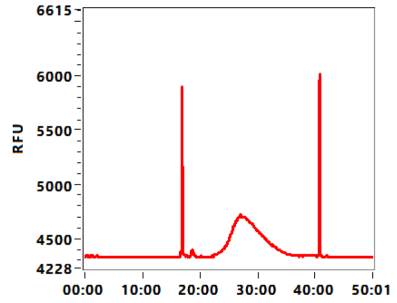

F6: 103613-001-046

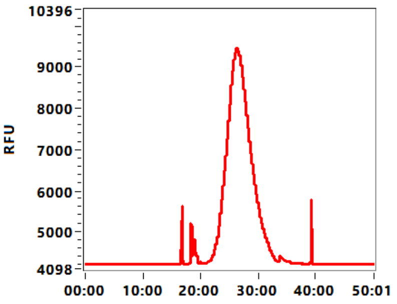

G6: 103613-001-047

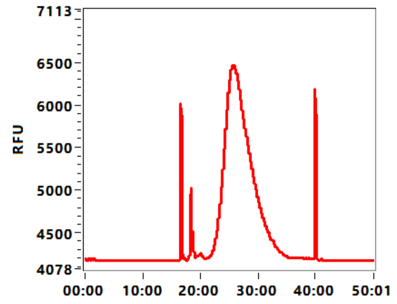

H6: 103613-001-048

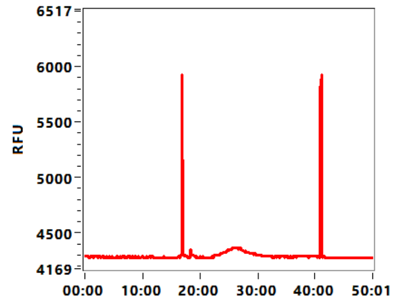

Copyright 2015 Advanced Analytical Technologies, Inc.

Filename and Data Path: X:\Lopende Opdrachten\GAII\103613\PrepQC_103613-NGS_HS 13-42-08\2019 06 21 13H 42M.raw

A7: 103613-001-049

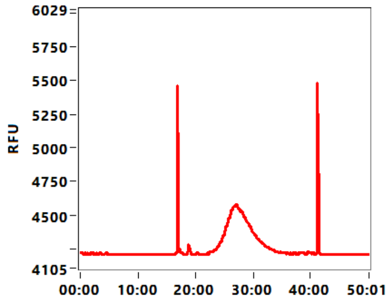

B7: 103613-001-050

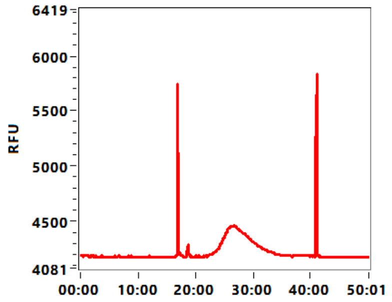

C7: 103613-001-051

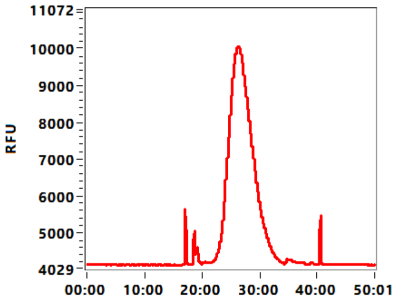

D7: 103613-001-052

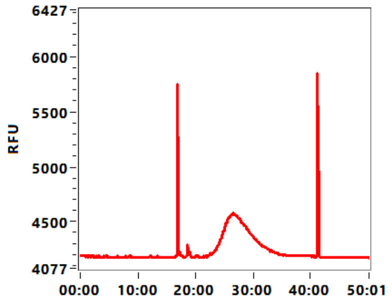

E7: 103613-001-053

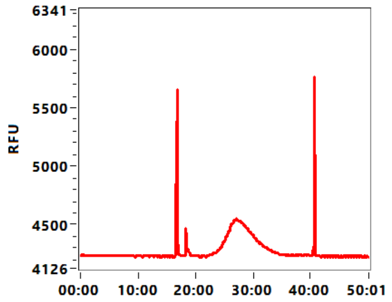

F7: 103613-001-054

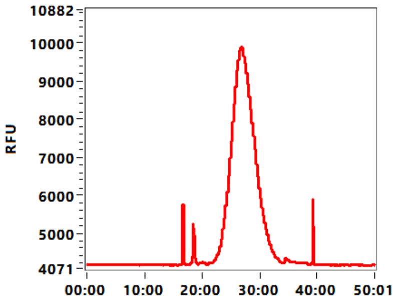

G7: 103613-001-055

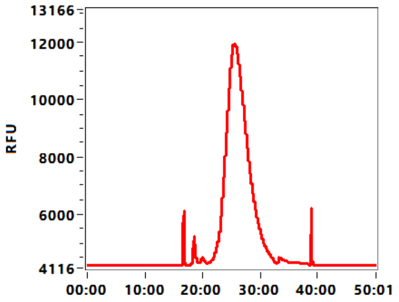

H7: 103613-001-056

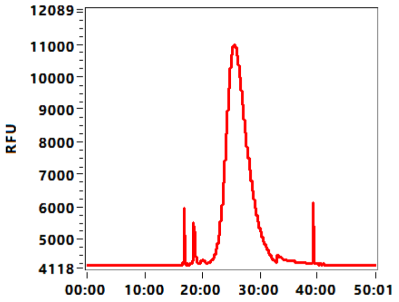

A8: 103613-001-057

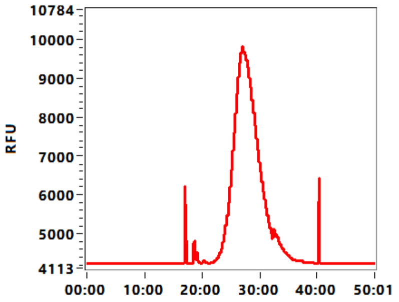

B8: 103613-001-058

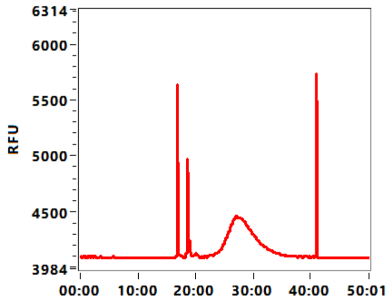

C8: 103613-001-059

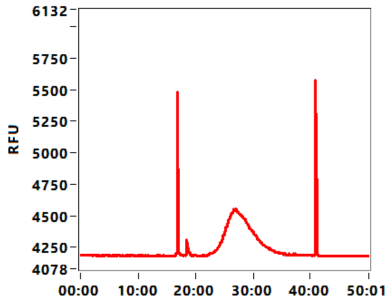

D8: 103613-001-060

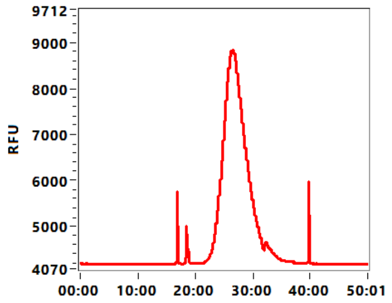

 Copyright 2015 Advanced Analytical Technologies, Inc.

Filename and Data Path: X:\Lopende Opdrachten\GAII\103613\PrepQC_103613-NGS_HS 13-42-08\2019 06 21 13H 42M.raw

E8: 103613-001-061

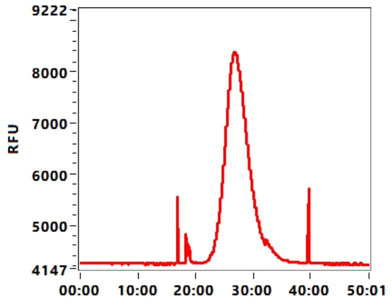

F8: 103613-001-062

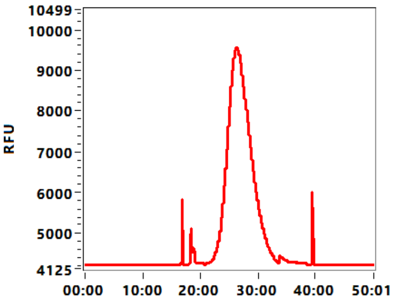

G8: 103613-001-063

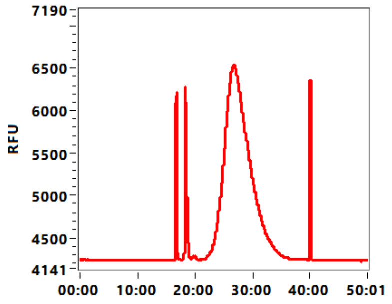

H8: 103613-001-064

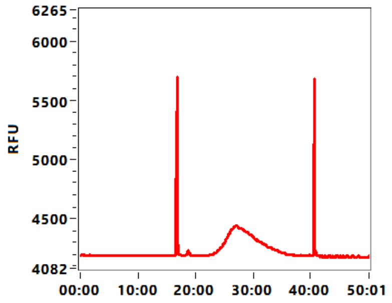

A9: 103613-001-065

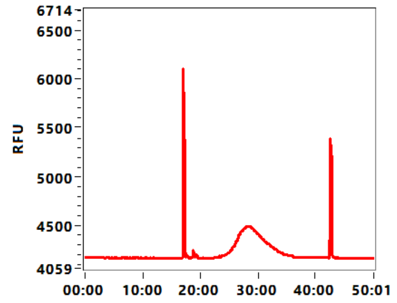

B9: 103613-001-066

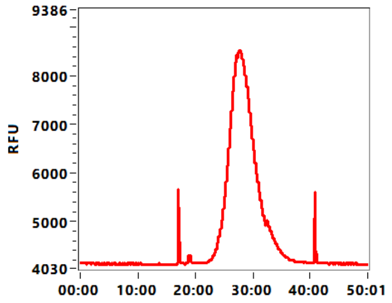

C9: 103613-001-067

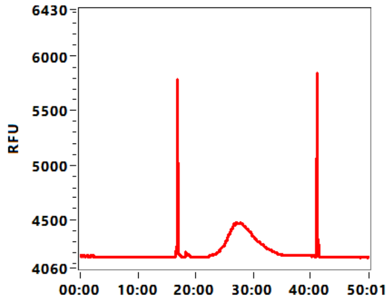

D9: 103613-001-068

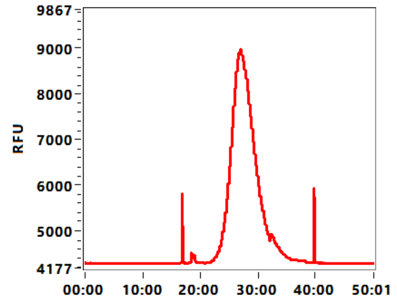

E9: 103613-001-069

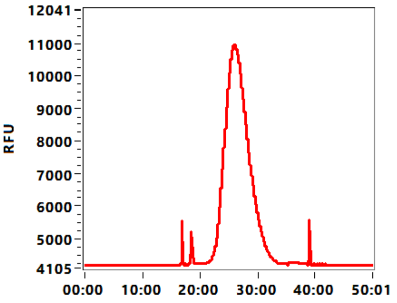

F9: 103613-001-070

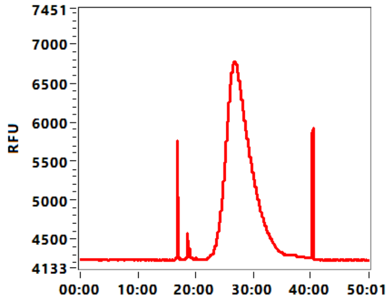

G9: 103613-001-071

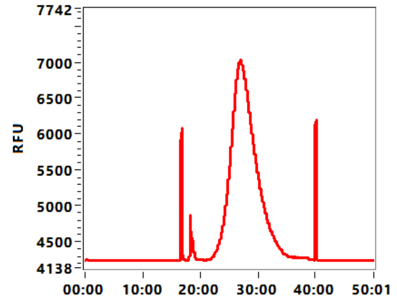

H9: 103613-001-072

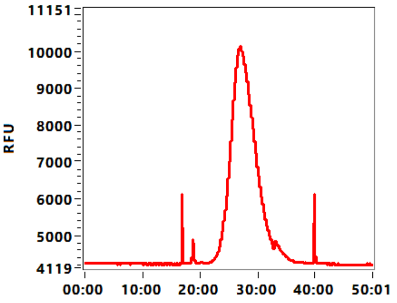

Copyright 2015 Advanced Analytical Technologies, Inc.

Filename and Data Path: X:\Lopende Opdrachten\GAII\103613\PrepQC_103613-NGS_HS 13-42-08\2019 06 21 13H 42M.raw

A10: 103613-001-073

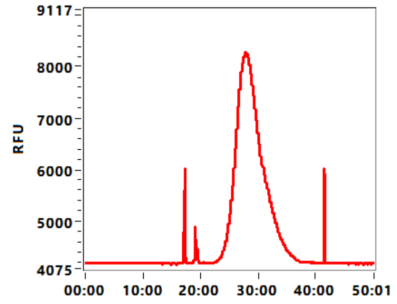

B10: 103613-001-074

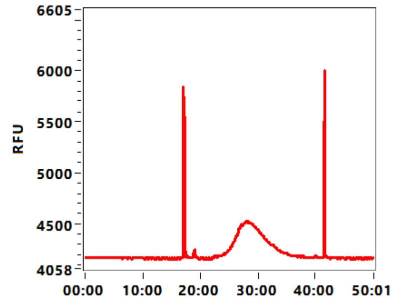

C10: 103613-001-075

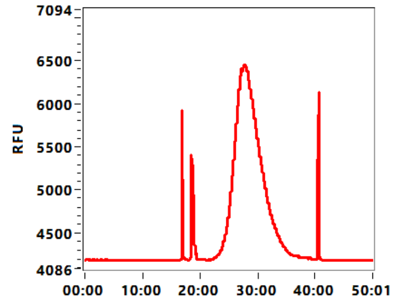

D10: 103613-001-076

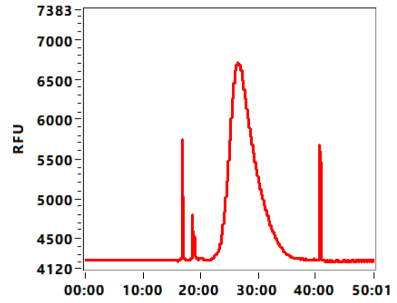

E10: 103613-001-077

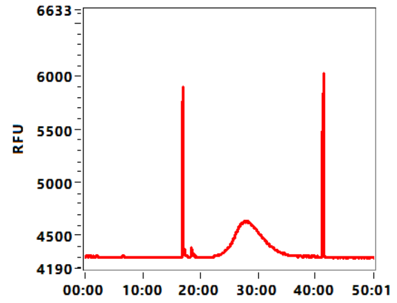

F10: 103613-001-078

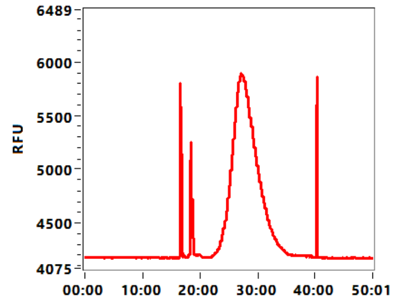

G10: 103613-001-079

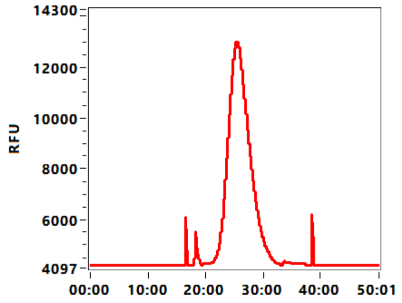

H10: 103613-001-080

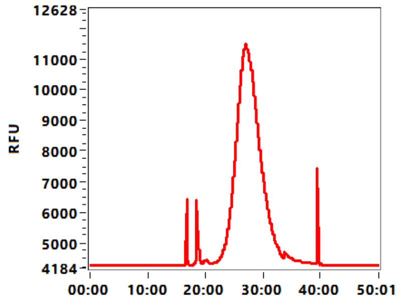

A11: 103613-001-081

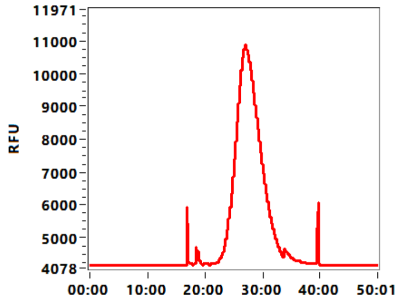

B11: 103613-001-082

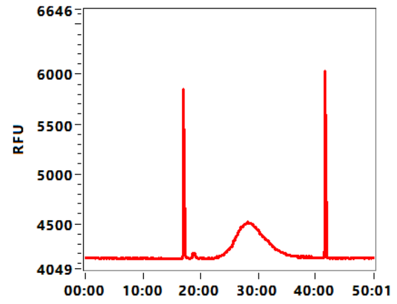

C11: 103613-001-083

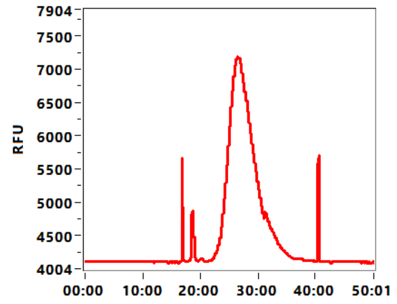

D11: 103613-001-084

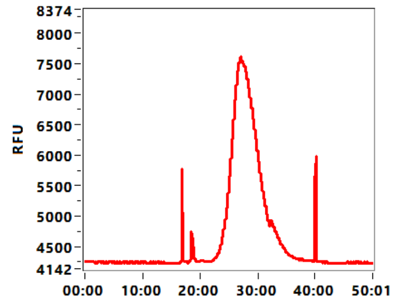

Copyright 2015 Advanced Analytical Technologies, Inc.

Filename and Data Path: X:\Lopende Opdrachten\GAII\103613\PrepQC_103613-NGS_HS 13-42-08\2019 06 21 13H 42M.raw

E11: 103613-001-085

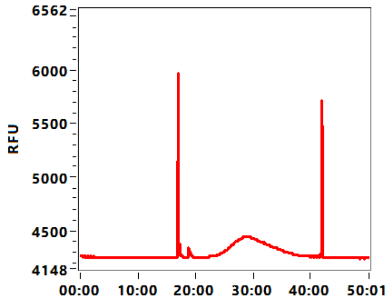

F11: 103613-001-086

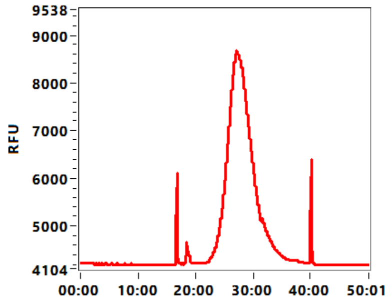

G11: 103613-001-087

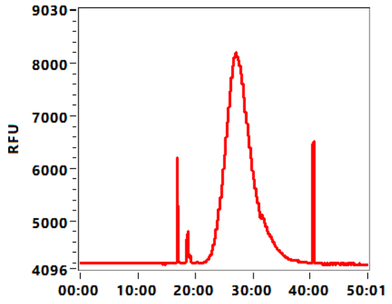

H11: 103613-001-088

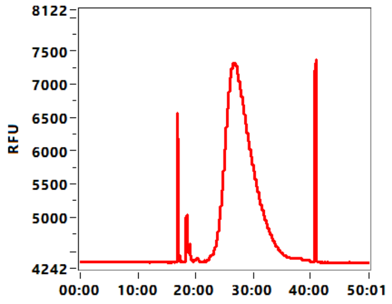

A12: 103613-001-089

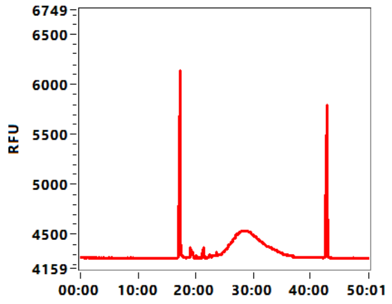

B12: 103613-001-090

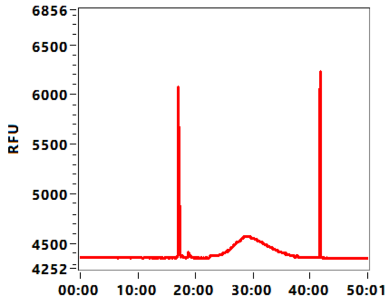

C12: 103613-001-091

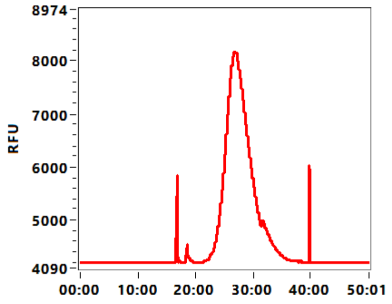

D12: 103613-001-092

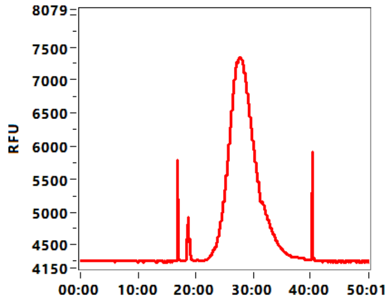

E12: 103613-001-093

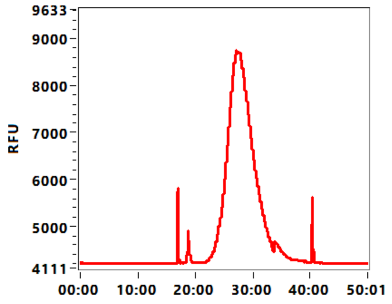

F12: 103613-001-094

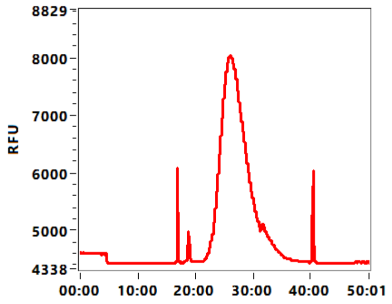

G12: 103613-001-095

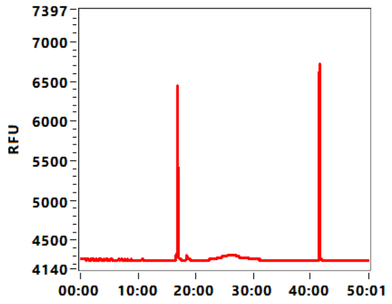

H12: 103613-001-096

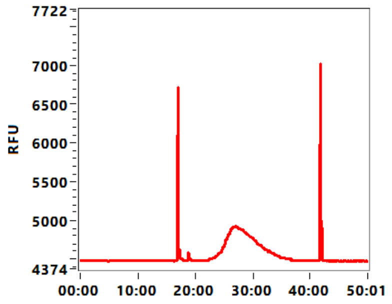

Copyright 2015 Advanced Analytical Technologies, Inc.

Sample: 103613-001-001
Well Location: A1
Created: Friday, June 21, 2019 2:11:36 PM

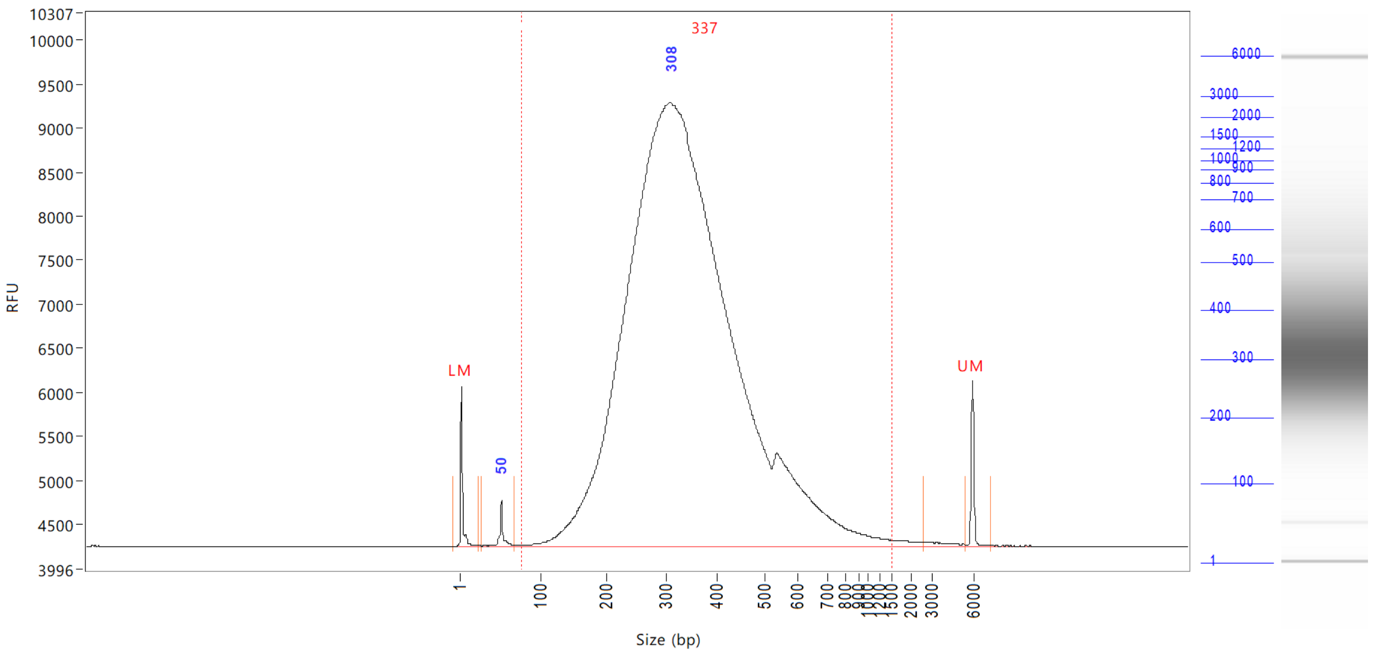

Peak	Size (bp)	Conc. (ng/uL)	From (bp)	To (bp)	RFU
1	1 (LM)	0.0124	0	23	1807
2	50	0.0664	26	68	527
3	308	13.5857	68	2600	5046
4	6000 (UM)	0.0077	5492	7364	1890
	TIC:	13.6522	ng/uL		
	TIM:	67.7187	nmole/L		
	Total Conc.:	13.6763	ng/uL		

Smear Analysis 75 bp to 1500 bp 13.5515 ng/ul 99.1 %Total 66.1277 nmole/L 337 Avg. Size (b.p.) 35.67 %CV

Sample Peak Width (sec): 50 Sample Min Peak Height: 25 Sample Baseline V to V?: Y Sample Baseline V to V pts: 3
Sample Filter: Binomial # of Pts for Filter: 3 Sample Start Region (min): 0 Sample End Region (min): 50
Manual Baseline Start (min): 10 Manual Baseline End (min): 48
Marker Peak Width (sec): 5 Marker Min Peak Height: 200 Marker Baseline V to V?: Y Marker Baseline V to V pts: 3
Lower Marker Selection: First Peak > 200 RFU Upper Marker Selection: Last Peak > 200 RFU
Ladder Size (bp): 1, 100, 200, 300, 400, 500, 600, 700, 800, 900, 1000, 1200, 1500, 2000, 3000, 6000
Quantification Using: Ladder Final Concentration (ng/uL): 0.0830 Dilution Factor: 12.0

Copyright 2015 Advanced Analytical Technologies, Inc.

Sample: 103613-001-002
Well Location: B1
Created: Friday, June 21, 2019 2:11:36 PM

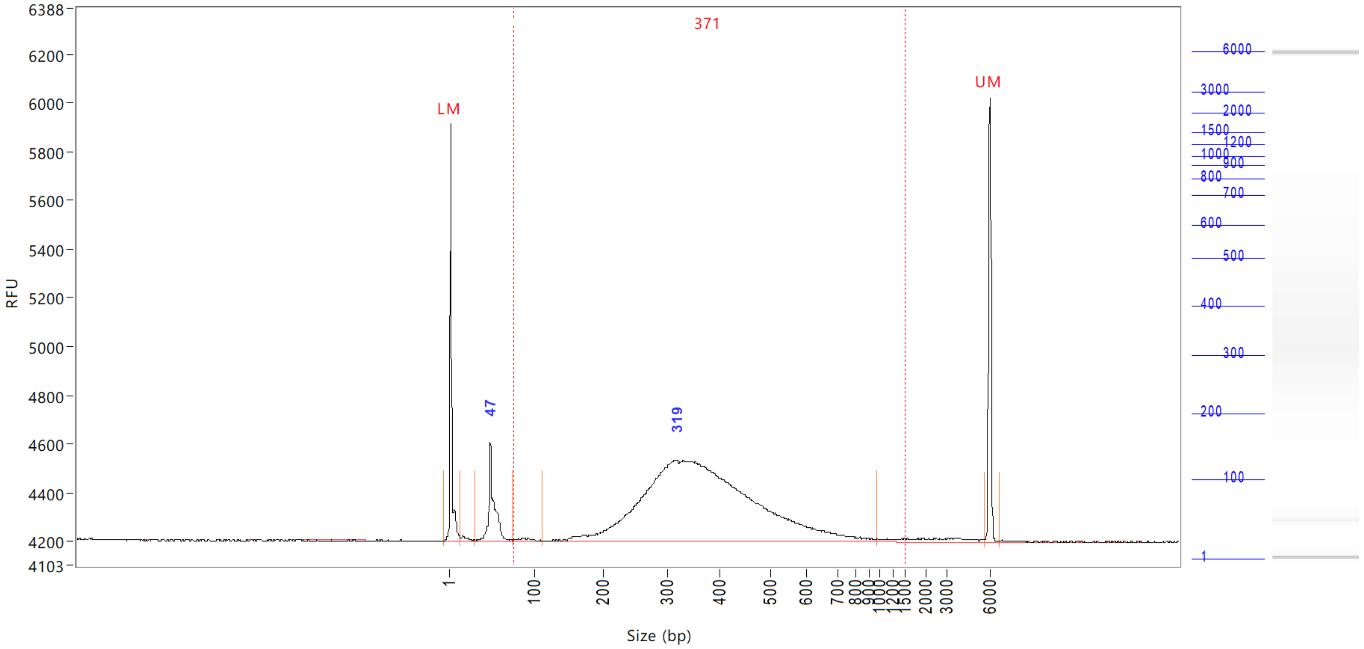

Peak	Size (bp)	Conc. (ng/uL)	From (bp)	To (bp)	RFU
1	1 (LM)	0.0124	0	14	1708
2	47	0.0795	30	75	402
3	319	0.9662	111	980	334
4	6000 (UM)	0.0075	5644	6657	1825

TIC:	1.0457	ng/uL
TIM:	6.9234	nmole/L
Total Conc.:	1.0744	ng/uL

Smear Analysis	75 bp to 1500 bp	0.9757 ng/ul	90.8 %Total	4.3278 nmole/L	371 Avg. Size (b.p.)	34.54 %CV

Sample Peak Width (sec): 50 Sample Min Peak Height: 25 Sample Baseline V to V?: Y Sample Baseline V to V pts: 3
Sample Filter: Binomial # of Pts for Filter: 3 Sample Start Region (min): 0 Sample End Region (min): 50
Manual Baseline Start (min): 10 Manual Baseline End (min): 48
Marker Peak Width (sec): 5 Marker Min Peak Height: 200 Marker Baseline V to V?: Y Marker Baseline V to V pts: 3
Lower Marker Selection: First Peak > 200 RFU Upper Marker Selection: Last Peak > 200 RFU
Ladder Size (bp): 1, 100, 200, 300, 400, 500, 600, 700, 800, 900, 1000, 1200, 1500, 2000, 3000, 6000
Quantification Using: Ladder Final Concentration (ng/uL): 0.0830 Dilution Factor: 12.0

Copyright 2015 Advanced Analytical Technologies, Inc.

Sample: 103613-001-003
Well Location: C1
Created: Friday, June 21, 2019 2:11:36 PM

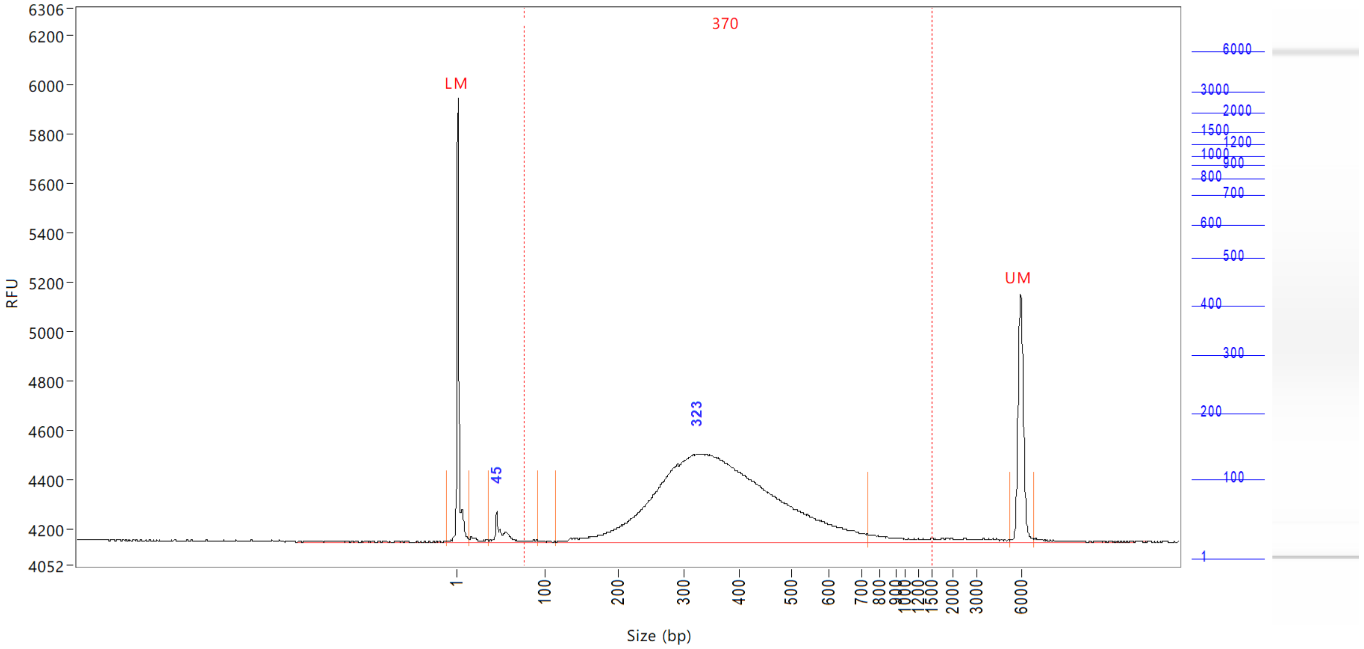

Peak	Size (bp)	Conc. (ng/uL)	From (bp)	To (bp)	RFU
1	1 (LM)	0.0124	0	14	1800
2	45	0.0277	36	92	127
3	323	1.0273	114	736	356
4	6000 (UM)	0.0074	5263	6834	1004

TIC:	1.0550	ng/uL
TIM:	5.5245	nmole/L
Total Conc.:	1.0930	ng/uL

Smear Analysis	75 bp to 1500 bp	1.0475 ng/ul	95.8 %Total	4.6548 nmole/L	370 Avg. Size (b.p.)	35.45 %CV

Sample Peak Width (sec): 50   Sample Min Peak Height: 25   Sample Baseline V to V?: Y   Sample Baseline V to V pts: 3
Sample Filter: Binomial   # of Pts for Filter: 3   Sample Start Region (min): 0   Sample End Region (min): 50
Manual Baseline Start (min): 10   Manual Baseline End (min): 48
Marker Peak Width (sec): 5   Marker Min Peak Height: 200   Marker Baseline V to V?: Y   Marker Baseline V to V pts: 3
Lower Marker Selection: First Peak > 200 RFU   Upper Marker Selection: Last Peak > 200 RFU
Ladder Size (bp): 1, 100, 200, 300, 400, 500, 600, 700, 800, 900, 1000, 1200, 1500, 2000, 3000, 6000
Quantification Using: Ladder   Final Concentration (ng/uL): 0.0830   Dilution Factor: 12.0

 Copyright 2015 Advanced Analytical Technologies, Inc.

Sample: 103613-001-004
Well Location: D1
Created: Friday, June 21, 2019 2:11:36 PM

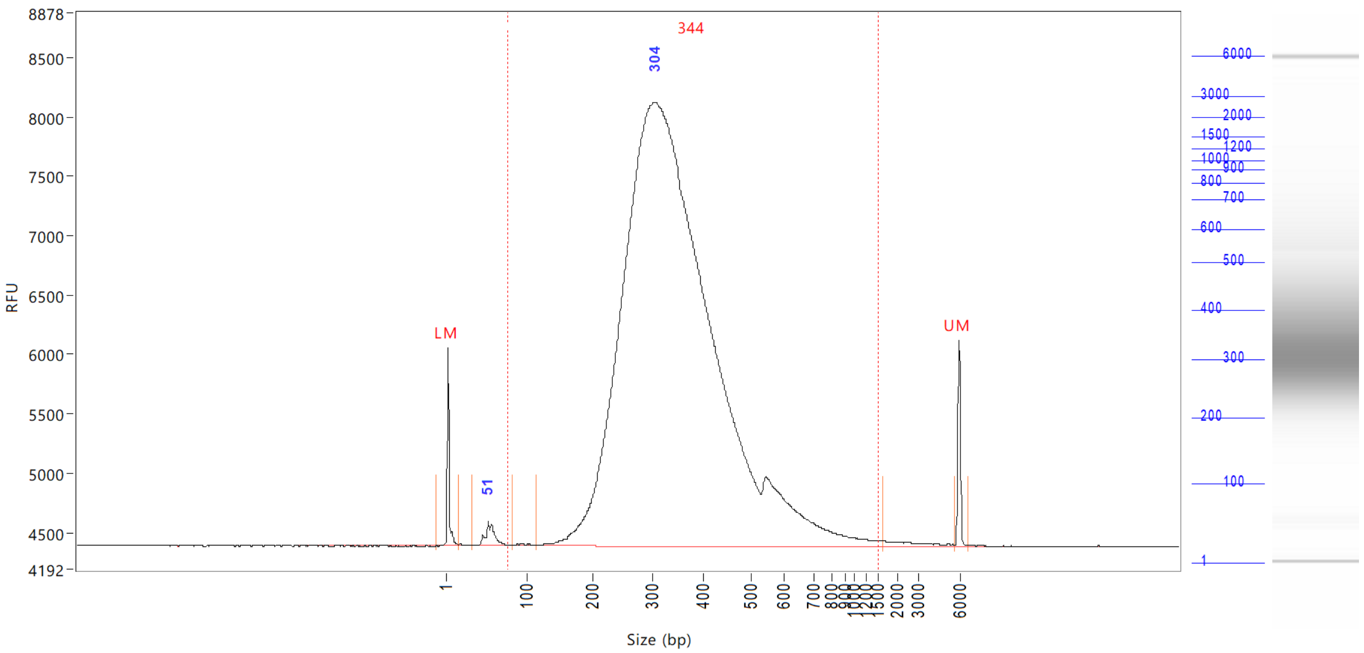

Peak	Size (bp)	Conc. (ng/uL)	From (bp)	To (bp)	RFU
1	1 (LM)	0.0124	0	15	1663
2	51	0.0777	33	82	203
3	304	9.6877	114	1635	3741
4	6000 (UM)	0.0077	5670	6682	1736

TIC:	9.7655	ng/uL
TIM:	48.5540	nmole/L
Total Conc.:	9.8104	ng/uL

Smear Analysis 75 bp to 1500 bp 9.6936 ng/ul 98.8 %Total 46.3589 nmole/L 344 Avg. Size (b.p.) 31.93 %CV

```
Sample Peak Width (sec): 50   Sample Min Peak Height: 25    Sample Baseline V to V?: Y    Sample Baseline V to V pts: 3
Sample Filter: Binomial       # of Pts for Filter: 3        Sample Start Region (min): 0  Sample End Region (min): 50
Manual Baseline Start (min): 10     Manual Baseline End (min): 48
Marker Peak Width (sec): 5    Marker Min Peak Height: 200   Marker Baseline V to V?: Y    Marker Baseline V to V pts: 3
Lower Marker Selection: First Peak > 200 RFU                Upper Marker Selection: Last Peak > 200 RFU
Ladder Size (bp): 1, 100, 200, 300, 400, 500, 600, 700, 800, 900, 1000, 1200, 1500, 2000, 3000, 6000
Quantification Using: Ladder          Final Concentration (ng/uL): 0.0830          Dilution Factor: 12.0
```


Copyright 2015 Advanced Analytical Technologies, Inc.

Sample: 103613-001-005
Well Location: E1
Created: Friday, June 21, 2019 2:11:36 PM

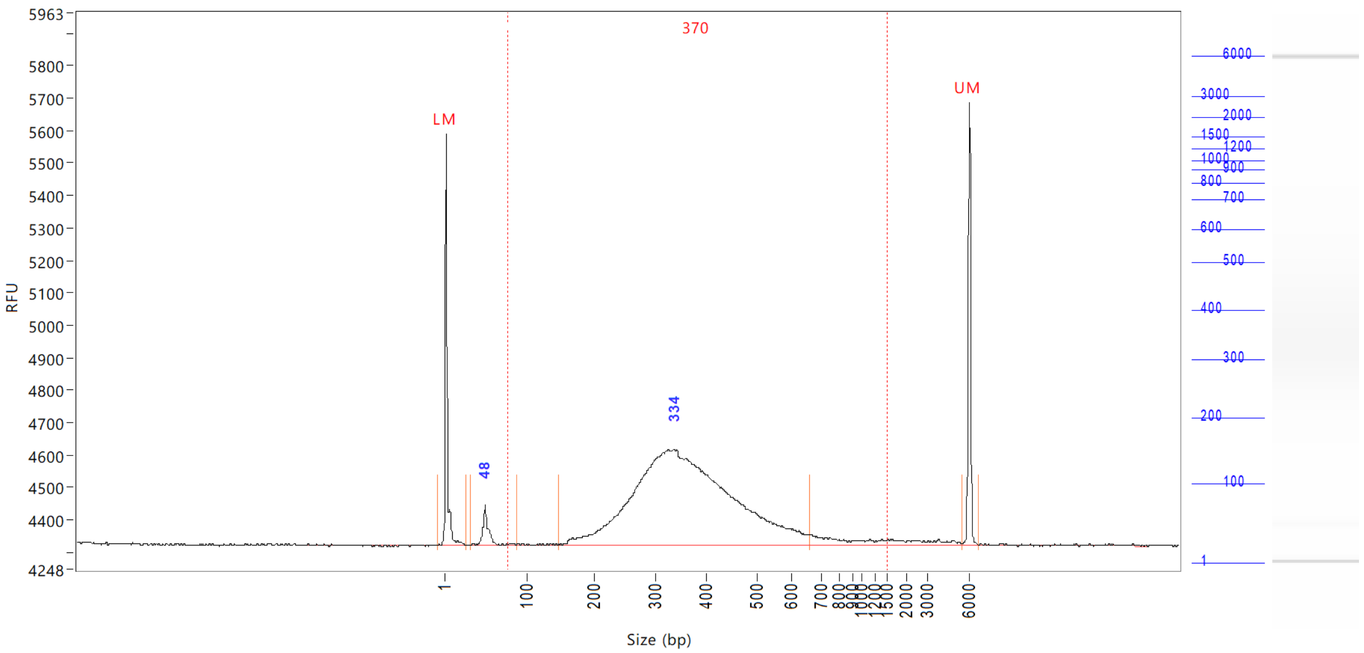

Peak	Size (bp)	Conc. (ng/uL)	From (bp)	To (bp)	RFU
1	1 (LM)	0.0124	0	27	1266
2	48	0.0352	31	87	124
3	334	1.0304	147	664	295
4	6000 (UM)	0.0075	5517	6682	1366

TIC:	1.0656	ng/uL
TIM:	5.8932	nmole/L
Total Conc.:	1.1185	ng/uL

Smear Analysis 75 bp to 1500 bp 1.0655 ng/ul 95.3 %Total 4.7430 nmole/L 370 Avg. Size (b.p.) 36.29 %CV

Sample Peak Width (sec): 50 Sample Min Peak Height: 25 Sample Baseline V to V?: Y Sample Baseline V to V pts: 3
Sample Filter: Binomial # of Pts for Filter: 3 Sample Start Region (min): 0 Sample End Region (min): 50
Manual Baseline Start (min): 10 Manual Baseline End (min): 48
Marker Peak Width (sec): 5 Marker Min Peak Height: 200 Marker Baseline V to V?: Y Marker Baseline V to V pts: 3
Lower Marker Selection: First Peak > 200 RFU Upper Marker Selection: Last Peak > 200 RFU
Ladder Size (bp): 1, 100, 200, 300, 400, 500, 600, 700, 800, 900, 1000, 1200, 1500, 2000, 3000, 6000
Quantification Using: Ladder Final Concentration (ng/uL): 0.0830 Dilution Factor: 12.0

Copyright 2015 Advanced Analytical Technologies, Inc.

Sample: 103613-001-006
Well Location: F1
Created: Friday, June 21, 2019 2:11:36 PM

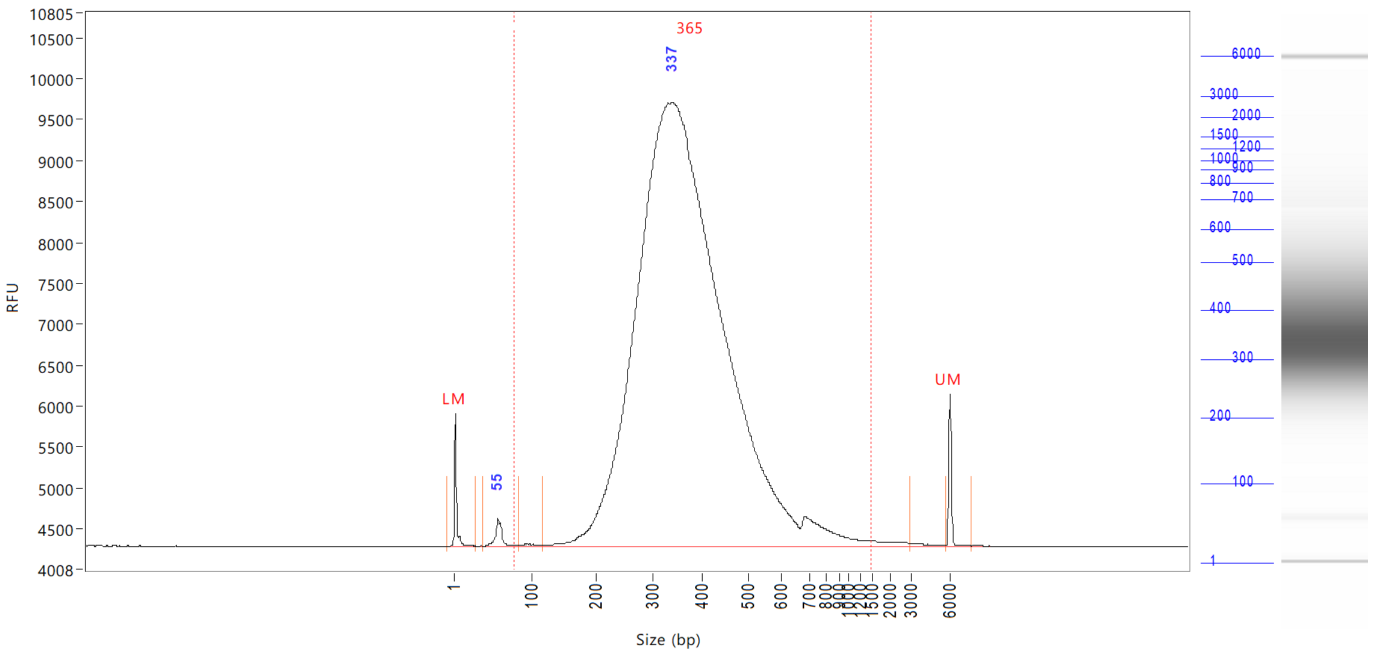

Peak	Size (bp)	Conc. (ng/uL)	From (bp)	To (bp)	RFU
1	1 (LM)	0.0124	0	27	1619
2	55	0.0878	36	82	349
3	337	13.4305	116	2950	5431
4	6000 (UM)	0.0084	5695	7617	1862

TIC:	13.5183	ng/uL
TIM:	62.2877	nmole/L
Total Conc.:	13.5492	ng/uL

Smear Analysis	75 bp to 1500 bp	13.4092 ng/ul	99.0 %Total	60.4428 nmole/L	365 Avg. Size (b.p.)	29.52 %CV

Sample Peak Width (sec): 50 Sample Min Peak Height: 25 Sample Baseline V to V?: Y Sample Baseline V to V pts: 3
Sample Filter: Binomial # of Pts for Filter: 3 Sample Start Region (min): 0 Sample End Region (min): 50
Manual Baseline Start (min): 10 Manual Baseline End (min): 48
Marker Peak Width (sec): 5 Marker Min Peak Height: 200 Marker Baseline V to V?: Y Marker Baseline V to V pts: 3
Lower Marker Selection: First Peak > 200 RFU Upper Marker Selection: Last Peak > 200 RFU
Ladder Size (bp): 1, 100, 200, 300, 400, 500, 600, 700, 800, 900, 1000, 1200, 1500, 2000, 3000, 6000
Quantification Using: Ladder Final Concentration (ng/uL): 0.0830 Dilution Factor: 12.0

Copyright 2015 Advanced Analytical Technologies, Inc.

Sample: 103613-001-007
Well Location: G1
Created: Friday, June 21, 2019 2:11:36 PM

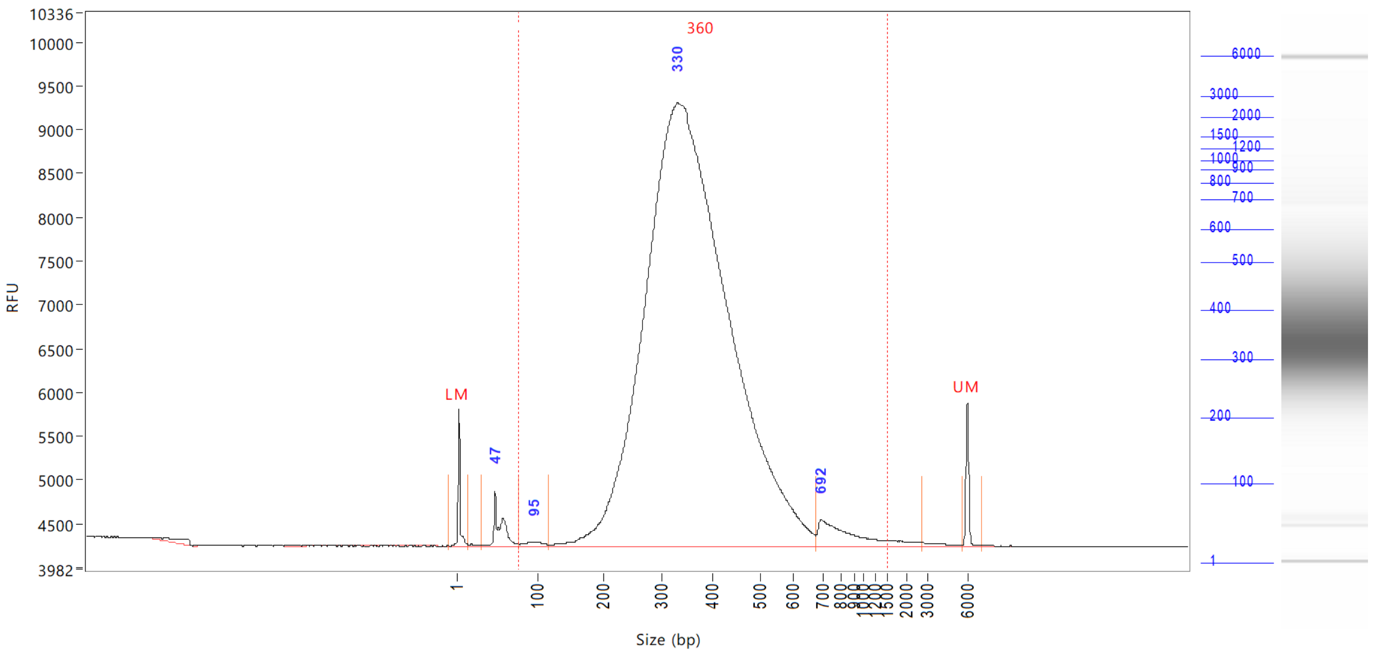

Peak	Size (bp)	Conc. (ng/uL)	From (bp)	To (bp)	RFU
1	1 (LM)	0.0124	0	15	1562
2	47	0.1757	31	77	632
3	95	0.0332	77	117	48
4	330	12.6916	117	675	5075
5	692	0.2537	675	2750	301
6	6000 (UM)	0.0080	5644	7036	1637

TIC:	13.1542	ng/uL
TIM:	65.5716	nmole/L
Total Conc.:	13.1774	ng/uL

Smear Analysis 75 bp to 1500 bp 12.9436 ng/ul 98.2 %Total 59.0834 nmole/L 360 Avg. Size (b.p.) 29.77 %CV

Sample Peak Width (sec): 50 Sample Min Peak Height: 25 Sample Baseline V to V?: Y Sample Baseline V to V pts: 3
Sample Filter: Binomial # of Pts for Filter: 3 Sample Start Region (min): 0 Sample End Region (min): 50
Manual Baseline Start (min): 10 Manual Baseline End (min): 48
Marker Peak Width (sec): 5 Marker Min Peak Height: 200 Marker Baseline V to V?: Y Marker Baseline V to V pts: 3
Lower Marker Selection: First Peak > 200 RFU Upper Marker Selection: Last Peak > 200 RFU
Ladder Size (bp): 1, 100, 200, 300, 400, 500, 600, 700, 800, 900, 1000, 1200, 1500, 2000, 3000, 6000
Quantification Using: Ladder Final Concentration (ng/uL): 0.0830 Dilution Factor: 12.0

 Copyright 2015 Advanced Analytical Technologies, Inc.

Sample: 103613-001-008
Well Location: H1
Created: Friday, June 21, 2019 2:11:36 PM

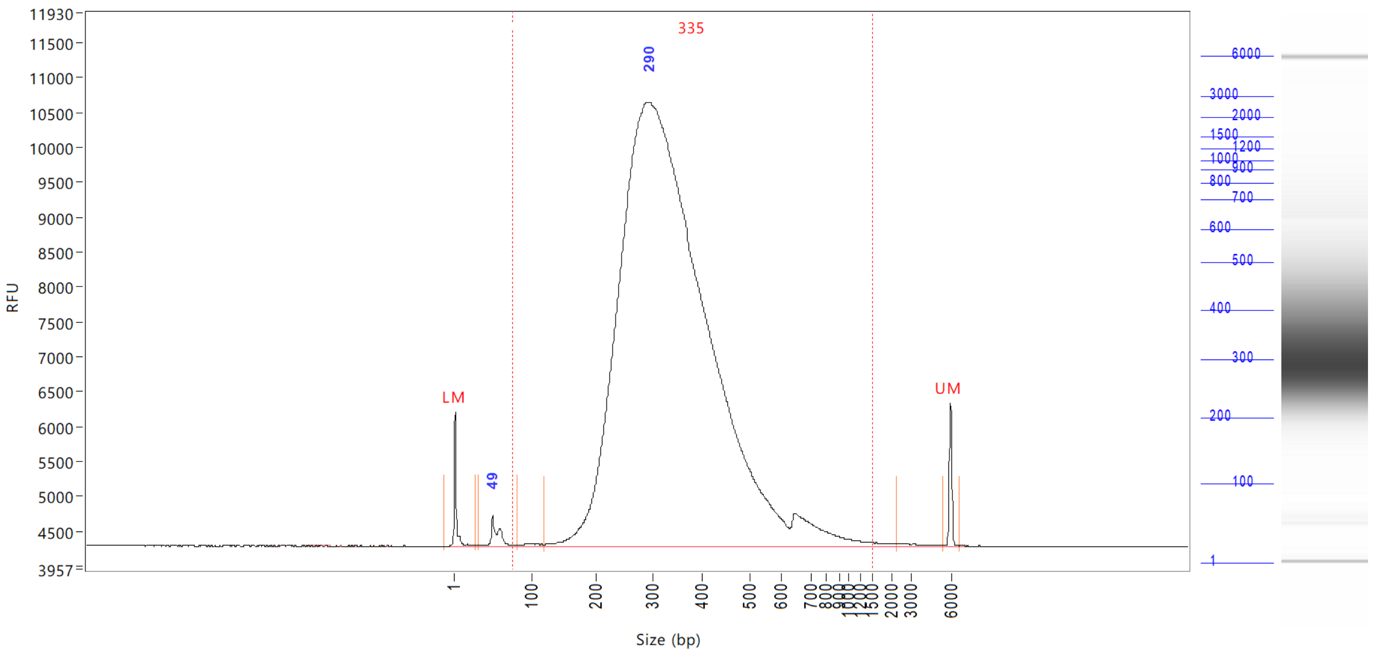

Peak	Size (bp)	Conc. (ng/uL)	From (bp)	To (bp)	RFU
1	1 (LM)	0.0124	0	28	1927
2	49	0.1014	31	82	434
3	290	13.8701	120	2267	6370
4	6000 (UM)	0.0077	5441	6631	2060

TIC:	13.9715	ng/uL
TIM:	70.5297	nmole/L
Total Conc.:	14.0071	ng/uL

Smear Analysis 75 bp to 1500 bp 13.8720 ng/ul 99.0 %Total 68.0549 nmole/L 335 Avg. Size (b.p.) 31.54 %CV

Sample Peak Width (sec): 50 Sample Min Peak Height: 25 Sample Baseline V to V?: Y Sample Baseline V to V pts: 3
Sample Filter: Binomial # of Pts for Filter: 3 Sample Start Region (min): 0 Sample End Region (min): 50
Manual Baseline Start (min): 10 Manual Baseline End (min): 48
Marker Peak Width (sec): 5 Marker Min Peak Height: 200 Marker Baseline V to V?: Y Marker Baseline V to V pts: 3
Lower Marker Selection: First Peak > 200 RFU Upper Marker Selection: Last Peak > 200 RFU
Ladder Size (bp): 1, 100, 200, 300, 400, 500, 600, 700, 800, 900, 1000, 1200, 1500, 2000, 3000, 6000
Quantification Using: Ladder Final Concentration (ng/uL): 0.0830 Dilution Factor: 12.0

 Copyright 2015 Advanced Analytical Technologies, Inc.

Sample: 103613-001-009
Well Location: A2
Created: Friday, June 21, 2019 2:11:36 PM

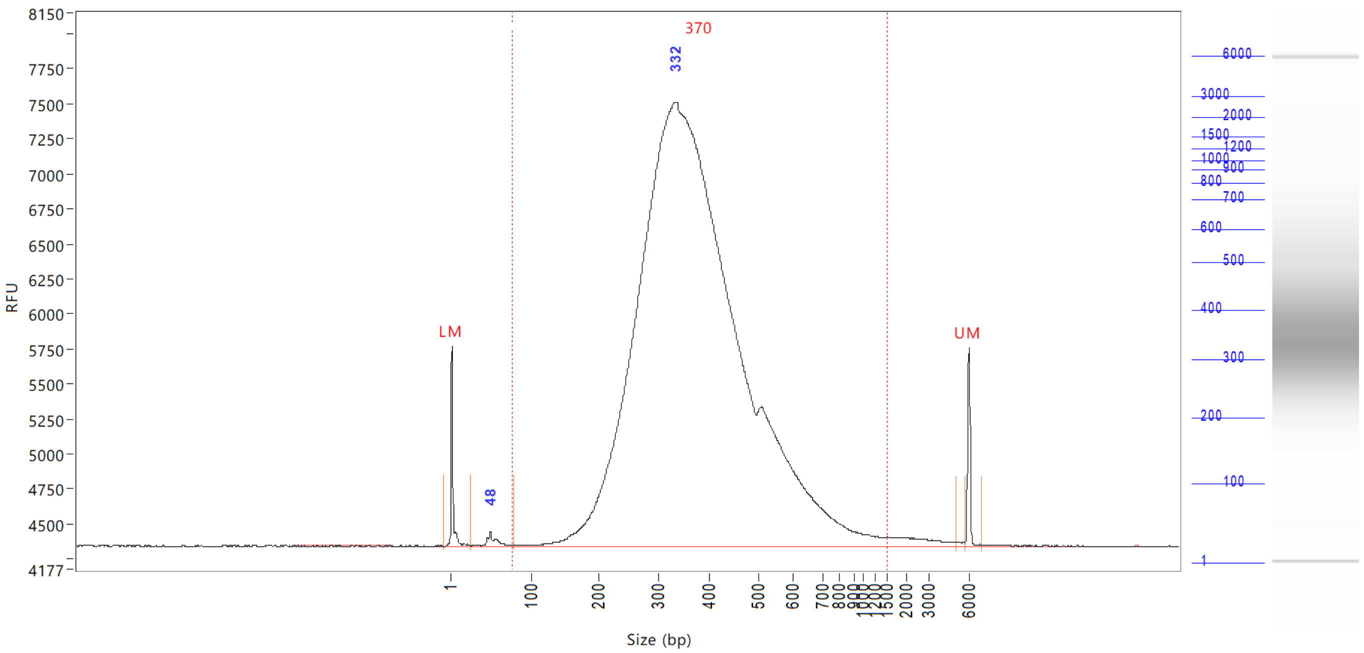

Peak	Size (bp)	Conc. (ng/uL)	From (bp)	To (bp)	RFU
1	1 (LM)	0.0124	0	26	1427
2	48	0.0365	26	77	102
3	332	10.3233	77	5060	3173
4	6000 (UM)	0.0072	5695	6909	1413

TIC:	10.3598	ng/uL
TIM:	45.4023	nmole/L
Total Conc.:	10.3641	ng/uL

Smear Analysis 75 bp to 1500 bp 10.2559 ng/ul 99.0 %Total 45.6594 nmole/L 370 Avg. Size (b.p.) 32.91 %CV

Sample Peak Width (sec): 50 Sample Min Peak Height: 25 Sample Baseline V to V?: Y Sample Baseline V to V pts: 3
Sample Filter: Binomial # of Pts for Filter: 3 Sample Start Region (min): 0 Sample End Region (min): 50
Manual Baseline Start (min): 10 Manual Baseline End (min): 48
Marker Peak Width (sec): 5 Marker Min Peak Height: 200 Marker Baseline V to V?: Y Marker Baseline V to V pts: 3
Lower Marker Selection: First Peak > 200 RFU Upper Marker Selection: Last Peak > 200 RFU
Ladder Size (bp): 1, 100, 200, 300, 400, 500, 600, 700, 800, 900, 1000, 1200, 1500, 2000, 3000, 6000
Quantification Using: Ladder Final Concentration (ng/uL): 0.0830 Dilution Factor: 12.0

 Copyright 2015 Advanced Analytical Technologies, Inc.

Sample: 103613-001-010
Well Location: B2
Created: Friday, June 21, 2019 2:11:36 PM

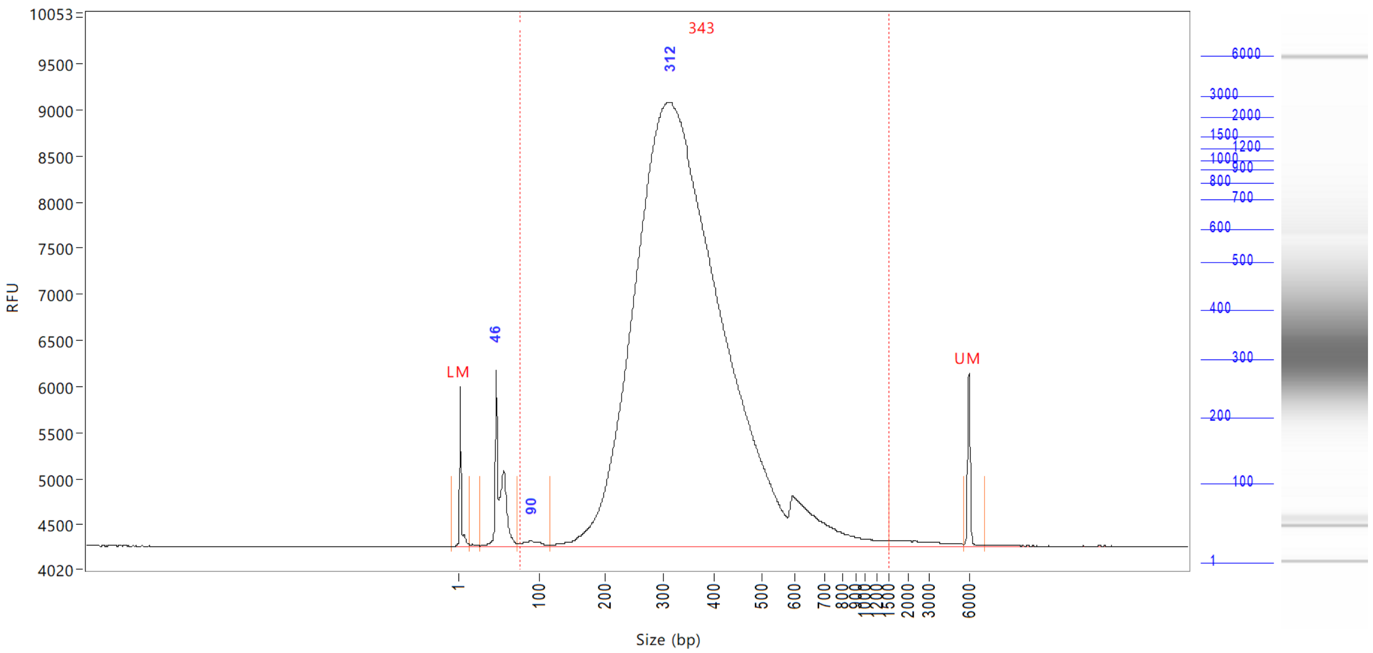

Peak	Size (bp)	Conc. (ng/uL)	From (bp)	To (bp)	RFU
1	1 (LM)	0.0124	0	15	1730
2	46	0.3996	26	74	1907
3	90	0.0396	74	116	58
4	312	11.9353	116	1519	4822
5	6000 (UM)	0.0081	5619	7111	1880

TIC:	12.3745	ng/uL
TIM:	70.6277	nmole/L
Total Conc.:	12.4369	ng/uL

Smear Analysis	75 bp to 1500 bp	11.9725 ng/ul	96.3 %Total	57.3533 nmole/L	343 Avg. Size (b.p.)	31.83 %CV

Sample Peak Width (sec): 50 Sample Min Peak Height: 25 Sample Baseline V to V?: Y Sample Baseline V to V pts: 3
Sample Filter: Binomial # of Pts for Filter: 3 Sample Start Region (min): 0 Sample End Region (min): 50
Manual Baseline Start (min): 10 Manual Baseline End (min): 48
Marker Peak Width (sec): 5 Marker Min Peak Height: 200 Marker Baseline V to V?: Y Marker Baseline V to V pts: 3
Lower Marker Selection: First Peak > 200 RFU Upper Marker Selection: Last Peak > 200 RFU
Ladder Size (bp): 1, 100, 200, 300, 400, 500, 600, 700, 800, 900, 1000, 1200, 1500, 2000, 3000, 6000
Quantification Using: Ladder Final Concentration (ng/uL): 0.0830 Dilution Factor: 12.0

Copyright 2015 Advanced Analytical Technologies, Inc.

Sample: 103613-001-011
Well Location: C2
Created: Friday, June 21, 2019 2:11:36 PM

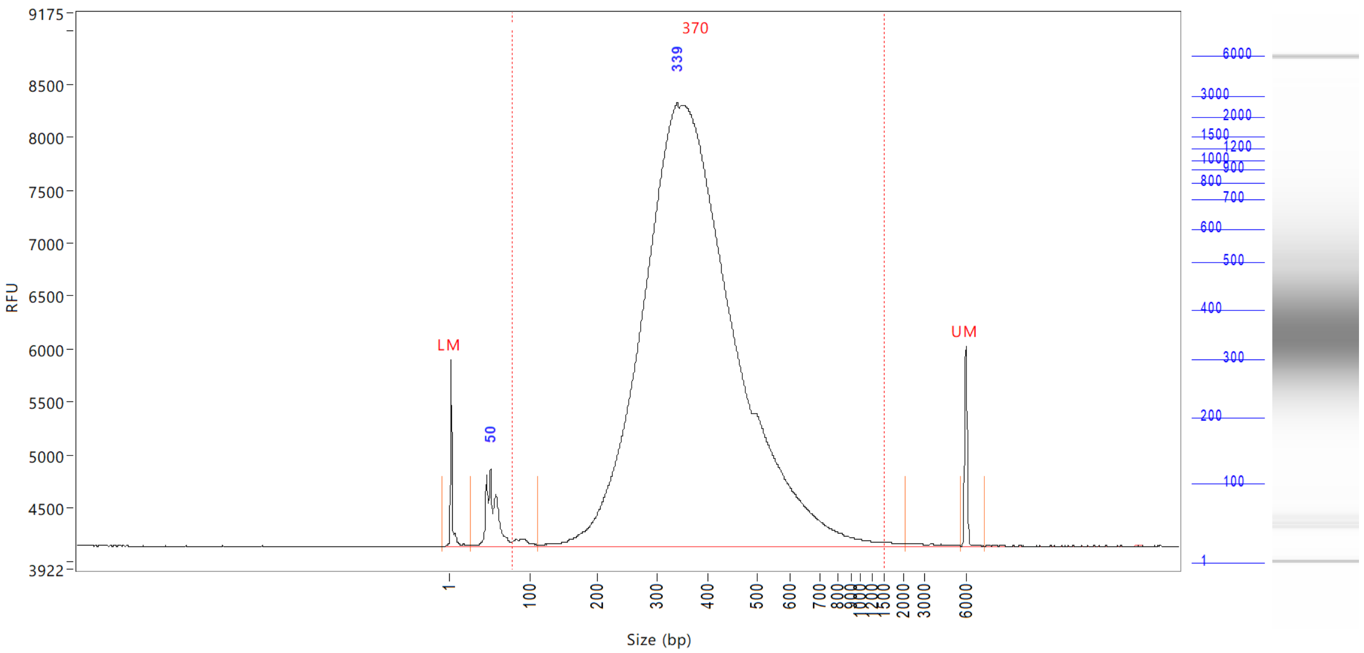

Peak	Size (bp)	Conc. (ng/uL)	From (bp)	To (bp)	RFU
1	1 (LM)	0.0124	0	27	1765
2	50	0.3080	27	111	729
3	339	9.8649	111	2068	4199
4	6000 (UM)	0.0076	5644	7389	1891

TIC:	10.1729	ng/uL
TIM:	52.5256	nmole/L
Total Conc.:	10.1892	ng/uL

Smear Analysis 75 bp to 1500 bp 9.8909 ng/ul 97.1 %Total 43.9755 nmole/L 370 Avg. Size (b.p.) 29.42 %CV

Sample Peak Width (sec): 50 Sample Min Peak Height: 25 Sample Baseline V to V?: Y Sample Baseline V to V pts: 3
Sample Filter: Binomial # of Pts for Filter: 3 Sample Start Region (min): 0 Sample End Region (min): 50
Manual Baseline Start (min): 10 Manual Baseline End (min): 48
Marker Peak Width (sec): 5 Marker Min Peak Height: 200 Marker Baseline V to V?: Y Marker Baseline V to V pts: 3
Lower Marker Selection: First Peak > 200 RFU Upper Marker Selection: Last Peak > 200 RFU
Ladder Size (bp): 1, 100, 200, 300, 400, 500, 600, 700, 800, 900, 1000, 1200, 1500, 2000, 3000, 6000
Quantification Using: Ladder Final Concentration (ng/uL): 0.0830 Dilution Factor: 12.0

 Copyright 2015 Advanced Analytical Technologies, Inc.

Sample: 103613-001-012
Well Location: D2
Created: Friday, June 21, 2019 2:11:36 PM

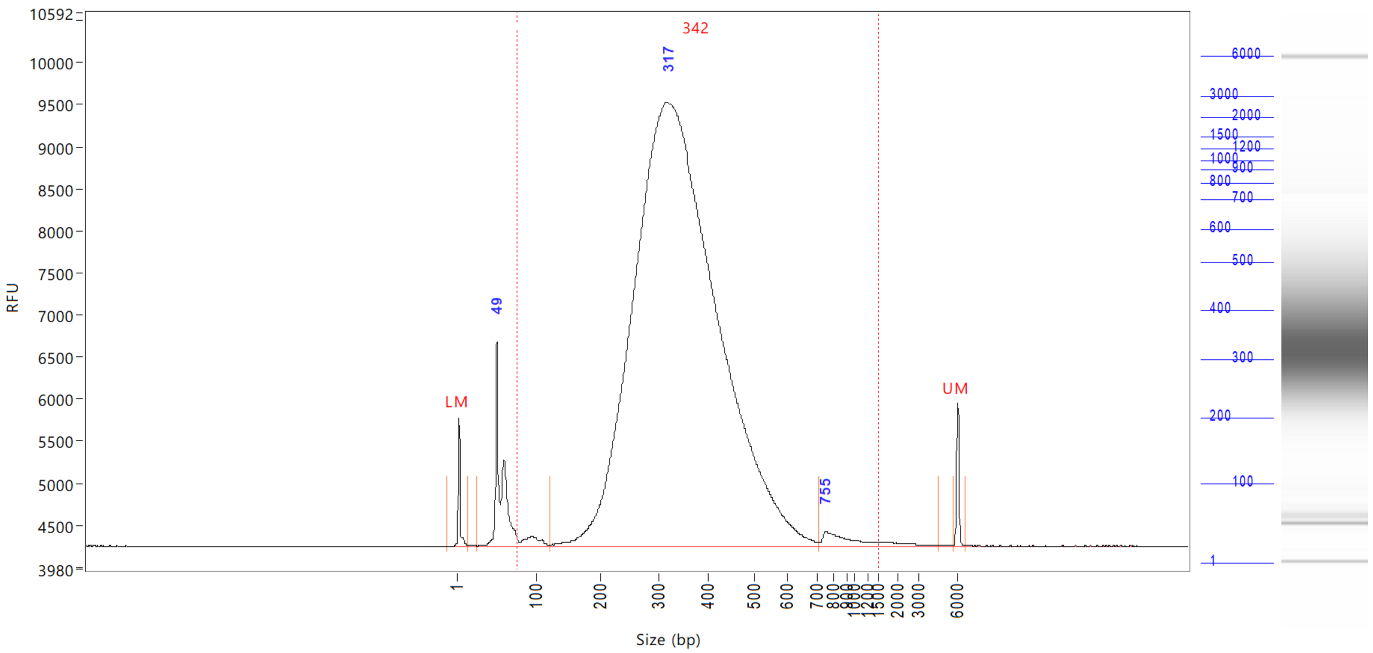

Peak	Size (bp)	Conc. (ng/uL)	From (bp)	To (bp)	RFU
1	1 (LM)	0.0124	0	15	1527
2	49	0.6711	24	121	2432
3	317	13.9625	121	715	5282
4	755	0.1412	715	4526	177
5	6000 (UM)	0.0080	5670	6581	1698

TIC:	14.7748	ng/uL
TIM:	86.2823	nmole/L
Total Conc.:	14.7814	ng/uL

Smear Analysis 75 bp to 1500 bp 14.1563 ng/ul 95.8 %Total 68.0430 nmole/L 342 Avg. Size (b.p.) 28.83 %CV

Sample Peak Width (sec): 50 Sample Min Peak Height: 25 Sample Baseline V to V?: Y Sample Baseline V to V pts: 3
Sample Filter: Binomial # of Pts for Filter: 3 Sample Start Region (min): 0 Sample End Region (min): 50
Manual Baseline Start (min): 10 Manual Baseline End (min): 48
Marker Peak Width (sec): 5 Marker Min Peak Height: 200 Marker Baseline V to V?: Y Marker Baseline V to V pts: 3
Lower Marker Selection: First Peak > 200 RFU Upper Marker Selection: Last Peak > 200 RFU
Ladder Size (bp): 1, 100, 200, 300, 400, 500, 600, 700, 800, 900, 1000, 1200, 1500, 2000, 3000, 6000
Quantification Using: Ladder Final Concentration (ng/uL): 0.0830 Dilution Factor: 12.0

 Copyright 2015 Advanced Analytical Technologies, Inc.

Sample: 103613-001-013
Well Location: E2
Created: Friday, June 21, 2019 2:11:36 PM

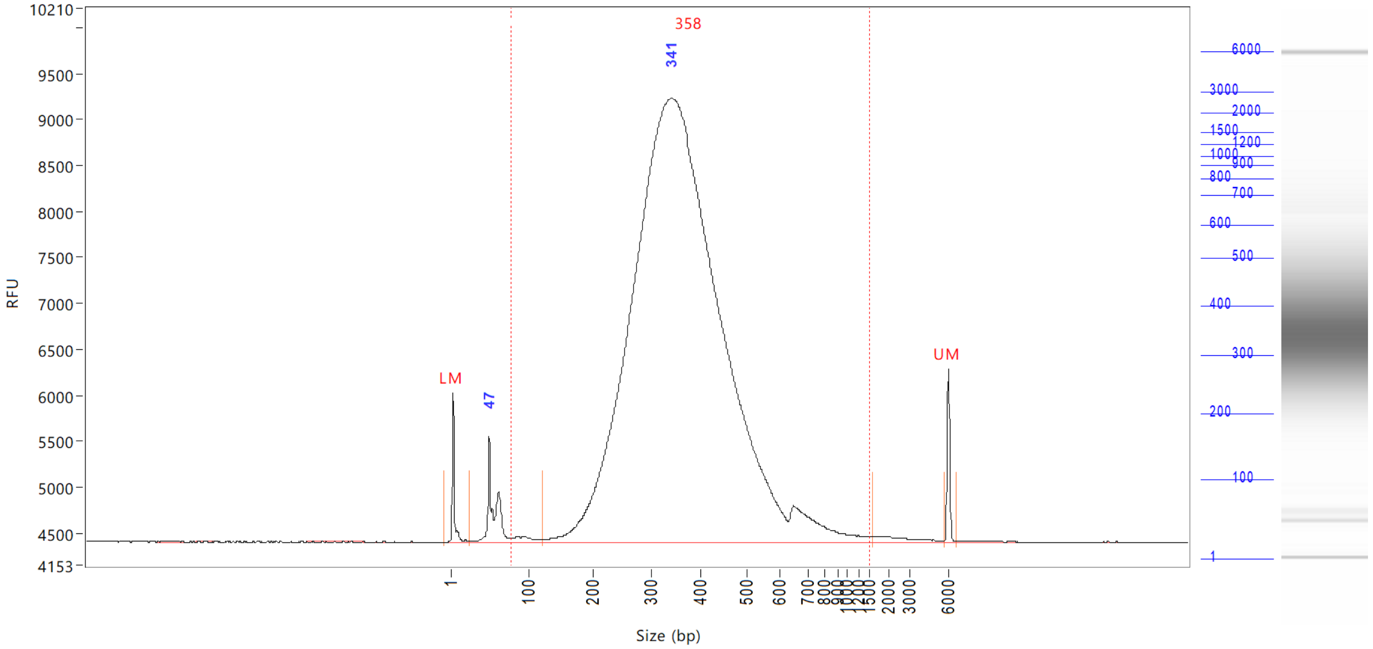

Peak	Size (bp)	Conc. (ng/uL)	From (bp)	To (bp)	RFU
1	1 (LM)	0.0124	0	24	1622
2	47	0.3158	24	119	1156
3	341	12.3053	119	1554	4837
4	6000 (UM)	0.0083	5670	6556	1884

TIC:	12.6210	ng/uL
TIM:	65.3137	nmole/L
Total Conc.:	12.6695	ng/uL

Smear Analysis	75 bp to 1500 bp	12.3442 ng/ul	97.4 %Total	56.7146 nmole/L	358 Avg. Size (b.p.)	30.43 %CV

Sample Peak Width (sec): 50 Sample Min Peak Height: 25 Sample Baseline V to V?: Y Sample Baseline V to V pts: 3
Sample Filter: Binomial # of Pts for Filter: 3 Sample Start Region (min): 0 Sample End Region (min): 50
Manual Baseline Start (min): 10 Manual Baseline End (min): 48
Marker Peak Width (sec): 5 Marker Min Peak Height: 200 Marker Baseline V to V?: Y Marker Baseline V to V pts: 3
Lower Marker Selection: First Peak > 200 RFU Upper Marker Selection: Last Peak > 200 RFU
Ladder Size (bp): 1, 100, 200, 300, 400, 500, 600, 700, 800, 900, 1000, 1200, 1500, 2000, 3000, 6000
Quantification Using: Ladder Final Concentration (ng/uL): 0.0830 Dilution Factor: 12.0

Copyright 2015 Advanced Analytical Technologies, Inc.

Sample: 103613-001-014
Well Location: F2
Created: Friday, June 21, 2019 2:11:36 PM

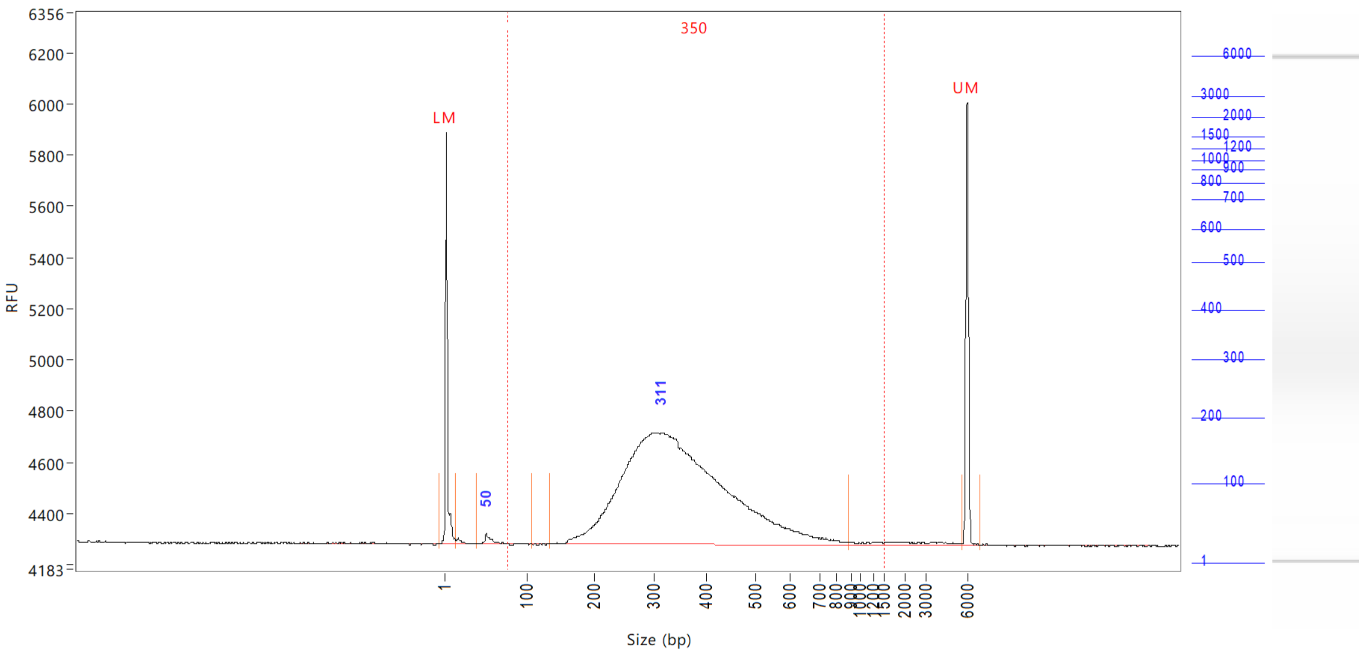

Peak	Size (bp)	Conc. (ng/uL)	From (bp)	To (bp)	RFU
1	1 (LM)	0.0124	0	14	1608
2	50	0.0103	39	108	41
3	311	1.3197	134	890	436
4	6000 (UM)	0.0079	5644	6960	1731
	TIC:	1.3300	ng/uL		
	TIM:	6.5687	nmole/L		
	Total Conc.:	1.3521	ng/uL		

Smear Analysis	75 bp to 1500 bp	1.3256 ng/ul	98.0 %Total	6.2232 nmole/L	350 Avg. Size (b.p.)	32.96 %CV

Sample Peak Width (sec): 50 Sample Min Peak Height: 25 Sample Baseline V to V?: Y Sample Baseline V to V pts: 3
Sample Filter: Binomial # of Pts for Filter: 3 Sample Start Region (min): 0 Sample End Region (min): 50
Manual Baseline Start (min): 10 Manual Baseline End (min): 48
Marker Peak Width (sec): 5 Marker Min Peak Height: 200 Marker Baseline V to V?: Y Marker Baseline V to V pts: 3
Lower Marker Selection: First Peak > 200 RFU Upper Marker Selection: Last Peak > 200 RFU
Ladder Size (bp): 1, 100, 200, 300, 400, 500, 600, 700, 800, 900, 1000, 1200, 1500, 2000, 3000, 6000
Quantification Using: Ladder Final Concentration (ng/uL): 0.0830 Dilution Factor: 12.0

Copyright 2015 Advanced Analytical Technologies, Inc.

Sample: 103613-001-015
Well Location: G2
Created: Friday, June 21, 2019 2:11:36 PM

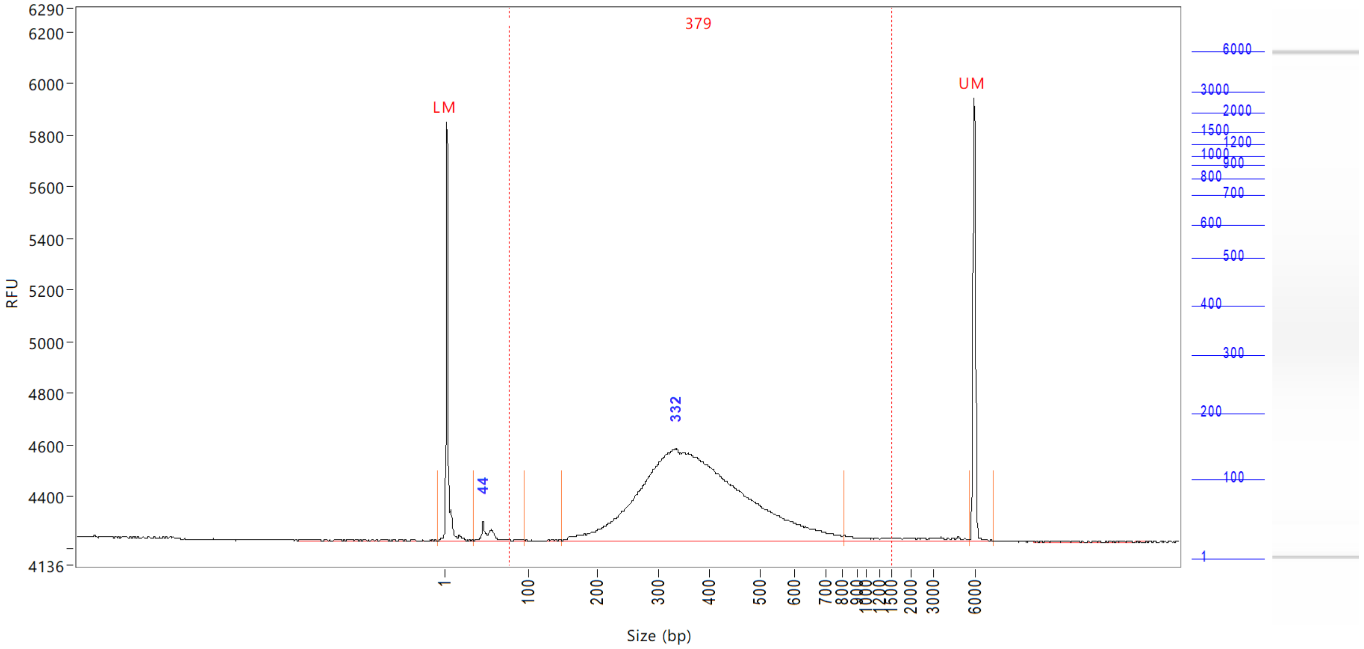

Peak	Size (bp)	Conc. (ng/uL)	From (bp)	To (bp)	RFU
1	1 (LM)	0.0124	0	34	1618
2	44	0.0245	34	94	76
3	332	1.0246	148	811	357
4	6000 (UM)	0.0074	5593	7389	1719

TIC:	1.0491	ng/uL
TIM:	5.2621	nmole/L
Total Conc.:	1.0797	ng/uL

Smear Analysis	75 bp to 1500 bp	1.0407 ng/ul	96.4 %Total	4.5184 nmole/L	379 Avg. Size (b.p.)	34.45 %CV

Sample Peak Width (sec): 50 Sample Min Peak Height: 25 Sample Baseline V to V?: Y Sample Baseline V to V pts: 3
Sample Filter: Binomial # of Pts for Filter: 3 Sample Start Region (min): 0 Sample End Region (min): 50
Manual Baseline Start (min): 10 Manual Baseline End (min): 48
Marker Peak Width (sec): 5 Marker Min Peak Height: 200 Marker Baseline V to V?: Y Marker Baseline V to V pts: 3
Lower Marker Selection: First Peak > 200 RFU Upper Marker Selection: Last Peak > 200 RFU
Ladder Size (bp): 1, 100, 200, 300, 400, 500, 600, 700, 800, 900, 1000, 1200, 1500, 2000, 3000, 6000
Quantification Using: Ladder Final Concentration (ng/uL): 0.0830 Dilution Factor: 12.0

Copyright 2015 Advanced Analytical Technologies, Inc.

Sample: 103613-001-016
Well Location: H2
Created: Friday, June 21, 2019 2:11:36 PM

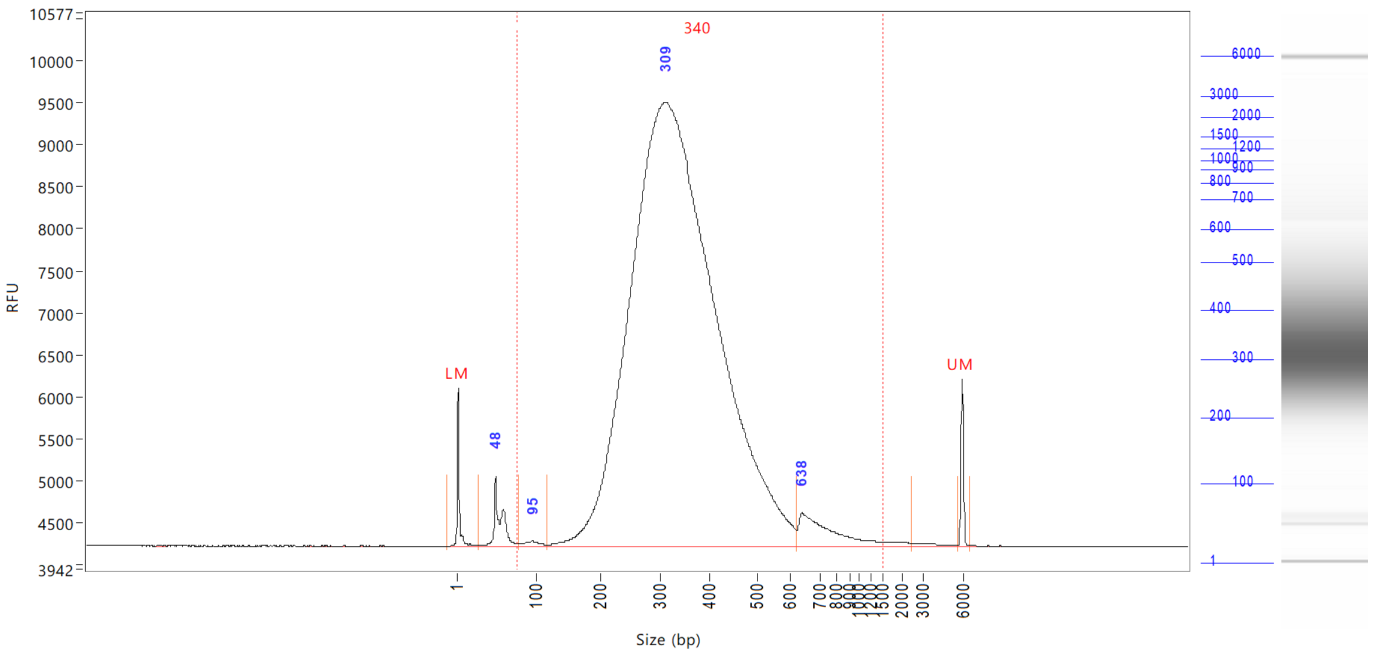

Peak	Size (bp)	Conc. (ng/uL)	From (bp)	To (bp)	RFU
1	1 (LM)	0.0124	0	27	1881
2	48	0.1971	27	78	838
3	95	0.0294	78	116	58
4	309	11.6091	116	622	5299
5	638	0.2761	622	2451	398
6	6000 (UM)	0.0075	5670	6606	1988

TIC:	12.1117	ng/uL
TIM:	64.7264	nmole/L
Total Conc.:	12.1263	ng/uL

Smear Analysis 75 bp to 1500 bp 11.8942 ng/ul 98.1 %Total 57.5852 nmole/L 340 Avg. Size (b.p.) 30.81 %CV

Sample Peak Width (sec): 50 Sample Min Peak Height: 25 Sample Baseline V to V?: Y Sample Baseline V to V pts: 3
Sample Filter: Binomial # of Pts for Filter: 3 Sample Start Region (min): 0 Sample End Region (min): 50
Manual Baseline Start (min): 10 Manual Baseline End (min): 48
Marker Peak Width (sec): 5 Marker Min Peak Height: 200 Marker Baseline V to V?: Y Marker Baseline V to V pts: 3
Lower Marker Selection: First Peak > 200 RFU Upper Marker Selection: Last Peak > 200 RFU
Ladder Size (bp): 1, 100, 200, 300, 400, 500, 600, 700, 800, 900, 1000, 1200, 1500, 2000, 3000, 6000
Quantification Using: Ladder Final Concentration (ng/uL): 0.0830 Dilution Factor: 12.0

 Copyright 2015 Advanced Analytical Technologies, Inc.

Sample: 103613-001-017
Well Location: A3
Created: Friday, June 21, 2019 2:11:36 PM

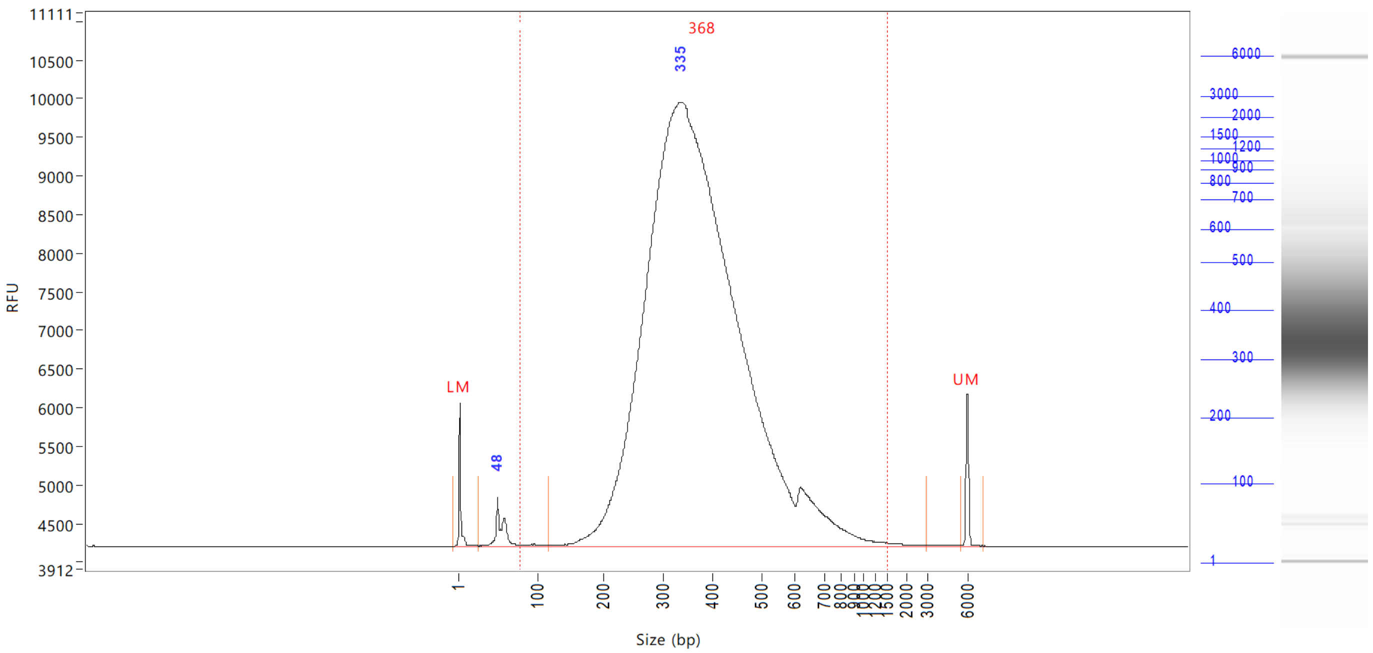

Peak	Size (bp)	Conc. (ng/uL)	From (bp)	To (bp)	RFU
1	1 (LM)	0.0124	0	26	1849
2	48	0.1667	26	115	629
3	335	13.0558	115	3000	5755
4	6000 (UM)	0.0076	5517	7162	1977

TIC:	13.2225	ng/uL
TIM:	62.7754	nmole/L
Total Conc.:	13.2317	ng/uL

Smear Analysis 75 bp to 1500 bp 13.0558 ng/ul 98.7 %Total 58.3082 nmole/L 368 Avg. Size (b.p.) 29.68 %CV

Sample Peak Width (sec): 50 Sample Min Peak Height: 25 Sample Baseline V to V?: Y Sample Baseline V to V pts: 3
Sample Filter: Binomial # of Pts for Filter: 3 Sample Start Region (min): 0 Sample End Region (min): 50
Manual Baseline Start (min): 10 Manual Baseline End (min): 48
Marker Peak Width (sec): 5 Marker Min Peak Height: 200 Marker Baseline V to V?: Y Marker Baseline V to V pts: 3
Lower Marker Selection: First Peak > 200 RFU Upper Marker Selection: Last Peak > 200 RFU
Ladder Size (bp): 1, 100, 200, 300, 400, 500, 600, 700, 800, 900, 1000, 1200, 1500, 2000, 3000, 6000
Quantification Using: Ladder Final Concentration (ng/uL): 0.0830 Dilution Factor: 12.0

Sample: 103613-001-018
Well Location: B3
Created: Friday, June 21, 2019 2:11:36 PM

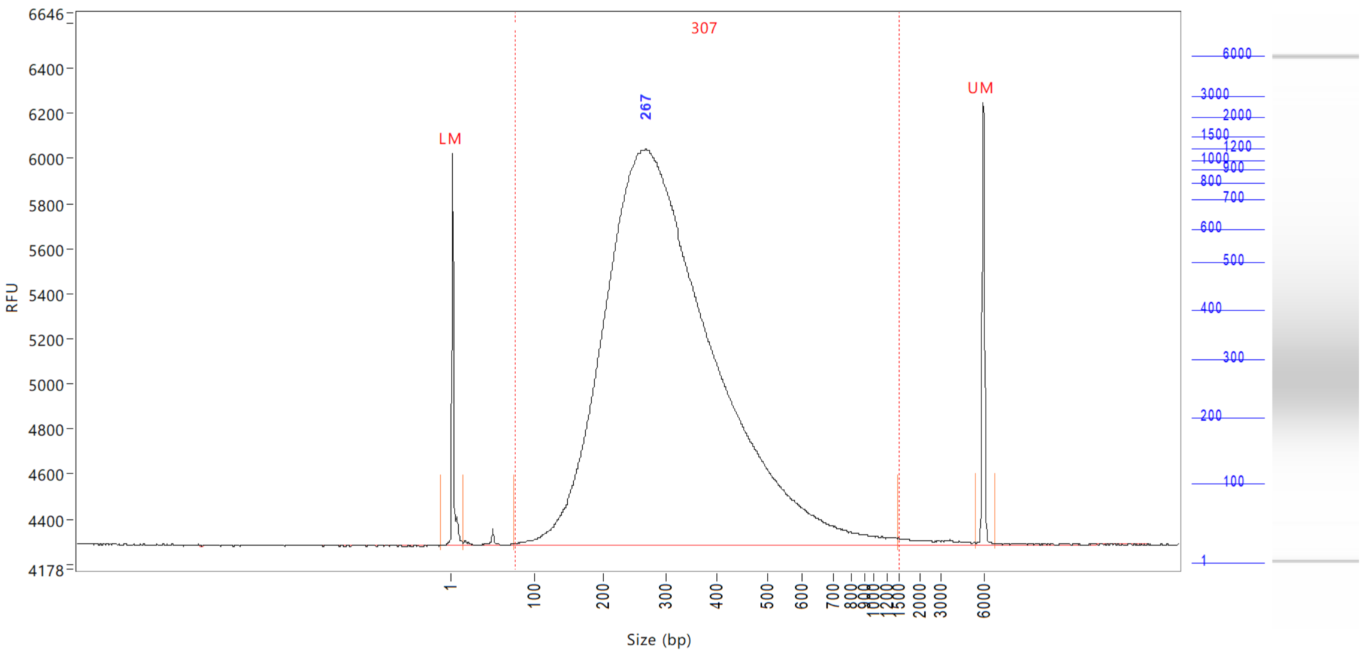

Peak	Size (bp)	Conc. (ng/uL)	From (bp)	To (bp)	RFU
1	1 (LM)	0.0124	0	14	1742
2	267	5.4291	74	1464	1759
3	6000 (UM)	0.0079	5466	6808	1965

TIC:	5.4291	ng/uL
TIM:	29.0902	nmole/L
Total Conc.:	5.4655	ng/uL

Smear Analysis 75 bp to 1500 bp 5.4296 ng/ul 99.3 %Total 29.0889 nmole/L 307 Avg. Size (b.p.) 38.38 %CV

Sample Peak Width (sec): 50 Sample Min Peak Height: 25 Sample Baseline V to V?: Y Sample Baseline V to V pts: 3
Sample Filter: Binomial # of Pts for Filter: 3 Sample Start Region (min): 0 Sample End Region (min): 50
Manual Baseline Start (min): 10 Manual Baseline End (min): 48
Marker Peak Width (sec): 5 Marker Min Peak Height: 200 Marker Baseline V to V?: Y Marker Baseline V to V pts: 3
Lower Marker Selection: First Peak > 200 RFU Upper Marker Selection: Last Peak > 200 RFU
Ladder Size (bp): 1, 100, 200, 300, 400, 500, 600, 700, 800, 900, 1000, 1200, 1500, 2000, 3000, 6000
Quantification Using: Ladder Final Concentration (ng/uL): 0.0830 Dilution Factor: 12.0

 Copyright 2015 Advanced Analytical Technologies, Inc.

Sample: 103613-001-019
Well Location: C3
Created: Friday, June 21, 2019 2:11:36 PM

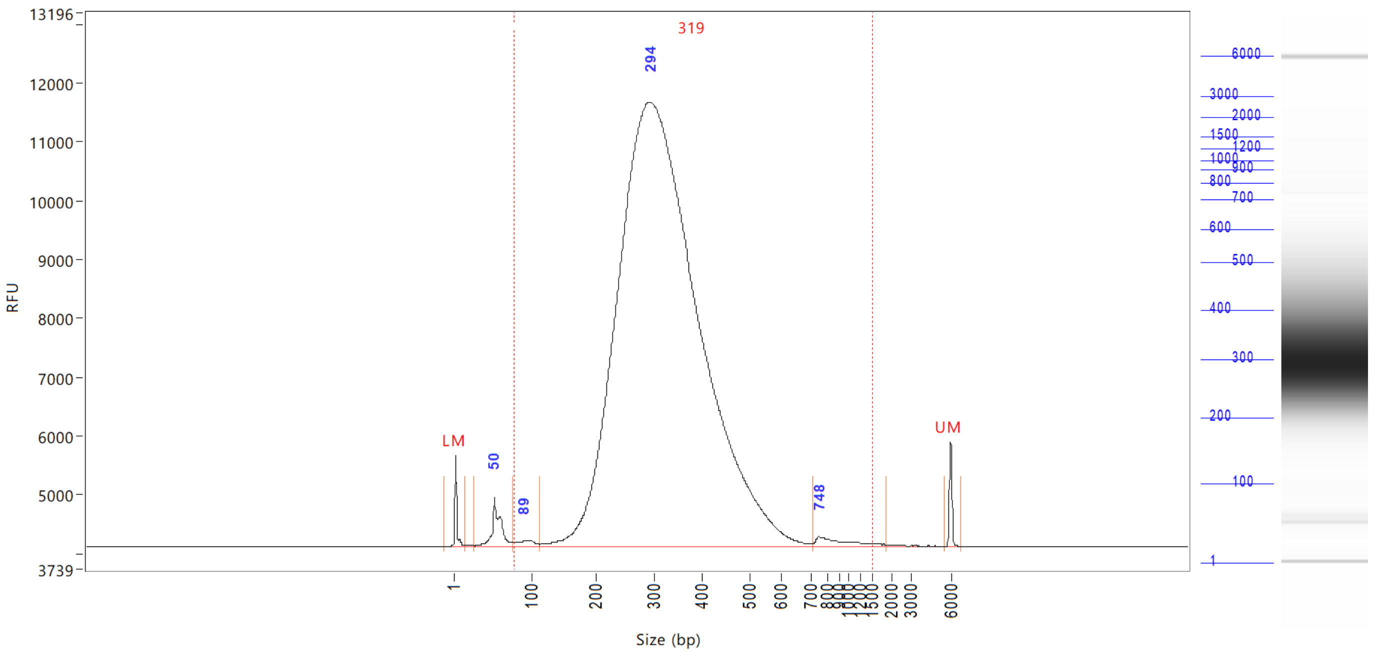

Peak	Size (bp)	Conc. (ng/uL)	From (bp)	To (bp)	RFU
1	1 (LM)	0.0124	0	14	1545
2	50	0.2939	26	74	846
3	89	0.0748	74	111	97
4	294	19.2305	111	713	7563
5	748	0.1214	713	1875	170
6	6000 (UM)	0.0083	5543	6808	1789

TIC:	19.7207	ng/uL
TIM:	110.2772	nmole/L
Total Conc.:	19.7412	ng/uL

Smear Analysis 75 bp to 1500 bp 19.4127 ng/ul 98.3 %Total 100.0091 nmole/L 319 Avg. Size (b.p.) 29.04 %CV

Sample Peak Width (sec): 50 Sample Min Peak Height: 25 Sample Baseline V to V?: Y Sample Baseline V to V pts: 3
Sample Filter: Binomial # of Pts for Filter: 3 Sample Start Region (min): 0 Sample End Region (min): 50
Manual Baseline Start (min): 10 Manual Baseline End (min): 48
Marker Peak Width (sec): 5 Marker Min Peak Height: 200 Marker Baseline V to V?: Y Marker Baseline V to V pts: 3
Lower Marker Selection: First Peak > 200 RFU Upper Marker Selection: Last Peak > 200 RFU
Ladder Size (bp): 1, 100, 200, 300, 400, 500, 600, 700, 800, 900, 1000, 1200, 1500, 2000, 3000, 6000
Quantification Using: Ladder Final Concentration (ng/uL): 0.0830 Dilution Factor: 12.0

 Copyright 2015 Advanced Analytical Technologies, Inc.

Sample: 103613-001-020
Well Location: D3
Created: Friday, June 21, 2019 2:11:36 PM

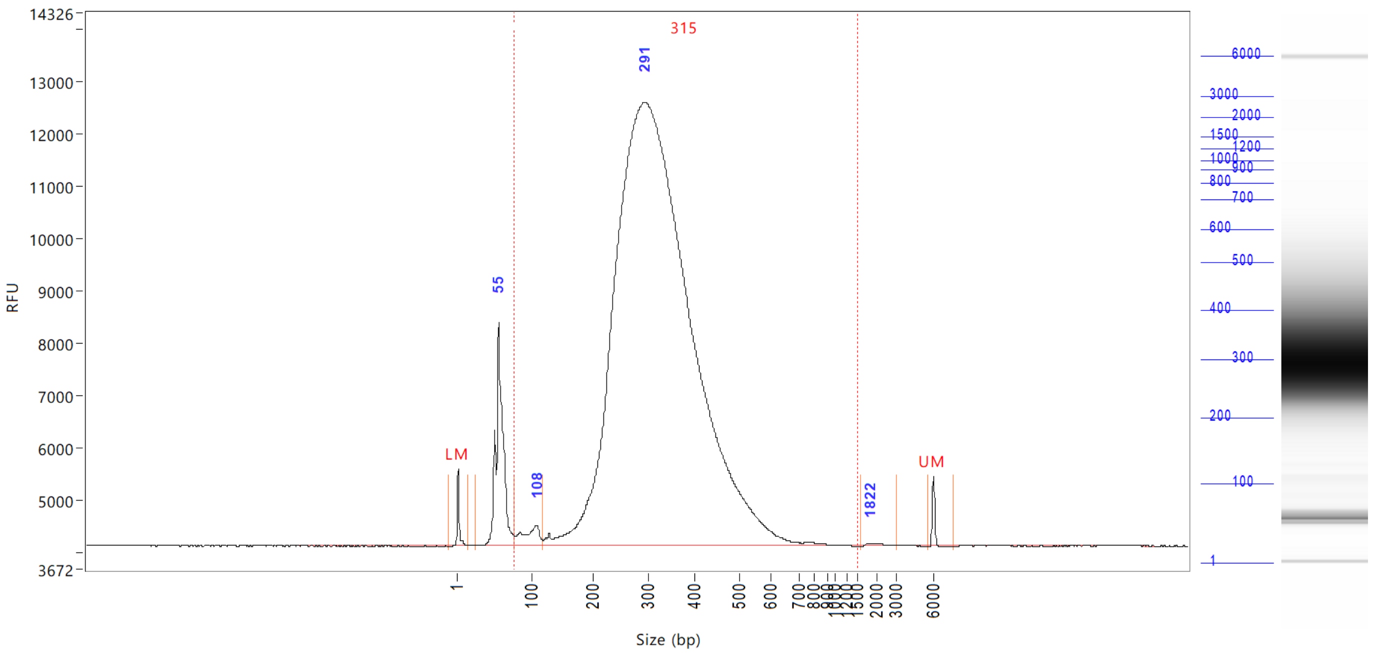

Peak	Size (bp)	Conc. (ng/uL)	From (bp)	To (bp)	RFU
1	1 (LM)	0.0124	0	15	1466
2	55	1.4379	26	78	4287
3	108	0.2615	78	118	398
4	291	22.4996	118	1581	8504
5	1822	0.0187	1581	3103	37
6	6000 (UM)	0.0065	5543	7566	1321
	TIC:	24.2178	ng/uL		
	TIM:	162.1750	nmole/L		
	Total Conc.:	24.2207	ng/uL		

Smear Analysis 75 bp to 1500 bp 22.7739 ng/ul 94.0 %Total 118.8363 nmole/L 315 Avg. Size (b.p.) 26.50 %CV

Sample Peak Width (sec): 50 Sample Min Peak Height: 25 Sample Baseline V to V?: Y Sample Baseline V to V pts: 3
Sample Filter: Binomial # of Pts for Filter: 3 Sample Start Region (min): 0 Sample End Region (min): 50
Manual Baseline Start (min): 10 Manual Baseline End (min): 48
Marker Peak Width (sec): 5 Marker Min Peak Height: 200 Marker Baseline V to V?: Y Marker Baseline V to V pts: 3
Lower Marker Selection: First Peak > 200 RFU Upper Marker Selection: Last Peak > 200 RFU
Ladder Size (bp): 1, 100, 200, 300, 400, 500, 600, 700, 800, 900, 1000, 1200, 1500, 2000, 3000, 6000
Quantification Using: Ladder Final Concentration (ng/uL): 0.0830 Dilution Factor: 12.0

 Copyright 2015 Advanced Analytical Technologies, Inc.

Sample: 103613-001-021
Well Location: E3
Created: Friday, June 21, 2019 2:11:36 PM

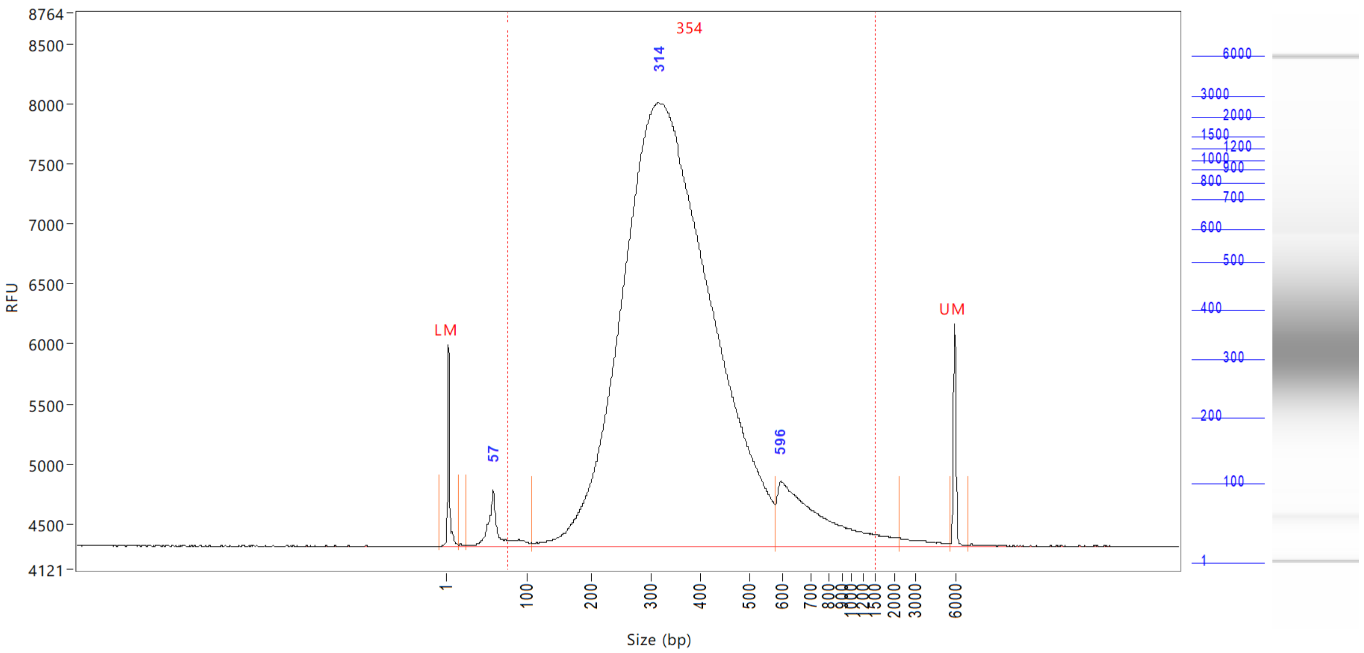

Peak	Size (bp)	Conc. (ng/uL)	From (bp)	To (bp)	RFU
1	1 (LM)	0.0124	0	14	1685
2	57	0.1855	24	109	475
3	314	9.6270	109	579	3708
4	596	0.5690	579	2267	540
5	6000 (UM)	0.0082	5670	6960	1861
	TIC:	10.3816	ng/uL		
	TIM:	53.4765	nmole/L		
	Total Conc.:	10.4182	ng/uL		

Smear Analysis 75 bp to 1500 bp 10.1907 ng/ul 97.8 %Total 47.3698 nmole/L 354 Avg. Size (b.p.) 36.20 %CV

Sample Peak Width (sec): 50 Sample Min Peak Height: 25 Sample Baseline V to V?: Y Sample Baseline V to V pts: 3
Sample Filter: Binomial # of Pts for Filter: 3 Sample Start Region (min): 0 Sample End Region (min): 50
Manual Baseline Start (min): 10 Manual Baseline End (min): 48
Marker Peak Width (sec): 5 Marker Min Peak Height: 200 Marker Baseline V to V?: Y Marker Baseline V to V pts: 3
Lower Marker Selection: First Peak > 200 RFU Upper Marker Selection: Last Peak > 200 RFU
Ladder Size (bp): 1, 100, 200, 300, 400, 500, 600, 700, 800, 900, 1000, 1200, 1500, 2000, 3000, 6000
Quantification Using: Ladder Final Concentration (ng/uL): 0.0830 Dilution Factor: 12.0

 Copyright 2015 Advanced Analytical Technologies, Inc.

Sample: 103613-001-022
Well Location: F3
Created: Friday, June 21, 2019 2:11:36 PM

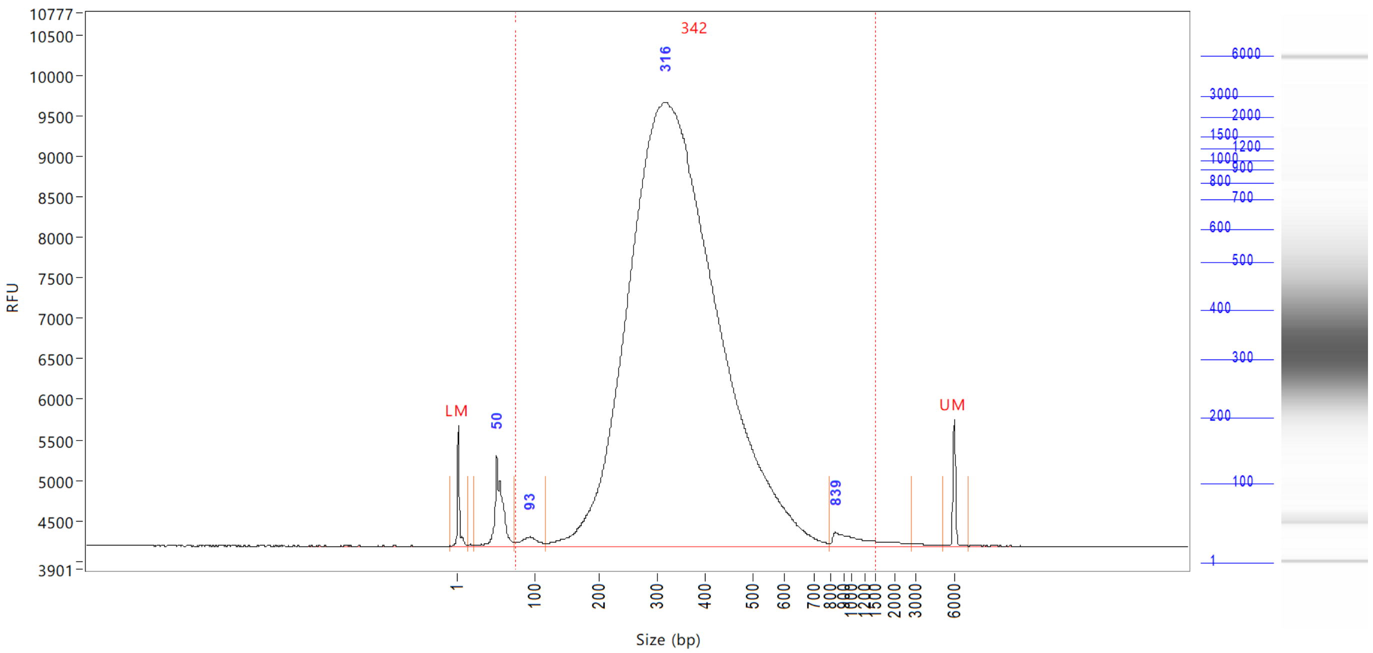

Peak	Size (bp)	Conc. (ng/uL)	From (bp)	To (bp)	RFU
1	1 (LM)	0.0124	0	15	1491
2	50	0.3614	23	75	1113
3	93	0.0788	75	117	116
4	316	15.5625	117	800	5493
5	839	0.1317	800	2834	173
6	6000 (UM)	0.0076	5110	7111	1560

TIC:	16.1344	ng/uL
TIM:	88.0513	nmole/L
Total Conc.:	16.1490	ng/uL

Smear Analysis 75 bp to 1500 bp 15.7391 ng/ul 97.5 %Total 75.6451 nmole/L 342 Avg. Size (b.p.) 30.62 %CV

```
Sample Peak Width (sec): 50   Sample Min Peak Height: 25    Sample Baseline V to V?: Y    Sample Baseline V to V pts: 3
Sample Filter: Binomial       # of Pts for Filter: 3        Sample Start Region (min): 0  Sample End Region (min): 50
Manual Baseline Start (min): 10     Manual Baseline End (min): 48
Marker Peak Width (sec): 5    Marker Min Peak Height: 200   Marker Baseline V to V?: Y    Marker Baseline V to V pts: 3
Lower Marker Selection: First Peak > 200 RFU                Upper Marker Selection: Last Peak > 200 RFU
Ladder Size (bp): 1, 100, 200, 300, 400, 500, 600, 700, 800, 900, 1000, 1200, 1500, 2000, 3000, 6000
Quantification Using: Ladder          Final Concentration (ng/uL): 0.0830          Dilution Factor: 12.0
```


Copyright 2015 Advanced Analytical Technologies, Inc.

Sample: 103613-001-023
Well Location: G3
Created: Friday, June 21, 2019 2:11:36 PM

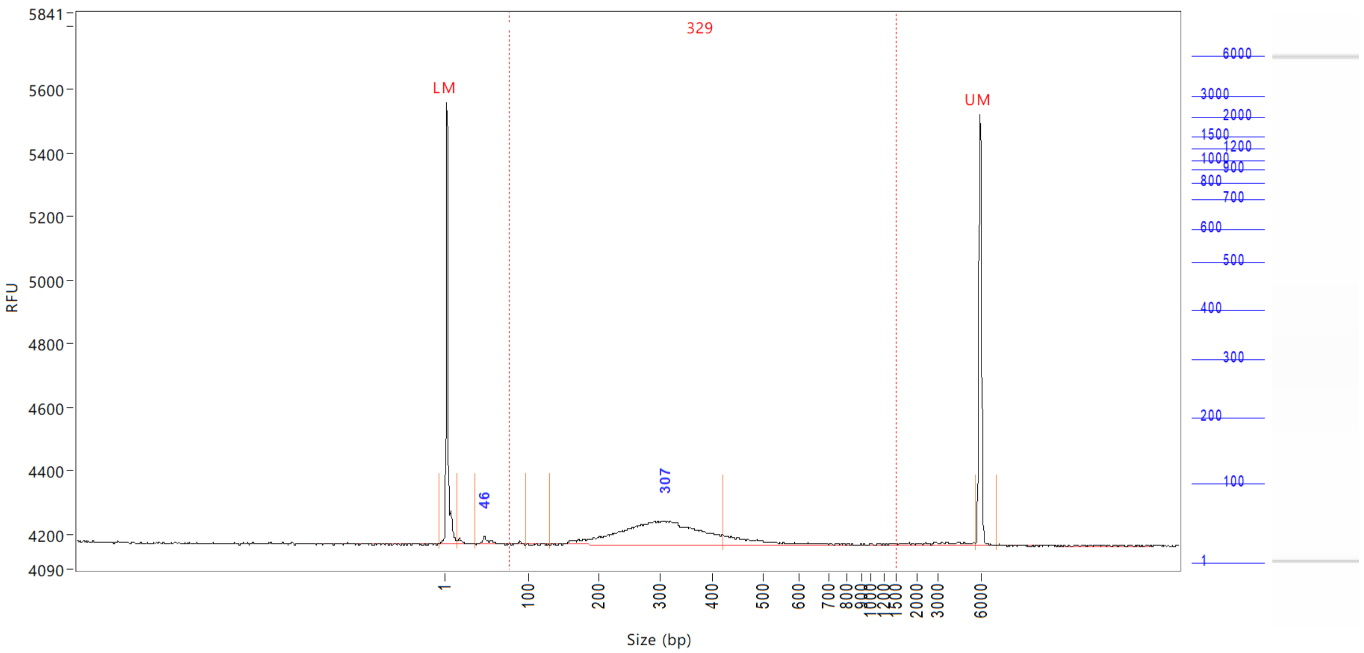

Peak	Size (bp)	Conc. (ng/uL)	From (bp)	To (bp)	RFU
1	1 (LM)	0.0124	0	14	1391
2	46	0.0095	36	95	27
3	307	0.2063	130	421	75
4	6000 (UM)	0.0072	5695	7137	1359
	TIC:	0.2158	ng/uL		
	TIM:	1.4363	nmole/L		
	Total Conc.:	0.2643	ng/uL		

Smear Analysis	75 bp to 1500 bp	0.2416 ng/ul	91.4 %Total	1.2078 nmole/L	329 Avg. Size (b.p.)	41.34 %CV

Sample Peak Width (sec): 50   Sample Min Peak Height: 25   Sample Baseline V to V?: Y   Sample Baseline V to V pts: 3
Sample Filter: Binomial   # of Pts for Filter: 3   Sample Start Region (min): 0   Sample End Region (min): 50
Manual Baseline Start (min): 10   Manual Baseline End (min): 48
Marker Peak Width (sec): 5   Marker Min Peak Height: 200   Marker Baseline V to V?: Y   Marker Baseline V to V pts: 3
Lower Marker Selection: First Peak > 200 RFU   Upper Marker Selection: Last Peak > 200 RFU
Ladder Size (bp): 1, 100, 200, 300, 400, 500, 600, 700, 800, 900, 1000, 1200, 1500, 2000, 3000, 6000
Quantification Using: Ladder   Final Concentration (ng/uL): 0.0830   Dilution Factor: 12.0

Copyright 2015 Advanced Analytical Technologies, Inc.

Sample: 103613-001-024
Well Location: H3
Created: Friday, June 21, 2019 2:11:36 PM

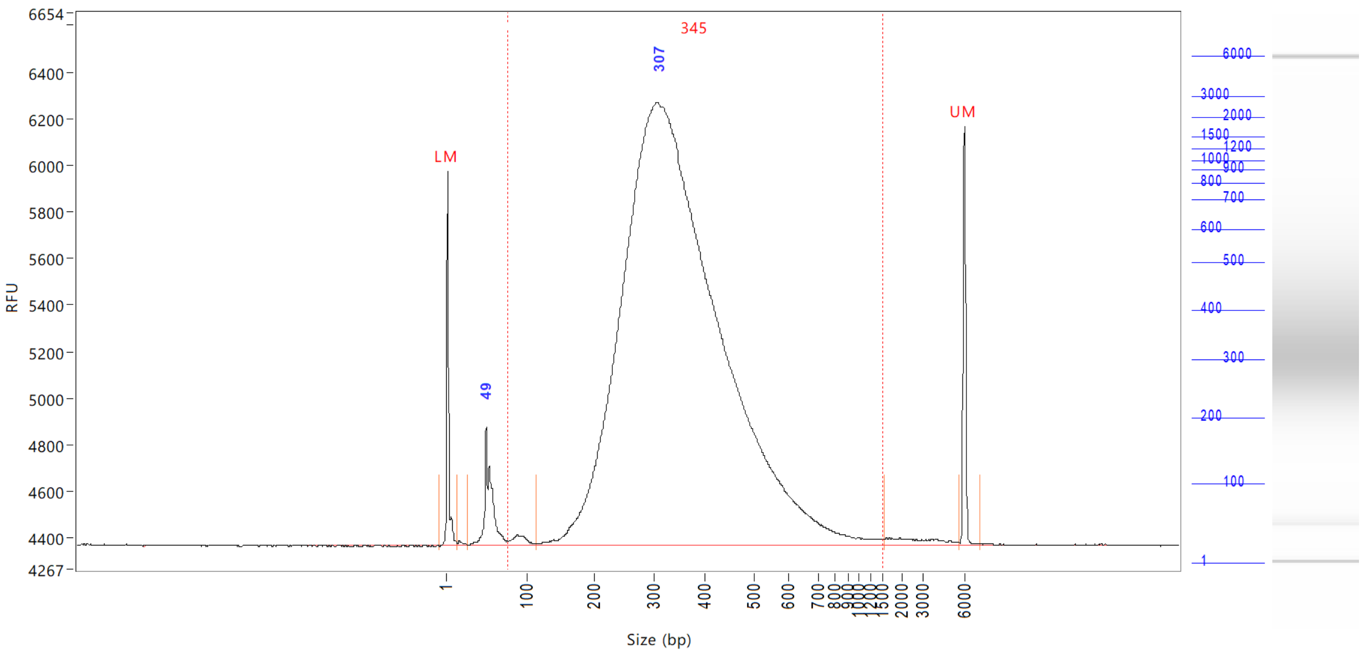

Peak	Size (bp)	Conc. (ng/uL)	From (bp)	To (bp)	RFU
1	1 (LM)	0.0124	0	14	1603
2	49	0.1714	26	113	508
3	307	5.4905	113	1554	1904
4	6000 (UM)	0.0082	5670	7137	1799

TIC:	5.6619	ng/uL
TIM:	30.9484	nmole/L
Total Conc.:	5.6993	ng/uL

Smear Analysis 75 bp to 1500 bp 5.5147 ng/ul 96.8 %Total 26.3096 nmole/L 345 Avg. Size (b.p.) 32.86 %CV

```
Sample Peak Width (sec): 50   Sample Min Peak Height: 25    Sample Baseline V to V?: Y    Sample Baseline V to V pts: 3
Sample Filter: Binomial       # of Pts for Filter: 3        Sample Start Region (min): 0  Sample End Region (min): 50
Manual Baseline Start (min): 10     Manual Baseline End (min): 48
Marker Peak Width (sec): 5    Marker Min Peak Height: 200   Marker Baseline V to V?: Y    Marker Baseline V to V pts: 3
Lower Marker Selection: First Peak > 200 RFU                Upper Marker Selection: Last Peak > 200 RFU
Ladder Size (bp): 1, 100, 200, 300, 400, 500, 600, 700, 800, 900, 1000, 1200, 1500, 2000, 3000, 6000
Quantification Using: Ladder          Final Concentration (ng/uL): 0.0830          Dilution Factor: 12.0
```

 Copyright 2015 Advanced Analytical Technologies, Inc.

Sample: 103613-001-025
Well Location: A4
Created: Friday, June 21, 2019 2:11:36 PM

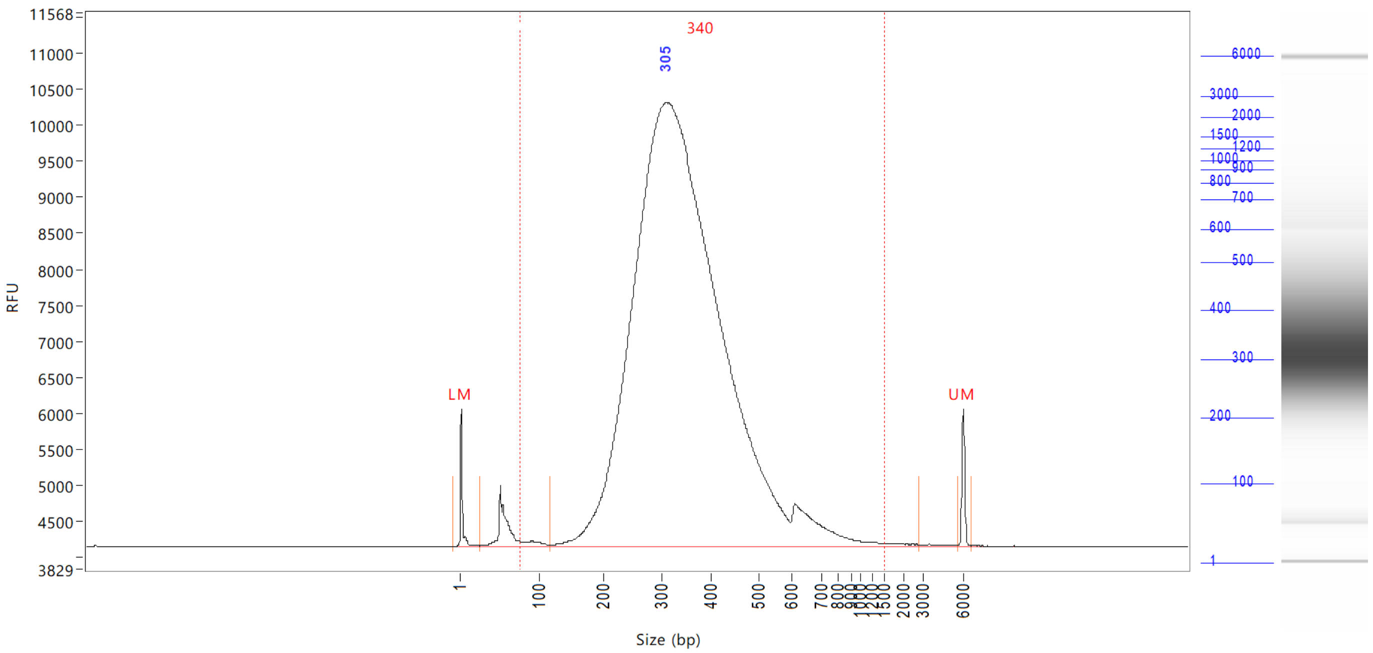

Peak	Size (bp)	Conc. (ng/uL)	From (bp)	To (bp)	RFU
1	1 (LM)	0.0124	0	26	1904
2	305	13.5275	118	2800	6187
3	6000 (UM)	0.0077	5619	6556	1914
	TIC:	13.5275	ng/uL		
	TIM:	64.8815	nmole/L		
	Total Conc.:	13.8100	ng/uL		

Smear Analysis 75 bp to 1500 bp 13.5571 ng/ul 98.2 %Total 65.5244 nmole/L 340 Avg. Size (b.p.) 30.34 %CV

Sample Peak Width (sec): 50 Sample Min Peak Height: 25 Sample Baseline V to V?: Y Sample Baseline V to V pts: 3
Sample Filter: Binomial # of Pts for Filter: 3 Sample Start Region (min): 0 Sample End Region (min): 50
Manual Baseline Start (min): 10 Manual Baseline End (min): 48
Marker Peak Width (sec): 5 Marker Min Peak Height: 200 Marker Baseline V to V?: Y Marker Baseline V to V pts: 3
Lower Marker Selection: First Peak > 200 RFU Upper Marker Selection: Last Peak > 200 RFU
Ladder Size (bp): 1, 100, 200, 300, 400, 500, 600, 700, 800, 900, 1000, 1200, 1500, 2000, 3000, 6000
Quantification Using: Ladder Final Concentration (ng/uL): 0.0830 Dilution Factor: 12.0

 Copyright 2015 Advanced Analytical Technologies, Inc.

Sample: 103613-001-026
Well Location: B4
Created: Friday, June 21, 2019 2:11:36 PM

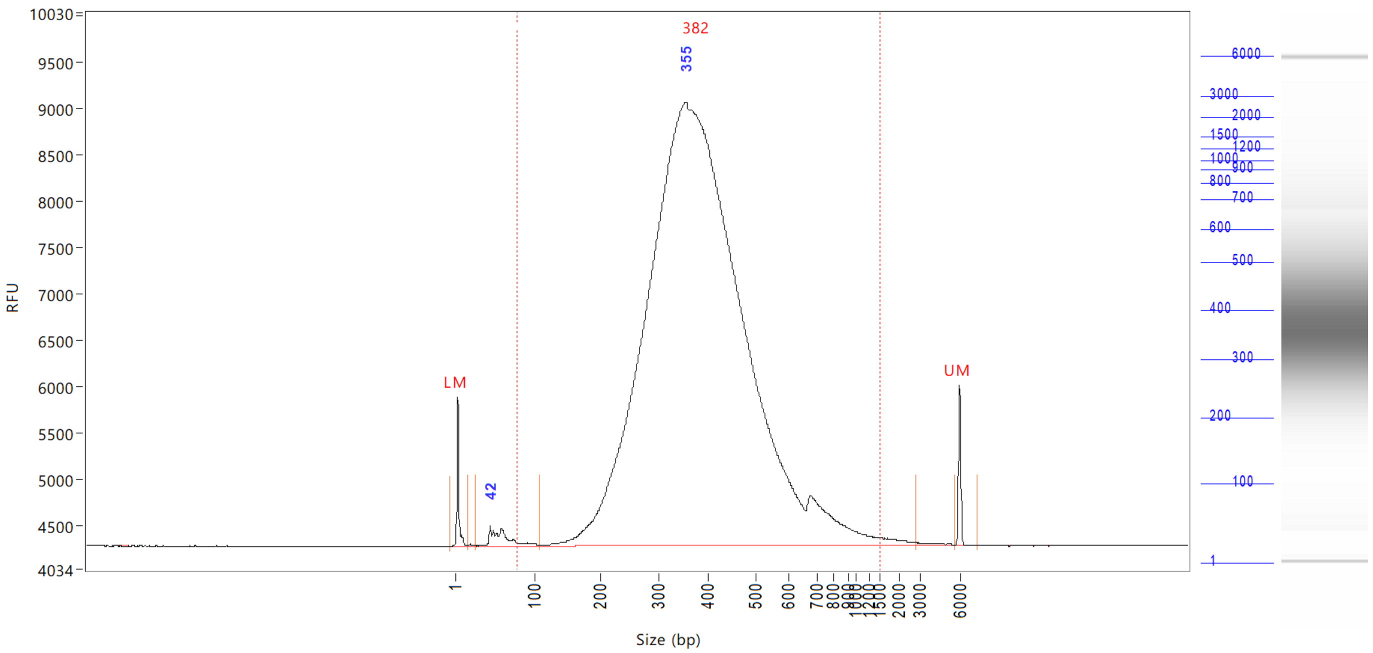

Peak	Size (bp)	Conc. (ng/uL)	From (bp)	To (bp)	RFU
1	1 (LM)	0.0124	0	15	1604
2	42	0.1406	25	108	215
3	355	13.7628	108	2817	4785
4	6000 (UM)	0.0078	5670	7314	1721

TIC:	13.9033	ng/uL
TIM:	62.5661	nmole/L
Total Conc.:	13.9188	ng/uL

Smear Analysis	75 bp to 1500 bp	13.7441 ng/ul	98.7 %Total	59.2654 nmole/L	382 Avg. Size (b.p.)	31.42 %CV

Sample Peak Width (sec): 50 Sample Min Peak Height: 25 Sample Baseline V to V?: Y Sample Baseline V to V pts: 3
Sample Filter: Binomial # of Pts for Filter: 3 Sample Start Region (min): 0 Sample End Region (min): 50
Manual Baseline Start (min): 10 Manual Baseline End (min): 48
Marker Peak Width (sec): 5 Marker Min Peak Height: 200 Marker Baseline V to V?: Y Marker Baseline V to V pts: 3
Lower Marker Selection: First Peak > 200 RFU Upper Marker Selection: Last Peak > 200 RFU
Ladder Size (bp): 1, 100, 200, 300, 400, 500, 600, 700, 800, 900, 1000, 1200, 1500, 2000, 3000, 6000
Quantification Using: Ladder Final Concentration (ng/uL): 0.0830 Dilution Factor: 12.0

 Copyright 2015 Advanced Analytical Technologies, Inc.

Sample: 103613-001-027
Well Location: C4
Created: Friday, June 21, 2019 2:11:36 PM

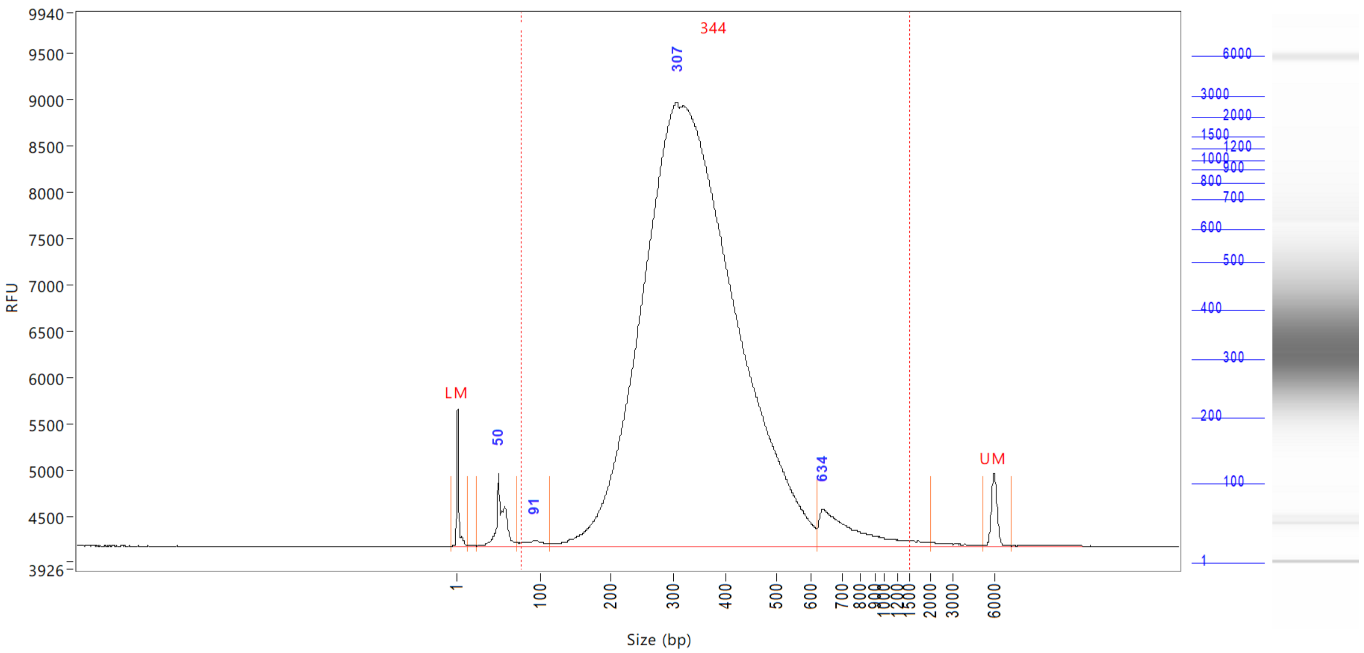

Peak	Size (bp)	Conc. (ng/uL)	From (bp)	To (bp)	RFU
1	1 (LM)	0.0124	0	15	1480
2	50	0.2453	23	72	788
3	91	0.0489	72	112	57
4	307	13.9253	112	618	4805
5	634	0.3637	618	2018	403
6	6000 (UM)	0.0081	5187	7212	790

TIC:	14.5831	ng/uL
TIM:	77.9307	nmole/L
Total Conc.:	14.6074	ng/uL

Smear Analysis 75 bp to 1500 bp 14.3116 ng/ul 98.0 %Total 68.5172 nmole/L 344 Avg. Size (b.p.) 31.61 %CV

Sample Peak Width (sec): 50 Sample Min Peak Height: 25 Sample Baseline V to V?: Y Sample Baseline V to V pts: 3
Sample Filter: Binomial # of Pts for Filter: 3 Sample Start Region (min): 0 Sample End Region (min): 50
Manual Baseline Start (min): 10 Manual Baseline End (min): 48
Marker Peak Width (sec): 5 Marker Min Peak Height: 200 Marker Baseline V to V?: Y Marker Baseline V to V pts: 3
Lower Marker Selection: First Peak > 200 RFU Upper Marker Selection: Last Peak > 200 RFU
Ladder Size (bp): 1, 100, 200, 300, 400, 500, 600, 700, 800, 900, 1000, 1200, 1500, 2000, 3000, 6000
Quantification Using: Ladder Final Concentration (ng/uL): 0.0830 Dilution Factor: 12.0

Copyright 2015 Advanced Analytical Technologies, Inc.

Sample: 103613-001-028
Well Location: D4
Created: Friday, June 21, 2019 2:11:36 PM

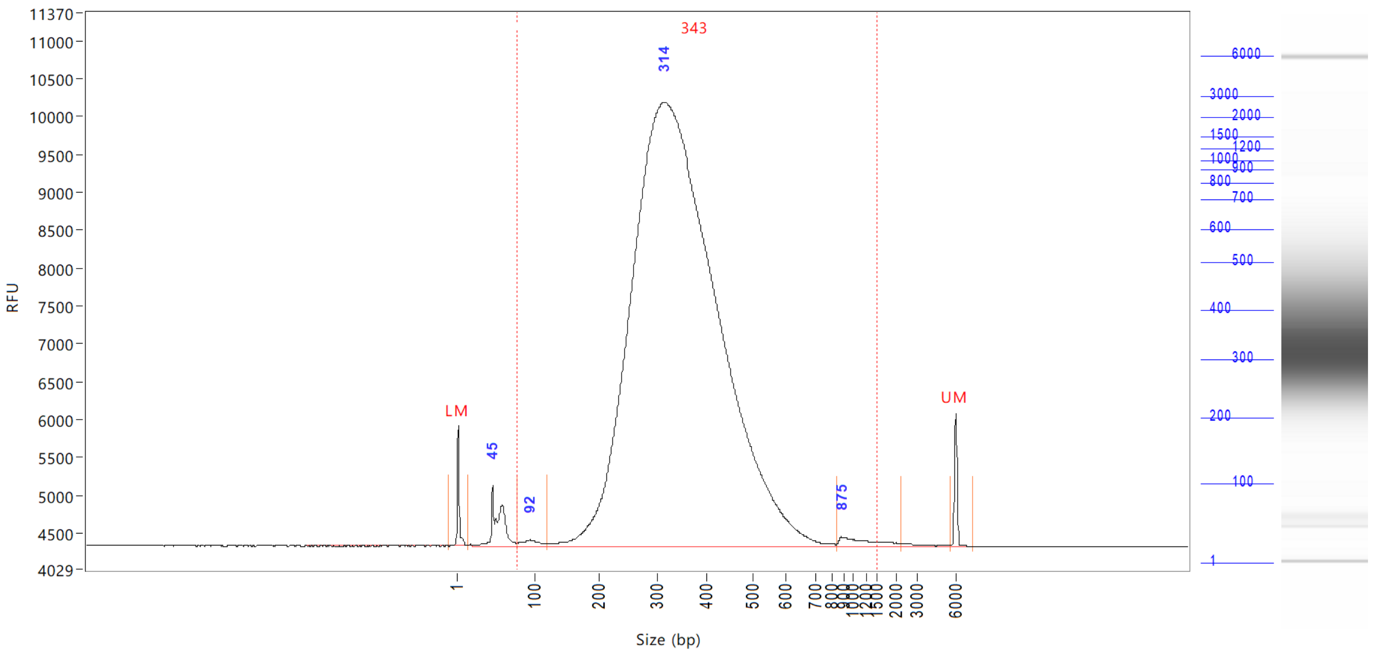

Peak	Size (bp)	Conc. (ng/uL)	From (bp)	To (bp)	RFU
1	1 (LM)	0.0124	0	15	1588
2	45	0.3057	15	78	800
3	92	0.0550	78	120	80
4	314	15.5094	120	839	5863
5	875	0.0859	839	2284	122
6	6000 (UM)	0.0082	5644	7314	1753

TIC:	15.9560	ng/uL
TIM:	85.3360	nmole/L
Total Conc.:	15.9689	ng/uL

Smear Analysis 75 bp to 1500 bp 15.6301 ng/ul 97.9 %Total 74.9245 nmole/L 343 Avg. Size (b.p.) 28.18 %CV

Sample Peak Width (sec): 50 Sample Min Peak Height: 25 Sample Baseline V to V?: Y Sample Baseline V to V pts: 3
Sample Filter: Binomial # of Pts for Filter: 3 Sample Start Region (min): 0 Sample End Region (min): 50
Manual Baseline Start (min): 10 Manual Baseline End (min): 48
Marker Peak Width (sec): 5 Marker Min Peak Height: 200 Marker Baseline V to V?: Y Marker Baseline V to V pts: 3
Lower Marker Selection: First Peak > 200 RFU Upper Marker Selection: Last Peak > 200 RFU
Ladder Size (bp): 1, 100, 200, 300, 400, 500, 600, 700, 800, 900, 1000, 1200, 1500, 2000, 3000, 6000
Quantification Using: Ladder Final Concentration (ng/uL): 0.0830 Dilution Factor: 12.0

Sample: 103613-001-029
Well Location: E4
Created: Friday, June 21, 2019 2:11:36 PM

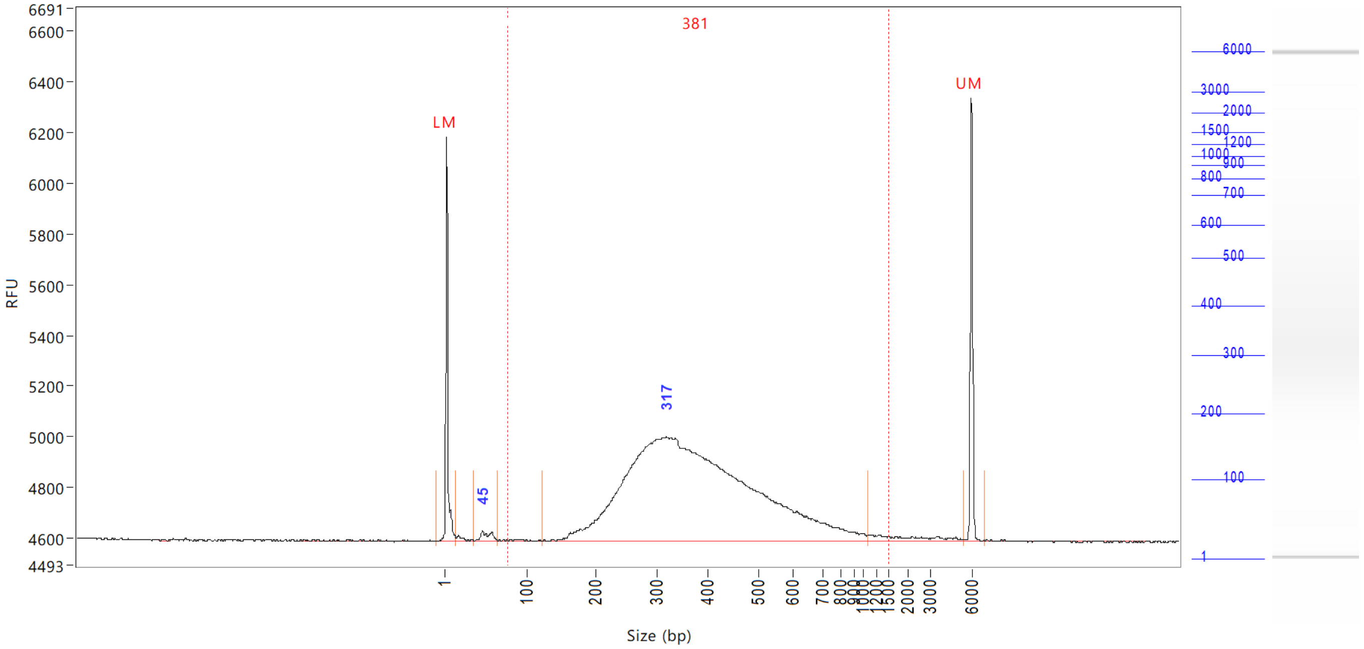

Peak	Size (bp)	Conc. (ng/uL)	From (bp)	To (bp)	RFU
1	1 (LM)	0.0124	0	13	1595
2	45	0.0160	35	64	39
3	317	1.4652	122	1073	410
4	6000 (UM)	0.0078	5441	6985	1753

TIC:	1.4813	ng/uL
TIM:	6.9195	nmole/L
Total Conc.:	1.5152	ng/uL

Smear Analysis 75 bp to 1500 bp 1.4755 ng/ul 97.4 %Total 6.3775 nmole/L 381 Avg. Size (b.p.) 38.86 %CV

```
Sample Peak Width (sec): 50    Sample Min Peak Height: 25    Sample Baseline V to V?: Y    Sample Baseline V to V pts: 3
Sample Filter: Binomial        # of Pts for Filter: 3        Sample Start Region (min): 0  Sample End Region (min): 50
Manual Baseline Start (min): 10     Manual Baseline End (min): 48
Marker Peak Width (sec): 5     Marker Min Peak Height: 200   Marker Baseline V to V?: Y    Marker Baseline V to V pts: 3
Lower Marker Selection: First Peak > 200 RFU                 Upper Marker Selection: Last Peak > 200 RFU
Ladder Size (bp): 1, 100, 200, 300, 400, 500, 600, 700, 800, 900, 1000, 1200, 1500, 2000, 3000, 6000
Quantification Using: Ladder          Final Concentration (ng/uL): 0.0830          Dilution Factor: 12.0
```


Copyright 2015 Advanced Analytical Technologies, Inc.

Sample: 103613-001-030
Well Location: F4
Created: Friday, June 21, 2019 2:11:36 PM

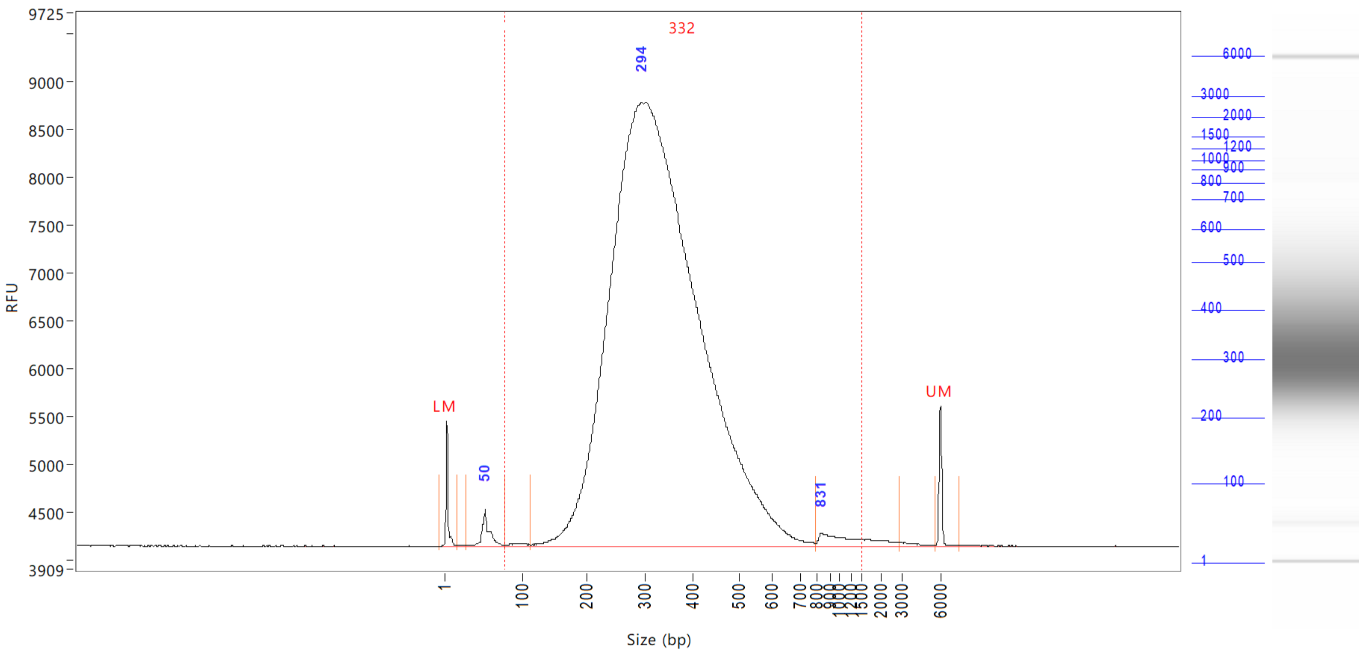

Peak	Size (bp)	Conc. (ng/uL)	From (bp)	To (bp)	RFU
1	1 (LM)	0.0124	0	15	1313
2	50	0.1378	28	76	389
3	294	15.5056	112	790	4646
4	831	0.1567	790	2950	134
5	6000 (UM)	0.0088	5670	7440	1474

TIC:	15.8001	ng/uL
TIM:	82.3007	nmole/L
Total Conc.:	15.8446	ng/uL

Smear Analysis 75 bp to 1500 bp 15.6345 ng/ul 98.7 %Total 77.3686 nmole/L 332 Avg. Size (b.p.) 31.44 %CV

Sample Peak Width (sec): 50 Sample Min Peak Height: 25 Sample Baseline V to V?: Y Sample Baseline V to V pts: 3
Sample Filter: Binomial # of Pts for Filter: 3 Sample Start Region (min): 0 Sample End Region (min): 50
Manual Baseline Start (min): 10 Manual Baseline End (min): 48
Marker Peak Width (sec): 5 Marker Min Peak Height: 200 Marker Baseline V to V?: Y Marker Baseline V to V pts: 3
Lower Marker Selection: First Peak > 200 RFU Upper Marker Selection: Last Peak > 200 RFU
Ladder Size (bp): 1, 100, 200, 300, 400, 500, 600, 700, 800, 900, 1000, 1200, 1500, 2000, 3000, 6000
Quantification Using: Ladder Final Concentration (ng/uL): 0.0830 Dilution Factor: 12.0

 Copyright 2015 Advanced Analytical Technologies, Inc.

Sample: 103613-001-031
Well Location: G4
Created: Friday, June 21, 2019 2:11:36 PM

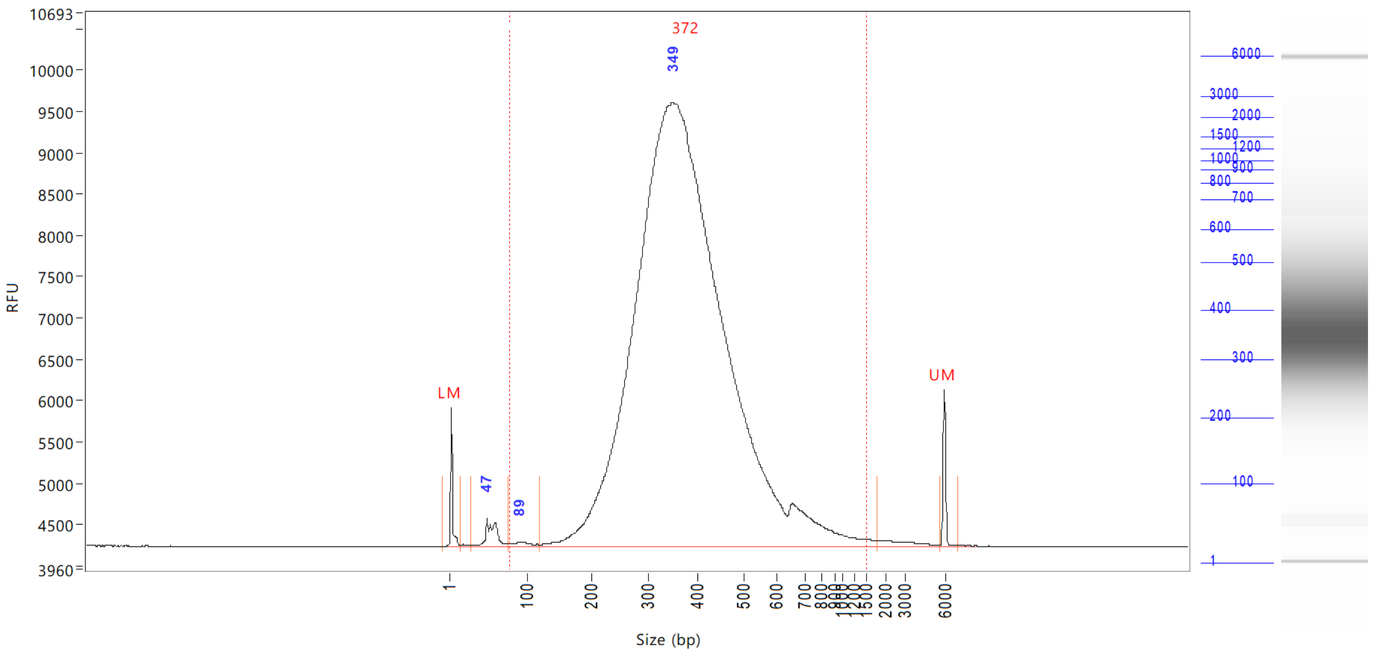

Peak	Size (bp)	Conc. (ng/uL)	From (bp)	To (bp)	RFU
1	1 (LM)	0.0124	0	14	1687
2	47	0.1357	28	74	343
3	89	0.0373	74	119	51
4	349	13.4853	119	1786	5381
5	6000 (UM)	0.0084	5670	7036	1892

TIC:	13.6582	ng/uL
TIM:	64.1725	nmole/L
Total Conc.:	13.7133	ng/uL

Smear Analysis 75 bp to 1500 bp 13.5056 ng/ul 98.5 %Total 59.7031 nmole/L 372 Avg. Size (b.p.) 30.96 %CV

Sample Peak Width (sec): 50 Sample Min Peak Height: 25 Sample Baseline V to V?: Y Sample Baseline V to V pts: 3
Sample Filter: Binomial # of Pts for Filter: 3 Sample Start Region (min): 0 Sample End Region (min): 50
Manual Baseline Start (min): 10 Manual Baseline End (min): 48
Marker Peak Width (sec): 5 Marker Min Peak Height: 200 Marker Baseline V to V?: Y Marker Baseline V to V pts: 3
Lower Marker Selection: First Peak > 200 RFU Upper Marker Selection: Last Peak > 200 RFU
Ladder Size (bp): 1, 100, 200, 300, 400, 500, 600, 700, 800, 900, 1000, 1200, 1500, 2000, 3000, 6000
Quantification Using: Ladder Final Concentration (ng/uL): 0.0830 Dilution Factor: 12.0

Copyright 2015 Advanced Analytical Technologies, Inc.

Sample: 103613-001-032
Well Location: H4
Created: Friday, June 21, 2019 2:11:36 PM

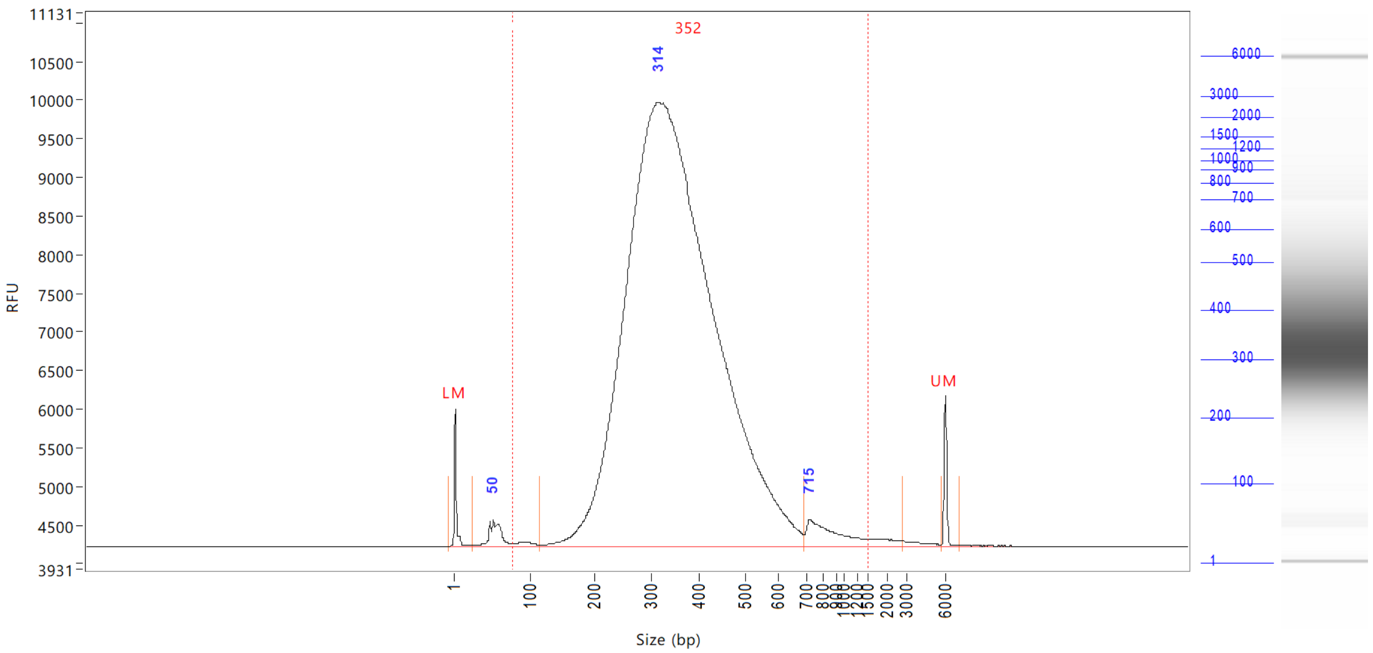

Peak	Size (bp)	Conc. (ng/uL)	From (bp)	To (bp)	RFU
1	1 (LM)	0.0124	0	25	1769
2	50	0.1864	25	114	340
3	314	13.7736	114	691	5754
4	715	0.2702	691	2800	343
5	6000 (UM)	0.0083	5670	7010	1959

TIC:	14.2303	ng/uL
TIM:	71.3674	nmole/L
Total Conc.:	14.2572	ng/uL

Smear Analysis 75 bp to 1500 bp 14.0271 ng/ul 98.4 %Total 65.5659 nmole/L 352 Avg. Size (b.p.) 32.13 %CV

```
Sample Peak Width (sec): 50   Sample Min Peak Height: 25    Sample Baseline V to V?: Y    Sample Baseline V to V pts: 3
Sample Filter: Binomial       # of Pts for Filter: 3        Sample Start Region (min): 0  Sample End Region (min): 50
Manual Baseline Start (min): 10     Manual Baseline End (min): 48
Marker Peak Width (sec): 5    Marker Min Peak Height: 200   Marker Baseline V to V?: Y    Marker Baseline V to V pts: 3
Lower Marker Selection: First Peak > 200 RFU                Upper Marker Selection: Last Peak > 200 RFU
Ladder Size (bp): 1, 100, 200, 300, 400, 500, 600, 700, 800, 900, 1000, 1200, 1500, 2000, 3000, 6000
Quantification Using: Ladder          Final Concentration (ng/uL): 0.0830          Dilution Factor: 12.0
```


Copyright 2015 Advanced Analytical Technologies, Inc.

Sample: 103613-001-033
Well Location: A5
Created: Friday, June 21, 2019 2:11:36 PM

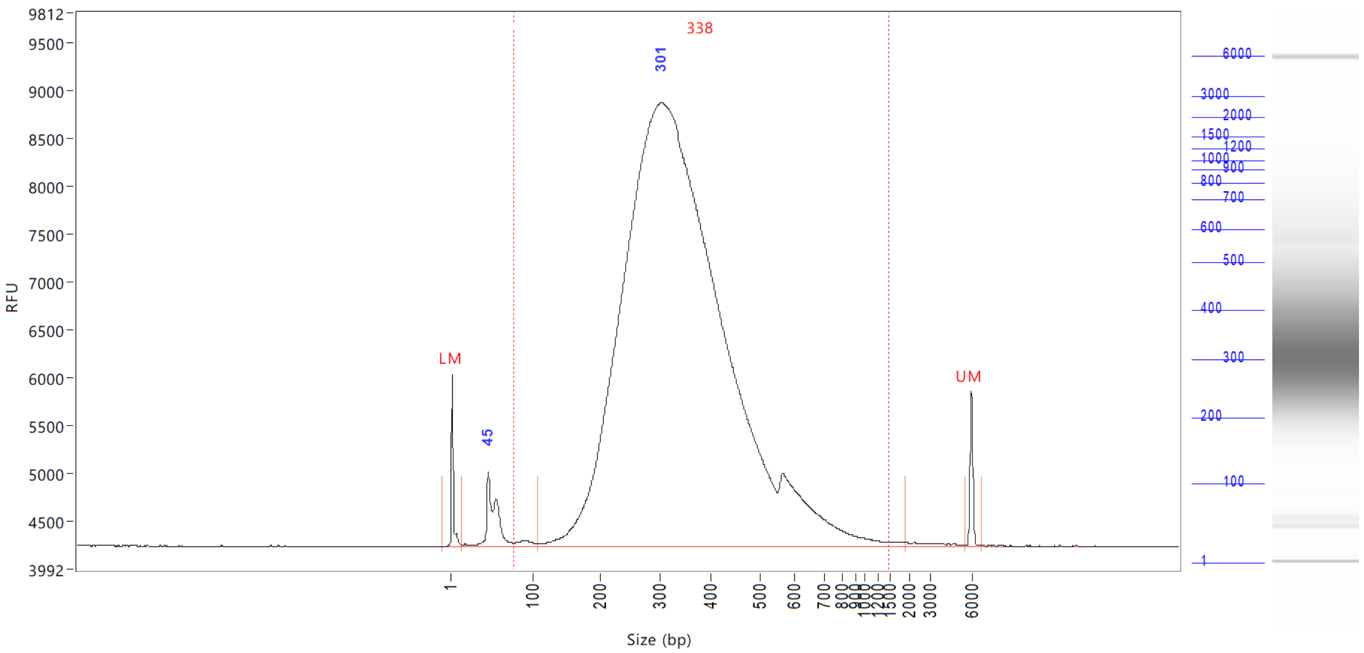

Peak	Size (bp)	Conc. (ng/uL)	From (bp)	To (bp)	RFU
1	1 (LM)	0.0124	0	15	1796
2	45	0.3060	15	109	774
3	301	12.6586	109	1902	4649
4	6000 (UM)	0.0078	5517	6733	1627

TIC:	12.9646	ng/uL
TIM:	70.5476	nmole/L
Total Conc.:	12.9903	ng/uL

Smear Analysis	75 bp to 1500 bp	12.6814 ng/ul	97.6 %Total	61.7849 nmole/L	338 Avg. Size (b.p.)	33.28 %CV

Sample Peak Width (sec): 50   Sample Min Peak Height: 25   Sample Baseline V to V?: Y   Sample Baseline V to V pts: 3
Sample Filter: Binomial   # of Pts for Filter: 3   Sample Start Region (min): 0   Sample End Region (min): 50
Manual Baseline Start (min): 10   Manual Baseline End (min): 48
Marker Peak Width (sec): 5   Marker Min Peak Height: 200   Marker Baseline V to V?: Y   Marker Baseline V to V pts: 3
Lower Marker Selection: First Peak > 200 RFU   Upper Marker Selection: Last Peak > 200 RFU
Ladder Size (bp): 1, 100, 200, 300, 400, 500, 600, 700, 800, 900, 1000, 1200, 1500, 2000, 3000, 6000
Quantification Using: Ladder   Final Concentration (ng/uL): 0.0830   Dilution Factor: 12.0

Copyright 2015 Advanced Analytical Technologies, Inc.

Sample: 103613-001-034
Well Location: B5
Created: Friday, June 21, 2019 2:11:36 PM

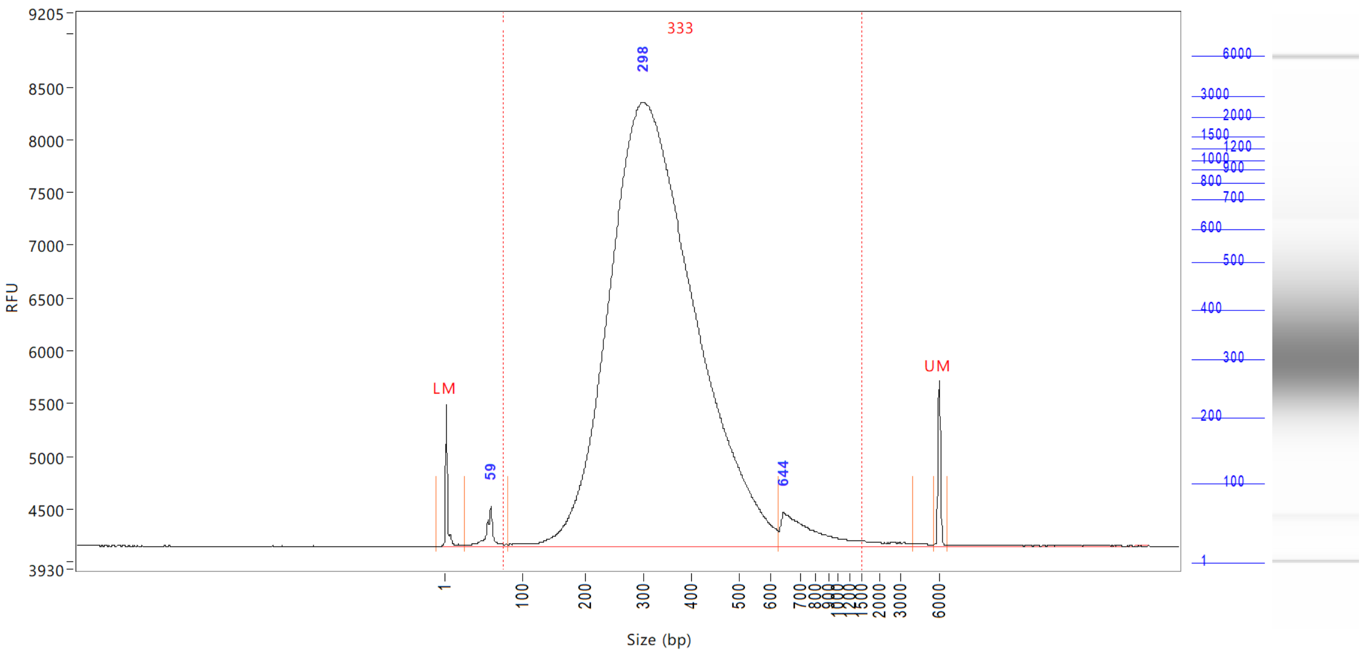

Peak	Size (bp)	Conc. (ng/uL)	From (bp)	To (bp)	RFU
1	1 (LM)	0.0124	0	26	1340
2	59	0.1187	26	81	375
3	298	12.7700	81	625	4215
4	644	0.3218	625	3992	323
5	6000 (UM)	0.0084	5543	6657	1569

TIC:	13.2105	ng/uL
TIM:	69.0622	nmole/L
Total Conc.:	13.2168	ng/uL

Smear Analysis 75 bp to 1500 bp 13.0564 ng/ul 98.8 %Total 64.4834 nmole/L 333 Avg. Size (b.p.) 32.40 %CV

```
Sample Peak Width (sec): 50   Sample Min Peak Height: 25    Sample Baseline V to V?: Y    Sample Baseline V to V pts: 3
Sample Filter: Binomial       # of Pts for Filter: 3        Sample Start Region (min): 0  Sample End Region (min): 50
Manual Baseline Start (min): 10     Manual Baseline End (min): 48
Marker Peak Width (sec): 5    Marker Min Peak Height: 200   Marker Baseline V to V?: Y    Marker Baseline V to V pts: 3
Lower Marker Selection: First Peak > 200 RFU                Upper Marker Selection: Last Peak > 200 RFU
Ladder Size (bp): 1, 100, 200, 300, 400, 500, 600, 700, 800, 900, 1000, 1200, 1500, 2000, 3000, 6000
Quantification Using: Ladder          Final Concentration (ng/uL): 0.0830          Dilution Factor: 12.0
```

Sample: 103613-001-035
Well Location: C5
Created: Friday, June 21, 2019 2:11:36 PM

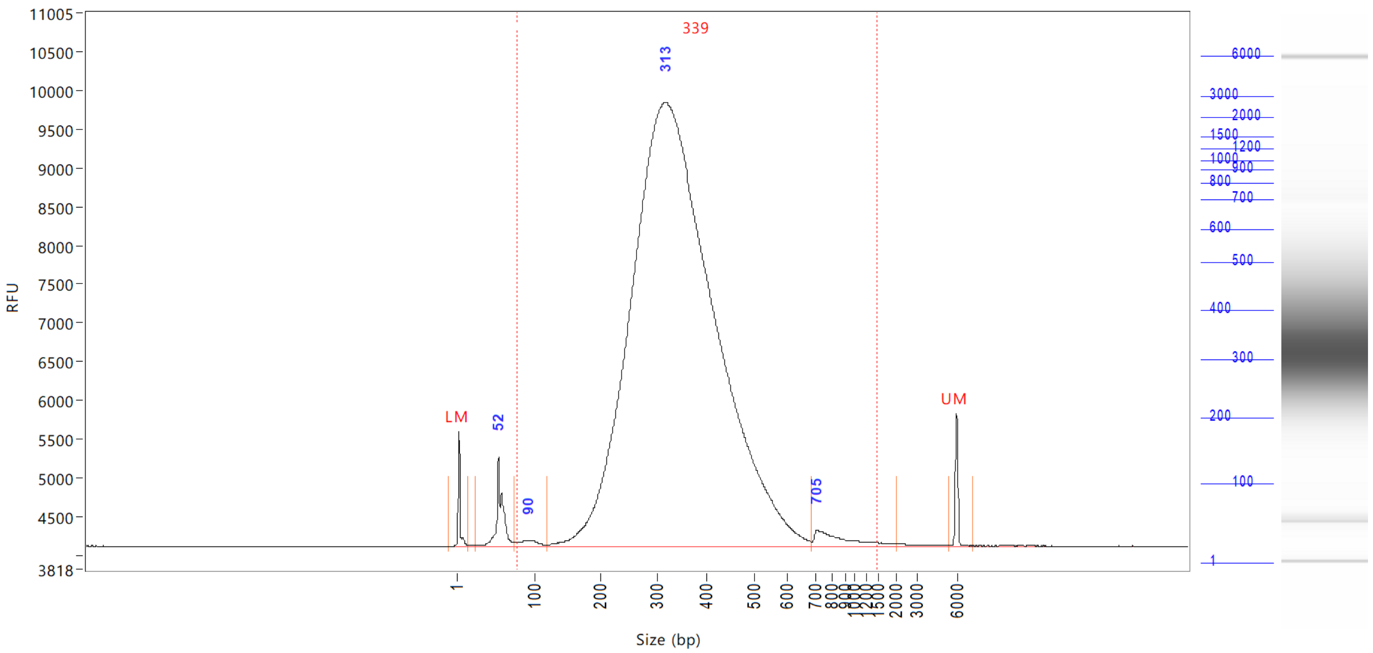

Peak	Size (bp)	Conc. (ng/uL)	From (bp)	To (bp)	RFU
1	1 (LM)	0.0124	0	15	1479
2	52	0.3087	24	73	1148
3	90	0.0639	73	118	74
4	313	15.5073	118	687	5744
5	705	0.1616	687	2001	214
6	6000 (UM)	0.0085	5466	7288	1711

TIC:	16.0415	ng/uL
TIM:	86.9515	nmole/L
Total Conc.:	16.0631	ng/uL

Smear Analysis 75 bp to 1500 bp 15.7135 ng/ul 97.8 %Total 76.2512 nmole/L 339 Avg. Size (b.p.) 29.22 %CV

Sample Peak Width (sec): 50 Sample Min Peak Height: 25 Sample Baseline V to V?: Y Sample Baseline V to V pts: 3
Sample Filter: Binomial # of Pts for Filter: 3 Sample Start Region (min): 0 Sample End Region (min): 50
Manual Baseline Start (min): 10 Manual Baseline End (min): 48
Marker Peak Width (sec): 5 Marker Min Peak Height: 200 Marker Baseline V to V?: Y Marker Baseline V to V pts: 3
Lower Marker Selection: First Peak > 200 RFU Upper Marker Selection: Last Peak > 200 RFU
Ladder Size (bp): 1, 100, 200, 300, 400, 500, 600, 700, 800, 900, 1000, 1200, 1500, 2000, 3000, 6000
Quantification Using: Ladder Final Concentration (ng/uL): 0.0830 Dilution Factor: 12.0

Copyright 2015 Advanced Analytical Technologies, Inc.

Sample: 103613-001-036
Well Location: D5
Created: Friday, June 21, 2019 2:11:36 PM

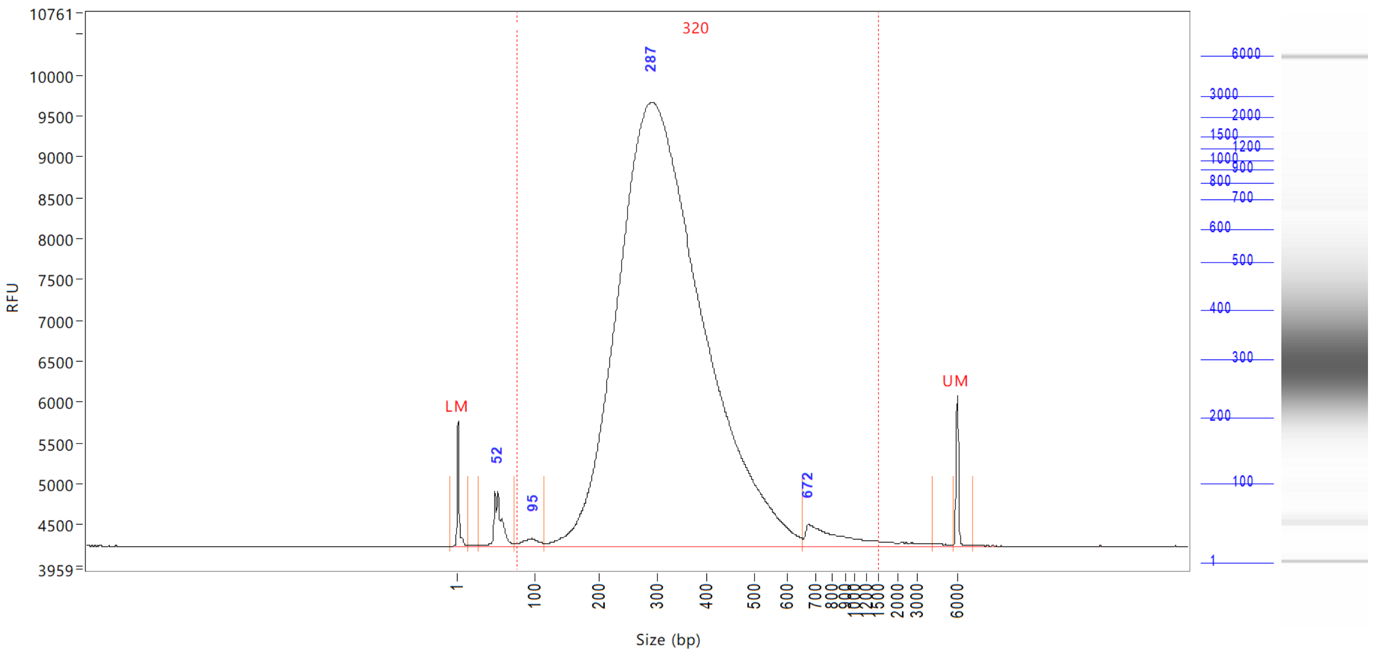

Peak	Size (bp)	Conc. (ng/uL)	From (bp)	To (bp)	RFU
1	1 (LM)	0.0124	0	15	1536
2	52	0.2259	29	73	676
3	95	0.0679	73	114	97
4	287	15.0670	114	655	5435
5	672	0.2621	655	4145	266
6	6000 (UM)	0.0091	5670	7162	1833

TIC:	15.6229	ng/uL
TIM:	87.5097	nmole/L
Total Conc.:	15.6350	ng/uL

Smear Analysis 75 bp to 1500 bp 15.3500 ng/ul 98.2 %Total 78.8659 nmole/L 320 Avg. Size (b.p.) 32.82 %CV

Sample Peak Width (sec): 50 Sample Min Peak Height: 25 Sample Baseline V to V?: Y Sample Baseline V to V pts: 3
Sample Filter: Binomial # of Pts for Filter: 3 Sample Start Region (min): 0 Sample End Region (min): 50
Manual Baseline Start (min): 10 Manual Baseline End (min): 48
Marker Peak Width (sec): 5 Marker Min Peak Height: 200 Marker Baseline V to V?: Y Marker Baseline V to V pts: 3
Lower Marker Selection: First Peak > 200 RFU Upper Marker Selection: Last Peak > 200 RFU
Ladder Size (bp): 1, 100, 200, 300, 400, 500, 600, 700, 800, 900, 1000, 1200, 1500, 2000, 3000, 6000
Quantification Using: Ladder Final Concentration (ng/uL): 0.0830 Dilution Factor: 12.0

 Copyright 2015 Advanced Analytical Technologies, Inc.

Sample: 103613-001-037
Well Location: E5
Created: Friday, June 21, 2019 2:11:36 PM

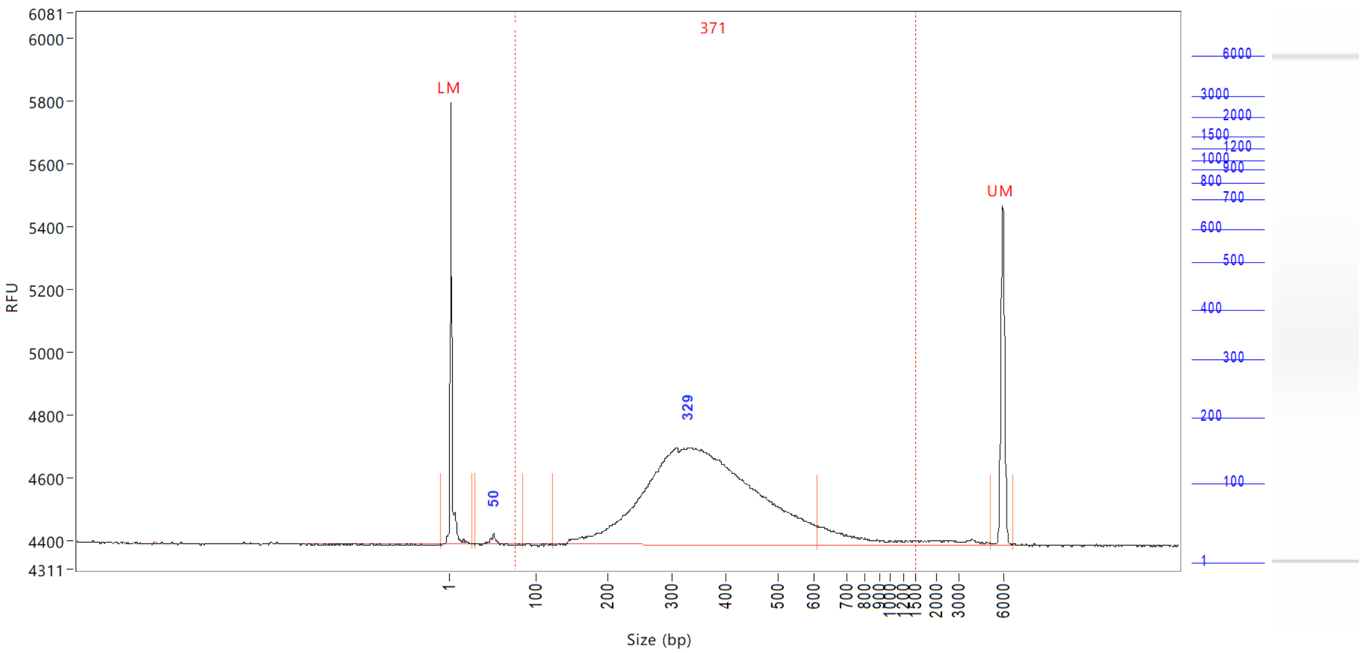

Peak	Size (bp)	Conc. (ng/uL)	From (bp)	To (bp)	RFU
1	1 (LM)	0.0124	0	26	1407
2	50	0.0070	29	84	33
3	329	1.0986	124	609	309
4	6000 (UM)	0.0074	5187	6707	1081
	TIC:	1.1055	ng/uL		
	TIM:	5.3528	nmole/L		
	Total Conc.:	1.1749	ng/uL		

Smear Analysis 75 bp to 1500 bp 1.1505 ng/ul 97.9 %Total 5.0965 nmole/L 371 Avg. Size (b.p.) 35.66 %CV

Sample Peak Width (sec): 50 Sample Min Peak Height: 25 Sample Baseline V to V?: Y Sample Baseline V to V pts: 3
Sample Filter: Binomial # of Pts for Filter: 3 Sample Start Region (min): 0 Sample End Region (min): 50
Manual Baseline Start (min): 10 Manual Baseline End (min): 48
Marker Peak Width (sec): 5 Marker Min Peak Height: 200 Marker Baseline V to V?: Y Marker Baseline V to V pts: 3
Lower Marker Selection: First Peak > 200 RFU Upper Marker Selection: Last Peak > 200 RFU
Ladder Size (bp): 1, 100, 200, 300, 400, 500, 600, 700, 800, 900, 1000, 1200, 1500, 2000, 3000, 6000
Quantification Using: Ladder Final Concentration (ng/uL): 0.0830 Dilution Factor: 12.0

Copyright 2015 Advanced Analytical Technologies, Inc.

Sample: 103613-001-038
Well Location: F5
Created: Friday, June 21, 2019 2:11:36 PM

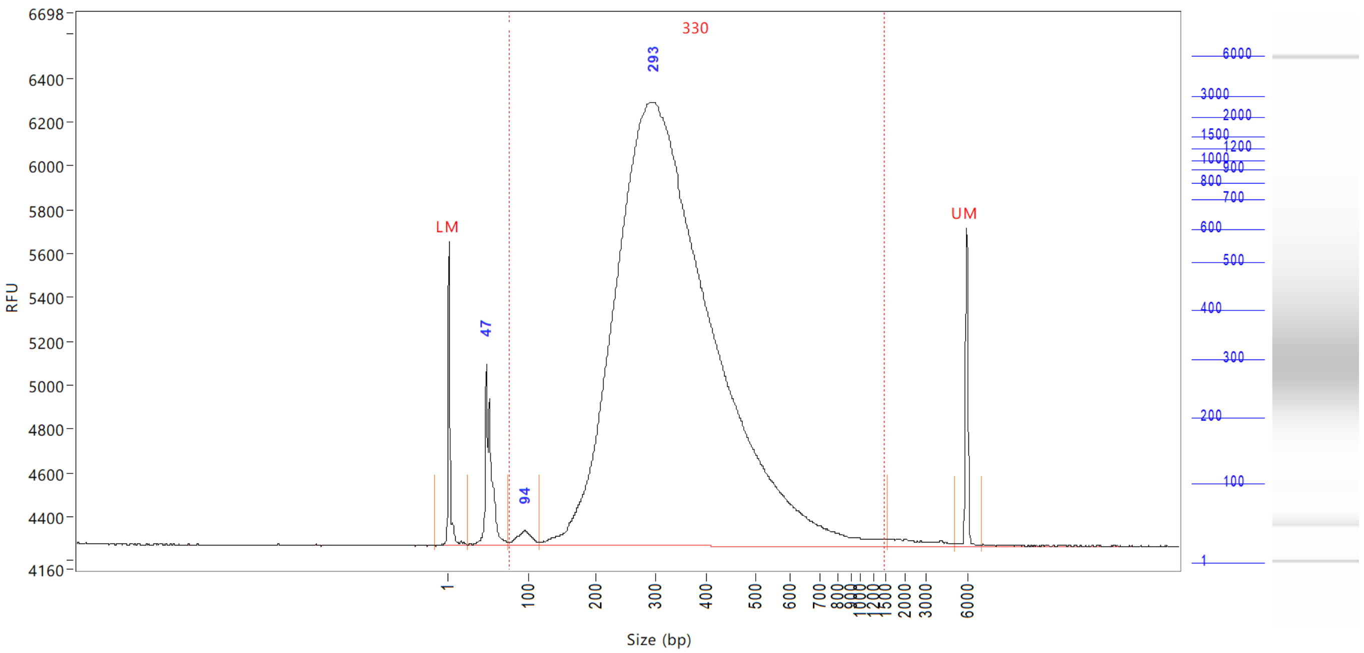

Peak	Size (bp)	Conc. (ng/uL)	From (bp)	To (bp)	RFU
1	1 (LM)	0.0124	0	25	1388
2	47	0.2274	25	74	826
3	94	0.0467	74	116	70
4	293	6.4875	116	1581	2026
5	6000 (UM)	0.0074	5110	7111	1454

TIC:	6.7616	ng/uL
TIM:	40.2740	nmole/L
Total Conc.:	6.7903	ng/uL

Smear Analysis 75 bp to 1500 bp 6.5310 ng/ul 96.2 %Total 32.5523 nmole/L 330 Avg. Size (b.p.) 34.21 %CV

Sample Peak Width (sec): 50 Sample Min Peak Height: 25 Sample Baseline V to V?: Y Sample Baseline V to V pts: 3
Sample Filter: Binomial # of Pts for Filter: 3 Sample Start Region (min): 0 Sample End Region (min): 50
Manual Baseline Start (min): 10 Manual Baseline End (min): 48
Marker Peak Width (sec): 5 Marker Min Peak Height: 200 Marker Baseline V to V?: Y Marker Baseline V to V pts: 3
Lower Marker Selection: First Peak > 200 RFU Upper Marker Selection: Last Peak > 200 RFU
Ladder Size (bp): 1, 100, 200, 300, 400, 500, 600, 700, 800, 900, 1000, 1200, 1500, 2000, 3000, 6000
Quantification Using: Ladder Final Concentration (ng/uL): 0.0830 Dilution Factor: 12.0

 Copyright 2015 Advanced Analytical Technologies, Inc.

Sample: 103613-001-039
Well Location: G5
Created: Friday, June 21, 2019 2:11:36 PM

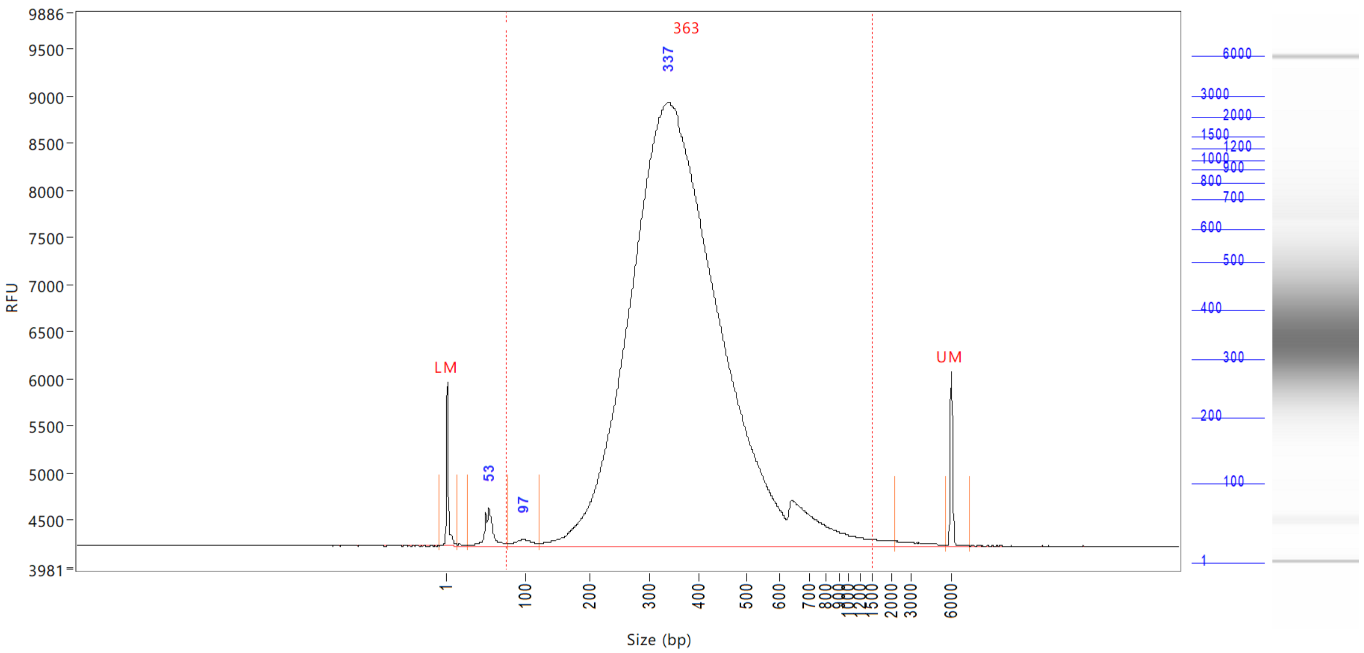

Peak	Size (bp)	Conc. (ng/uL)	From (bp)	To (bp)	RFU
1	1 (LM)	0.0124	0	14	1740
2	53	0.1273	27	78	398
3	97	0.0430	78	121	67
4	337	11.7220	121	2168	4717
5	6000 (UM)	0.0079	5619	7364	1861

TIC:	11.8923	ng/uL
TIM:	57.1299	nmole/L
Total Conc.:	11.9257	ng/uL

Smear Analysis 75 bp to 1500 bp 11.7403 ng/ul 98.4 %Total 53.2245 nmole/L 363 Avg. Size (b.p.) 31.98 %CV

Sample Peak Width (sec): 50 Sample Min Peak Height: 25 Sample Baseline V to V?: Y Sample Baseline V to V pts: 3
Sample Filter: Binomial # of Pts for Filter: 3 Sample Start Region (min): 0 Sample End Region (min): 50
Manual Baseline Start (min): 10 Manual Baseline End (min): 48
Marker Peak Width (sec): 5 Marker Min Peak Height: 200 Marker Baseline V to V?: Y Marker Baseline V to V pts: 3
Lower Marker Selection: First Peak > 200 RFU Upper Marker Selection: Last Peak > 200 RFU
Ladder Size (bp): 1, 100, 200, 300, 400, 500, 600, 700, 800, 900, 1000, 1200, 1500, 2000, 3000, 6000
Quantification Using: Ladder Final Concentration (ng/uL): 0.0830 Dilution Factor: 12.0

 Copyright 2015 Advanced Analytical Technologies, Inc.

Sample: 103613-001-040
Well Location: H5
Created: Friday, June 21, 2019 2:11:36 PM

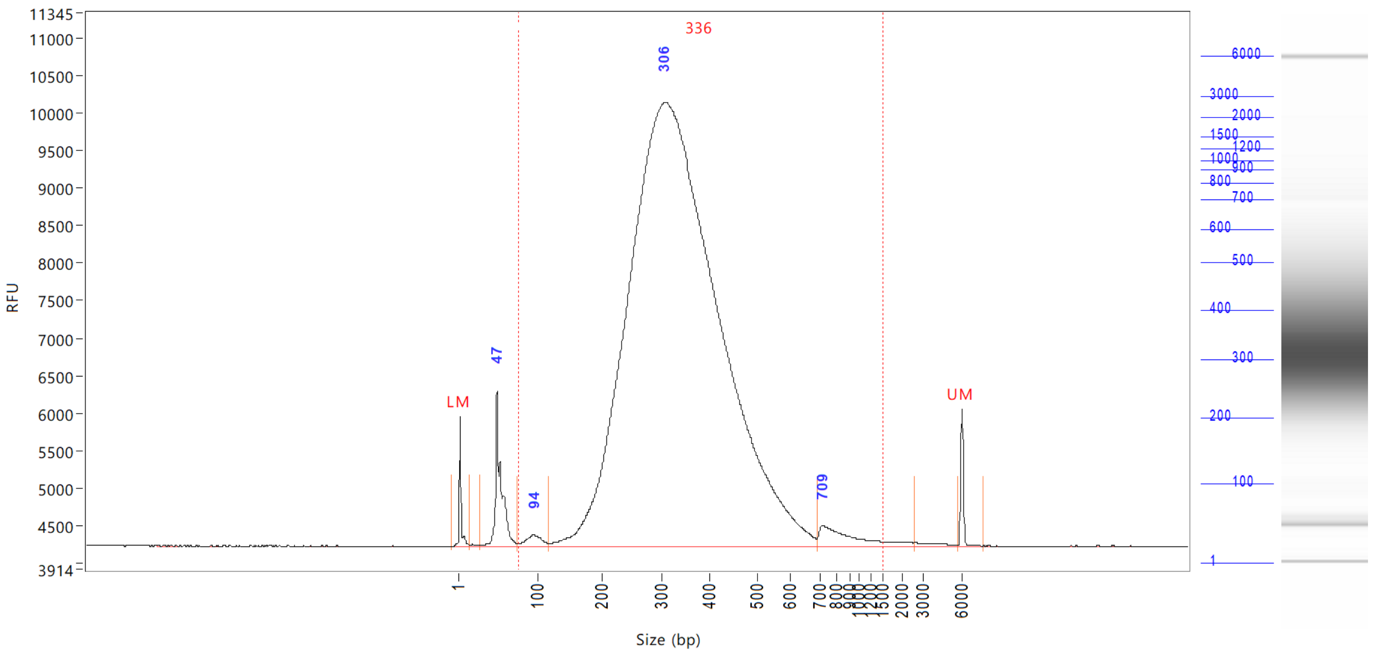

Peak	Size (bp)	Conc. (ng/uL)	From (bp)	To (bp)	RFU
1	1 (LM)	0.0124	0	15	1726
2	47	0.4499	27	75	2068
3	94	0.0837	75	116	150
4	306	14.8532	116	690	5936
5	709	0.2025	690	2634	278
6	6000 (UM)	0.0078	5670	7566	1834

TIC:	15.5893	ng/uL
TIM:	90.0548	nmole/L
Total Conc.:	15.6155	ng/uL

Smear Analysis 75 bp to 1500 bp 15.1077 ng/ul 96.7 %Total 74.0712 nmole/L 336 Avg. Size (b.p.) 31.69 %CV

Sample Peak Width (sec): 50 Sample Min Peak Height: 25 Sample Baseline V to V?: Y Sample Baseline V to V pts: 3
Sample Filter: Binomial # of Pts for Filter: 3 Sample Start Region (min): 0 Sample End Region (min): 50
Manual Baseline Start (min): 10 Manual Baseline End (min): 48
Marker Peak Width (sec): 5 Marker Min Peak Height: 200 Marker Baseline V to V?: Y Marker Baseline V to V pts: 3
Lower Marker Selection: First Peak > 200 RFU Upper Marker Selection: Last Peak > 200 RFU
Ladder Size (bp): 1, 100, 200, 300, 400, 500, 600, 700, 800, 900, 1000, 1200, 1500, 2000, 3000, 6000
Quantification Using: Ladder Final Concentration (ng/uL): 0.0830 Dilution Factor: 12.0

 Copyright 2015 Advanced Analytical Technologies, Inc.

Sample: 103613-001-041
Well Location: A6
Created: Friday, June 21, 2019 2:11:36 PM

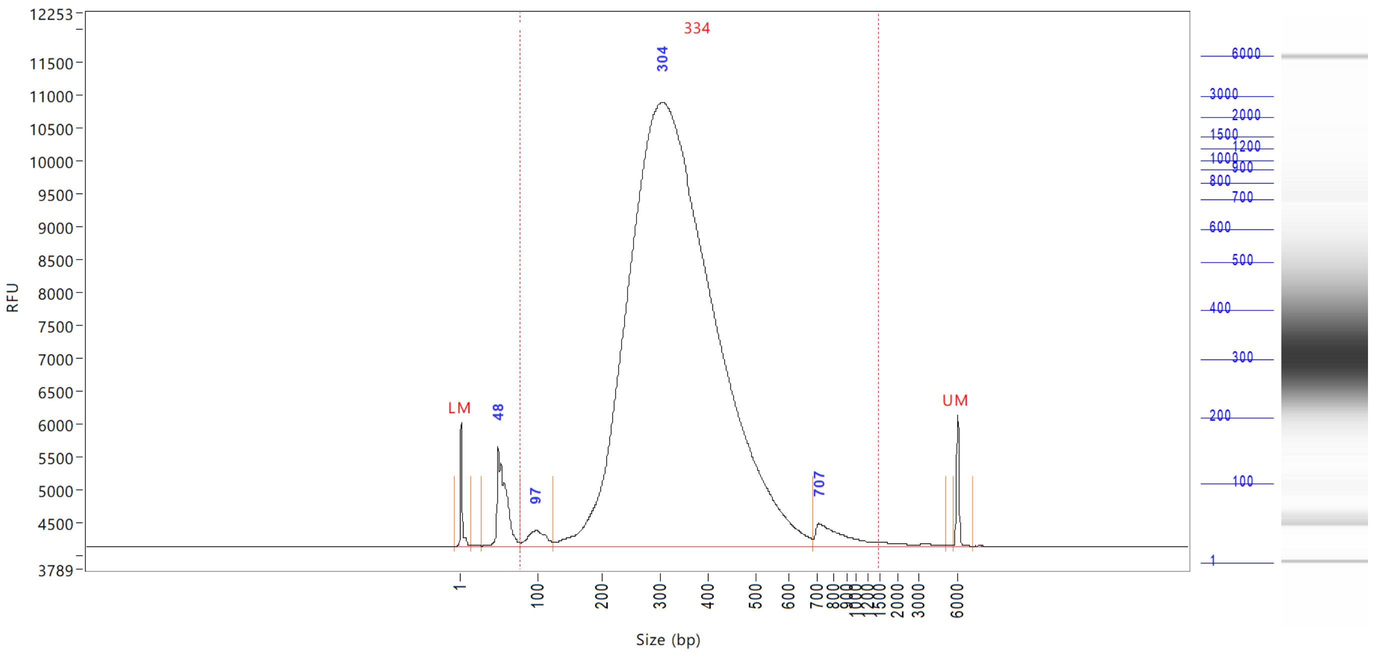

Peak	Size (bp)	Conc. (ng/uL)	From (bp)	To (bp)	RFU
1	1 (LM)	0.0124	0	15	1889
2	48	0.4883	28	77	1511
3	97	0.1482	77	122	242
4	304	15.3314	122	685	6765
5	707	0.2325	685	5085	349
6	6000 (UM)	0.0083	5670	7137	1999

TIC:	16.2004	ng/uL
TIM:	94.0626	nmole/L
Total Conc.:	16.2054	ng/uL

Smear Analysis 75 bp to 1500 bp 15.6762 ng/ul 96.7 %Total 77.1707 nmole/L 334 Avg. Size (b.p.) 31.64 %CV

Sample Peak Width (sec): 50 Sample Min Peak Height: 25 Sample Baseline V to V?: Y Sample Baseline V to V pts: 3
Sample Filter: Binomial # of Pts for Filter: 3 Sample Start Region (min): 0 Sample End Region (min): 50
Manual Baseline Start (min): 10 Manual Baseline End (min): 48
Marker Peak Width (sec): 5 Marker Min Peak Height: 200 Marker Baseline V to V?: Y Marker Baseline V to V pts: 3
Lower Marker Selection: First Peak > 200 RFU Upper Marker Selection: Last Peak > 200 RFU
Ladder Size (bp): 1, 100, 200, 300, 400, 500, 600, 700, 800, 900, 1000, 1200, 1500, 2000, 3000, 6000
Quantification Using: Ladder Final Concentration (ng/uL): 0.0830 Dilution Factor: 12.0

Sample: 103613-001-042
Well Location: B6
Created: Friday, June 21, 2019 2:11:36 PM

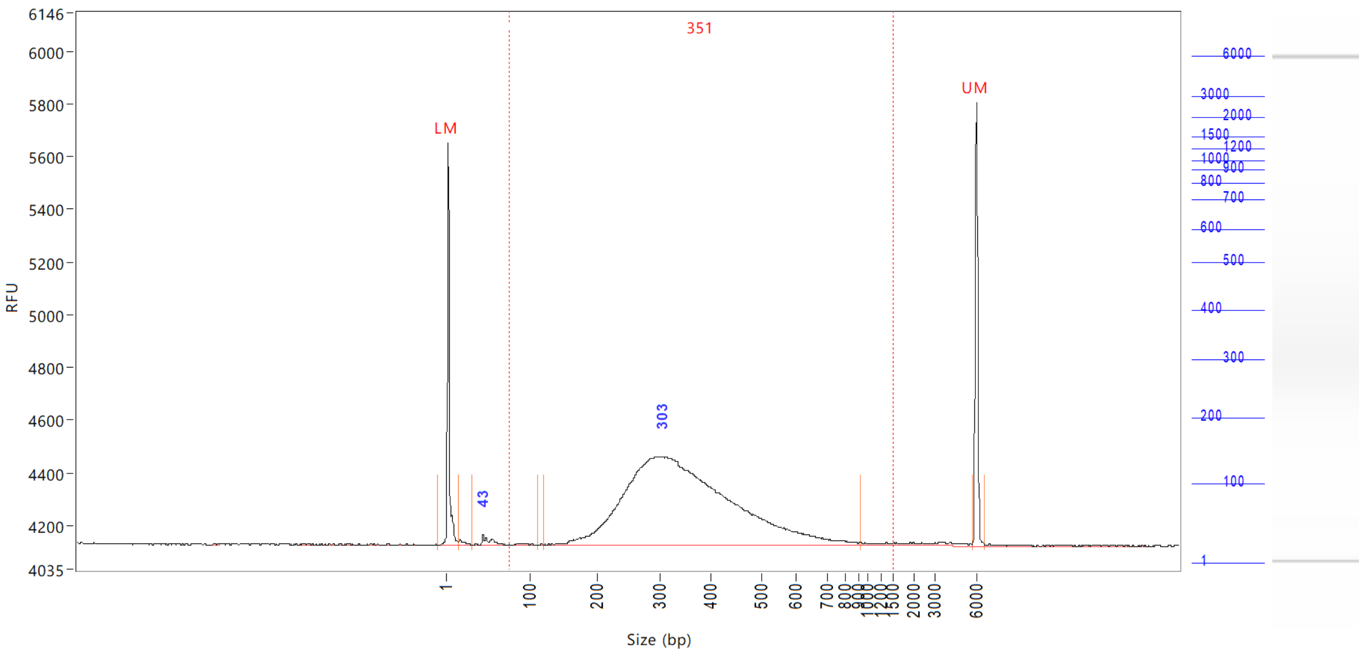

Peak	Size (bp)	Conc. (ng/uL)	From (bp)	To (bp)	RFU
1	1 (LM)	0.0124	0	14	1526
2	43	0.0184	31	112	42
3	303	1.0816	121	921	339
4	6000 (UM)	0.0075	5695	6606	1686

TIC:	1.1000	ng/uL
TIM:	5.6243	nmole/L
Total Conc.:	1.1249	ng/uL

Smear Analysis 75 bp to 1500 bp 1.0916 ng/ul 97.0 %Total 5.1162 nmole/L 351 Avg. Size (b.p.) 35.60 %CV

Sample Peak Width (sec): 50 Sample Min Peak Height: 25 Sample Baseline V to V?: Y Sample Baseline V to V pts: 3
Sample Filter: Binomial # of Pts for Filter: 3 Sample Start Region (min): 0 Sample End Region (min): 50
Manual Baseline Start (min): 10 Manual Baseline End (min): 48
Marker Peak Width (sec): 5 Marker Min Peak Height: 200 Marker Baseline V to V?: Y Marker Baseline V to V pts: 3
Lower Marker Selection: First Peak > 200 RFU Upper Marker Selection: Last Peak > 200 RFU
Ladder Size (bp): 1, 100, 200, 300, 400, 500, 600, 700, 800, 900, 1000, 1200, 1500, 2000, 3000, 6000
Quantification Using: Ladder Final Concentration (ng/uL): 0.0830 Dilution Factor: 12.0

 Copyright 2015 Advanced Analytical Technologies, Inc.

Sample: 103613-001-043
Well Location: C6
Created: Friday, June 21, 2019 2:11:36 PM

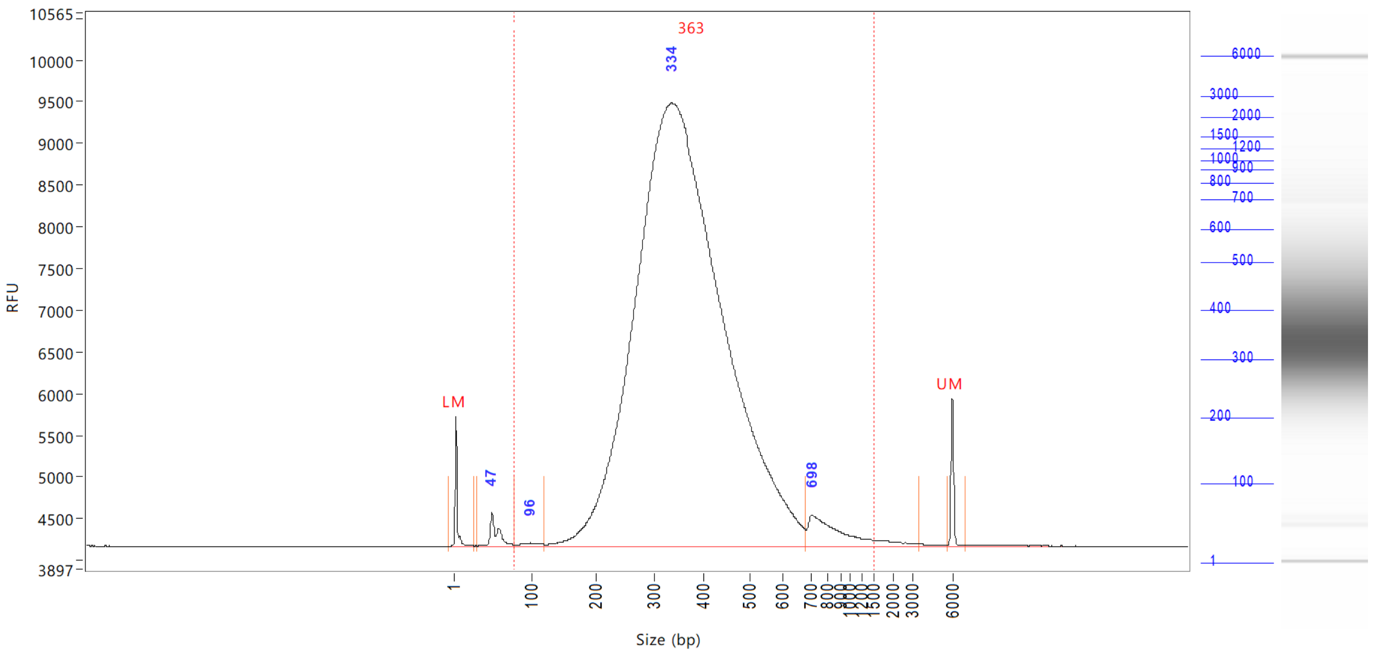

Peak	Size (bp)	Conc. (ng/uL)	From (bp)	To (bp)	RFU
1	1 (LM)	0.0124	0	26	1563
2	47	0.1288	29	77	403
3	96	0.0312	77	119	40
4	334	14.1946	119	680	5327
5	698	0.3152	680	3509	379
6	6000 (UM)	0.0084	5619	7061	1770

TIC:	14.6697	ng/uL
TIM:	71.0550	nmole/L
Total Conc.:	14.6778	ng/uL

Smear Analysis	75 bp to 1500 bp	14.4967 ng/ul	98.8 %Total	65.7633 nmole/L	363 Avg. Size (b.p.)	31.29 %CV

Sample Peak Width (sec): 50 Sample Min Peak Height: 25 Sample Baseline V to V?: Y Sample Baseline V to V pts: 3
Sample Filter: Binomial # of Pts for Filter: 3 Sample Start Region (min): 0 Sample End Region (min): 50
Manual Baseline Start (min): 10 Manual Baseline End (min): 48
Marker Peak Width (sec): 5 Marker Min Peak Height: 200 Marker Baseline V to V?: Y Marker Baseline V to V pts: 3
Lower Marker Selection: First Peak > 200 RFU Upper Marker Selection: Last Peak > 200 RFU
Ladder Size (bp): 1, 100, 200, 300, 400, 500, 600, 700, 800, 900, 1000, 1200, 1500, 2000, 3000, 6000
Quantification Using: Ladder Final Concentration (ng/uL): 0.0830 Dilution Factor: 12.0

Copyright 2015 Advanced Analytical Technologies, Inc.

Sample: 103613-001-044
Well Location: D6
Created: Friday, June 21, 2019 2:11:36 PM

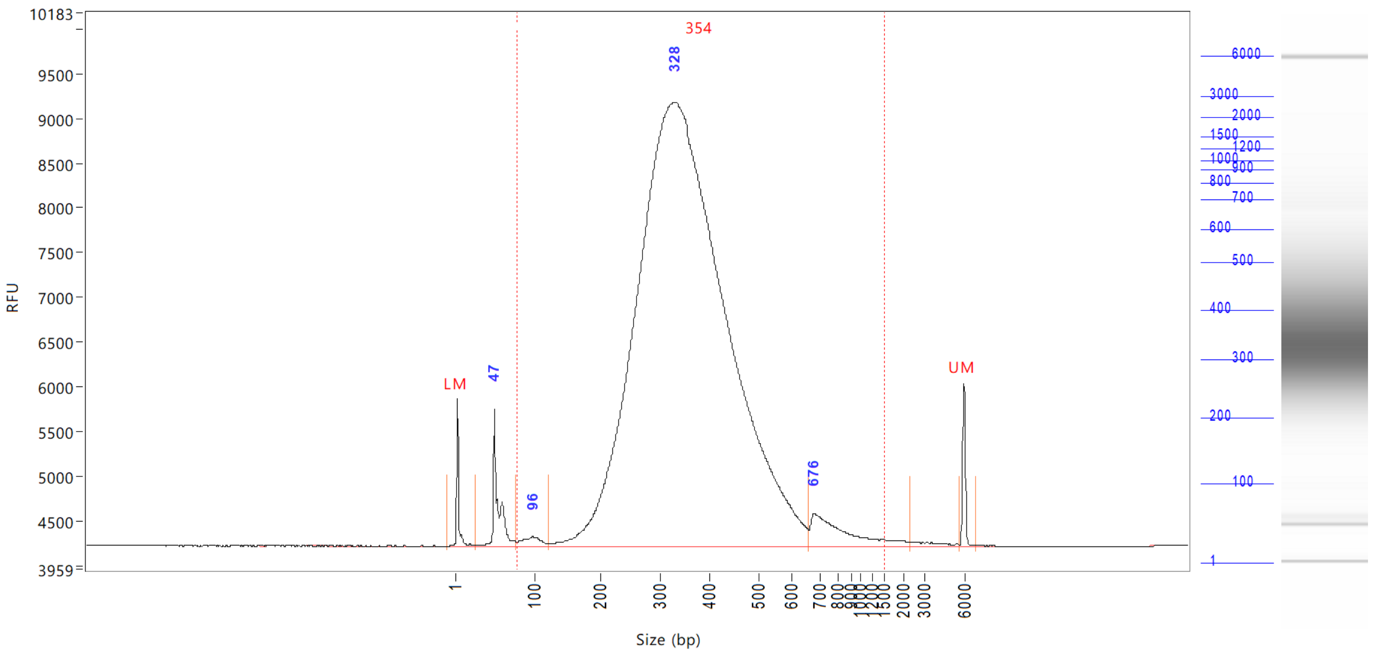

Peak	Size (bp)	Conc. (ng/uL)	From (bp)	To (bp)	RFU
1	1 (LM)	0.0124	0	25	1644
2	47	0.2958	25	75	1530
3	96	0.0724	75	120	105
4	328	12.6587	120	659	4972
5	676	0.2907	659	2301	370
6	6000 (UM)	0.0084	5670	6808	1825

TIC:	13.3176	ng/uL
TIM:	71.2917	nmole/L
Total Conc.:	13.3447	ng/uL

Smear Analysis 75 bp to 1500 bp 12.9915 ng/ul 97.4 %Total 60.3957 nmole/L 354 Avg. Size (b.p.) 31.77 %CV

Sample Peak Width (sec): 50 Sample Min Peak Height: 25 Sample Baseline V to V?: Y Sample Baseline V to V pts: 3
Sample Filter: Binomial # of Pts for Filter: 3 Sample Start Region (min): 0 Sample End Region (min): 50
Manual Baseline Start (min): 10 Manual Baseline End (min): 48
Marker Peak Width (sec): 5 Marker Min Peak Height: 200 Marker Baseline V to V?: Y Marker Baseline V to V pts: 3
Lower Marker Selection: First Peak > 200 RFU Upper Marker Selection: Last Peak > 200 RFU
Ladder Size (bp): 1, 100, 200, 300, 400, 500, 600, 700, 800, 900, 1000, 1200, 1500, 2000, 3000, 6000
Quantification Using: Ladder Final Concentration (ng/uL): 0.0830 Dilution Factor: 12.0

 Copyright 2015 Advanced Analytical Technologies, Inc.

Sample: 103613-001-045
Well Location: E6
Created: Friday, June 21, 2019 2:11:36 PM

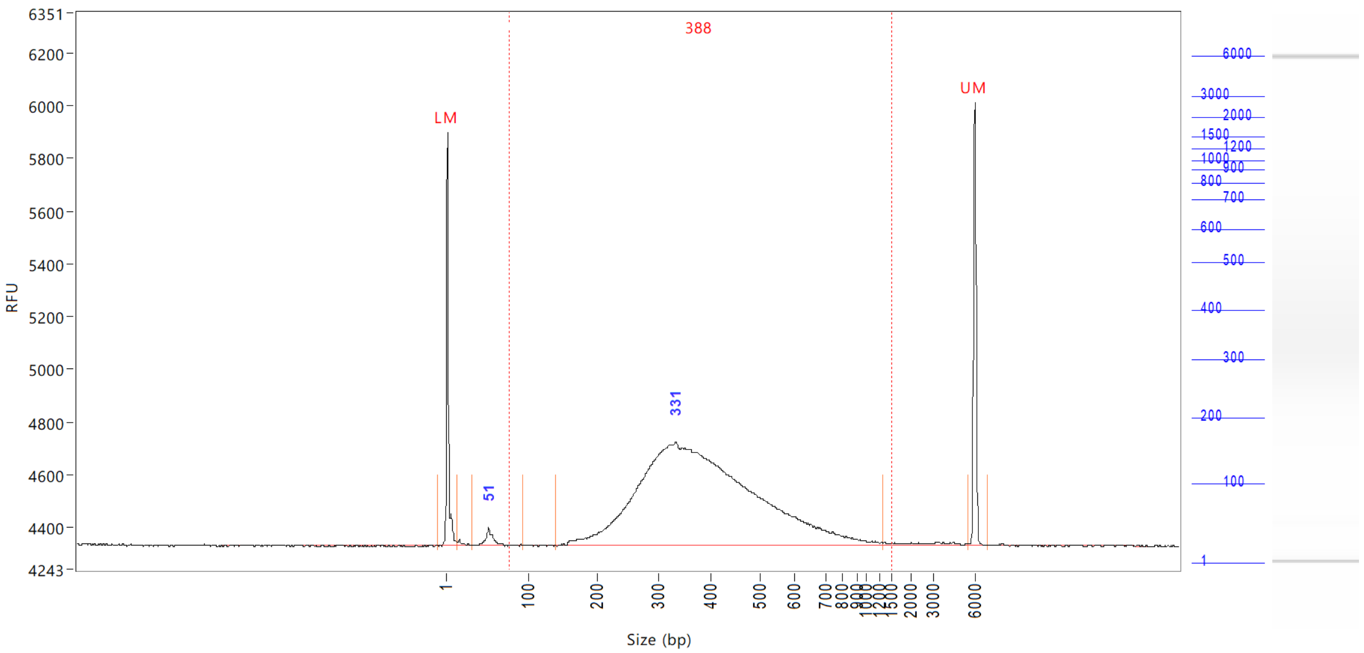

Peak	Size (bp)	Conc. (ng/uL)	From (bp)	To (bp)	RFU
1	1 (LM)	0.0124	0	14	1566
2	51	0.0225	32	92	69
3	331	1.3128	140	1273	392
4	6000 (UM)	0.0078	5543	6909	1683

TIC:	1.3353	ng/uL
TIM:	6.2640	nmole/L
Total Conc.:	1.3552	ng/uL

Smear Analysis 75 bp to 1500 bp 1.3172 ng/ul 97.2 %Total 5.5894 nmole/L 388 Avg. Size (b.p.) 34.82 %CV

Sample Peak Width (sec): 50 Sample Min Peak Height: 25 Sample Baseline V to V?: Y Sample Baseline V to V pts: 3
Sample Filter: Binomial # of Pts for Filter: 3 Sample Start Region (min): 0 Sample End Region (min): 50
Manual Baseline Start (min): 10 Manual Baseline End (min): 48
Marker Peak Width (sec): 5 Marker Min Peak Height: 200 Marker Baseline V to V?: Y Marker Baseline V to V pts: 3
Lower Marker Selection: First Peak > 200 RFU Upper Marker Selection: Last Peak > 200 RFU
Ladder Size (bp): 1, 100, 200, 300, 400, 500, 600, 700, 800, 900, 1000, 1200, 1500, 2000, 3000, 6000
Quantification Using: Ladder Final Concentration (ng/uL): 0.0830 Dilution Factor: 12.0

 Copyright 2015 Advanced Analytical Technologies, Inc.

Sample: 103613-001-046
Well Location: F6
Created: Friday, June 21, 2019 2:11:36 PM

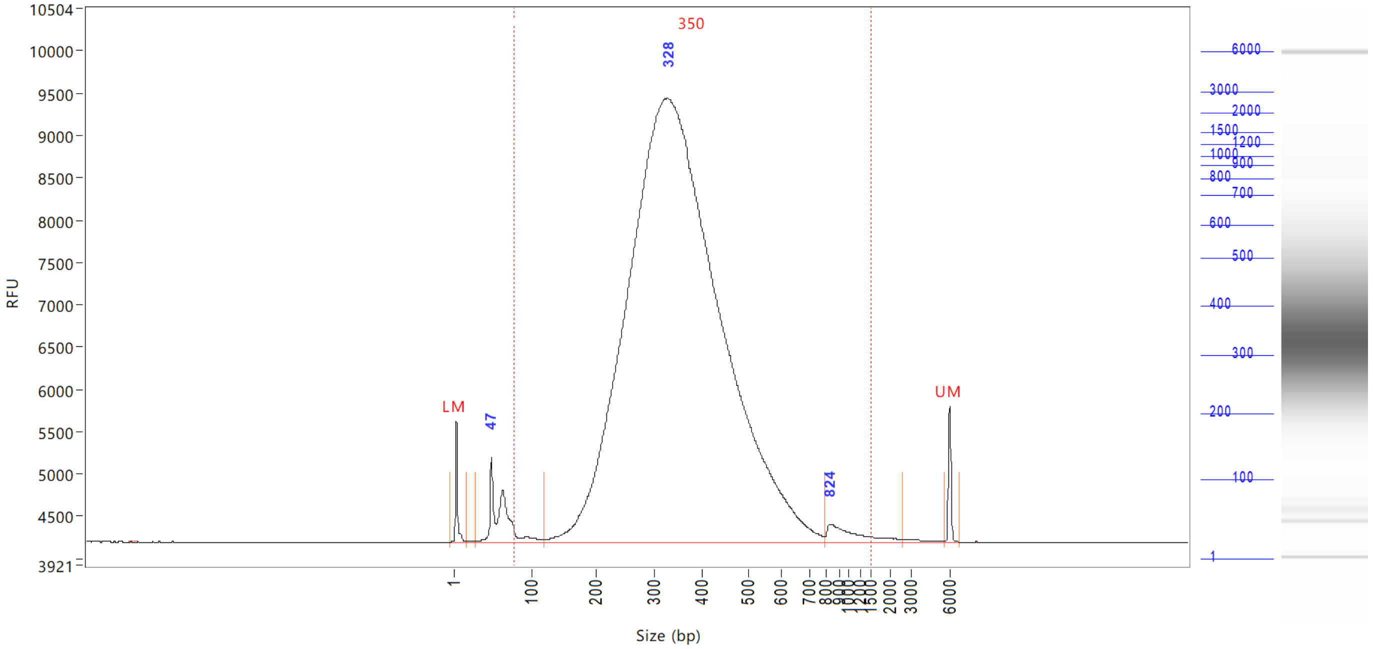

Peak	Size (bp)	Conc. (ng/uL)	From (bp)	To (bp)	RFU
1	1 (LM)	0.0124	0	15	1432
2	47	0.4876	28	118	1005
3	328	16.2209	118	792	5259
4	824	0.1566	792	2617	215
5	6000 (UM)	0.0081	5568	6733	1597

TIC:	16.8651	ng/uL
TIM:	90.4509	nmole/L
Total Conc.:	16.8858	ng/uL

Smear Analysis 75 bp to 1500 bp 16.4106 ng/ul 97.2 %Total 77.1775 nmole/L 350 Avg. Size (b.p.) 31.65 %CV

Sample Peak Width (sec): 50 Sample Min Peak Height: 25 Sample Baseline V to V?: Y Sample Baseline V to V pts: 3
Sample Filter: Binomial # of Pts for Filter: 3 Sample Start Region (min): 0 Sample End Region (min): 50
Manual Baseline Start (min): 10 Manual Baseline End (min): 48
Marker Peak Width (sec): 5 Marker Min Peak Height: 200 Marker Baseline V to V?: Y Marker Baseline V to V pts: 3
Lower Marker Selection: First Peak > 200 RFU Upper Marker Selection: Last Peak > 200 RFU
Ladder Size (bp): 1, 100, 200, 300, 400, 500, 600, 700, 800, 900, 1000, 1200, 1500, 2000, 3000, 6000
Quantification Using: Ladder Final Concentration (ng/uL): 0.0830 Dilution Factor: 12.0

Copyright 2015 Advanced Analytical Technologies, Inc.

Sample: 103613-001-047
Well Location: G6
Created: Friday, June 21, 2019 2:11:36 PM

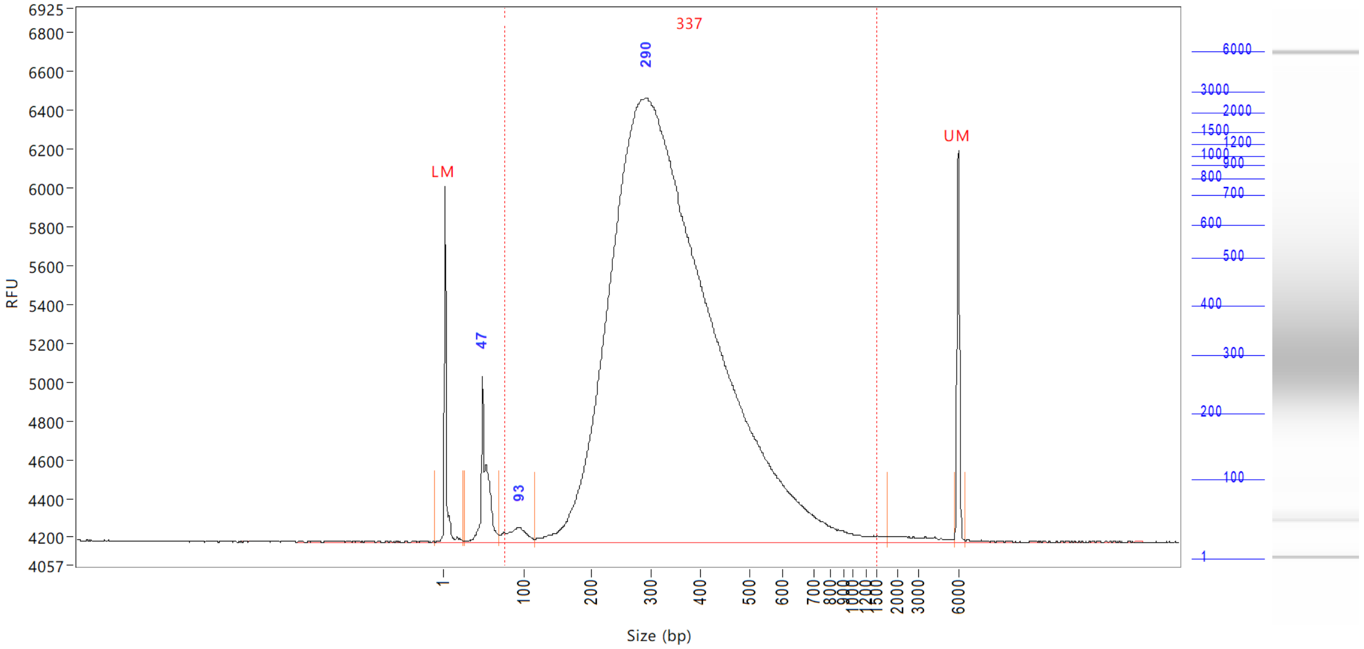

Peak	Size (bp)	Conc. (ng/uL)	From (bp)	To (bp)	RFU
1	1 (LM)	0.0124	0	24	1833
2	47	0.1691	27	69	851
3	93	0.0495	69	115	75
4	290	5.9514	115	1759	2291
5	6000 (UM)	0.0077	5695	6505	2016

TIC:	6.1700	ng/uL
TIM:	35.1417	nmole/L
Total Conc.:	6.1935	ng/uL

Smear Analysis	75 bp to 1500 bp	5.9891 ng/ul	96.7 %Total	29.2457 nmole/L	337 Avg. Size (b.p.)	34.60 %CV

```
Sample Peak Width (sec): 50   Sample Min Peak Height: 25    Sample Baseline V to V?: Y    Sample Baseline V to V pts: 3
Sample Filter: Binomial       # of Pts for Filter: 3        Sample Start Region (min): 0  Sample End Region (min): 50
Manual Baseline Start (min): 10     Manual Baseline End (min): 48
Marker Peak Width (sec): 5    Marker Min Peak Height: 200   Marker Baseline V to V?: Y    Marker Baseline V to V pts: 3
Lower Marker Selection: First Peak > 200 RFU                Upper Marker Selection: Last Peak > 200 RFU
Ladder Size (bp): 1, 100, 200, 300, 400, 500, 600, 700, 800, 900, 1000, 1200, 1500, 2000, 3000, 6000
Quantification Using: Ladder          Final Concentration (ng/uL): 0.0830          Dilution Factor: 12.0
```


Copyright 2015 Advanced Analytical Technologies, Inc.

Sample: 103613-001-048
Well Location: H6
Created: Friday, June 21, 2019 2:11:36 PM

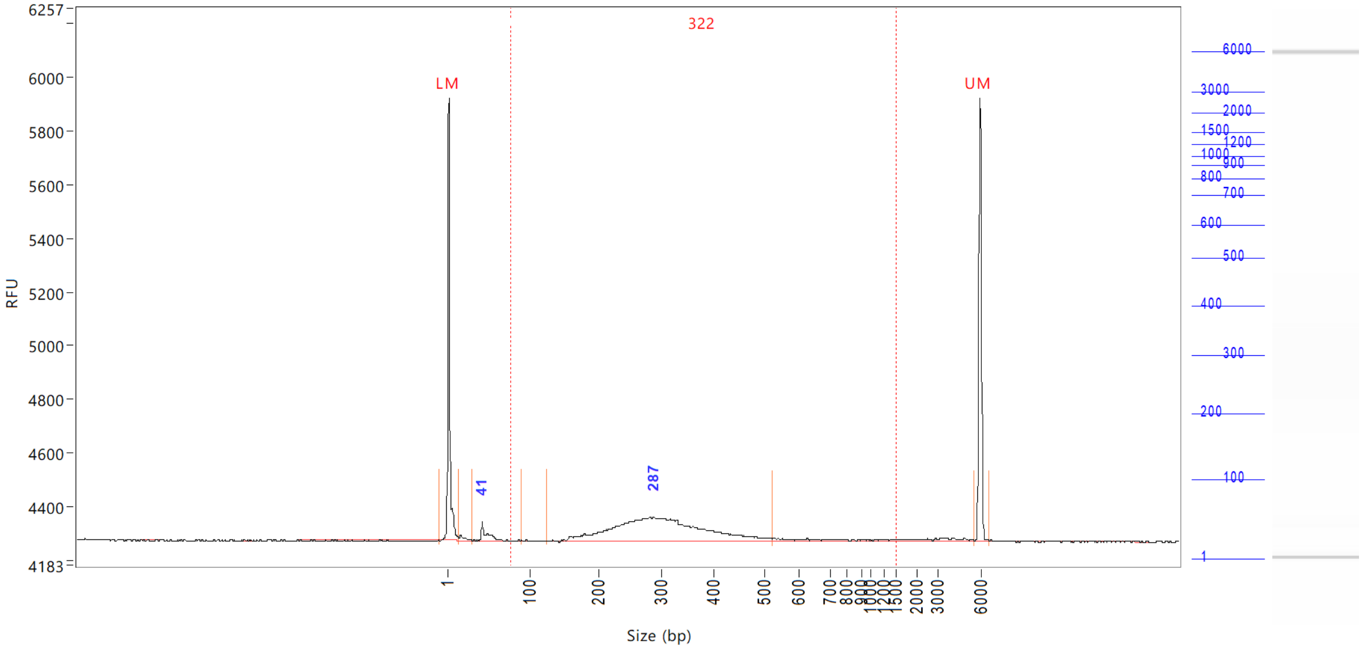

Peak	Size (bp)	Conc. (ng/uL)	From (bp)	To (bp)	RFU
1	1 (LM)	0.0124	0	14	1648
2	41	0.0140	30	89	67
3	287	0.2290	124	522	86
4	6000 (UM)	0.0072	5619	6682	1654
	TIC:	0.2430	ng/uL		
	TIM:	1.7515	nmole/L		
	Total Conc.:	0.2690	ng/uL		

Smear Analysis	75 bp to 1500 bp	0.2413 ng/ul	89.7 %Total	1.2328 nmole/L	322 Avg. Size (b.p.)	43.37 %CV

Sample Peak Width (sec): 50   Sample Min Peak Height: 25   Sample Baseline V to V?: Y   Sample Baseline V to V pts: 3
Sample Filter: Binomial   # of Pts for Filter: 3   Sample Start Region (min): 0   Sample End Region (min): 50
Manual Baseline Start (min): 10   Manual Baseline End (min): 48
Marker Peak Width (sec): 5   Marker Min Peak Height: 200   Marker Baseline V to V?: Y   Marker Baseline V to V pts: 3
Lower Marker Selection: First Peak > 200 RFU   Upper Marker Selection: Last Peak > 200 RFU
Ladder Size (bp): 1, 100, 200, 300, 400, 500, 600, 700, 800, 900, 1000, 1200, 1500, 2000, 3000, 6000
Quantification Using: Ladder   Final Concentration (ng/uL): 0.0830   Dilution Factor: 12.0

Copyright 2015 Advanced Analytical Technologies, Inc.

Sample: 103613-001-049
Well Location: A7
Created: Friday, June 21, 2019 2:11:36 PM

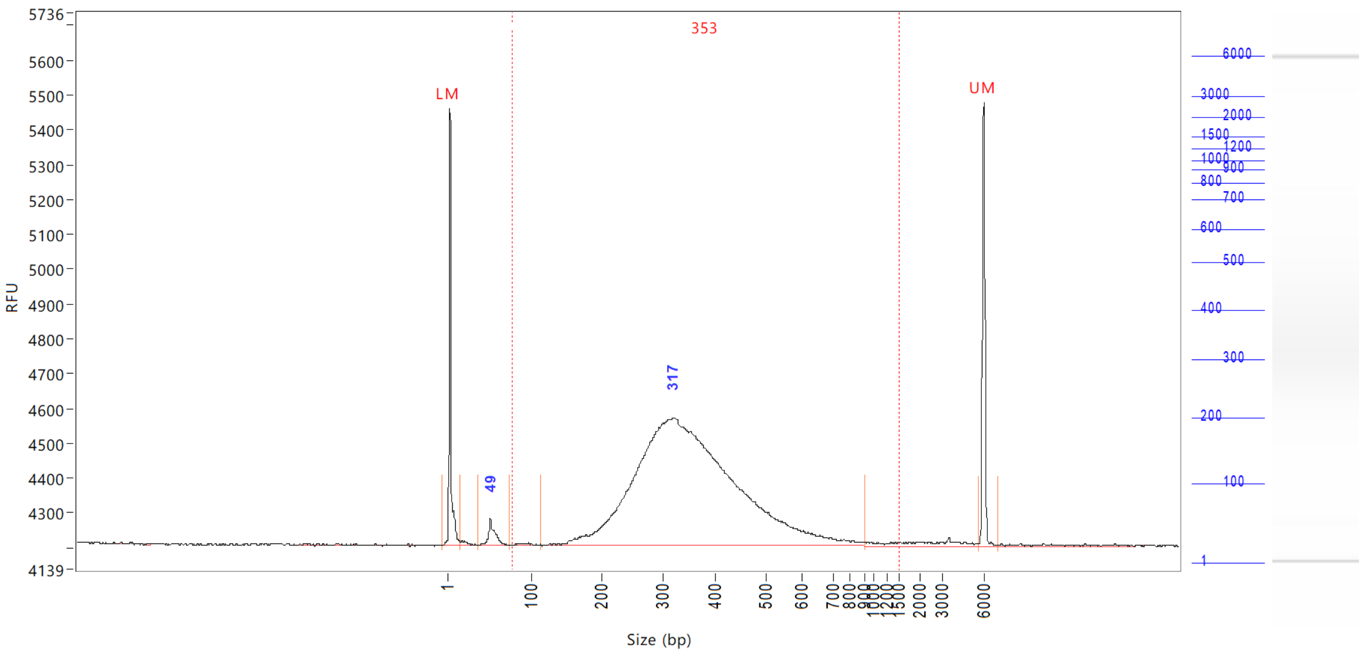

Peak	Size (bp)	Conc. (ng/uL)	From (bp)	To (bp)	RFU
1	1 (LM)	0.0124	0	15	1254
2	49	0.0258	36	72	76
3	317	1.3263	113	905	368
4	6000 (UM)	0.0070	5695	6985	1276
	TIC:	1.3521	ng/uL		
	TIM:	7.0476	nmole/L		
	Total Conc.:	1.3851	ng/uL		

Smear Analysis	75 bp to 1500 bp	1.3372 ng/ul	96.5 %Total	6.2363 nmole/L	353 Avg. Size (b.p.)	33.66 %CV

```
Sample Peak Width (sec): 50    Sample Min Peak Height: 25    Sample Baseline V to V?: Y    Sample Baseline V to V pts: 3
Sample Filter: Binomial        # of Pts for Filter: 3        Sample Start Region (min): 0  Sample End Region (min): 50
Manual Baseline Start (min): 10     Manual Baseline End (min): 48
Marker Peak Width (sec): 5     Marker Min Peak Height: 200   Marker Baseline V to V?: Y    Marker Baseline V to V pts: 3
Lower Marker Selection: First Peak > 200 RFU                 Upper Marker Selection: Last Peak > 200 RFU
Ladder Size (bp): 1, 100, 200, 300, 400, 500, 600, 700, 800, 900, 1000, 1200, 1500, 2000, 3000, 6000
Quantification Using: Ladder          Final Concentration (ng/uL): 0.0830          Dilution Factor: 12.0
```


Copyright 2015 Advanced Analytical Technologies, Inc.

Sample: 103613-001-050
Well Location: B7
Created: Friday, June 21, 2019 2:11:36 PM

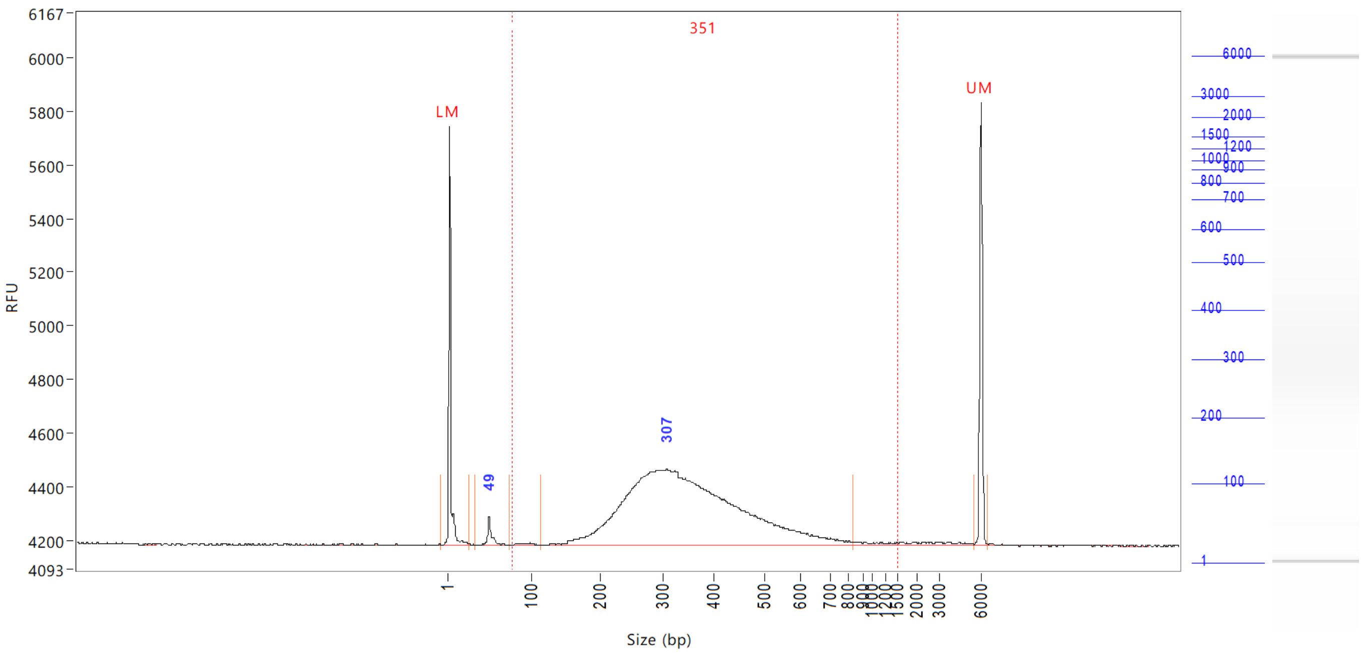

Peak	Size (bp)	Conc. (ng/uL)	From (bp)	To (bp)	RFU
1	1 (LM)	0.0124	0	25	1562
2	49	0.0179	32	73	105
3	307	0.9357	112	837	283
4	6000 (UM)	0.0074	5492	6505	1655

TIC:	0.9536	ng/uL
TIM:	5.0351	nmole/L
Total Conc.:	0.9810	ng/uL

Smear Analysis	75 bp to 1500 bp	0.9485 ng/ul	96.7 %Total	4.4411 nmole/L	351 Avg. Size (b.p.)	37.37 %CV

Sample Peak Width (sec): 50 Sample Min Peak Height: 25 Sample Baseline V to V?: Y Sample Baseline V to V pts: 3
Sample Filter: Binomial # of Pts for Filter: 3 Sample Start Region (min): 0 Sample End Region (min): 50
Manual Baseline Start (min): 10 Manual Baseline End (min): 48
Marker Peak Width (sec): 5 Marker Min Peak Height: 200 Marker Baseline V to V?: Y Marker Baseline V to V pts: 3
Lower Marker Selection: First Peak > 200 RFU Upper Marker Selection: Last Peak > 200 RFU
Ladder Size (bp): 1, 100, 200, 300, 400, 500, 600, 700, 800, 900, 1000, 1200, 1500, 2000, 3000, 6000
Quantification Using: Ladder Final Concentration (ng/uL): 0.0830 Dilution Factor: 12.0

 Copyright 2015 Advanced Analytical Technologies, Inc.

Sample: 103613-001-051
Well Location: C7
Created: Friday, June 21, 2019 2:11:36 PM

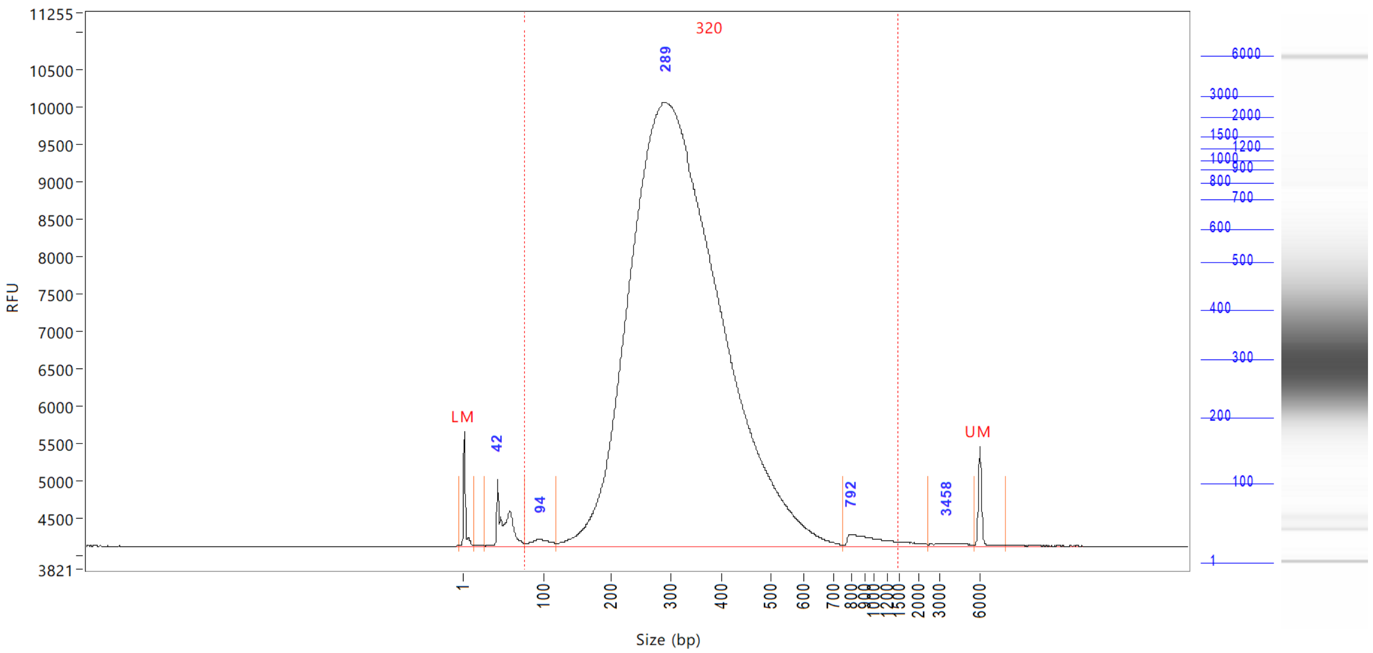

Peak	Size (bp)	Conc. (ng/uL)	From (bp)	To (bp)	RFU
1	1 (LM)	0.0124	0	14	1532
2	42	0.3136	27	75	902
3	94	0.0704	75	116	93
4	289	17.1690	116	755	5939
5	792	0.1366	755	2451	163
6	3458	0.0252	2451	5568	41
7	6000 (UM)	0.0083	5568	7895	1334

TIC:	17.7148	ng/uL
TIM:	100.8776	nmole/L
Total Conc.:	17.7181	ng/uL

Smear Analysis 75 bp to 1500 bp 17.3519 ng/ul 97.9 %Total 89.2609 nmole/L 320 Avg. Size (b.p.) 31.30 %CV

Sample Peak Width (sec): 50 Sample Min Peak Height: 25 Sample Baseline V to V?: Y Sample Baseline V to V pts: 3
Sample Filter: Binomial # of Pts for Filter: 3 Sample Start Region (min): 0 Sample End Region (min): 50
Manual Baseline Start (min): 10 Manual Baseline End (min): 48
Marker Peak Width (sec): 5 Marker Min Peak Height: 200 Marker Baseline V to V?: Y Marker Baseline V to V pts: 3
Lower Marker Selection: First Peak > 200 RFU Upper Marker Selection: Last Peak > 200 RFU
Ladder Size (bp): 1, 100, 200, 300, 400, 500, 600, 700, 800, 900, 1000, 1200, 1500, 2000, 3000, 6000
Quantification Using: Ladder Final Concentration (ng/uL): 0.0830 Dilution Factor: 12.0

Copyright 2015 Advanced Analytical Technologies, Inc.

Sample: 103613-001-052
Well Location: D7
Created: Friday, June 21, 2019 2:11:36 PM

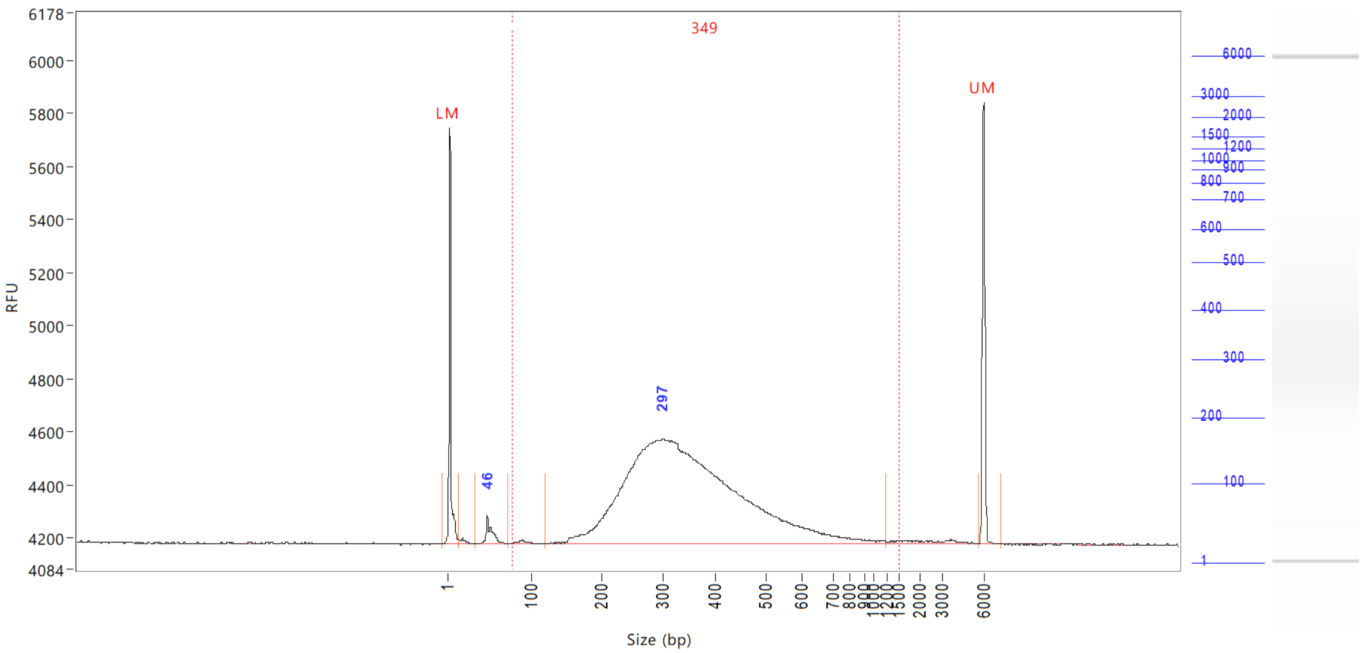

Peak	Size (bp)	Conc. (ng/uL)	From (bp)	To (bp)	RFU
1	1 (LM)	0.0124	0	14	1562
2	46	0.0250	31	71	106
3	297	1.3200	119	1172	395
4	6000 (UM)	0.0076	5644	7212	1666

TIC:	1.3450	ng/uL
TIM:	7.0765	nmole/L
Total Conc.:	1.3713	ng/uL

Smear Analysis 75 bp to 1500 bp 1.3280 ng/ul 96.8 %Total 6.2655 nmole/L 349 Avg. Size (b.p.) 36.75 %CV

Sample Peak Width (sec): 50 Sample Min Peak Height: 25 Sample Baseline V to V?: Y Sample Baseline V to V pts: 3
Sample Filter: Binomial # of Pts for Filter: 3 Sample Start Region (min): 0 Sample End Region (min): 50
Manual Baseline Start (min): 10 Manual Baseline End (min): 48
Marker Peak Width (sec): 5 Marker Min Peak Height: 200 Marker Baseline V to V?: Y Marker Baseline V to V pts: 3
Lower Marker Selection: First Peak > 200 RFU Upper Marker Selection: Last Peak > 200 RFU
Ladder Size (bp): 1, 100, 200, 300, 400, 500, 600, 700, 800, 900, 1000, 1200, 1500, 2000, 3000, 6000
Quantification Using: Ladder Final Concentration (ng/uL): 0.0830 Dilution Factor: 12.0

 Copyright 2015 Advanced Analytical Technologies, Inc.

Sample: 103613-001-053
Well Location: E7
Created: Friday, June 21, 2019 2:11:36 PM

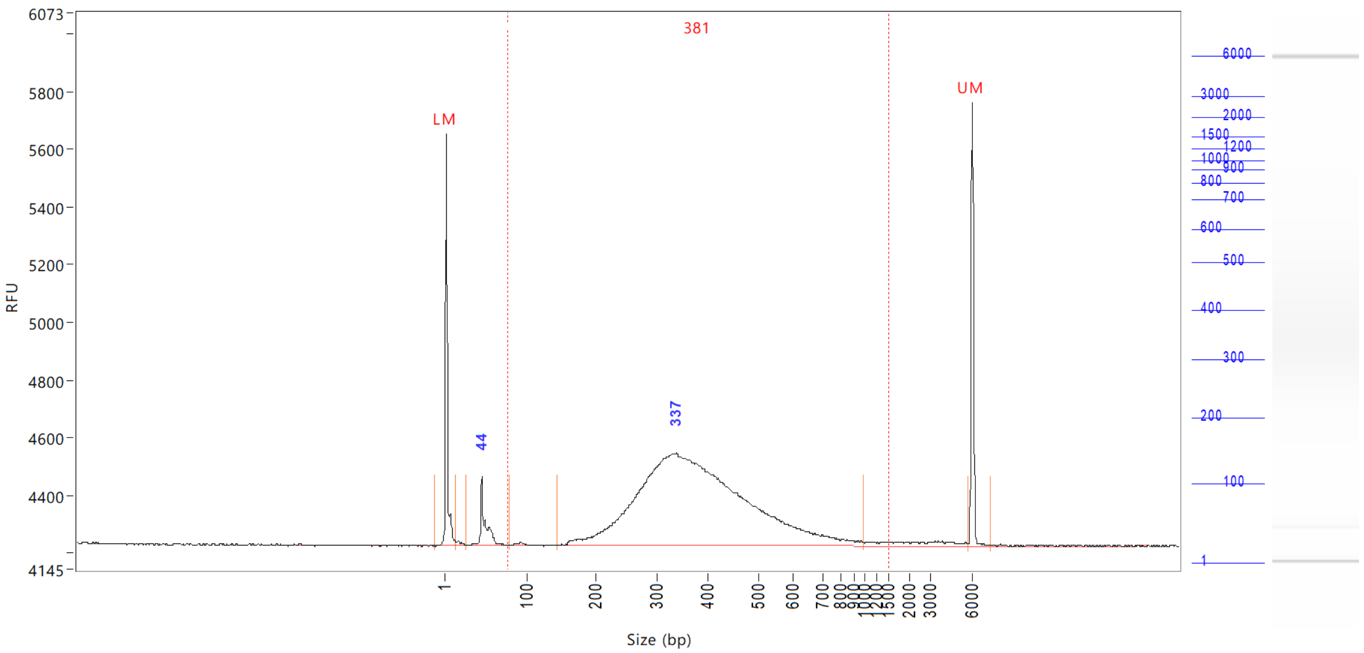

Peak	Size (bp)	Conc. (ng/uL)	From (bp)	To (bp)	RFU
1	1 (LM)	0.0124	0	14	1426
2	44	0.0505	27	77	237
3	337	1.0923	143	1001	321
4	6000 (UM)	0.0077	5720	7288	1540

TIC:	1.1428	ng/uL
TIM:	6.4903	nmole/L
Total Conc.:	1.1760	ng/uL

Smear Analysis 75 bp to 1500 bp 1.1032 ng/ul 93.8 %Total 4.7600 nmole/L 381 Avg. Size (b.p.) 35.36 %CV

Sample Peak Width (sec): 50 Sample Min Peak Height: 25 Sample Baseline V to V?: Y Sample Baseline V to V pts: 3
Sample Filter: Binomial # of Pts for Filter: 3 Sample Start Region (min): 0 Sample End Region (min): 50
Manual Baseline Start (min): 10 Manual Baseline End (min): 48
Marker Peak Width (sec): 5 Marker Min Peak Height: 200 Marker Baseline V to V?: Y Marker Baseline V to V pts: 3
Lower Marker Selection: First Peak > 200 RFU Upper Marker Selection: Last Peak > 200 RFU
Ladder Size (bp): 1, 100, 200, 300, 400, 500, 600, 700, 800, 900, 1000, 1200, 1500, 2000, 3000, 6000
Quantification Using: Ladder Final Concentration (ng/uL): 0.0830 Dilution Factor: 12.0

 Copyright 2015 Advanced Analytical Technologies, Inc.

Sample: 103613-001-054
Well Location: F7
Created: Friday, June 21, 2019 2:11:36 PM

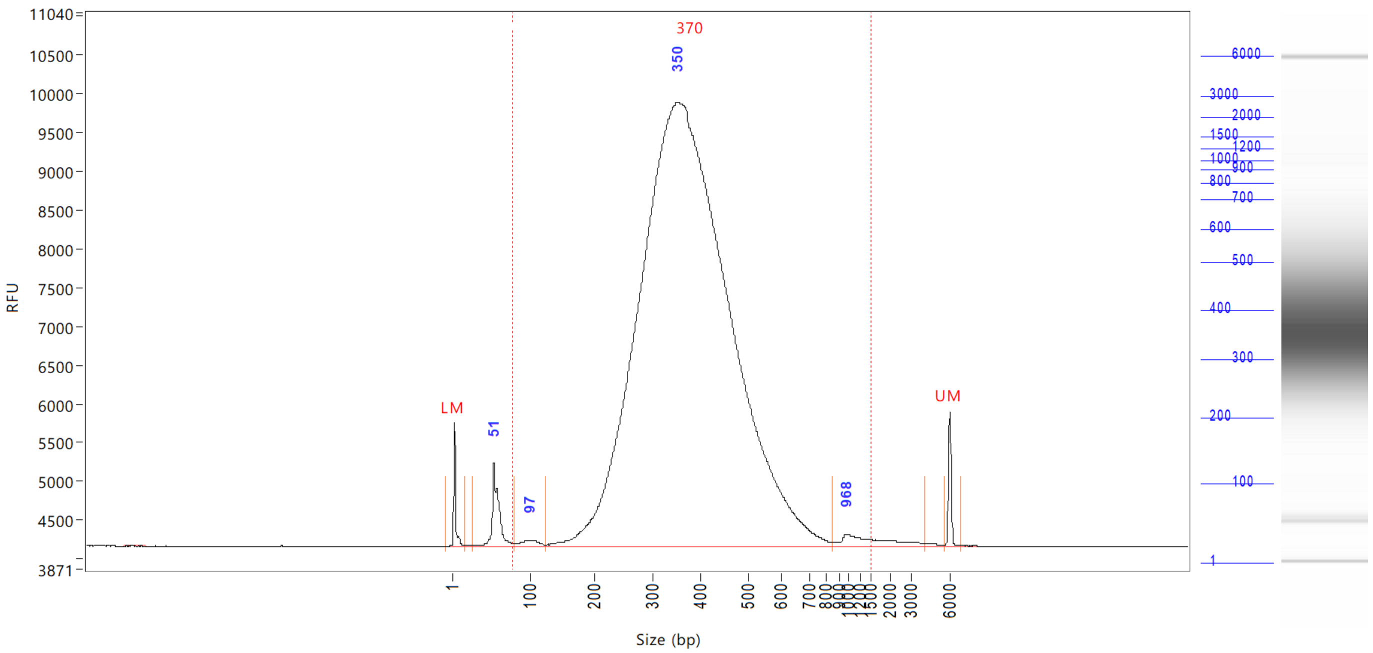

Peak	Size (bp)	Conc. (ng/uL)	From (bp)	To (bp)	RFU
1	1 (LM)	0.0124	0	15	1588
2	51	0.2936	26	79	1068
3	97	0.0566	79	124	81
4	350	15.1445	124	847	5730
5	968	0.1410	847	4094	158
6	6000 (UM)	0.0082	5593	6834	1726

TIC:	15.6356	ng/uL
TIM:	77.7241	nmole/L
Total Conc.:	15.6502	ng/uL

Smear Analysis 75 bp to 1500 bp 15.2817 ng/ul 97.6 %Total 67.9497 nmole/L 370 Avg. Size (b.p.) 29.14 %CV

```
Sample Peak Width (sec): 50   Sample Min Peak Height: 25    Sample Baseline V to V?: Y    Sample Baseline V to V pts: 3
Sample Filter: Binomial       # of Pts for Filter: 3        Sample Start Region (min): 0  Sample End Region (min): 50
Manual Baseline Start (min): 10     Manual Baseline End (min): 48
Marker Peak Width (sec): 5    Marker Min Peak Height: 200   Marker Baseline V to V?: Y    Marker Baseline V to V pts: 3
Lower Marker Selection: First Peak > 200 RFU                Upper Marker Selection: Last Peak > 200 RFU
Ladder Size (bp): 1, 100, 200, 300, 400, 500, 600, 700, 800, 900, 1000, 1200, 1500, 2000, 3000, 6000
Quantification Using: Ladder          Final Concentration (ng/uL): 0.0830          Dilution Factor: 12.0
```

 Copyright 2015 Advanced Analytical Technologies, Inc.

Sample: 103613-001-055
Well Location: G7
Created: Friday, June 21, 2019 2:11:36 PM

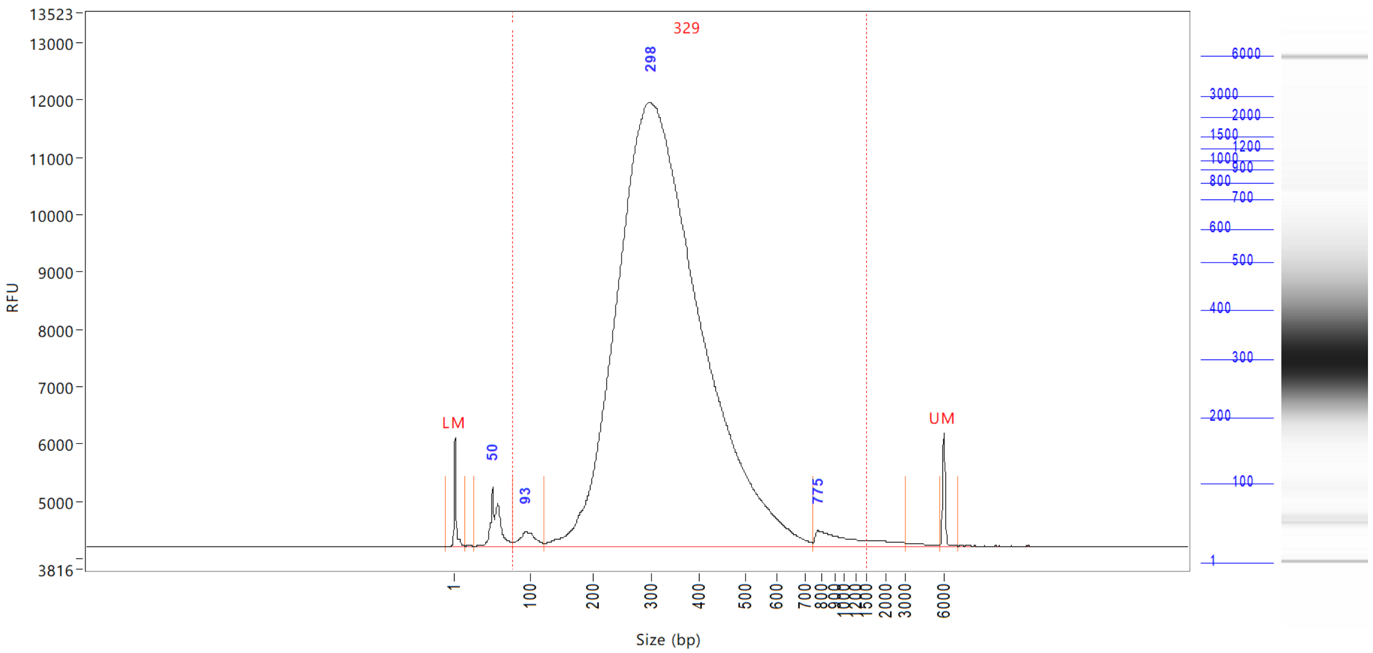

Peak	Size (bp)	Conc. (ng/uL)	From (bp)	To (bp)	RFU
1	1 (LM)	0.0124	0	15	1893
2	50	0.2977	28	77	1028
3	93	0.1387	77	122	268
4	298	17.0557	122	742	7759
5	775	0.2127	742	3000	277
6	6000 (UM)	0.0078	5670	7086	1970

TIC:	17.7049	ng/uL
TIM:	97.5888	nmole/L
Total Conc.:	17.7294	ng/uL

Smear Analysis 75 bp to 1500 bp 17.3543 ng/ul 97.9 %Total 86.6620 nmole/L 329 Avg. Size (b.p.) 32.46 %CV

Sample Peak Width (sec): 50 Sample Min Peak Height: 25 Sample Baseline V to V?: Y Sample Baseline V to V pts: 3
Sample Filter: Binomial # of Pts for Filter: 3 Sample Start Region (min): 0 Sample End Region (min): 50
Manual Baseline Start (min): 10 Manual Baseline End (min): 48
Marker Peak Width (sec): 5 Marker Min Peak Height: 200 Marker Baseline V to V?: Y Marker Baseline V to V pts: 3
Lower Marker Selection: First Peak > 200 RFU Upper Marker Selection: Last Peak > 200 RFU
Ladder Size (bp): 1, 100, 200, 300, 400, 500, 600, 700, 800, 900, 1000, 1200, 1500, 2000, 3000, 6000
Quantification Using: Ladder Final Concentration (ng/uL): 0.0830 Dilution Factor: 12.0

Copyright 2015 Advanced Analytical Technologies, Inc.

Sample: 103613-001-056
Well Location: H7
Created: Friday, June 21, 2019 2:11:36 PM

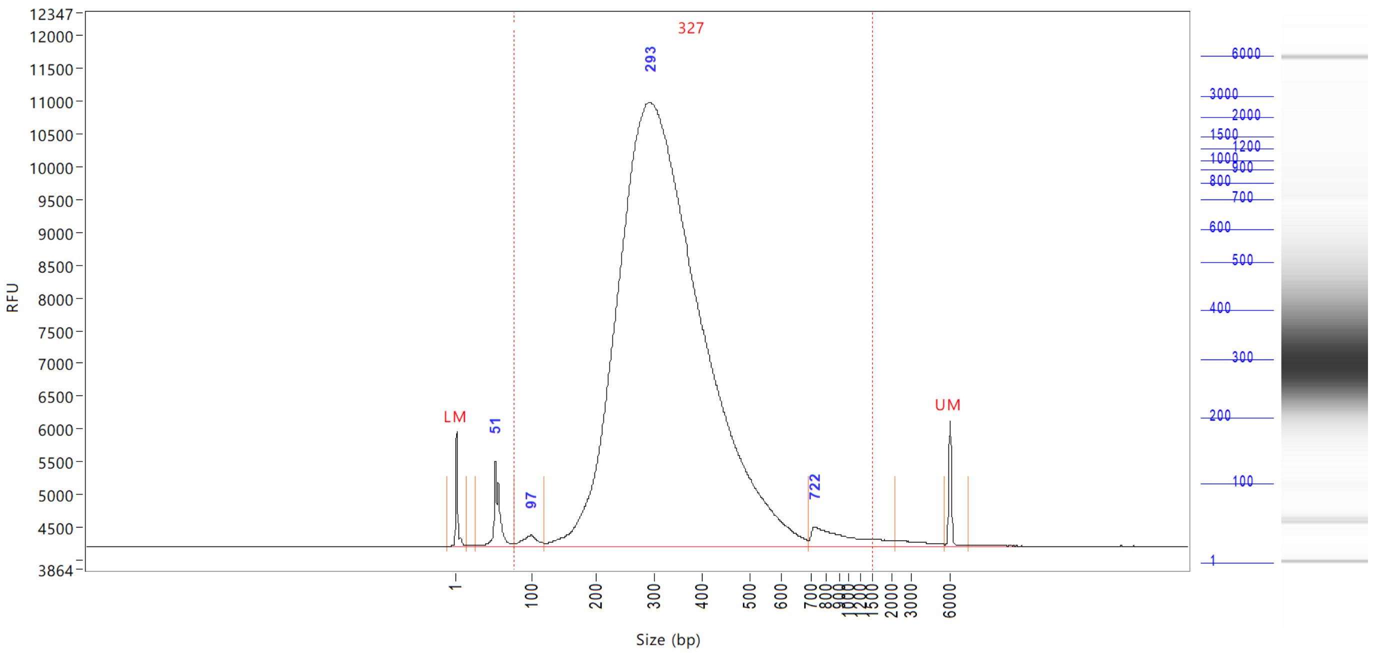

Peak	Size (bp)	Conc. (ng/uL)	From (bp)	To (bp)	RFU
1	1 (LM)	0.0124	0	15	1755
2	51	0.2689	27	76	1302
3	97	0.0929	76	118	170
4	293	15.7427	118	694	6781
5	722	0.2410	694	2201	303
6	6000 (UM)	0.0084	5593	7364	1913

TIC:	16.3455	ng/uL
TIM:	90.8209	nmole/L
Total Conc.:	16.4000	ng/uL

Smear Analysis	75 bp to 1500 bp	16.0383 ng/ul	97.8 %Total	80.7347 nmole/L	327 Avg. Size (b.p.)	33.32 %CV

```
Sample Peak Width (sec): 50   Sample Min Peak Height: 25    Sample Baseline V to V?: Y    Sample Baseline V to V pts: 3
Sample Filter: Binomial       # of Pts for Filter: 3        Sample Start Region (min): 0  Sample End Region (min): 50
Manual Baseline Start (min): 10     Manual Baseline End (min): 48
Marker Peak Width (sec): 5    Marker Min Peak Height: 200   Marker Baseline V to V?: Y    Marker Baseline V to V pts: 3
Lower Marker Selection: First Peak > 200 RFU                Upper Marker Selection: Last Peak > 200 RFU
Ladder Size (bp): 1, 100, 200, 300, 400, 500, 600, 700, 800, 900, 1000, 1200, 1500, 2000, 3000, 6000
Quantification Using: Ladder          Final Concentration (ng/uL): 0.0830         Dilution Factor: 12.0
```


Copyright 2015 Advanced Analytical Technologies, Inc.

Sample: 103613-001-057
Well Location: A8
Created: Friday, June 21, 2019 2:11:36 PM

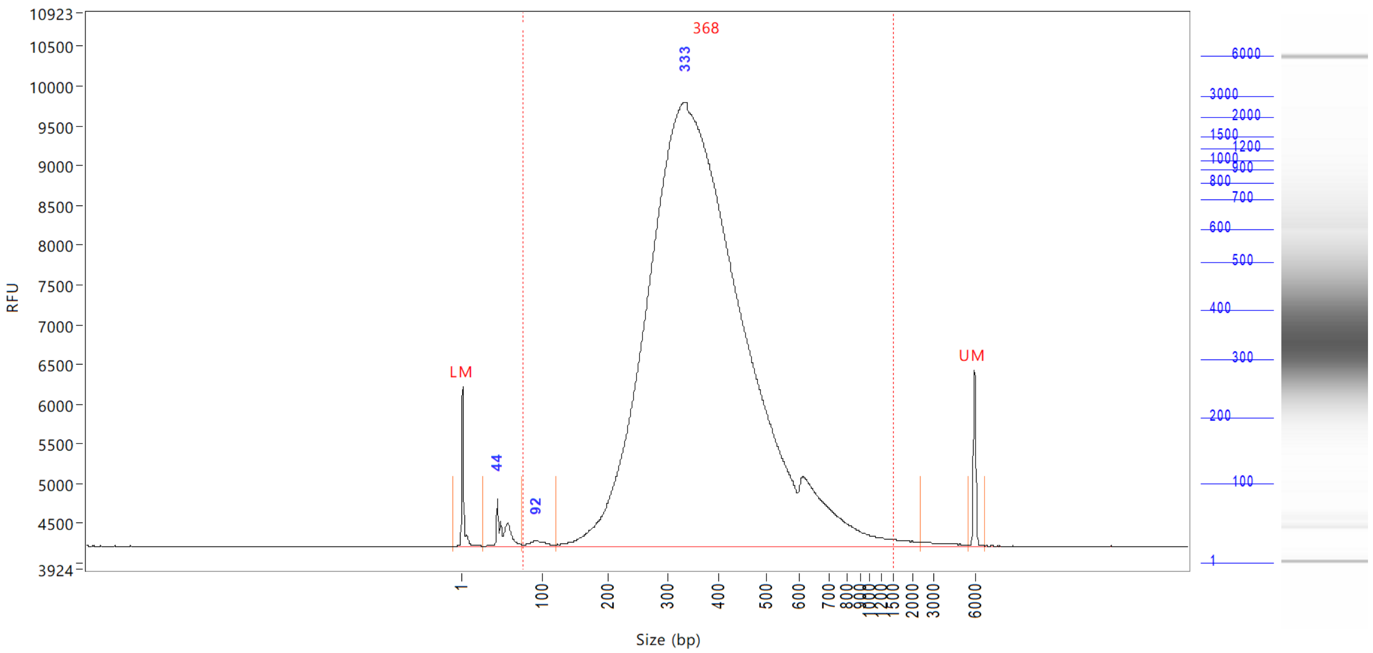

Peak	Size (bp)	Conc. (ng/uL)	From (bp)	To (bp)	RFU
1	1 (LM)	0.0124	0	28	2005
2	44	0.1360	28	75	601
3	92	0.0367	75	120	73
4	333	12.6332	120	2367	5592
5	6000 (UM)	0.0078	5543	6758	2225

TIC:	12.8059	ng/uL
TIM:	60.7902	nmole/L
Total Conc.:	12.8284	ng/uL

Smear Analysis	75 bp to 1500 bp	12.6429 ng/ul	98.6 %Total	56.4800 nmole/L	368 Avg. Size (b.p.)	32.21 %CV

Sample Peak Width (sec): 50 Sample Min Peak Height: 25 Sample Baseline V to V?: Y Sample Baseline V to V pts: 3
Sample Filter: Binomial # of Pts for Filter: 3 Sample Start Region (min): 0 Sample End Region (min): 50
Manual Baseline Start (min): 10 Manual Baseline End (min): 48
Marker Peak Width (sec): 5 Marker Min Peak Height: 200 Marker Baseline V to V?: Y Marker Baseline V to V pts: 3
Lower Marker Selection: First Peak > 200 RFU Upper Marker Selection: Last Peak > 200 RFU
Ladder Size (bp): 1, 100, 200, 300, 400, 500, 600, 700, 800, 900, 1000, 1200, 1500, 2000, 3000, 6000
Quantification Using: Ladder Final Concentration (ng/uL): 0.0830 Dilution Factor: 12.0

 Copyright 2015 Advanced Analytical Technologies, Inc.

Sample: 103613-001-058
Well Location: B8
Created: Friday, June 21, 2019 2:11:36 PM

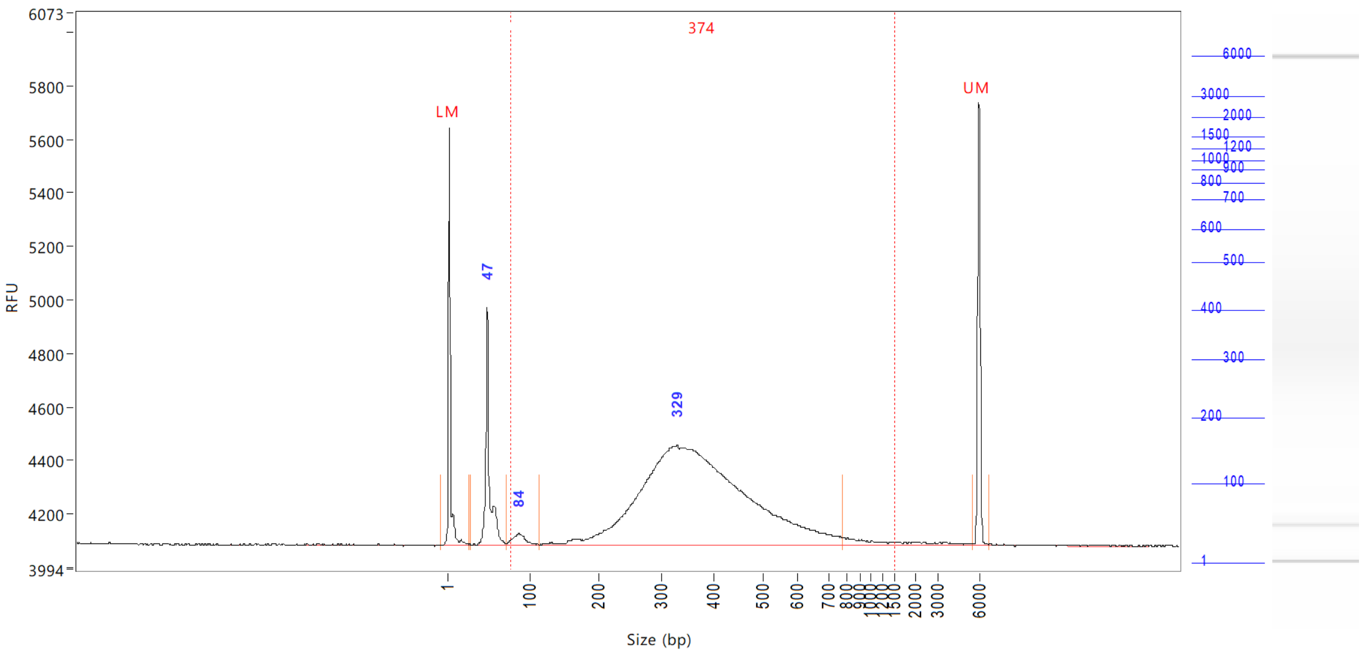

Peak	Size (bp)	Conc. (ng/uL)	From (bp)	To (bp)	RFU
1	1 (LM)	0.0124	0	26	1560
2	47	0.1412	27	71	886
3	84	0.0223	71	111	43
4	329	1.1406	111	777	377
5	6000 (UM)	0.0075	5517	6682	1659

TIC:	1.3041	ng/uL
TIM:	10.2346	nmole/L
Total Conc.:	1.3363	ng/uL

Smear Analysis	75 bp to 1500 bp	1.1794 ng/ul	88.3 %Total	5.1908 nmole/L	374 Avg. Size (b.p.)	36.75 %CV

Sample Peak Width (sec): 50 Sample Min Peak Height: 25 Sample Baseline V to V?: Y Sample Baseline V to V pts: 3
Sample Filter: Binomial # of Pts for Filter: 3 Sample Start Region (min): 0 Sample End Region (min): 50
Manual Baseline Start (min): 10 Manual Baseline End (min): 48
Marker Peak Width (sec): 5 Marker Min Peak Height: 200 Marker Baseline V to V?: Y Marker Baseline V to V pts: 3
Lower Marker Selection: First Peak > 200 RFU Upper Marker Selection: Last Peak > 200 RFU
Ladder Size (bp): 1, 100, 200, 300, 400, 500, 600, 700, 800, 900, 1000, 1200, 1500, 2000, 3000, 6000
Quantification Using: Ladder Final Concentration (ng/uL): 0.0830 Dilution Factor: 12.0

 Copyright 2015 Advanced Analytical Technologies, Inc.

Sample: 103613-001-059
Well Location: C8
Created: Friday, June 21, 2019 2:11:36 PM

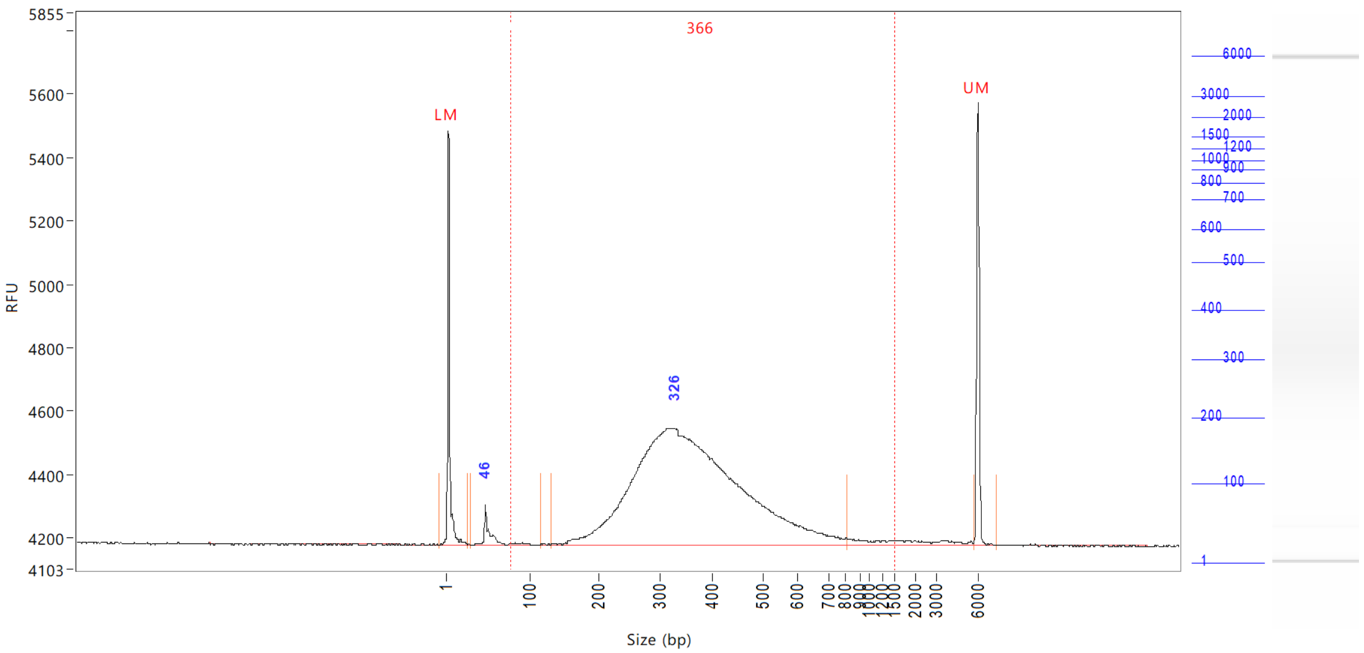

Peak	Size (bp)	Conc. (ng/uL)	From (bp)	To (bp)	RFU
1	1 (LM)	0.0124	0	26	1302
2	46	0.0294	28	115	124
3	326	1.3231	130	806	368
4	6000 (UM)	0.0074	5670	7263	1396
	TIC:	1.3524	ng/uL		
	TIM:	6.9710	nmole/L		
	Total Conc.:	1.3851	ng/uL		

Smear Analysis 75 bp to 1500 bp 1.3404 ng/ul 96.8 %Total 6.0308 nmole/L 366 Avg. Size (b.p.) 35.29 %CV

Sample Peak Width (sec): 50 Sample Min Peak Height: 25 Sample Baseline V to V?: Y Sample Baseline V to V pts: 3
Sample Filter: Binomial # of Pts for Filter: 3 Sample Start Region (min): 0 Sample End Region (min): 50
Manual Baseline Start (min): 10 Manual Baseline End (min): 48
Marker Peak Width (sec): 5 Marker Min Peak Height: 200 Marker Baseline V to V?: Y Marker Baseline V to V pts: 3
Lower Marker Selection: First Peak > 200 RFU Upper Marker Selection: Last Peak > 200 RFU
Ladder Size (bp): 1, 100, 200, 300, 400, 500, 600, 700, 800, 900, 1000, 1200, 1500, 2000, 3000, 6000
Quantification Using: Ladder Final Concentration (ng/uL): 0.0830 Dilution Factor: 12.0

Copyright 2015 Advanced Analytical Technologies, Inc.

Sample: 103613-001-060
Well Location: D8
Created: Friday, June 21, 2019 2:11:36 PM

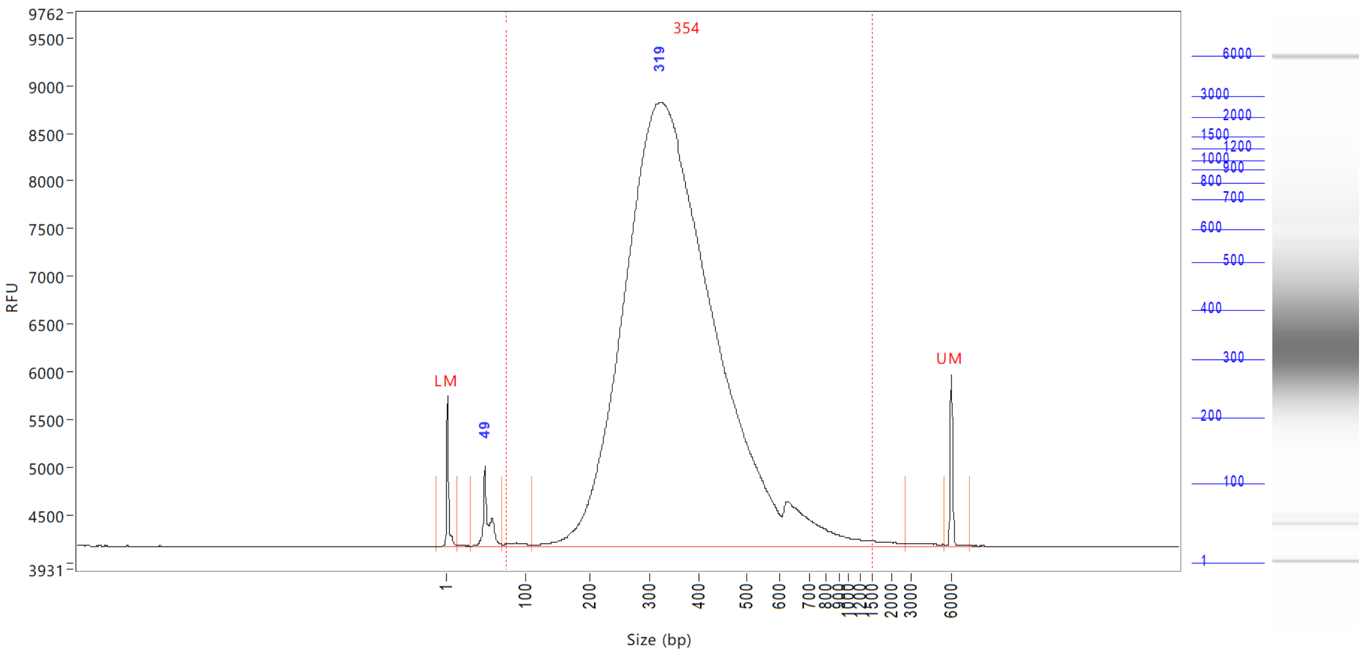

Peak	Size (bp)	Conc. (ng/uL)	From (bp)	To (bp)	RFU
1	1 (LM)	0.0124	0	14	1577
2	49	0.1843	32	71	846
3	319	12.6735	111	2734	4660
4	6000 (UM)	0.0085	5466	7339	1800

TIC:	12.8578	ng/uL
TIM:	64.2088	nmole/L
Total Conc.:	12.9071	ng/uL

Smear Analysis 75 bp to 1500 bp 12.6724 ng/ul 98.2 %Total 58.9463 nmole/L 354 Avg. Size (b.p.) 31.40 %CV

```
Sample Peak Width (sec): 50   Sample Min Peak Height: 25    Sample Baseline V to V?: Y    Sample Baseline V to V pts: 3
Sample Filter: Binomial       # of Pts for Filter: 3        Sample Start Region (min): 0  Sample End Region (min): 50
Manual Baseline Start (min): 10     Manual Baseline End (min): 48
Marker Peak Width (sec): 5    Marker Min Peak Height: 200   Marker Baseline V to V?: Y    Marker Baseline V to V pts: 3
Lower Marker Selection: First Peak > 200 RFU                Upper Marker Selection: Last Peak > 200 RFU
Ladder Size (bp): 1, 100, 200, 300, 400, 500, 600, 700, 800, 900, 1000, 1200, 1500, 2000, 3000, 6000
Quantification Using: Ladder          Final Concentration (ng/uL): 0.0830         Dilution Factor: 12.0
```

 Copyright 2015 Advanced Analytical Technologies, Inc.

Sample: 103613-001-061
Well Location: E8
Created: Friday, June 21, 2019 2:11:36 PM

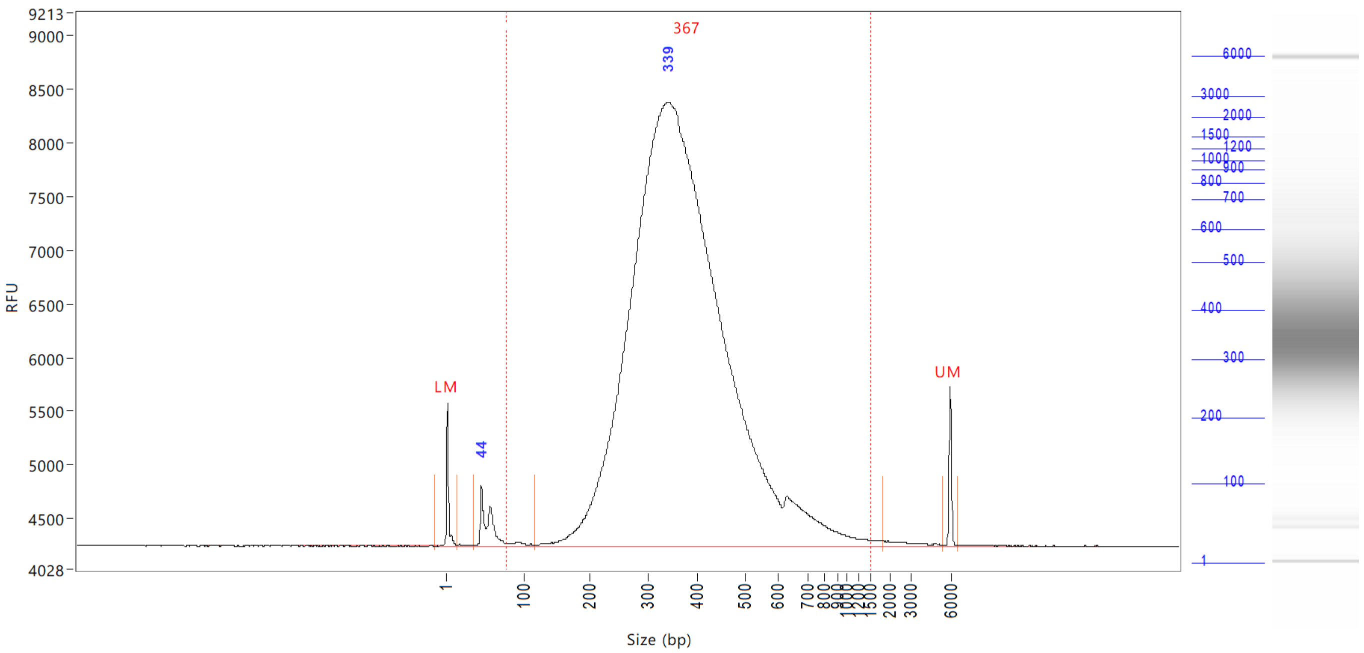

Peak	Size (bp)	Conc. (ng/uL)	From (bp)	To (bp)	RFU
1	1 (LM)	0.0124	0	15	1325
2	44	0.2453	35	115	571
3	339	13.3039	115	1813	4141
4	6000 (UM)	0.0082	5415	6581	1488
	TIC:	13.5492	ng/uL		
	TIM:	66.6564	nmole/L		
	Total Conc.:	13.5908	ng/uL		

Smear Analysis 75 bp to 1500 bp 13.3162 ng/ul 98.0 %Total 59.7602 nmole/L 367 Avg. Size (b.p.) 31.19 %CV

```
Sample Peak Width (sec): 50   Sample Min Peak Height: 25    Sample Baseline V to V?: Y    Sample Baseline V to V pts: 3
Sample Filter: Binomial       # of Pts for Filter: 3        Sample Start Region (min): 0  Sample End Region (min): 50
Manual Baseline Start (min): 10     Manual Baseline End (min): 48
Marker Peak Width (sec): 5    Marker Min Peak Height: 200   Marker Baseline V to V?: Y    Marker Baseline V to V pts: 3
Lower Marker Selection: First Peak > 200 RFU                Upper Marker Selection: Last Peak > 200 RFU
Ladder Size (bp): 1, 100, 200, 300, 400, 500, 600, 700, 800, 900, 1000, 1200, 1500, 2000, 3000, 6000
Quantification Using: Ladder          Final Concentration (ng/uL): 0.0830          Dilution Factor: 12.0
```


Copyright 2015 Advanced Analytical Technologies, Inc.

Sample: 103613-001-062
Well Location: F8
Created: Friday, June 21, 2019 2:11:36 PM

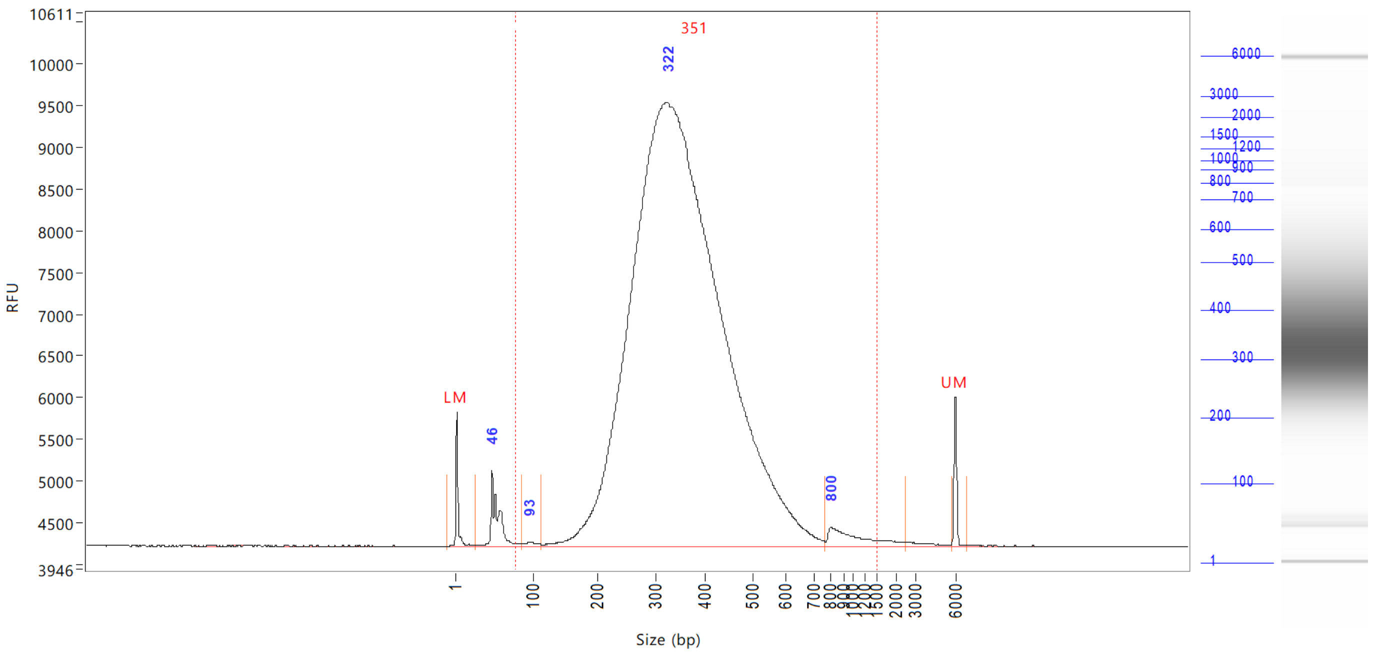

Peak	Size (bp)	Conc. (ng/uL)	From (bp)	To (bp)	RFU
1	1 (LM)	0.0124	0	26	1611
2	46	0.2483	26	84	905
3	93	0.0202	84	111	44
4	322	13.6600	111	767	5323
5	800	0.1563	767	2467	226
6	6000 (UM)	0.0078	5695	6834	1788

TIC:	14.0848	ng/uL
TIM:	73.1936	nmole/L
Total Conc.:	14.1077	ng/uL

Smear Analysis 75 bp to 1500 bp 13.8120 ng/ul 97.9 %Total 64.7943 nmole/L 351 Avg. Size (b.p.) 30.84 %CV

```
Sample Peak Width (sec): 50   Sample Min Peak Height: 25    Sample Baseline V to V?: Y    Sample Baseline V to V pts: 3
Sample Filter: Binomial       # of Pts for Filter: 3        Sample Start Region (min): 0  Sample End Region (min): 50
Manual Baseline Start (min): 10     Manual Baseline End (min): 48
Marker Peak Width (sec): 5    Marker Min Peak Height: 200   Marker Baseline V to V?: Y    Marker Baseline V to V pts: 3
Lower Marker Selection: First Peak > 200 RFU                Upper Marker Selection: Last Peak > 200 RFU
Ladder Size (bp): 1, 100, 200, 300, 400, 500, 600, 700, 800, 900, 1000, 1200, 1500, 2000, 3000, 6000
Quantification Using: Ladder          Final Concentration (ng/uL): 0.0830          Dilution Factor: 12.0
```


Copyright 2015 Advanced Analytical Technologies, Inc.

Sample: 103613-001-063
Well Location: G8
Created: Friday, June 21, 2019 2:11:36 PM

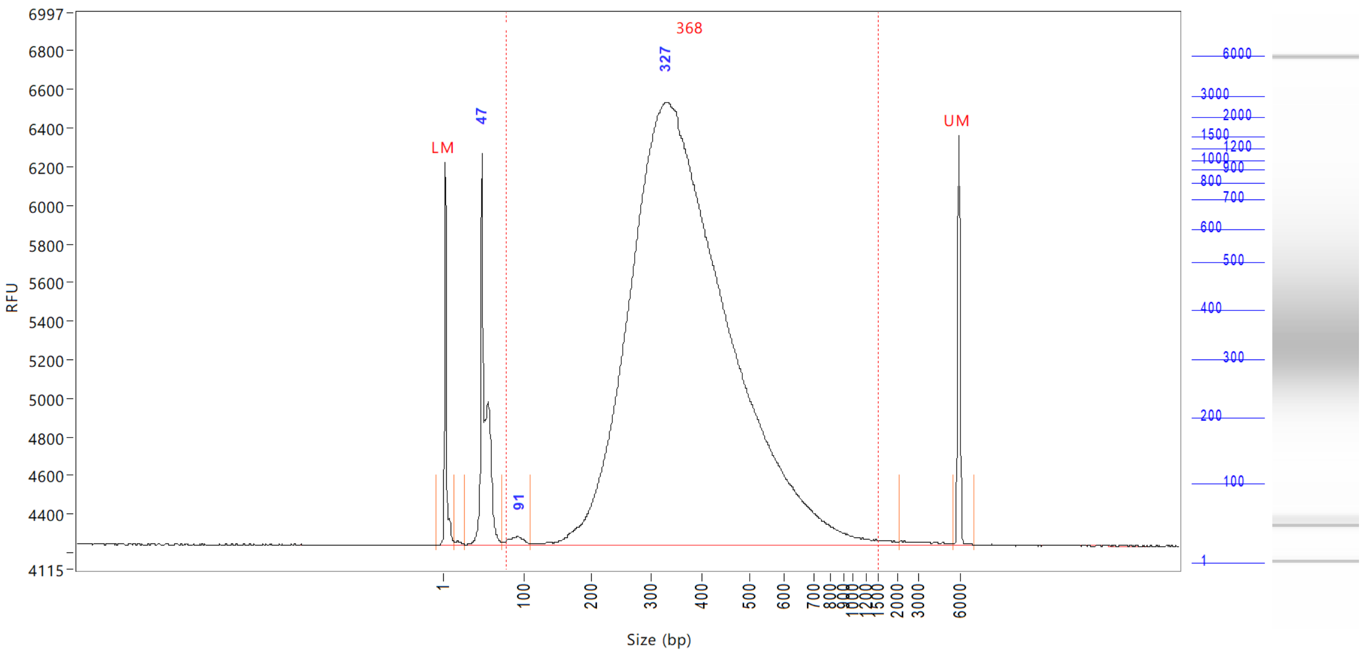

Peak	Size (bp)	Conc. (ng/uL)	From (bp)	To (bp)	RFU
1	1 (LM)	0.0124	0	14	1979
2	47	0.3443	26	72	2029
3	91	0.0228	72	109	44
4	327	5.4479	109	2068	2297
5	6000 (UM)	0.0079	5619	7061	2124

TIC:	5.8150	ng/uL
TIM:	35.8675	nmole/L
Total Conc.:	5.8320	ng/uL

Smear Analysis 75 bp to 1500 bp 5.4613 ng/ul 93.6 %Total 24.4461 nmole/L 368 Avg. Size (b.p.) 31.47 %CV

Sample Peak Width (sec): 50 Sample Min Peak Height: 25 Sample Baseline V to V?: Y Sample Baseline V to V pts: 3
Sample Filter: Binomial # of Pts for Filter: 3 Sample Start Region (min): 0 Sample End Region (min): 50
Manual Baseline Start (min): 10 Manual Baseline End (min): 48
Marker Peak Width (sec): 5 Marker Min Peak Height: 200 Marker Baseline V to V?: Y Marker Baseline V to V pts: 3
Lower Marker Selection: First Peak > 200 RFU Upper Marker Selection: Last Peak > 200 RFU
Ladder Size (bp): 1, 100, 200, 300, 400, 500, 600, 700, 800, 900, 1000, 1200, 1500, 2000, 3000, 6000
Quantification Using: Ladder Final Concentration (ng/uL): 0.0830 Dilution Factor: 12.0

Copyright 2015 Advanced Analytical Technologies, Inc.

Sample: 103613-001-064
Well Location: H8
Created: Friday, June 21, 2019 2:11:36 PM

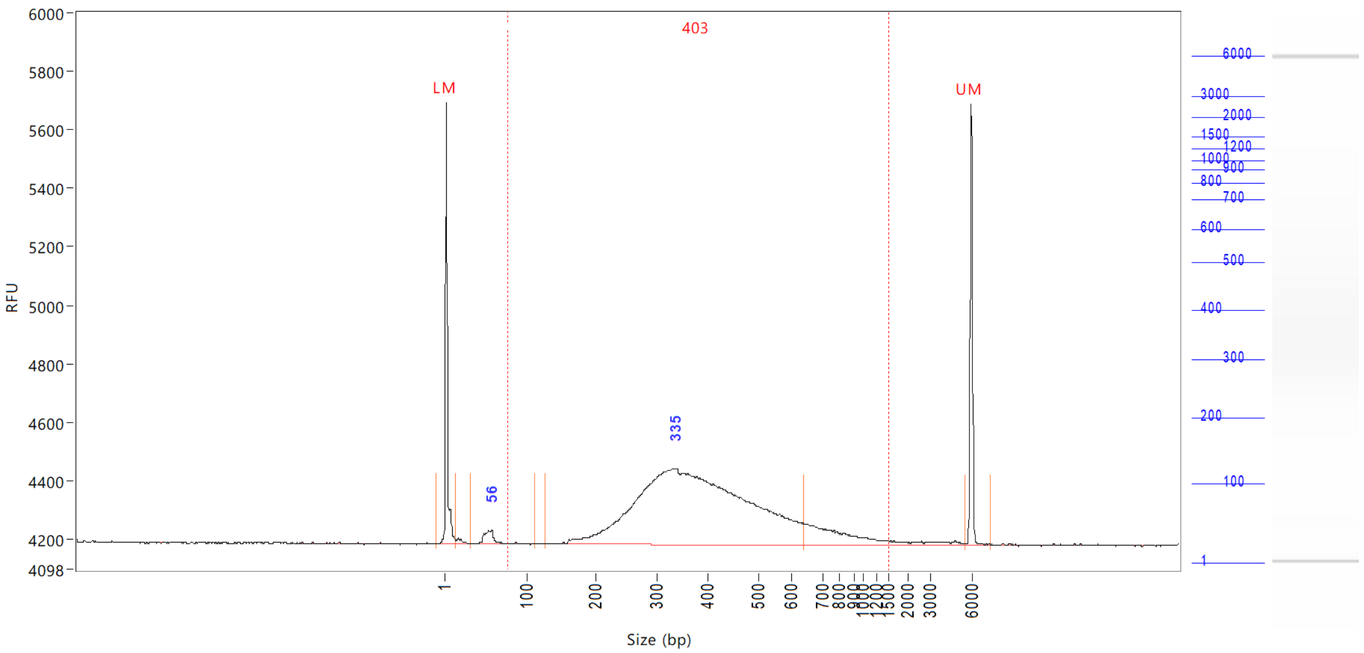

Peak	Size (bp)	Conc. (ng/uL)	From (bp)	To (bp)	RFU
1	1 (LM)	0.0124	0	14	1511
2	56	0.0223	32	110	49
3	335	0.8790	126	640	261
4	6000 (UM)	0.0071	5593	7389	1507
	TIC:	0.9012	ng/uL		
	TIM:	4.5385	nmole/L		
	Total Conc.:	0.9884	ng/uL		

Smear Analysis 75 bp to 1500 bp 0.9485 ng/ul 96.0 %Total 3.8766 nmole/L 403 Avg. Size (b.p.) 39.02 %CV

Sample Peak Width (sec): 50 Sample Min Peak Height: 25 Sample Baseline V to V?: Y Sample Baseline V to V pts: 3
Sample Filter: Binomial # of Pts for Filter: 3 Sample Start Region (min): 0 Sample End Region (min): 50
Manual Baseline Start (min): 10 Manual Baseline End (min): 48
Marker Peak Width (sec): 5 Marker Min Peak Height: 200 Marker Baseline V to V?: Y Marker Baseline V to V pts: 3
Lower Marker Selection: First Peak > 200 RFU Upper Marker Selection: Last Peak > 200 RFU
Ladder Size (bp): 1, 100, 200, 300, 400, 500, 600, 700, 800, 900, 1000, 1200, 1500, 2000, 3000, 6000
Quantification Using: Ladder Final Concentration (ng/uL): 0.0830 Dilution Factor: 12.0

 Copyright 2015 Advanced Analytical Technologies, Inc.

Sample: 103613-001-065
Well Location: A9
Created: Friday, June 21, 2019 2:11:36 PM

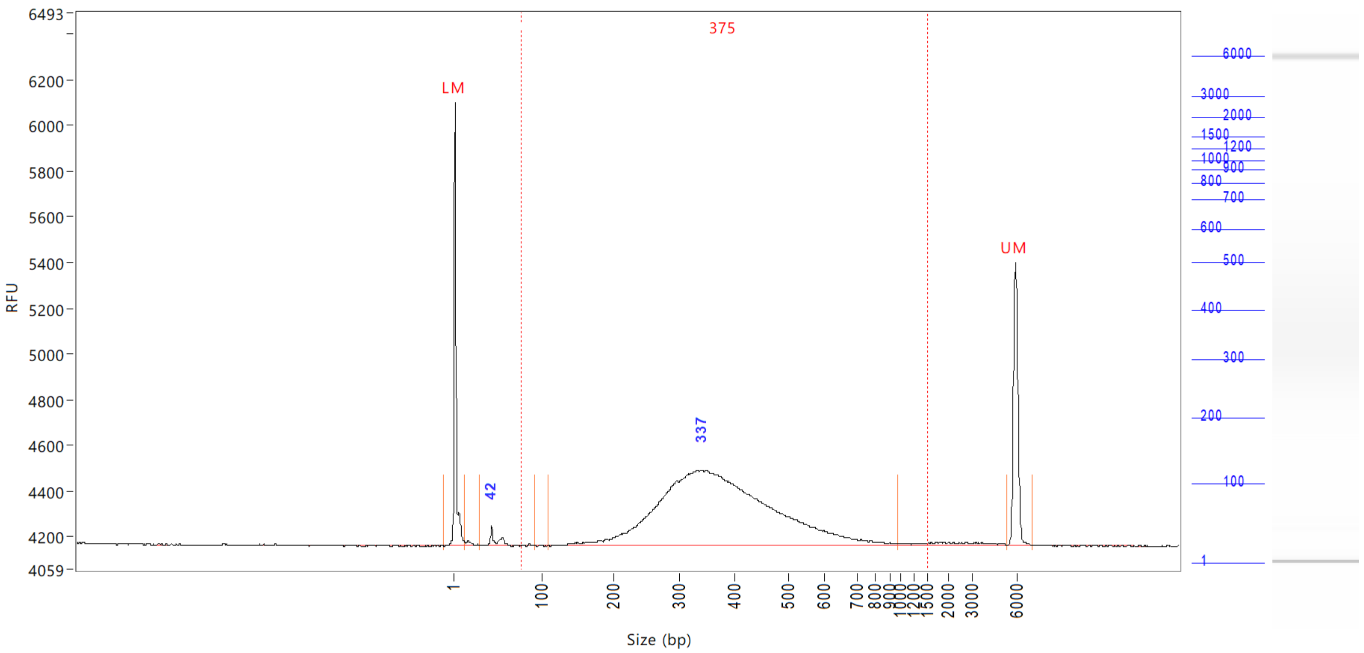

Peak	Size (bp)	Conc. (ng/uL)	From (bp)	To (bp)	RFU
1	1 (LM)	0.0124	0	13	1941
2	42	0.0140	30	92	87
3	337	0.8391	108	976	329
4	6000 (UM)	0.0075	5415	7162	1239

TIC:	0.8531	ng/uL
TIM:	4.1998	nmole/L
Total Conc.:	0.8733	ng/uL

Smear Analysis	75 bp to 1500 bp	0.8433 ng/ul	96.6 %Total	3.6973 nmole/L	375 Avg. Size (b.p.)	33.26 %CV

Sample Peak Width (sec): 50   Sample Min Peak Height: 25   Sample Baseline V to V?: Y   Sample Baseline V to V pts: 3
Sample Filter: Binomial   # of Pts for Filter: 3   Sample Start Region (min): 0   Sample End Region (min): 50
Manual Baseline Start (min): 10   Manual Baseline End (min): 48
Marker Peak Width (sec): 5   Marker Min Peak Height: 200   Marker Baseline V to V?: Y   Marker Baseline V to V pts: 3
Lower Marker Selection: First Peak > 200 RFU   Upper Marker Selection: Last Peak > 200 RFU
Ladder Size (bp): 1, 100, 200, 300, 400, 500, 600, 700, 800, 900, 1000, 1200, 1500, 2000, 3000, 6000
Quantification Using: Ladder   Final Concentration (ng/uL): 0.0830   Dilution Factor: 12.0

Copyright 2015 Advanced Analytical Technologies, Inc.

Sample: 103613-001-066
Well Location: B9
Created: Friday, June 21, 2019 2:11:36 PM

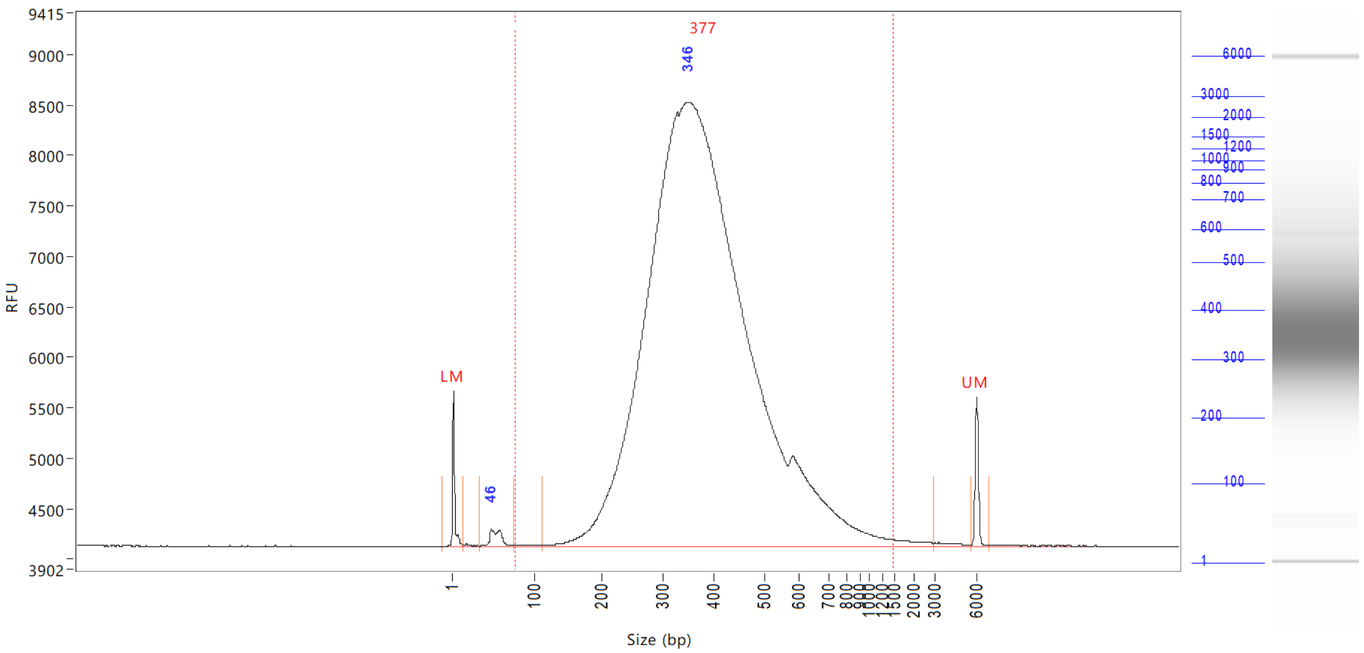

Peak	Size (bp)	Conc. (ng/uL)	From (bp)	To (bp)	RFU
1	1 (LM)	0.0124	0	14	1542
2	46	0.0841	34	74	173
3	346	12.8808	110	2917	4406
4	6000 (UM)	0.0083	5568	6909	1486

TIC:	12.9649	ng/uL
TIM:	58.1256	nmole/L
Total Conc.:	12.9977	ng/uL

Smear Analysis 75 bp to 1500 bp 12.8561 ng/ul 98.9 %Total 56.0885 nmole/L 377 Avg. Size (b.p.) 31.50 %CV

Sample Peak Width (sec): 50 Sample Min Peak Height: 25 Sample Baseline V to V?: Y Sample Baseline V to V pts: 3
Sample Filter: Binomial # of Pts for Filter: 3 Sample Start Region (min): 0 Sample End Region (min): 50
Manual Baseline Start (min): 10 Manual Baseline End (min): 48
Marker Peak Width (sec): 5 Marker Min Peak Height: 200 Marker Baseline V to V?: Y Marker Baseline V to V pts: 3
Lower Marker Selection: First Peak > 200 RFU Upper Marker Selection: Last Peak > 200 RFU
Ladder Size (bp): 1, 100, 200, 300, 400, 500, 600, 700, 800, 900, 1000, 1200, 1500, 2000, 3000, 6000
Quantification Using: Ladder Final Concentration (ng/uL): 0.0830 Dilution Factor: 12.0

Copyright 2015 Advanced Analytical Technologies, Inc.

Sample: 103613-001-067
Well Location: C9
Created: Friday, June 21, 2019 2:11:36 PM

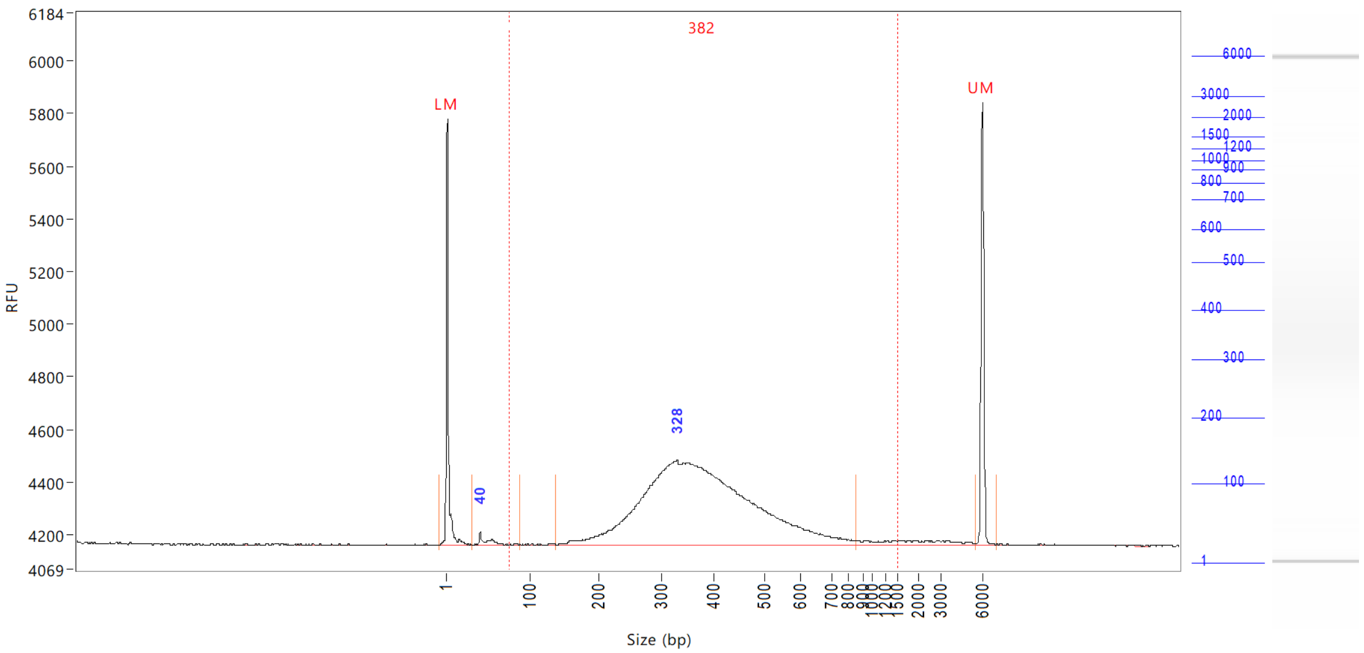

Peak	Size (bp)	Conc. (ng/uL)	From (bp)	To (bp)	RFU
1	1 (LM)	0.0124	0	31	1617
2	40	0.0129	31	87	52
3	328	0.9505	137	854	321
4	6000 (UM)	0.0076	5543	6935	1686

TIC:	0.9633	ng/uL
TIM:	4.6027	nmole/L
Total Conc.:	0.9950	ng/uL

Smear Analysis	75 bp to 1500 bp	0.9638 ng/ul	96.9 %Total	4.1531 nmole/L	382 Avg. Size (b.p.)	35.62 %CV

Sample Peak Width (sec): 50 Sample Min Peak Height: 25 Sample Baseline V to V?: Y Sample Baseline V to V pts: 3
Sample Filter: Binomial # of Pts for Filter: 3 Sample Start Region (min): 0 Sample End Region (min): 50
Manual Baseline Start (min): 10 Manual Baseline End (min): 48
Marker Peak Width (sec): 5 Marker Min Peak Height: 200 Marker Baseline V to V?: Y Marker Baseline V to V pts: 3
Lower Marker Selection: First Peak > 200 RFU Upper Marker Selection: Last Peak > 200 RFU
Ladder Size (bp): 1, 100, 200, 300, 400, 500, 600, 700, 800, 900, 1000, 1200, 1500, 2000, 3000, 6000
Quantification Using: Ladder Final Concentration (ng/uL): 0.0830 Dilution Factor: 12.0

 Copyright 2015 Advanced Analytical Technologies, Inc.

Sample: 103613-001-068
Well Location: D9
Created: Friday, June 21, 2019 2:11:36 PM

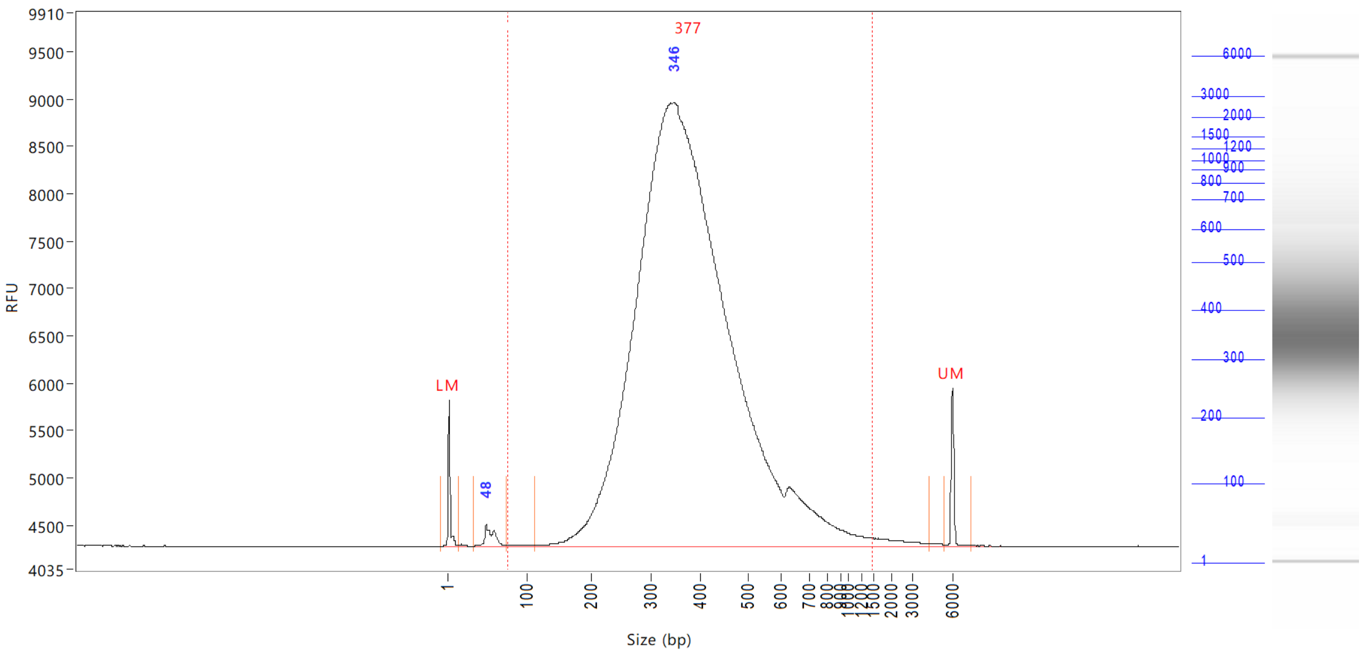

Peak	Size (bp)	Conc. (ng/uL)	From (bp)	To (bp)	RFU
1	1 (LM)	0.0124	0	15	1544
2	48	0.0904	34	74	230
3	346	13.3757	112	4221	4693
4	6000 (UM)	0.0086	5390	7389	1672
	TIC:	13.4661	ng/uL		
	TIM:	59.8072	nmole/L		
	Total Conc.:	13.4881	ng/uL		

Smear Analysis 75 bp to 1500 bp 13.3265 ng/ul 98.8 %Total 58.1605 nmole/L 377 Avg. Size (b.p.) 31.72 %CV

Sample Peak Width (sec): 50 Sample Min Peak Height: 25 Sample Baseline V to V?: Y Sample Baseline V to V pts: 3
Sample Filter: Binomial # of Pts for Filter: 3 Sample Start Region (min): 0 Sample End Region (min): 50
Manual Baseline Start (min): 10 Manual Baseline End (min): 48
Marker Peak Width (sec): 5 Marker Min Peak Height: 200 Marker Baseline V to V?: Y Marker Baseline V to V pts: 3
Lower Marker Selection: First Peak > 200 RFU Upper Marker Selection: Last Peak > 200 RFU
Ladder Size (bp): 1, 100, 200, 300, 400, 500, 600, 700, 800, 900, 1000, 1200, 1500, 2000, 3000, 6000
Quantification Using: Ladder Final Concentration (ng/uL): 0.0830 Dilution Factor: 12.0

Copyright 2015 Advanced Analytical Technologies, Inc.

Sample: 103613-001-069
Well Location: E9
Created: Friday, June 21, 2019 2:11:36 PM

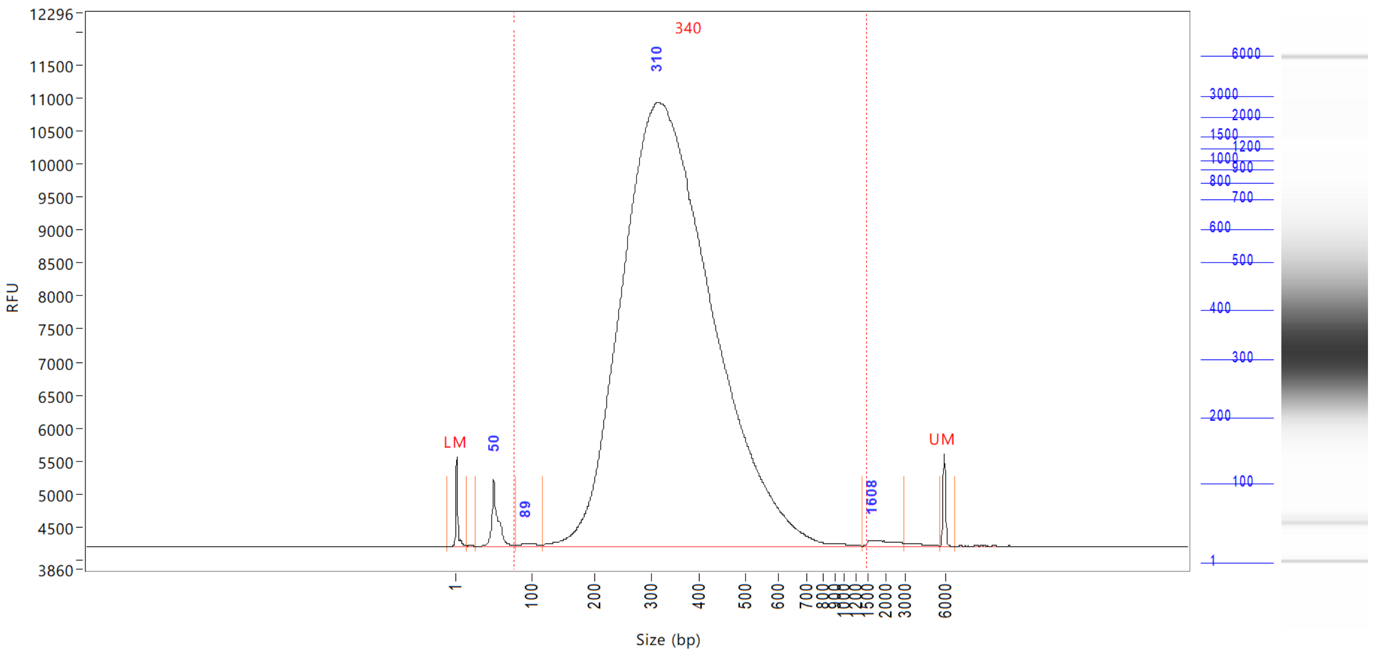

Peak	Size (bp)	Conc. (ng/uL)	From (bp)	To (bp)	RFU
1	1 (LM)	0.0124	0	16	1365
2	50	0.3054	27	79	1028
3	89	0.0397	79	118	47
4	310	21.9774	118	1373	6745
5	1608	0.0628	1373	2967	85
6	6000 (UM)	0.0075	5670	6859	1395
	TIC:	22.3854	ng/uL		
	TIM:	116.6835	nmole/L		
	Total Conc.:	22.4120	ng/uL		

Smear Analysis	75 bp to 1500 bp	22.0210 ng/ul	98.3 %Total	106.5466 nmole/L	340 Avg. Size (b.p.)	27.75 %CV

Sample Peak Width (sec): 50 Sample Min Peak Height: 25 Sample Baseline V to V?: Y Sample Baseline V to V pts: 3
Sample Filter: Binomial # of Pts for Filter: 3 Sample Start Region (min): 0 Sample End Region (min): 50
Manual Baseline Start (min): 10 Manual Baseline End (min): 48
Marker Peak Width (sec): 5 Marker Min Peak Height: 200 Marker Baseline V to V?: Y Marker Baseline V to V pts: 3
Lower Marker Selection: First Peak > 200 RFU Upper Marker Selection: Last Peak > 200 RFU
Ladder Size (bp): 1, 100, 200, 300, 400, 500, 600, 700, 800, 900, 1000, 1200, 1500, 2000, 3000, 6000
Quantification Using: Ladder Final Concentration (ng/uL): 0.0830 Dilution Factor: 12.0

Copyright 2015 Advanced Analytical Technologies, Inc.

Sample: 103613-001-070
Well Location: F9
Created: Friday, June 21, 2019 2:11:36 PM

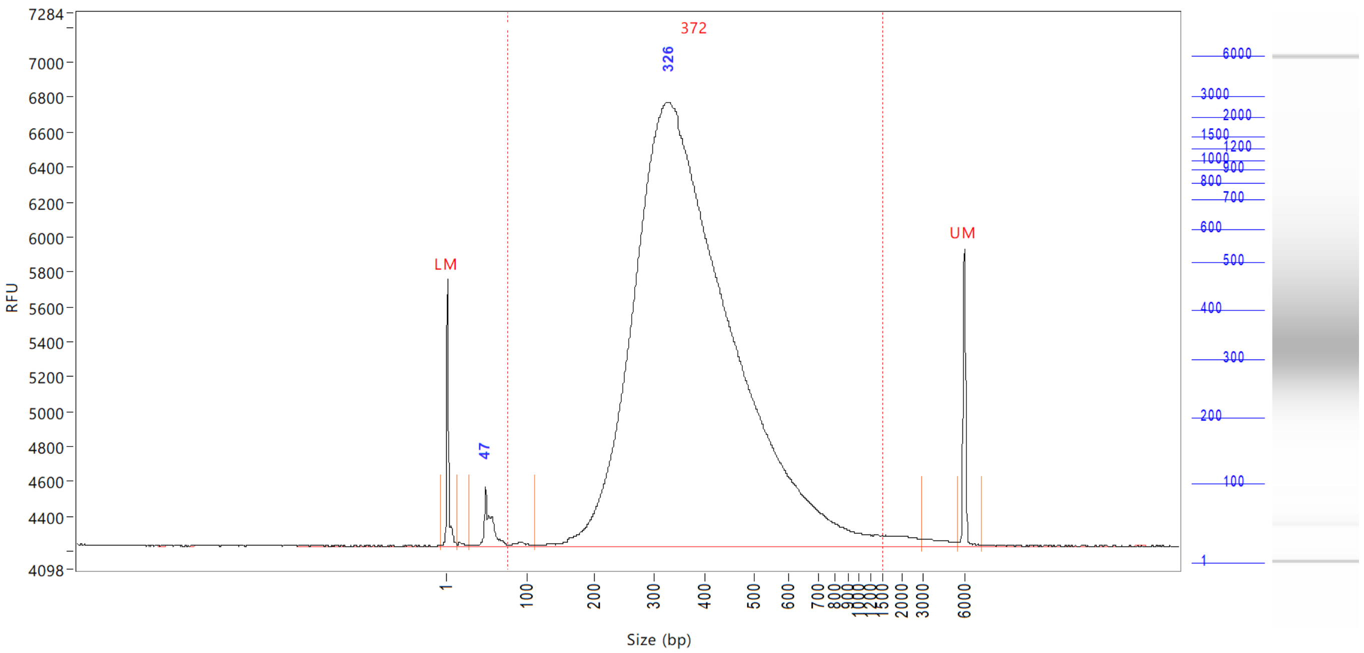

Peak	Size (bp)	Conc. (ng/uL)	From (bp)	To (bp)	RFU
1	1 (LM)	0.0124	0	14	1531
2	47	0.1085	29	111	343
3	326	7.3158	111	2933	2545
4	6000 (UM)	0.0080	5517	7238	1703

TIC:	7.4242	ng/uL
TIM:	34.6272	nmole/L
Total Conc.:	7.4506	ng/uL

Smear Analysis 75 bp to 1500 bp 7.2870 ng/ul 97.8 %Total 32.2003 nmole/L 372 Avg. Size (b.p.) 33.00 %CV

Sample Peak Width (sec): 50 Sample Min Peak Height: 25 Sample Baseline V to V?: Y Sample Baseline V to V pts: 3
Sample Filter: Binomial # of Pts for Filter: 3 Sample Start Region (min): 0 Sample End Region (min): 50
Manual Baseline Start (min): 10 Manual Baseline End (min): 48
Marker Peak Width (sec): 5 Marker Min Peak Height: 200 Marker Baseline V to V?: Y Marker Baseline V to V pts: 3
Lower Marker Selection: First Peak > 200 RFU Upper Marker Selection: Last Peak > 200 RFU
Ladder Size (bp): 1, 100, 200, 300, 400, 500, 600, 700, 800, 900, 1000, 1200, 1500, 2000, 3000, 6000
Quantification Using: Ladder Final Concentration (ng/uL): 0.0830 Dilution Factor: 12.0

 Copyright 2015 Advanced Analytical Technologies, Inc.

Sample: 103613-001-071
Well Location: G9
Created: Friday, June 21, 2019 2:11:36 PM

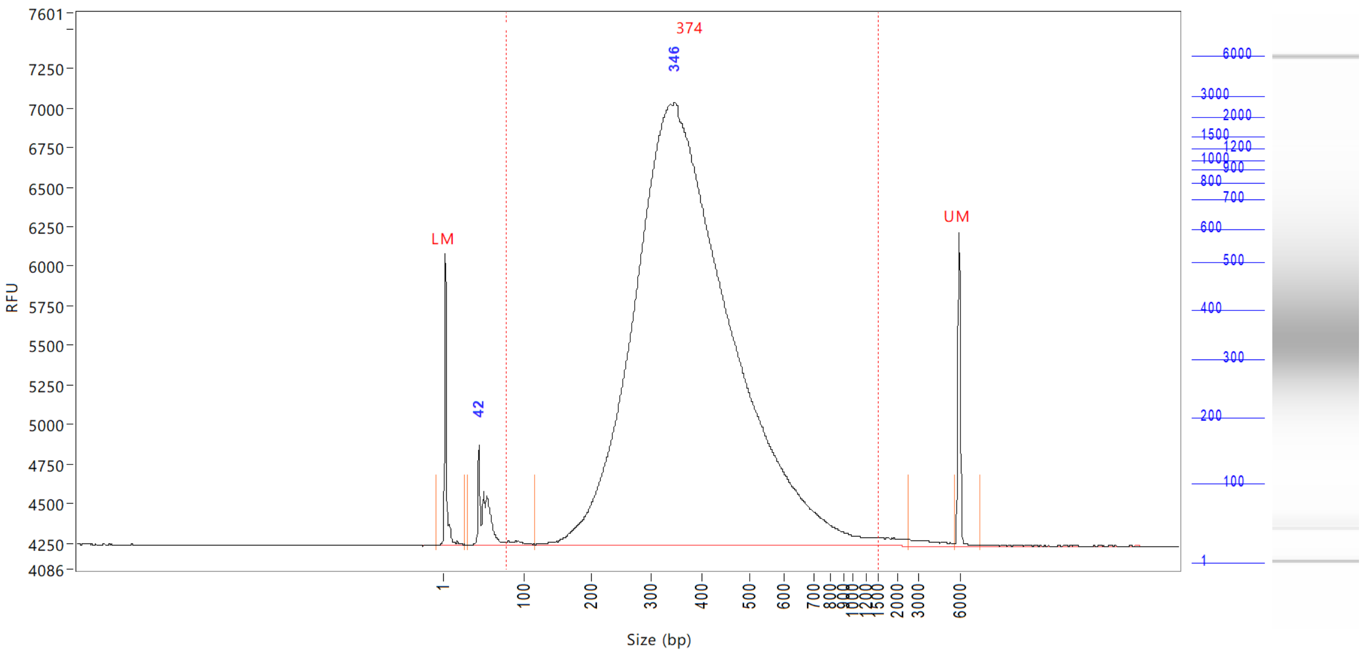

Peak	Size (bp)	Conc. (ng/uL)	From (bp)	To (bp)	RFU
1	1 (LM)	0.0124	0	26	1846
2	42	0.1644	29	115	634
3	346	6.7812	115	2534	2804
4	6000 (UM)	0.0077	5670	7541	1977

TIC:	6.9456	ng/uL
TIM:	34.3469	nmole/L
Total Conc.:	6.9654	ng/uL

Smear Analysis	75 bp to 1500 bp	6.7729 ng/ul	97.2 %Total	29.7789 nmole/L	374 Avg. Size (b.p.)	31.70 %CV

Sample Peak Width (sec): 50 Sample Min Peak Height: 25 Sample Baseline V to V?: Y Sample Baseline V to V pts: 3
Sample Filter: Binomial # of Pts for Filter: 3 Sample Start Region (min): 0 Sample End Region (min): 50
Manual Baseline Start (min): 10 Manual Baseline End (min): 48
Marker Peak Width (sec): 5 Marker Min Peak Height: 200 Marker Baseline V to V?: Y Marker Baseline V to V pts: 3
Lower Marker Selection: First Peak > 200 RFU Upper Marker Selection: Last Peak > 200 RFU
Ladder Size (bp): 1, 100, 200, 300, 400, 500, 600, 700, 800, 900, 1000, 1200, 1500, 2000, 3000, 6000
Quantification Using: Ladder Final Concentration (ng/uL): 0.0830 Dilution Factor: 12.0

Copyright 2015 Advanced Analytical Technologies, Inc.

Sample: 103613-001-072
Well Location: H9
Created: Friday, June 21, 2019 2:11:36 PM

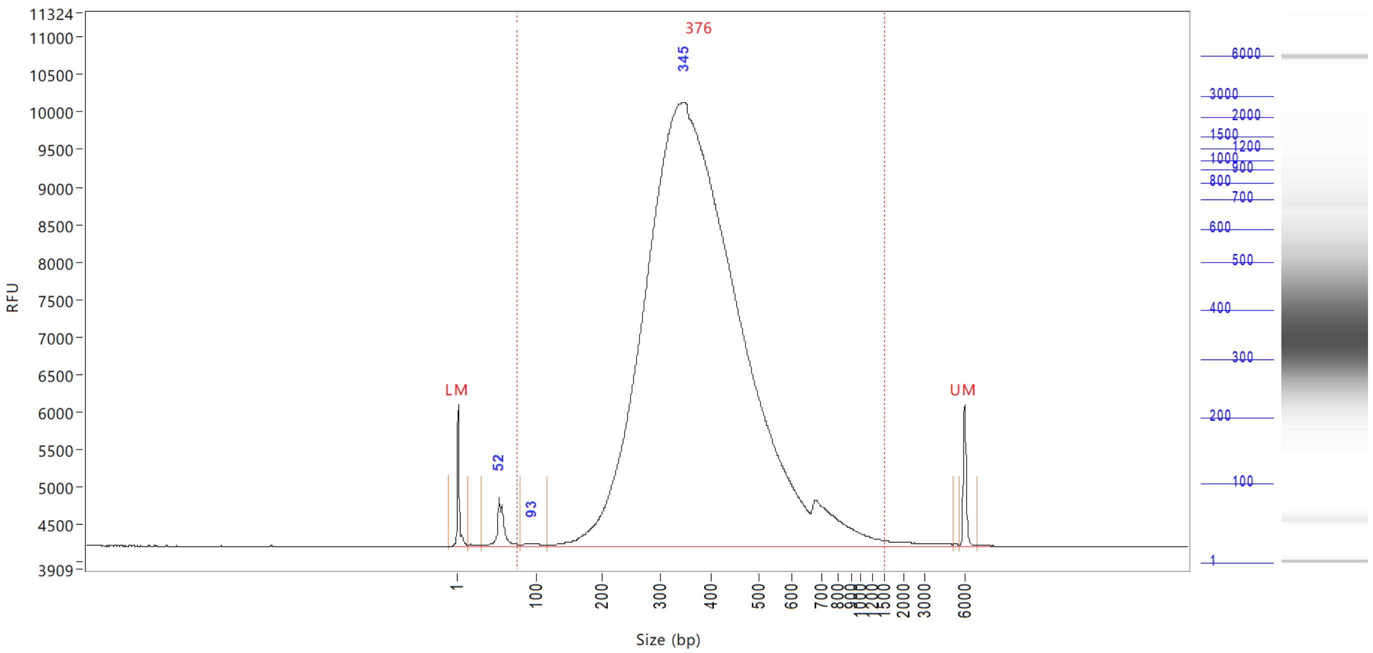

Peak	Size (bp)	Conc. (ng/uL)	From (bp)	To (bp)	RFU
1	1 (LM)	0.0124	0	14	1884
2	52	0.1534	31	79	650
3	93	0.0209	79	116	40
4	345	14.1495	116	5212	5924
5	6000 (UM)	0.0085	5593	6960	1900

TIC:	14.3238	ng/uL
TIM:	65.5550	nmole/L
Total Conc.:	14.3310	ng/uL

Smear Analysis	75 bp to 1500 bp	14.1217 ng/ul	98.5 %Total	61.8132 nmole/L	376 Avg. Size (b.p.)	31.38 %CV

Sample Peak Width (sec): 50 Sample Min Peak Height: 25 Sample Baseline V to V?: Y Sample Baseline V to V pts: 3
Sample Filter: Binomial # of Pts for Filter: 3 Sample Start Region (min): 0 Sample End Region (min): 50
Manual Baseline Start (min): 10 Manual Baseline End (min): 48
Marker Peak Width (sec): 5 Marker Min Peak Height: 200 Marker Baseline V to V?: Y Marker Baseline V to V pts: 3
Lower Marker Selection: First Peak > 200 RFU Upper Marker Selection: Last Peak > 200 RFU
Ladder Size (bp): 1, 100, 200, 300, 400, 500, 600, 700, 800, 900, 1000, 1200, 1500, 2000, 3000, 6000
Quantification Using: Ladder Final Concentration (ng/uL): 0.0830 Dilution Factor: 12.0

Copyright 2015 Advanced Analytical Technologies, Inc.

Sample: 103613-001-073
Well Location: A10
Created: Friday, June 21, 2019 2:11:36 PM

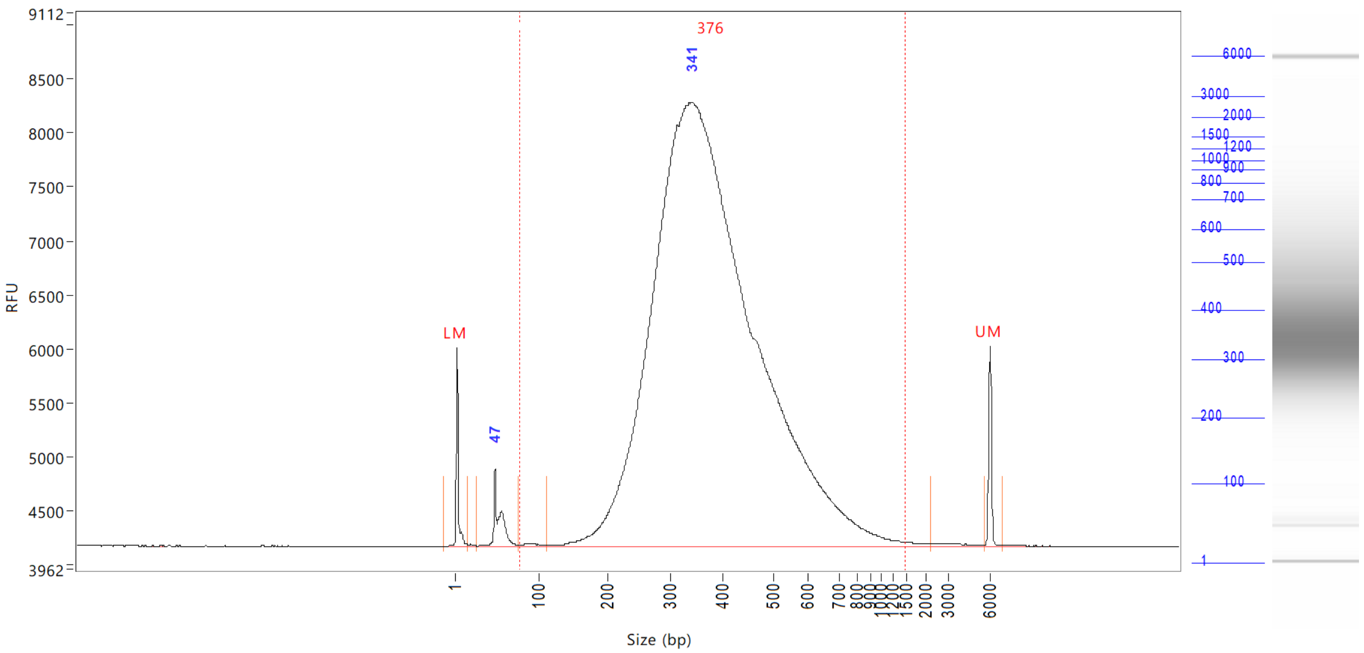

Peak	Size (bp)	Conc. (ng/uL)	From (bp)	To (bp)	RFU
1	1 (LM)	0.0124	0	15	1839
2	47	0.1479	26	74	714
3	341	10.2593	111	2251	4115
4	6000 (UM)	0.0081	5593	6884	1852

TIC:	10.4072	ng/uL
TIM:	49.4044	nmole/L
Total Conc.:	10.4428	ng/uL

Smear Analysis	75 bp to 1500 bp	10.2613 ng/ul	98.3 %Total	44.9589 nmole/L	376 Avg. Size (b.p.)	30.83 %CV

Sample Peak Width (sec): 50 Sample Min Peak Height: 25 Sample Baseline V to V?: Y Sample Baseline V to V pts: 3
Sample Filter: Binomial # of Pts for Filter: 3 Sample Start Region (min): 0 Sample End Region (min): 50
Manual Baseline Start (min): 10 Manual Baseline End (min): 48
Marker Peak Width (sec): 5 Marker Min Peak Height: 200 Marker Baseline V to V?: Y Marker Baseline V to V pts: 3
Lower Marker Selection: First Peak > 200 RFU Upper Marker Selection: Last Peak > 200 RFU
Ladder Size (bp): 1, 100, 200, 300, 400, 500, 600, 700, 800, 900, 1000, 1200, 1500, 2000, 3000, 6000
Quantification Using: Ladder Final Concentration (ng/uL): 0.0830 Dilution Factor: 12.0

Copyright 2015 Advanced Analytical Technologies, Inc.

Sample: 103613-001-074
Well Location: B10
Created: Friday, June 21, 2019 2:11:36 PM

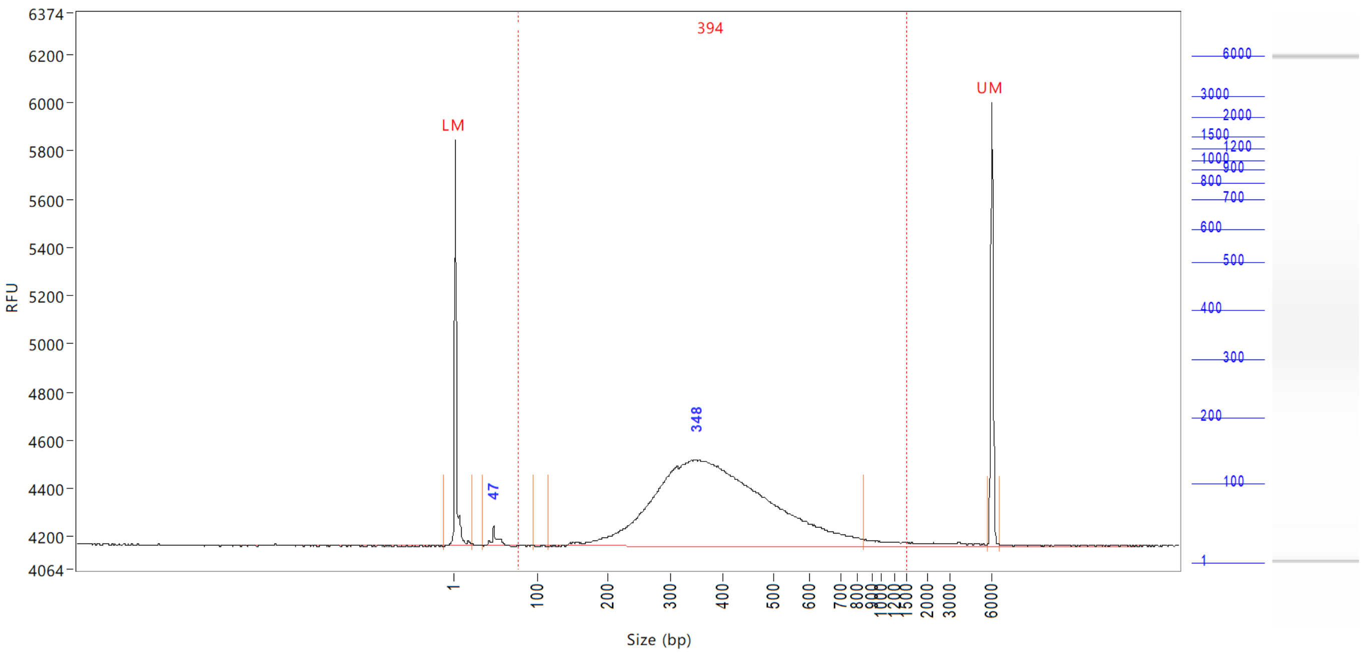

Peak	Size (bp)	Conc. (ng/uL)	From (bp)	To (bp)	RFU
1	1 (LM)	0.0124	0	21	1686
2	47	0.0172	34	95	84
3	348	1.0716	114	837	358
4	6000 (UM)	0.0080	5670	6606	1845

TIC:	1.0888	ng/uL
TIM:	5.1425	nmole/L
Total Conc.:	1.1176	ng/uL

Smear Analysis	75 bp to 1500 bp	1.0866 ng/ul	97.2 %Total	4.5403 nmole/L	394 Avg. Size (b.p.)	35.06 %CV

Sample Peak Width (sec): 50 Sample Min Peak Height: 25 Sample Baseline V to V?: Y Sample Baseline V to V pts: 3
Sample Filter: Binomial # of Pts for Filter: 3 Sample Start Region (min): 0 Sample End Region (min): 50
Manual Baseline Start (min): 10 Manual Baseline End (min): 48
Marker Peak Width (sec): 5 Marker Min Peak Height: 200 Marker Baseline V to V?: Y Marker Baseline V to V pts: 3
Lower Marker Selection: First Peak > 200 RFU Upper Marker Selection: Last Peak > 200 RFU
Ladder Size (bp): 1, 100, 200, 300, 400, 500, 600, 700, 800, 900, 1000, 1200, 1500, 2000, 3000, 6000
Quantification Using: Ladder Final Concentration (ng/uL): 0.0830 Dilution Factor: 12.0

Copyright 2015 Advanced Analytical Technologies, Inc.

Sample: 103613-001-075
Well Location: C10
Created: Friday, June 21, 2019 2:11:36 PM

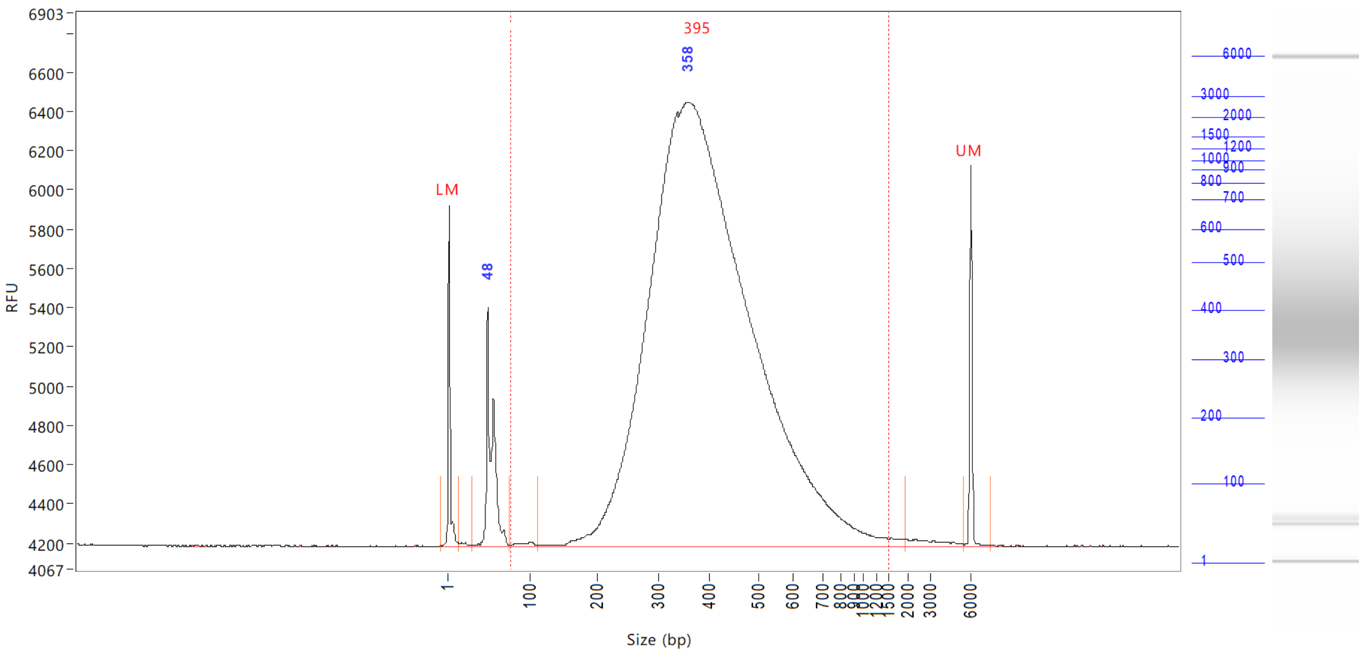

Peak	Size (bp)	Conc. (ng/uL)	From (bp)	To (bp)	RFU
1	1 (LM)	0.0124	0	15	1734
2	48	0.2747	29	75	1218
3	358	5.9964	111	1938	2266
4	6000 (UM)	0.0080	5517	7389	1943

TIC:	6.2711	ng/uL
TIM:	33.2791	nmole/L
Total Conc.:	6.3102	ng/uL

Smear Analysis 75 bp to 1500 bp 5.9970 ng/ul 95.0 %Total 25.0038 nmole/L 395 Avg. Size (b.p.) 30.74 %CV

Sample Peak Width (sec): 50 Sample Min Peak Height: 25 Sample Baseline V to V?: Y Sample Baseline V to V pts: 3
Sample Filter: Binomial # of Pts for Filter: 3 Sample Start Region (min): 0 Sample End Region (min): 50
Manual Baseline Start (min): 10 Manual Baseline End (min): 48
Marker Peak Width (sec): 5 Marker Min Peak Height: 200 Marker Baseline V to V?: Y Marker Baseline V to V pts: 3
Lower Marker Selection: First Peak > 200 RFU Upper Marker Selection: Last Peak > 200 RFU
Ladder Size (bp): 1, 100, 200, 300, 400, 500, 600, 700, 800, 900, 1000, 1200, 1500, 2000, 3000, 6000
Quantification Using: Ladder Final Concentration (ng/uL): 0.0830 Dilution Factor: 12.0

 Copyright 2015 Advanced Analytical Technologies, Inc.

Sample: 103613-001-076
Well Location: D10
Created: Friday, June 21, 2019 2:11:36 PM

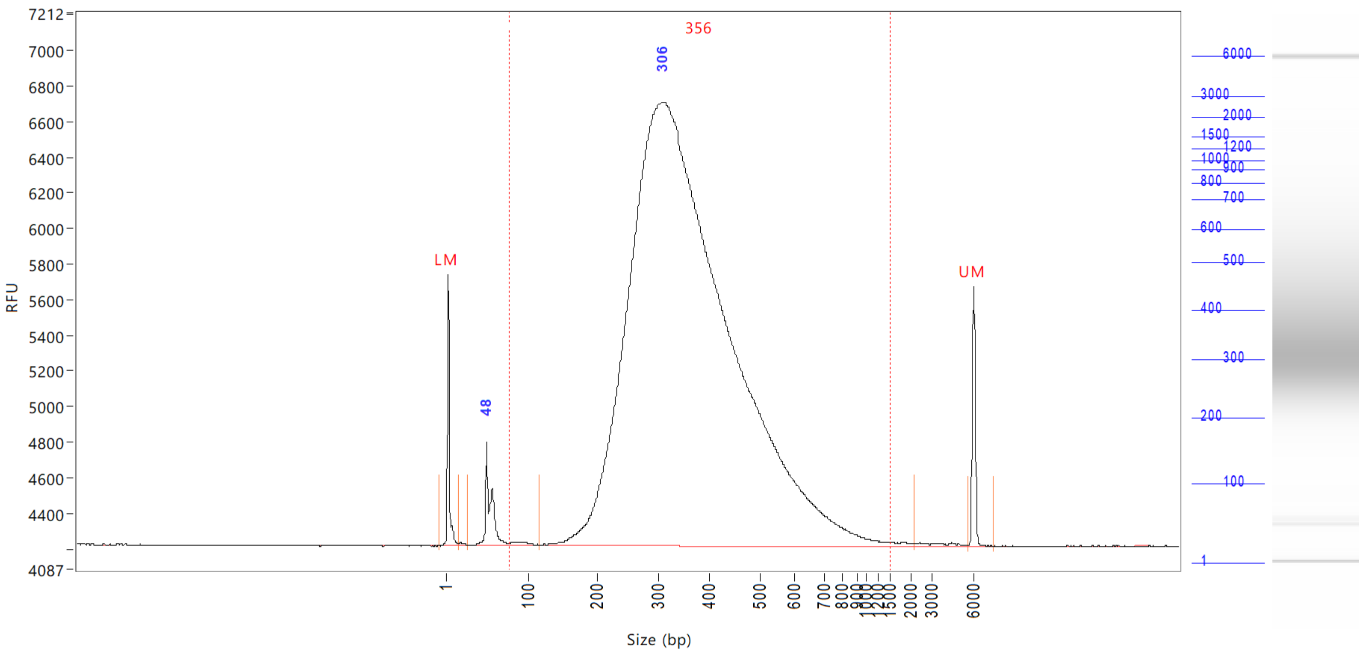

Peak	Size (bp)	Conc. (ng/uL)	From (bp)	To (bp)	RFU
1	1 (LM)	0.0124	0	14	1521
2	48	0.1551	26	114	579
3	306	7.4241	114	2184	2494
4	6000 (UM)	0.0077	5568	7415	1457

TIC:	7.5792	ng/uL
TIM:	38.6560	nmole/L
Total Conc.:	7.5949	ng/uL

Smear Analysis 75 bp to 1500 bp 7.4319 ng/ul 97.9 %Total 34.3894 nmole/L 356 Avg. Size (b.p.) 31.87 %CV

Sample Peak Width (sec): 50 Sample Min Peak Height: 25 Sample Baseline V to V?: Y Sample Baseline V to V pts: 3
Sample Filter: Binomial # of Pts for Filter: 3 Sample Start Region (min): 0 Sample End Region (min): 50
Manual Baseline Start (min): 10 Manual Baseline End (min): 48
Marker Peak Width (sec): 5 Marker Min Peak Height: 200 Marker Baseline V to V?: Y Marker Baseline V to V pts: 3
Lower Marker Selection: First Peak > 200 RFU Upper Marker Selection: Last Peak > 200 RFU
Ladder Size (bp): 1, 100, 200, 300, 400, 500, 600, 700, 800, 900, 1000, 1200, 1500, 2000, 3000, 6000
Quantification Using: Ladder Final Concentration (ng/uL): 0.0830 Dilution Factor: 12.0

 Copyright 2015 Advanced Analytical Technologies, Inc.

Sample: 103613-001-077
Well Location: E10
Created: Friday, June 21, 2019 2:11:36 PM

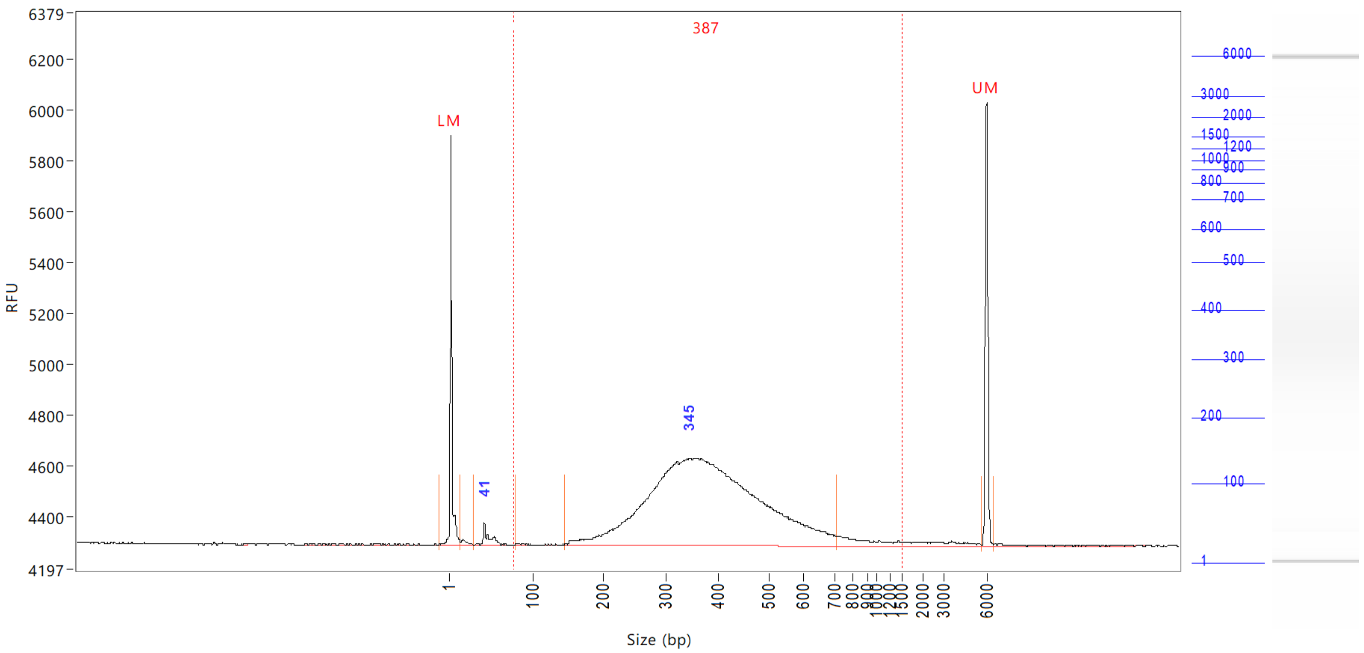

Peak	Size (bp)	Conc. (ng/uL)	From (bp)	To (bp)	RFU
1	1 (LM)	0.0124	0	13	1607
2	41	0.0222	28	79	89
3	345	1.0894	144	709	343
4	6000 (UM)	0.0077	5619	6480	1743

TIC:	1.1116	ng/uL
TIM:	5.5660	nmole/L
Total Conc.:	1.1688	ng/uL

Smear Analysis 75 bp to 1500 bp 1.1220 ng/ul 96.0 %Total 4.7726 nmole/L 387 Avg. Size (b.p.) 35.89 %CV

Sample Peak Width (sec): 50 Sample Min Peak Height: 25 Sample Baseline V to V?: Y Sample Baseline V to V pts: 3
Sample Filter: Binomial # of Pts for Filter: 3 Sample Start Region (min): 0 Sample End Region (min): 50
Manual Baseline Start (min): 10 Manual Baseline End (min): 48
Marker Peak Width (sec): 5 Marker Min Peak Height: 200 Marker Baseline V to V?: Y Marker Baseline V to V pts: 3
Lower Marker Selection: First Peak > 200 RFU Upper Marker Selection: Last Peak > 200 RFU
Ladder Size (bp): 1, 100, 200, 300, 400, 500, 600, 700, 800, 900, 1000, 1200, 1500, 2000, 3000, 6000
Quantification Using: Ladder Final Concentration (ng/uL): 0.0830 Dilution Factor: 12.0

 Copyright 2015 Advanced Analytical Technologies, Inc.

Sample: 103613-001-078
Well Location: F10
Created: Friday, June 21, 2019 2:11:36 PM

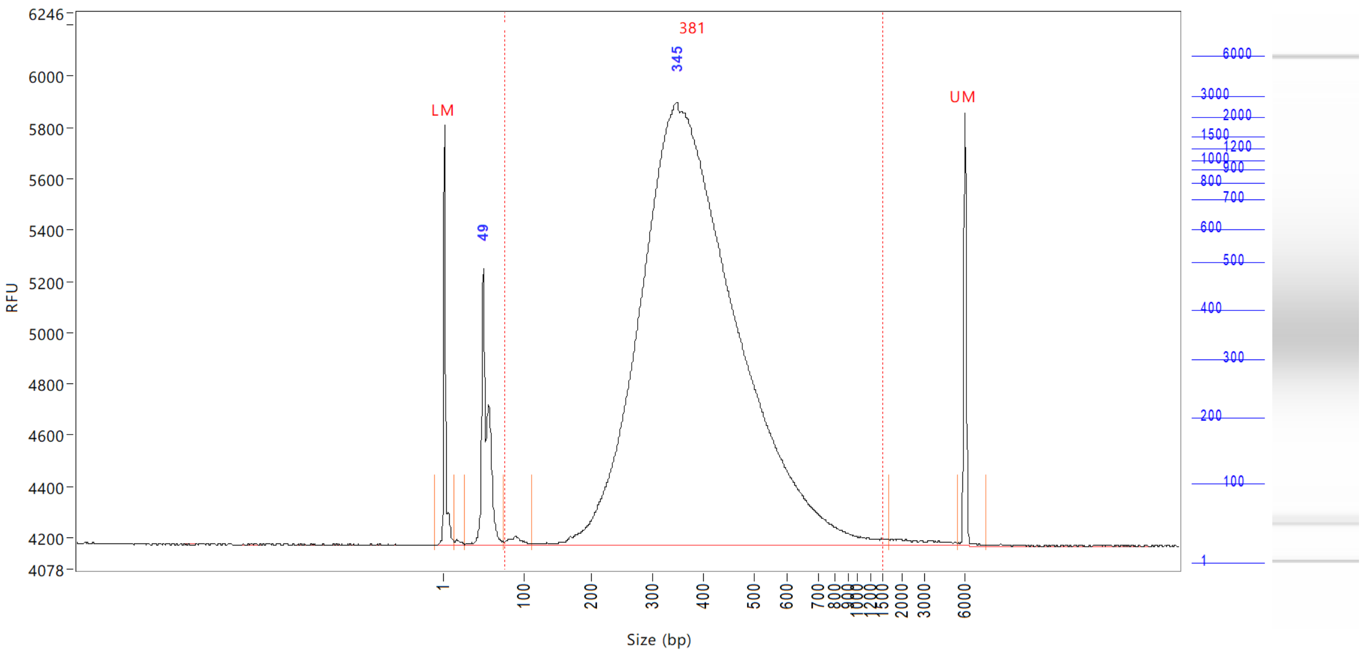

Peak	Size (bp)	Conc. (ng/uL)	From (bp)	To (bp)	RFU
1	1 (LM)	0.0124	0	15	1637
2	49	0.2686	26	74	1076
3	345	4.5942	110	1652	1728
4	6000 (UM)	0.0074	5441	7490	1684

TIC:	4.8628	ng/uL
TIM:	28.1972	nmole/L
Total Conc.:	4.9059	ng/uL

Smear Analysis	75 bp to 1500 bp	4.6076 ng/ul	93.9 %Total	19.9056 nmole/L	381 Avg. Size (b.p.)	30.12 %CV

Sample Peak Width (sec): 50 Sample Min Peak Height: 25 Sample Baseline V to V?: Y Sample Baseline V to V pts: 3
Sample Filter: Binomial # of Pts for Filter: 3 Sample Start Region (min): 0 Sample End Region (min): 50
Manual Baseline Start (min): 10 Manual Baseline End (min): 48
Marker Peak Width (sec): 5 Marker Min Peak Height: 200 Marker Baseline V to V?: Y Marker Baseline V to V pts: 3
Lower Marker Selection: First Peak > 200 RFU Upper Marker Selection: Last Peak > 200 RFU
Ladder Size (bp): 1, 100, 200, 300, 400, 500, 600, 700, 800, 900, 1000, 1200, 1500, 2000, 3000, 6000
Quantification Using: Ladder Final Concentration (ng/uL): 0.0830 Dilution Factor: 12.0

 Copyright 2015 Advanced Analytical Technologies, Inc.

Sample: 103613-001-079
Well Location: G10
Created: Friday, June 21, 2019 2:11:36 PM

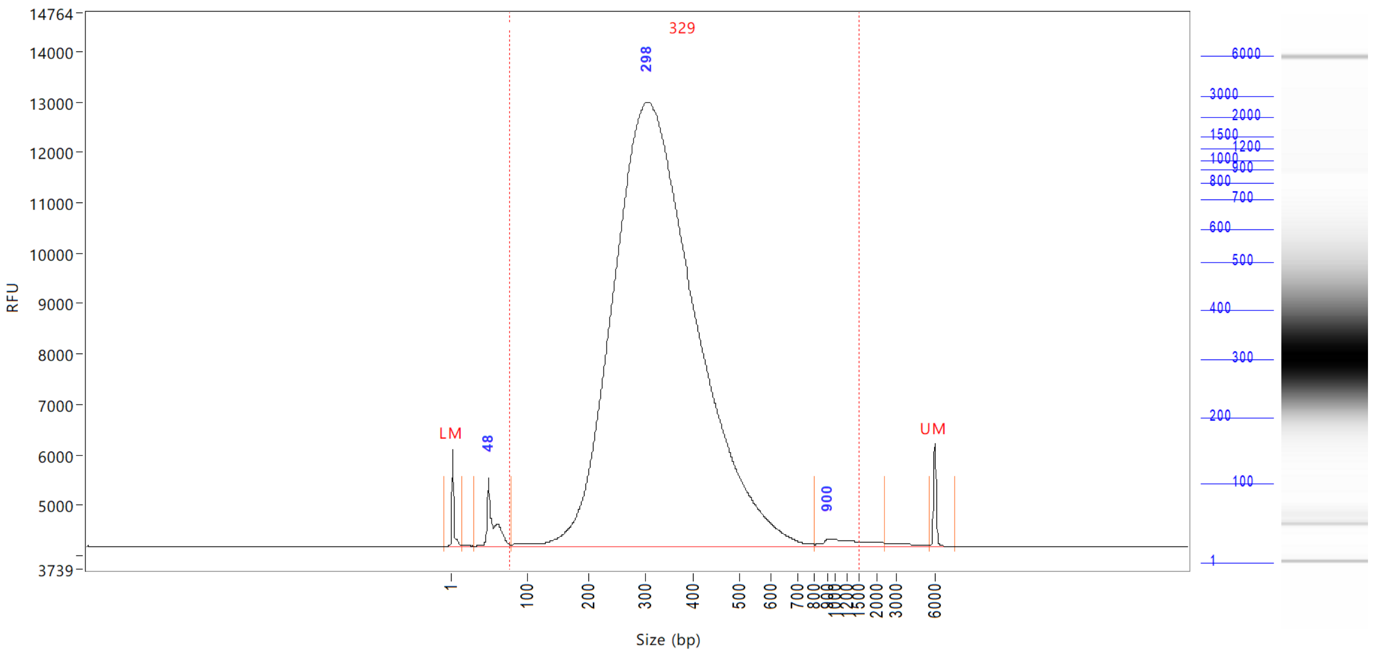

Peak	Size (bp)	Conc. (ng/uL)	From (bp)	To (bp)	RFU
1	1 (LM)	0.0124	0	15	1916
2	48	0.2998	31	78	1352
3	298	18.5979	78	804	8814
4	900	0.1142	804	2417	160
5	6000 (UM)	0.0082	5593	7617	2052

TIC:	19.0119	ng/uL
TIM:	102.9239	nmole/L
Total Conc.:	19.0488	ng/uL

Smear Analysis 75 bp to 1500 bp 18.6787 ng/ul 98.1 %Total 93.4157 nmole/L 329 Avg. Size (b.p.) 29.82 %CV

Sample Peak Width (sec): 50 Sample Min Peak Height: 25 Sample Baseline V to V?: Y Sample Baseline V to V pts: 3
Sample Filter: Binomial # of Pts for Filter: 3 Sample Start Region (min): 0 Sample End Region (min): 50
Manual Baseline Start (min): 10 Manual Baseline End (min): 48
Marker Peak Width (sec): 5 Marker Min Peak Height: 200 Marker Baseline V to V?: Y Marker Baseline V to V pts: 3
Lower Marker Selection: First Peak > 200 RFU Upper Marker Selection: Last Peak > 200 RFU
Ladder Size (bp): 1, 100, 200, 300, 400, 500, 600, 700, 800, 900, 1000, 1200, 1500, 2000, 3000, 6000
Quantification Using: Ladder Final Concentration (ng/uL): 0.0830 Dilution Factor: 12.0

Copyright 2015 Advanced Analytical Technologies, Inc.

Sample: 103613-001-080
Well Location: H10
Created: Friday, June 21, 2019 2:11:36 PM

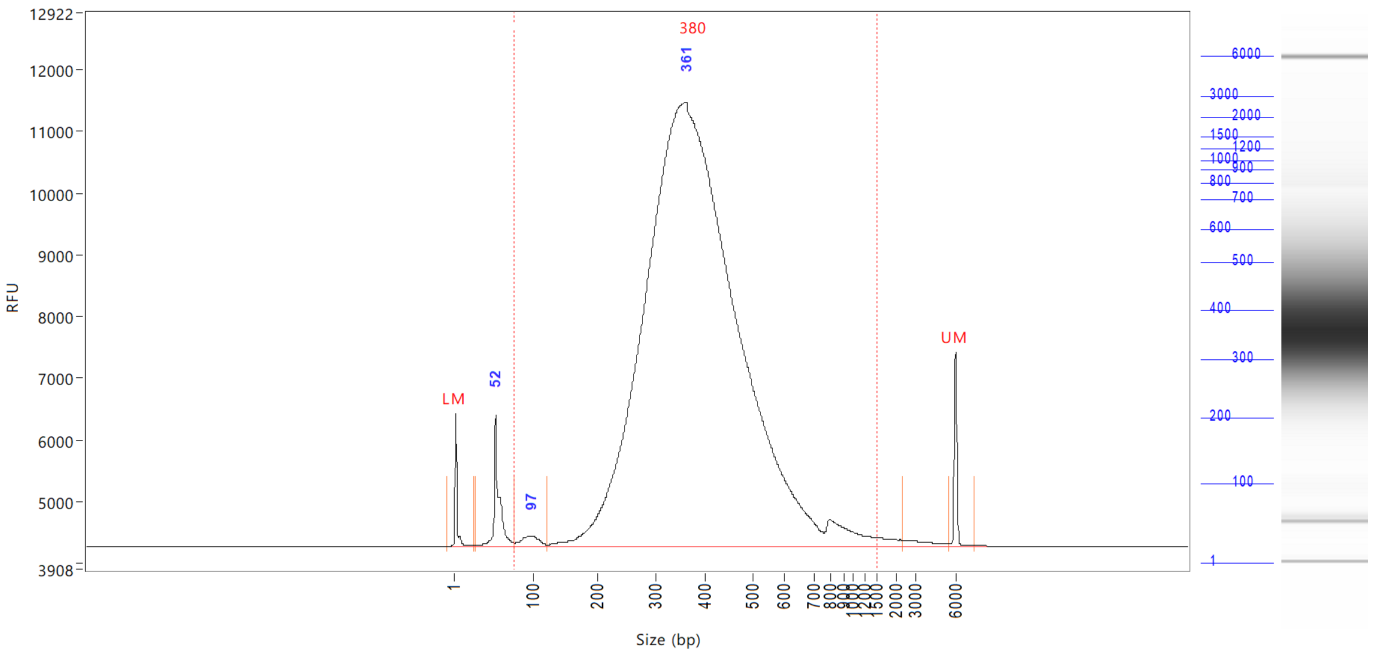

Peak	Size (bp)	Conc. (ng/uL)	From (bp)	To (bp)	RFU
1	1 (LM)	0.0124	0	26	2151
2	52	0.3105	27	76	2138
3	97	0.0865	76	122	164
4	361	14.0777	122	2384	7207
5	6000 (UM)	0.0108	5517	7415	3157

TIC:	14.4747	ng/uL
TIM:	70.9032	nmole/L
Total Conc.:	14.5166	ng/uL

Smear Analysis	75 bp to 1500 bp	14.1203 ng/ul	97.3 %Total	61.1409 nmole/L	380 Avg. Size (b.p.)	31.60 %CV

Sample Peak Width (sec): 50 Sample Min Peak Height: 25 Sample Baseline V to V?: Y Sample Baseline V to V pts: 3
Sample Filter: Binomial # of Pts for Filter: 3 Sample Start Region (min): 0 Sample End Region (min): 50
Manual Baseline Start (min): 10 Manual Baseline End (min): 48
Marker Peak Width (sec): 5 Marker Min Peak Height: 200 Marker Baseline V to V?: Y Marker Baseline V to V pts: 3
Lower Marker Selection: First Peak > 200 RFU Upper Marker Selection: Last Peak > 200 RFU
Ladder Size (bp): 1, 100, 200, 300, 400, 500, 600, 700, 800, 900, 1000, 1200, 1500, 2000, 3000, 6000
Quantification Using: Ladder Final Concentration (ng/uL): 0.0830 Dilution Factor: 12.0

Sample: 103613-001-081
Well Location: A11
Created: Friday, June 21, 2019 2:11:36 PM

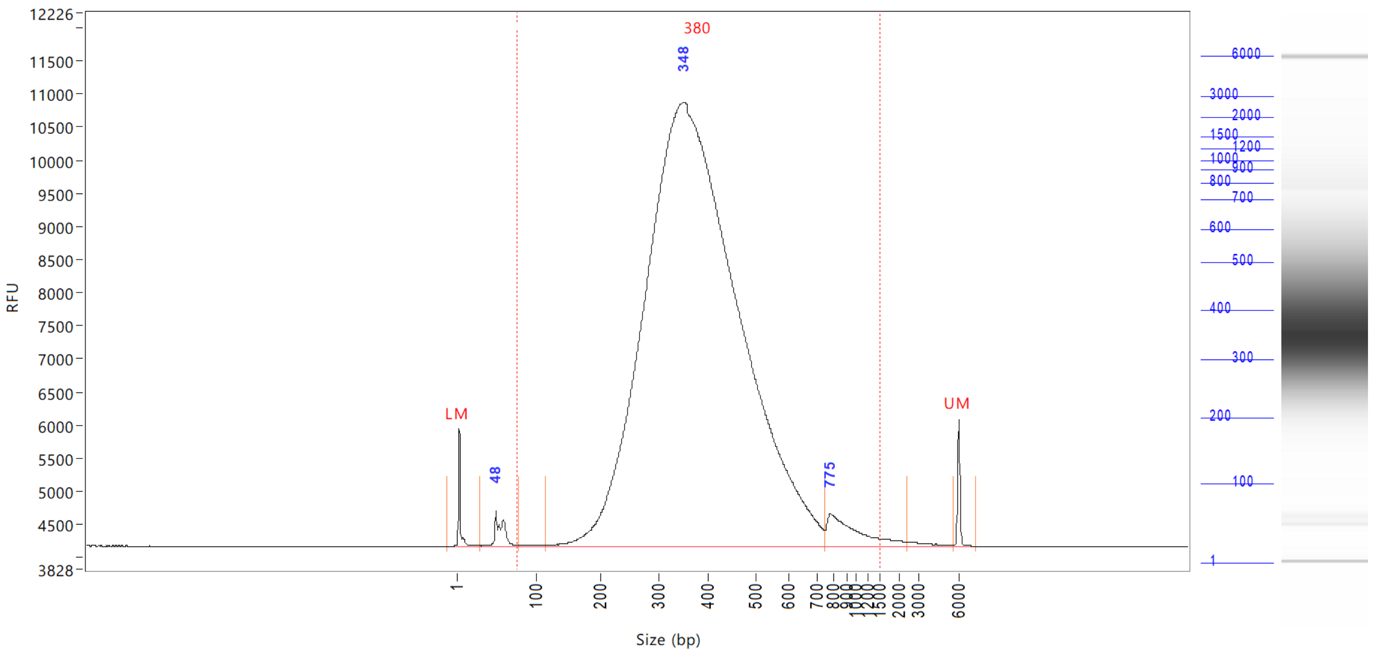

Peak	Size (bp)	Conc. (ng/uL)	From (bp)	To (bp)	RFU
1	1 (LM)	0.0124	0	29	1770
2	48	0.1562	29	78	543
3	348	16.1599	114	744	6712
4	775	0.3095	744	2384	495
5	6000 (UM)	0.0077	5644	7238	1906

TIC:	16.6256	ng/uL
TIM:	76.8851	nmole/L
Total Conc.:	16.6694	ng/uL

Smear Analysis	75 bp to 1500 bp	16.4410 ng/ul	98.6 %Total	71.1799 nmole/L	380 Avg. Size (b.p.)	31.62 %CV

```
Sample Peak Width (sec): 50   Sample Min Peak Height: 25    Sample Baseline V to V?: Y    Sample Baseline V to V pts: 3
Sample Filter: Binomial       # of Pts for Filter: 3        Sample Start Region (min): 0  Sample End Region (min): 50
Manual Baseline Start (min): 10     Manual Baseline End (min): 48
Marker Peak Width (sec): 5    Marker Min Peak Height: 200   Marker Baseline V to V?: Y    Marker Baseline V to V pts: 3
Lower Marker Selection: First Peak > 200 RFU                Upper Marker Selection: Last Peak > 200 RFU
Ladder Size (bp): 1, 100, 200, 300, 400, 500, 600, 700, 800, 900, 1000, 1200, 1500, 2000, 3000, 6000
Quantification Using: Ladder          Final Concentration (ng/uL): 0.0830          Dilution Factor: 12.0
```


Copyright 2015 Advanced Analytical Technologies, Inc.

Sample: 103613-001-082
Well Location: B11
Created: Friday, June 21, 2019 2:11:36 PM

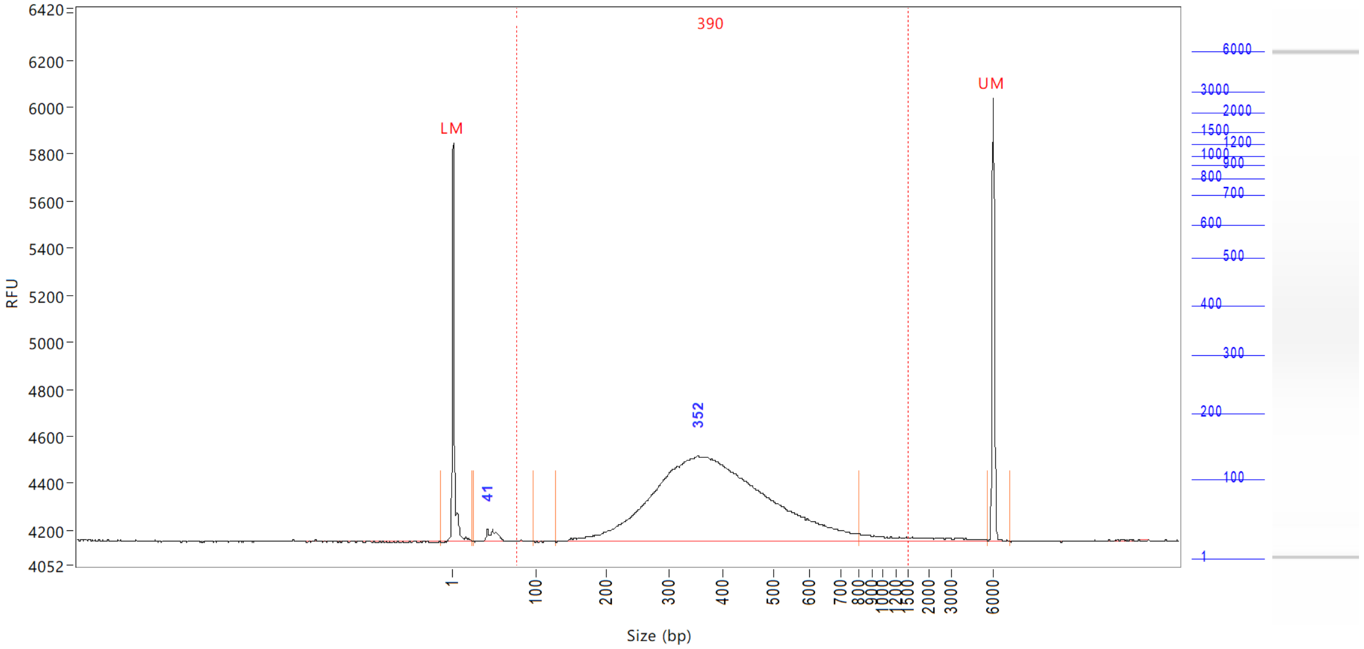

Peak	Size (bp)	Conc. (ng/uL)	From (bp)	To (bp)	RFU
1	1 (LM)	0.0124	0	24	1696
2	41	0.0180	26	96	53
3	352	1.0708	127	804	363
4	6000 (UM)	0.0076	5593	7212	1886

TIC:	1.0888	ng/uL
TIM:	5.2272	nmole/L
Total Conc.:	1.1155	ng/uL

Smear Analysis 75 bp to 1500 bp 1.0862 ng/ul 97.4 %Total 4.5777 nmole/L 390 Avg. Size (b.p.) 34.20 %CV

Sample Peak Width (sec): 50 Sample Min Peak Height: 25 Sample Baseline V to V?: Y Sample Baseline V to V pts: 3
Sample Filter: Binomial # of Pts for Filter: 3 Sample Start Region (min): 0 Sample End Region (min): 50
Manual Baseline Start (min): 10 Manual Baseline End (min): 48
Marker Peak Width (sec): 5 Marker Min Peak Height: 200 Marker Baseline V to V?: Y Marker Baseline V to V pts: 3
Lower Marker Selection: First Peak > 200 RFU Upper Marker Selection: Last Peak > 200 RFU
Ladder Size (bp): 1, 100, 200, 300, 400, 500, 600, 700, 800, 900, 1000, 1200, 1500, 2000, 3000, 6000
Quantification Using: Ladder Final Concentration (ng/uL): 0.0830 Dilution Factor: 12.0

 Copyright 2015 Advanced Analytical Technologies, Inc.

Sample: 103613-001-083
Well Location: C11
Created: Friday, June 21, 2019 2:11:36 PM

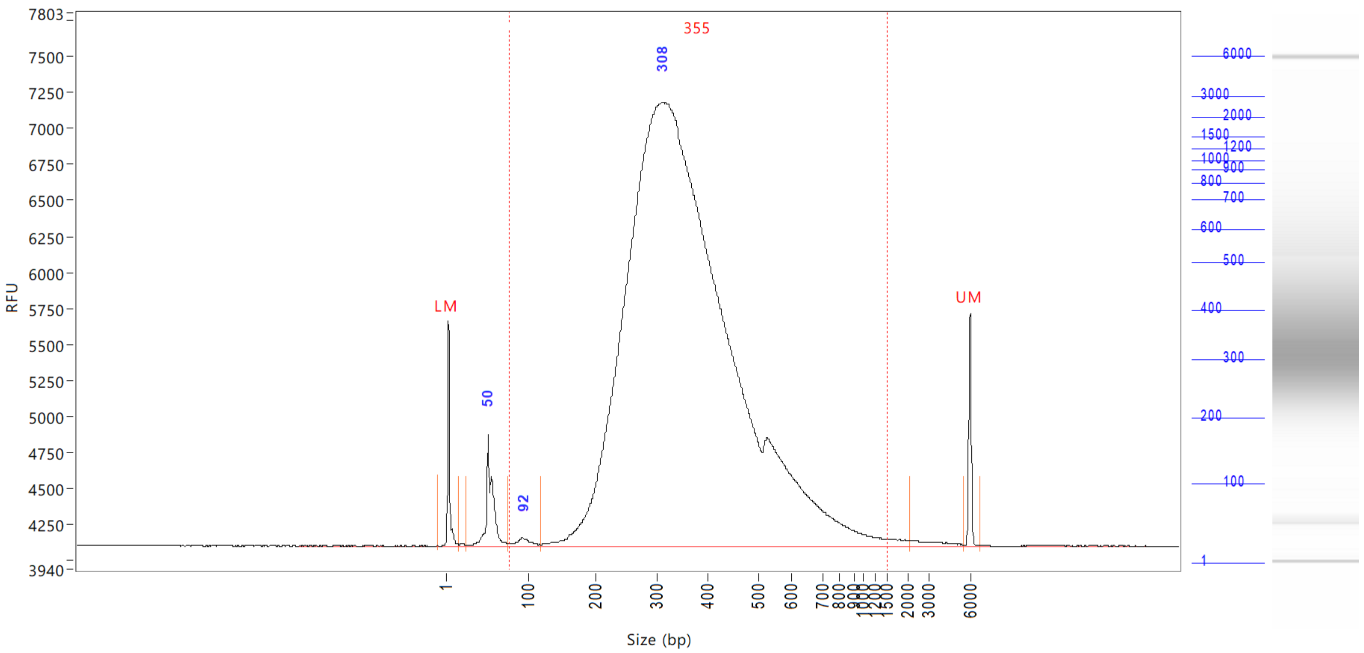

Peak	Size (bp)	Conc. (ng/uL)	From (bp)	To (bp)	RFU
1	1 (LM)	0.0124	0	15	1566
2	50	0.2050	24	75	774
3	92	0.0324	75	117	55
4	308	9.3691	117	2084	3086
5	6000 (UM)	0.0078	5517	6783	1618

TIC:	9.6066	ng/uL
TIM:	50.0865	nmole/L
Total Conc.:	9.6361	ng/uL

Smear Analysis 75 bp to 1500 bp 9.3826 ng/ul 97.4 %Total 43.5352 nmole/L 355 Avg. Size (b.p.) 34.34 %CV

Sample Peak Width (sec): 50 Sample Min Peak Height: 25 Sample Baseline V to V?: Y Sample Baseline V to V pts: 3
Sample Filter: Binomial # of Pts for Filter: 3 Sample Start Region (min): 0 Sample End Region (min): 50
Manual Baseline Start (min): 10 Manual Baseline End (min): 48
Marker Peak Width (sec): 5 Marker Min Peak Height: 200 Marker Baseline V to V?: Y Marker Baseline V to V pts: 3
Lower Marker Selection: First Peak > 200 RFU Upper Marker Selection: Last Peak > 200 RFU
Ladder Size (bp): 1, 100, 200, 300, 400, 500, 600, 700, 800, 900, 1000, 1200, 1500, 2000, 3000, 6000
Quantification Using: Ladder Final Concentration (ng/uL): 0.0830 Dilution Factor: 12.0

 Copyright 2015 Advanced Analytical Technologies, Inc.

Sample: 103613-001-084
Well Location: D11
Created: Friday, June 21, 2019 2:11:36 PM

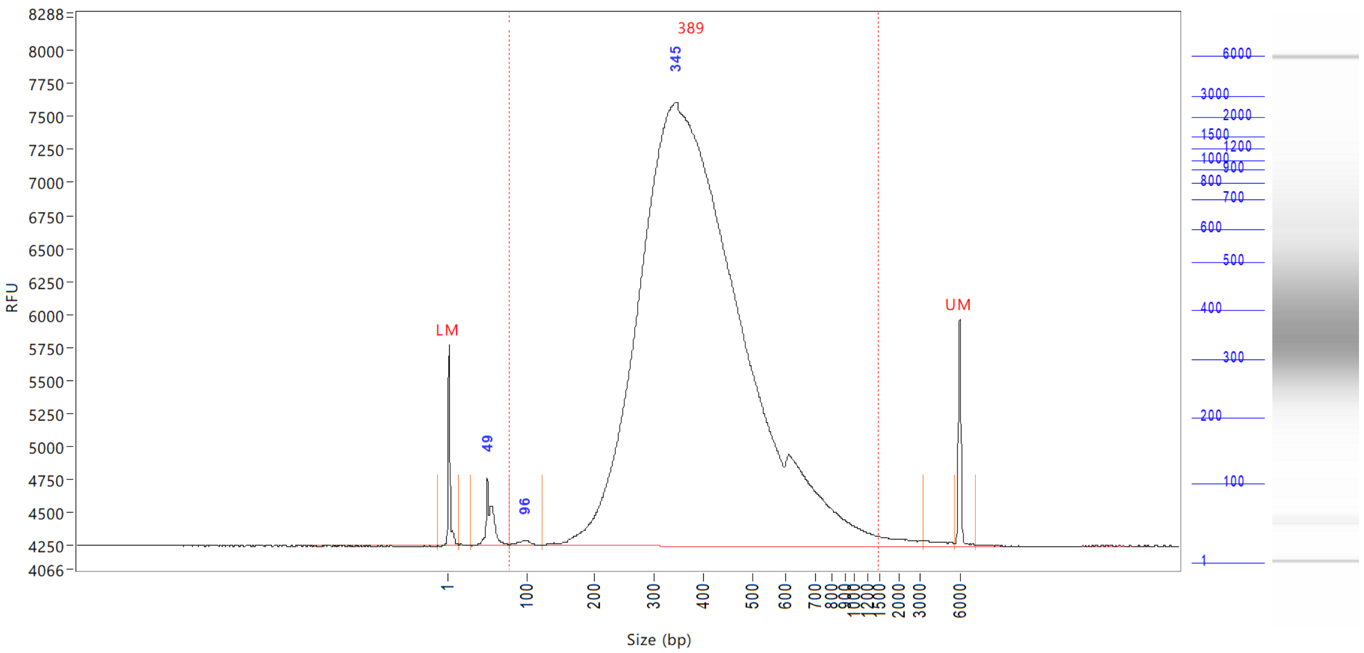

Peak	Size (bp)	Conc. (ng/uL)	From (bp)	To (bp)	RFU
1	1 (LM)	0.0124	0	15	1523
2	49	0.1410	30	76	513
3	96	0.0249	76	122	39
4	345	10.3934	122	3255	3370
5	6000 (UM)	0.0084	5593	7111	1725

TIC:	10.5594	ng/uL
TIM:	47.8682	nmole/L
Total Conc.:	10.5799	ng/uL

Smear Analysis 75 bp to 1500 bp 10.3761 ng/ul 98.1 %Total 43.8325 nmole/L 389 Avg. Size (b.p.) 33.25 %CV

Sample Peak Width (sec): 50 Sample Min Peak Height: 25 Sample Baseline V to V?: Y Sample Baseline V to V pts: 3
Sample Filter: Binomial # of Pts for Filter: 3 Sample Start Region (min): 0 Sample End Region (min): 50
Manual Baseline Start (min): 10 Manual Baseline End (min): 48
Marker Peak Width (sec): 5 Marker Min Peak Height: 200 Marker Baseline V to V?: Y Marker Baseline V to V pts: 3
Lower Marker Selection: First Peak > 200 RFU Upper Marker Selection: Last Peak > 200 RFU
Ladder Size (bp): 1, 100, 200, 300, 400, 500, 600, 700, 800, 900, 1000, 1200, 1500, 2000, 3000, 6000
Quantification Using: Ladder Final Concentration (ng/uL): 0.0830 Dilution Factor: 12.0

Copyright 2015 Advanced Analytical Technologies, Inc.

Sample: 103613-001-085
Well Location: E11
Created: Friday, June 21, 2019 2:11:36 PM

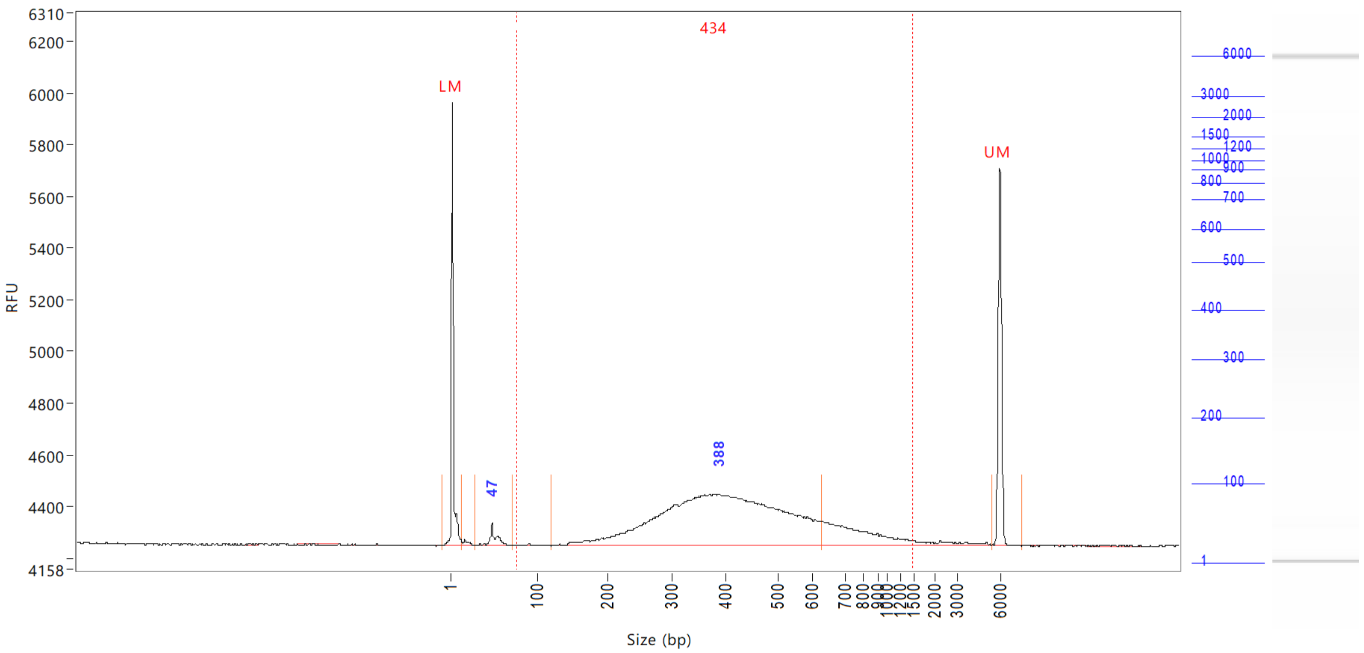

Peak	Size (bp)	Conc. (ng/uL)	From (bp)	To (bp)	RFU
1	1 (LM)	0.0124	0	14	1713
2	47	0.0165	28	72	84
3	388	0.6644	121	630	197
4	6000 (UM)	0.0076	5441	7465	1457

TIC:	0.6809	ng/uL
TIM:	3.3774	nmole/L
Total Conc.:	0.7837	ng/uL

Smear Analysis 75 bp to 1500 bp 0.7500 ng/ul 95.7 %Total 2.8417 nmole/L 434 Avg. Size (b.p.) 40.70 %CV

Sample Peak Width (sec): 50 Sample Min Peak Height: 25 Sample Baseline V to V?: Y Sample Baseline V to V pts: 3
Sample Filter: Binomial # of Pts for Filter: 3 Sample Start Region (min): 0 Sample End Region (min): 50
Manual Baseline Start (min): 10 Manual Baseline End (min): 48
Marker Peak Width (sec): 5 Marker Min Peak Height: 200 Marker Baseline V to V?: Y Marker Baseline V to V pts: 3
Lower Marker Selection: First Peak > 200 RFU Upper Marker Selection: Last Peak > 200 RFU
Ladder Size (bp): 1, 100, 200, 300, 400, 500, 600, 700, 800, 900, 1000, 1200, 1500, 2000, 3000, 6000
Quantification Using: Ladder Final Concentration (ng/uL): 0.0830 Dilution Factor: 12.0

 Copyright 2015 Advanced Analytical Technologies, Inc.

Sample: 103613-001-086
Well Location: F11
Created: Friday, June 21, 2019 2:11:36 PM

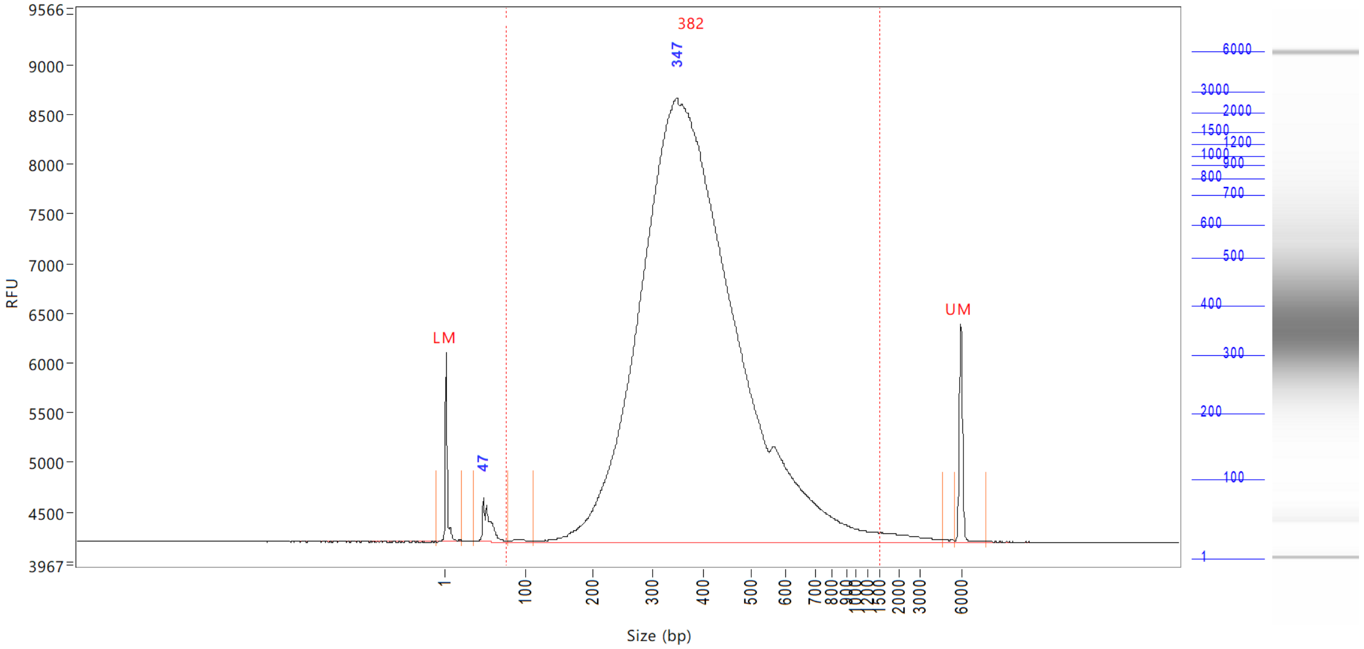

Peak	Size (bp)	Conc. (ng/uL)	From (bp)	To (bp)	RFU
1	1 (LM)	0.0124	0	21	1910
2	47	0.1105	35	78	445
3	347	10.0243	112	4628	4472
4	6000 (UM)	0.0101	5543	7794	2198

TIC:	10.1348	ng/uL
TIM:	45.2761	nmole/L
Total Conc.:	10.1514	ng/uL

Smear Analysis	75 bp to 1500 bp	9.9780 ng/ul	98.3 %Total	42.9242 nmole/L	382 Avg. Size (b.p.)	31.68 %CV

Sample Peak Width (sec): 50   Sample Min Peak Height: 25   Sample Baseline V to V?: Y   Sample Baseline V to V pts: 3
Sample Filter: Binomial   # of Pts for Filter: 3   Sample Start Region (min): 0   Sample End Region (min): 50
Manual Baseline Start (min): 10   Manual Baseline End (min): 48
Marker Peak Width (sec): 5   Marker Min Peak Height: 200   Marker Baseline V to V?: Y   Marker Baseline V to V pts: 3
Lower Marker Selection: First Peak > 200 RFU   Upper Marker Selection: Last Peak > 200 RFU
Ladder Size (bp): 1, 100, 200, 300, 400, 500, 600, 700, 800, 900, 1000, 1200, 1500, 2000, 3000, 6000
Quantification Using: Ladder   Final Concentration (ng/uL): 0.0830   Dilution Factor: 12.0

Copyright 2015 Advanced Analytical Technologies, Inc.

Sample: 103613-001-087
Well Location: G11
Created: Friday, June 21, 2019 2:11:36 PM

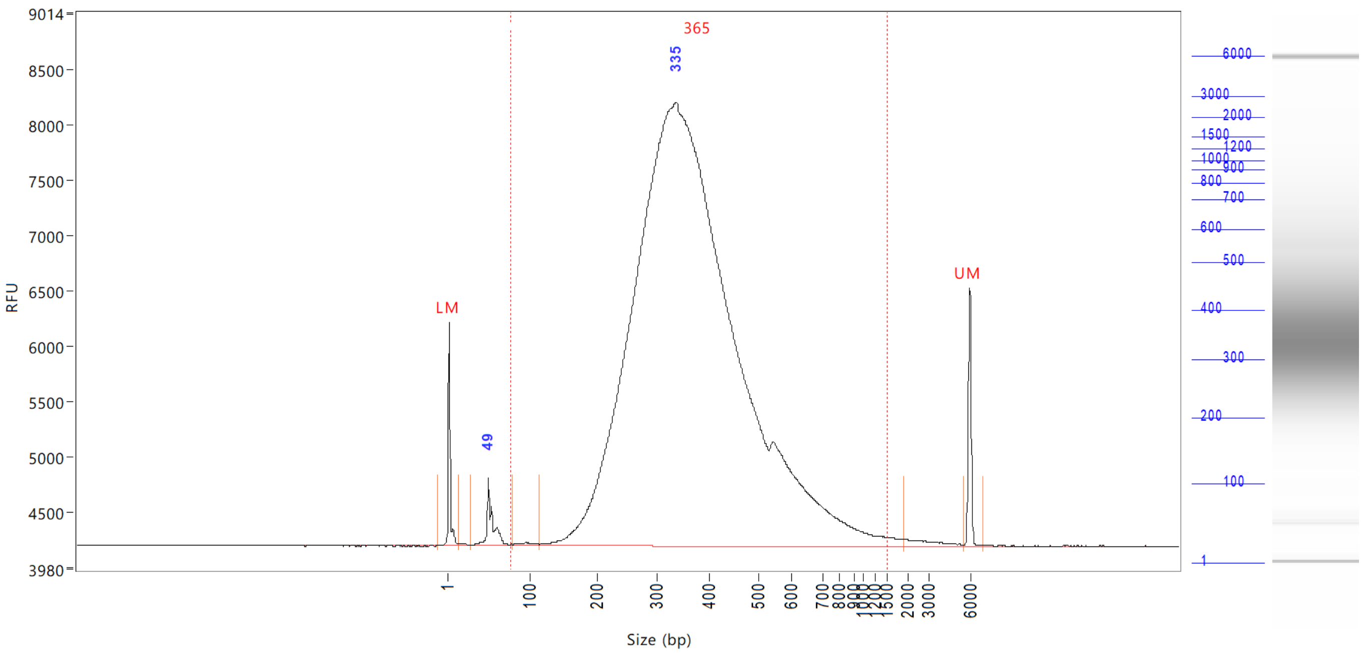

Peak	Size (bp)	Conc. (ng/uL)	From (bp)	To (bp)	RFU
1	1 (LM)	0.0124	0	15	2027
2	49	0.1130	28	78	615
3	335	9.3054	114	1911	4019
4	6000 (UM)	0.0097	5517	7010	2335
	TIC:	9.4184	ng/uL		
	TIM:	45.0938	nmole/L		
	Total Conc.:	9.4671	ng/uL		

Smear Analysis	75 bp to 1500 bp	9.3034 ng/ul	98.3 %Total	41.9077 nmole/L	365 Avg. Size (b.p.)	34.06 %CV

Sample Peak Width (sec): 50 Sample Min Peak Height: 25 Sample Baseline V to V?: Y Sample Baseline V to V pts: 3
Sample Filter: Binomial # of Pts for Filter: 3 Sample Start Region (min): 0 Sample End Region (min): 50
Manual Baseline Start (min): 10 Manual Baseline End (min): 48
Marker Peak Width (sec): 5 Marker Min Peak Height: 200 Marker Baseline V to V?: Y Marker Baseline V to V pts: 3
Lower Marker Selection: First Peak > 200 RFU Upper Marker Selection: Last Peak > 200 RFU
Ladder Size (bp): 1, 100, 200, 300, 400, 500, 600, 700, 800, 900, 1000, 1200, 1500, 2000, 3000, 6000
Quantification Using: Ladder Final Concentration (ng/uL): 0.0830 Dilution Factor: 12.0

Copyright 2015 Advanced Analytical Technologies, Inc.

Sample: 103613-001-088
Well Location: H11
Created: Friday, June 21, 2019 2:11:36 PM

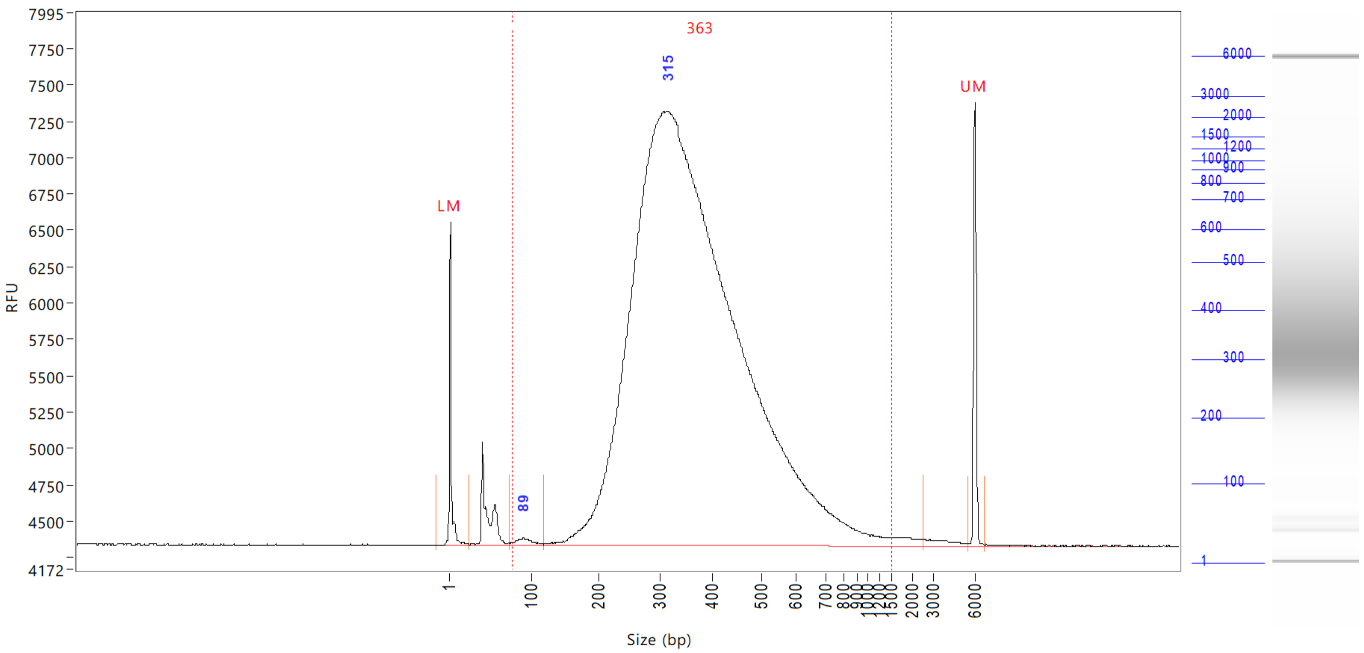

Peak	Size (bp)	Conc. (ng/uL)	From (bp)	To (bp)	RFU
1	1 (LM)	0.0124	0	26	2220
2	89	0.0225	73	118	48
3	315	6.3337	118	2567	2984
4	6000 (UM)	0.0097	5517	6707	3054

TIC:	6.3561	ng/uL
TIM:	28.6441	nmole/L
Total Conc.:	6.5036	ng/uL

Smear Analysis	75 bp to 1500 bp	6.3328 ng/ul	97.4 %Total	28.7321 nmole/L	363 Avg. Size (b.p.)	33.82 %CV

Sample Peak Width (sec): 50 Sample Min Peak Height: 25 Sample Baseline V to V?: Y Sample Baseline V to V pts: 3
Sample Filter: Binomial # of Pts for Filter: 3 Sample Start Region (min): 0 Sample End Region (min): 50
Manual Baseline Start (min): 10 Manual Baseline End (min): 48
Marker Peak Width (sec): 5 Marker Min Peak Height: 200 Marker Baseline V to V?: Y Marker Baseline V to V pts: 3
Lower Marker Selection: First Peak > 200 RFU Upper Marker Selection: Last Peak > 200 RFU
Ladder Size (bp): 1, 100, 200, 300, 400, 500, 600, 700, 800, 900, 1000, 1200, 1500, 2000, 3000, 6000
Quantification Using: Ladder Final Concentration (ng/uL): 0.0830 Dilution Factor: 12.0

Copyright 2015 Advanced Analytical Technologies, Inc.

Sample: 103613-001-089
Well Location: A12
Created: Friday, June 21, 2019 2:11:36 PM

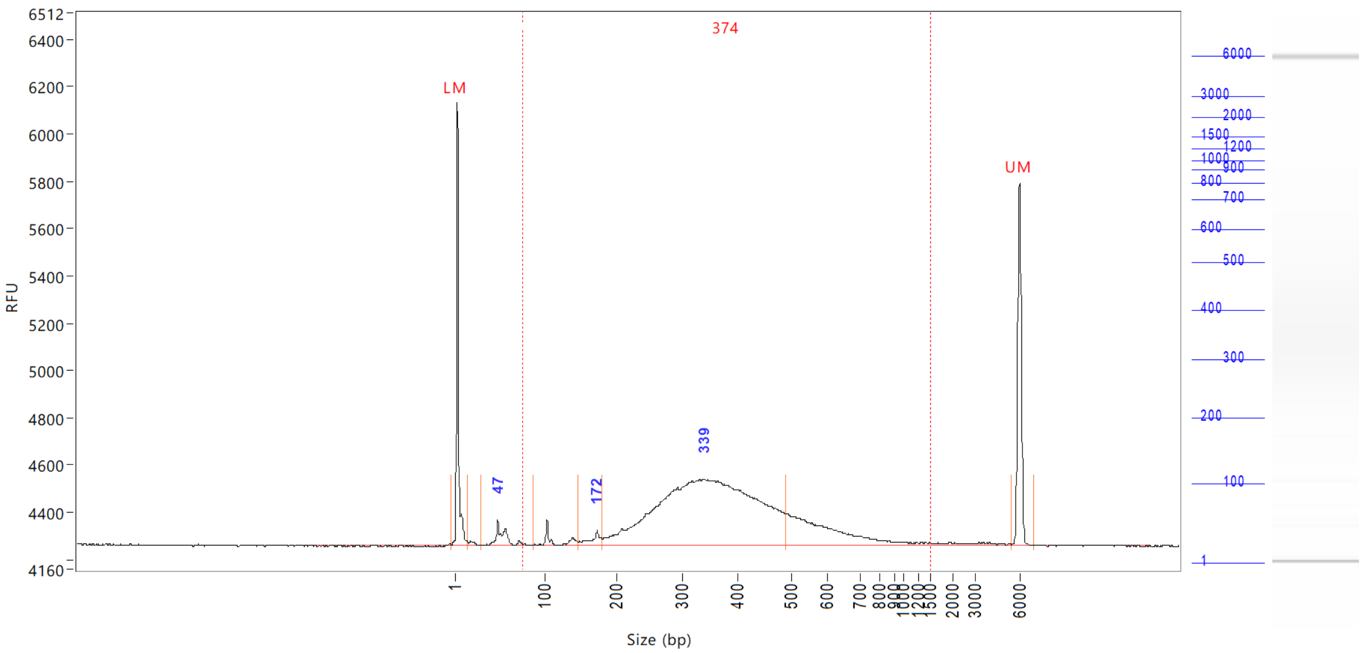

Peak	Size (bp)	Conc. (ng/uL)	From (bp)	To (bp)	RFU
1	1 (LM)	0.0124	0	14	1875
2	47	0.0309	28	87	107
3	172	0.0173	145	179	66
4	339	0.7201	179	489	279
5	6000 (UM)	0.0078	5441	6909	1532

TIC:	0.7683	ng/uL
TIM:	4.6109	nmole/L
Total Conc.:	0.9348	ng/uL

Smear Analysis	75 bp to 1500 bp	0.8894 ng/ul	95.1 %Total	3.9164 nmole/L	374 Avg. Size (b.p.)	38.49 %CV

Sample Peak Width (sec): 50 Sample Min Peak Height: 25 Sample Baseline V to V?: Y Sample Baseline V to V pts: 3
Sample Filter: Binomial # of Pts for Filter: 3 Sample Start Region (min): 0 Sample End Region (min): 50
Manual Baseline Start (min): 10 Manual Baseline End (min): 48
Marker Peak Width (sec): 5 Marker Min Peak Height: 200 Marker Baseline V to V?: Y Marker Baseline V to V pts: 3
Lower Marker Selection: First Peak > 200 RFU Upper Marker Selection: Last Peak > 200 RFU
Ladder Size (bp): 1, 100, 200, 300, 400, 500, 600, 700, 800, 900, 1000, 1200, 1500, 2000, 3000, 6000
Quantification Using: Ladder Final Concentration (ng/uL): 0.0830 Dilution Factor: 12.0

 Copyright 2015 Advanced Analytical Technologies, Inc.

Sample: 103613-001-090
Well Location: B12
Created: Friday, June 21, 2019 2:11:36 PM

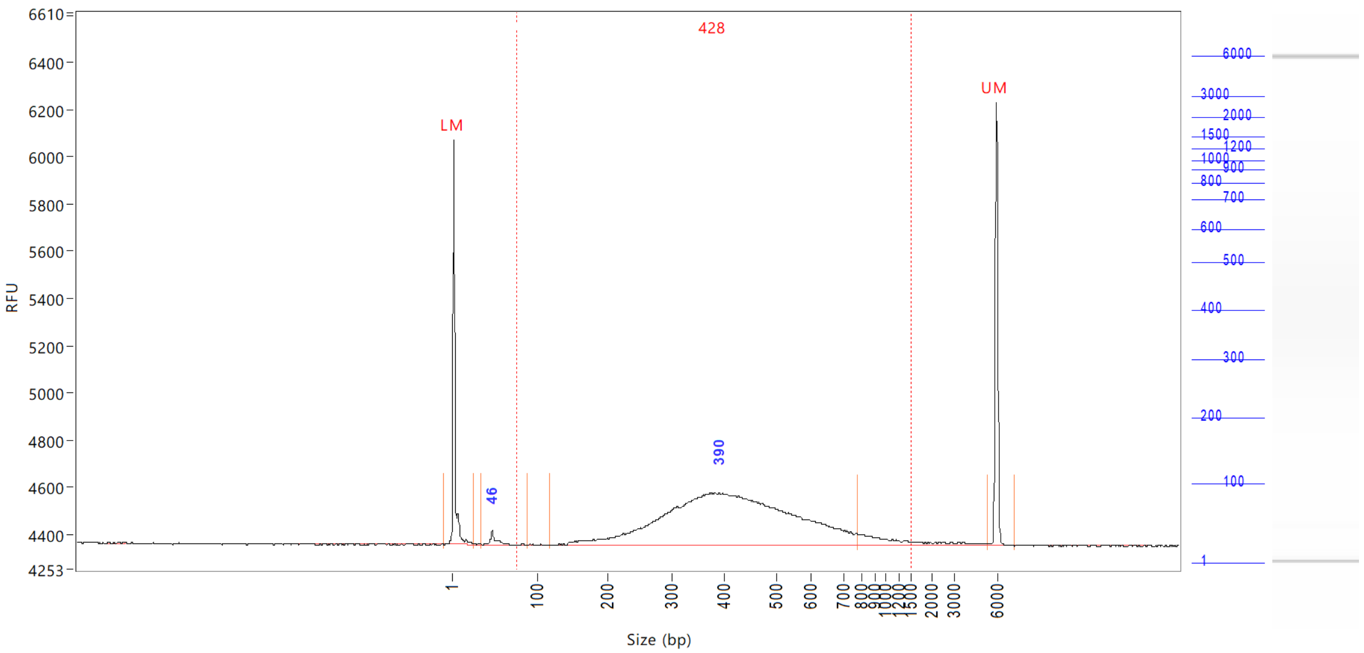

Peak	Size (bp)	Conc. (ng/uL)	From (bp)	To (bp)	RFU
1	1 (LM)	0.0124	0	25	1712
2	46	0.0118	35	88	62
3	390	0.7247	118	784	221
4	6000 (UM)	0.0077	5415	7288	1879

TIC:	0.7366	ng/uL
TIM:	3.3034	nmole/L
Total Conc.:	0.7742	ng/uL

Smear Analysis	75 bp to 1500 bp	0.7505 ng/ul	96.9 %Total	2.8830 nmole/L	428 Avg. Size (b.p.)	37.92 %CV

Sample Peak Width (sec): 50 Sample Min Peak Height: 25 Sample Baseline V to V?: Y Sample Baseline V to V pts: 3
Sample Filter: Binomial # of Pts for Filter: 3 Sample Start Region (min): 0 Sample End Region (min): 50
Manual Baseline Start (min): 10 Manual Baseline End (min): 48
Marker Peak Width (sec): 5 Marker Min Peak Height: 200 Marker Baseline V to V?: Y Marker Baseline V to V pts: 3
Lower Marker Selection: First Peak > 200 RFU Upper Marker Selection: Last Peak > 200 RFU
Ladder Size (bp): 1, 100, 200, 300, 400, 500, 600, 700, 800, 900, 1000, 1200, 1500, 2000, 3000, 6000
Quantification Using: Ladder Final Concentration (ng/uL): 0.0830 Dilution Factor: 12.0

 Copyright 2015 Advanced Analytical Technologies, Inc.

Sample: 103613-001-091
Well Location: C12
Created: Friday, June 21, 2019 2:11:36 PM

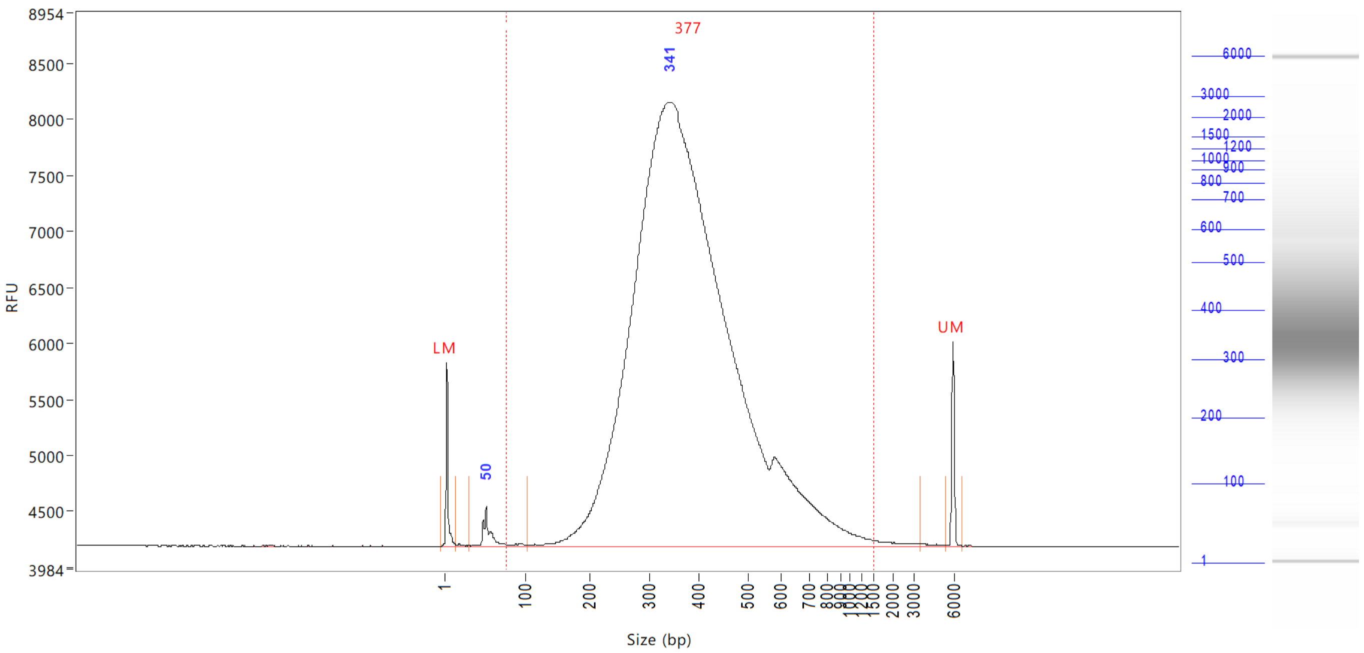

Peak	Size (bp)	Conc. (ng/uL)	From (bp)	To (bp)	RFU
1	1 (LM)	0.0124	0	14	1646
2	50	0.1003	31	104	354
3	341	10.8618	104	3560	3971
4	6000 (UM)	0.0080	5415	6707	1833

TIC:	10.9622	ng/uL
TIM:	49.7887	nmole/L
Total Conc.:	10.9733	ng/uL

Smear Analysis 75 bp to 1500 bp 10.8462 ng/ul 98.8 %Total 47.3841 nmole/L 377 Avg. Size (b.p.) 32.41 %CV

Sample Peak Width (sec): 50 Sample Min Peak Height: 25 Sample Baseline V to V?: Y Sample Baseline V to V pts: 3
Sample Filter: Binomial # of Pts for Filter: 3 Sample Start Region (min): 0 Sample End Region (min): 50
Manual Baseline Start (min): 10 Manual Baseline End (min): 48
Marker Peak Width (sec): 5 Marker Min Peak Height: 200 Marker Baseline V to V?: Y Marker Baseline V to V pts: 3
Lower Marker Selection: First Peak > 200 RFU Upper Marker Selection: Last Peak > 200 RFU
Ladder Size (bp): 1, 100, 200, 300, 400, 500, 600, 700, 800, 900, 1000, 1200, 1500, 2000, 3000, 6000
Quantification Using: Ladder Final Concentration (ng/uL): 0.0830 Dilution Factor: 12.0

 Copyright 2015 Advanced Analytical Technologies, Inc.

Sample: 103613-001-092
Well Location: D12
Created: Friday, June 21, 2019 2:11:36 PM

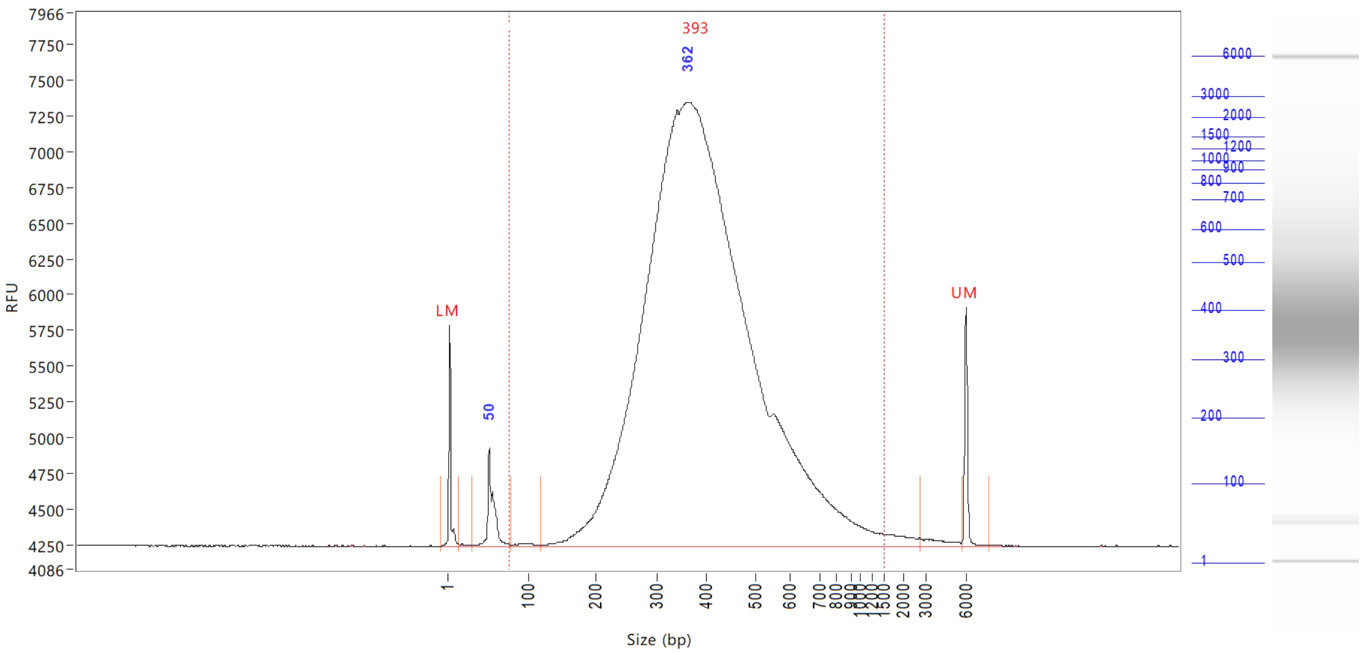

Peak	Size (bp)	Conc. (ng/uL)	From (bp)	To (bp)	RFU
1	1 (LM)	0.0124	0	15	1542
2	50	0.1677	30	78	688
3	362	9.4379	118	2784	3101
4	6000 (UM)	0.0084	5670	7692	1668

TIC:	9.6056	ng/uL
TIM:	43.9066	nmole/L
Total Conc.:	9.6544	ng/uL

Smear Analysis 75 bp to 1500 bp 9.4058 ng/ul 97.4 %Total 39.3889 nmole/L 393 Avg. Size (b.p.) 33.61 %CV

Sample Peak Width (sec): 50 Sample Min Peak Height: 25 Sample Baseline V to V?: Y Sample Baseline V to V pts: 3
Sample Filter: Binomial # of Pts for Filter: 3 Sample Start Region (min): 0 Sample End Region (min): 50
Manual Baseline Start (min): 10 Manual Baseline End (min): 48
Marker Peak Width (sec): 5 Marker Min Peak Height: 200 Marker Baseline V to V?: Y Marker Baseline V to V pts: 3
Lower Marker Selection: First Peak > 200 RFU Upper Marker Selection: Last Peak > 200 RFU
Ladder Size (bp): 1, 100, 200, 300, 400, 500, 600, 700, 800, 900, 1000, 1200, 1500, 2000, 3000, 6000
Quantification Using: Ladder Final Concentration (ng/uL): 0.0830 Dilution Factor: 12.0

 Copyright 2015 Advanced Analytical Technologies, Inc.

Sample: 103613-001-093
Well Location: E12
Created: Friday, June 21, 2019 2:11:36 PM

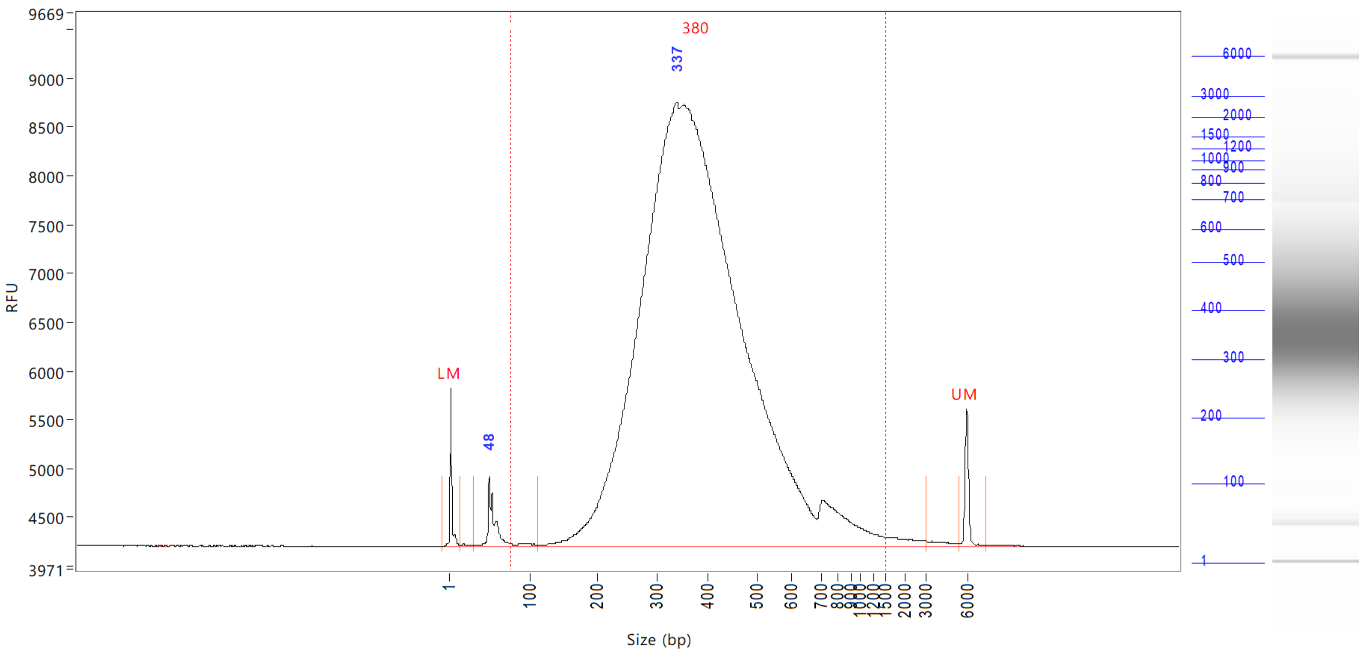

Peak	Size (bp)	Conc. (ng/uL)	From (bp)	To (bp)	RFU
1	1 (LM)	0.0124	0	15	1613
2	48	0.2009	30	111	711
3	337	13.2972	111	3001	4553
4	6000 (UM)	0.0086	5492	7364	1406

TIC:	13.4980	ng/uL
TIM:	62.4081	nmole/L
Total Conc.:	13.5274	ng/uL

Smear Analysis	75 bp to 1500 bp	13.2585 ng/ul	98.0 %Total	57.4537 nmole/L	380 Avg. Size (b.p.)	33.26 %CV

Sample Peak Width (sec): 50 Sample Min Peak Height: 25 Sample Baseline V to V?: Y Sample Baseline V to V pts: 3
Sample Filter: Binomial # of Pts for Filter: 3 Sample Start Region (min): 0 Sample End Region (min): 50
Manual Baseline Start (min): 10 Manual Baseline End (min): 48
Marker Peak Width (sec): 5 Marker Min Peak Height: 200 Marker Baseline V to V?: Y Marker Baseline V to V pts: 3
Lower Marker Selection: First Peak > 200 RFU Upper Marker Selection: Last Peak > 200 RFU
Ladder Size (bp): 1, 100, 200, 300, 400, 500, 600, 700, 800, 900, 1000, 1200, 1500, 2000, 3000, 6000
Quantification Using: Ladder Final Concentration (ng/uL): 0.0830 Dilution Factor: 12.0

Copyright 2015 Advanced Analytical Technologies, Inc.

Sample: 103613-001-094
Well Location: F12
Created: Friday, June 21, 2019 2:11:36 PM

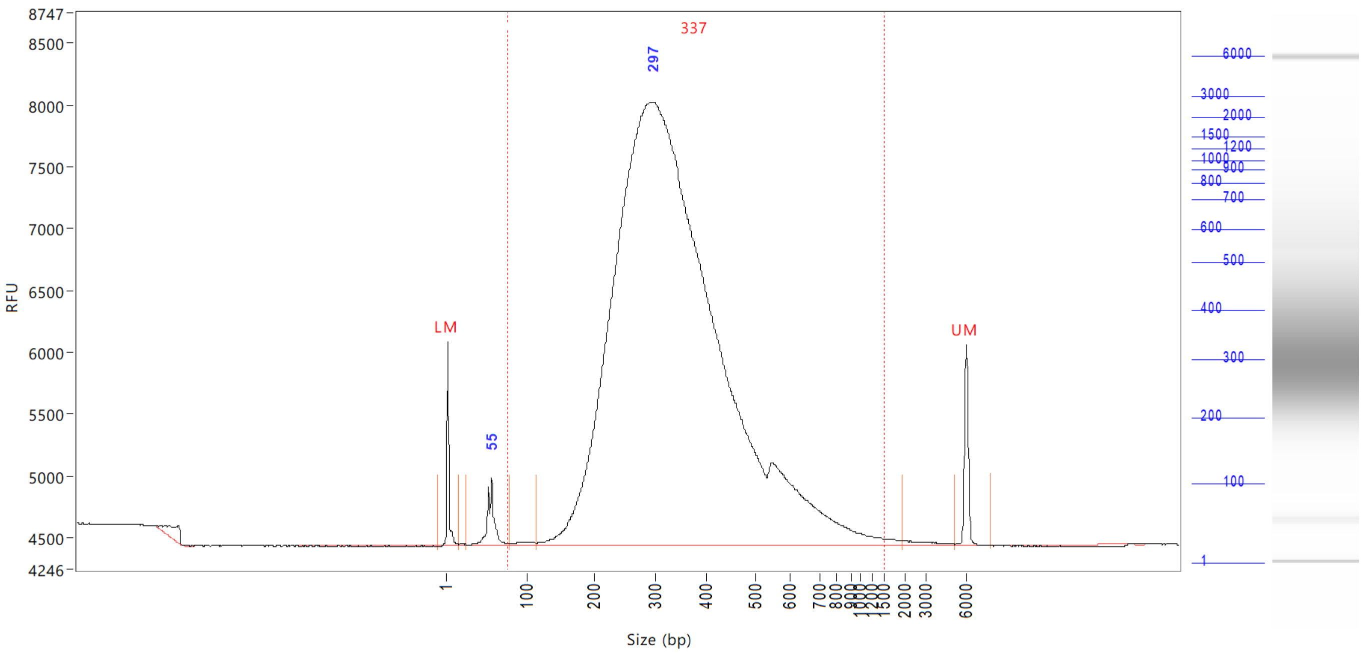

Peak	Size (bp)	Conc. (ng/uL)	From (bp)	To (bp)	RFU
1	1 (LM)	0.0124	0	15	1651
2	55	0.1457	25	78	551
3	297	10.6549	112	1973	3587
4	6000 (UM)	0.0098	5161	7794	1611

TIC:	10.8006	ng/uL
TIM:	56.1272	nmole/L
Total Conc.:	10.8363	ng/uL

Smear Analysis 75 bp to 1500 bp 10.6605 ng/ul 98.4 %Total 52.0620 nmole/L 337 Avg. Size (b.p.) 35.96 %CV

Sample Peak Width (sec): 50 Sample Min Peak Height: 25 Sample Baseline V to V?: Y Sample Baseline V to V pts: 3
Sample Filter: Binomial # of Pts for Filter: 3 Sample Start Region (min): 0 Sample End Region (min): 50
Manual Baseline Start (min): 10 Manual Baseline End (min): 48
Marker Peak Width (sec): 5 Marker Min Peak Height: 200 Marker Baseline V to V?: Y Marker Baseline V to V pts: 3
Lower Marker Selection: First Peak > 200 RFU Upper Marker Selection: Last Peak > 200 RFU
Ladder Size (bp): 1, 100, 200, 300, 400, 500, 600, 700, 800, 900, 1000, 1200, 1500, 2000, 3000, 6000
Quantification Using: Ladder Final Concentration (ng/uL): 0.0830 Dilution Factor: 12.0

Copyright 2015 Advanced Analytical Technologies, Inc.

Sample: 103613-001-095
Well Location: G12
Created: Friday, June 21, 2019 2:11:36 PM

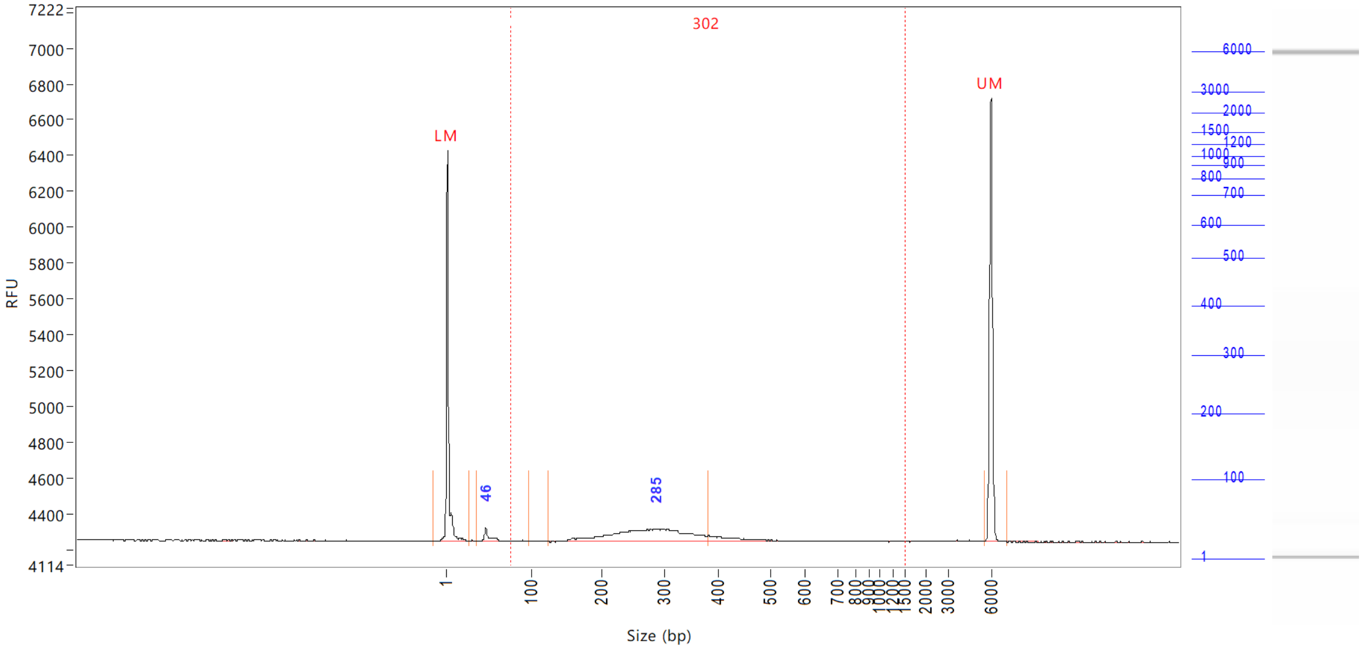

Peak	Size (bp)	Conc. (ng/uL)	From (bp)	To (bp)	RFU
1	1 (LM)	0.0124	0	28	2182
2	46	0.0104	36	97	72
3	285	0.1140	125	380	67
4	6000 (UM)	0.0094	5568	7111	2481

TIC:	0.1244	ng/uL
TIM:	1.0341	nmole/L
Total Conc.:	0.1492	ng/uL

Smear Analysis	75 bp to 1500 bp	0.1347 ng/ul	90.3 %Total	0.7342 nmole/L	302 Avg. Size (b.p.)	36.98 %CV

Sample Peak Width (sec): 50 Sample Min Peak Height: 25 Sample Baseline V to V?: Y Sample Baseline V to V pts: 3
Sample Filter: Binomial # of Pts for Filter: 3 Sample Start Region (min): 0 Sample End Region (min): 50
Manual Baseline Start (min): 10 Manual Baseline End (min): 48
Marker Peak Width (sec): 5 Marker Min Peak Height: 200 Marker Baseline V to V?: Y Marker Baseline V to V pts: 3
Lower Marker Selection: First Peak > 200 RFU Upper Marker Selection: Last Peak > 200 RFU
Ladder Size (bp): 1, 100, 200, 300, 400, 500, 600, 700, 800, 900, 1000, 1200, 1500, 2000, 3000, 6000
Quantification Using: Ladder Final Concentration (ng/uL): 0.0830 Dilution Factor: 12.0

 Copyright 2015 Advanced Analytical Technologies, Inc.

Sample: 103613-001-096
Well Location: H12
Created: Friday, June 21, 2019 2:11:36 PM

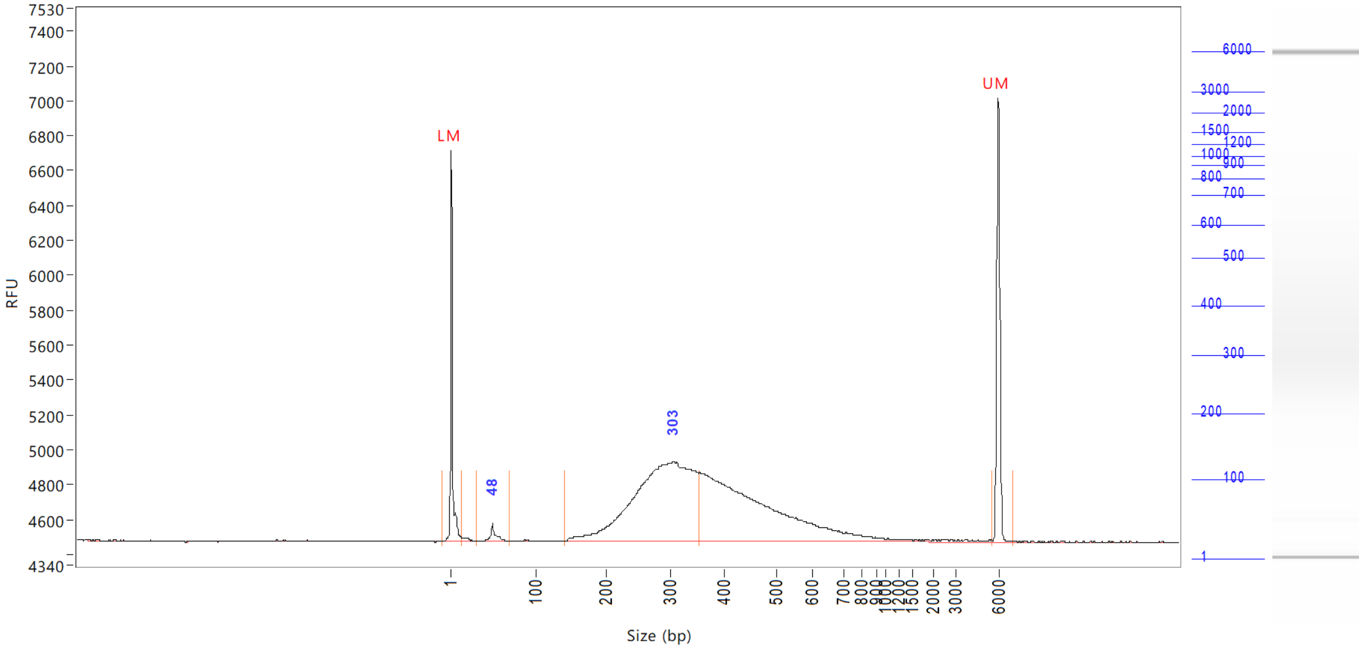

Peak	Size (bp)	Conc. (ng/uL)	From (bp)	To (bp)	RFU
1	1 (LM)	0.0124	0	15	2234
2	48	0.0147	31	70	106
3	303	0.5975	141	354	453
4	6000 (UM)	0.0091	5593	7036	2547

TIC:	0.6123	ng/uL
TIM:	3.9929	nmole/L
Total Conc.:	1.1100	ng/uL

Smear Analysis	75 bp to 1500 bp	1.0832 ng/ul	97.6 %Total	4.8779 nmole/L	365 Avg. Size (b.p.)	36.35 %CV

Sample Peak Width (sec): 10 Sample Min Peak Height: 100 Sample Baseline V to V?: Y Sample Baseline V to V pts: 3
Sample Filter: Binomial # of Pts for Filter: 3 Sample Start Region (min): 0 Sample End Region (min): 50
Manual Baseline Start (min): 10 Manual Baseline End (min): 48
Marker Peak Width (sec): 5 Marker Min Peak Height: 200 Marker Baseline V to V?: Y Marker Baseline V to V pts: 3
Lower Marker Selection: First Peak > 200 RFU Upper Marker Selection: Last Peak > 200 RFU
Ladder Size (bp): 1, 100, 200, 300, 400, 500, 600, 700, 800, 900, 1000, 1200, 1500, 2000, 3000, 6000
Quantification Using: Ladder Final Concentration (ng/uL): 0.0830 Dilution Factor: 12.0

Copyright 2015 Advanced Analytical Technologies, Inc.

Sample: DNA Size Ladder
Well Location: H12
Created: Friday, June 21, 2019 2:11:36 PM
Import From: X:\Lopende Opdrachten\Diagnostics\BRAVO targeted Seq\103291-065 Exp92170\Sample prep\PrepQC_103291-065_1033
Fit Type: Point to Point

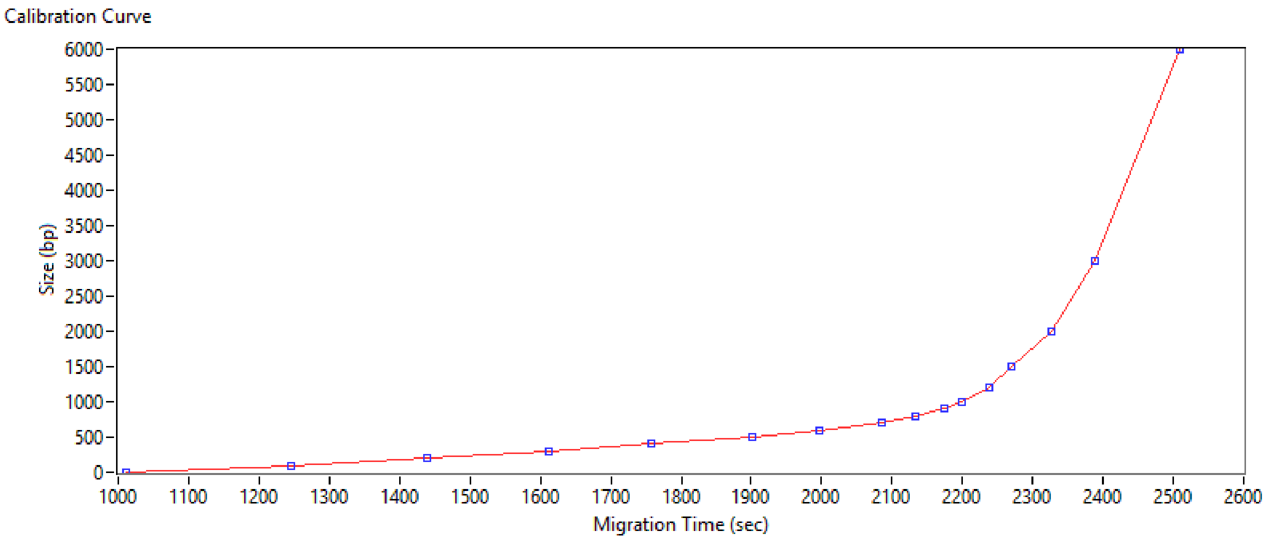

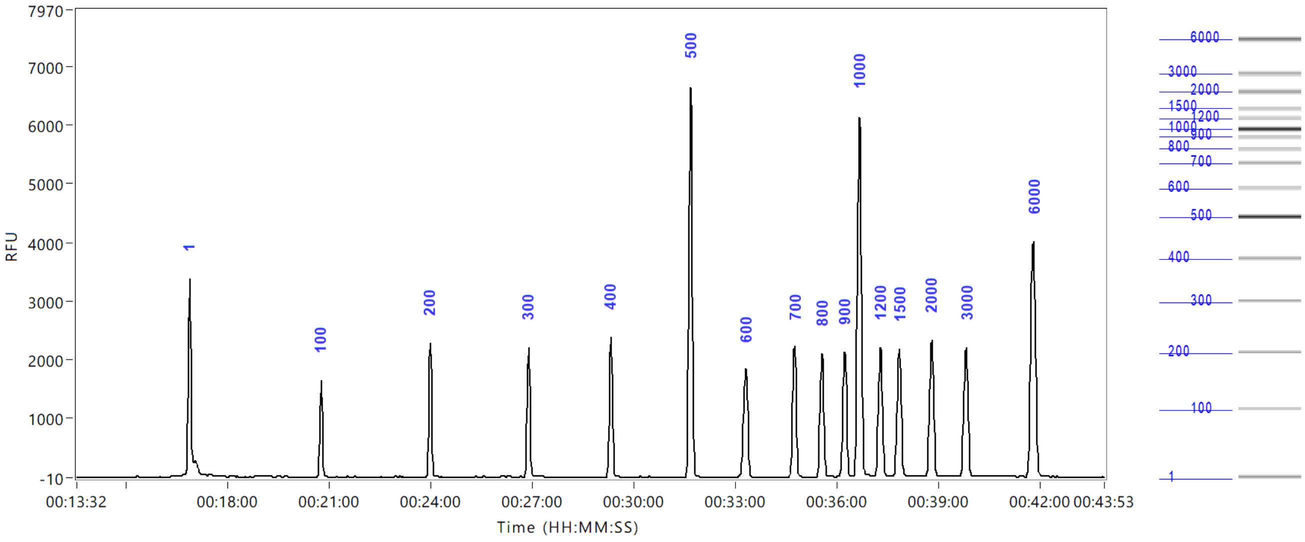

Copyright 2015 Advanced Analytical Technologies, Inc.
